# Of mice and men: the host response to influenza virus infection

**DOI:** 10.1007/s00335-018-9750-y

**Published:** 2018-06-15

**Authors:** Heike Kollmus, Carolin Pilzner, Sarah R. Leist, Mark Heise, Robert Geffers, Klaus Schughart

**Affiliations:** 1 Department of Infection Genetics, Helmholtz Centre for Infection Research, Brunswick, Germany; 2 Department of Epidemiology, University of North Carolina at Chapel Hill, Chapel Hill, USA; 3 Department of Genetics, University of North Carolina at Chapel Hill, Chapel Hill, USA; 4 Helmholtz Centre for Infection Research, Genome Analytics Research Group, Brunswick, Germany; 5 University of Veterinary Medicine Hannover, Hannover, Germany; 6 Department of Microbiology, Immunology and Biochemistry, University of Tennessee Health Science Center, Memphis, TN USA

## Abstract

Influenza virus (IV) infections represent a very serious public health problem. At present, no established biomarkers exist to support diagnosis for respiratory viral infections and more importantly for severe IV disease. Studies in animal models are extremely important to understand the biological, genetic, and environmental factors that contribute to severe IV disease and to validate biomarker candidates from human studies. However, mouse human cross-species comparisons are often compromised by the fact that animal studies concentrate on the infected lungs, whereas in humans almost all studies use peripheral blood from patients. In addition, human studies do not consider genetic background as variable although human populations are genetically very diverse. Therefore, in this study, we performed a cross-species gene expression study of the peripheral blood from human patients and from the highly genetically diverse Collaborative Cross (CC) mouse population after IV infection. Our results demonstrate that changes of gene expression in individual genes are highly similar in mice and humans. The top-regulated genes in humans were also differentially regulated in mice. We conclude that the mouse is a highly valuable in vivo model system to validate and to discover gene candidates which can be used as biomarkers in humans. Furthermore, mouse studies allow confirmation of findings in humans in a well-controlled experimental system adding enormous value to the understanding of expression and function of human candidate genes.

**Electronic supplementary material:**

The online version of this article (10.1007/s00335-018-9750-y) contains supplementary material, which is available to authorized users.

## Introduction

Each year, about 500 million people are infected worldwide by the influenza virus (IV) type A and B, of which about 500,000 die (Fauci 2006). For the diagnosis and treatment of severe IV infections, it is important to determine the infection status and the status (timing and quality) of the host response. Detection of virus in nasal swaps by rapid influenza diagnostic tests or by polymerase chain reaction tests is the first step to diagnose an IV infection. However, detection of virus per se in these swabs is not very informative for treatment decisions. It does not provide information on the level of virus replication in the lung, which is most relevant for severe IV disease nor does it allow clinicians to evaluate the degree or quality of the host response. For example, hypoxia is a hallmark for severe IV infections, but it represents a very late stage marker of disease. Therefore, a significant need exists for earlier biomarkers that have prognostic value for predicting the development of severe IV disease. Furthermore, whether an individual is suffering from viral or bacterial pneumonia has important implications for treatment; however, it is often difficult to differentiate between the two types of pneumonia using current diagnostic methods (Parnell et al. 2012). Therefore, more and better biomarkers are needed that allow diagnosis of disease driven by IV infection, bacterial co-infection, or the infection-induced host response.

Transcriptional or proteomic signatures hold promise for identifying early prognostic biomarkers that can be used to predict patient outcome during IV infection, and we and others have performed transcriptome and proteome studies to identify virus-specific signatures in IV-infected patients (Herberg et al. 2013; Huang et al. 2011; Ioannidis et al. 2012; Marion et al. 2016; Parnell et al. 2012; Ramilo et al. 2007; Suarez et al. 2015; Tang et al. 2017; Thach et al. 2005; Tsalik et al. 2016; Woods et al. 2013; Zaas et al. 2009; Zhai et al. 2015). Now, there is an urgent need to validate these markers and to understand the biological context of their expression, so that these findings can be applied to the clinic. However, due to difficulties associated with performing mechanistic validation studies in humans, it will be important to compare findings in well-controlled animal models to the results in humans.

The mouse model has been instrumental in evaluating the virulence and the pathogenic mechanisms of IV infections and the associated host response (e.g., Kollmus et al. 2014). Studies in genetically well-defined mouse populations have shown the importance of genetic background for susceptibility and resistance (Boon et al. 2009; Ferris et al. 2013; Leist et al. 2016; Srivastava et al. 2009; Trammell et al. 2012). In particular, the highly genetically diverse Collaborative Cross (CC) genetic reference population (GRP) represents an ideal tool to study phenotypic diversity which is caused by genotypic variations (Collaborative Cross Consortium 2012). The CC is derived from eight genetically diverse founder strains, which were intercrossed and then inbred to produce a reproducible population of recombinant inbred (RI) strains where each strain is a genetic mosaic of the original eight founder strains (Collaborative Cross Consortium 2012). Importantly, the CC strains exhibit more than 40 million single-nucleotide polymorphisms and many insertions, deletions, and structural variations, which is comparable to the genetic diversity in human populations (Collaborative Cross Consortium 2012). Furthermore, this diversity is evenly distributed across the genomes of the CC at the population level. In addition, the CC represents an ideal GRP for the mapping of quantitative traits to identify genetic variations that contribute to the host response to IV infections which may be highly relevant for severe course of IV infections in humans (Collaborative Cross Consortium 2012; van Sluijs et al. 2017).

Biomarker studies in humans are mainly limited to the analysis of readily available body fluids. For respiratory infections, nasal swabs or blood is the most preferable source for diagnosis. For severe infections, blood is more preferable than nasal swabs since the critical infection is taking place in the lung. Therefore, almost all current human studies have analyzed the blood. On the other hand, almost all studies in mice investigate the infected organ (lung for IV) because they aim to understand the biological mechanisms at the site of infection. This raises an important caveat; comparisons between responses in IV-infected mouse lungs are of limited value to validate biomarkers that have been identified in human peripheral blood or vice versa.

The value of extending mouse studies to humans was further called into question by a study that compared inflammatory responses after burns, trauma, and endotoxemia in mouse and human blood (Seok et al. 2013). In this study, which has received a significant amount of attention in the media, the authors concluded that the changes in the transcriptome observed in human patients could not be replicated in mice. However, these conclusions have been challenged later (Takao and Miyakawa 2014). It is also very important to note that Seok et al. used only a single inbred mouse strain for their model, C57BL/6J, while the human samples came from patients with different genetic backgrounds (Seok et al. 2013). Therefore, to truly assess whether mouse models can be used to identify and validate biomarkers that are relevant to human infectious diseases, it will be important to carefully compare responses in the appropriate tissues, in this case the blood, while also accounting for the impact of host genetic variation. Furthermore, since these types of responses are likely to be specific for individual pathogens or classes of pathogens, it will also be important to extend this analysis to viral pathogens such as IV.

Here, we analyzed the peripheral blood transcriptome as an indicator of the host response after IV infection in different CC strains and compared it to blood transcriptome changes in human patients infected with IV. We show that the global responses are heterogeneous both in mice and humans and that in the mouse model, genetic background plays an important role for the severity of IV disease and that it strongly influences the transcriptional response. Furthermore, at the single gene level, responses in mouse models and human patients are very similar or even identical. In conclusion, our results support the use of experimental mouse models to validate findings in human IV studies, to suggest additional biomarkers, and to better understand the biology and environmental factors that drive disease severity and transcriptional responses in the host.

## Results

### Highly variable phenotype in the host response to IV in different CC strains

Here, we compared the host transcriptome responses after IV infection in the blood of human patients and experimental mouse models. Since humans are genetically diverse, we also chose to compare these responses to the CC due to the high levels of genetic diversity present in this genetically complex mouse genetic reference population. In this study, we chose eleven CC strains that, at the time, were readily available from the resource at the University of North Carolina (http://csbio.unc.edu/CCstatus/index.py). These eleven CC strains were highly inbred (4–12% at residual heterozygosity). Nine carried genomic regions from all eight founder strains. Three strains, CC019, CC041, and CC051, contained genomic regions of six founder strains. Together, the eleven strains exhibited a good representation of all eight founder haplotypes across the genome (Fig. S1).

**Fig. 1:**
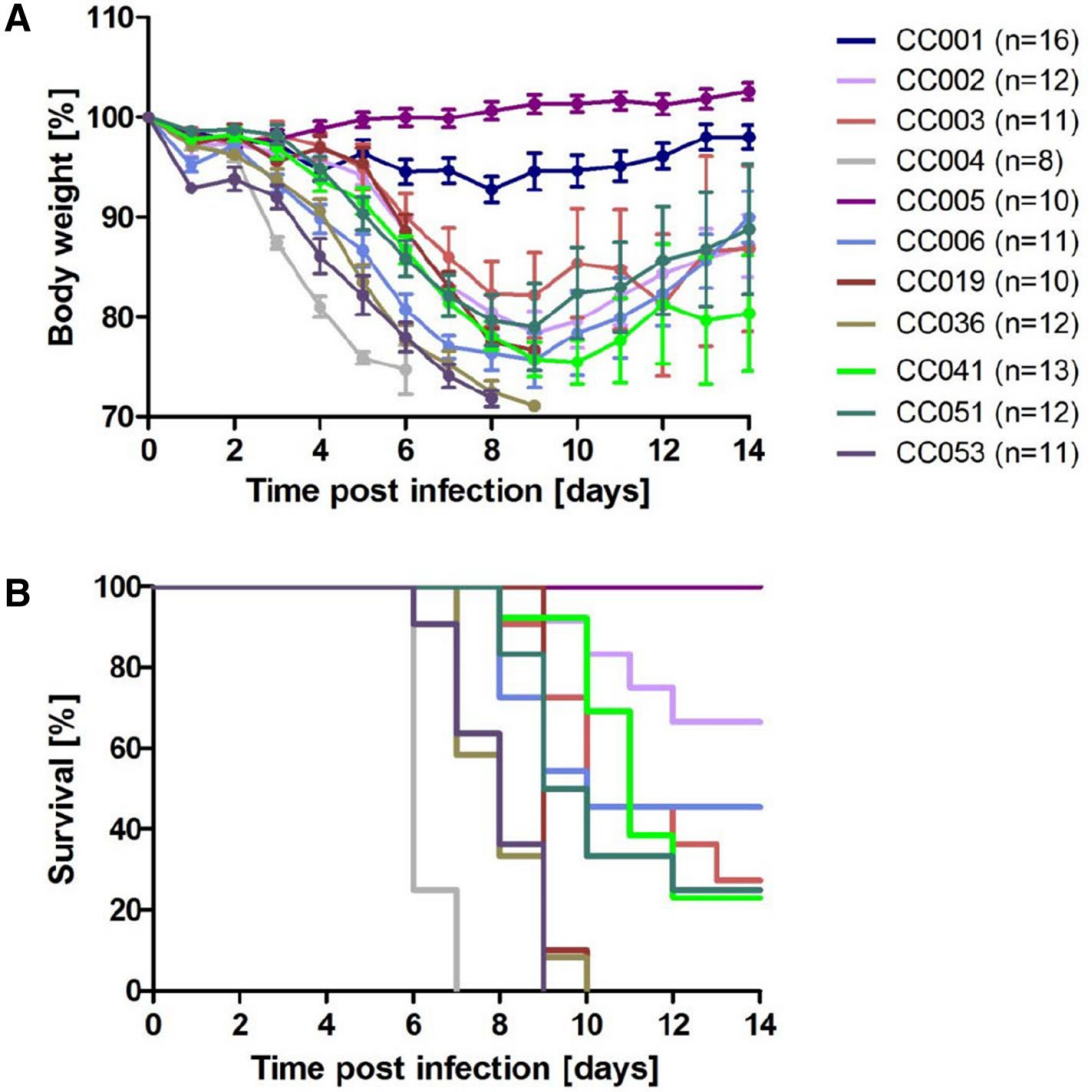
Highly divergent changes in body weight and survival rates of CC strains after infection with influenza A H3N2 virus. Eight to twelve weeks old female mice of the indicated CC strains were infected intra-nasally with 10 FFU of the mouse-adapted influenza H3N2 virus (A/HK/01/68). **a** Changes in body weight and **b** survival were monitored for 14 days pi. Mice that exhibited a weight loss of more than 30% relative to their starting weight were euthanized and scored as dead

Female CC mice (*n* = 8–16) at the age of 8–12 weeks were infected with 10 focus forming units (FFU) of mouse-adapted H3N2 virus. Changes in body weight (BW) and survival were followed over the next 14 days post infection (pi). As shown in Fig. 1a, large variation of BW loss and survival ratios was observed across the 11 strains. ANOVA revealed a significant effect of strain and the interaction of strain and day (repeated measures using data from day 0 to day 5 when all mice from all strains were still alive, model: BW ~ strain × day). Broad sense heritability was 62% for the day 5 BW values. Two strains, CC001 and CC005, were resistant to the infection: all infected mice survived (Fig. 1b). All other strains exhibited a severe phenotype (> 15% of infected mice died). We refer to these strains as ‘severe’ strains in this manuscript. Table 1 describes the CC strains, their susceptibility category, and group sizes used in our study.

**Table 1:** Classification of strains and number of mice per group

CC strain	Category	Number of mice for BW & survival	Number of mice for Mock_day3	Number of mice for Day_3	Number of mice for Day_5	Number of mice for Day_8
CC001	Resistant	16	3	3	3	4
CC002	Severe	12	3	3	3	3
CC003	Severe	11	3	3	3	5
CC004	Severe	8	3	3	3	0
CC005	Resistant	10	3	3	3	3
CC006	Severe	11	3	3	3	0
CC019	Severe	10	3	3	5	3
CC036	Severe	12	3	3	3	0
CC041	Severe	13	4	4	3	3
CC051	Severe	12	3	3	3	3
CC053	Severe	11	3	3	3	0

These results demonstrate that the large genetic diversity of CC strains results in highly diverse IV response phenotypes. They also revealed that susceptibility and resistance after IV infection is a trait that is strongly influenced by genetic background. Humans also exhibit high levels of variation in susceptibility and response to infections. Therefore, we hypothesize that a major part of this variation is caused by genetic variation, and that sets of CC strains should better represent human IV infectious disease than studies using a single inbred mouse strain.

### Global blood transcriptome response in mice and human after IV infection leads to similar separation/overlap between infected and control groups

Based on the variation in IV-induced disease observed in the CC strains, we compared responses to IV infections in mouse and human by analyzing transcriptome changes in whole blood from infected CC strains and human patients. For this, we collected peripheral blood from infected CC mice at days 3, 5, and 8 pi and performed expression array studies. This experimental setting is very important for cross-species comparisons, since studies in humans mainly use peripheral blood, and we found that changes in transcriptomes were very different in lung and blood (Fig. S2, blood data are from this study, lung data are taken from Leist et al. 2016). For example, many more DEGs were observed in lung compared to blood which is most likely due to the fact that the lung is the primary organ of infection and that the cell composition is much more complex in lung (lung tissue plus immune cell infiltrates) than blood. In addition, mice that were mock infected with PBS served as controls, which allowed us to exclude the influence of the infection procedure at early time points after infection on gene expression changes (Preusse et al. 2013). This contrasts with human studies where patients are already symptomatic at the time of analysis in field studies.

The results were then compared to transcriptome changes in peripheral blood of human patients from data sets that we downloaded from public databases. We chose three publically available human data sets from the GEO gene expression database (http://www.ncbi.nlm.nih.gov/geo/). The first study was composed of human volunteers infected with H1N1 and H3N2 virus (GSE52428 Woods et al. 2013), a second field study compared healthy controls with IV-infected patients (GSE68310 Zhai et al. 2015), and the third data set is derived from our own study (GSE82050 Tang et al. 2017). These data sets were chosen because they were publically available and each of them had a reasonably large group size of infected patients and controls (*n* ≥ 15 for each). GSE52428 represents a study with controlled infections of human volunteers and GSE68310 has analyzed the currently largest group of IV-infected patients (*n* > 45 depending on the day pi). Both data sets followed transcriptome changes over time with comparable times to our mouse data (day 2–day 4.5 pi for GSE52428 and days 2, 5, and 8 pi for GSE68310). Furthermore, our own study (GSE82050) identified *IFI27* as one of the most strongly up-regulated genes, and we suggested it as a marker for IV and other respiratory viral infections (Tang et al. 2017).

Here, we aimed to compare mouse to human host responses at early time points after infection since these are most relevant for diagnosis and treatment decisions in human patients with the risk for severe infections. Therefore, we selected only the relevant time points (until day 8 pi) from the above data sets. From the GSE52428 data set, baseline values (before infection challenge) was selected as control group, and 45.5 h pi (about day 2) and 93.5 h pi (about day 4) for the infected groups. Also, we only selected samples from infected participants that showed symptoms. This data set was named Woods_13. For GSE68310, we selected only the samples that were derived from IV-infected patients and from early time points after infection. Baseline (taken prospectively at enrollment before occurrence of symptoms) values were selected as controls and day 0, day 2, and day 4 (day 0: first visit within 48 h of occurrence of symptoms) as infected groups. This data set was named Zhai_15. From GSE82050, the entire data set was used and named Tang_17. Table 2 provides a description of all data sets used in our study with the respective time points and group sizes and Tables S1–S4 list all samples.

**Table 2:** Description of data sets for PCA, DEGs, and boxplot analyses

Data set name	Reference GEO data set	Selected time points combined to infected group	Number of individuals in infected group	Selected for control groups	Number of individuals in control group	References
Woods_13	GSE53248	45.5 h, 93.5 h^a^	20	pre-ch-basl	20	Woods et al. (2013)
Zhai_15	GSE68310	Days 2, 4, 6^a^	49^b^	Baseline	48	Zhai et al. (2015)
Tang_17	GSE82050	Day 1^a^	24	hlty_ctr	15	Tang et al. (2017)
CC strains	GSE110384	Days 3, 5, 8^c^	127; 11 strains	Mock	34	This publication

^a^Days of analysis (after infection for Woods_13, for day after hospital admission for Zhai_15 and Tang_17)

^b^Note that not for all 49 patients data were available for all days (baseline: 48, day 2: 49, day 4: 45, day 6: 46)

^c^Day 8 was only analyzed when the survival rate for this strain was higher than 60%

**Fig. 2:**
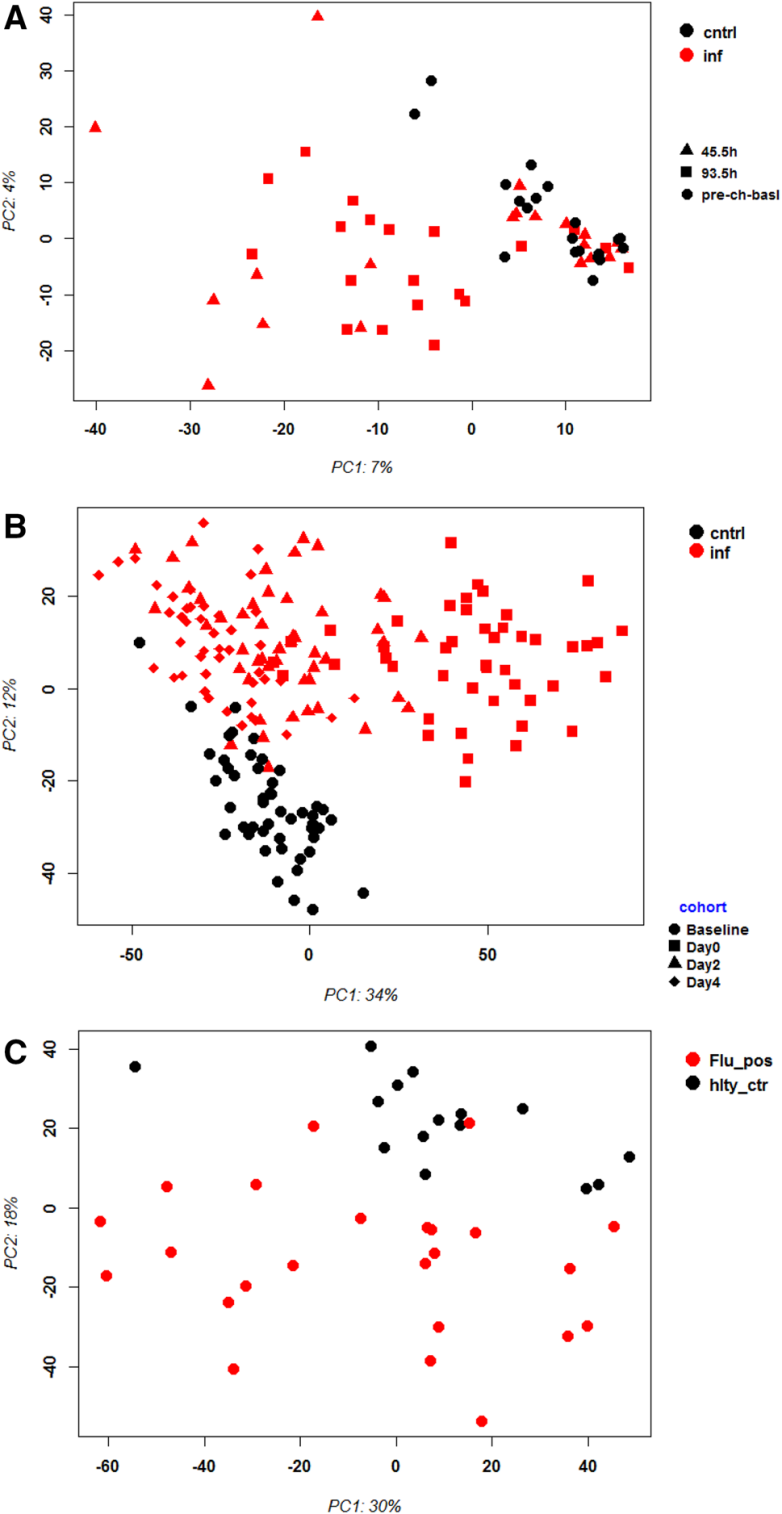
Principle component analysis of gene expression values from blood of patients infected with influenza virus. Principle component analysis (PCA) revealed separate grouping of controls versus infected patients and mice. Black: controls, red: infected; days pi are indicated by symbols. **a** Woods_13, **b** Zhai_15, (C) Tang_17. *cntrl* controls, *inf* infected, *pre_ch_basal* pre-challenge basal, *Flu_pos* infected, *hlty_ctr* healthy controls. Days pi/admission are indicated by symbols. *cntrl* healthy controls, *hlty_ctr* healthy controls, *inf* infected individuals, *Flu_pos* infected individuals

**Fig. 3:**
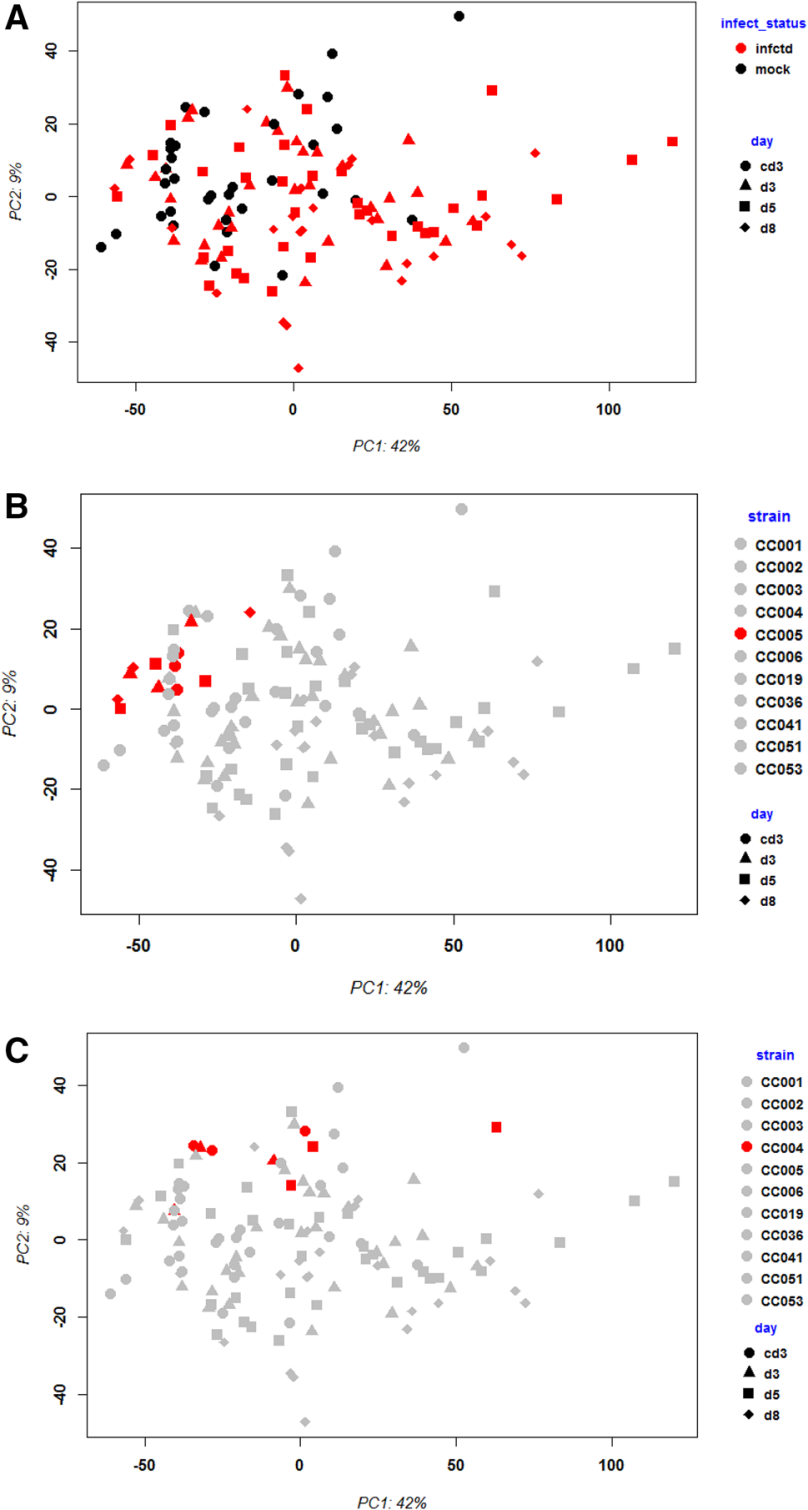
Principle component analysis of gene expression values from blood of CC mouse strains after infection with influenza virus. Principle component analysis (PCA) revealed separate grouping of mock controls versus infected mice. **a** Black: mock-infected controls (mock), red: infected mice (infctd); days pi are indicated by symbols. **b** and **c** The same PCA as in (**a**) highlighting a single CC strain in red; days pi are indicated by symbols. *cd3* mock-treated controls at 3 days after treatment, *d3* day3 pi, *d5* day 5 pi, *d8* day 8 pi

First, we performed principle component analysis (PCA) for controls and infected groups from human and mouse to compare the global changes in gene expression after infection. The analysis was performed with the top 3000 most variant probe sets to avoid too high noise by low level expressed transcripts. Infected and control groups separated in the PCA in all human data sets (Fig. 2a, b, c) as well as for the mouse CC strains (Fig. 3a). However, there was considerable overlap between infected and control groups in all data sets. In addition, there was no clear distinction of groups according to their days pi/collection in any data set. In humans, the factors that may cause the overlap between infected and control groups are not known. They may represent differences in the host response because of vaccine status, infection dose, comorbidities, etc. However, also in the well-controlled mouse studies the overlap between infected and control groups was considerable. In the mouse data set, it was possible to stratify groups with respect to their genetic background. This stratification showed that different CC strains differ largely in their global transcriptome response to IV infections (Fig. 3b, c). For example, the resistant strain CC005 exhibited hardly any change in gene expression and the response was back to control levels at day 5 (Fig. 3b). On the other hand, the highly susceptible CC strain CC004 exhibited a very strong overall response to the infection (Fig. 3c). The PCA showed that day 3 and day 5 pi responses differ largely from baseline responses for this strain.

In conclusion, variation of the host response between case and control groups at the transcriptome level was similarly heterogeneous in mice as in humans. However, in our well-controlled experimental mouse model, a large portion of this variation was due to genetic diversity. It was shown before that genetic background in CC strains and CC founders (Ferris et al. 2013; Leist et al. 2016) strongly influences virus replication in the lung. Our observations in mice suggest that genetic variation also contributes significantly to heterogeneity of susceptibility and host response in humans.

### Many infection-related differentially expressed genes are identical between mouse and human

Next, we identified differentially expressed genes (DEGs) between infected and control groups in human and mouse data sets to determine the overlaps between and within a species. In the human data sets, we combined the various time points after infection to a single infected group and then contrasted healthy controls to infected patients. For the mouse, we also combined the different days pi to a single infected group and mock-infected animals were used as controls. Since there were no severe cases (deaths) in humans, we performed two separate comparisons in CC mice—between infected resistant strains and all controls and between infected severe strains and all controls. Differentially expressed genes (note that some genes are represented by multiple probe sets which were analyzed separately) were defined to exhibit > 1.5-fold difference in expression value (delta log_2_ > 0.585) between infected and controls and a false discovery rate (using BH for multiple testing correction) of FDR < 10%. Based on the outcome of the infections in mice, we distinguished resistant and severe strains (see above). Table 3 shows the number of differentially expressed probe sets for each pairwise comparison of infected versus controls. Detailed lists for all DEGs can be found in the supplementary data (Tables S5–S10). In the human data sets, more down-regulated than up-regulated DEGs were found. This was the inverse for the mouse comparisons. Also in the mouse studies, many more DEGs were detected compared to humans. The reason for this may be that mouse experiments are well-controlled and thereby are less prone to noise from uncontrolled confounding factors as in humans.

**Table 3:** Number of up- and down-regulated differentially expressed probe sets between infected and control groups

Comparison	Dataset	DEG up-regulated	DEG down-regulated
Woods_13	Human	138	302
Zhai_15	Human	23	121
Tang_17	Human	195	1144
CC strains resistant versus controls	Mouse	439	192
CC strains severe versus controls	Mouse	877	507
CC strains severe versus resistant	Mouse	904	1122

**Fig. 4:**
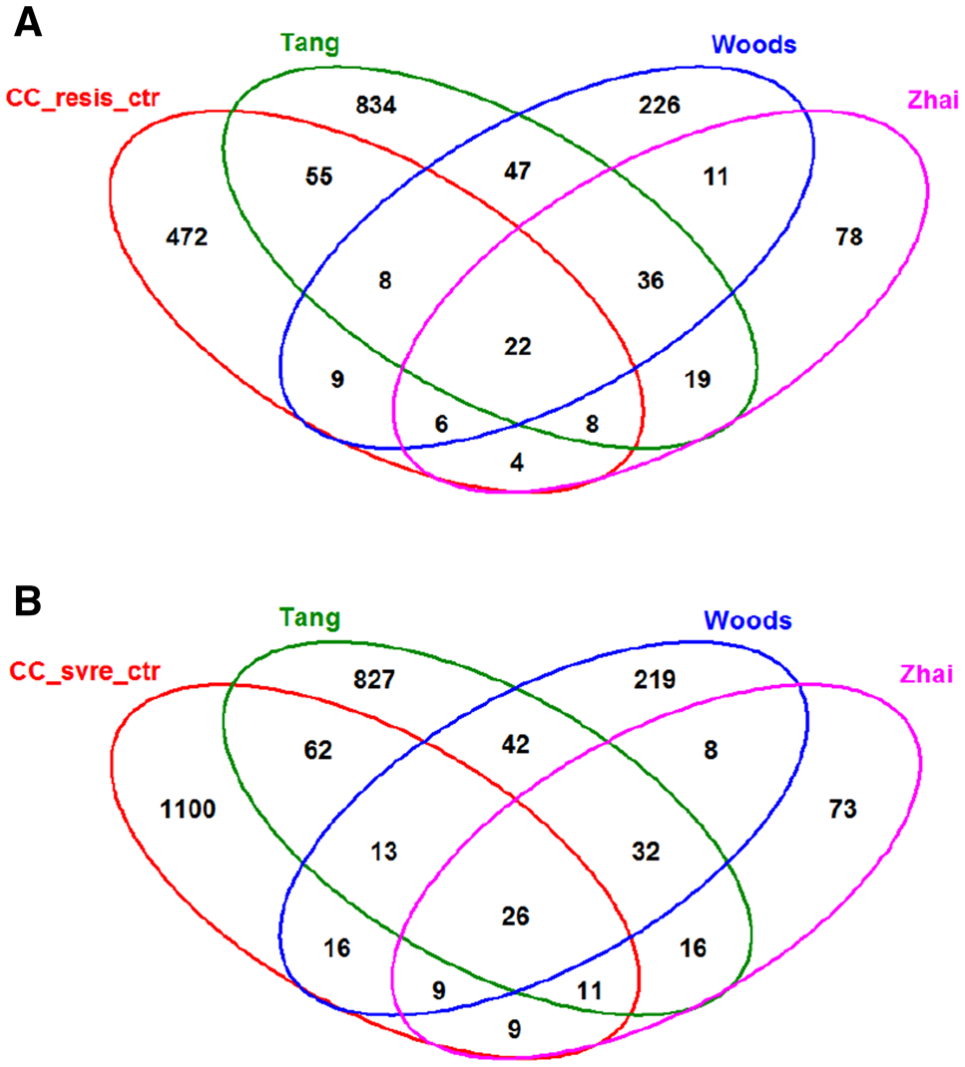
VENN diagram of differentially expressed genes for mouse and human data sets. VENN diagram of differentially expressed genes (DEGs) revealed similar overlaps between human cohorts and between mouse and human data sets. **a** DEGs from human comparisons of infected versus controls, and mouse DEGs from the comparison between controls (mock-infected mice from all strains) and infected mice from resistant CC strains (CC_resis_ctr). **b** DEGs from human comparisons of infected versus controls (datasets indicated by names Tang, Woods, Zhai, respectively), and mouse CC DEGs of the comparison between mock and infected mice from severe CC strains (CC_ svre _ctr)

We then determined the overlap between the DEGs from all of the above comparisons, (Fig. 4a: DEGs from all human data sets with DEGs from mouse resistant versus control; Fig. 4b: DEGs from all human data sets with DEGs from mouse severe versus control). 22 DEGs (*BST2, DHX58, HERC6, IFI27, IFI35, IFI44, IFIH1, IFIT1, IFIT2, IFIT3, IFITM1, IRF7, ISG15, MX1, OAS2, OAS3, PLAC8, RSAD2, RTP4, USP18, XAF1, ZBP1*) overlapped between the human data sets and the mouse resistant versus controls and 26 DEGs (*BST2, DHX58, EIF2AK2, IFI27, IFI35, IFI44, IFIH1, IFIT1, IFIT2, IFIT3, IFITM1, IRF7, ISG15, LY6E, MX1, OAS2, OAS3, PLAC8, PLSCR1, RTP4, SAT1, SERPING1, TAP1, USP18*) overlapped between the human data sets and the mouse severe versus controls DEGs. Many of these overlapping DEGs belonged to the interferon pathway which is activated after viral infection. Each of the mouse and human data sets had a unique number of DEGs that was not shared with any other group. These unique genes are not enriched for any particular annotation across all data sets using the R package clusterProfiler (Yu et al. 2012), suggesting that these unique differences between cohorts are random. Only the CC and Tang_17 shared the annotations “Neutrophil degranulation” and “Antigen processing-Cross presentation” for the unique groups. Most remarkably, this set of unique DEGs in CC mice was not specifically higher compared to the human data sets (80% for CC (unique DEGs divided by total number of DEGs), 62% for Woods_13, 42% for Zhai_15, 81% for Tang_17 in Fig. 4a). Also, the overlap of DEGs between Tang_17 and CC mice (55 DEGs) was similarly large as the overlap between Tang_17 and Woods_13 (47 DEGs; Fig. 4a). Based on these results, we conclude that, at the global level, differences in the identification of DEGs between various human studies are mainly caused by differences between their designs (cohorts, time points pi, technical platforms, *etc*.) and not because of species differences. The overlaps observed for human DEGs between different studies were not higher than between mouse and human.

### Cross-species comparison of the expression of individual DEGs reveals similar changes in mice and human

**Fig. 5:**
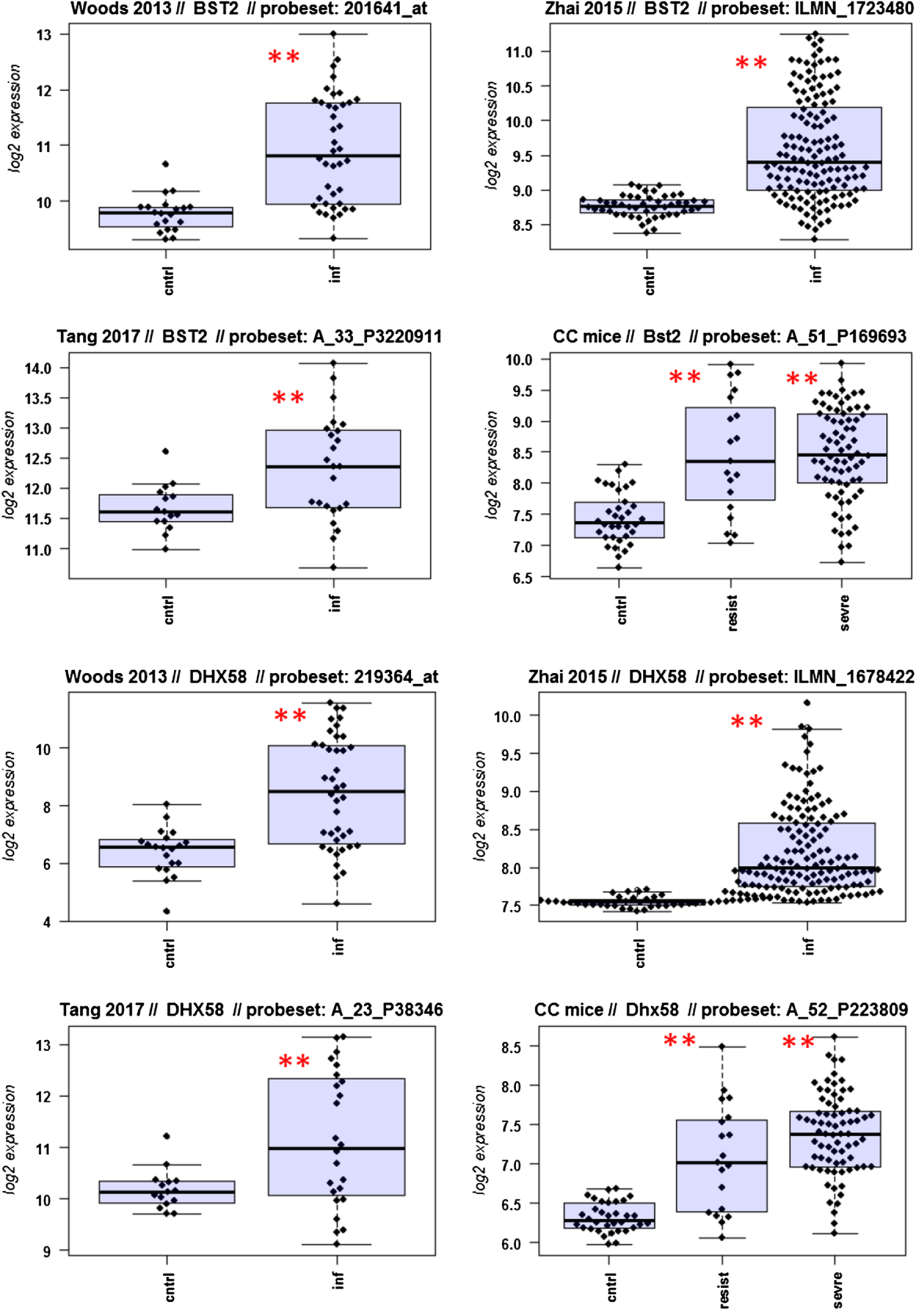

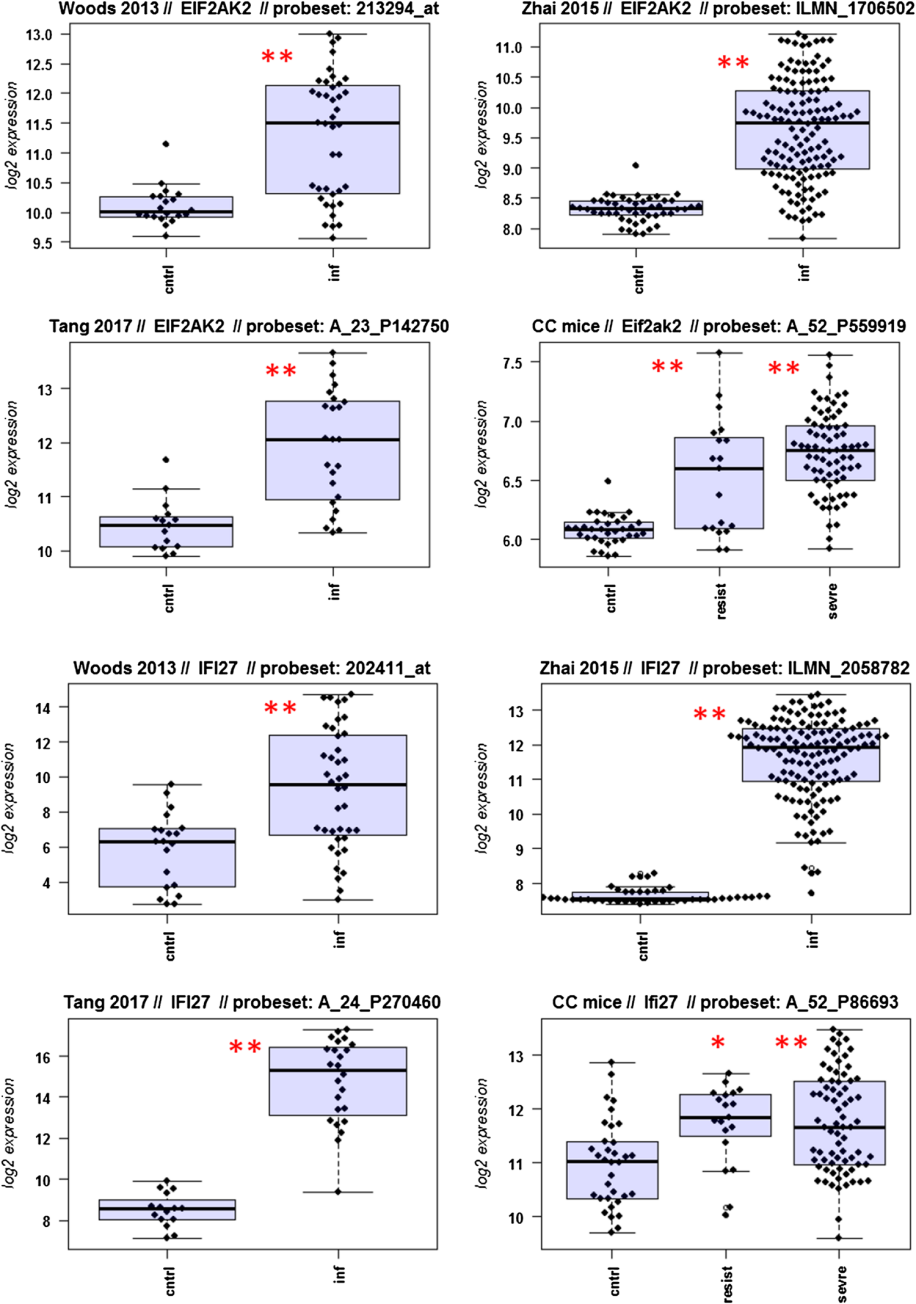

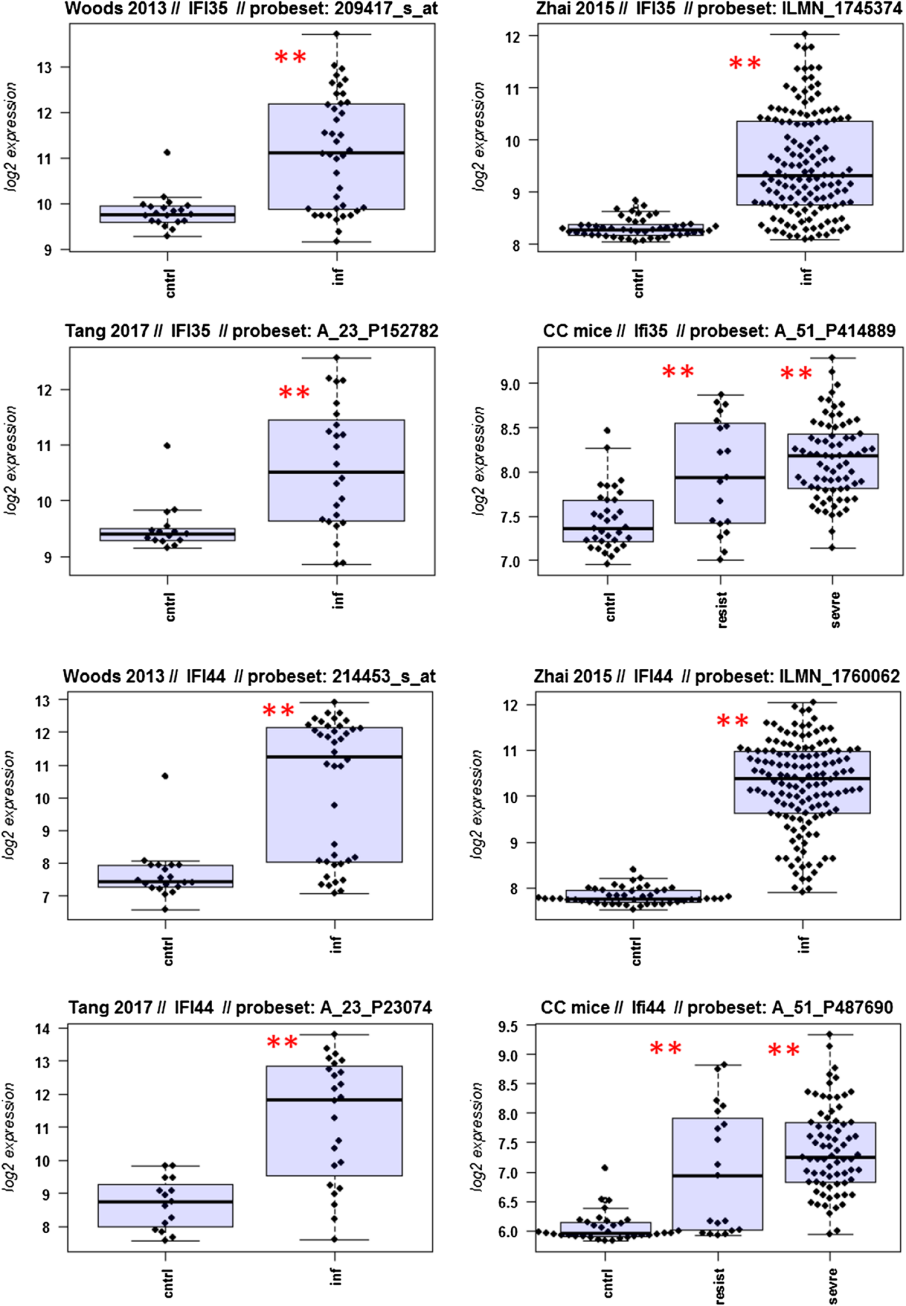

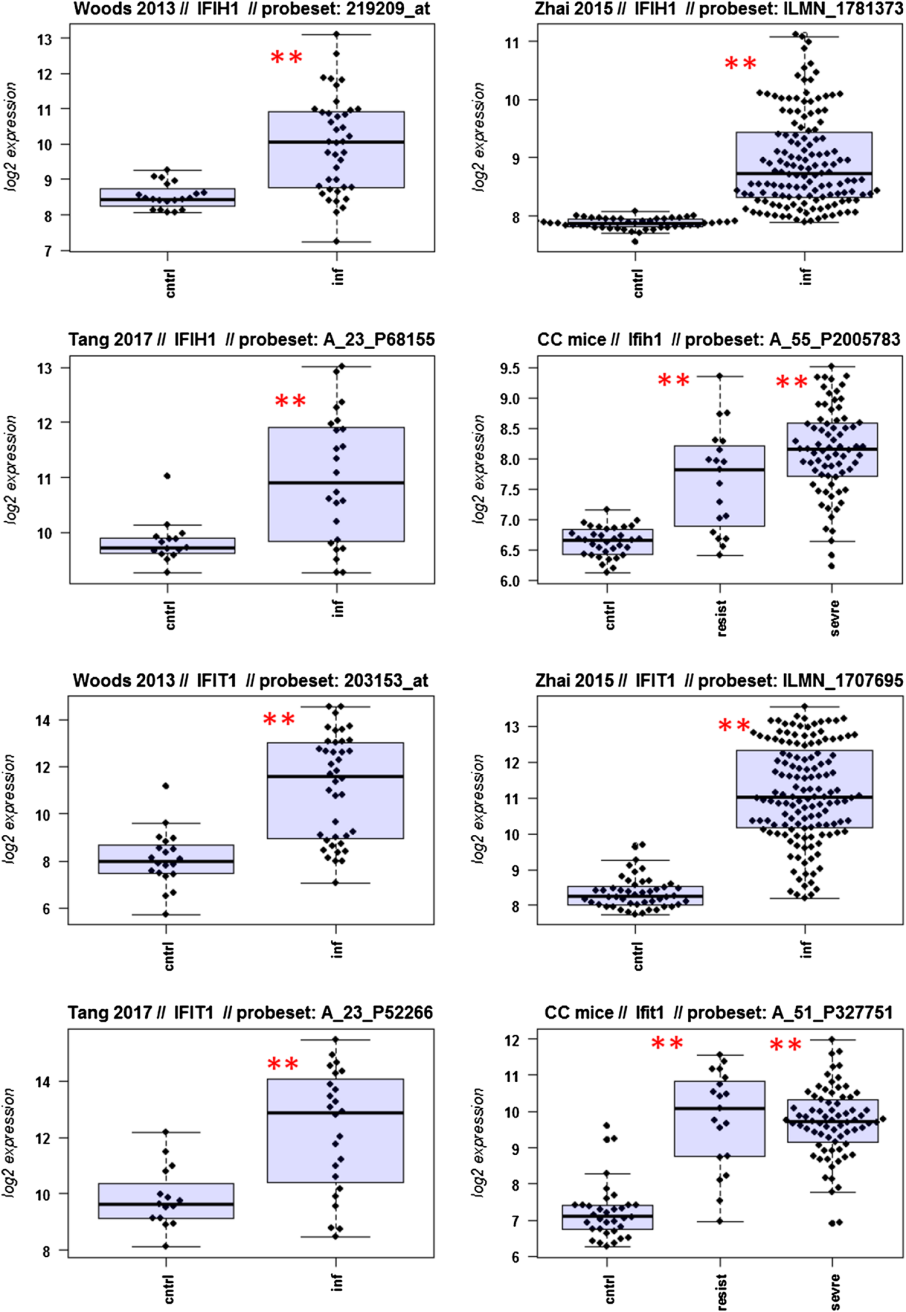

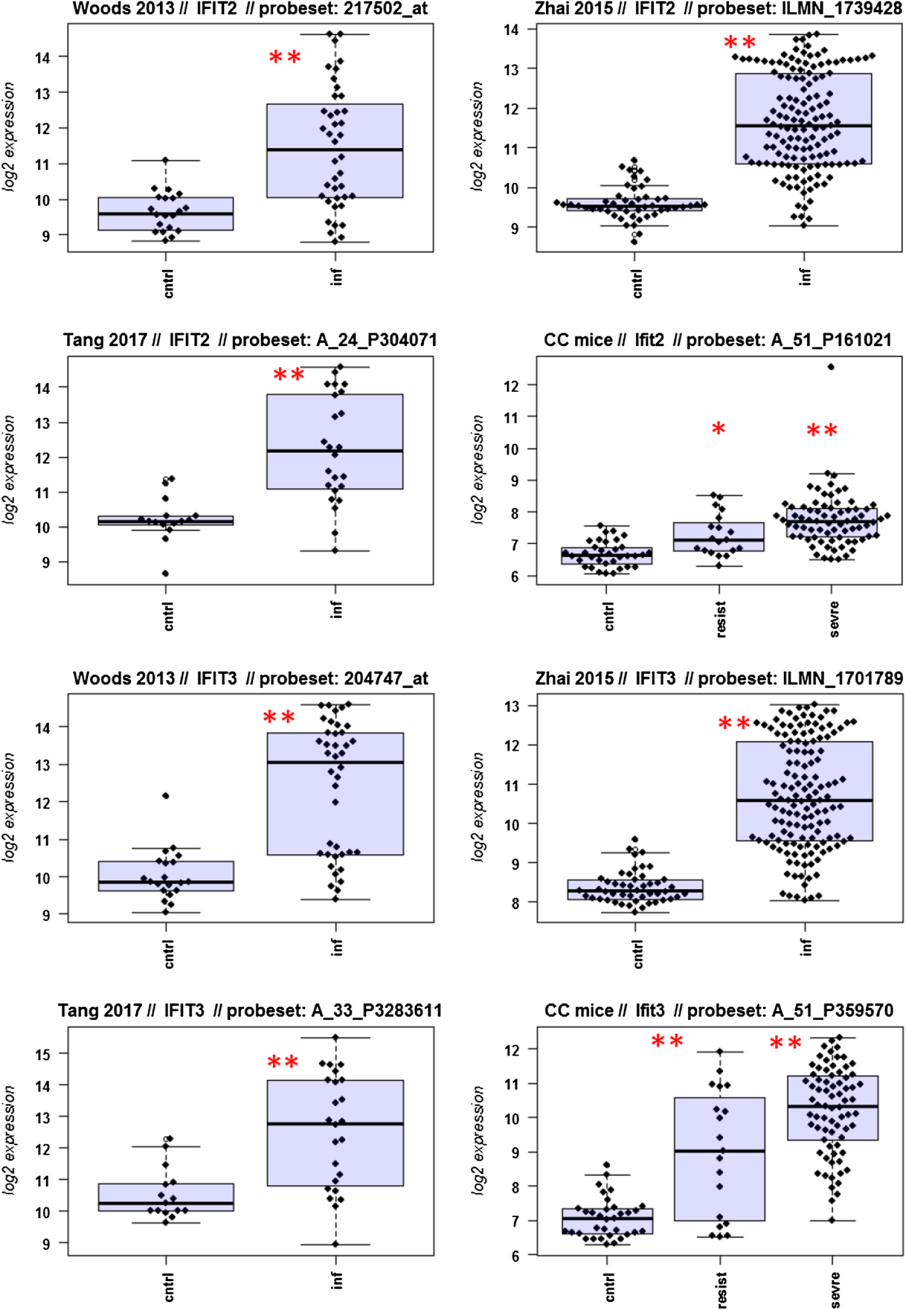

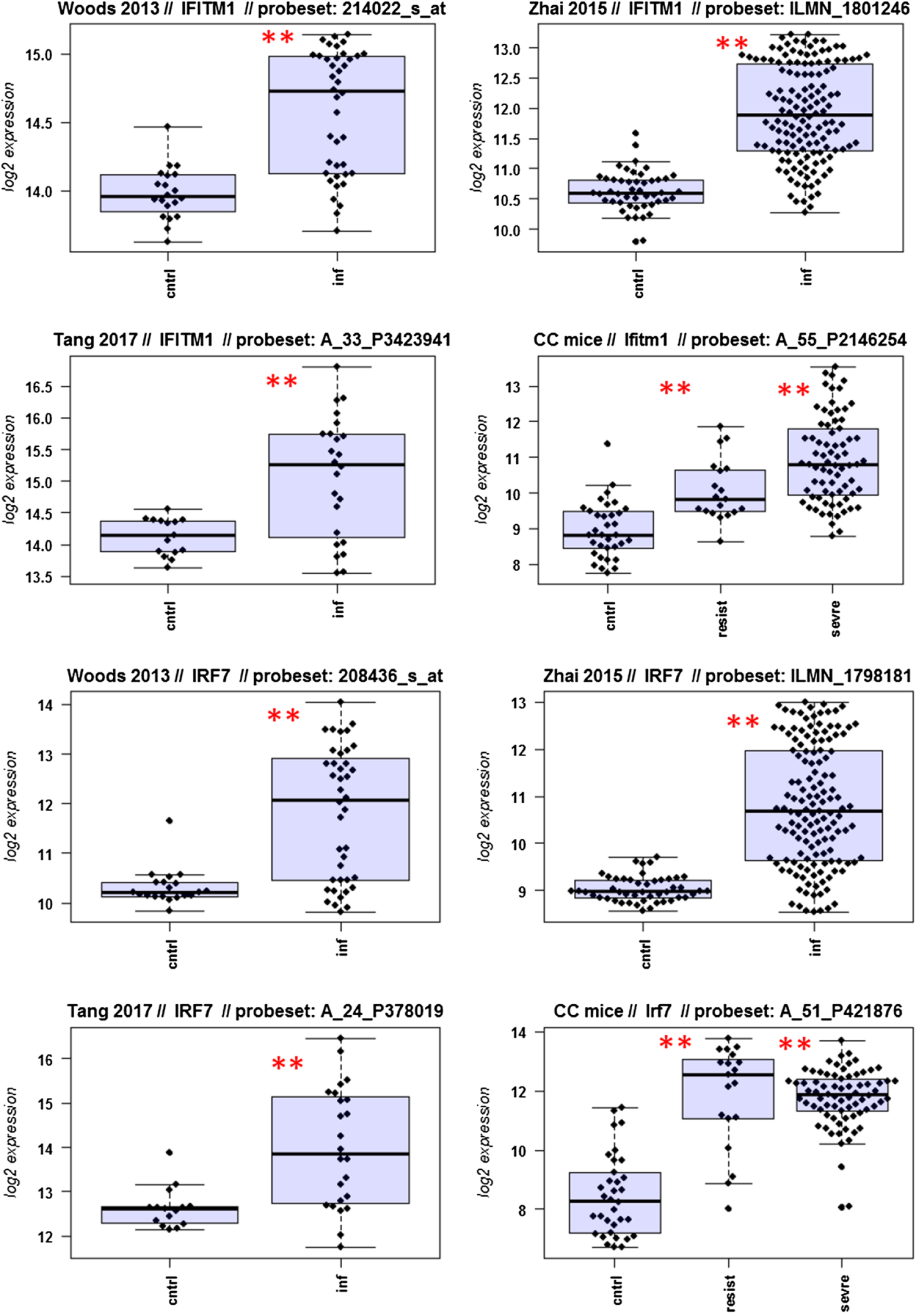

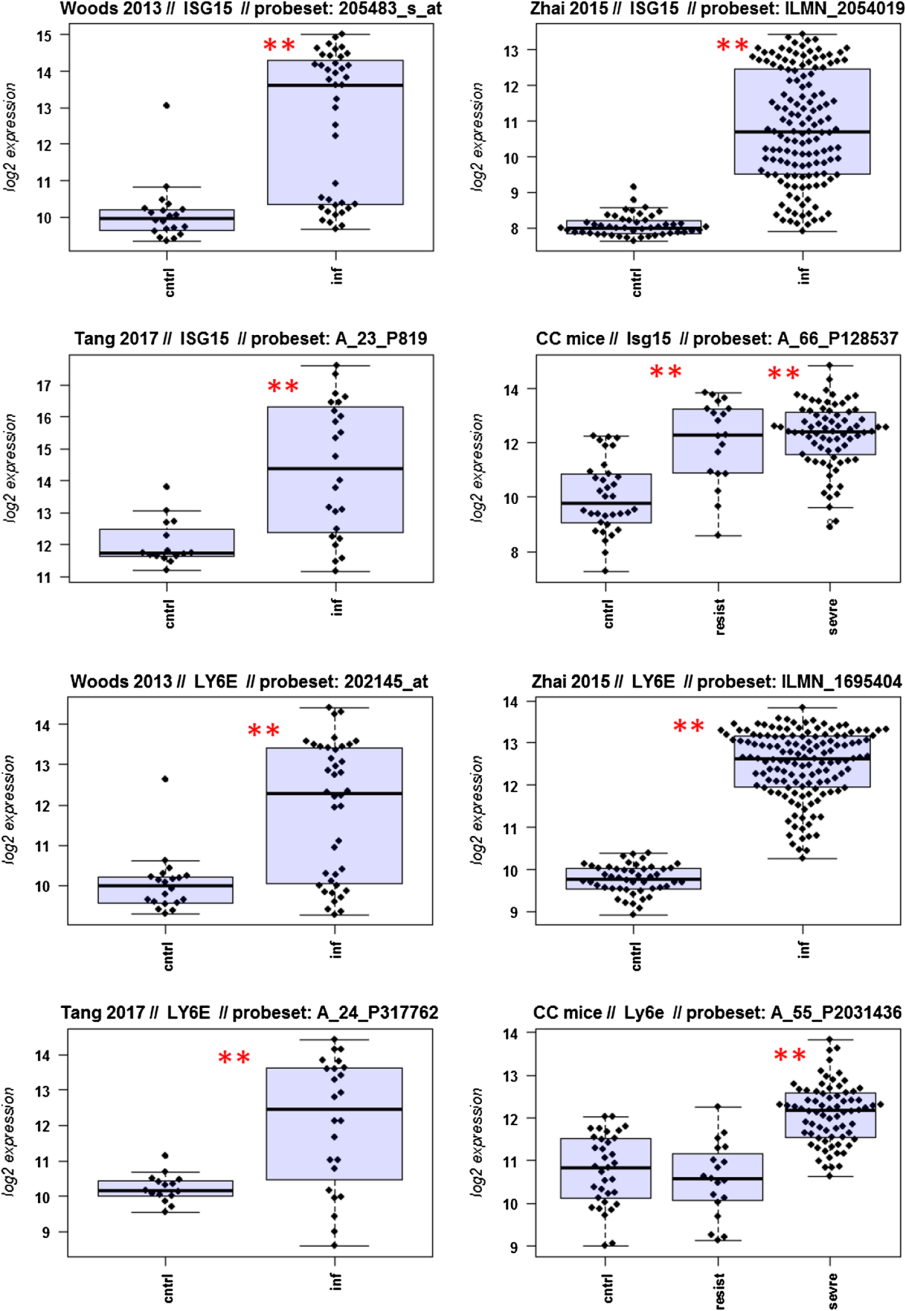

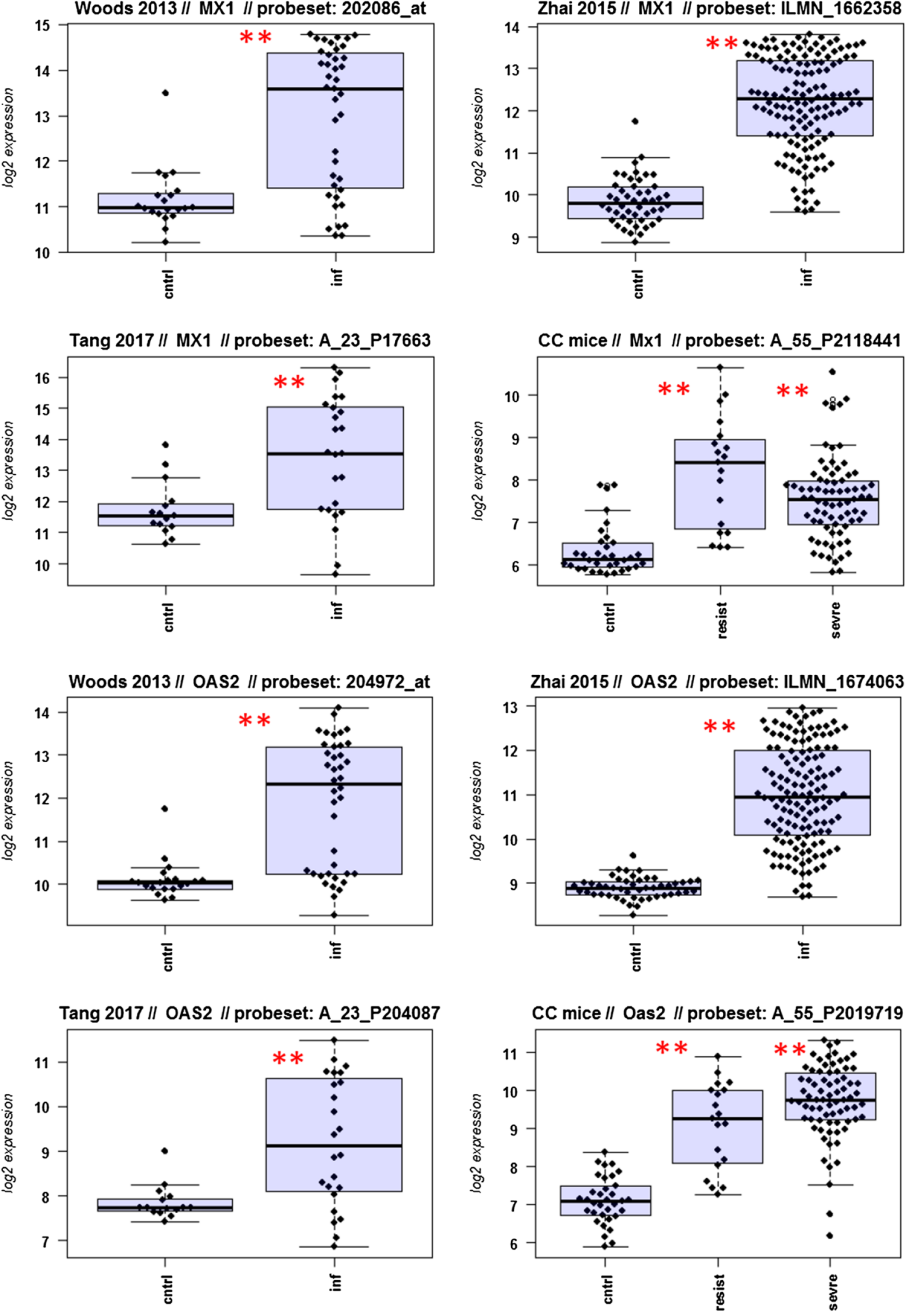

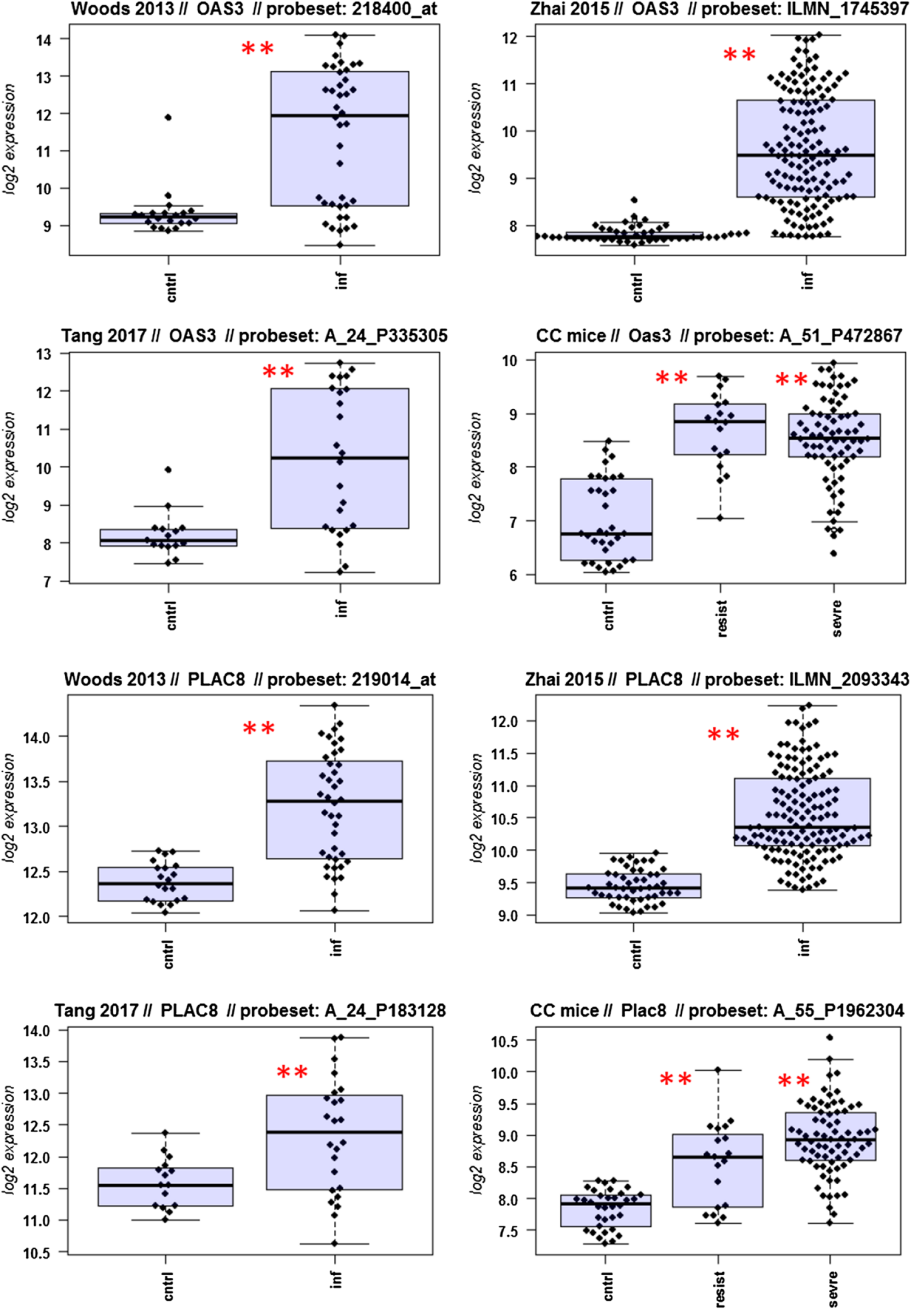

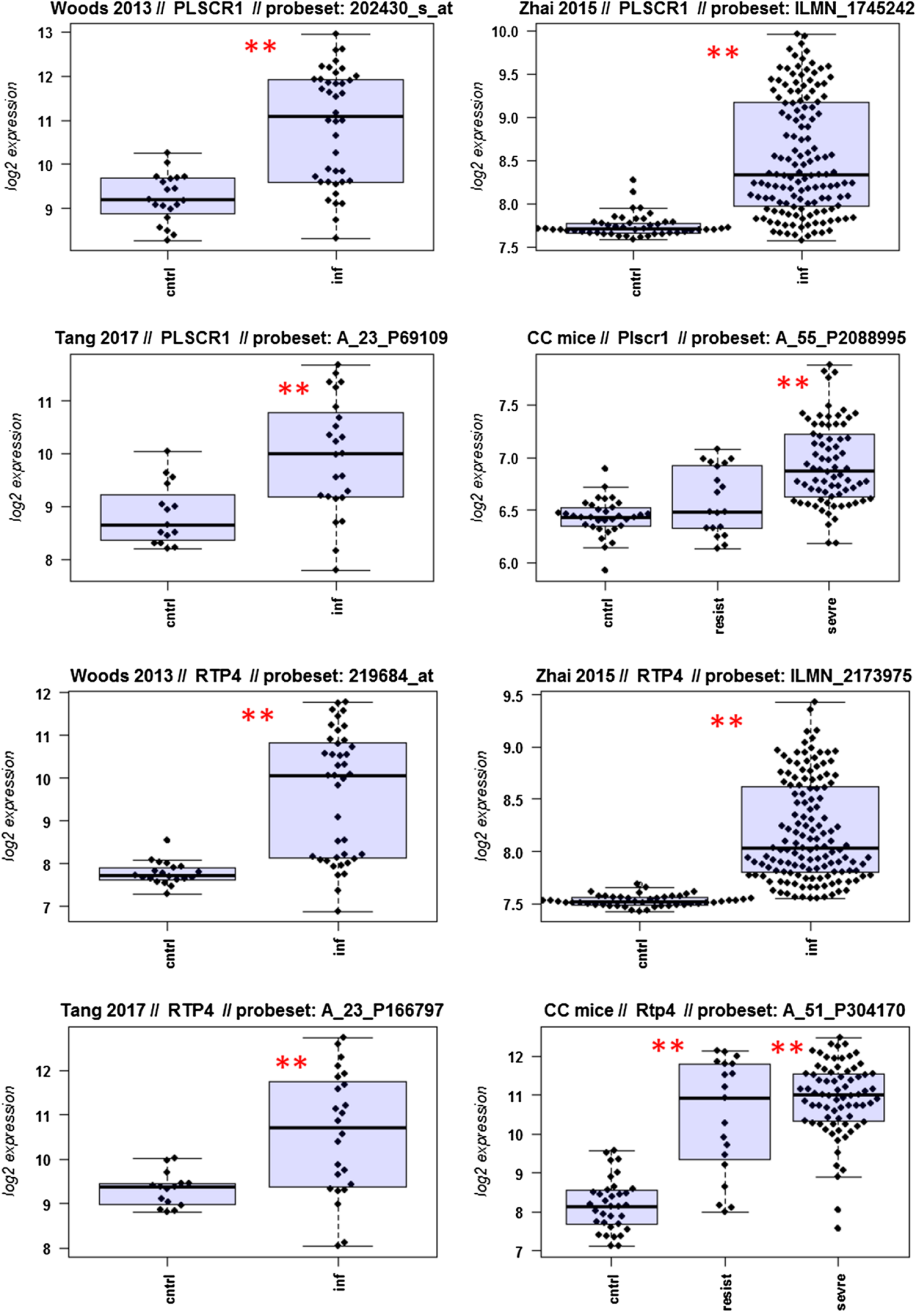

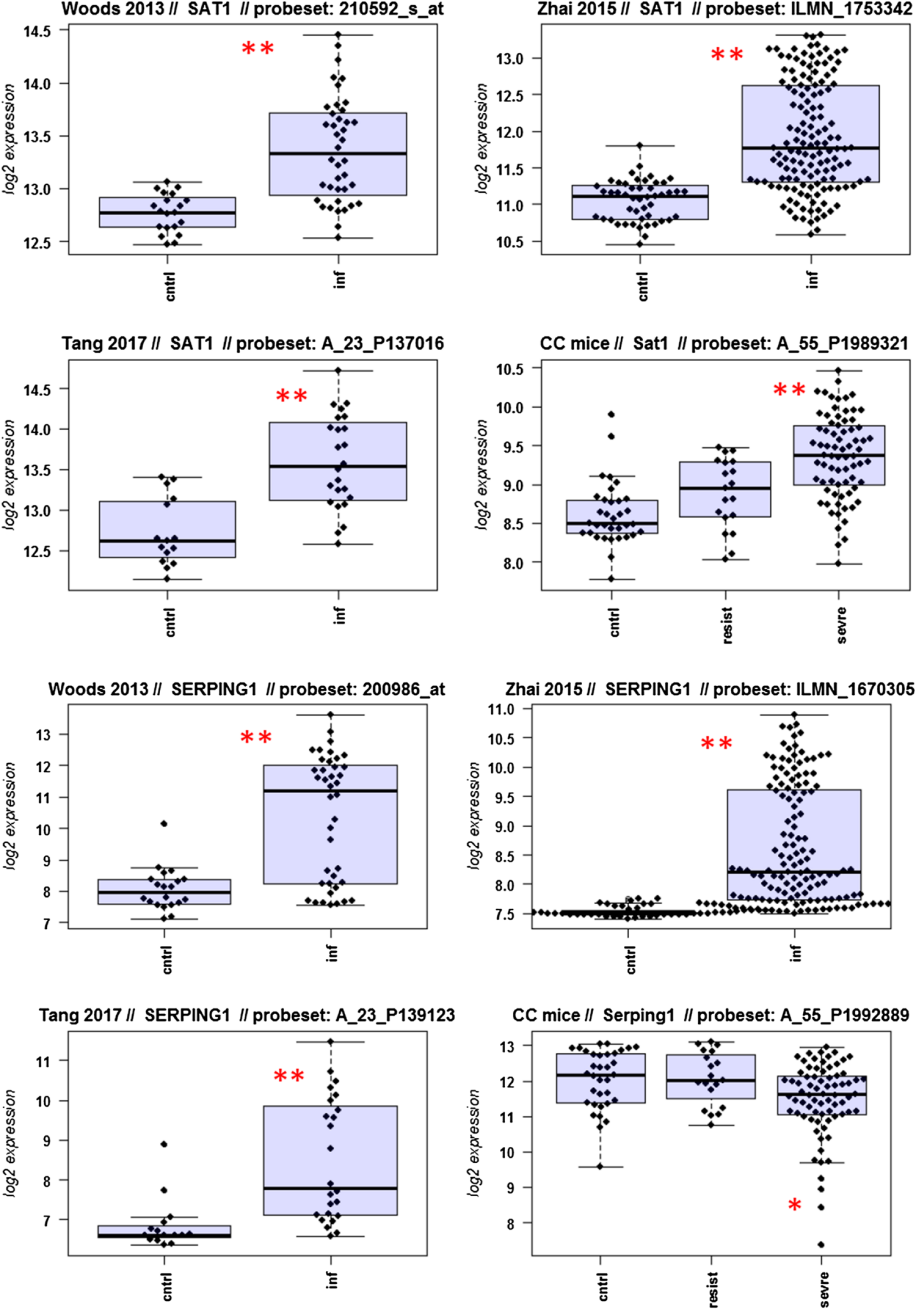

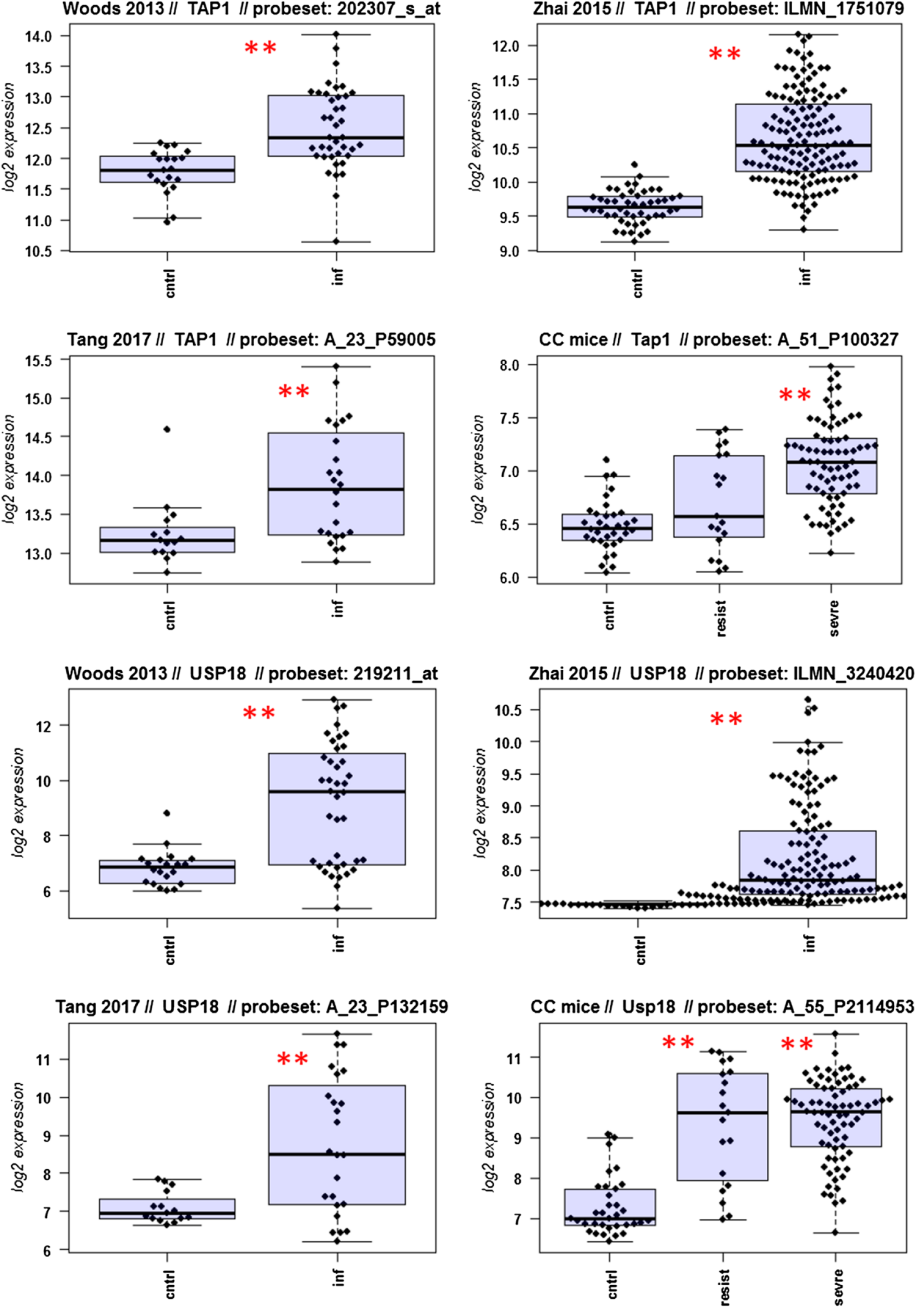

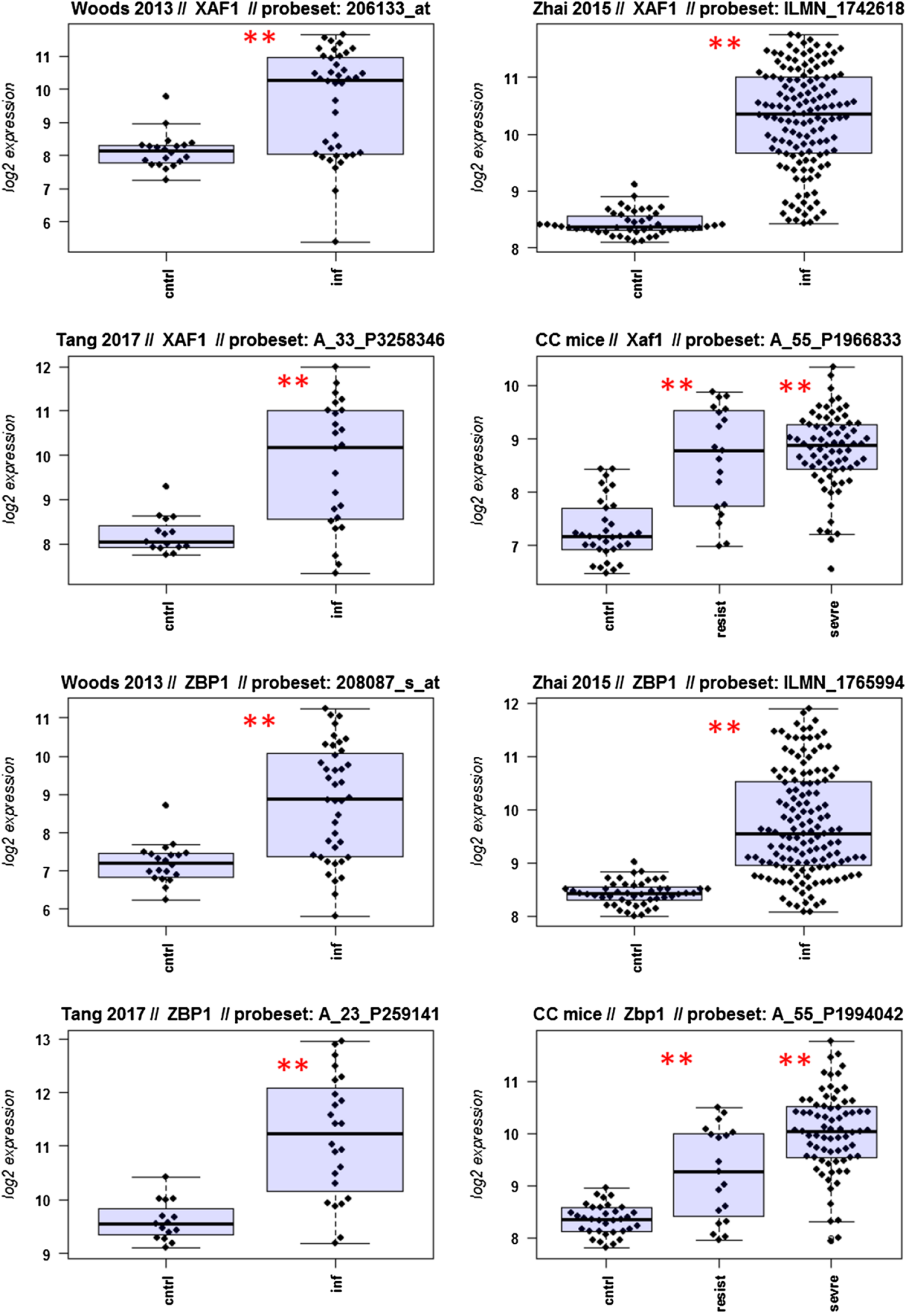
Changes in the expression levels of 26 overlapping differentially expressed genes in human and mouse data sets. Boxplots for individual expression values of normalized log_2_-transformed signal intensities in the blood of human patients and mice for infected individuals and controls are shown. **a** Woods_2013, **b** Zhai_15, **c** Tang_17, **d** CC mouse strains. *cntrl* controls, *inf* infected, mouse: cntrl: mock-treated controls, *resist* resistant mouse strains after infection, sevre: severe mouse strains after infection. Significant *p* values of pairwise comparisons between controls and respective infected groups are indicated as *p < 0.05; **p < 0.01

A major goal in human studies is to identify molecular signatures that can be used as biomarkers for viral versus bacterial infection or severe versus moderate infections. Such biomarkers may be genes that change expression levels as a function of infection (yes/no), pathogen (viral/bacterial), or disease severity (moderate/severe). Therefore, we compared changes in the expression of individual genes between human and mouse to determine how well the mouse model mimics human responses at the single gene level. For this, we performed a comparative analysis of all 26 overlapping DEGs from Fig. 4b (Fig. 5-1 to -26). For the human data sets, we combined the indicated days pi to a single infected group as described above. For the mouse, we combined all resistant strains into a single group, and the severe strains to a second group. Mock-treated animals were used as controls.

Differences in expression between infected and healthy controls in humans were significant for all overlap DEGs. Similarly, all differences between mice with a severe phenotype and mock controls were significant. However, for some genes (*Ly6e, Plscr1, Sat1, Serping1, Tap1*), no significant difference between mice from the resistant group and controls were observed in the mouse studies, whereas differences between the severe group and controls were significantly different. These results demonstrate that at the single gene level, results from mouse and human are highly comparable. In addition, mouse studies revealed differences between strains that exhibited severe influenza disease versus resistant strains. The differences represent valuable candidates to validate in humans with severe versus moderate infection outcomes. Thus, the mouse is a well-suited model for human responses to IV infections at the transcriptome level when the same tissues (blood) in both species and genetically variant mouse populations are used.

### Temporal changes of single differentially expressed genes in mice and human are very similar

A big advantage of controlled animal infection experiments is the possibility to study defined time points after infection in biological replicates, including individuals of the same sex, age, and genetic background. In human studies following natural infection, these variables are often not controlled and at best the time after onset of symptoms is recorded. The study by Woods et al. is an exception. This study analyzed the blood of infected volunteers from 5 to 165.5 h after infection. Thus, this is one of the very few well-controlled analyses with respect to different time points pi. In our analysis, we included only the time points until 108 h for this data set since the later time points had only samples from a single person. Also, we only analyzed symptomatic patients. The study by Zhai et al. recorded also the temporal change in gene expression. In this case, samples were collected from days 0 (first visit within 48 h of occurrence of symptoms) to 21 days after the first visit and also post-seasonal samples after complete recovery (‘spring’). For the Zhai data set, we included all time points from the study. Please note that both human data sets are larger than the ones described above and in Table 1 since they contain additional time points. As an example, we performed an analysis of the kinetics of gene expression changes for genes *IFI27* and *IRF7* in humans and mice. *IFI27*, an interferon-regulated gene, had been proposed as potential marker to distinguish respiratory viral from bacterial infections (Tang et al. 2017). *IFI27* was the top DEG in humans when contrasting expression levels in controls versus infected patients in all three human data sets (this study). *IRF7*, an interferon-regulating transcription factor, is an important host gene for the successful defense to IV infections. Mice with a knockout in this gene are more susceptible to IV infections (Hatesuer et al. 2017). *IRF7* was significantly up-regulated in both human and mouse blood after infection (this study).

**Fig. 6:**
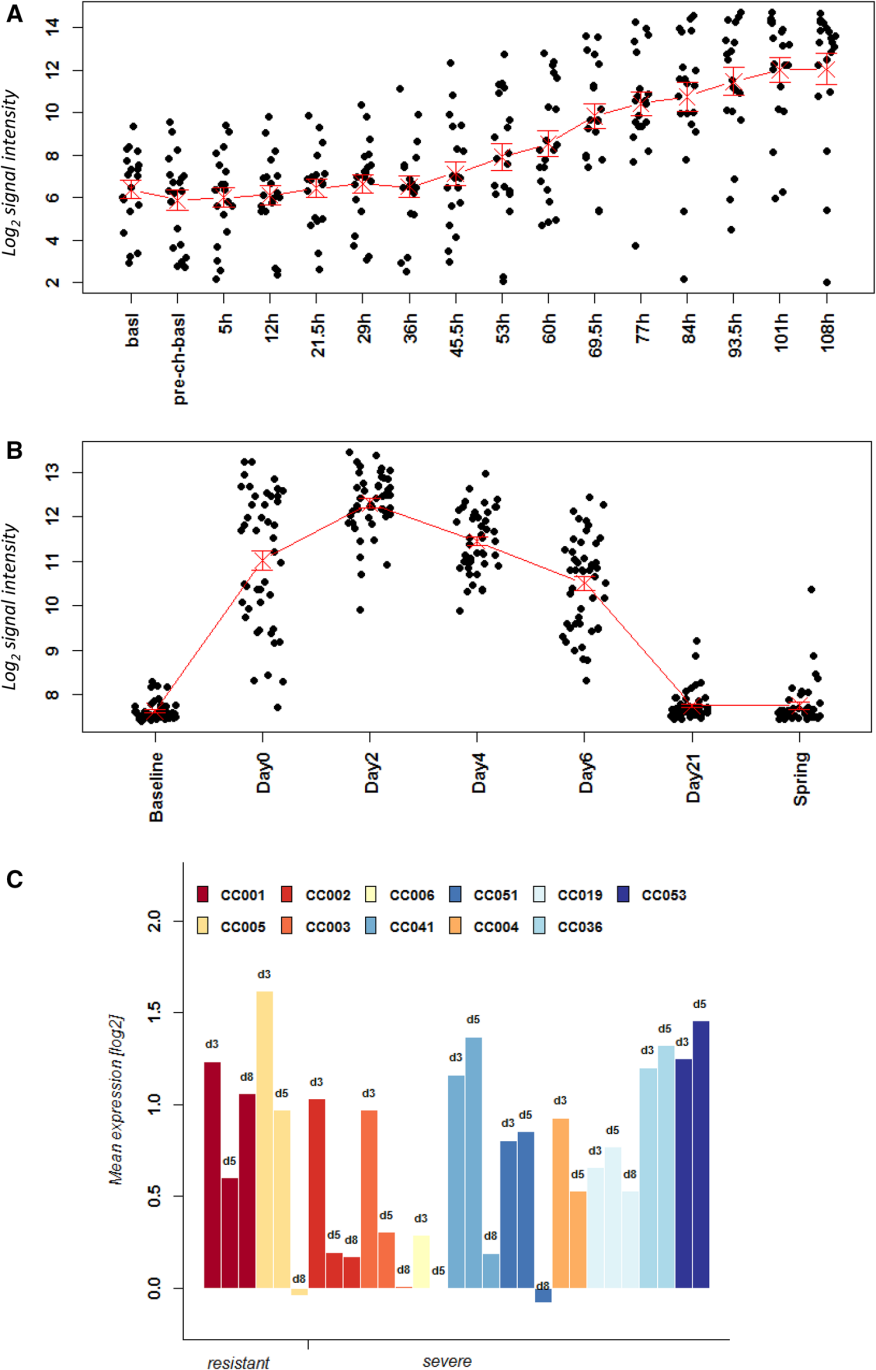
Time course of gene expression changes of *IFI27* in human and mouse data sets. Expression values of normalized log_2_-transformed signal intensities in the blood human and mice in infected individuals versus controls are shown at the indicated times pi. Stripcharts for individual expression values from **a** GSE52428 (Woods et al. 2013), **b** GSE68310 (Zhai et al. 2015). **c** Histogram representing the difference in the expression changes between infected mice at the indicated time pi minus mock control values for each CC mouse strain. Strains are sorted into resistant and severe groups

Our analyses showed that in humans, *IFI27* expression steadily increased from 53 h (day 6.6) pi until 108.5 h (day 13.6) pi in the Woods data set reaching the highest level at 108.5 h (Fig. 6a). In the Zhai data set, *IFI27* expression increased from day 0 to day 2 and then declined on day 4 and day 6 (Fig. 6b). Expression levels were back to baseline levels on day 21 and post-seasonal (Fig. 6b). The differences in the timings between the two datasets are due to different definitions of the time post infections. Woods counts the actual time after infection, whereas Zhai designates the day when patients come into the clinic with symptoms as day 0. In mice, individual CC strains were analyzed and for this comparison, we calculated the mean difference at each day with respect to mock controls. The expression kinetic in the CC mice strains was very similar to that in humans. Expression of *Ifi27* increased at day 3 pi and then declined on day 5 pi in most strains (Fig. 6c). However, individual differences for different strains could be observed. For example, CC001 still had high levels at day 8, whereas CC006 had lower levels already on day 3 and baseline levels on day 5. Also, other strains exhibited an increase in expression from day 3 to day 5. Visualization of individual expression values demonstrated that gene expression levels were highly reproducible within a given mouse strain and that the variation is not due to experimental variation (Fig. S3a). Of note, the baseline levels (mock-treated) were already different between CC mouse strains and profiles were different between strains although the general trends were identical (Fig. S3a). The results in the mouse model demonstrate again that genetic background is a major factor causing individual differences.

**Fig. 7:**
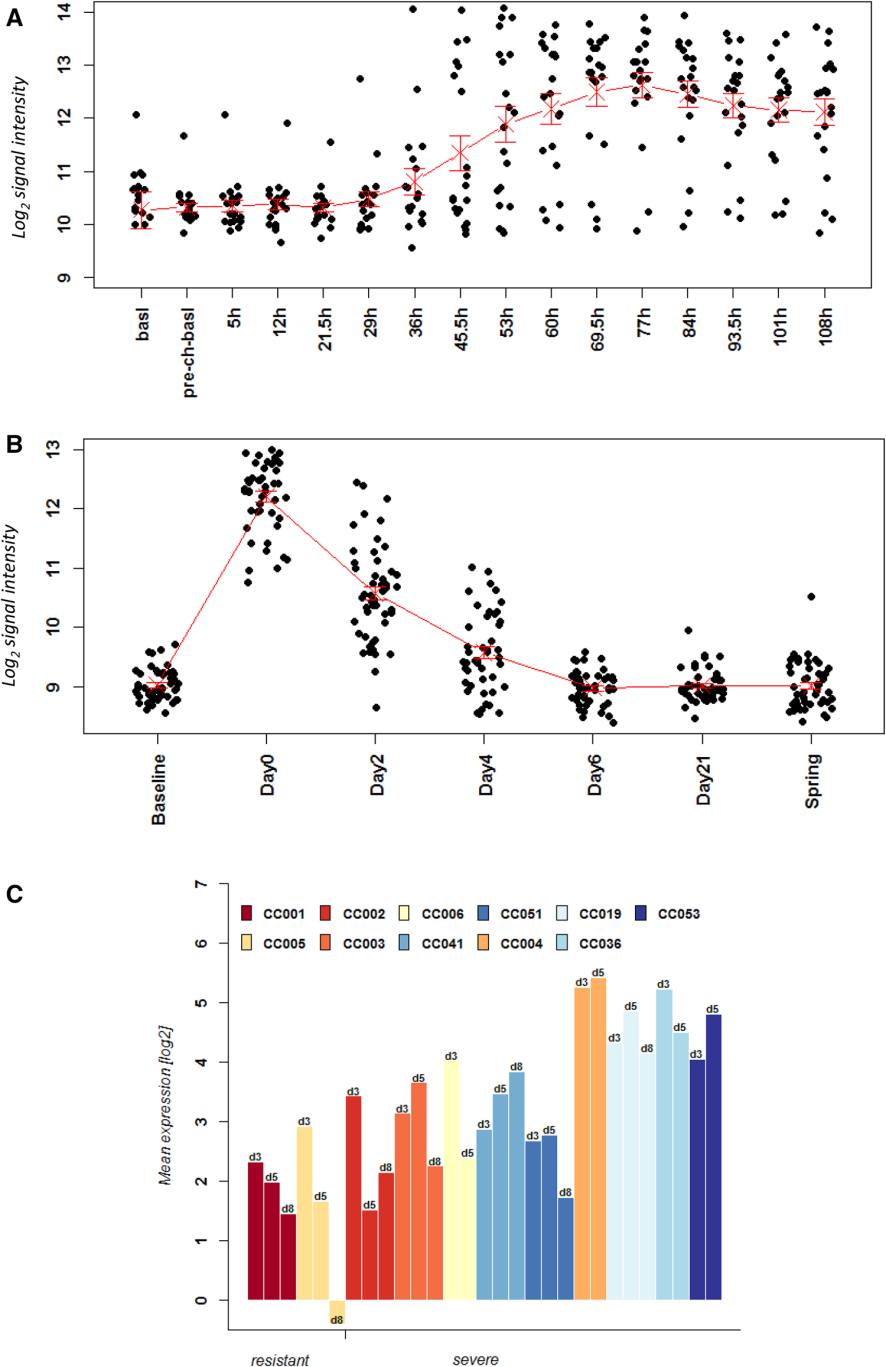
Time course of gene expression changes of *IRF7* in human and mouse data sets. Expression values of normalized log_2_-transformed signal intensities in the blood human and mice in infected individuals versus controls are shown at the indicated times pi. Stripcharts for individual expression values from **a** GSE52428 (Woods et al. 2013), **b** GSE68310 (Zhai et al. 2015). **c** Histogram representing the difference in the expression changes between infected mice at the indicated time pi minus mock control values for each CC mouse strain. Strains are sorted into resistant and severe groups

Expression analyses of *IRF7* showed similar results (Fig. 7c). It was up-regulated early in infection, increased steadily, and then declined at a later time point when the immune response in mice switches from innate to adaptive as previously described in mice (Pommerenke et al. 2012). Here again, the mouse model reproduced very well the general kinetics of gene expression that was observed in humans—with differences between individual strains (Fig. 7c, Fig. S3b). It is worth noting that in the Woods data set, expression of *IFI27* and *IRF7* separated into two different groups. One group showed an up-regulation of expression, and the other did not exhibit an up-regulation although volunteers were infected under very controlled conditions. The reason for this separation is unclear.

## Materials and methods

### Ethics statement

All experiments in mice were approved by an external committee according to the national guidelines of the animal welfare law in Germany (BGBl. I S. 1206, 1313 and BGBl. I S. 1934). The protocol used in these experiments has been reviewed by an ethics committee and approved by the relevant authority, the ‘Niedersächsisches Landesamt für Verbraucherschutz und Lebensmittelsicherheit, Oldenburg, Germany’ (Permit Numbers: 33.9.42502-04-051/09 and 3392 42502-04-13/1234).

### Viruses

A/Hong Kong/01/68 (H3N2) was originally obtained from Otto Haller, University of Freiburg (Haller et al. 1979). All viruses were propagated in the chorio-allantoic cavity of 10-day-old SPF (specific pathogen-free) embryonated chicken eggs (Charles River Laboratories, Germany) for 48 h at 37 °C, as described previously (Wilk and Schughart 2012), aliquoted, and stored at − 80 °C. The titer of the stock viruses was determined by focus forming unit assay (FFU/ml) in Madin-Darby Canine Kidney II (MDCK) cells (ATCC) as described previously (Wilk and Schughart 2012). Viral RNA was extracted using the QIAamp Viral RNA Extraction Kit (Qiagen) according to the manufacturer’s instructions and submitted for sequencing by Next-Generation Sequencing (Illumina) to validate identity of virus stocks.

### Infection of mice

The CC strains (CC001, CC002, CC003, CC004, CC005, CC006, CC019, CC036, CC041, CC051, and CC053) were obtained from the Systems Genetics Core Facility at the University of North Carolina (Welsh et al. 2012). Previous to their relocation to UNC, CC lines were generated and bred at Tel Aviv University in Israel (Iraqi et al. 2008), Geniad in Australia (Morahan et al. 2008), and Oak Ridge National Laboratory in the US (Chesler et al. 2008). Mice were bred in our animal facility at the HZI in Braunschweig by brother–sister mating for one to eight generations depending on the respective strain and time point of experiments. For experimental mice, also cousin mating was performed. Since only a few generations of breeding were done, the inbreeding status is expected to be similar to the mice that were received from UNC (http://csbio.unc.edu/CCstatus/CCGenomes/#genotypes). All mice were maintained under specific pathogen-free conditions and according to the German animal welfare law. Female, 8- to 12-week-old mice were anesthetized by intra-peritoneal injection with Ketamine/Xylazine (100 mg/ml Ketamine and 20 mg/ml Xylazine) in sterile sodium chloride solution. The doses were adjusted to the individual body weight using 200 µl/20 g body weight. Mice were then intra-nasally infected with 20 µl virus solution (10 FFU). Body weight was monitored daily as percentage of initial weight at the day of the infection. Mice that lost more than 30% of their body weight were euthanized for ethical reasons and were scored as dead.

### RNA isolation for arrays

Blood was taken for RNA isolation at different time points after virus infection (days 3, 5, and 8) and mock infection (day 3 post sterile phosphate-buffered saline infection). Body weight was measured until the day of sample collection. For every treatment and day post infection (pi), 3–5 mice per CC strain were analyzed. Blood was collected from anesthetized mice by retro-orbital bleeding into RNA protect animal blood tubes (Qiagen), kept at room temperature for at least 2 h and stored at -20 °C for long-term storage. Blood RNA was isolated with the RNeasy protect animal blood kit (Qiagen). RNA concentration was measured with the NanoDrop (Thermo Scientific).

### Mouse gene expression analysis

For gene expression profiling, the Mouse Gene expression kit from Agilent was used. 200 ng of total RNA was transcribed into cDNA, amplified using T7 RNA polymerase while incorporating cyanine 3-labeled CTP and then hybridized according to the manufacturer’s protocol (Quick Amp, Agilent) to the Agilent 4 × 44 k Mouse V2 Design ID: 026655. Signal intensities were extracted from scan images using Feature Extraction Software v10.7.3.1. Array data from mouse blood samples were analyzed using the R software (RCoreTeam 2013). Pre-processing steps included background correction, quantile normalization, and annotation using the MmAgilentDesign026655.db (Carlson 2014), limma (Smyth 2004), and Agi4 × 44PreProcess (Gentleman et al. 2004) packages. Identification of differentially expressed probe sets (DEPS) were performed with the LIMMA package (Smyth 2004) using BH correction for multiple testing (Benjamini and Hochberg 1995). Expression data have been deposited in the GEO expression database (https://www.ncbi.nlm.nih.gov/geo/info/linking.html) under the accession number GSE110384.

### Human data sets

The following human data were downloaded from the GEO expression data base (http://www.ncbi.nlm.nih.gov/geo/): GSE82050 (Tang et al. 2017), GSE52428 (Woods et al. 2013), GSE68310 (Zhai et al. 2015). GSE52428 expression data matrix was log_2_-transformed and quantile normalized and only data from volunteers with symptoms after the infection were used (Woods et al. 2013). This data set contained expression values from the blood of 20 volunteers for 18 time points including baseline and pre-challenge data. The 141.5 and 165.5 h time points pi contained only data from 1 volunteer and were omitted. GSE68310 expression data matrix, containing log_2_-transformed values, was downloaded from GEO and quantile normalized. Only data from influenza-infected patients were used (Zhai et al. 2015). This data set contained expression values from the blood of 49 patients for 7 time points including baseline and pre-challenge data. GSE82050 expression data were log_2_-transformed and quantile normalized as described (Tang et al. 2017). This data set contained expression values from the blood of 39 patients for 1 time point pi. As described in the result section and Table 2, only subsets that were relevant for our study were used. These were renamed to Woods_13, Zhai-15 and Tang_17, respectively, and are described in Table 2.

### Statistics

Data were analyzed using GraphPad Prism version 5.04 for Windows (GraphPad Software, San Diego, California). Mean and standard error of the mean (SEM) were calculated for all groups. Analysis of variance (ANOVA) was performed using the lm and aov functions of the R software package (RCoreTeam 2013). A repeated measure ANOVA was performed using the model weight loss ~ strain × day from day 0 to day 5 when all mice from all strains were still alive. Broad sense heritability was determined by calculating the interclass correlation (Rutledge et al. 2014): (MSB − MSW)/(MSB + (*n* − 1) × MSW) with MSB = mean square between groups, MSW = mean square within groups, n = mean number of mice per strain. Functional analysis of gene groups was performed with the R package clusterProfiler (Yu et al. 2012).

## Discussion/conclusions

Seasonal IV infections represent a significant public health burden, with an estimated 500 million cases of influenza infection occurring globally each year (Fauci 2006). This is further illustrated by that fact that in the United States there are approximately 200,000 IV-associated hospitalizations and 23,000 IV-associated deaths each year, with associated medical costs in excess of $10 billion dollars (Young-Xu et al. 2017). Therefore, given the large number of IV-associated hospitalizations and the significant health consequences of severe IV-associated disease, new methods are needed to rapidly assess an individual’s prognosis so that appropriate treatment regimens can be initiated in a timely manner to ensure better clinical outcomes. Transcriptional or proteomic analysis of readily available clinical samples, such as peripheral blood, represents a promising avenue for identifying prognostic biomarkers that can be used to predict patient outcome during IV infection (Herberg et al. 2013; Huang et al. 2011; Ioannidis et al. 2012; Marion et al. 2016; Parnell et al. 2012; Ramilo et al. 2007; Suarez et al. 2015; Tang et al. 2017; Thach et al. 2005; Tsalik et al. 2016; Woods et al. 2013; Zaas et al. 2009; Zhai et al. 2015). Due to difficulties associated with performing follow-up studies in humans, the validation and mechanistic evaluation of these bio-signatures often relies on animal models of IV disease.

However, the value of experimental models, especially the mouse, has recently been questioned in the scientific literature and in public (Seok et al. 2013). These results have been challenged later (Takao and Miyakawa 2014). Here, we addressed two important aspects that are often ignored in cross-species mouse/human comparisons: the influence of genetic background and the choice of the correct tissues. Our results clearly demonstrate that by comparing the appropriate tissue types (peripheral blood), and accounting for the impact of genetic variation on IV-associated gene expression through the use of genetically complex mouse panels, the bio-signatures identified in the mouse show good concordance with human studies. Therefore, these experimental animal systems represent an important set of tools for the identification, mechanistic analysis, and/or validation of bio-signatures of IV-induced disease.

IV infection of inbred mouse strains represents one of the most commonly used systems for studying IV disease pathogenesis and identifying host factors associated with virus-induced disease outcome (Kollmus et al. 2014). This is due to a wide range of factors, including the reproducibility of standard inbred strains, such as C57BL/6 mice, the ability to study the role of specific genes in IV disease pathogenesis through gene knockout animals, and the availability of an extensive toolkit of reagents for quantifying immune and other host responses in the mouse. However, as noted above, mouse models have recently come under attack due to questions about whether the mouse can reproduce disease states and gene expression profiles observed in humans (Seok et al. 2013). While the studies of Seok et al. raise concerns about mouse models, it is important to note that their study did not account for the impact of genetic diversity on differential findings in mice and humans. Therefore, our finding that studying a population of genetically diverse mouse strains, such as the CC, results in IV-induced transcriptional signatures that better reproduce human biomarkers has important implications not just for IV but also the study of other pathogens. Similar to the CC founders (Ferris et al. 2013; Leist et al. 2016), CC mice exhibit a broad range of phenotypes following infection with other pathogens (Gralinski et al. 2015; Rasmussen et al. 2014) which suggests that using the CC to identify bio-signatures for other pathogens may also provide greater concordance with human studies than is seen with existing mouse models.

Another important aspect of our studies is the direct comparison of transcriptional responses in the peripheral blood of mice and humans. Our analysis of IV-induced responses between lung and peripheral blood suggests that gene expression patterns and kinetics are different between these tissues which in addition to the effects of genetic diversity discussed above, likely contribute to the discordance between mouse and human gene expression studies. Therefore, it will be important to directly compare the same tissues in mice and humans when performing marker validation studies. Furthermore, our results illustrate the importance of performing carefully controlled gene expression studies in mice, since the ability to assess baseline gene expression in mock-infected animals provides an opportunity to specifically identify those genes which are differentially expressed due to IV infection.

Our studies address an important public health issue since there are no good biomarkers to distinguish bacterial from viral respiratory infections and for predicting severe course of influenza disease. Thus, transcripts that change in mice may represent potential biomarkers that need to be validated in humans. Therefore, the use of appropriately diverse mouse models, combined with assessment of gene expression differences in the appropriate tissue types (e.g., peripheral blood), provides an excellent system for identifying and confirming the most biologically relevant biomarkers for differentiating between IV versus other infections or predicting IV disease outcome.

Our analysis of mouse–human transcriptome changes in the peripheral blood demonstrated that the mouse represents a highly valuable model for validation and discovery of changes in single genes from human patients. At the level of single genes, the mouse model very well reproduces the responses observed in human cohorts, for differential expression between infected and non-infected groups and with respect to the time course of expression. Both *IFI27* and *IRF7* are up-regulated after IV infection in mice and humans, and these genes therefore represent good biomarker candidates for respiratory viral infections. Indeed, we have already followed up on this aspect and confirmed *IFI27* in an independent cohort in humans and shown that dendritic cells (DCs) activate expression of *IFI27* after exposure to IV.

In summary, gene expression changes at the single gene level demonstrate high reproducibility across species. Thus, results in humans can be validated in mice and vice versa. Therefore, well-designed cross-species studies are highly informative, in our opinion essential, for the development of better treatments and diagnosis in humans.

## Electronic supplementary material

Below is the link to the electronic supplementary material.

Supplementary material 1 (PDF 296 KB)

Supplementary material 2 (PDF 26 KB)

Supplementary material 3 (PDF 40 KB)

Supplementary material 4 (PDF 29 KB)

Supplementary material 5 (PDF 40 KB)

Supplementary material 6 (PDF 81 KB)

Supplementary material 7 (PDF 123 KB)

Supplementary material 8 (PDF 66 KB)

Supplementary material 9 (PDF 57 KB)

Supplementary material 10 (PDF 118 KB)

Supplementary material 11 (PDF 162 KB)

Supplementary material 12 (PDF 22 KB)

Supplementary material 13 (PDF 26 KB)
