# Supplementary material for: Of mice and men: the host response to influenza virus infection
Source: Mamm Genome. 2018 Jun 15;29(7):446–70. doi: 10.1007/s00335-018-9750-y (PMC6132725; doi:10.1007/s00335-018-9750-y)
Supplement: Supplementary file 1 — Supplementary material 1 (PDF 296 KB) [file 335_2018_9750_MOESM1_ESM.pdf]

Supplements

Of mice and men – the host response to influenza virus infection

Heike Kollmus[1], Carolin Pilzner[1], Sarah R. Leist[1,2], Mark Heise[3], Robert Geffers[3], Klaus Schughart[1,4,#]

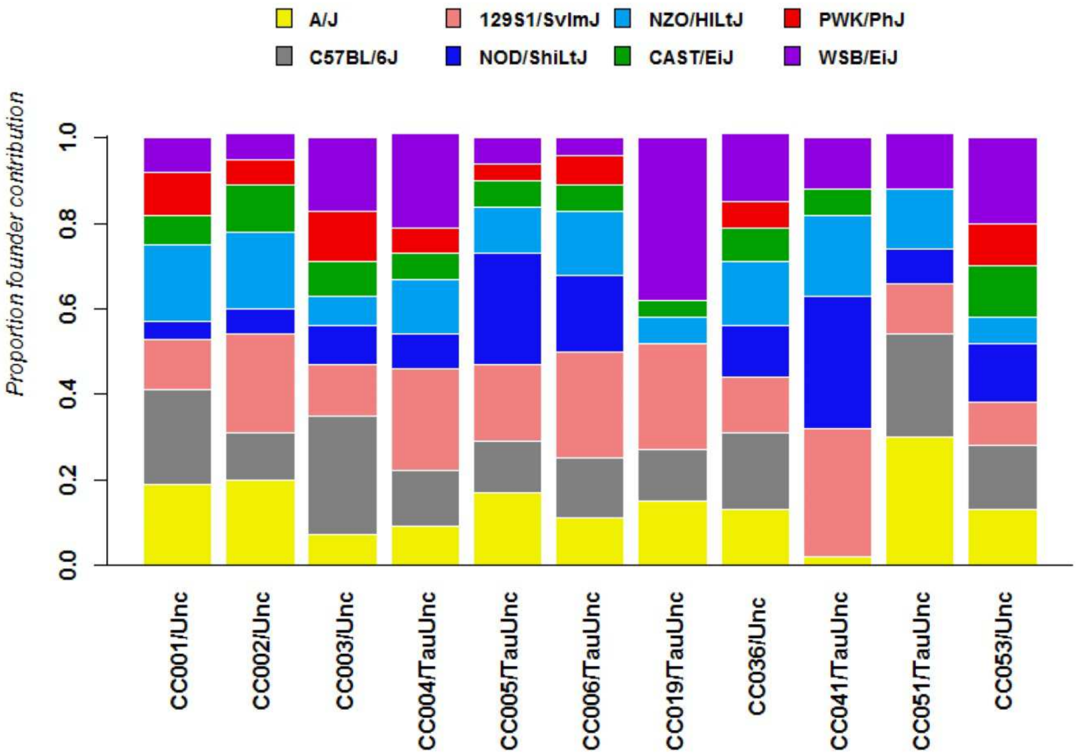

Fig. S1. Founder contributions in eleven CC strains from this study

Founder contributions for each CC strain as provided by the System Genetics Core facility at the University of North Carolina (http://csbio.unc.edu/CCstatus/index.py?run=availableLines); status January 2018.

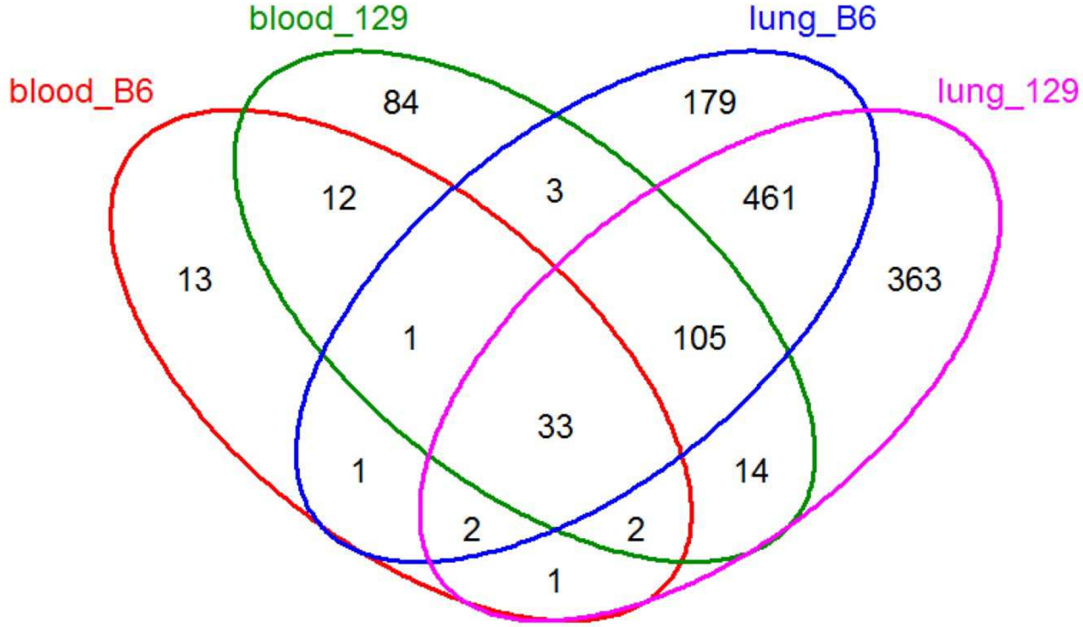

Fig. S2. VENN diagram comparing DEGs in mouse founder strains in blood and lungs

DEGs in lungs and blood were determined between infected and control mouse of C57BL/6J (B6) and 129S1/SvImJ (129) CC founder strains using the data set described by (Leist et al. 2016) (GSE74077). 26% of DEGs were shared in the lungs of B6 and 129 strains and 3.8 % in the blood between B6 and 129 strains. However, only 0.1 % were shared when blood of B6 was compared to lung of 129 and 0.3 % when lung of B6 was compared to blood of 129. Thus, lung and blood DEG do not show a strong overlap and, therefore, lung DEGs of mice should not be compared with blood DEGs in humans to evaluate validity of the mouse model.

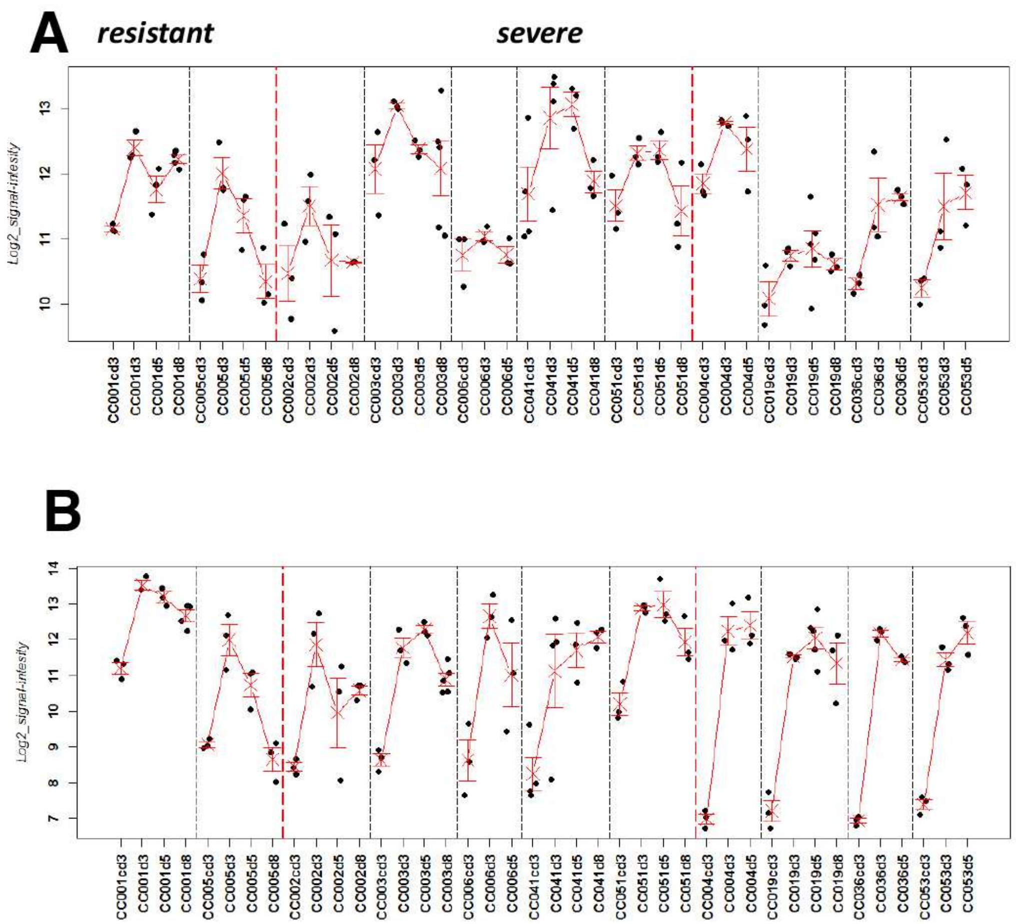

Fig. S3. Expression levels of genes in CC strains over time

Normalized log_2–transformed relative gene expression values of (A) *Ifi27* and (B) *Irf7* in CC mice. Dots represent values from individual mice. Crosses represent mean expression values per group, bars represent +/- 1 SEM (standard error of mean). Mock-infected control mice are designated ‘cd3’, infected mouse groups are labelled ‘d3’, ‘d5’, and ‘d8’ representing the time points p.i.

Supplementary Tables

Table S1: Sample list with annotations for CC strains

Table S2: Sample list with annotations for Woods_13

Table S3: Sample list with annotations for Zhai_15

Table S4: Sample list with annotations for Tang_17

Table S5: Differentially expressed probesets for comparison of CC infected resistant strains to mock controls

Table S6: Differentially expressed probesets for comparison of CC infected severe strains to mock controls

Table S7: Differentially expressed probesets for comparison of Woods_13 infected versus controls

Table S8: Differentially expressed probesets for comparison of Zhai_15 infected versus controls

Table S9: Differentially expressed probesets for comparison of Tang_17 infected versus controls

Table S10: Differentially expressed probesets for comparison of CC infected severe strains to resistant strains

Table S11: Genes in different groups from VENN diagram in Figure 4A.

Table S12: Genes in different groups from VENN diagram in Figure 4B.

References

Leist SR, Pilzner C, van den Brand JM, Dengler L, Geffers R, Kuiken T, Balling R, Kollmus H, Schughart K (2016) Influenza H3N2 infection of the collaborative cross founder strains reveals highly divergent host responses and identifies a unique phenotype in CAST/EiJ mice. BMC Genomics 17, 143
