# Supplementary material for: Of mice and men: the host response to influenza virus infection
Source: Mamm Genome. 2018 Jun 15;29(7):446–70. doi: 10.1007/s00335-018-9750-y (PMC6132725; doi:10.1007/s00335-018-9750-y)
Supplement: Supplementary file 2 — Supplementary material 2 (PDF 26 KB) [file 335_2018_9750_MOESM2_ESM.pdf]

des	strain	day	sev_bi
CC001cd3_1	CC001	cd3	control
CC001cd3_2	CC001	cd3	control
CC001cd3_3	CC001	cd3	control
CC001d3_1	CC001	d3	resistant
CC001d3_2	CC001	d3	resistant
CC001d3_3	CC001	d3	resistant
CC001d5_1	CC001	d5	resistant
CC001d5_2	CC001	d5	resistant
CC001d5_3	CC001	d5	resistant
CC001d8_1	CC001	d8	resistant
CC001d8_2	CC001	d8	resistant
CC001d8_5	CC001	d8	resistant
CC001d8_6	CC001	d8	resistant
CC002cd3_3	CC002	cd3	control
CC002cd3_4	CC002	cd3	control
CC002cd3_5	CC002	cd3	control
CC002d3_2	CC002	d3	severe
CC002d3_3	CC002	d3	severe
CC002d3_4	CC002	d3	severe
CC002d5_2	CC002	d5	severe
CC002d5_6	CC002	d5	severe
CC002d5_7	CC002	d5	severe
CC002d8_3	CC002	d8	severe
CC002d8_4	CC002	d8	severe
CC002d8_5	CC002	d8	severe
CC003cd3_4	CC003	cd3	control
CC003cd3_5	CC003	cd3	control
CC003cd3_6	CC003	cd3	control
CC003d3_4	CC003	d3	severe
CC003d3_5	CC003	d3	severe
CC003d3_6	CC003	d3	severe
CC003d5_3	CC003	d5	severe
CC003d5_4	CC003	d5	severe
CC003d5_5	CC003	d5	severe
CC003d8_1	CC003	d8	severe
CC003d8_2	CC003	d8	severe
CC003d8_3	CC003	d8	severe
CC003d8_4	CC003	d8	severe
CC003d8_5	CC003	d8	severe
CC004cd3_2	CC004	cd3	control
CC004cd3_3	CC004	cd3	control
CC004cd3_4	CC004	cd3	control
CC004d3_1	CC004	d3	severe
CC004d3_2	CC004	d3	severe
CC004d3_3	CC004	d3	severe
CC004d5_4	CC004	d5	severe
CC004d5_5	CC004	d5	severe
CC004d5_6	CC004	d5	severe
CC005cd3_1	CC005	cd3	control

CC005cd3_2	CC005	cd3	control
CC005cd3_3	CC005	cd3	control
CC005d3_1	CC005	d3	resistant
CC005d3_2	CC005	d3	resistant
CC005d3_3	CC005	d3	resistant
CC005d5_1	CC005	d5	resistant
CC005d5_2	CC005	d5	resistant
CC005d5_3	CC005	d5	resistant
CC005d8_1	CC005	d8	resistant
CC005d8_2	CC005	d8	resistant
CC005d8_3	CC005	d8	resistant
CC006cd3_3	CC006	cd3	control
CC006cd3_5	CC006	cd3	control
CC006cd3_6	CC006	cd3	control
CC006d3_2	CC006	d3	severe
CC006d3_4	CC006	d3	severe
CC006d3_5	CC006	d3	severe
CC006d5_1	CC006	d5	severe
CC006d5_2	CC006	d5	severe
CC006d5_4	CC006	d5	severe
CC019cd3_1	CC019	cd3	control
CC019cd3_2	CC019	cd3	control
CC019cd3_3	CC019	cd3	control
CC019d3_1	CC019	d3	severe
CC019d3_2	CC019	d3	severe
CC019d3_3	CC019	d3	severe
CC019d5_2	CC019	d5	severe
CC019d5_3	CC019	d5	severe
CC019d5_4	CC019	d5	severe
CC019d5_5	CC019	d5	severe
CC019d5_6	CC019	d5	severe
CC019d8_1	CC019	d8	severe
CC019d8_3	CC019	d8	severe
CC019d8_4	CC019	d8	severe
CC036cd3_1	CC036	cd3	control
CC036cd3_2	CC036	cd3	control
CC036cd3_3	CC036	cd3	control
CC036d3_4	CC036	d3	severe
CC036d3_5	CC036	d3	severe
CC036d3_6	CC036	d3	severe
CC036d5_1	CC036	d5	severe
CC036d5_3	CC036	d5	severe
CC036d5_5	CC036	d5	severe
CC041cd3_1	CC041	cd3	control
CC041cd3_2	CC041	cd3	control
CC041cd3_3	CC041	cd3	control
CC041cd3_7	CC041	cd3	control
CC041d3_3	CC041	d3	severe
CC041d3_4	CC041	d3	severe
CC041d3_5	CC041	d3	severe

CC041d3_6	CC041	d3	severe
CC041d5_4	CC041	d5	severe
CC041d5_5	CC041	d5	severe
CC041d5_6	CC041	d5	severe
CC041d8_1	CC041	d8	severe
CC041d8_3	CC041	d8	severe
CC041d8_6	CC041	d8	severe
CC051cd3_2	CC051	cd3	control
CC051cd3_3	CC051	cd3	control
CC051cd3_5	CC051	cd3	control
CC051d3_1	CC051	d3	severe
CC051d3_2	CC051	d3	severe
CC051d3_3	CC051	d3	severe
CC051d5_1	CC051	d5	severe
CC051d5_4	CC051	d5	severe
CC051d5_5	CC051	d5	severe
CC051d8_2	CC051	d8	severe
CC051d8_4	CC051	d8	severe
CC051d8_5	CC051	d8	severe
CC053cd3_4	CC053	cd3	control
CC053cd3_5	CC053	cd3	control
CC053cd3_6	CC053	cd3	control
CC053d3_3	CC053	d3	severe
CC053d3_4	CC053	d3	severe
CC053d3_5	CC053	d3	severe
CC053d5_2	CC053	d5	severe
CC053d5_3	CC053	d5	severe
CC053d5_4	CC053	d5	severe
