# Supplementary material for: Of mice and men: the host response to influenza virus infection
Source: Mamm Genome. 2018 Jun 15;29(7):446–70. doi: 10.1007/s00335-018-9750-y (PMC6132725; doi:10.1007/s00335-018-9750-y)
Supplement: Supplementary file 3 — Supplementary material 3 (PDF 40 KB) [file 335_2018_9750_MOESM3_ESM.pdf]

des	subject	virus	time	symptom	inf_stat
T_93.5h_H3N2_015	H3N2_015	H3N2	93.5h	y	inf
T_93.5h_H3N2_013	H3N2_013	H3N2	93.5h	y	inf
T_93.5h_H3N2_012	H3N2_012	H3N2	93.5h	y	inf
T_93.5h_H3N2_010	H3N2_010	H3N2	93.5h	y	inf
T_93.5h_H3N2_008	H3N2_008	H3N2	93.5h	y	inf
T_93.5h_H3N2_007	H3N2_007	H3N2	93.5h	y	inf
T_93.5h_H3N2_006	H3N2_006	H3N2	93.5h	y	inf
T_93.5h_H3N2_005	H3N2_005	H3N2	93.5h	y	inf
T_93.5h_H3N2_001	H3N2_001	H3N2	93.5h	y	inf
T_93.5h_H1N1_021	H1N1_021	H1N1	93.5h	y	inf
T_93.5h_H1N1_020	H1N1_020	H1N1	93.5h	y	inf
T_93.5h_H1N1_015	H1N1_015	H1N1	93.5h	y	inf
T_93.5h_H1N1_013	H1N1_013	H1N1	93.5h	y	inf
T_93.5h_H1N1_012	H1N1_012	H1N1	93.5h	y	inf
T_93.5h_H1N1_010	H1N1_010	H1N1	93.5h	y	inf
T_93.5h_H1N1_008	H1N1_008	H1N1	93.5h	y	inf
T_93.5h_H1N1_007	H1N1_007	H1N1	93.5h	y	inf
T_93.5h_H1N1_006	H1N1_006	H1N1	93.5h	y	inf
T_93.5h_H1N1_005	H1N1_005	H1N1	93.5h	y	inf
T_93.5h_H1N1_001	H1N1_001	H1N1	93.5h	y	inf
T_45.5h_H3N2_015	H3N2_015	H3N2	45.5h	y	inf
T_45.5h_H3N2_013	H3N2_013	H3N2	45.5h	y	inf
T_45.5h_H3N2_012	H3N2_012	H3N2	45.5h	y	inf
T_45.5h_H3N2_010	H3N2_010	H3N2	45.5h	y	inf
T_45.5h_H3N2_008	H3N2_008	H3N2	45.5h	y	inf
T_45.5h_H3N2_007	H3N2_007	H3N2	45.5h	y	inf
T_45.5h_H3N2_006	H3N2_006	H3N2	45.5h	y	inf
T_45.5h_H3N2_005	H3N2_005	H3N2	45.5h	y	inf
T_45.5h_H3N2_001	H3N2_001	H3N2	45.5h	y	inf
T_45.5h_H1N1_021	H1N1_021	H1N1	45.5h	y	inf
T_45.5h_H1N1_020	H1N1_020	H1N1	45.5h	y	inf
T_45.5h_H1N1_015	H1N1_015	H1N1	45.5h	y	inf
T_45.5h_H1N1_013	H1N1_013	H1N1	45.5h	y	inf
T_45.5h_H1N1_012	H1N1_012	H1N1	45.5h	y	inf
T_45.5h_H1N1_010	H1N1_010	H1N1	45.5h	y	inf
T_45.5h_H1N1_008	H1N1_008	H1N1	45.5h	y	inf
T_45.5h_H1N1_007	H1N1_007	H1N1	45.5h	y	inf
T_45.5h_H1N1_006	H1N1_006	H1N1	45.5h	y	inf
T_45.5h_H1N1_005	H1N1_005	H1N1	45.5h	y	inf
T_45.5h_H1N1_001	H1N1_001	H1N1	45.5h	y	inf
pre.ch.basl_H3N2_015	H3N2_015	H3N2	pre-ch-basl	y	cntrl
pre.ch.basl_H3N2_013	H3N2_013	H3N2	pre-ch-basl	y	cntrl
pre.ch.basl_H3N2_012	H3N2_012	H3N2	pre-ch-basl	y	cntrl
pre.ch.basl_H3N2_010	H3N2_010	H3N2	pre-ch-basl	y	cntrl
pre.ch.basl_H3N2_008	H3N2_008	H3N2	pre-ch-basl	y	cntrl
pre.ch.basl_H3N2_007	H3N2_007	H3N2	pre-ch-basl	y	cntrl
pre.ch.basl_H3N2_006	H3N2_006	H3N2	pre-ch-basl	y	cntrl
pre.ch.basl_H3N2_005	H3N2_005	H3N2	pre-ch-basl	y	cntrl
pre.ch.basl_H3N2_001	H3N2_001	H3N2	pre-ch-basl	y	cntrl

pre.ch.basl_H1N1_021	H1N1_021	H1N1	pre-ch-basl	y	cntrl
pre.ch.basl_H1N1_020	H1N1_020	H1N1	pre-ch-basl	y	cntrl
pre.ch.basl_H1N1_015	H1N1_015	H1N1	pre-ch-basl	y	cntrl
pre.ch.basl_H1N1_013	H1N1_013	H1N1	pre-ch-basl	y	cntrl
pre.ch.basl_H1N1_012	H1N1_012	H1N1	pre-ch-basl	y	cntrl
pre.ch.basl_H1N1_010	H1N1_010	H1N1	pre-ch-basl	y	cntrl
pre.ch.basl_H1N1_008	H1N1_008	H1N1	pre-ch-basl	y	cntrl
pre.ch.basl_H1N1_007	H1N1_007	H1N1	pre-ch-basl	y	cntrl
pre.ch.basl_H1N1_006	H1N1_006	H1N1	pre-ch-basl	y	cntrl
pre.ch.basl_H1N1_005	H1N1_005	H1N1	pre-ch-basl	y	cntrl
pre.ch.basl_H1N1_001	H1N1_001	H1N1	pre-ch-basl	y	cntrl
