# Supplementary material for: Of mice and men: the host response to influenza virus infection
Source: Mamm Genome. 2018 Jun 15;29(7):446–70. doi: 10.1007/s00335-018-9750-y (PMC6132725; doi:10.1007/s00335-018-9750-y)
Supplement: Supplementary file 4 — Supplementary material 4 (PDF 29 KB) [file 335_2018_9750_MOESM4_ESM.pdf]

sample_ID	subject.ID	time	sex	pthgn	inf_stat
JR0021_bsl	JR0021	Baseline	female	Flu	cntrl
JR0021_d2	JR0021	Day2	female	Flu	inf
JR0021_d4	JR0021	Day4	female	Flu	inf
JR0021_d6	JR0021	Day6	female	Flu	inf
JR0023_bsl	JR0023	Baseline	female	Flu	cntrl
JR0023_d2	JR0023	Day2	female	Flu	inf
JR0023_d4	JR0023	Day4	female	Flu	inf
JR0023_d6	JR0023	Day6	female	Flu	inf
JR0035_bsl	JR0035	Baseline	female	Flu	cntrl
JR0035_d2	JR0035	Day2	female	Flu	inf
JR0035_d4	JR0035	Day4	female	Flu	inf
JR0035_d6	JR0035	Day6	female	Flu	inf
JR0053_bsl	JR0053	Baseline	male	Flu	cntrl
JR0053_d2	JR0053	Day2	male	Flu	inf
JR0053_d4	JR0053	Day4	male	Flu	inf
JR0053_d6	JR0053	Day6	male	Flu	inf
JR0080_bsl	JR0080	Baseline	female	Flu	cntrl
JR0080_d2	JR0080	Day2	female	Flu	inf
JR0080_d4	JR0080	Day4	female	Flu	inf
JR0080_d6	JR0080	Day6	female	Flu	inf
JR0082_bsl	JR0082	Baseline	male	Flu	cntrl
JR0082_d2	JR0082	Day2	male	Flu	inf
JR0082_d4	JR0082	Day4	male	Flu	inf
JR0082_d6	JR0082	Day6	male	Flu	inf
JR0158_bsl	JR0158	Baseline	male	Flu	cntrl
JR0158_d2	JR0158	Day2	male	Flu	inf
JR0158_d4	JR0158	Day4	male	Flu	inf
JR0158_d6	JR0158	Day6	male	Flu	inf
JR0167_bsl	JR0167	Baseline	male	Flu	cntrl
JR0167_d2	JR0167	Day2	male	Flu	inf
JR0167_d4	JR0167	Day4	male	Flu	inf
JR0167_d6	JR0167	Day6	male	Flu	inf
JR0198_bsl	JR0198	Baseline	male	Flu	cntrl
JR0198_d2	JR0198	Day2	male	Flu	inf
JR0198_d4	JR0198	Day4	male	Flu	inf
JR0198_d6	JR0198	Day6	male	Flu	inf
JR0215_bsl	JR0215	Baseline	male	Flu	cntrl
JR0215_d2	JR0215	Day2	male	Flu	inf
JR0215_d6	JR0215	Day6	male	Flu	inf
JR0225_bsl	JR0225	Baseline	female	Flu	cntrl
JR0225_d2	JR0225	Day2	female	Flu	inf
JR0225_d4	JR0225	Day4	female	Flu	inf
JR0225_d6	JR0225	Day6	female	Flu	inf
JR0251_bsl	JR0251	Baseline	female	Flu	cntrl
JR0251_d2	JR0251	Day2	female	Flu	inf
JR0251_d4	JR0251	Day4	female	Flu	inf
JR0251_d6	JR0251	Day6	female	Flu	inf
JR0271_bsl	JR0271	Baseline	female	Flu	cntrl
JR0271_d2	JR0271	Day2	female	Flu	inf

JR0271_d4	JR0271	Day4	female	Flu	inf
JR0271_d6	JR0271	Day6	female	Flu	inf
JR0302_bsl	JR0302	Baseline	female	Flu	cntrl
JR0302_d2	JR0302	Day2	female	Flu	inf
JR0302_d4	JR0302	Day4	female	Flu	inf
JR0302_d6	JR0302	Day6	female	Flu	inf
JR0308_bsl	JR0308	Baseline	female	Flu	cntrl
JR0308_d2	JR0308	Day2	female	Flu	inf
JR0308_d4	JR0308	Day4	female	Flu	inf
JR0308_d6	JR0308	Day6	female	Flu	inf
JR0315_bsl	JR0315	Baseline	female	Flu	cntrl
JR0315_d2	JR0315	Day2	female	Flu	inf
JR0315_d4	JR0315	Day4	female	Flu	inf
JR0315_d6	JR0315	Day6	female	Flu	inf
JR0357_bsl	JR0357	Baseline	female	Flu	cntrl
JR0357_d2	JR0357	Day2	female	Flu	inf
JR0357_d4	JR0357	Day4	female	Flu	inf
JR0357_d6	JR0357	Day6	female	Flu	inf
JR0373_bsl	JR0373	Baseline	female	Flu	cntrl
JR0373_d2	JR0373	Day2	female	Flu	inf
JR0373_d4	JR0373	Day4	female	Flu	inf
JR0393_bsl	JR0393	Baseline	male	Flu	cntrl
JR0393_d2	JR0393	Day2	male	Flu	inf
JR0393_d4	JR0393	Day4	male	Flu	inf
JR0393_d6	JR0393	Day6	male	Flu	inf
JR0487_bsl	JR0487	Baseline	female	Flu	cntrl
JR0487_d2	JR0487	Day2	female	Flu	inf
JR0487_d4	JR0487	Day4	female	Flu	inf
JR0487_d6	JR0487	Day6	female	Flu	inf
JR0488_bsl	JR0488	Baseline	female	Flu	cntrl
JR0488_d2	JR0488	Day2	female	Flu	inf
JR0488_d4	JR0488	Day4	female	Flu	inf
JR0488_d6	JR0488	Day6	female	Flu	inf
JR0516_bsl	JR0516	Baseline	female	Flu	cntrl
JR0516_d2	JR0516	Day2	female	Flu	inf
JR0516_d4	JR0516	Day4	female	Flu	inf
JR0516_d6	JR0516	Day6	female	Flu	inf
JR0538_bsl	JR0538	Baseline	male	Flu	cntrl
JR0538_d2	JR0538	Day2	male	Flu	inf
JR0538_d4	JR0538	Day4	male	Flu	inf
JR0538_d6	JR0538	Day6	male	Flu	inf
JR0608_d2	JR0608	Day2	male	Flu	inf
JR0608_d4	JR0608	Day4	male	Flu	inf
JR0608_d6	JR0608	Day6	male	Flu	inf
JR1089_bsl	JR1089	Baseline	male	Flu	cntrl
JR1089_d2	JR1089	Day2	male	Flu	inf
JR1089_d4	JR1089	Day4	male	Flu	inf
JR1089_d6	JR1089	Day6	male	Flu	inf
JR1132_bsl	JR1132	Baseline	female	Flu	cntrl
JR1132_d2	JR1132	Day2	female	Flu	inf

JR1132_d4	JR1132	Day4	female	Flu	inf
JR1132_d6	JR1132	Day6	female	Flu	inf
JR1182_bsl	JR1182	Baseline	male	Flu	cntrl
JR1182_d2	JR1182	Day2	male	Flu	inf
JR1182_d4	JR1182	Day4	male	Flu	inf
JR1182_d6	JR1182	Day6	male	Flu	inf
JR1189_bsl	JR1189	Baseline	female	Flu	cntrl
JR1189_d2	JR1189	Day2	female	Flu	inf
JR1189_d4	JR1189	Day4	female	Flu	inf
JR1189_d6	JR1189	Day6	female	Flu	inf
JR1200_bsl	JR1200	Baseline	female	Flu	cntrl
JR1200_d2	JR1200	Day2	female	Flu	inf
JR1200_d4	JR1200	Day4	female	Flu	inf
JR1200_d6	JR1200	Day6	female	Flu	inf
JR1273_bsl	JR1273	Baseline	female	Flu	cntrl
JR1273_d2	JR1273	Day2	female	Flu	inf
JR1273_d4	JR1273	Day4	female	Flu	inf
JR1273_d6	JR1273	Day6	female	Flu	inf
JR1407_bsl	JR1407	Baseline	male	Flu	cntrl
JR1407_d2	JR1407	Day2	male	Flu	inf
JR1407_d4	JR1407	Day4	male	Flu	inf
JR1407_d6	JR1407	Day6	male	Flu	inf
JR1412_bsl	JR1412	Baseline	male	Flu	cntrl
JR1412_d2	JR1412	Day2	male	Flu	inf
JR1412_d4	JR1412	Day4	male	Flu	inf
JR1412_d6	JR1412	Day6	male	Flu	inf
JR1463_bsl	JR1463	Baseline	female	Flu	cntrl
JR1463_d2	JR1463	Day2	female	Flu	inf
JR1463_d4	JR1463	Day4	female	Flu	inf
JR1463_d6	JR1463	Day6	female	Flu	inf
JR1484_bsl	JR1484	Baseline	male	Flu	cntrl
JR1484_d2	JR1484	Day2	male	Flu	inf
JR1484_d4	JR1484	Day4	male	Flu	inf
JR1484_d6	JR1484	Day6	male	Flu	inf
JR1491_bsl	JR1491	Baseline	male	Flu	cntrl
JR1491_d2	JR1491	Day2	male	Flu	inf
JR1491_d4	JR1491	Day4	male	Flu	inf
JR1491_d6	JR1491	Day6	male	Flu	inf
JR1527_bsl	JR1527	Baseline	male	Flu	cntrl
JR1527_d2	JR1527	Day2	male	Flu	inf
JR1527_d4	JR1527	Day4	male	Flu	inf
JR1527_d6	JR1527	Day6	male	Flu	inf
JR1539_bsl	JR1539	Baseline	female	Flu	cntrl
JR1539_d2	JR1539	Day2	female	Flu	inf
JR1727_bsl	JR1727	Baseline	female	Flu	cntrl
JR1727_d2	JR1727	Day2	female	Flu	inf
JR1727_d4	JR1727	Day4	female	Flu	inf
JR1727_d6	JR1727	Day6	female	Flu	inf
JR1759_bsl	JR1759	Baseline	male	Flu	cntrl
JR1759_d2	JR1759	Day2	male	Flu	inf

JR1759_d6	JR1759	Day6	male	Flu	inf
JR1760_bsl	JR1760	Baseline	male	Flu	cntrl
JR1760_d2	JR1760	Day2	male	Flu	inf
JR1760_d4	JR1760	Day4	male	Flu	inf
JR1760_d6	JR1760	Day6	male	Flu	inf
JR1784_bsl	JR1784	Baseline	female	Flu	cntrl
JR1784_d2	JR1784	Day2	female	Flu	inf
JR1784_d4	JR1784	Day4	female	Flu	inf
JR1784_d6	JR1784	Day6	female	Flu	inf
JR1818_bsl	JR1818	Baseline	male	Flu	cntrl
JR1818_d2	JR1818	Day2	male	Flu	inf
JR1825_bsl	JR1825	Baseline	female	Flu	cntrl
JR1825_d2	JR1825	Day2	female	Flu	inf
JR1825_d4	JR1825	Day4	female	Flu	inf
JR1825_d6	JR1825	Day6	female	Flu	inf
JR1866_bsl	JR1866	Baseline	female	Flu	cntrl
JR1866_d2	JR1866	Day2	female	Flu	inf
JR1866_d4	JR1866	Day4	female	Flu	inf
JR1866_d6	JR1866	Day6	female	Flu	inf
JR1905_bsl	JR1905	Baseline	female	Flu	cntrl
JR1905_d2	JR1905	Day2	female	Flu	inf
JR1905_d4	JR1905	Day4	female	Flu	inf
JR1905_d6	JR1905	Day6	female	Flu	inf
JR1939_bsl	JR1939	Baseline	male	Flu	cntrl
JR1939_d2	JR1939	Day2	male	Flu	inf
JR1939_d4	JR1939	Day4	male	Flu	inf
JR1939_d6	JR1939	Day6	male	Flu	inf
JR1978_bsl	JR1978	Baseline	male	Flu	cntrl
JR1978_d2	JR1978	Day2	male	Flu	inf
JR1978_d4	JR1978	Day4	male	Flu	inf
JR1978_d6	JR1978	Day6	male	Flu	inf
JR1981_bsl	JR1981	Baseline	male	Flu	cntrl
JR1981_d2	JR1981	Day2	male	Flu	inf
JR1981_d4	JR1981	Day4	male	Flu	inf
JR1981_d6	JR1981	Day6	male	Flu	inf
JR1995_bsl	JR1995	Baseline	female	Flu	cntrl
JR1995_d2	JR1995	Day2	female	Flu	inf
JR1995_d4	JR1995	Day4	female	Flu	inf
JR1995_d6	JR1995	Day6	female	Flu	inf
