# Supplementary material for: Of mice and men: the host response to influenza virus infection
Source: Mamm Genome. 2018 Jun 15;29(7):446–70. doi: 10.1007/s00335-018-9750-y (PMC6132725; doi:10.1007/s00335-018-9750-y)
Supplement: Supplementary file 5 — Supplementary material 5 (PDF 40 KB) [file 335_2018_9750_MOESM5_ESM.pdf]

des_ID	Flu_detection	age	sex
INFG_ID_51	healthy_controls	50	f
INFG_ID_52	healthy_controls	47	f
INFG_ID_53	healthy_controls	43	m
INFG_ID_54	healthy_controls	67	m
INFG_ID_55	healthy_controls	59	f
INFG_ID_56	healthy_controls	45	f
INFG_ID_57	healthy_controls	27	f
INFG_ID_58	healthy_controls	29	m
INFG_ID_59	healthy_controls	48	f
INFG_ID_60	healthy_controls	37	f
INFG_ID_62	healthy_controls	50	f
INFG_ID_63	healthy_controls	30	m
INFG_ID_64	healthy_controls	56	m
INFG_ID_65	healthy_controls	30	f
INFG_ID_66	healthy_controls	58	m
INFG_ID_2	Influenza B	92	m
INFG_ID_4	Influenza A	24	f
INFG_ID_5	Influenza A	69	m
INFG_ID_7	Influenza A	55	f
INFG_ID_8	Influenza A	60	f
INFG_ID_9	Influenza A	47	m
INFG_ID_11	Influenza A	51	f
INFG_ID_17	Influenza A	26	f
INFG_ID_20	Influenza A	49	m
INFG_ID_21	Influenza A	29	f
INFG_ID_22	Influenza B	75	f
INFG_ID_23	Influenza A	45	m
INFG_ID_28	Influenza B	62	m
INFG_ID_30	Influenza A	73	f
INFG_ID_31	Influenza A	74	f
INFG_ID_33	Influenza A	75	m
INFG_ID_34	Influenza A	67	m
INFG_ID_35	Influenza A	90	m
INFG_ID_36	Influenza A	72	f
INFG_ID_38	Influenza A	32	m
INFG_ID_39	Influenza A	58	m
INFG_ID_40	Influenza B	83	m
INFG_ID_41	Influenza A	70	m
INFG_ID_42	Influenza A	80	m
