# Supplementary material for: Of mice and men: the host response to influenza virus infection
Source: Mamm Genome. 2018 Jun 15;29(7):446–70. doi: 10.1007/s00335-018-9750-y (PMC6132725; doi:10.1007/s00335-018-9750-y)
Supplement: Supplementary file 6 — Supplementary material 6 (PDF 81 KB) [file 335_2018_9750_MOESM6_ESM.pdf]

PROBE	ACCNUM	SYMBOL	ENTREZID	logFC	AveExpr	adj.P.Val
A_51_P421876	NM_016850	Irf7	54123	3,398893189	9,663735849	2,29E-07
A_51_P387123	NM_011854	Oasl2	23962	3,264877709	8,735339623	1,45E-10
A_51_P327751	NM_008331	Ifit1	15957	2,513472136	8,159320755	9,22E-08
A_51_P304170	NM_023386	Rtp4	67775	2,251371517	8,989566038	6,40E-07
A_66_P101942	AK019325	Gm9706	677168	2,227735294	11,61088679	4,80E-05
A_55_P2114953	NM_011909	Usp18	24110	2,066921053	8,006471698	2,79E-06
A_66_P128537	NM_015783	Isg15	100038882	2,066574303	10,74364151	0,000327965
A_55_P2103698	NM_015783	Isg15	100038882	2,044094427	14,47764151	0,000120151
A_55_P2019719	NM_145227	Oas2	246728	1,971091331	7,814	1,18E-07
A_51_P359570	NM_010501	Ifit3	15959	1,908117647	7,743924528	0,000123272
A_52_P700056	NM_001081957	Wfdc17	100034251	1,90571517	8,129886792	1,23E-05
A_55_P2105858	NM_030693	Atf5	107503	1,890928793	10,7660566	0,000300721
A_55_P2118441	NM_010846	Mx1	17857	1,854667183	6,999792453	2,01E-06
A_55_P1998943	NM_145211	Oas1a	246730	1,763	8,815018868	0,000816634
A_51_P256827	NM_013650	S100a8	20201	1,738705882	11,62860377	0,002912931
A_55_P2410875	AK034163	C030037D09Rik	193280	1,735534056	9,888849057	0,007276211
A_55_P1972872	NM_001005858	I830012O16Rik	667370	1,723270898	6,88945283	4,84E-06
A_51_P514085	NM_013606	Mx2	17858	1,722486068	7,085377358	0,009938069
A_51_P333274	NM_013542	Gzmb	14939	1,71479257	7,597207547	1,18E-07
A_55_P2086433	NM_145209	Oasl1	231655	1,692513932	8,117867925	9,27E-06
A_55_P1958250	NM_030743	Rnf114	81018	1,673428793	12,95558491	1,45E-10
A_51_P472867	NM_145226	Oas3	246727	1,6715	7,618716981	2,01E-07
A_51_P154842	NM_145153	Oas1f	243262	1,670224458	8,186377358	7,40E-05
A_55_P1996973	NM_029000	Gvin1	74558	1,662902477	10,57781132	0,000120151
A_51_P517695	NM_008530	Ly6f	17071	1,580402477	7,755735849	3,18E-05
A_55_P1999213	NM_146142	Tdrd7	100121	1,532527864	8,623132075	7,44E-07
A_51_P183812	NM_011410	Slfn4	20558	1,51226935	6,974075472	9,27E-07
A_52_P90363	NM_029803	Ifi27l2a	76933	1,509125387	12,18956604	0,000160945
A_55_P1958255	NM_030743	Rnf114	81018	1,50398452	8,880811321	3,96E-08
A_55_P2037343	NM_010391	H2-Q10	15007	1,501520124	8,168339623	1,40E-05
A_55_P2087118	NM_020498	Ly6i	57248	1,49604644	7,692377358	0,000142508
A_55_P2169356	AK156879	Gm1966	434223	1,489422601	8,806679245	0,000157509
A_66_P106388	NM_029499	Ms4a4c	64380	1,483441176	7,389358491	0,000402809
A_55_P2091461	NM_007609	Casp4	12363	1,472643963	8,411811321	8,84E-05
A_55_P1988975	BC028433	Ms4a4b	60361	1,455357585	8,643584906	0,046104374
A_55_P1998471	NM_009114	S100a9	20202	1,452060372	13,85922642	0,000165647
A_51_P128876	NM_025378	Ifitm3	66141	1,443509288	10,64939623	6,37E-05
A_55_P2331804	AK139027	AU015791	104932	1,431750774	8,022886792	0,001389331
A_55_P1998942	NM_145211	Oas1a	246730	1,426823529	7,342679245	0,000116126
A_55_P1984168	NM_007609	Casp4	12363	1,417179567	8,009339623	3,84E-05
A_55_P2013038	NM_148942	Serpinb6c	97848	1,410637771	9,096641509	0,009704785
A_55_P2047768	NM_148942	Serpinb6c	97848	1,400100619	10,01071698	0,01393898
A_55_P1979147	NM_001159904	Klrb1c	17059	1,37555418	7,656358491	0,000120151
A_55_P2062246	NM_001145164	Tgtp2	100039796	1,373930341	10,80145283	2,32E-05
A_55_P2066727	NM_001164118	Serpinb6a	20719	1,35919195	10,53743396	0,01149013
A_51_P203955	NM_010260	Gbp2	14469	1,35129257	8,187396226	2,60E-05
A_55_P1966833	NM_001037713	Xaf1	327959	1,339354489	7,767528302	1,40E-05
A_55_P2136880	NM_008654	Ppp1r15a	17872	1,337434985	11,01607547	2,60E-05
A_51_P346938	NM_029796	Lrg1	76905	1,312219814	8,642830189	0,0018094

A_51_P163444	NM_025821	Carhsp1	52502	1,305902477	10,40783019	0,000128517
A_51_P112355	NM_018738	Igtp	16145	1,304325077	10,758	7,40E-07
A_55_P2019557	NM_153101	Mrgpra2b	235712	1,267348297	7,594773585	3,11E-06
A_55_P2172001	NM_001172588	Mrgpra2a	668727	1,264623839	7,526679245	4,37E-06
A_52_P638459	NM_013653	Ccl5	20304	1,262470588	11,81711321	0,000186871
A_55_P1960231	NM_019913	Txn2	56551	1,25329257	12,02526415	0,059124029
A_52_P518808	BC027020	Mmd	67468	1,236956656	8,448849057	1,22E-06
A_51_P151182	NM_011940	Ifi202b	26388	1,229241486	7,114377358	0,000440342
A_55_P2051455	NM_178882	D2hgdh	98314	1,227582043	10,62254717	0,018981481
A_51_P213691	NM_011324	Scnn1a	20276	1,227557276	7,622773585	1,97E-05
A_55_P1959748	NM_012055	Asns	27053	1,225659443	7,875622642	1,96E-07
A_55_P2026639	NM_001100614	Gm11564	670496	1,221702786	8,411792453	0,002899772
A_51_P501312	NM_025294	Natd1	24083	1,218628483	8,133396226	1,45E-10
A_51_P254656	NM_008230	Hdc	15186	1,216275542	8,59990566	4,82E-05
A_55_P2094060	NM_010370	Gzma	14938	1,213625387	11,75613208	4,89E-05
A_55_P2100088	NM_001013784	E130309D14Rik	432582	1,213544892	6,699867925	1,18E-07
A_55_P2088401	NM_010399	H2-T9	15051	1,204386997	7,446584906	1,04E-05
A_55_P2081164	NM_001169153	Cd300lf	246746	1,199204334	8,571962264	2,43E-06
A_51_P149714	NM_026835	Ms4a6d	68774	1,197866873	6,775188679	1,72E-05
A_55_P2032167	NM_008327	Ifi202b	26388	1,192859133	7,040716981	0,000294933
A_52_P422494	NM_145634	Cd300lf	246746	1,167109907	8,378603774	1,47E-06
A_51_P262171	NM_008326	Irgm1	15944	1,159239938	8,00654717	8,30E-06
A_55_P2148478	NM_029652	Klhl25	207952	1,147356037	8,826433962	0,000455424
A_51_P246653	NM_020008	Clec7a	56644	1,131208978	9,214792453	0,000560332
A_55_P2019601	NM_033616	Csprs	114564	1,11003096	7,867641509	8,88E-06
A_55_P2180096	NM_001178039	Gm10136	672214	1,109582043	13,18824528	3,27E-05
A_51_P296608	NM_007836	Gadd45a	13197	1,108134675	9,687226415	3,47E-05
A_51_P288138	NM_008039	Fpr2	14289	1,104408669	7,495037736	0,000105034
A_55_P2146254	NM_001112715	Ifitm1	68713	1,104320433	9,35209434	0,001173854
A_55_P1953169	NM_011315	Saa3	20210	1,09921517	6,464264151	0,064102639
A_52_P450934	NM_198414	Paqr9	75552	1,079479876	11,11792453	0,058592576
A_55_P2156288	XM_001479076	Gm3932	100042616	1,074475232	7,090924528	0,030086243
A_51_P325856	NM_026985	Mcemp1	69189	1,067676471	7,806075472	0,000283603
A_66_P139683	NM_021394	Zbp1	58203	1,06672291	7,042792453	3,29E-05
A_51_P126198	NM_010019	Dapk2	13143	1,06669969	8,645754717	0,000372126
A_55_P2066578	BC010546	Ifi204	15951	1,065859133	6,865188679	5,86E-05
A_55_P2065567	NM_026527	Chac2	68044	1,063289474	9,198679245	0,007703597
A_52_P375312	NM_001005421	Amica1	270152	1,062831269	9,233867925	0,000300721
A_51_P383032	NM_010819	Clec4d	17474	1,060335913	9,418679245	0,000282464
A_55_P1957459	NM_013532	Lilrb4	14728	1,051393189	8,797679245	0,000122325
A_52_P190405	NM_010509	Ifnar2	15976	1,050089783	8,96209434	1,11E-07
A_55_P1988844	NM_016970	Klrg1	50928	1,045434985	7,269396226	2,42E-06
A_51_P490023	NM_009450	Tubb2a	22151	1,040599071	11,47160377	0,005085532
A_52_P104824	NM_019670	Diap3	56419	1,036352941	11,36716981	0,081484999
A_52_P535484	NM_029000	Gvin1	74558	1,035865325	10,92937736	0,000122325
A_51_P129012	NM_009735	B2m	12010	1,034074303	13,509	0,00013013
A_55_P2170509	NM_026417	Yipf4	67864	1,024323529	10,86388679	0,069651923
A_51_P169693	NM_198095	Bst2	69550	1,022998452	7,803	0,000160945
A_55_P2172396	NM_178796	A530064D06Rik	328830	1,021400929	6,900603774	2,93E-06
A_52_P127362	NM_029478	Vmp1	75909	1,019236842	9,164886792	0,000142544

A_51_P463846	NM_145545	Gbp7	229900	1,01830031	7,359698113	7,40E-05
A_55_P2005783	NM_027835	Ifih1	71586	1,016690402	6,991415094	8,48E-06
A_51_P169516	NM_001085501	Ppp1r3d	228966	1,010328173	7,475075472	1,33E-05
A_55_P2334424	AK158188	D630024D03Rik	414116	1,005970588	7,639660377	0,000615321
A_55_P2011991	NM_022332	St7	64213	1,000890093	9,275603774	0,000723139
A_51_P272817	NM_008928	Map2k3	26397	0,999630031	9,595622642	2,60E-05
A_52_P476560	NM_145853	Tpcn1	252972	0,995996904	8,342584906	9,41E-06
A_51_P362066	NM_007695	Chil1	12654	0,995893189	7,186283019	0,000123736
A_51_P112627	NM_009180	St6galnac2	20446	0,993634675	7,340679245	0,012619511
A_51_P249118	NM_008830	Abcb4	18670	0,98378483	8,468471698	0,00047072
A_55_P2025248	NM_010751	Mxd1	17119	0,980371517	10,74892453	3,21E-06
A_55_P1962305	NM_139198	Plac8	231507	0,967394737	10,68530189	0,014082886
A_51_P487690	NM_133871	Ifi44	99899	0,960360681	6,398603774	0,00047247
A_55_P2070079	NM_010708	Lgals9	16859	0,960071207	7,78	0,001203076
A_55_P2197638	AK004186	1110046J04Rik	68808	0,951128483	7,172	0,003441842
A_51_P134030	NM_145210	Oas1e	231699	0,946839009	6,793962264	9,22E-08
A_55_P1994927	NM_144538	Rab3il1	74760	0,945956656	9,199528302	0,011842179
A_52_P559975	NM_009909	Cxcr2	12765	0,944020124	8,437981132	0,001740062
A_52_P332788	AK016231	C130026I21Rik	620078	0,942575851	7,435433962	0,000124861
A_51_P241769	NM_011270	Rhd	19746	0,938701238	9,261603774	0,060820888
A_55_P1979708	NM_023305	Ubap1	67123	0,935572755	9,04245283	0,001478037
A_51_P470079	NM_010555	Il1r2	16178	0,932335913	6,911792453	0,000734254
A_55_P2030568	NM_053217	2010002M12Rik	112419	0,924371517	7,111849057	4,53E-05
A_66_P103398	NM_001081079	Ogfrl1	70155	0,918354489	8,235603774	0,01579915
A_55_P2034864	NM_023716	Tubb2b	73710	0,914394737	7,714301887	0,003543777
A_52_P670026	NM_021384	Rsad2	58185	0,913894737	14,90862264	0,000473384
A_51_P155142	NM_026560	Cdca8	52276	0,910417957	6,99290566	3,87E-06
A_51_P275454	NM_009099	Trim30a	20128	0,905860681	7,624566038	4,06E-05
A_55_P2117656	NM_181545	Slfn8	276950	0,905162539	7,683698113	0,012749009
A_55_P2052181	NM_025534	Ccdc82	66396	0,901139319	9,251226415	9,19E-06
A_51_P221014	NM_010581	Cd47	16423	0,899843653	10,32732075	0,000529085
A_55_P1998811	XM_001476688	Gm3430	100041612	0,899470588	8,507981132	0,038485254
A_51_P262515	NM_172603	Phf11a	219131	0,899105263	7,633320755	0,000189056
A_51_P159503	AK173199	Rnf213	672511	0,898026316	7,823433962	8,33E-05
A_55_P2048607	NM_017370	Hp	15439	0,897743034	10,74677358	0,004621397
A_51_P413866	NM_008198	Cfb	14962	0,896710526	6,579962264	0,00288003
A_51_P464918	NM_019453	Mefv	54483	0,896157895	7,597264151	0,000318713
A_55_P2015687	NM_001164323	Phf11d	219132	0,89494582	7,75209434	0,000956406
A_55_P1994042	NM_001139519	Zbp1	58203	0,893498452	8,661075472	0,000198839
A_51_P444447	NM_007679	Cebpd	12609	0,889010836	9,316849057	0,004084006
A_55_P1997997	NM_024470	Klra23	79410	0,885741486	6,958735849	5,70E-06
A_52_P186937	NM_020557	Cmpk2	22169	0,883303406	6,755773585	2,63E-05
A_55_P2004016	NM_030209	Crispld2	78892	0,88325387	6,771226415	0,000335715
A_52_P431615	XM_001000891	Gm1966	434223	0,880859133	8,295867925	0,002153606
A_52_P467046	NM_026236	Wdr48	67561	0,880705882	7,166018868	0,000161581
A_51_P212782	NM_008361	Il1b	16176	0,874063467	8,685490566	0,005649284
A_55_P2052016	NM_030209	Crispld2	78892	0,873312693	6,611603774	0,000186871
A_55_P2016462	NM_021274	Cxcl10	15945	0,871801858	6,269415094	4,06E-05
A_55_P2170349	NM_053152	Klra22	93969	0,871208978	8,571584906	0,002940716
A_66_P112006	NM_010395	H2-T10	15024	0,866902477	7,75445283	0,000641121

A_55_P2059154	NM_001013371	Dtx3l	209200	0,866662539	8,460396226	2,63E-05
A_55_P2167451	NM_033569	Cnnm2	94219	0,863904025	8,289113208	0,004921733
A_51_P198675	NM_138951	Ttc36	192653	0,861255418	7,883075472	0,017900123
A_52_P1020860	NM_001177351	AW112010	107350	0,860995356	9,08445283	0,004910531
A_55_P2051596	XM_001479558	Gm4499	100043524	0,855565015	7,82709434	0,038694704
A_55_P2048279	NM_205820	Tlr13	279572	0,851077399	8,563867925	0,000392564
A_51_P373461	NM_145956	Brcc3	210766	0,848693498	8,124660377	1,96E-06
A_55_P1975560	NM_008329	Ifi204	15951	0,84619969	8,824207547	0,000403702
A_51_P299062	NM_032540	Kel	23925	0,845733746	9,867716981	0,009112054
A_52_P493620	NM_026218	Fgfr1op2	67529	0,844399381	9,728415094	0,009938069
A_55_P2129354	NM_153510	Pilra	231805	0,842006192	8,045792453	0,000122325
A_55_P2004527	NM_010650	Klra8	16639	0,841247678	7,339226415	0,000300721
A_55_P2019690	NM_001081349	Slc43a1	72401	0,83744582	7,909981132	0,005266879
A_52_P266132	NM_008013	Fgl2	14190	0,836863777	7,428301887	1,39E-05
A_51_P338878	NM_027571	P2ry12	70839	0,834710526	9,833735849	0,003554136
A_51_P120066	NR_033222	9330151L19Rik	414085	0,829987616	8,107660377	0,010302611
A_55_P2168185	XM_357571	Gm5299	384325	0,82953096	15,20958491	0,000561949
A_55_P2063376	NM_013458	Add2	11519	0,829396285	8,436566038	0,009211127
A_52_P484838	NM_011266	Rfxank	19727	0,829063467	8,956358491	0,000160945
A_52_P110052	NM_010045	Ackr1	13349	0,827541796	8,794018868	0,001395685
A_55_P2069842	NM_001081232	D5Ertd579e	320661	0,827450464	8,262603774	0,000241849
A_51_P108108	NM_153074	Lrrc25	211228	0,826473684	7,501283019	2,49E-05
A_55_P2320283	AK033703	Hmga2-ps1	15365	0,823472136	10,3194717	0,055942086
A_52_P199633	NM_199146	Trim30d	209387	0,822611455	6,957339623	8,30E-06
A_52_P511821	NM_027057	Wdfy1	69368	0,820235294	6,798811321	1,56E-05
A_51_P459070	NM_024251	Aplf	72103	0,812326625	7,005358491	6,27E-07
A_55_P2072115	NR_030671	AW011738	100382	0,809890093	6,900132075	0,003318544
A_55_P1985693	NM_001033301	Fhdc1	229474	0,808467492	7,145886792	0,000213769
A_55_P1998957	NM_033541	Oas1c	114643	0,806013932	7,204566038	4,34E-05
A_55_P2141699	NM_001033135	Rnf149	67702	0,80571517	7,98954717	1,37E-05
A_51_P258372	NM_030691	Igsf6	80719	0,803294118	8,613679245	0,000509104
A_51_P147274	NM_153197	Clec4a3	73149	0,798557276	7,048981132	0,000473384
A_51_P501364	NM_019507	Tbx21	57765	0,797226006	7,457150943	5,99E-05
A_55_P2040838	NM_001166672	Gm14548	100038909	0,797060372	8,208415094	0,000937343
A_55_P1978424	NM_007536	Bcl2a1d	12047	0,792606811	8,092377358	0,013472454
A_51_P499838	NM_009763	Bst1	12182	0,782294118	6,828150943	0,000575583
A_55_P2057936	NM_001135115	Gm12250	631323	0,77794582	7,549150943	0,001581545
A_51_P248666	NM_021893	Cd274	60533	0,774843653	7,271509434	1,52E-05
A_51_P359636	NM_011150	Lgals3bp	19039	0,773029412	9,45009434	0,037022708
A_55_P2042184	XM_001476516	Gm3181	100041175	0,772626935	6,973773585	0,04517934
A_52_P139650	NM_025581	Ska1	66468	0,771004644	6,483603774	0,000239357
A_52_P577384	NM_010531	Il18bp	16068	0,769543344	6,356962264	0,000111931
A_55_P2011084	AK172348	Trim30b	244183	0,766179567	6,950962264	0,00061332
A_55_P1991931	NM_025533	Nosip	66394	0,765778638	7,893377358	0,006330986
A_55_P1992487	XM_888318	Gm6453	623818	0,765306502	9,102943396	0,009112054
A_51_P438083	NM_144512	Slc6a13	14412	0,765089783	6,662924528	9,23E-10
A_51_P400543	NM_019467	Aif1	11629	0,765013932	6,839867925	0,000120151
A_51_P469568	NM_020504	Cldn13	57255	0,764229102	6,616792453	2,49E-05
A_52_P183524	NM_023440	Tmem86b	68255	0,763653251	6,614056604	0,002698558
A_66_P109986	NM_001111058	Cd33	12489	0,762817337	6,600698113	1,23E-05

A_55_P2131238	NM_153392	Ttc39a	230603	0,762390093	7,740603774	0,01733287
A_51_P281333	NM_018784	St3gal6	54613	0,761383901	7,423301887	9,27E-07
A_51_P370678	NM_008114	Gfi1b	14582	0,760495356	7,605924528	0,001235134
A_52_P223809	NM_030150	Dhx58	80861	0,757726006	6,586490566	2,87E-05
A_66_P119518	NM_017379	Tuba8	53857	0,756487616	7,707811321	0,003431106
A_52_P42245	NM_010737	Klrb1a	17057	0,753557276	6,962849057	0,005528871
A_55_P2137611	NM_019440	Irgm2	54396	0,751218266	7,628981132	0,000877034
A_66_P120567	NM_010730	Anxa1	16952	0,749843653	8,07054717	0,001786263
A_55_P2004541	NM_001110323	Klra7	16638	0,745088235	7,294018868	0,001471779
A_55_P2324976	NR_029464	5033406O09Rik	77675	0,744766254	6,721962264	0,010863676
A_66_P121787	NM_010156	Samd9l	209086	0,740919505	8,645377358	0,001316951
A_55_P2150717	NM_010136	Eomes	13813	0,73848452	8,740886792	0,011871383
A_55_P2013043	NM_011454	Serpinb6b	20708	0,738190402	7,108075472	1,22E-06
A_55_P2090184	NM_026218	Fgfr1op2	67529	0,737990712	10,72515094	0,010219772
A_55_P1962404	NM_201410	Ugt1a6b	394435	0,736490712	7,731113208	0,00135556
A_55_P2113498	NM_010654	Klrd1	16643	0,736210526	7,864924528	0,001207429
A_52_P615247	AK087744	A530040E14Rik	621875	0,733428793	7,504603774	0,000791945
A_52_P86693	NM_026790	Ifi27	52668	0,728758514	11,24154717	0,035533373
A_55_P2059606	NM_019963	Stat2	20847	0,726654799	7,548528302	0,000321788
A_55_P1972634	NM_010839	Mtcp1	17763	0,726419505	8,312679245	0,001573331
A_51_P225793	NM_175181	Prr5l	72446	0,726060372	8,611962264	0,002004919
A_55_P2076543	XM_001476757	Gm10144	100041121	0,725326625	11,15016981	0,025773647
A_55_P2144364	XM_001479624	Gm4320	100043258	0,72506192	7,002339623	0,036307298
A_55_P2151601	NM_010156	Samd9l	209086	0,723600619	9,210698113	0,00388012
A_55_P2149363	NM_172286	6430548M08Rik	234797	0,721970588	8,054849057	0,000915152
A_51_P465281	NM_008495	Lgals1	16852	0,721676471	10,19903774	0,009526163
A_55_P1971054	NM_198414	Paqr9	75552	0,718216718	7,573415094	0,035533373
A_51_P352968	NM_008538	Marcks	17118	0,718001548	8,519132075	0,003345639
A_55_P1978316	NM_001081127	Adamts14	237360	0,716543344	7,816962264	0,005525044
A_55_P2078494	NM_001080812	Cib3	234421	0,716134675	7,382698113	0,055137383
A_51_P490795	NM_010751	Mxd1	17119	0,715752322	9,006943396	7,96E-05
A_52_P313285	AF108397	Slc8a1	20541	0,713534056	7,591471698	0,037022479
A_55_P2094626	NM_175211	Ralgps1	241308	0,713049536	8,689150943	0,000239357
A_51_P181517	NM_144559	Fcgr4	246256	0,713034056	7,671792453	0,001642855
A_55_P1962304	NM_139198	Plac8	231507	0,710182663	8,091358491	0,000198839
A_55_P1959985	NM_020559	Alas1	11655	0,708851393	11,2245283	0,078791852
A_51_P425632	NM_025649	Mad2l1bp	66591	0,705834365	9,192358491	0,000290242
A_55_P2066017	XM_001478769	Gm4015	100042759	0,705630031	6,568226415	0,002410946
A_55_P1978681	NM_146010	Tspan8	216350	0,703662539	6,949962264	0,000724706
A_55_P2095754	NM_207534	Mrgpra5	404235	0,703325077	6,29154717	0,00110303
A_52_P482124	NM_026455	Fam32a	67922	0,701006192	9,395245283	0,000142544
A_51_P197378	NM_026217	Atg12	67526	0,700885449	11,32684906	0,003188176
A_55_P2017764	NM_028120	Cep89	72140	0,69894582	7,102830189	7,40E-05
A_51_P224983	NM_012000	Cln8	26889	0,698794118	7,448716981	0,04299498
A_55_P2094686	AK078025	2700089E24Rik	381820	0,698622291	8,123037736	0,025031068
A_55_P2076631	NM_001048207	Gypc	71683	0,698143963	9,307660377	0,000300721
A_55_P2077783	NR_003964	Tubb2a-ps2	627110	0,696506192	7,471132075	0,007150345
A_55_P2045886	NM_019635	Stk3	56274	0,695414861	7,898358491	0,058756707
A_51_P485594	NM_025729	Tab3	66724	0,69230031	8,705830189	0,013455178
A_55_P2092826	NM_010730	Anxa1	16952	0,692089783	9,717754717	0,012619511

A_51_P323180	NM_172777	Gbp9	236573	0,69177709	7,515113208	0,000417836
A_55_P1998578	NM_030694	Ifitm2	80876	0,691320433	8,581037736	0,004796554
A_55_P2133195	NM_001033767	Gm4951	240327	0,690608359	6,26554717	0,003068395
A_52_P541161	NM_022881	Rgs18	64214	0,690185759	11,35866038	0,086310014
A_66_P122086	NM_001039720	9030619P08Rik	105892	0,688749226	7,071792453	0,000403702
A_51_P488739	NM_030701	Hcar2	80885	0,687834365	6,52290566	7,44E-07
A_55_P2179413	NM_011150	Lgals3bp	19039	0,68549226	9,159566038	0,003059803
A_55_P2063166	NM_177767	Ogfod1	270086	0,681294118	11,1299434	0,003271941
A_51_P209319	NM_016752	Slc35b1	110172	0,680651703	8,905566038	0,000236389
A_55_P2077958	NM_008463	Klra5	16636	0,678051084	6,350339623	8,66E-05
A_51_P326191	NM_009251	Serpina3g	20715	0,676524768	7,540792453	0,065975736
A_51_P500082	NM_001110517	Gm14446	667373	0,674287926	6,863490566	0,030129721
A_55_P2004526	NM_010650	Klra8	16639	0,673631579	7,179490566	0,001262137
A_55_P2009116	NR_033558	F830002L21Rik	414125	0,671359133	8,156264151	0,020450715
A_55_P2097279	NM_011095	Pirb	18733	0,669510836	9,742660377	0,038609517
A_51_P499195	NM_024253	Nkg7	72310	0,669419505	9,272245283	0,028886904
A_52_P425890	NM_011407	Slfn1	20555	0,668622291	7,076283019	2,91E-05
A_55_P2136786	NM_133906	Zkscan1	74570	0,667193498	14,69909434	0,013472454
A_55_P2102065	NM_001122660	Gm10639	100042314	0,665702786	6,425471698	0,020806546
A_55_P2038262	NM_001164327	Phf11b	236451	0,66545356	8,479	0,003938959
A_55_P2042813	NM_001039647	Gbp11	634650	0,664758514	7,029603774	4,19E-05
A_51_P377452	NM_008677	Ncf4	17972	0,663467492	11,62390566	2,85E-05
A_51_P231320	NM_008611	Mmp8	17394	0,662424149	6,969943396	0,035613561
A_51_P238722	NM_010740	Cd93	17064	0,659747678	8,092660377	0,000357686
A_55_P2043837	NM_028791	Cmtr1	74157	0,659441176	10,09596226	1,97E-05
A_55_P1981479	NM_008326	Irgm1	15944	0,657244582	6,639792453	3,84E-05
A_66_P133928	NM_026126	Fundc2	67391	0,656267802	12,9164717	0,001135028
A_55_P1958867	NM_001112668	Gm9790	100042265	0,655589783	8,927169811	0,019805468
A_55_P2151143	NM_001166630	Dynlt1c	100040563	0,654896285	10,18750943	0,01502002
A_51_P501248	NM_025367	Sphk1	20698	0,654102167	7,081018868	0,01609795
A_55_P2032718	NM_010651	Klra9	16640	0,65221517	7,235018868	1,22E-06
A_51_P312485	NM_013521	Fpr1	14293	0,651119195	8,169037736	0,000515478
A_51_P499061	NM_173755	Ube2o	217342	0,649835913	15,05401887	0,009680214
A_55_P2035757	NR_026561	Gm8884	667933	0,648243034	7,227830189	0,000141568
A_55_P2142863	NM_030253	Parp9	80285	0,646009288	7,394	1,78E-05
A_52_P232580	NM_145508	Dyrk3	226419	0,643695046	6,82890566	0,000349548
A_55_P2016675	NM_031373	Ogfr	72075	0,643433437	8,16454717	0,000685274
A_55_P2075200	NM_023141	Tor3a	30935	0,641786378	6,853603774	0,000597687
A_55_P1976112	NM_178692	C130074G19Rik	226777	0,640914861	11,52532075	0,017964873
A_51_P123625	NM_008392	Irg1	16365	0,640628483	6,174188679	2,53E-05
A_51_P357744	NM_010592	Jund	16478	0,640366873	13,23983019	0,003797084
A_55_P1956827	NM_031373	Ogfr	72075	0,638831269	10,37986792	0,002479771
A_55_P2024595	NM_001033436	Atxn7l1	380753	0,638670279	9,763339623	0,032383086
A_55_P2163857	NM_001146007	Trim12c	319236	0,638041796	7,150584906	0,000160945
A_51_P519251	NM_019738	Nupr1	56312	0,636095975	7,190622642	0,012310539
A_51_P192860	NM_008248	Hint1	15254	0,635775542	11,87530189	0,007278383
A_55_P1990919	NM_013759	Msrb1	27361	0,6355	12,35432075	0,009443613
A_52_P652316	NM_153058	Mapre2	212307	0,633142415	8,551622642	0,048283213
A_51_P281089	NM_011313	S100a6	20200	0,63270743	11,17184906	0,029904404
A_55_P1981959	NM_001163470	Trafd1	231712	0,630899381	11,20937736	3,77E-05

A_55_P2085716	NM_026217	Atg12	67526	0,630342105	9,537471698	0,003820288
A_55_P2008740	NM_010186	Fcgr1	14129	0,628221362	6,293358491	0,008251643
A_55_P1959425	NM_030696	Slc16a3	80879	0,627280186	8,013962264	0,009443613
A_55_P1983044	NM_001081687	Gm5150	381484	0,627091331	7,229188679	0,010771677
A_65_P19395	NM_010380	H2-D1	14964	0,62577709	11,99045283	0,018195949
A_55_P2083894	XM_001478221	Gm3697	100042151	0,625198142	6,723245283	0,068201853
A_55_P2114938	NM_001159417	Irf9	16391	0,624495356	8,710169811	0,002255956
A_51_P515965	NM_008685	Nfe2	18022	0,624325077	10,37422642	0,031672779
A_55_P1965448	XM_907154	Gm14989	632883	0,620315789	12,58837736	0,054053854
A_51_P500424	NM_133763	Dnttip1	76233	0,620103715	9,288566038	5,79E-07
A_66_P119034	NM_013737	Pla2g7	27226	0,616665635	9,031245283	0,003874741
A_55_P2016376	NM_030174	Mctp1	78771	0,616113003	8,18154717	0,093520064
A_55_P1962918	NM_001033450	Mnda	381308	0,616044892	6,365169811	0,003880831
A_51_P212754	NM_009369	Tgfbi	21810	0,614919505	9,297207547	0,036812699
A_51_P294555	NM_001033632	Ifitm6	213002	0,612874613	8,489150943	0,008112602
A_52_P416327	NM_178687	Cd226	225825	0,612735294	9,806924528	0,06865149
A_52_P192426	NM_011609	Tnfrsf1a	21937	0,611708978	8,335056604	0,002518654
A_55_P2001518	NM_023898	Pde6h	78600	0,610044892	6,673018868	0,000316586
A_55_P1985337	NM_031395	Sytl3	83672	0,609176471	7,587207547	0,034173131
A_55_P2071176	NM_153511	Il1f9	215257	0,608911765	6,312377358	1,22E-07
A_55_P2117681	NM_026406	Rnf115	67845	0,606763158	9,031018868	0,003505279
A_55_P2030284	NM_021406	Trem1	58217	0,604921053	7,026358491	0,000761365
A_52_P42069	NM_021381	Prokr1	58182	0,604086687	7,270735849	0,008028577
A_51_P263246	NM_008748	Dusp8	18218	0,60345356	6,424773585	9,57E-06
A_51_P241457	NM_013532	Lilrb4	14728	0,60255418	6,693245283	0,000402809
A_55_P2001871	NM_009479	Uros	22276	0,602190402	7,720320755	0,000160826
A_51_P161021	NM_008332	Ifit2	15958	0,602105263	6,885849057	0,004750559
A_52_P64514	NM_025992	Herc6	67138	0,602034056	6,47	0,000183144
A_55_P2052924	NM_009156	Sepw1	20364	0,601871517	10,97673585	0,02595351
A_52_P432685	NM_146012	Ctdsp2	52468	0,601119195	8,554113208	0,028391682
A_55_P2030209	NM_025854	Cir1	66935	0,600718266	9,100528302	0,015254664
A_55_P2094966	NM_178216	Hist2h3c1	15077	0,596366873	7,413056604	0,001931048
A_55_P2165869	NM_009883	Cebpb	12608	0,594772446	8,930132075	0,038596389
A_55_P2094831	NM_021471	Slco1c1	58807	0,59475387	7,513301887	0,031159464
A_52_P117408	NM_009375	Tg	21819	0,594498452	7,397886792	0,000172585
A_52_P550173	NM_013730	Slamf1	27218	0,594065015	8,063849057	0,015744574
A_51_P401907	NM_001082547	Gm5483	433016	0,593656347	6,894584906	0,078791852
A_52_P91235	NM_017367	Ccni	12453	0,593363777	11,75250943	0,023586148
A_55_P2060303	NM_008624	Mras	17532	0,592178019	7,368849057	0,027499331
A_55_P2006277	NM_010956	Ogdh	18293	0,591611455	8,778528302	0,000269086
A_52_P426768	NM_019563	Cited4	56222	0,590913313	7,076660377	0,000379954
A_55_P2103115	NM_172843	Tor1aip2	240832	0,589170279	7,46609434	9,57E-06
A_55_P2137121	NM_001013817	Sp140	434484	0,588665635	7,090207547	0,000785306
A_51_P430423	NM_007398	Ada	11486	0,588196594	6,866245283	0,000119923
A_55_P2158102	NM_009921	Camp	12796	0,587119195	7,73709434	0,022506001
A_55_P2048022	NR_003631	Gm6578	625347	0,586136223	8,552830189	0,009112054
A_51_P474459	NM_007707	Socs3	12702	0,586106811	8,651849057	0,011386721
A_51_P208240	NM_019418	Tnfsf14	50930	0,58100774	7,074962264	0,000324464
A_55_P2106150	NM_021790	Cenpk	60411	0,58076161	7,954962264	0,019268735
A_55_P2108763	NM_013673	Sp100	20684	0,578729102	7,954792453	0,000380922

A_55_P2110713	NM_007585	Anxa2	12306	0,578719814	10,90637736	0,014945442
A_55_P2067505	NM_030696	Slc16a3	80879	0,578131579	7,973754717	0,017829376
A_52_P309337	NM_019648	Metap2	56307	0,577167183	7,980320755	0,04755479
A_55_P2020128	NM_011303	Dhrs3	20148	0,576623839	9,733037736	0,060676996
A_55_P2182483	NM_010581	Cd47	16423	0,576065015	11,77939623	3,34E-05
A_51_P454993	NM_178874	Tmcc2	68875	0,574643963	8,622886792	0,023578057
A_51_P464029	NM_021890	Fads3	60527	0,573978328	6,396471698	1,27E-06
A_55_P2025765	NM_007403	Adam8	11501	0,573941176	8,421811321	0,017696698
A_55_P2035286	NM_010931	Uhrf1	18140	0,573721362	7,430320755	0,003067638
A_52_P559498	NM_007713	Clk3	102414	0,573464396	7,394169811	2,01E-07
A_55_P1995092	NM_010391	H2-Q10	15007	0,57303096	8,532132075	0,001831692
A_55_P1978895	NM_177578	Skint3	195564	0,570789474	6,478622642	0,000755013
A_51_P109050	NM_023824	Paqr4	76498	0,570701238	7,118679245	0,08660221
A_55_P1998011	NM_024470	Klra23	79410	0,569886997	6,918622642	0,009774091
A_51_P452629	NM_011905	Tlr2	24088	0,569719814	6,819150943	0,001368728
A_55_P2152605	NM_170756	Spata2	263876	0,569035604	7,73490566	0,001155107
A_55_P1985623	NM_029600	Abcc3	76408	0,568798762	6,907320755	0,007875638
A_55_P2069969	NM_001122660	Gm10639	100042314	0,568105263	6,248660377	0,018025084
A_55_P2016034	NM_001033207	Nlrc5	434341	0,564914861	8,188075472	0,004750559
A_51_P452779	NM_133198	Pygl	110095	0,561811146	9,338698113	0,027928446
A_55_P1972322	NM_009770	Btg3	12228	0,56119969	11,05603774	0,087626595
A_55_P2002122	NM_173402	Rgs12	71729	0,559568111	7,07545283	4,35E-05
A_51_P141818	NM_029271	Mrpl32	75398	0,558365325	8,631698113	0,015048568
A_55_P2151138	NM_001166630	Dynlt1c	100040563	0,557229102	9,985584906	0,015037736
A_55_P2039699	NM_009921	Camp	12796	0,557055728	8,365169811	0,020671125
A_51_P498631	NM_018769	Dfna5	54722	0,55694582	6,758924528	0,000545467
A_51_P237754	NM_010398	H2-T23	15040	0,554383901	11,96209434	0,011876855
A_52_P475870	NM_001164686	Tmem29	382245	0,55424613	8,158603774	0,042872085
A_51_P219789	NM_010392	H2-Q2	15013	0,553430341	11,83181132	0,005043013
A_51_P500090	NM_014194	Klra7	16638	0,552823529	6,340358491	0,001351271
A_55_P1999561	NM_001002842	Pram1	378460	0,552764706	7,185396226	0,003785163
A_55_P2033041	NM_001173460	Sirpb1b	668101	0,552249226	8,989358491	0,067465614
A_55_P2107696	NM_205821	Mrgpra6	381886	0,552181115	6,274981132	0,000201133
A_55_P2009988	NM_175093	Trib3	228775	0,548472136	6,677886792	0,019974241
A_52_P549827	NM_019946	Mgst1	56615	0,547111455	6,955075472	0,050620727
A_55_P2073099	NM_011637	Trex1	22040	0,54630031	11,17349057	0,000321971
A_51_P214747	NM_172893	Parp12	243771	0,545820433	6,273377358	0,000377684
A_55_P2106106	NM_176912	C5ar2	319430	0,545783282	6,763716981	0,0028043
A_51_P368009	NM_177733	E2f2	242705	0,545569659	14,16816981	0,046331595
A_52_P117393	NM_011604	Tlr6	21899	0,54344582	7,020584906	0,000360389
A_55_P1975903	NM_001003961	Dnmt3b	13436	0,543066563	10,01730189	0,090349984
A_51_P390538	NM_010821	Mpeg1	17476	0,54172291	9,959584906	0,076943915
A_55_P2064652	NM_001146007	Trim12c	319236	0,540744582	6,407528302	0,00022165
A_51_P110301	NM_009778	C3	12266	0,538018576	8,130698113	0,028778703
A_55_P2110875	NR_033198	2700046G09Rik	67188	0,537439628	6,725490566	0,000152173
A_55_P1978511	NM_010394	H2-Q7	15018	0,537417957	11,41018868	0,072842378
A_51_P401987	NM_019432	Tmem37	170706	0,536896285	7,101207547	0,015048568
A_55_P2016114	NM_010177	Fasl	14103	0,535640867	6,486433962	4,53E-05
A_55_P1973941	NM_011404	Slc7a5	20539	0,535578947	8,431	0,065532103
A_55_P2059904	NM_001081163	Chsy1	269941	0,533374613	7,639150943	0,00026504

A_51_P422124	NM_053090	Fam126a	84652	0,533314241	8,59045283	0,009680214
A_55_P2089080	NM_024272	Ssbp2	66970	0,533190402	9,056584906	0,012619511
A_51_P407690	NM_175195	Sppl2b	73218	0,53226935	9,466754717	0,007301627
A_52_P380369	BC030186	Phf11d	219132	0,530074303	6,797320755	0,003685095
A_55_P2043466	NM_173363	Eif5	217869	0,529803406	11,72854717	0,011218088
A_55_P2090617	NM_153510	Pilra	231805	0,529091331	6,901056604	0,002070824
A_52_P164136	NM_001042591	Arrdc3	105171	0,528642415	6,694660377	0,001201302
A_52_P547662	NM_008772	P2ry1	18441	0,526710526	7,240320755	0,0306468
A_55_P2004536	NM_010649	Klra4	16635	0,526196594	7,425018868	0,076011815
A_52_P486322	NM_019757	Fzr1	56371	0,525814241	9,987264151	0,067465614
A_55_P2101910	NM_009438	Rpl13a	22121	0,525705882	8,953754717	0,020981489
A_52_P220879	NM_009373	Tgm2	21817	0,524939628	7,156509434	0,007731256
A_51_P430900	NM_013642	Dusp1	19252	0,524010836	7,787	0,041558514
A_52_P467389	NM_023044	Slc15a3	65221	0,522934985	6,531584906	0,001314986
A_51_P308549	NM_021422	Dnaja4	58233	0,522362229	6,990320755	0,001941324
A_55_P1995195	NM_008037	Fosl2	14284	0,521582043	7,02445283	0,000160945
A_51_P414889	NM_027320	Ifi35	70110	0,519842105	7,645358491	0,009606594
A_55_P1966660	NM_001025208	LOC547349	547349	0,519809598	9,77290566	0,090029678
A_55_P2184189	BC003730	Ncf2	17970	0,519637771	8,607226415	0,032742574
A_55_P2042713	NM_011822	Pigq	14755	0,519595975	10,50235849	0,002964356
A_55_P2009121	NM_021604	Agrn	11603	0,518137771	6,841188679	0,002054421
A_51_P311362	AK015429	4930449E01Rik	74864	0,517552632	8,622037736	0,099172572
A_55_P1964615	NM_133763	Dnttip1	76233	0,51751548	7,316377358	0,000517793
A_55_P2133248	AK134184	Gm8995	668139	0,517286378	8,156471698	0,000350976
A_55_P2173629	NM_001115154	Samd3	268288	0,515606811	6,806075472	0,000589465
A_55_P2077956	NM_001099918	Klrb1	100043861	0,513993808	6,764320755	0,000298957
A_55_P2037787	XM_001478340	Gm3798	100042341	0,513893189	6,522490566	0,057400562
A_55_P1967133	NM_177741	Ppp1r3b	244416	0,512787926	9,17409434	0,010342499
A_51_P514029	NM_010780	Cma1	17228	0,512377709	6,43309434	0,020676128
A_55_P2129047	NM_133211	Tlr7	170743	0,510244582	6,59409434	5,50E-05
A_51_P466759	NM_028993	Mau2	74549	0,509069659	7,135584906	1,28E-05
A_55_P1990653	NM_001111062	Comt	12846	0,508764706	8,342622642	0,024313265
A_51_P235726	NM_023892	Icam4	78369	0,507713622	6,965981132	0,00288003
A_55_P1998912	NM_013711	Txnrd2	26462	0,505243034	10,11756604	0,090061002
A_55_P2011061	NM_173007	Tspan12	269831	0,504922601	6,205245283	0,014869814
A_52_P514407	NM_013793	Klra15	27423	0,504654799	7,087943396	0,035752543
A_51_P374900	NM_028808	P2ry13	74191	0,504171827	6,296358491	2,57E-06
A_51_P514712	NM_001039530	Parp14	547253	0,502931889	7,867943396	0,009112054
A_52_P390127	AK138801	Klrc1	16641	0,502791022	6,587981132	0,001030904
A_55_P2009774	NM_009573	Zic1	22771	0,500828173	8,643924528	0,02248494
A_66_P124497	NM_133976	Imp3	102462	-0,500249226	9,013283019	0,026712416
A_55_P1987290	NR_004413	Rnu1b6	19847	-0,500626935	11,20273585	0,056422071
A_51_P322265	NM_027817	Grap	71520	-0,501198142	8,352207547	0,002891602
A_51_P162471	NM_134081	Dnajc9	108671	-0,50226161	8,659679245	0,023911366
A_55_P2088385	NM_011618	Tnnt1	21955	-0,502724458	7,722660377	0,061192919
A_55_P2037585	NM_133977	Trf	22041	-0,507487616	6,91245283	0,088315896
A_51_P278868	NM_010387	H2-DMb1	14999	-0,508517028	9,351113208	0,077990894
A_55_P2177539	NM_011434	Sod1	20655	-0,508614551	11,09575472	0,004427294
A_51_P104897	NM_080553	Itpr3	16440	-0,509452012	8,637660377	0,015539934
A_52_P552589	NM_008279	Map4k1	26411	-0,509537152	8,794188679	0,024313265

A_55_P2170225	NM_172507	Sh3bgrl2	212531	-0,511726006	7,141698113	0,032551164
A_51_P417469	NM_026616	Rnaseh2c	68209	-0,513752322	9,744471698	0,007724
A_55_P2097206	NM_009793	Camk4	12326	-0,514781734	6,623132075	0,010060292
A_55_P2057877	NM_001163554	Pou2f2	18987	-0,514917957	7,119377358	0,000430652
A_52_P532227	NM_007901	S1pr1	13609	-0,515244582	8,622113208	0,022892462
A_55_P1956973	NM_011602	Tln1	21894	-0,516828173	10,93133962	0,080862226
A_51_P475995	NM_178060	Thra	21833	-0,51898452	7,150301887	0,001282124
A_55_P1953356	NM_001024716	Triobp	110253	-0,519082043	12,2319434	0,058424088
A_55_P1964648	NM_001037719	Btla	208154	-0,519393189	8,436037736	0,082979756
A_51_P247157	NM_008258	Hn1	15374	-0,520071207	8,214735849	0,001135028
A_51_P100034	NM_027162	Mif4gd	69674	-0,520373065	8,389245283	0,016573398
A_55_P1960053	NM_001042489	Hvcn1	74096	-0,521349845	9,118924528	0,064784011
A_55_P2139181	NM_145575	Cald1	109624	-0,521869969	6,556679245	0,02484757
A_51_P327778	NM_011070	Pfdn2	18637	-0,522239938	7,857811321	0,048283213
A_55_P1965101	NM_001081643	Xlr3b	574437	-0,522987616	7,144396226	0,08314768
A_55_P2019083	NM_001114334	Rps6kb1	72508	-0,523283282	8,411849057	0,015744574
A_55_P1997751	NM_138659	Prpf8	192159	-0,523806502	10,08213208	0,041019218
A_52_P517896	NM_028636	Man2c1	73744	-0,524882353	8,265716981	0,005024815
A_52_P282500	NM_001039472	Kif21b	16565	-0,524897833	10,05235849	0,081465312
A_66_P133112	AK075658	Gm14635	100043946	-0,526294118	12,75762264	0,088745789
A_52_P78023	NM_172397	Limd2	67803	-0,526611455	12,52677358	0,022503962
A_55_P2021841	NM_007590	Calm3	12315	-0,529865325	12,65601887	0,01617229
A_55_P2005853	NM_001037098	Nacc2	67991	-0,530117647	7,360075472	0,007711143
A_55_P1996299	NM_001039055	Pofut1	140484	-0,530201238	7,829339623	0,001155107
A_51_P357573	NM_145575	Cald1	109624	-0,530698142	6,699132075	0,026713762
A_66_P129048	NM_001190445	2610002J02Rik	67513	-0,531527864	9,212716981	0,001577031
A_55_P2050877	NM_138752	Plekhg2	101497	-0,532702786	8,646207547	0,007446848
A_55_P2118570	NM_007645	Cd37	12493	-0,53321517	10,21164151	0,000164603
A_51_P490348	NM_025423	1110059E24Rik	66206	-0,533447368	7,232264151	0,005264724
A_55_P2174490	NM_007645	Cd37	12493	-0,533464396	7,958169811	0,002293462
A_51_P407323	NM_007976	F5	14067	-0,534	7,199566038	0,072720231
A_51_P517870	NM_198429	Nfatc1	18018	-0,534760062	7,879264151	0,022412794
A_51_P418526	NM_027324	Sfxn1	14057	-0,535029412	8,021226415	0,013040657
A_55_P2305420	AK038263	D9Wsu90e	27962	-0,535625387	6,433924528	0,001748339
A_51_P519364	NM_145986	Fam83f	213956	-0,537665635	14,17307547	0,058694006
A_52_P418665	NM_029512	Ttpal	76080	-0,537749226	8,002339623	0,010432633
A_55_P2183208	NM_001045532	Prl2c1	666317	-0,538283282	6,528471698	0,011871383
A_51_P232858	NM_025974	Rpl14	67115	-0,538382353	6,251377358	0,000960907
A_51_P302358	NM_008518	Ltb	16994	-0,540673375	11,32532075	0,037962847
A_55_P2089233	NM_011136	Pou2af1	18985	-0,540879257	9,07345283	0,065933624
A_52_P453884	NM_019739	Foxo1	56458	-0,542879257	7,831735849	0,000833518
A_55_P2142430	NM_001033350	Bank1	242248	-0,544255418	7,108566038	0,000169027
A_52_P260994	NM_013710	Fgd2	26382	-0,547770898	7,59145283	0,097365752
A_55_P2015426	NM_133728	Asnsd1	70396	-0,549897833	7,996396226	0,005147837
A_55_P2409088	AK138015	BB163080	106459	-0,551642415	7,17709434	0,004725668
A_55_P2004746	NM_183319	Xkrx	331524	-0,553770898	7,471301887	0,011015933
A_55_P2072985	NM_001076554	Sptan1	20740	-0,554369969	8,817528302	0,005173529
A_55_P1957038	NM_181796	Gstp2	14869	-0,554849845	11,18941509	0,004217308
A_51_P406796	NM_013873	Sult4a1	29859	-0,555379257	6,157754717	0,00388012
A_66_P124858	NM_001024474	Diras2	68203	-0,557840557	7,032754717	0,006180773

A_55_P2152009	NM_021344	Tesc	57816	-0,558260062	9,377339623	0,036652445
A_51_P193794	NM_008512	Lrp1	16971	-0,559859133	7,010207547	0,07656962
A_51_P427663	NM_007725	Cnn2	12798	-0,561196594	10,25443396	0,036850893
A_55_P2154595	NM_017404	Mrpl39	27393	-0,564789474	6,906528302	0,002122728
A_51_P421664	NM_133739	Tmem123	71929	-0,565278638	8,888	0,014247952
A_55_P2124586	NM_001081642	Xlr4a	434794	-0,566798762	7,180396226	0,035256199
A_55_P1980302	NM_025498	Psenen	66340	-0,566989164	11,15288679	0,068575062
A_51_P140942	NM_018739	Rp9	55934	-0,568298762	8,398358491	0,003068395
A_51_P121447	NM_054099	1110038F14Rik	117171	-0,569210526	7,788943396	0,005664358
A_51_P292357	NM_016959	Rps3a1	20091	-0,569232198	11,48664151	0,0216602
A_51_P365409	NM_028040	Rpusd4	71989	-0,570301858	6,308169811	2,60E-06
A_51_P143805	NM_025339	Tmem42	66079	-0,573778638	7,63845283	0,001666883
A_66_P125862	NR_015487	A930005H10Rik	68161	-0,574922601	7,991660377	0,011156832
A_55_P1981964	AK004668	Tnfrsf13b	57916	-0,580153251	8,168226415	0,003188176
A_55_P1987770	NM_025498	Psenen	66340	-0,582198142	11,74916981	0,053634025
A_52_P276792	NM_053202	Foxp1	108655	-0,583678019	7,838698113	0,001438135
A_55_P2391619	BG063913	AI449595	58901	-0,585113003	6,452566038	0,073109841
A_55_P2079076	NM_001044308	Cacna1i	239556	-0,585450464	6,834150943	0,000592971
A_55_P2150734	BC004786	Igh-VJ558	16061	-0,58593808	7,128358491	0,098788873
A_51_P202050	NM_008052	Dtx1	14357	-0,588743034	8,853	0,024313265
A_51_P384148	NM_013649	Ryk	20187	-0,589552632	6,220150943	0,026729564
A_55_P2024431	NM_008064	Gaa	14387	-0,590130031	7,349679245	0,003441537
A_52_P417859	NM_025757	Gid4	66771	-0,591356037	6,837886792	0,000160945
A_55_P2174743	NM_018747	Akap7	432442	-0,594770898	8,306603774	0,045655328
A_51_P391955	NM_029723	Dapl1	76747	-0,594890093	8,947943396	0,0131758
A_55_P2008907	NM_009609	Actg1	11465	-0,597475232	12,91807547	0,010332345
A_52_P125350	NM_023210	Anp32e	66471	-0,601105263	8,667509434	0,027984913
A_55_P1974554	NM_145625	Eif4b	75705	-0,606679567	8,858716981	0,038114201
A_51_P199135	NM_009856	Cd83	12522	-0,60729257	7,922320755	0,006018432
A_55_P2046101	NM_021365	Xlr4b	27083	-0,608452012	7,173169811	0,033266181
A_55_P2007196	NM_001177307	Aldoa	11674	-0,614823529	12,17541509	0,00081807
A_51_P232281	NM_011109	Pla2g2d	18782	-0,618083591	6,530716981	0,000150694
A_52_P252737	NM_011241	Rangap1	19387	-0,620032508	7,813283019	0,00953741
A_55_P2087622	NM_010215	Il4i1	14204	-0,624772446	8,254113208	0,051940574
A_55_P2076871	AK083328	Lef1	16842	-0,625159443	10,70715094	0,012127965
A_55_P2043182	NM_153175	Gimap6	231931	-0,625580495	10,528	0,003990645
A_55_P2083121	NM_001164567	Vill	22351	-0,625911765	6,763528302	0,004530881
A_55_P2113523	NM_001177884	Whsc1	107823	-0,627342105	8,068603774	0,090029678
A_51_P221632	NM_026091	1700037H04Rik	67326	-0,629250774	12,09030189	0,080381789
A_55_P1971729	NM_007758	Cr2	12902	-0,62969195	8,396584906	0,021902855
A_55_P2019577	NR_027818	1500011B03Rik	66236	-0,629832817	7,256622642	0,012410199
A_55_P1953301	NM_146126	Sord	20322	-0,635834365	7,625735849	0,034526305
A_55_P2070686	NM_027057	Wdfy1	69368	-0,640074303	11,36224528	0,00141712
A_52_P77245	NM_172277	Snx8	231834	-0,641739938	7,626471698	0,000600734
A_55_P2052425	NM_145482	Setd4	224440	-0,642388545	7,404150943	0,006047609
A_55_P2099650	NM_053195	Slc24a3	94249	-0,647026316	8,69054717	0,071306362
A_51_P154485	NM_145141	Fcrla	98752	-0,649065015	7,355433962	0,0028043
A_66_P115061	NM_025695	Smc6	67241	-0,649396285	7,119962264	0,018145875
A_55_P2332731	AK051661	D130062J21Rik	100038651	-0,655721362	8,679283019	0,024685976
A_52_P199614	NM_001146022	Wdfy4	545030	-0,665964396	9,191169811	0,007539411

A_51_P266248	AK134885	Ighv14-2	668421	-0,666181115	10,32415094	0,00953741
A_55_P2112737	NM_007393	Actb	11461	-0,668354489	15,49101887	0,011262914
A_52_P613498	NM_026127	4833420G17Rik	67392	-0,669181115	7,981075472	0,067739361
A_55_P2117345	NM_013517	Fcer2a	14128	-0,676247678	6,882924528	0,000202519
A_55_P2023542	NM_007719	Ccr7	12775	-0,677027864	11,1370566	0,003355547
A_52_P277104	NM_001033350	Bank1	242248	-0,677297214	7,699018868	0,001105833
A_51_P362054	NM_025983	Atp5e	67126	-0,678987616	11,7544717	4,90E-05
A_55_P2088825	NM_053202	Foxp1	108655	-0,679106811	9,319811321	0,006945732
A_55_P2101585	NM_010016	Cd55	13136	-0,67999226	7,57490566	0,012003364
A_55_P2180869	NM_029865	Ocel1	77090	-0,682162539	8,368245283	0,010108345
A_52_P279425	NM_032465	Cd96	84544	-0,68247678	7,259867925	0,000575583
A_51_P428372	NM_023785	Ppbp	57349	-0,686363777	12,37515094	0,098788873
A_55_P2068812	AK203298	Pkm	18746	-0,687391641	10,92454717	0,017700698
A_55_P2079079	NM_009844	Cd19	12478	-0,692565015	8,288339623	0,006316208
A_55_P2117614	NM_028075	Tnfrsf13c	72049	-0,694013932	7,604584906	0,00561741
A_55_P2116650	NR_002860	A130040M12Rik	319269	-0,694947368	10,21486792	0,085028845
A_51_P397920	NM_001033279	D17Wsu92e	224647	-0,695520124	10,15260377	0,034033226
A_55_P2337138	AK003962	Ago2	239528	-0,697399381	8,477283019	0,032547082
A_55_P2028600	NM_022410	Myh9	17886	-0,69751548	11,39609434	0,006986487
A_55_P1986296	NM_178598	Tagln2	21346	-0,702106811	13,02456604	0,01134711
A_51_P322273	NM_008975	Ptp4a3	19245	-0,706229102	12,661	0,013535438
A_51_P138044	NM_019739	Foxo1	56458	-0,70947678	8,285188679	0,000327965
A_55_P2030506	NM_018739	Rp9	55934	-0,715749226	7,470528302	0,000123272
A_55_P2106175	NM_008102	Gch1	14528	-0,72325387	8,951132075	0,057684859
A_52_P390944	NM_016803	Chst3	53374	-0,727379257	7,96709434	0,000685234
A_55_P2001920	NM_010227	Flna	192176	-0,734233746	11,45175472	0,00659087
A_55_P2003221	NM_031247	Gimap3	83408	-0,735981424	9,635981132	0,018025084
A_55_P2297485	AK075904	Ago2	239528	-0,739433437	8,518037736	0,013784338
A_66_P136801	NR_002864	Peg13	353342	-0,748380805	7,620830189	2,85E-06
A_55_P1957168	NM_133777	Ube2s	77891	-0,751544892	11,20975472	0,078791852
A_52_P644972	NM_175245	Mzt1	76789	-0,751916409	7,489150943	0,067756598
A_51_P269084	NM_175329	Chchd10	103172	-0,755928793	11,35683019	0,007148187
A_52_P478256	NM_178614	Samm50	68653	-0,756060372	7,563283019	0,074942278
A_51_P449325	NM_008206	H2-Oa	15001	-0,757883901	8,65545283	0,000130642
A_51_P247359	NM_016933	Ptprcap	19265	-0,763419505	11,0900566	0,002476394
A_51_P505823	NM_028013	Endod1	71946	-0,776605263	8,54409434	0,019591046
A_55_P2144526	NM_001080381	Fam65b	193385	-0,778126935	9,052754717	0,009696752
A_51_P457528	NM_007630	Ccnb2	12442	-0,782264706	8,360830189	0,014468837
A_51_P194628	NM_173037	Tango6	272538	-0,788243034	10,61298113	0,022892462
A_51_P415809	NM_026954	Tusc1	69136	-0,801470588	8,966150943	0,02278191
A_55_P2108943	NM_009835	Ccr6	12458	-0,808249226	8,086867925	0,005524517
A_51_P270733	NM_009303	Syngr1	20972	-0,817043344	8,102509434	0,07606077
A_55_P2002757	NM_008528	Blnk	17060	-0,817956656	9,381358491	0,002762857
A_55_P2136906	NM_009514	Vpreb3	22364	-0,820944272	8,790169811	0,004608473
A_55_P2113703	NM_019866	Spib	272382	-0,825565015	8,711660377	0,002120516
A_55_P2082929	NM_010389	H2-Ob	15002	-0,840743034	8,331660377	0,003940468
A_51_P226269	NM_025427	Rgcc	66214	-0,84203096	7,799433962	5,98E-05
A_55_P2019113	NM_001024848	Apol7b	278679	-0,84623065	7,136075472	0,006870251
A_51_P284608	NM_001042605	Cd74	16149	-0,852091331	11,25315094	0,005085532
A_55_P1953459	NM_001171147	Yap1	22601	-0,862690402	7,373792453	0,007705719

A_51_P365019	NM_010295	Gclc	14629	-0,900608359	6,852169811	2,57E-06
A_55_P2085295	NM_198411	Inf2	70435	-0,905029412	9,258584906	0,00486563
A_52_P70796	NM_007551	Cxcr5	12145	-0,922167183	8,127	4,82E-05
A_55_P2000107	NM_009720	Atox1	11927	-0,942287926	11,49043396	0,002667884
A_51_P342652	NM_008339	Cd79b	15985	-0,957462848	11,19990566	0,001645397
A_51_P378298	NM_026976	Faim3	69169	-0,964986068	9,829679245	0,000639315
A_66_P123055	NR_033535	Gm10845	100038734	-0,975589783	9,400113208	0,063385275
A_52_P553890	NM_016780	Itgb3	16416	-1,000154799	10,65392453	0,009247668
A_55_P2055875	NM_025368	Josd2	66124	-1,032278638	7,545584906	0,055797199
A_66_P132493	NM_031181	Siglece	83382	-1,038413313	11,89141509	0,09474964
A_55_P2146560	NM_207105	H2-Ab1	14961	-1,065071207	12,04635849	0,000870906
A_51_P286814	NM_011424	Ncor2	20602	-1,0785	8,515867925	6,89E-06
A_55_P1962747	NM_207105	H2-Ab1	14961	-1,084640867	11,99275472	0,000290242
A_55_P2064351	NM_011703	Vipr1	22354	-1,098657895	7,832641509	0,007296493
A_55_P1983754	NM_025557	Pcp4l1	66425	-1,115540248	9,870471698	0,089417616
A_51_P295215	NM_172943	Alkbh5	268420	-1,120078947	9,318962264	1,20E-05
A_51_P237752	NM_008986	Ptrf	19285	-1,125379257	9,224415094	0,020628085
A_52_P483336	NM_007641	Ms4a1	12482	-1,136952012	9,384207547	0,00013066
A_51_P105520	NM_153057	Nomo1	211548	-1,143179567	9,098886792	0,00045239
A_55_P2211164	AK017236	5330406M23Rik	76671	-1,143894737	7,673924528	0,004836778
A_51_P103397	NM_011708	Vwf	22371	-1,146165635	9,693433962	0,018183469
A_55_P2060630	NM_027886	Stk11ip	71728	-1,180390093	7,26254717	0,056358549
A_55_P2078735	NR_015519	AI662270	100043636	-1,257419505	6,636962264	0,004552273
A_55_P1956457	NM_029998	6030458C11Rik	77877	-1,265931889	7,272528302	0,003371748
A_52_P37894	NM_009945	Cox7a2	12866	-1,317122291	10,35673585	6,24E-05
A_52_P562676	NM_013873	Sult4a1	29859	-1,345410217	8,022830189	0,004262494
A_55_P1991770	NM_019417	Pdlim4	30794	-1,39148452	8,181018868	1,18E-07
A_55_P2141938	NR_027875	1810058I24Rik	67705	-1,463908669	6,904584906	0,011149522
A_55_P2008061	NM_019923	Itpr2	16439	-1,489427245	9,117113208	4,13E-06
A_51_P254471	NM_007465	Birc2	11797	-1,63005418	7,60190566	3,11E-06
A_55_P2017929	NM_013492	Clu	12759	-1,862770898	11,74803774	0,001313209
A_51_P343517	NM_010742	Ly6d	17068	-2,105518576	8,364867925	3,83E-05
