# Supplementary material for: Of mice and men: the host response to influenza virus infection
Source: Mamm Genome. 2018 Jun 15;29(7):446–70. doi: 10.1007/s00335-018-9750-y (PMC6132725; doi:10.1007/s00335-018-9750-y)
Supplement: Supplementary file 7 — Supplementary material 7 (PDF 123 KB) [file 335_2018_9750_MOESM7_ESM.pdf]

PROBE	ACCNUM	SYMBOL	ENTREZID	logFC	AveExpr	adj.P.Val
A_51_P421876	NM_016850	Irf7	54123	3,296032591	10,70365741	1,95E-22
A_51_P359570	NM_010501	Ifit3	15959	3,141036566	9,212074074	4,91E-22
A_51_P387123	NM_011854	Oasl2	23962	3,093007154	9,684194444	1,28E-24
A_55_P1998943	NM_145211	Oas1a	246730	2,985067568	10,22832407	3,87E-20
A_51_P304170	NM_023386	Rtp4	67775	2,715515898	10,04310185	2,92E-24
A_55_P2086433	NM_145209	Oasl1	231655	2,691571542	9,355342593	1,65E-20
A_51_P154842	NM_145153	Oas1f	243262	2,615720191	9,37987037	1,65E-20
A_55_P2019719	NM_145227	Oas2	246728	2,607496025	8,894	5,83E-23
A_55_P1972872	NM_001005858	I830012O16Rik	667370	2,530499205	8,005537037	5,64E-19
A_66_P101942	AK019325	Gm9706	677168	2,479613672	12,51125926	5,49E-14
A_51_P327751	NM_008331	Ifit1	15957	2,432302862	8,924842593	7,16E-20
A_55_P1961499	NM_010741	Ly6c1	17067	2,38691097	9,576240741	4,85E-16
A_52_P90363	NM_029803	Ifi27l2a	76933	2,376387122	13,27682407	1,02E-17
A_55_P2103698	NM_015783	Isg15	100038882	2,345444356	15,35191667	1,20E-15
A_51_P346938	NM_029796	Lrg1	76905	2,304209857	9,751222222	1,71E-10
A_55_P2064771	NM_010741	Ly6c1	17067	2,293054054	12,04116667	1,64E-17
A_51_P183812	NM_011410	Slfn4	20558	2,263883148	7,98312037	1,32E-14
A_66_P128537	NM_015783	Isg15	100038882	2,255665342	11,54834259	3,78E-12
A_55_P1998471	NM_009114	S100a9	20202	2,205472178	14,84983333	1,19E-12
A_51_P359636	NM_011150	Lgals3bp	19039	2,200718601	10,68087037	7,05E-21
A_55_P2048607	NM_017370	Hp	15439	2,175369634	11,91547222	2,37E-15
A_55_P2114953	NM_011909	Usp18	24110	2,173054054	8,754444444	4,59E-17
A_52_P398925	NM_173869	Stfa2l1	268885	2,155852146	8,768981481	1,70E-07
A_55_P1998942	NM_145211	Oas1a	246730	2,145850556	8,301481481	3,87E-20
A_51_P256827	NM_013650	S100a8	20201	2,067368045	12,42182407	1,37E-05
A_66_P139618	NM_001082545	Stfa2	20862	2,047139905	9,014611111	3,84E-07
A_51_P203955	NM_010260	Gbp2	14469	1,981961844	9,060981481	4,54E-09
A_55_P1962305	NM_139198	Plac8	231507	1,981864865	11,69644444	1,06E-14
A_55_P2146254	NM_001112715	Ifitm1	68713	1,966537361	10,30364815	6,61E-12
A_51_P254656	NM_008230	Hdc	15186	1,937874404	9,491685185	1,12E-14
A_51_P288138	NM_008039	Fpr2	14289	1,897949921	8,399564815	3,59E-15
A_51_P401907	NM_001082547	Gm5483	433016	1,866343402	7,960555556	2,52E-06
A_51_P128876	NM_025378	Ifitm3	66141	1,849777424	11,39935185	1,45E-16
A_52_P700056	NM_001081957	Wfdc17	100034251	1,847713037	8,712731481	1,70E-05
A_52_P559975	NM_009909	Cxcr2	12765	1,841346582	9,361222222	5,19E-13
A_51_P231320	NM_008611	Mmp8	17394	1,832840223	7,988305556	4,85E-16
A_55_P2062246	NM_001145164	Tgtp2	100039796	1,802101749	11,54368519	1,10E-21
A_51_P413866	NM_008198	Cfb	14962	1,79527027	7,488592593	6,92E-14
A_51_P212754	NM_009369	Tgfbi	21810	1,7796407	10,29614815	1,92E-13
A_55_P1957459	NM_013532	Lilrb4	14728	1,716937997	9,597185185	3,51E-13
A_55_P1990919	NM_013759	Msrb1	27361	1,706054054	13,29546296	6,70E-15
A_55_P2081164	NM_001169153	Cd300lf	246746	1,700062798	9,306916667	1,41E-12
A_51_P262171	NM_008326	Irgm1	15944	1,691515898	8,749972222	1,21E-23
A_52_P422494	NM_145634	Cd300lf	246746	1,689848172	9,118064815	1,02E-12
A_55_P1994042	NM_001139519	Zbp1	58203	1,675843402	9,489027778	1,63E-18
A_51_P265495	NM_010738	Ly6a	110454	1,667430843	10,22585185	3,63E-10
A_51_P174961	NM_007972	F10	14058	1,666419714	8,43562963	4,65E-13
A_51_P294555	NM_001033632	Ifitm6	213002	1,664072337	9,409638889	5,72E-17
A_55_P2004016	NM_030209	Crispld2	78892	1,663479332	7,59437963	3,71E-10
A_51_P246653	NM_020008	Clec7a	56644	1,658694754	9,945777778	4,71E-13

A_51_P181517	NM_144559	Fcgr4	246256	1,657066773	8,551574074	1,47E-17
A_55_P2110758	NM_177083	B430306N03Rik	320148	1,653955485	8,908148148	1,29E-18
A_51_P470079	NM_010555	Il1r2	16178	1,643441176	7,70362037	3,11E-11
A_52_P425839	NM_181596	Retnlg	245195	1,595478537	11,72646296	3,83E-09
A_55_P1955308	NM_001002898	Sirpb1a	320832	1,560505564	9,976	1,59E-09
A_51_P325856	NM_026985	Mcemp1	69189	1,558689984	8,491314815	3,13E-14
A_55_P2052016	NM_030209	Crispld2	78892	1,546930048	7,358462963	4,74E-10
A_55_P1998578	NM_030694	Ifitm2	80876	1,541699523	9,389555556	1,98E-18
A_55_P2179413	NM_011150	Lgals3bp	19039	1,540108903	9,969083333	1,61E-17
A_55_P2171116	NM_001145953	Lgals3	16854	1,529147854	10,84292593	1,04E-11
A_52_P472324	NM_011414	Slpi	20568	1,514941971	11,62539815	5,70E-10
A_66_P139683	NM_021394	Zbp1	58203	1,51036089	7,695259259	9,78E-18
A_55_P2091461	NM_007609	Casp4	12363	1,503631161	8,914148148	6,72E-08
A_55_P2005783	NM_027835	Ifih1	71586	1,493653418	7,65037037	8,97E-18
A_55_P1966833	NM_001037713	Xaf1	327959	1,493009539	8,31037037	5,28E-16
A_55_P2033041	NM_001173460	Sirpb1b	668101	1,489712242	9,812111111	1,13E-11
A_51_P472867	NM_145226	Oas3	246727	1,486067568	8,037731481	9,95E-13
A_51_P362066	NM_007695	Chil1	12654	1,46668124	7,834212963	3,84E-10
A_51_P474459	NM_007707	Socs3	12702	1,463940382	9,444805556	1,74E-14
A_51_P326191	NM_009251	Serpina3g	20715	1,463046105	8,300722222	2,48E-09
A_51_P169516	NM_001085501	Ppp1r3d	228966	1,455982512	8,1105	3,33E-12
A_55_P2092826	NM_010730	Anxa1	16952	1,455852941	10,46717593	1,16E-09
A_55_P2046145	AK137448	Gm10693	675749	1,445852941	9,645824074	2,44E-12
A_51_P225793	NM_175181	Prr5l	72446	1,438431638	9,337268519	1,92E-16
A_55_P1999561	NM_001002842	Pram1	378460	1,435372814	7,970731481	8,98E-13
A_55_P2066578	BC010546	Ifi204	15951	1,431655008	7,464037037	6,15E-11
A_51_P464918	NM_019453	Mefv	54483	1,424432432	8,252	1,63E-15
A_51_P108226	NM_183249	Wfdc21	66107	1,423648649	7,609962963	5,36E-06
A_51_P281089	NM_011313	S100a6	20200	1,419024642	11,91732407	6,66E-12
A_51_P452779	NM_133198	Pygl	110095	1,418732909	10,10938889	1,33E-11
A_55_P2151685	NM_011088	Pira11	18724	1,418077901	9,310351852	2,44E-12
A_55_P1955305	NM_001002898	Sirpb1a	320832	1,417979332	9,452166667	1,10E-12
A_55_P2016462	NM_021274	Cxcl10	15945	1,41759062	6,928194444	2,39E-16
A_51_P444447	NM_007679	Cebpd	12609	1,415379968	9,967944444	8,42E-08
A_55_P2172001	NM_001172588	Mrgpra2a	668727	1,414960254	8,042833333	3,98E-10
A_51_P129012	NM_009735	B2m	12010	1,412381558	14,10603704	2,89E-11
A_55_P2079579	NM_011094	Pira7	18730	1,411310811	9,714509259	2,67E-12
A_52_P487686	NM_001082546	BC100530	100034684	1,400071542	8,753925926	0,000889056
A_51_P212782	NM_008361	Il1b	16176	1,396609698	9,329083333	1,96E-09
A_52_P375312	NM_001005421	Amica1	270152	1,396417329	9,809657407	4,52E-08
A_55_P2017929	NM_013492	Clu	12759	1,395298092	13,37186111	4,60E-06
A_55_P2019557	NM_153101	Mrgpra2b	235712	1,390950715	8,0935	9,52E-10
A_52_P52618	NM_007780	Csf2rb	12983	1,385621622	9,030907407	1,09E-14
A_55_P1959425	NM_030696	Slc16a3	80879	1,385209062	8,738212963	2,02E-13
A_55_P1966838	NM_001037713	Xaf1	327959	1,377281399	7,471722222	0,003812014
A_55_P1958250	NM_030743	Rnf114	81018	1,360161367	13,28763889	1,89E-13
A_55_P1984168	NM_007609	Casp4	12363	1,354395072	8,429305556	2,73E-07
A_55_P2015687	NM_001164323	Phf11d	219132	1,349829889	8,356148148	6,86E-12
A_66_P119034	NM_013737	Pla2g7	27226	1,34836407	9,734055556	4,77E-11
A_55_P1960238	NM_172659	Slc2a6	227659	1,343212242	9,056231481	2,35E-11
A_55_P2048279	NM_205820	Tlr13	279572	1,341167727	9,177712963	1,06E-10

A_55_P2059606	NM_019963	Stat2	20847	1,338186804	8,204935185	8,01E-16
A_55_P2175880	NM_019946	Mgst1	56615	1,337504769	7,98987963	4,06E-14
A_51_P364485	NM_009396	Tnfaip2	21928	1,336725755	8,619787037	3,54E-15
A_55_P2067505	NM_030696	Slc16a3	80879	1,329567568	8,6775	2,64E-13
A_51_P487690	NM_133871	Ifi44	99899	1,324338633	6,961740741	8,17E-15
A_55_P2040838	NM_001166672	Gm14548	100038909	1,324161367	8,829972222	3,33E-12
A_51_P151576	NM_001001999	Gp1bb	14724	1,322108903	9,916712963	5,98E-07
A_52_P186937	NM_020557	Cmpk2	22169	1,321314785	7,344462963	6,46E-17
A_51_P176156	NM_001173459	LOC100038947	100038947	1,321284579	8,532648148	1,00E-11
A_51_P151182	NM_011940	Ifi202b	26388	1,32072655	7,578648148	1,61E-06
A_55_P2079560	NM_011090	Lilra6	18726	1,319209062	9,715990741	1,38E-11
A_55_P2031436	NM_001164036	Ly6e	17069	1,319081081	11,68631481	1,27E-11
A_51_P405476	NM_010185	Fcer1g	14127	1,317529412	12,57672222	1,79E-19
A_51_P333274	NM_013542	Gzmb	14939	1,310205087	7,880203704	4,35E-16
A_55_P1999102	NM_023734	Pi16	74116	1,304987281	8,943481481	0,000970943
A_55_P2097279	NM_011095	Pirb	18733	1,302069157	10,39480556	5,69E-09
A_55_P2110713	NM_007585	Anxa2	12306	1,298155803	11,58838889	1,03E-10
A_55_P2016852	NM_011777	Zyx	22793	1,296738474	12,99256481	2,99E-09
A_51_P262515	NM_172603	Phf11a	219131	1,288905405	8,194138889	4,51E-12
A_51_P192800	NM_008175	Grn	14824	1,280936407	8,198796296	1,21E-14
A_66_P120567	NM_010730	Anxa1	16952	1,267305246	8,670074074	1,44E-09
A_51_P149714	NM_026835	Ms4a6d	68774	1,267262321	7,214074074	1,64E-17
A_52_P363216	NM_133219	Gcnt2	14538	1,265205087	7,80987037	1,54E-12
A_55_P1963017	NM_001082543	Stfa1	20861	1,262506359	7,968638889	0,001532139
A_51_P275454	NM_009099	Trim30a	20128	1,252987281	8,158351852	7,87E-15
A_55_P2059154	NM_001013371	Dtx3l	209200	1,245415739	9,003046296	1,89E-16
A_55_P2032167	NM_008327	Ifi202b	26388	1,232668521	7,457694444	9,82E-07
A_55_P2109857	NM_009061	Rgs2	19735	1,225400636	10,73068519	1,62E-11
A_51_P499838	NM_009763	Bst1	12182	1,225199523	7,387194444	1,11E-11
A_55_P2068812	AK203298	Pkm	18746	1,222948331	12,00891667	2,33E-10
A_55_P1974577	NM_028728	Nfam1	74039	1,221680445	9,612518519	3,24E-11
A_55_P2137611	NM_019440	Irgm2	54396	1,220269475	8,195787037	1,53E-17
A_55_P2118441	NM_010846	Mx1	17857	1,214844992	7,167305556	4,52E-08
A_55_P2066230	NM_001172117	Hck	15162	1,208737679	10,16344444	1,34E-11
A_55_P2472435	NM_018734	Gbp3	55932	1,206170111	7,853685185	6,17E-10
A_52_P315976	NM_009416	Tpm2	22004	1,205753577	7,54437037	2,12E-05
A_51_P312121	NM_011723	Xdh	22436	1,204172496	7,215787037	1,47E-17
A_55_P2121408	NM_009416	Tpm2	22004	1,201646264	7,76887963	1,16E-05
A_52_P527800	NM_145158	Emilin2	246707	1,20086407	9,937990741	2,30E-09
A_52_P456640	NM_010208	Fgr	14191	1,198403816	8,336481481	4,14E-13
A_52_P549827	NM_019946	Mgst1	56615	1,191693959	7,575472222	4,83E-14
A_51_P512992	NM_178676	Entpd3	215446	1,190716216	6,763861111	1,61E-08
A_66_P122086	NM_001039720	9030619P08Rik	105892	1,190306836	7,640462963	5,97E-10
A_55_P2037697	NM_001076679	Gm9733	751864	1,188116057	7,711814815	4,12E-12
A_55_P1975560	NM_008329	Ifi204	15951	1,180660572	9,329824074	8,33E-08
A_55_P2165869	NM_009883	Cebpb	12608	1,178385533	9,524324074	8,42E-09
A_51_P261517	NM_011662	Tyrobp	22177	1,177804452	11,69292593	1,17E-09
A_55_P2172396	NM_178796	A530064D06Rik	328830	1,175220986	7,339685185	4,89E-08
A_55_P2081488	NM_009402	Pglyrp1	21946	1,17254372	9,253703704	6,29E-09
A_55_P2079561	NM_011090	Lilra6	18726	1,160769475	10,35451852	1,20E-09
A_55_P2070079	NM_010708	Lgals9	16859	1,156960254	8,228555556	9,08E-14

A_55_P2011084	AK172348	Trim30b	244183	1,156854531	7,468953704	3,19E-11
A_55_P1999213	NM_146142	Tdrd7	100121	1,150697138	8,862175926	1,84E-09
A_51_P258372	NM_030691	Igsf6	80719	1,150415739	9,113953704	1,89E-13
A_51_P352968	NM_008538	Marcks	17118	1,144345787	9,045824074	2,37E-09
A_51_P441426	NM_019932	Pf4	56744	1,143436407	15,11558333	9,81E-07
A_55_P1983523	NM_145437	Cd300ld	217305	1,141658983	8,609453704	3,14E-05
A_55_P2093994	NM_030700	Maged2	80884	1,136999205	8,932231481	6,76E-08
A_51_P237752	NM_008986	Ptrf	19285	1,136147059	10,40632407	0,000493868
A_51_P383032	NM_010819	Clec4d	17474	1,133211447	9,815018519	1,17E-07
A_55_P2130178	NM_010233	Fn1	14268	1,131542925	8,456287037	5,46E-05
A_51_P153995	NM_018762	Gp9	54368	1,128844197	12,64255556	1,14E-06
A_51_P308298	NM_172118	Myl9	98932	1,127644674	14,24152778	0,000317477
A_55_P2051334	NR_004446	H2-K2	630499	1,12581717	12,67698148	8,35E-06
A_52_P635338	NM_010194	Fes	14159	1,122967409	9,334175926	1,01E-10
A_55_P2035757	NR_026561	Gm8884	667933	1,121180445	7,763657407	9,85E-09
A_55_P2098697	NM_009396	Tnfaip2	21928	1,119900636	7,626898148	3,54E-15
A_55_P2008740	NM_010186	Fcgr1	14129	1,119569157	6,835259259	1,17E-10
A_52_P151853	NM_001025262	Tpd52	21985	1,112387917	8,111574074	2,05E-12
A_52_P553890	NM_016780	Itgb3	16416	1,111596979	11,77412037	2,53E-05
A_65_P19395	NM_010380	H2-D1	14964	1,111193164	12,52749074	1,78E-08
A_55_P2136880	NM_008654	Ppp1r15a	17872	1,109503975	11,29683333	5,31E-10
A_51_P103397	NM_011708	Vwf	22371	1,109433227	10,86449074	0,000280302
A_51_P490023	NM_009450	Tubb2a	22151	1,108751987	11,85825926	3,63E-06
A_52_P459564	NM_010575	Itga2b	16399	1,106461049	10,13727778	7,63E-05
A_66_P139546	NM_008344	Igfbp6	16012	1,103279014	7,514009259	4,14E-09
A_55_P1962304	NM_139198	Plac8	231507	1,1011407	8,59125	8,01E-16
A_52_P199633	NM_199146	Trim30d	209387	1,098410175	7,415055556	9,49E-16
A_66_P133328	NM_001025261	Tpd52	21985	1,09663593	9,342222222	9,02E-13
A_51_P159503	AK173199	Rnf213	672511	1,095391892	8,252046296	1,24E-10
A_55_P1981479	NM_008326	Irgm1	15944	1,093715421	7,153574074	1,71E-22
A_55_P1956130	BC025170	LOC68395	68395	1,093665342	11,79365741	9,56E-10
A_55_P1957353	XM_907302	Slfn13l	631406	1,092869634	8,742259259	1,14E-09
A_51_P499698	NM_026414	Asprv1	67855	1,084946741	8,288712963	1,23E-05
A_55_P2054409	NM_011089	Pira2	18725	1,084029412	8,089731481	3,83E-10
A_55_P2130970	NM_001163576	Parp10	671535	1,082426073	10,38657407	4,89E-10
A_55_P1958255	NM_030743	Rnf114	81018	1,082298887	9,083222222	2,46E-09
A_55_P2141699	NM_001033135	Rnf149	67702	1,080064388	8,44075	1,77E-10
A_51_P474169	NM_144883	Proser2	227545	1,077435612	8,294537037	1,70E-07
A_52_P577384	NM_010531	Il18bp	16068	1,075830684	6,818231481	9,94E-13
A_55_P2065991	NM_016740	S100a11	20195	1,073603339	9,719175926	2,23E-08
A_51_P161021	NM_008332	Ifit2	15958	1,072054054	7,404555556	1,67E-08
A_52_P192426	NM_011609	Tnfrsf1a	21937	1,071167727	8,849712963	1,12E-11
A_52_P365011	NM_010194	Fes	14159	1,069860095	8,486611111	1,26E-11
A_52_P282762	NM_010851	Myd88	17874	1,06958744	8,025953704	4,76E-15
A_51_P241457	NM_013532	Lilrb4	14728	1,068683625	7,209481481	1,06E-14
A_52_P466090	NM_018851	Samhd1	56045	1,068605723	8,733222222	4,02E-12
A_51_P213691	NM_011324	Scnn1a	20276	1,067145469	7,913898148	8,21E-09
A_51_P112355	NM_018738	Igtp	16145	1,066007154	11,02082407	6,92E-14
A_51_P156274	NM_010736	Ltbr	17000	1,065555644	7,63825	2,27E-15
A_55_P1992959	NM_173019	Pfkfb4	270198	1,065321145	9,056648148	2,99E-09
A_51_P108108	NM_153074	Lrrc25	211228	1,061283784	7,932175926	2,18E-13

A_55_P1962404	NM_201410	Ugt1a6b	394435	1,058127981	8,192101852	6,89E-09
A_52_P223809	NM_030150	Dhx58	80861	1,057322734	7,039314815	2,13E-17
A_55_P2125049	AB359227	Gm11127	100529082	1,057232114	11,90187037	1,02E-09
A_55_P1983044	NM_001081687	Gm5150	381484	1,052779809	7,725731481	1,36E-09
A_51_P493558	NM_145417	Rnpep	215615	1,048091415	8,022842593	3,42E-16
A_55_P2057936	NM_001135115	Gm12250	631323	1,045478537	7,986611111	8,97E-14
A_51_P228768	NM_011409	Slfn3	20557	1,041708267	6,742527778	4,12E-06
A_55_P2166501	NM_009851	Cd44	12505	1,041604134	8,228074074	1,45E-13
A_55_P2045136	AK153212	I830127L07Rik	546643	1,041534976	6,75237963	1,87E-08
A_51_P279062	NM_027763	Treml1	71326	1,038555644	12,29475	0,000114054
A_55_P2025765	NM_007403	Adam8	11501	1,038495231	8,92762037	2,41E-06
A_52_P467389	NM_023044	Slc15a3	65221	1,038395866	7,055611111	5,30E-17
A_52_P84096	NM_001081274	Pgd	110208	1,038325119	9,041768519	7,82E-12
A_55_P1981804	NM_010326	Gp1ba	14723	1,03567965	8,77625	7,95E-06
A_51_P235687	NM_009663	Alox5ap	11690	1,035657393	11,89267593	5,29E-08
A_51_P219789	NM_010392	H2-Q2	15013	1,034561208	12,34227778	3,44E-09
A_51_P234864	NM_178639	Sfxn5	94282	1,034317965	7,237611111	3,24E-11
A_51_P169693	NM_198095	Bst2	69550	1,032870429	8,143972222	1,17E-09
A_55_P2060107	NM_011099	Pkm	18746	1,032744038	9,964944444	0,001775066
A_52_P592909	NM_026384	Dgat2	67800	1,030937997	7,678148148	1,19E-07
A_55_P2129354	NM_153510	Pilra	231805	1,030856121	8,450268519	1,08E-07
A_51_P507801	NM_028784	F13a1	74145	1,027	10,18468519	3,88E-07
A_55_P2095345	NM_001177302	Rara	19401	1,025741653	10,30317593	6,55E-07
A_55_P1978506	NM_207648	H2-Q6	110557	1,025511129	11,26769444	3,13E-09
A_55_P1962010	NM_019985	Clec1b	56760	1,024020668	10,84555556	1,23E-06
A_55_P1979463	NM_012021	Prdx5	54683	1,021286963	9,774564815	5,24E-10
A_51_P502456	NR_004446	H2-K2	630499	1,017825914	11,8260463	2,58E-06
A_55_P2035320	NM_017373	Nfil3	18030	1,015885533	7,490981481	3,99E-13
A_51_P203675	NM_021407	Trem3	58218	1,015116852	7,014601852	2,17E-16
A_51_P515965	NM_008685	Nfe2	18022	1,014966614	10,84585185	3,63E-14
A_51_P514085	NM_013606	Mx2	17858	1,014766296	7,163185185	0,00077785
A_51_P193146	NM_028595	Ms4a6c	73656	1,013889507	8,116731481	2,91E-13
A_66_P112006	NM_010395	H2-T10	15024	1,012026232	8,137101852	6,75E-17
A_51_P390538	NM_010821	Mpeg1	17476	1,011847377	10,45868519	3,16E-06
A_51_P237754	NM_010398	H2-T23	15040	1,011065978	12,45612037	1,38E-07
A_66_P109986	NM_001111058	Cd33	12489	1,009116057	7,018666667	2,03E-10
A_55_P1992910	NM_013755	Gyg	27357	1,008505564	9,051777778	4,09E-10
A_55_P2016049	NM_001163014	Gp6	243816	1,007170906	9,363157407	3,28E-07
A_55_P1996973	NM_029000	Gvin1	74558	1,003458665	10,66923148	1,98E-06
A_55_P1978511	NM_010394	H2-Q7	15018	1,001984102	11,90407407	1,01E-08
A_51_P123625	NM_008392	Irg1	16365	1,001767886	6,630925926	5,38E-13
A_51_P109449	NM_008519	Ltb4r1	16995	1,001305246	8,250814815	4,97E-12
A_55_P2052834	NM_010734	Lst1	16988	0,996500795	11,34011111	7,50E-09
A_55_P2025248	NM_010751	Mxd1	17119	0,99536725	11,07948148	4,15E-11
A_51_P341465	NM_009970	Csf2ra	12982	0,994786963	9,477907407	5,82E-08
A_52_P327664	NM_153564	Gbp5	229898	0,994209857	7,28162963	2,42E-10
A_55_P1956827	NM_031373	Ogfr	72075	0,993714626	10,83173148	8,17E-15
A_55_P2022585	NM_011093	Pira6	18729	0,990354531	9,66987037	3,75E-11
A_55_P2100824	XM_992161	LOC676689	676689	0,986672496	11,38825926	5,90E-07
A_55_P2012201	NM_007471	App	11820	0,984506359	7,750157407	1,22E-10
A_51_P198434	NM_001001892	H2-K1	14972	0,983919714	12,90149074	6,49E-07

A_55_P1981494	AY989854	H2-Bl	14963	0,982360095	8,753157407	1,26E-06
A_51_P172853	NM_009841	Cd14	12475	0,981464229	7,309425926	8,45E-06
A_55_P2030284	NM_021406	Trem1	58217	0,980297297	7,481185185	2,17E-10
A_51_P425048	NM_010392	H2-Q2	15013	0,979962639	10,48325	2,19E-09
A_51_P240019	NM_013755	Gyg	27357	0,979171701	9,095296296	5,52E-10
A_55_P2049647	NM_001001892	H2-K1	14972	0,978884738	11,81080556	1,27E-08
A_55_P2026233	NM_023738	Uba7	74153	0,977883943	11,4132963	5,03E-08
A_66_P134453	NM_001033780	I830077J02Rik	433638	0,977418919	7,598712963	2,44E-10
A_55_P2142863	NM_030253	Parp9	80285	0,974939587	7,830425926	5,32E-17
A_55_P2129348	NM_153510	Pilra	231805	0,971608108	7,477231481	1,37E-11
A_55_P2038262	NM_001164327	Phf11b	236451	0,969234499	8,904546296	4,38E-09
A_51_P389988	NM_016917	Slc40a1	53945	0,967573132	7,817731481	0,000174357
A_55_P1962011	NM_019985	Clec1b	56760	0,964871224	12,4597037	8,90E-06
A_52_P535484	NM_029000	Gvin1	74558	0,955281399	11,21257407	4,38E-09
A_52_P408757	NM_010188	Fcgr3	14131	0,953613672	7,847166667	8,43E-13
A_55_P2034300	NM_001168256	Tmem40	94346	0,951850556	12,09337037	4,29E-05
A_51_P514712	NM_001039530	Parp14	547253	0,951285374	8,339453704	2,23E-10
A_51_P146753	NM_007781	Csf2rb2	12984	0,948285374	8,364898148	7,95E-12
A_55_P1992617	NM_019985	Clec1b	56760	0,948107313	12,17530556	4,91E-06
A_51_P519251	NM_019738	Nupr1	56312	0,944411765	7,609685185	1,11E-08
A_55_P2058942	NM_026316	Aldh3b1	67689	0,943701113	7,499462963	3,98E-10
A_55_P2088401	NM_010399	H2-T9	15051	0,937108903	7,656916667	9,56E-10
A_52_P207335	NM_025944	Tmem246	67063	0,934926073	8,164009259	0,000634524
A_55_P1983448	NM_011311	S100a4	20198	0,934434022	10,34440741	7,80E-06
A_51_P439996	NM_008148	Gp5	14729	0,934426073	8,655166667	1,21E-05
A_55_P2019699	NM_001139520	Samhd1	56045	0,93431399	10,59947222	1,01E-09
A_55_P2019601	NM_033616	Csprs	114564	0,933172496	8,109101852	1,95E-07
A_55_P2105271	NM_001114088	Pdlim7	67399	0,931397456	9,595944444	1,62E-07
A_51_P490795	NM_010751	Mxd1	17119	0,931295707	9,388462963	2,42E-10
A_51_P498631	NM_018769	Dfna5	54722	0,930802862	7,197037037	2,06E-08
A_55_P2034864	NM_023716	Tubb2b	73710	0,927459459	8,021981481	4,23E-05
A_66_P114784	NM_013737	Pla2g7	27226	0,92550318	7,383935185	7,89E-11
A_55_P2114938	NM_001159417	Irf9	16391	0,924895072	9,120018519	1,61E-13
A_55_P2000062	NM_008390	Irf1	16362	0,922169316	10,14926852	2,06E-07
A_51_P110301	NM_009778	C3	12266	0,920662957	8,568648148	9,28E-08
A_55_P1999648	NM_007408	Plin2	11520	0,919897456	7,913064815	5,09E-11
A_52_P266132	NM_008013	Fgl2	14190	0,915746423	7,75575	3,42E-07
A_51_P202633	NM_015766	Ebi3	50498	0,914775835	7,957555556	2,29E-11
A_52_P467449	NM_007440	Alox12	11684	0,91458903	10,98589815	8,67E-05
A_51_P248666	NM_021893	Cd274	60533	0,914170111	7,620111111	3,89E-09
A_55_P2066727	NM_001164118	Serpinb6a	20719	0,907796502	10,67218519	0,051302984
A_55_P1959818	NM_001168256	Tmem40	94346	0,905483307	11,87162963	7,20E-05
A_55_P2169356	AK156879	Gm1966	434223	0,902224165	8,890925926	0,00016934
A_51_P222657	NM_009775	Tspo	12257	0,901991256	10,63697222	2,36E-12
A_55_P2009121	NM_021604	Agrn	11603	0,901964229	7,273453704	5,40E-11
A_52_P661	NM_175750	Plxna4	243743	0,901828299	7,934537037	0,000131748
A_51_P187082	NM_008062	G6pdx	14381	0,900439587	9,30237963	3,46E-12
A_51_P156857	NM_134133	Smim3	106878	0,90022655	10,55702778	6,73E-05
A_55_P1998416	NM_008330	Ifi47	15953	0,898506359	11,20523148	8,69E-10
A_55_P2000107	NM_009720	Atox1	11927	0,891521463	12,43909259	0,000131043
A_55_P2048912	NM_001033308	Themis2	230787	0,891068362	7,94637037	2,44E-12

A_52_P161488	NM_019948	Clec4e	56619	0,889790938	8,303083333	0,000307311
A_66_P112305	NM_053214	Myo1f	17916	0,887928458	9,032777778	8,74E-08
A_55_P2066116	NM_033601	Bcl3	12051	0,883978537	8,867453704	6,13E-10
A_55_P1995092	NM_010391	H2-Q10	15007	0,88222655	8,931194444	1,77E-11
A_55_P2016623	XM_204772	Gm5068	277089	0,881934817	8,429759259	2,51E-07
A_51_P140321	NM_026779	Mocos	68591	0,87976868	7,033157407	8,39E-15
A_55_P2125208	NM_001172205	Arid5a	214855	0,879463434	9,996212963	9,79E-08
A_51_P186476	NM_013612	Slc11a1	18173	0,878012719	7,780277778	3,12E-10
A_51_P384629	NM_009983	Ctsd	13033	0,876710652	11,38094444	9,85E-09
A_51_P245989	NM_009915	Ccr2	12772	0,876306836	7,587314815	7,84E-08
A_66_P119968	NM_199311	Clec4a1	269799	0,87613593	8,787638889	3,91E-08
A_55_P2099560	NM_031159	Apobec1	11810	0,875677266	8,852648148	2,01E-08
A_55_P2024461	NM_198100	Tbkbp1	73174	0,874940382	7,950731481	2,56E-11
A_55_P2047768	NM_148942	Serpinb6c	97848	0,874151828	10,10775	0,071452009
A_55_P2063736	NM_008147	Gp49a	14727	0,872490461	6,785935185	1,02E-12
A_55_P1962918	NM_001033450	Mnda	381308	0,872176471	6,741925926	0,001153843
A_55_P2037343	NM_010391	H2-Q10	15007	0,871630366	8,227287037	0,023113425
A_55_P2124273	NM_053214	Myo1f	17916	0,867613672	8,695740741	3,70E-08
A_52_P425890	NM_011407	Slfn1	20555	0,8675469	7,431018519	1,49E-13
A_55_P2003996	NM_008207	H2-T24	15042	0,865961844	8,357314815	2,87E-09
A_51_P203182	NM_138310	Apobr	171504	0,863944356	7,357814815	6,15E-11
A_52_P380369	BC030186	Phf11d	219132	0,861489666	7,197574074	4,86E-11
A_55_P1966660	NM_001025208	LOC547349	547349	0,861468203	10,17682407	0,001840849
A_55_P2106106	NM_176912	C5ar2	319430	0,861279014	7,158194444	4,13E-09
A_55_P2027278	NM_144560	Gas2l1	78926	0,859494436	11,77264815	0,000674374
A_55_P1986531	NM_001191012	G6b	106722	0,858918124	8,984194444	0,002100546
A_51_P329928	NM_013750	Phlda3	27280	0,854691574	8,308592593	1,55E-05
A_55_P2020128	NM_011303	Dhrs3	20148	0,853000795	10,11078704	3,90E-05
A_51_P452629	NM_011905	Tlr2	24088	0,8519531	7,198657407	4,74E-10
A_52_P507214	NM_013599	Mmp9	17395	0,85191256	6,87512963	1,52E-07
A_55_P1972582	XM_136331	Gm4955	240921	0,849961844	7,062851852	1,27E-05
A_55_P2052181	NM_025534	Ccdc82	66396	0,849526232	9,510259259	4,74E-10
A_55_P2118037	NM_007547	Sirpa	19261	0,848983307	8,088916667	1,05E-08
A_55_P2184189	BC003730	Ncf2	17970	0,844937202	8,99987963	6,76E-08
A_52_P117393	NM_011604	Tlr6	21899	0,844154213	7,404166667	4,38E-09
A_55_P1979341	NM_007806	Cyba	13057	0,843694754	11,73835185	7,42E-08
A_55_P2016675	NM_031373	Ogfr	72075	0,840036566	8,509462963	7,57E-16
A_51_P500082	NM_001110517	Gm14446	667373	0,83445151	7,193518519	1,76E-07
A_51_P105520	NM_153057	Nomo1	211548	0,831631955	10,07852778	0,003904102
A_51_P173100	NM_145925	Pttg1ip	108705	0,831003975	11,09700926	7,44E-05
A_55_P2070869	NM_008491	Lcn2	16819	0,830580286	10,63827778	0,000923188
A_51_P482711	NM_053272	Dhcr24	74754	0,830460254	10,31034259	5,38E-05
A_51_P412835	NM_007829	Daxx	13163	0,829811606	9,660898148	3,07E-09
A_52_P253004	NM_022327	Ralb	64143	0,829806836	8,354453704	4,42E-12
A_51_P312437	NM_025522	Dhrs7	66375	0,829604134	9,247814815	5,25E-06
A_51_P293688	NM_026405	Rab32	67844	0,82895787	7,472342593	4,51E-11
A_55_P2139473	NM_001161724	Ilk	16202	0,828674086	10,57764815	1,40E-05
A_51_P424499	NM_011660	Txn1	22166	0,82832194	9,936583333	2,19E-12
A_55_P1978465	NM_023124	H2-Q8	15019	0,827598569	8,988675926	2,29E-10
A_52_P8324	NM_026516	Tmem178	68027	0,827286169	6,916314815	9,01E-07
A_55_P2192961	NM_175648	Trim30b	244183	0,827143084	6,812981481	4,74E-10

A_52_P66371	NM_178420	Nlrx1	270151	0,827033386	7,984259259	4,43E-06
A_52_P135873	XM_001475453	Gm2744	100040381	0,82450159	9,115583333	0,000875863
A_52_P604629	NM_153287	Csrnp1	215418	0,823748808	7,959185185	6,75E-10
A_55_P2029574	NM_008898	Por	18984	0,822833863	8,714675926	2,97E-09
A_55_P1965000	NM_172689	Ddx58	230073	0,822800477	8,992064815	3,28E-10
A_55_P1962084	NM_013549	Hist2h2aa1	15267	0,822593005	12,61398148	4,06E-06
A_51_P165330	NM_025796	Mrpl33	66845	0,821338633	10,59259259	8,71E-08
A_55_P2181738	NM_013590	Lyz1	17110	0,819774245	12,10081481	4,76E-05
A_55_P2076545	NM_007976	F5	14067	0,818621622	9,038907407	6,48E-05
A_55_P2017636	NM_011580	Thbs1	21825	0,816430843	9,693759259	0,007617948
A_55_P2004137	NM_008138	Gnai2	14678	0,814654213	12,3024537	6,12E-06
A_55_P1978216	NM_134158	AF251705	140497	0,81377027	8,442583333	1,61E-06
A_51_P293982	NM_153119	Plekho2	102595	0,810984897	7,763027778	5,34E-09
A_51_P321150	NM_017372	Lyz2	17105	0,810922893	9,60375	9,46E-06
A_55_P1988158	NM_016903	Esd	13885	0,810675676	11,24446296	0,000254075
A_51_P448664	NM_011539	Tbxas1	21391	0,80936248	8,486592593	1,10E-06
A_55_P2103055	NM_010344	Gsr	14782	0,809259936	8,490287037	0,000253726
A_55_P2041075	NM_001081636	Ccnd3	12445	0,808991256	11,76825	7,21E-06
A_55_P1953169	NM_011315	Saa3	20210	0,808631955	6,624268519	0,001480374
A_55_P2006277	NM_010956	Ogdh	18293	0,803126391	9,116731481	2,19E-07
A_55_P2022251	NM_009878	Cdkn2d	12581	0,80218283	10,00405556	1,29E-09
A_55_P2019577	NR_027818	1500011B03Rik	66236	0,801628776	8,031675926	3,23E-05
A_55_P2001218	NM_009855	Cd80	12519	0,795337043	8,23812963	2,03E-06
A_51_P189361	NM_027950	Osgin1	71839	0,795259141	7,57487037	1,77E-11
A_55_P2001920	NM_010227	Flna	192176	0,794259141	12,25918519	4,60E-05
A_51_P423578	NM_011408	Slfn2	20556	0,792804452	8,79012963	3,88E-07
A_55_P2024888	NM_021281	Ctss	13040	0,792676471	10,9524537	2,92E-05
A_51_P358722	NM_173414	Lancl3	236285	0,792124801	6,974046296	0,000126056
A_55_P1965150	NM_021549	Pnkp	59047	0,790755167	9,693166667	2,70E-07
A_52_P195018	NM_139206	Arap3	106952	0,789329094	8,389277778	1,08E-08
A_55_P1999349	NM_027722	Nudt4	71207	0,787515898	9,025564815	4,47E-07
A_55_P1956973	NM_011602	Tln1	21894	0,786152623	11,65527778	0,0002855
A_55_P2075200	NM_023141	Tor3a	30935	0,785984102	7,162074074	8,41E-07
A_51_P428372	NM_023785	Ppbp	57349	0,785469793	13,15939815	0,000951795
A_55_P1966843	NR_002890	Gm12070	654472	0,785327504	12,78138889	2,39E-07
A_55_P1994032	NM_013842	Xbp1	22433	0,784781399	9,39325	9,13E-10
A_55_P2066613	NM_008879	Lcp1	18826	0,783934022	8,950787037	1,53E-05
A_55_P2106956	NR_004447	Gm5523	433273	0,783797297	12,7600463	1,41E-07
A_51_P405397	NM_007899	Ecm1	13601	0,783395072	11,60706481	0,000427923
A_55_P2186027	NM_008084	Gapdh	14433	0,783354531	12,90253704	1,51E-07
A_55_P2013113	NM_007523	Bak1	12018	0,781475358	8,519425926	5,80E-11
A_55_P2422164	AI851140	C130093G08Rik	320032	0,780710652	6,990166667	2,37E-08
A_55_P2045741	NM_011815	Fyb	23880	0,778432432	7,61237037	3,56E-06
A_55_P2064996	NM_009798	Capzb	12345	0,77782035	9,313333333	8,73E-07
A_55_P2021266	NM_152803	Hpse	15442	0,77754849	9,6035	1,80E-05
A_52_P516021	NM_011201	Ptpn1	19246	0,77667806	8,320638889	1,29E-10
A_55_P1990944	NM_009807	Casp1	12362	0,776040541	7,859231481	1,11E-10
A_51_P463120	NM_025760	Ptplad2	66775	0,774533386	9,639787037	4,04E-05
A_55_P2251181	AK013795	2900076A13Rik	73002	0,774465024	7,560416667	1,05E-08
A_66_P136899	NM_001159637	Nadk	192185	0,774328299	8,396175926	1,17E-09
A_55_P2133195	NM_001033767	Gm4951	240327	0,773286169	6,547814815	9,95E-06

A_55_P2058757	NM_007598	Cap1	12331	0,773172496	10,52847222	1,72E-06
A_55_P1994939	NM_008252	Hmgb2	97165	0,772825914	9,225175926	2,05E-06
A_51_P208240	NM_019418	Tnfsf14	50930	0,772350556	7,39587963	2,60E-07
A_55_P2243431	BC052902	Gdap10	100504486	0,771848172	7,355064815	2,23E-08
A_55_P1971074	NM_015804	Atp11a	50770	0,771419714	7,035888889	2,30E-07
A_51_P256202	NM_022325	Ctsz	64138	0,770535771	10,44901852	1,44E-08
A_55_P2090617	NM_153510	Pilra	231805	0,770171701	7,239092593	2,71E-08
A_55_P2044653	NM_009999	Cyp2b10	13088	0,768719396	7,743509259	0,001180721
A_51_P343252	NM_009842	Cd151	12476	0,7683593	10,1862037	0,000115259
A_52_P196105	NM_175274	Ttyh3	78339	0,767658188	9,01687037	1,21E-05
A_51_P369803	NM_013585	Psmb9	16912	0,765824324	9,379231481	2,99E-07
A_52_P681310	NM_011113	Plaur	18793	0,765606518	8,350435185	1,66E-06
A_55_P2086811	NM_001013817	Sp140	434484	0,763647059	8,052092593	1,76E-07
A_55_P1957413	NM_019391	Lsp1	16985	0,762348172	13,13555556	8,15E-05
A_55_P1990785	NM_027678	Zranb3	226409	0,762348172	7,552055556	0,000643623
A_51_P247249	NM_009662	Alox5	11689	0,76200159	7,785259259	6,01E-07
A_55_P2139385	NR_002890	Gm12070	654472	0,761525437	11,84163889	1,56E-08
A_55_P2033120	NM_029688	Srxn1	76650	0,761445151	10,16190741	0,000468879
A_66_P121787	NM_010156	Samd9l	209086	0,761221781	8,901342593	3,22E-05
A_51_P381558	NM_133914	Rasa4	54153	0,759670111	7,07225	3,53E-10
A_55_P2004511	NM_001169153	Cd300lf	246746	0,759423688	8,190787037	5,02E-06
A_52_P220879	NM_009373	Tgm2	21817	0,757973768	7,487675926	1,24E-10
A_52_P126266	NM_182997	Prkab2	108097	0,754452305	7,897527778	3,14E-06
A_55_P2072631	NM_001083810	Prr5l	72446	0,753208267	7,089351852	6,85E-18
A_55_P2168667	NM_028030	Rbpms2	71973	0,751612878	9,199935185	0,002959954
A_55_P2090798	NM_001005863	Mtus1	102103	0,751248808	6,671009259	9,80E-09
A_51_P327796	NM_010580	Itgb5	16419	0,750742448	9,999074074	0,070325731
A_52_P463977	NM_197986	Tmem140	68487	0,750554054	10,68826852	0,000233987
A_52_P86693	NM_026790	Ifi27	52668	0,749246423	11,49366667	0,001258234
A_52_P84901	NM_133891	Slc44a1	100434	0,749056439	8,460712963	0,001129028
A_51_P370678	NM_008114	Gfi1b	14582	0,74827345	7,846	0,000307803
A_52_P409769	NM_194464	Mrvi1	17540	0,748120827	8,877777778	0,003002363
A_55_P1984243	NM_010872	Naip2	17948	0,747255962	7,172685185	1,42E-14
A_55_P1989321	NM_009121	Sat1	20229	0,745868045	9,123851852	7,64E-09
A_55_P2151601	NM_010156	Samd9l	209086	0,745381558	9,462018519	9,16E-05
A_55_P1995195	NM_008037	Fosl2	14284	0,744502385	7,347592593	4,45E-08
A_66_P116252	NM_130447	Dusp16	70686	0,743300477	7,518592593	5,83E-07
A_51_P225592	NM_001001491	Tpm4	326618	0,742470588	11,62825926	7,50E-05
A_55_P2149363	NM_172286	6430548M08Rik	234797	0,742375994	8,304694444	1,52E-05
A_55_P1990235	NM_021328	Bin3	57784	0,742360095	9,066212963	6,15E-11
A_52_P586004	NM_001033245	Hk3	212032	0,741038951	6,869101852	7,47E-13
A_55_P2066827	NM_027658	Hexim2	71059	0,740643084	7,166212963	3,99E-05
A_52_P431615	XM_001000891	Gm1966	434223	0,740290143	8,487324074	1,12E-10
A_55_P2172852	NM_025760	Ptplad2	66775	0,7399531	8,653916667	7,42E-05
A_55_P1986296	NM_178598	Tagln2	21346	0,73991097	13,78324074	0,000320753
A_55_P1970597	NM_009609	Actg1	11465	0,739275835	14,70530556	0,000418579
A_55_P2055819	NM_001037722	Adam15	11490	0,738936407	8,749925926	5,19E-06
A_51_P407323	NM_007976	F5	14067	0,738445946	7,896972222	0,000423841
A_55_P1982668	NM_001159637	Nadk	192185	0,737872019	8,584490741	1,15E-07
A_51_P400543	NM_019467	Aif1	11629	0,732963434	7,067833333	3,88E-07
A_55_P2007001	NM_023465	Ctnnbip1	67087	0,73232035	6,655657407	3,39E-07

A_52_P633410	NM_020590	Gabarapl1	57436	0,731555644	11,60639815	0,001077527
A_55_P1966982	NM_010344	Gsr	14782	0,731031797	10,30383333	0,001318903
A_55_P2043837	NM_028791	Cmtr1	74157	0,729103339	10,35912963	1,26E-12
A_51_P408363	NM_008823	Cfp	18636	0,728244833	8,62962963	3,82E-05
A_55_P2111264	NM_001025565	Lhx9	16876	0,725802862	6,742074074	2,97E-07
A_55_P1955656	NM_007796	Ctla2a	13024	0,72549841	15,5409537	5,39E-05
A_51_P450100	NM_026124	1110008F13Rik	67388	0,72540461	9,614712963	2,58E-10
A_55_P2081133	NM_008084	Gapdh	14433	0,725326709	11,4069537	5,83E-07
A_55_P2158404	NM_020557	Cmpk2	22169	0,724976153	6,532537037	6,21E-11
A_55_P2183597	NM_198664	Tbc1d2	381605	0,724487281	6,877231481	5,51E-08
A_51_P238448	NM_007632	Ccnd3	12445	0,72340779	9,886638889	4,00E-06
A_51_P381260	NM_008761	Fxyd5	18301	0,721424483	11,63407407	0,000154076
A_51_P243914	NM_018773	Skap2	54353	0,72140779	9,947768519	8,58E-07
A_52_P354744	NM_011401	Slc2a3	20527	0,719881558	10,2760463	0,000350572
A_51_P258690	NM_009136	Scrg1	20284	0,719824324	6,510712963	6,03E-06
A_55_P2037425	NM_008084	Gapdh	14433	0,718060413	11,66209259	7,54E-07
A_55_P2066778	NM_206536	AB124611	382062	0,717296502	8,022657407	6,35E-09
A_55_P2071858	NM_008598	Mgmt	17314	0,716413355	7,548111111	4,79E-05
A_66_P126159	NM_001080971	Tubb1	545486	0,715220191	8,388175926	0,00064513
A_51_P144868	NM_029934	Mboat7	77582	0,713553259	8,691592593	4,39E-08
A_55_P1980831	NM_029711	Arpc2	76709	0,711838633	12,91356481	4,69E-05
A_51_P341746	NM_010823	Mpl	17480	0,710106518	9,446907407	0,001192703
A_51_P430900	NM_013642	Dusp1	19252	0,709298887	8,085148148	0,000420899
A_55_P2075195	NM_025422	Cd302	66205	0,707372019	6,731592593	9,15E-10
A_51_P488739	NM_030701	Hcar2	80885	0,707000795	6,76075	1,84E-13
A_55_P1957954	NM_009105	Rsu1	20163	0,705168521	9,101259259	0,000123106
A_51_P455807	NM_133838	Ehd4	98878	0,705023052	10,5294537	3,05E-07
A_55_P2015670	NM_008397	Itga6	16403	0,704330684	12,40418519	0,007170061
A_55_P2056973	NM_001033405	Treml2	328833	0,704006359	8,990962963	9,24E-05
A_52_P38639	NM_153795	Fermt3	108101	0,703262321	9,05762963	0,000315498
A_51_P111164	NM_172612	Rnd1	223881	0,702697933	7,253537037	6,00E-06
A_55_P2133248	AK134184	Gm8995	668139	0,702538156	8,452398148	5,90E-07
A_51_P374900	NM_028808	P2ry13	74191	0,702449921	6,596925926	7,71E-11
A_51_P191463	AY302216	H2-M2	14990	0,702097774	10,41486111	1,57E-07
A_51_P458866	NM_172647	F11r	16456	0,701539746	7,816861111	0,000139854
A_55_P1963868	NM_153119	Plekho2	102595	0,700100954	11,31878704	3,76E-08
A_52_P159050	BC059776	Cds2	110911	0,700052464	7,524518519	0,01492015
A_55_P1952166	NM_007880	Arid3a	13496	0,700036566	8,214537037	1,71E-09
A_55_P2085295	NM_198411	Inf2	70435	0,698254372	10,06146296	0,000347049
A_55_P2102126	NM_001017959	Lamp2	16784	0,695188394	8,590509259	2,99E-07
A_55_P1996862	NM_011190	Psme2	19188	0,695166932	11,75825926	3,88E-07
A_51_P464023	NM_021528	Chst12	59031	0,691531002	7,815944444	2,33E-05
A_51_P392687	NM_011701	Vim	22352	0,690555644	10,62230556	0,00043753
A_52_P483280	NM_010685	Lamp2	16784	0,688441971	7,947592593	3,02E-09
A_55_P2052769	NM_001042743	Mast2	17776	0,687534976	9,082824074	0,000898059
A_55_P2083121	NM_001164567	Vill	22351	0,68741256	7,458916667	0,003664353
A_55_P1983450	NM_011311	S100a4	20198	0,687197933	9,767916667	1,21E-05
A_51_P279606	NM_009896	Socs1	12703	0,686680445	7,461444444	2,57E-09
A_51_P387235	NM_021524	Nampt	59027	0,686426073	6,882240741	1,59E-12
A_51_P377452	NM_008677	Ncf4	17972	0,684333068	11,8549537	3,04E-05
A_51_P120717	NM_010721	Lmnb1	16906	0,683875199	7,258287037	3,73E-09

A_55_P2060966	NM_001163440	Mov10	17454	0,682843402	8,540138889	5,97E-08
A_51_P489522	NM_007797	Ctla2b	13025	0,682384738	14,83314815	6,56E-05
A_51_P414889	NM_027320	Ifi35	70110	0,682378378	7,926555556	2,44E-10
A_55_P1966659	NM_001025208	LOC547349	547349	0,681976153	8,173574074	1,05E-06
A_55_P2088425	NM_015749	Tcn2	21452	0,681416534	8,187425926	6,69E-07
A_66_P138137	NM_013632	Pnp	18950	0,681191574	8,288712963	1,93E-07
A_52_P476754	NM_176860	Ubash3b	72828	0,680378378	9,304685185	0,000180388
A_51_P173622	NM_011202	Ptpn11	19247	0,680176471	10,46587037	0,000407581
A_55_P1966194	NM_019549	Plek	56193	0,679790143	11,53537037	0,000357856
A_52_P282741	NM_011520	Sdc3	20970	0,679771065	6,544092593	5,52E-09
A_51_P460954	NM_009139	Ccl6	20305	0,678856121	11,12508333	0,000746876
A_51_P463846	NM_145545	Gbp7	229900	0,678447536	7,459509259	0,001267173
A_55_P2048176	XM_001475694	Gm3224	100041238	0,678444356	6,952712963	1,76E-05
A_55_P2037662	NM_144921	Atp1a3	232975	0,678401431	7,536712963	4,26E-08
A_55_P2064652	NM_001146007	Trim12c	319236	0,677742448	6,678055556	9,63E-10
A_55_P1967330	NR_003623	Gm5069	277333	0,677724165	9,873601852	1,21E-07
A_55_P2166985	NM_207677	Dedd2	67379	0,677420509	10,24730556	1,53E-08
A_52_P1197913	NM_008655	Gadd45b	17873	0,677096979	7,602907407	2,92E-10
A_55_P2114993	NM_009609	Actg1	11465	0,676769475	14,44538889	0,000528072
A_55_P2007768	NR_033633	Gm8709	667572	0,676185215	11,20519444	1,38E-06
A_51_P201480	NM_213659	Stat3	20848	0,675352941	9,488388889	7,43E-05
A_55_P1987264	NM_010312	Gnb2	14693	0,674875199	11,07212037	1,84E-05
A_55_P1965194	NM_178729	Fbxl5	242960	0,674786963	6,744148148	8,18E-09
A_55_P1989772	NM_021507	Sqrdl	59010	0,674662957	7,579592593	1,26E-10
A_55_P1995074	NM_176902	Ubald2	319370	0,673796502	11,53085185	7,80E-12
A_55_P2038007	NM_007791	Csrp1	13007	0,673124006	10,81168519	0,000951795
A_51_P146044	NM_144819	Ccdc92	215707	0,673015103	9,708287037	0,052684205
A_51_P451052	NM_175013	Pgm5	226041	0,672483307	6,775481481	5,66E-08
A_55_P2151986	NM_001038664	Gngt2	14710	0,670657393	9,464083333	2,00E-05
A_55_P2099650	NM_053195	Slc24a3	94249	0,669932432	9,381527778	0,0022381
A_55_P2051322	NM_025994	Efhd2	27984	0,669918124	8,214694444	1,07E-07
A_51_P252677	NM_026738	1110007C09Rik	68480	0,669735294	10,53015741	3,37E-06
A_55_P1981959	NM_001163470	Trafd1	231712	0,667267091	11,44040741	3,34E-10
A_51_P330428	NM_007918	Eif4ebp1	13685	0,667236089	10,03876852	2,18E-06
A_55_P2112737	NM_007393	Actb	11461	0,665301272	16,18647222	0,001663403
A_55_P2000548	NM_007687	Cfl1	12631	0,664399046	12,52064815	8,98E-06
A_51_P306527	NM_011738	Ywhah	22629	0,663267091	12,94266667	0,003090763
A_66_P113059	NM_010578	Itgb1	16412	0,662255167	8,41912037	1,52E-05
A_51_P147274	NM_153197	Clec4a3	73149	0,661699523	7,216092593	2,93E-05
A_55_P1955747	NR_002890	Gm12070	654472	0,661372814	11,52489815	2,33E-07
A_51_P222475	NM_053252	Ehbp1l1	114601	0,659647059	8,317333333	3,43E-14
A_55_P1961188	NR_023357	Gm6981	629557	0,658402226	9,767333333	5,53E-07
A_51_P312485	NM_013521	Fpr1	14293	0,657801272	8,386333333	0,000111148
A_55_P2163857	NM_001146007	Trim12c	319236	0,65749841	7,372361111	1,89E-07
A_55_P2124097	NR_023357	Gm6981	629557	0,656462639	10,60459259	5,62E-07
A_55_P1975714	NM_001160018	Tor1aip1	208263	0,655851351	8,01287963	4,74E-14
A_55_P2172470	NM_022029	Nrgn	64011	0,655387917	11,42794444	0,001322619
A_52_P127362	NM_029478	Vmp1	75909	0,65522973	9,248453704	0,001156244
A_52_P559919	NM_011163	Eif2ak2	19106	0,654197933	6,528305556	4,35E-16
A_55_P2149763	NM_001025313	Tapbp	21356	0,653983307	9,568805556	8,18E-09
A_66_P105689	NM_030684	Trim34a	94094	0,653703498	7,264731481	0,003742019

A_51_P427663	NM_007725	Cnn2	12798	0,653544515	10,90341667	0,000401774
A_51_P308590	NM_134084	Ppif	105675	0,653387122	7,10575	3,93E-06
A_55_P2102240	NM_181569	Nprl3	17168	0,651091415	8,230324074	4,05E-07
A_52_P374897	NM_009705	Arg2	11847	0,65036725	6,545092593	3,65E-10
A_55_P2107696	NM_205821	Mrgpra6	381886	0,649916534	6,522342593	6,52E-08
A_55_P2017759	NM_001033711	Evi2a	14017	0,649420509	7,86112037	1,92E-07
A_51_P131358	NM_009151	Selplg	20345	0,648932432	10,73363889	5,38E-05
A_51_P419246	NR_028427	5830416P10Rik	381232	0,648489666	8,18162963	0,000891573
A_55_P1989748	XM_001476309	Gm3051	100040936	0,648277424	6,977101852	1,86E-05
A_55_P2046852	NM_008089	Gata1	14460	0,648005564	9,688268519	6,04E-06
A_51_P131800	NM_007806	Cyba	13057	0,647864865	9,063907407	1,20E-08
A_55_P2095342	NM_001177302	Rara	19401	0,647717806	8,163953704	1,39E-06
A_51_P472249	NM_011405	Slc7a7	20540	0,647430048	7,558638889	1,21E-10
A_55_P1961690	NM_025329	Tctex1d2	66061	0,647257552	6,941814815	1,99E-06
A_55_P2007022	NM_011925	Cd97	26364	0,646765501	12,10471296	0,00097985
A_52_P99411	NM_008410	Itm2b	16432	0,646018283	11,63208333	2,19E-12
A_52_P215106	NM_001004435	Pik3r6	104709	0,645603339	7,751416667	1,53E-09
A_55_P2035509	NM_175026	Pyhin1	236312	0,645462639	8,288055556	0,000175199
A_52_P489202	AK169506	4732465J04Rik	414105	0,645324324	6,756666667	1,51E-08
A_51_P238722	NM_010740	Cd93	17064	0,644947536	8,298055556	0,000194669
A_55_P2181222	NM_001162938	Pydc3	100033459	0,644755962	7,531953704	0,016318923
A_55_P2104572	NM_144905	6330416G13Rik	230279	0,644356916	7,190768519	1,70E-06
A_55_P2059342	NM_010684	Lamp1	16783	0,643747218	11,8472037	1,82E-07
A_55_P1984284	NM_008084	Gapdh	14433	0,642964229	11,14699074	2,58E-06
A_52_P161495	NM_009744	Bcl6	12053	0,642605723	8,821333333	7,27E-06
A_55_P2176729	NM_007453	Prdx6	11758	0,642482512	10,89010185	0,000107364
A_55_P1952882	AF233647	Cyp4f18	72054	0,641027027	10,98222222	0,000334971
A_52_P56751	NM_008879	Lcp1	18826	0,640393482	10,01043519	0,000614439
A_52_P355084	NM_144797	Metrnl	210029	0,639885533	7,852851852	6,41E-07
A_51_P257550	NM_010807	Marcksl1	17357	0,639277424	7,062435185	5,09E-09
A_55_P2134790	NM_001024945	Qsox1	104009	0,639180445	9,475898148	4,28E-06
A_55_P2035613	NM_027154	Tmbim1	69660	0,638615262	7,629481481	0,005364669
A_52_P93933	NM_008562	Mcl1	17210	0,637007949	8,620203704	7,98E-05
A_51_P242201	NM_025972	Naaa	67111	0,636697138	7,513990741	8,87E-07
A_55_P2042183	XM_001476516	Gm3181	100041175	0,636492846	6,895203704	3,30E-05
A_55_P2052799	NM_009806	Cask	12361	0,635860095	6,842240741	8,81E-09
A_51_P111259	NM_026373	Cdk2ap2	52004	0,633669316	9,939592593	9,84E-05
A_51_P354077	NM_153153	Svil	225115	0,633504769	7,720009259	8,02E-07
A_55_P2005552	NM_172415	Arhgef10l	72754	0,631897456	7,420231481	2,44E-06
A_51_P279100	NM_008969	Ptgs1	19224	0,631895072	8,957759259	0,007170061
A_55_P2094925	NM_011157	Srgn	19073	0,631411765	12,97172222	1,08E-05
A_55_P2099961	NM_178186	Hist1h2ag	319167	0,631091415	12,70462037	1,55E-05
A_55_P2067362	AK171155	Dpep2	319446	0,628943561	6,568972222	5,25E-06
A_55_P2106690	NM_011527	Tal1	21349	0,628881558	8,784694444	0,000493075
A_51_P309158	NM_027840	Snx20	71607	0,62877027	10,35032407	1,30E-05
A_55_P2020378	NM_133838	Ehd4	98878	0,628290143	7,397583333	8,45E-09
A_52_P591153	NM_178911	Pld4	104759	0,628243243	7,923962963	1,33E-06
A_52_P179068	NM_017469	Gucy1b3	54195	0,626753577	7,648648148	0,000212318
A_52_P317393	NM_018882	Gpr56	14766	0,626327504	8,471944444	0,005284327
A_55_P2083411	NM_024444	Cyp4f18	72054	0,625950715	10,70783333	0,001325323
A_55_P2090880	NM_001145827	Stk40	74178	0,623603339	9,673342593	9,83E-08

A_55_P2068607	NM_029219	Rnf19b	75234	0,623445151	9,236851852	1,04E-06
A_51_P328652	NM_133348	Acot7	70025	0,623396661	9,069583333	2,82E-07
A_55_P2117741	NM_010879	Nck2	17974	0,622286963	9,471175926	0,000559997
A_52_P570266	NM_013640	Psmb10	19171	0,622215421	10,45750926	0,000366427
A_52_P12806	NR_003623	Gm5069	277333	0,621694754	10,84724074	4,57E-08
A_55_P2136832	NR_023357	Gm6981	629557	0,621627186	11,03919444	5,80E-07
A_51_P291460	NR_003630	Gm6498	624367	0,62136089	10,71912963	2,64E-07
A_52_P508089	NM_011179	Psap	19156	0,620753577	10,01703704	0,000208463
A_55_P1954021	NM_016741	Scarb1	20778	0,619329094	7,129296296	1,55E-07
A_55_P2007196	NM_001177307	Aldoa	11674	0,618527822	12,81962963	7,14E-07
A_55_P2139341	NM_001008427	Gm5595	434179	0,616878378	9,220175926	0,00057415
A_51_P234113	NM_172729	Nod1	107607	0,616868045	8,864962963	5,48E-08
A_51_P341918	NM_009366	Tsc22d1	21807	0,616036566	12,52748148	0,016370591
A_52_P616392	NM_183426	Sbno2	216161	0,615651033	6,858305556	5,21E-12
A_55_P2152009	NM_021344	Tesc	57816	0,615394277	9,99912963	0,019519464
A_55_P2028600	NM_022410	Myh9	17886	0,612947536	12,06612963	0,000408472
A_55_P2142830	NM_013610	Ninj1	18081	0,612779014	11,74842593	0,000511212
A_65_P19784	NM_080443	Asb7	117589	0,612639905	6,939712963	7,34E-07
A_51_P494675	NM_028071	Cotl1	72042	0,612398251	12,01169444	0,000421192
A_55_P2136501	NM_021565	Midn	59090	0,611624801	8,92987037	3,60E-06
A_51_P191469	NM_194346	Rnf31	268749	0,6114531	8,10537037	2,87E-09
A_66_P136788	NM_176902	Ubald2	319370	0,611203498	10,71011111	6,27E-10
A_55_P2030030	NM_007421	Adssl1	11565	0,61118601	7,980481481	0,000433243
A_55_P1992099	NM_008982	Ptprj	19271	0,611124006	8,732703704	0,000317477
A_55_P1987201	NM_009794	Capn2	12334	0,610240859	9,118657407	1,19E-06
A_51_P479786	NM_013870	Smtn	29856	0,610212242	6,857490741	5,81E-06
A_52_P370203	NM_021878	Jarid2	16468	0,60986407	8,271046296	0,000207336
A_51_P467751	NM_029884	Hgsnat	52120	0,609758347	7,766444444	8,96E-05
A_51_P357573	NM_145575	Cald1	109624	0,609306836	7,30687037	0,00148057
A_55_P1956160	NM_001081032	Gm8909	667977	0,607980922	7,244814815	0,054631964
A_51_P214747	NM_172893	Parp12	243771	0,60718601	6,493740741	5,79E-14
A_51_P227392	NM_133955	Rhou	69581	0,605856121	6,411064815	2,78E-12
A_51_P332201	NM_009976	Cst3	13010	0,605508744	11,99844444	0,000396507
A_52_P515247	NM_007486	Arhgdib	11857	0,605476153	12,99865741	0,00017093
A_51_P162437	NM_001081412	Bcr	110279	0,604387122	8,680175926	0,001437178
A_55_P1997275	NM_133216	Xpnpep1	170750	0,604377583	9,249287037	0,00042135
A_55_P2137203	NM_020579	B4galt3	57370	0,602494436	8,575055556	1,83E-05
A_51_P267239	NM_019980	Litaf	56722	0,602377583	11,43841667	0,001341602
A_51_P501453	NM_022430	Ms4a8a	64381	0,601143084	6,60012963	2,99E-06
A_55_P2090209	NM_022314	Tpm3	59069	0,601010334	12,52000926	0,000553691
A_55_P2169445	NM_133829	Mfsd6	98682	0,600989666	8,310583333	4,74E-10
A_55_P1958840	AK159653	Itga6	16403	0,599919714	7,93437963	0,000150191
A_55_P2043171	NM_011090	Lilra6	18726	0,599888712	6,779740741	1,15E-10
A_52_P132165	NM_053262	Hsd17b11	114664	0,59958903	8,018064815	5,46E-08
A_52_P195839	NM_009982	Ctsc	13032	0,598804452	8,140703704	0,0002938
A_51_P396854	NM_019502	Timm10b	14356	0,597185215	9,505564815	2,75E-05
A_51_P176352	NM_013864	Ndrg2	29811	0,597166932	7,586611111	0,000881161
A_55_P2073642	NM_001085385	1600014C10Rik	72244	0,596515103	6,39637037	2,25E-05
A_55_P1963144	NM_026849	Mtmr14	97287	0,596037361	8,113101852	2,85E-06
A_55_P2151273	NM_001039176	Elovl1	54325	0,59567806	9,43862037	1,20E-06
A_55_P2143219	NM_011242	Rasgrp2	19395	0,594918124	10,58030556	0,001048428

A_55_P1973854	NM_013662	Sema6b	20359	0,594775835	6,439796296	1,13E-07
A_51_P136355	NM_025331	Gng11	66066	0,594638315	14,14473148	0,008261841
A_51_P100327	NM_013683	Tap1	21354	0,594023052	6,887398148	1,63E-10
A_55_P2161865	NM_026131	Pdlim7	67399	0,593734499	7,124759259	1,98E-06
A_55_P2089804	NM_146065	Atf7	223922	0,593626391	7,779685185	8,60E-06
A_55_P2071176	NM_153511	Il1f9	215257	0,593290143	6,500601852	1,00E-07
A_51_P423290	NM_027613	Mmrn1	70945	0,592947536	9,287925926	0,005351185
A_51_P209280	NM_133685	Rab31	106572	0,592561208	6,707425926	5,05E-12
A_55_P2069306	NM_011206	Ptpn18	19253	0,59163593	12,4197037	0,000468879
A_55_P1957378	NM_011676	Unc119	22248	0,591430843	9,308092593	6,52E-08
A_55_P2077783	NR_003964	Tubb2a-ps2	627110	0,590869634	7,626296296	9,24E-05
A_55_P2081530	NM_080559	Sh3bgrl3	73723	0,58995628	13,82543519	0,000175701
A_55_P2014229	NM_013658	Sema4a	20351	0,589532591	10,1707037	5,79E-05
A_55_P2091486	NM_026827	Tmem219	68742	0,589438792	7,869462963	2,60E-08
A_52_P420466	NM_175660	Hist1h2ab	319172	0,58913434	8,086342593	0,000224514
A_51_P308961	NM_008254	Hmgcl	15356	0,587104134	8,436657407	1,29E-06
A_55_P1979330	NM_011932	Dapp1	26377	0,586855326	7,741722222	0,000317477
A_55_P2088385	NM_011618	Tnnt1	21955	0,585482512	8,304046296	0,004147538
A_52_P183181	NM_001038587	Adar	56417	0,584974563	7,421962963	4,51E-09
A_52_P676956	NM_054096	Tirap	117149	0,583033386	6,662074074	3,83E-09
A_55_P2072115	NR_030671	AW011738	100382	0,583030207	7,009277778	0,000342352
A_55_P2007470	NM_008808	Pdgfa	18590	0,582876789	9,293731481	0,013714316
A_65_P13209	NM_009983	Ctsd	13033	0,581430843	7,944740741	1,31E-05
A_55_P2135200	NM_181542	Slfn10-ps	237887	0,58072655	6,917111111	9,29E-08
A_55_P2139181	NM_145575	Cald1	109624	0,580478537	7,1415	0,002483852
A_55_P2018847	NM_001164735	Crlf2	57914	0,580152623	10,32212963	5,68E-05
A_66_P119518	NM_017379	Tuba8	53857	0,579490461	7,833675926	0,01323739
A_66_P110633	NM_001168660	Apol9b	71898	0,578890302	6,52	0,000301268
A_55_P1965725	NM_008084	Gapdh	14433	0,578735294	8,476805556	7,91E-08
A_55_P2091191	NM_172980	Slc28a2	269346	0,578350556	8,225953704	0,000412334
A_55_P2036627	NM_001162938	Pydc3	100033459	0,577033386	6,714962963	0,000582562
A_55_P2035038	XM_001473590	Gm2437	100039816	0,576512719	10,77069444	0,025220765
A_55_P2115136	NM_028732	4632428N05Rik	74048	0,576436407	8,745583333	6,34E-06
A_55_P2184364	NM_020579	B4galt3	57370	0,576333068	7,759953704	0,002928697
A_55_P2039878	NM_010327	Gp1bb	14724	0,576278219	6,676592593	9,70E-07
A_51_P185660	NM_011338	Ccl9	20308	0,57623132	8,714972222	0,006329618
A_55_P2148478	NM_029652	Klhl25	207952	0,575503975	8,809444444	8,93E-06
A_55_P2158990	NM_010591	Jun	16476	0,574547695	7,340083333	6,48E-05
A_55_P2162136	NM_016957	Hmgn2	15331	0,5740938	8,317037037	2,00E-07
A_51_P327295	NM_021473	Akr1a1	58810	0,573801272	9,529277778	2,96E-06
A_52_P59579	NM_026669	Tmbim6	110213	0,573536566	9,492361111	2,65E-06
A_55_P2034663	NM_007781	Csf2rb2	12984	0,572753577	6,868148148	1,23E-10
A_52_P671769	NM_176837	Arhgap18	73910	0,572538951	7,302648148	2,26E-05
A_55_P2023076	NM_023142	Arpc1b	11867	0,57218283	11,02796296	4,18E-05
A_51_P481159	NM_173047	Cbr3	109857	0,570787758	6,488712963	2,69E-07
A_55_P2052385	NM_194336	Gbp6	100702	0,570282989	6,722925926	5,57E-08
A_55_P2091691	NM_019837	Nudt3	56409	0,570074722	11,09701852	0,00963391
A_55_P2010152	NM_001164059	Sell	20343	0,569461844	10,68915741	0,001167255
A_55_P2041584	XM_001003844	F420015M19Rik	619329	0,56940938	8,729768519	0,000519528
A_55_P2000409	NM_001002786	Rab44	442827	0,568875994	7,850814815	0,000675838
A_55_P2004562	NM_001170851	Klra2	16633	0,568720191	6,637796296	0,012669294

A_55_P2016034	NM_001033207	Nlrc5	434341	0,568657393	8,375194444	0,000112183
A_51_P323180	NM_172777	Gbp9	236573	0,568436407	7,656601852	0,000800126
A_55_P2034928	NM_001037925	BC147527	625360	0,568415739	8,488175926	0,0026868
A_55_P1953459	NM_001171147	Yap1	22601	0,568265501	8,072425926	0,005896461
A_51_P198645	NM_133167	Parvb	170736	0,568194754	7,921083333	0,005306955
A_51_P465281	NM_008495	Lgals1	16852	0,567460254	10,32913889	7,61E-05
A_55_P2061170	NM_198664	Tbc1d2	381605	0,567430048	6,612824074	4,75E-07
A_51_P345367	NM_010724	Psmb8	16913	0,567050079	9,702416667	9,26E-05
A_55_P2137121	NM_001013817	Sp140	434484	0,566769475	7,267518519	3,57E-06
A_55_P2064043	NM_009851	Cd44	12505	0,566651033	8,328231481	2,83E-06
A_55_P1966719	XM_001478568	Gm3806	100042368	0,566311606	8,587851852	0,000523779
A_51_P297679	NM_008225	Hcls1	15163	0,566287758	11,24012963	9,07E-05
A_55_P2025611	NM_011190	Psme2	19188	0,56472337	9,746851852	3,81E-07
A_51_P356283	NM_133765	Fbxo31	76454	0,564350556	7,914861111	5,00E-05
A_66_P104815	NM_007899	Ecm1	13601	0,563922893	7,854509259	0,001136536
A_55_P1960960	NM_172465	Zdhhc9	208884	0,563786963	8,284092593	1,68E-06
A_55_P1994258	NM_008084	Gapdh	14433	0,563704293	8,384388889	5,52E-07
A_55_P2125588	NM_008808	Pdgfa	18590	0,563411765	8,87212963	0,012095691
A_52_P116134	NM_009656	Aldh2	11669	0,56240461	10,23852778	0,045898641
A_51_P487360	NM_016677	Hpcal1	53602	0,560825914	8,708416667	0,000147355
A_55_P2176731	NM_007453	Prdx6	11758	0,560297297	10,33840741	0,000989618
A_55_P2039038	NM_001081750	Zfp664	269704	0,560158983	8,292018519	0,014650064
A_55_P1960216	NM_009842	Cd151	12476	0,559999205	8,02787963	0,005406054
A_51_P423091	NM_008027	Flot1	14251	0,559683625	8,323222222	1,38E-06
A_52_P233441	NM_008090	Gata2	14461	0,559532591	7,210648148	0,000119513
A_52_P518808	BC027020	Mmd	67468	0,559304452	8,388638889	0,028253126
A_55_P2042823	NM_001145859	Sh3bp2	24055	0,558265501	8,209074074	2,26E-05
A_51_P153053	NM_020561	Smpdl3a	57319	0,556477742	9,800231481	0,000347883
A_55_P2119463	NM_010281	Ggh	14590	0,556271065	8,060472222	0,00045426
A_55_P1976212	NM_009151	Selplg	20345	0,55599841	9,165814815	2,12E-06
A_55_P1959703	BC098458	Zfp646	233905	0,555878378	11,88937963	1,13E-05
A_52_P253915	NM_138651	Cds2	110911	0,555058029	7,374935185	4,03E-09
A_52_P590396	NM_019972	Sort1	20661	0,554605723	6,741037037	9,86E-08
A_55_P1970763	NM_001024932	Pilrb2	545812	0,554024642	6,661138889	2,67E-07
A_55_P2179030	NM_017375	Ostf1	20409	0,553557234	9,541583333	4,35E-07
A_55_P2171463	NM_009145	Nptn	20320	0,553510334	11,59846296	0,01295726
A_55_P1987709	AK034145	Ptprd	19266	0,552261526	6,466342593	2,88E-05
A_55_P2060303	NM_008624	Mras	17532	0,551333068	7,534324074	0,002707688
A_51_P417839	NM_207225	Hdac4	208727	0,550934022	6,886638889	8,01E-06
A_55_P2108690	NR_033633	Gm8709	667572	0,550265501	8,472092593	8,87E-07
A_55_P1977583	NM_013469	Anxa11	11744	0,550012719	8,883537037	0,000951997
A_55_P2241299	NM_001025613	Otud7b	229603	0,54995469	7,24587963	0,000167408
A_51_P483617	NM_029554	0610040J01Rik	76261	0,549942766	6,296018519	9,29E-08
A_55_P2107347	NM_013835	Trove2	20822	0,549322734	7,488740741	8,19E-05
A_55_P2141395	NM_023463	Ly6g6c	68468	0,548799682	10,2525	0,010118654
A_55_P2359797	NM_145857	Nod2	257632	0,5486407	6,546685185	1,22E-09
A_55_P2008907	NM_009609	Actg1	11465	0,54845151	13,50805556	0,003459158
A_55_P2027102	NM_134257	Rgs3	50780	0,547888712	9,168611111	4,02E-05
A_55_P2098558	NM_013901	Slc39a1	30791	0,547662162	8,40875	1,80E-07
A_55_P2069850	NM_011123	Plp1	18823	0,547613672	8,760481481	0,023399487
A_55_P2065824	NM_001159284	Smtn	29856	0,547153418	8,808842593	0,000520587

A_55_P2051099	NM_011609	Tnfrsf1a	21937	0,547072337	8,569287037	2,39E-08
A_55_P2011490	XM_001473460	Gm16510	100039766	0,546188394	7,759916667	6,91E-08
A_55_P2072706	NM_001081163	Chsy1	269941	0,545944356	8,097925926	6,36E-06
A_55_P2182358	NM_007880	Arid3a	13496	0,545782989	7,017638889	1,47E-08
A_51_P357341	NM_033444	Clic1	114584	0,545500795	11,42609259	2,00E-05
A_51_P433824	NM_009415	Tpi1	21991	0,543504769	9,855842593	0,000294935
A_55_P2014319	NM_001007578	Armcx6	278097	0,543062798	7,052157407	2,16E-05
A_55_P2103115	NM_172843	Tor1aip2	240832	0,54282035	7,626814815	3,40E-08
A_65_P12392	NM_011594	Timp2	21858	0,540882353	7,313722222	1,66E-05
A_55_P2033420	NM_023220	Sppl2a	66552	0,540763911	7,662935185	3,13E-07
A_55_P1967533	NR_003623	Gm5069	277333	0,540633545	8,733787037	1,61E-06
A_51_P459350	NM_019771	Dstn	56431	0,54045628	8,376518519	0,002626582
A_51_P155142	NM_026560	Cdca8	52276	0,540281399	7,036722222	2,04E-09
A_55_P2074291	NM_015797	Fbxo6	50762	0,540174881	8,558296296	6,99E-07
A_51_P148355	NM_175433	Zfp710	209225	0,53963275	8,594777778	0,000117251
A_55_P2003991	NM_008207	H2-T24	15042	0,539071542	7,127981481	4,15E-10
A_55_P1979708	NM_023305	Ubap1	67123	0,538197933	9,075824074	0,003234261
A_55_P1960237	NM_172659	Slc2a6	227659	0,537294913	6,482175926	9,20E-12
A_51_P371051	NM_028608	Glipr1	73690	0,536761526	8,132222222	8,17E-06
A_66_P123635	NM_007780	Csf2rb	12983	0,536513514	7,196111111	4,10E-09
A_55_P2030638	NM_026725	Dusp23	68440	0,535897456	7,128453704	0,005311018
A_55_P2068892	NM_010559	Il6ra	16194	0,535693164	10,31016667	0,00310577
A_55_P1959305	NM_178446	Rbm47	245945	0,535573132	6,663731481	2,65E-07
A_55_P2082319	NM_001024721	BC094916	545384	0,535561208	7,76187037	0,007237909
A_51_P494361	NM_175341	Mbnl2	105559	0,535182035	8,717787037	8,25E-05
A_55_P2135986	NM_029499	Ms4a4c	64380	0,534199523	9,663231481	0,013261634
A_55_P2001233	NM_001162938	Pydc3	100033459	0,533943561	8,71787963	0,024387837
A_51_P392705	NM_009531	Xpc	22591	0,533037361	7,845435185	5,63E-06
A_52_P329917	NM_022999	Prrg2	65116	0,531169316	7,190861111	1,06E-05
A_55_P2116924	NM_001037999	Dbi	13167	0,529570747	9,234648148	1,68E-05
A_55_P2107207	AK009736	Gpr137b-ps	664862	0,529243243	7,61862963	6,01E-07
A_55_P2106666	NM_009624	Adcy9	11515	0,528652623	7,185842593	0,001056444
A_66_P117933	NM_001005858	I830012O16Rik	667370	0,528476153	6,184398148	1,69E-09
A_66_P119174	NM_026036	Cmtm6	67213	0,52763752	9,873	0,000111734
A_55_P2099378	NM_172472	Tfe3	209446	0,526627186	8,377601852	6,25E-11
A_51_P390715	NM_011577	Tgfb1	21803	0,526486486	7,828740741	0,000208144
A_55_P1965772	NM_001163337	Atp2a3	53313	0,525864865	9,958814815	0,001394893
A_66_P124549	NM_001159551	H13	14950	0,523899841	9,615203704	0,000176448
A_51_P350817	NM_009922	Cnn1	12797	0,523484102	6,673712963	0,01501257
A_55_P2130975	NM_010881	Ncoa1	17977	0,523430843	8,1285	0,000241468
A_52_P393306	NM_009222	Snap23	20619	0,523114467	8,369518519	0,00083244
A_55_P2017944	NM_026695	Etfb	110826	0,523109698	9,463074074	3,17E-05
A_52_P550173	NM_013730	Slamf1	27218	0,52259062	8,208953704	0,009383272
A_55_P1998957	NM_033541	Oas1c	114643	0,521787758	7,273138889	0,000327033
A_51_P127681	NM_013885	Clic4	29876	0,52167806	9,962916667	0,038086832
A_55_P2143233	NM_011242	Rasgrp2	19395	0,521677266	10,20209259	0,002083081
A_51_P435339	NM_010149	Epor	13857	0,521304452	7,149101852	1,33E-06
A_51_P238786	NM_008562	Mcl1	17210	0,521177266	11,44275	0,000974178
A_55_P2063251	NM_028207	Dusp3	72349	0,520821145	7,589064815	0,005535374
A_55_P2074942	NM_010484	Slc6a4	15567	0,52054531	8,301111111	0,013155525
A_55_P2086810	NM_001013817	Sp140	434484	0,520146264	6,868425926	1,24E-08

A_52_P191975	NM_001003934	Rtn3	20168	0,519098569	7,720796296	4,16E-05
A_55_P1994927	NM_144538	Rab3il1	74760	0,518628776	9,215768519	0,049701688
A_51_P308681	NM_001081059	Mcur1	76137	0,518620827	7,367527778	2,91E-07
A_55_P2071329	NM_019566	Rhog	56212	0,518357711	11,73825926	1,14E-06
A_51_P227718	NM_145149	Rasgrp4	233046	0,518279014	6,534675926	1,73E-08
A_55_P2129291	NM_146114	Dclre1c	227525	0,518111288	6,952296296	3,17E-05
A_51_P342669	NM_023418	Pgam1	18648	0,517946741	9,157212963	0,000119368
A_51_P509573	NM_013652	Ccl4	20303	0,517934817	6,682351852	6,39E-07
A_55_P2064507	NM_026322	Msra	110265	0,517645469	7,634888889	0,012848279
A_51_P350073	NM_023668	Ndel1	83431	0,517342607	8,375416667	5,81E-06
A_51_P154973	NM_021547	Stard3	59045	0,516085056	8,154731481	3,30E-05
A_55_P1960416	NM_146069	Nrros	224109	0,515130366	9,995518519	0,002243703
A_55_P2103837	NM_008620	Gbp4	17472	0,515094595	7,996435185	8,05E-05
A_55_P2013948	NM_146120	Gsn	227753	0,514602544	10,84983333	0,006344857
A_52_P359965	NM_007754	Cpd	12874	0,514337043	6,522092593	8,56E-06
A_51_P403334	NM_024192	Cuedc2	67116	0,514275835	9,044638889	2,12E-05
A_55_P2074206	NM_146128	Dlgap4	228836	0,514180445	9,12725	2,63E-05
A_52_P563617	NM_133772	Ssbp4	76900	0,513921304	8,691601852	3,34E-06
A_55_P2061737	NM_021278	Tmsb4x	19241	0,513563593	16,31826852	0,001880406
A_55_P2062851	NM_020575	March7	57438	0,513102544	8,875805556	0,000565862
A_55_P2080603	NM_031165	Hspa8	15481	0,513038951	13,41937963	0,019217955
A_52_P498086	NM_011355	Spi1	20375	0,512751987	6,518388889	9,78E-11
A_55_P2184601	NM_008400	Itgal	16408	0,512426868	7,820342593	0,000333282
A_66_P126138	NM_010881	Ncoa1	17977	0,511235294	7,293055556	1,50E-05
A_55_P1953301	NM_146126	Sord	20322	0,511066773	8,203851852	0,072720766
A_52_P522427	NM_197944	Hsh2d	209488	0,510577901	6,769546296	1,26E-09
A_55_P2024841	NM_008185	Gstt1	14871	0,510135135	7,105537037	1,93E-05
A_55_P2066384	NM_009652	Akt1	11651	0,509993641	9,380851852	0,000487393
A_55_P1970788	NM_176912	C5ar2	319430	0,509800477	6,424601852	0,000996563
A_55_P1993789	NM_016957	Hmgn2	15331	0,509599364	9,055611111	0,000241468
A_55_P2086500	NM_021494	Dennd5a	19347	0,509428458	9,487935185	2,19E-07
A_55_P2059904	NM_001081163	Chsy1	269941	0,508788553	7,796555556	8,76E-06
A_55_P1955841	NM_008906	Ctsa	19025	0,508623211	10,78064815	0,005398597
A_55_P1967266	NM_138749	Plxnb2	140570	0,508472973	7,975898148	0,000263144
A_52_P578790	NM_013739	Dok3	27261	0,508212242	8,499101852	0,000183453
A_55_P2158478	NM_028024	Nkiras2	71966	0,507958665	8,143222222	7,91E-08
A_66_P113487	NM_011862	Pacsin2	23970	0,507732909	10,22968519	0,002985087
A_51_P263965	NM_010442	Hmox1	15368	0,507495231	7,333287037	4,28E-09
A_51_P295192	NM_010907	Nfkbia	18035	0,50667965	10,42178704	0,004806087
A_55_P2117525	NM_001166410	Rbm3	19652	0,50641256	9,456398148	0,000861671
A_55_P2000127	NM_009365	Tgfb1i1	21804	0,506201908	7,973018519	0,006907127
A_51_P172231	NM_026960	Gsdmd	69146	0,50613752	7,919268519	3,87E-08
A_51_P146303	NM_028617	Mvb12a	73711	0,505866455	7,879759259	6,90E-07
A_52_P513177	NM_001029841	Sla	20491	0,505747218	8,923148148	0,00206063
A_51_P217336	NM_029153	Scamp1	107767	0,505565183	7,997935185	0,018725611
A_55_P2033445	NM_011610	Tnfrsf1b	21938	0,505337043	6,874425926	6,12E-06
A_55_P1990548	NM_145476	Tbc1d22a	223754	0,505104928	8,897296296	0,000375458
A_52_P429774	NM_146105	Cnst	226744	0,505038951	8,049898148	0,00023345
A_51_P382849	NM_010330	Emb	13723	0,505007949	8,506759259	0,004964674
A_52_P498208	NM_178183	Hist1h2ak	319169	0,504312401	8,741694444	5,09E-05
A_55_P2088995	NM_011636	Plscr1	22038	0,504026232	6,777027778	5,34E-09

A_55_P2144850	NM_011186	Psmb5	19173	0,504022258	11,48990741	1,18E-05
A_55_P2000224	NM_001042580	Cd63	12512	0,504010334	8,546046296	8,19E-05
A_55_P1973033	NM_019919	Ltbp1	268977	0,501868839	7,646490741	0,004087291
A_55_P2064557	NM_011046	Furin	18550	0,501685215	8,37212963	2,95E-05
A_55_P1988202	NM_001045481	Ifi203	15950	0,501218601	8,755898148	0,076954084
A_51_P510849	NM_030018	Tmem50b	77975	0,500843402	8,594935185	2,36E-06
A_51_P440047	NM_173752	Lgalsl	216551	0,500704293	8,528222222	0,07059954
A_52_P190973	NM_009502	Vcl	22330	0,500658188	7,454425926	0,000228018
A_52_P456134	NM_010046	Dgat1	13350	0,500160572	6,949555556	2,40E-06
A_55_P1971076	NM_015804	Atp11a	50770	0,500151828	6,461490741	8,98E-07
A_51_P503303	NM_021550	C1galt1c1	59048	0,50008744	6,985740741	3,83E-06
A_66_P108152	NM_007620	Cbr1	12408	-0,50063752	9,5085	1,35E-05
A_52_P658034	NM_009433	Tspyl1	22110	-0,500812401	10,5812037	0,001246843
A_55_P2405244	AV207074	1700061N14Rik	73432	-0,500880763	7,946333333	0,012460523
A_55_P2088690	XM_001478162	Gm3652	100042078	-0,500950715	7,932314815	0,017242737
A_66_P111089	NM_026454	Ube2f	67921	-0,501470588	8,46137037	0,04954512
A_55_P1995607	NM_207551	Olfr209	404311	-0,501981717	8,671990741	0,032817934
A_55_P2042086	NM_008738	Nrtn	18188	-0,502399046	11,10085185	0,013935694
A_52_P230688	NM_025620	Rep15	66532	-0,502550874	7,178453704	0,000351883
A_55_P2259500	AK083809	D130012P04Rik	319377	-0,502630366	8,600546296	0,01053706
A_55_P2048550	NM_001127686	Hbb-bh2	436003	-0,502674086	6,994722222	0,044564652
A_55_P1984118	NM_020014	Gfra4	14588	-0,503096979	13,78931481	0,032817934
A_51_P159612	NM_019487	Hebp2	56016	-0,503933227	8,501888889	0,026914082
A_66_P102719	NM_025287	Spop	20747	-0,504647854	10,5330463	0,000174432
A_51_P307325	NM_009030	Rbbp4	19646	-0,505424483	10,03292593	0,007875388
A_51_P516148	NM_016984	Trpc4	22066	-0,505846582	8,521842593	0,063117413
A_55_P2422318	AK014953	4921523P09Rik	70926	-0,505897456	7,079101852	0,000565841
A_51_P481768	NM_018756	Tcstv1	54382	-0,506058029	7,001638889	0,001612931
A_55_P2000613	NM_178703	Slc6a1	232333	-0,50622973	8,656138889	0,011440199
A_55_P1966169	BC091753	Ppdpf	66496	-0,506431638	10,59382407	0,000401136
A_51_P412914	NM_010112	Efs	13644	-0,50654213	7,413277778	0,001715561
A_55_P2136763	XM_886587	Gm6293	622178	-0,508891892	9,784814815	0,002200084
A_55_P2168663	NM_007507	Atp5k	11958	-0,508971383	11,11140741	0,000176155
A_55_P2019784	NM_028880	Lrrtm1	74342	-0,509034181	8,402305556	0,011843864
A_55_P1996365	NM_206975	Ifna14	404549	-0,509460254	7,831601852	0,010236283
A_55_P1984640	NM_026454	Ube2f	67921	-0,510559618	9,785907407	0,03911542
A_55_P2002578	NM_010145	Ephx1	13849	-0,510569157	8,963518519	0,008111721
A_51_P346453	NM_153570	Noc4l	100608	-0,512120032	9,99475	0,018194783
A_55_P2102155	NM_001177795	Rgs20	58175	-0,512737679	11,27644444	0,044009794
A_55_P2041634	NM_001081041	Vps51	68505	-0,51281399	11,68883333	0,067145066
A_55_P2171773	AK076976	LOC102636217	102636217	-0,513386328	8,417	0,01330405
A_55_P2149615	NM_009750	Ngfrap1	12070	-0,514350556	11,55289815	0,019486788
A_55_P2097340	NM_145621	Camkv	235604	-0,514515103	7,106314815	0,000204804
A_51_P136888	NM_009029	Rb1	19645	-0,514599364	9,978462963	0,011498059
A_52_P650855	NM_177390	Myo1d	338367	-0,515364865	8,70737963	0,054465974
A_55_P2083184	NR_026831	Gm10272	16697	-0,515754372	8,883083333	0,004572907
A_55_P1960664	XM_001475067	Gm3035	100040908	-0,516077107	10,92350926	0,01756973
A_55_P2106844	XM_001476912	Gm7475	665070	-0,516159777	9,469305556	0,034149744
A_55_P1989149	NM_147007	Olfr394	259009	-0,516183625	9,102083333	0,036574024
A_55_P2152547	NM_010859	Myl3	17897	-0,516597774	9,244740741	0,016736731
A_55_P2007991	NM_009449	Tuba3b	22147	-0,517183625	9,159898148	0,0113863

A_55_P1961458	NR_001586	Speer1-ps1	70896	-0,517600159	12,95308333	0,0659369
A_55_P1999108	NM_010031	Defa1	13216	-0,517810016	10,35452778	0,024186873
A_66_P112593	NM_024171	Sec61b	66212	-0,517922893	11,93300926	0,059097451
A_55_P2008258	NM_001040399	Larp1b	214048	-0,517935612	8,362824074	0,005535374
A_55_P2105220	NM_153571	Hscb	100900	-0,518086645	12,28975	0,061222345
A_55_P1985911	NM_010919	Nkx2-2	18088	-0,518149444	8,336648148	0,005318003
A_55_P2089198	NM_011880	Rgs7	24012	-0,518401431	10,60941667	0,030995856
A_55_P2062926	NM_001033347	D430041D05Rik	241589	-0,518876789	11,15462037	0,013333039
A_55_P2008021	NM_026838	Srpx2	68792	-0,519580286	9,944314815	0,025103246
A_52_P237652	NR_015556	2610035D17Rik	72386	-0,519639905	7,479509259	0,050852152
A_55_P1961152	NM_011141	Pou3f1	18991	-0,519666932	9,065990741	0,005541575
A_66_P125634	NM_172864	Wdr63	242253	-0,519906995	10,41312037	0,008167814
A_66_P139586	NM_001033404	Gm813	328695	-0,520383943	8,312675926	0,03385873
A_55_P2040962	NM_001033442	Gm1604b	381059	-0,520553259	10,48164815	0,003104604
A_55_P2129776	NM_030890	Prrt1	260297	-0,521035771	8,515935185	0,007141074
A_52_P483959	NM_207233	C1ql2	226359	-0,521573132	9,628861111	0,040585911
A_55_P2101074	XM_001472853	Gm3848	100042441	-0,521846582	8,53387963	0,011219685
A_55_P1970920	NM_011591	Timm17b	21855	-0,522114467	10,49316667	0,000189691
A_55_P2037712	NM_133786	Smc4	70099	-0,522153418	8,451787037	4,34E-06
A_55_P2096485	NM_025784	Bcs1l	66821	-0,522192369	9,751907407	0,001351427
A_51_P311362	AK015429	4930449E01Rik	74864	-0,523445946	8,077842593	0,007532339
A_55_P2144781	NM_173421	Erich5	239368	-0,523463434	9,602212963	0,035620182
A_55_P1963639	NM_026716	Sycn	68416	-0,523631955	9,225509259	0,031045272
A_52_P475870	NM_001164686	Tmem29	382245	-0,523655008	7,601111111	0,001299702
A_55_P2023937	NM_022016	Impg1	63859	-0,524084261	10,28211111	0,040359559
A_66_P110490	NM_030229	Polr3h	78929	-0,524474563	7,156490741	0,000189691
A_55_P2037235	NM_175397	Sp110	109032	-0,524672496	9,110296296	0,00385399
A_51_P266248	AK134885	Ighv14-2	668421	-0,524781399	10,20339815	0,002405638
A_55_P2030938	NM_025863	Trim59	66949	-0,525135135	8,752685185	0,017363642
A_55_P1960496	NM_025800	Ppp1r2	66849	-0,52513752	9,301212963	0,00423013
A_66_P130449	NM_207270	Ptprh	545902	-0,525191574	7,812175926	0,017147599
A_51_P359485	NM_024231	Zfpl1	81909	-0,525248013	10,18866667	0,007427134
A_55_P2200628	AK014629	4733401D01Rik	70847	-0,52536725	10,58355556	0,025716125
A_52_P638283	NM_207258	Fam71f1	330277	-0,525460254	7,163138889	0,000934895
A_55_P2096768	NM_009027	Rasgrf2	19418	-0,525955485	7,998740741	0,004724707
A_55_P1970274	NM_001033425	Zscan10	332221	-0,526473768	8,541944444	0,012591497
A_55_P2051039	NM_001099346	Gm11937	100041488	-0,526546105	11,0864537	0,009070448
A_55_P2038152	NM_183173	Sowaha	237761	-0,526983307	8,312712963	0,032407207
A_51_P159711	NM_053070	Car7	12354	-0,527248013	11,4307963	0,013700943
A_55_P2362601	AK078562	D4Ertd681e	52421	-0,527648649	9,265962963	0,009187017
A_55_P2177721	NM_007416	Adra1b	11548	-0,528220986	10,80462963	0,050959399
A_55_P2207335	AK042215	Nron	320482	-0,529193959	12,86596296	0,024209876
A_55_P1982818	NM_013614	Odc1	18263	-0,529507949	9,488953704	0,000376599
A_55_P1957850	NM_025664	Snx9	66616	-0,52963275	7,824574074	0,0154609
A_55_P2066219	XM_001473544	Gm3455	100041653	-0,529893482	7,794777778	0,007427134
A_66_P100932	NM_026982	Tmem256	69186	-0,530657393	13,91134259	0,037016124
A_51_P299632	NM_027641	Spef1	70997	-0,530693959	8,884935185	0,003176709
A_51_P133638	NR_030776	3110021A11Rik	67289	-0,531258347	7,930842593	0,003042339
A_55_P2087013	NM_010432	Hipk1	15257	-0,531341812	8,165814815	0,014206579
A_55_P1955542	NM_146952	Olfr522	258954	-0,531562003	9,025546296	0,010384693
A_51_P133097	NR_033452	Gm4013	100042757	-0,531692369	8,638898148	0,006971495

A_52_P69109	NM_011387	Slc10a1	20493	-0,531873609	8,269009259	0,036099654
A_66_P122729	NM_025594	Zmat2	66492	-0,532011129	8,644944444	0,00201568
A_55_P2101585	NM_010016	Cd55	13136	-0,532230525	7,454	0,000429605
A_55_P2168316	NM_001177438	Aldh3b2	621603	-0,532420509	6,588046296	0,020362125
A_55_P2062444	NM_133771	Memo1	76890	-0,533394277	11,14605556	0,025006508
A_55_P2037111	NM_009391	Ran	19384	-0,533609698	7,074731481	1,25E-06
A_65_P12359	NM_001033768	Pin1rt1	241593	-0,533653418	10,25990741	0,005579146
A_51_P160413	NM_178691	Yod1	226418	-0,533972178	7,531953704	0,00364044
A_66_P118316	AK006709	1700047G07Rik	73323	-0,534427663	8,213759259	0,002942116
A_51_P521052	NM_029627	Ly6k	76486	-0,534853736	8,894055556	0,003952076
A_55_P2122666	NM_153169	Pnma3	245468	-0,535236884	10,59535185	0,01538586
A_55_P1995173	NM_013614	Odc1	18263	-0,53545628	11,83790741	0,000142795
A_55_P2000658	NR_033578	Gm15645	626055	-0,53568601	8,94975	0,000214591
A_52_P412452	NM_017383	Cntn6	53870	-0,536062798	11,07763889	0,056380301
A_52_P390944	NM_016803	Chst3	53374	-0,536096184	7,860527778	0,000116894
A_52_P457529	NM_177069	Fbxw21	320082	-0,536157393	11,40557407	0,020915898
A_55_P2148655	NM_198410	Paqr6	68957	-0,536289348	8,728777778	0,00722329
A_55_P2182700	NM_031869	Prkab1	19079	-0,536910175	9,443175926	0,006347139
A_55_P2080163	NM_025821	Carhsp1	52502	-0,536995231	15,0215	0,001815965
A_55_P2088725	NM_021522	Usp14	59025	-0,53709539	8,796666667	0,021513429
A_55_P2152901	AK013627	2900040C04Rik	72893	-0,537879968	10,72530556	0,034448184
A_55_P2279997	AK016075	4930549C15Rik	75243	-0,538054054	10,73083333	0,019786514
A_55_P2184931	NM_028762	Rbm19	74111	-0,538522258	8,513453704	0,010600933
A_55_P2155620	NM_025282	Mef2c	17260	-0,538874404	9,667888889	0,037565457
A_55_P1989996	NM_013688	Tcte1	21645	-0,539497615	8,897314815	0,006066438
A_66_P101835	NM_007646	Cd38	12494	-0,541418124	8,302851852	0,000238607
A_52_P91402	NM_153160	Zcchc17	619605	-0,541482512	9,498101852	3,49E-09
A_55_P2035495	NM_019778	Zbtb20	56490	-0,541957075	9,15112963	0,000347883
A_55_P2153904	XM_884529	Gm6132	620143	-0,542551669	12,33322222	0,002423921
A_55_P2089955	NM_001025086	Rhox7	547168	-0,543025437	11,50607407	0,015088776
A_55_P2159565	NM_207030	Tas2r131	387356	-0,543081081	7,885388889	0,006720943
A_55_P2061492	AK139523	Pnldc1	240023	-0,543786169	9,808435185	0,00419657
A_52_P589391	NM_010069	Doc2a	13446	-0,54386407	8,391175926	0,009324676
A_55_P2172299	BC008275	Timm17b	21855	-0,544389507	10,69146296	0,000160811
A_52_P473106	NM_153114	Otos	260301	-0,544761526	7,725296296	0,000839833
A_55_P2066927	AK088379	Galt	14430	-0,544845787	9,148444444	0,006604927
A_66_P109562	XM_890719	Gm12271	626150	-0,545310016	10,93118519	0,002499312
A_55_P2140107	NM_009995	Cyp21a1	13079	-0,546071542	8,899722222	0,008095618
A_55_P2008407	NM_133485	Ppp1r14c	76142	-0,546076312	11,88877778	0,022827576
A_55_P2073705	NM_178667	Tfdp2	211586	-0,54681717	7,852240741	0,022122984
A_51_P450527	NM_011526	Tagln	21345	-0,547277424	11,73410185	0,00924514
A_55_P2006018	NM_009207	Slc4a2	20535	-0,547396661	9,797990741	0,000279194
A_55_P2032192	NM_001164249	Tpm1	22003	-0,547903021	10,08955556	0,010123427
A_55_P1966070	NM_199063	Ssx9	382206	-0,548151033	9,461944444	0,025342173
A_55_P2085370	NM_001172114	Traf7	224619	-0,548613672	8,245833333	0,004336721
A_51_P148684	NM_175006	Pou6f2	218030	-0,549671701	9,033490741	0,024026127
A_55_P2009345	NM_172908	Ovch2	244199	-0,54968601	10,99415741	0,018124622
A_55_P2112085	NM_027906	Vwa8	219189	-0,5498593	9,918009259	0,074813606
A_55_P2054913	NM_001011863	Olfr406	258181	-0,550413355	8,88612963	0,094691563
A_51_P343309	NM_009908	Cmas	12764	-0,550599364	10,9852963	0,04428996
A_51_P273005	NM_009611	Actl7a	11470	-0,551505564	7,265851852	0,003477286

A_52_P309337	NM_019648	Metap2	56307	-0,55181717	7,395314815	0,006378983
A_52_P136751	NM_007886	Dtnb	13528	-0,552123211	8,035046296	0,01619767
A_55_P1980677	NM_027037	1700007K09Rik	69318	-0,552147059	12,74182407	0,026643936
A_66_P120612	NR_002873	Vax2os	574519	-0,552967409	8,71237963	0,007383344
A_55_P1968774	NM_001162365	Ptk2b	19229	-0,553254372	9,316388889	0,003234261
A_52_P640194	NM_001033531	Klhl32	212390	-0,553328299	8,99275	0,00687919
A_55_P2015405	NM_001114679	9930111J21Rik1	667214	-0,553562798	9,889148148	0,012494139
A_55_P2117959	NM_007823	Cyp4b1	13120	-0,553630366	7,932601852	0,029734741
A_55_P2013559	NM_010075	Dpp6	13483	-0,553687599	8,631768519	0,004609462
A_51_P439612	NM_020266	Dnajb2	56812	-0,553878378	10,06549074	0,024439103
A_55_P1970067	NM_001129787	Igsf9b	235086	-0,554281399	9,003185185	0,004587747
A_55_P2147736	NM_011993	Dpysl4	26757	-0,554458665	9,102416667	0,003312561
A_66_P108247	NM_009464	Ucp3	22229	-0,554611288	13,23519444	0,005237385
A_66_P106536	NM_027360	2010107E04Rik	70257	-0,554891892	11,8737963	7,04E-05
A_55_P2169046	NM_146444	Olfr458	258436	-0,555011924	9,200861111	0,009646801
A_55_P2022509	NM_172948	Mgat5b	268510	-0,555381558	9,739166667	0,030642685
A_55_P2026982	NM_173744	Tdrp	72148	-0,555852146	7,723814815	1,29E-05
A_55_P1961908	NM_001099324	Gm15217	100041724	-0,55636089	9,644407407	0,020297121
A_55_P2117028	NM_028860	Mtmr3	74302	-0,55681399	10,38918519	0,009938056
A_55_P2100864	NM_183250	Tma7	66167	-0,556991256	11,59841667	0,001235786
A_55_P2103626	NM_011989	Slc27a4	26569	-0,557649444	9,489083333	0,001953743
A_55_P2132800	AK041361	Zfp367	238673	-0,557757552	9,924509259	0,012923396
A_55_P2164428	NM_001033126	Cd27	21940	-0,558094595	9,205101852	0,002126623
A_51_P401263	NM_177752	Eme1	268465	-0,55895469	8,659953704	0,009488596
A_55_P2396375	AK007978	1810073O08Rik	72285	-0,559178855	10,26906481	0,011939115
A_52_P337259	NM_013905	Heyl	56198	-0,559210652	10,01710185	0,019786514
A_55_P1960423	AK018877	Atp8b3	67331	-0,559542925	9,712138889	0,021391995
A_55_P1990319	NM_025516	Ergic3	66366	-0,559891097	12,64469444	0,009624334
A_51_P261107	NM_139144	Ogt	108155	-0,559944356	7,722981481	2,80E-05
A_55_P2172822	AK008925	Mettl7a1	70152	-0,56032194	9,197546296	0,031205607
A_55_P2337138	AK003962	Ago2	239528	-0,561131955	8,342814815	0,001472733
A_51_P263667	NM_007391	Acrv1	11451	-0,561445151	8,00562963	0,009484255
A_55_P2168664	NM_007507	Atp5k	11958	-0,561626391	11,05224074	3,30E-05
A_51_P485594	NM_025729	Tab3	66724	-0,562538951	8,072203704	0,016782469
A_66_P101935	NM_010669	Krt6b	16688	-0,562715421	7,995759259	0,009879852
A_66_P104365	NM_026782	Ccdc167	68597	-0,562728935	8,41475	0,001117539
A_51_P480861	NM_021453	Pga5	58803	-0,563742448	8,213555556	0,005628682
A_51_P177491	NM_023182	Ctrl	109660	-0,565725755	8,485990741	0,008700917
A_55_P1960231	NM_019913	Txn2	56551	-0,566281399	11,18796296	0,016126905
A_55_P2145401	NM_175653	Hist1h3c	319148	-0,56717965	10,49275926	0,002247365
A_51_P282092	NM_009478	Urod	22275	-0,567520668	11,24073148	0,013771371
A_55_P1986213	NM_001004156	Plekhg5	269608	-0,567557234	9,486824074	0,039269362
A_66_P132496	AY261387	Asic3	171209	-0,567920509	8,330722222	0,006066438
A_52_P510877	NM_009743	Bcl2l1	12048	-0,568135135	9,312222222	0,012723103
A_55_P1979432	NM_011563	Prdx2	21672	-0,568141494	15,02212963	0,030505513
A_52_P87793	NM_147118	Olfr635	259122	-0,568911765	8,711601852	0,010521716
A_55_P2128324	NM_153489	Ubap2l	74383	-0,569030207	10,05231481	0,033980453
A_52_P238858	NM_001174086	Shisa9	72555	-0,569113672	11,27528704	0,031135885
A_51_P441745	AK021396	E130119H09Rik	78550	-0,569239269	9,752083333	0,078982064
A_55_P2155876	NM_001113395	Gm16501	100042840	-0,570476153	7,546324074	0,001717953
A_55_P2136906	NM_009514	Vpreb3	22364	-0,572011129	8,692537037	0,002664341

A_55_P2039896	NM_028873	Dnajc14	74330	-0,573238474	9,006666667	0,013957564
A_55_P1953545	NM_010238	Brd2	14312	-0,573290143	7,859101852	0,011114533
A_55_P2225460	AK157022	AW555355	99413	-0,573336248	9,941305556	0,00580606
A_55_P2251082	AK033279	Qk	19317	-0,573538156	8,964990741	0,006523313
A_51_P295315	NM_020033	Ankrd2	56642	-0,573799682	8,16337037	0,001491411
A_55_P1976574	NM_016712	Tmod4	50874	-0,574079491	8,880796296	0,011821572
A_51_P267700	NM_026860	Gkn3	68888	-0,576871224	9,200648148	0,004103585
A_66_P122219	NM_001039347	Kcnd3	56543	-0,577384738	9,445296296	0,015121753
A_55_P1964174	NM_008704	Nme1	18102	-0,577413355	9,05912963	0,002897839
A_52_P238523	NM_183147	Sprn	212518	-0,577709857	9,85025	0,019486788
A_55_P2065113	NM_001177484	Gm11559	100415785	-0,578258347	10,84613889	0,001858845
A_55_P1992889	NM_009776	Serping1	12258	-0,578427663	11,64761111	0,032011697
A_51_P506111	NM_019544	Msgn1	56184	-0,57872496	8,146407407	0,005658956
A_55_P2004354	NR_033430	Gm2694	100040294	-0,579180445	8,646212963	0,006753719
A_55_P2025008	NM_001042489	Hvcn1	74096	-0,579258347	12,2989537	0,001382983
A_55_P1973970	NM_212485	Krt73	223915	-0,579437202	10,72153704	0,024702114
A_66_P127024	NM_013595	Mbd3	17192	-0,579512719	10,24775	3,59E-06
A_55_P2001159	NM_025699	Oser1	66680	-0,580015898	11,27411111	0,002988081
A_55_P2083180	NR_026831	Gm10272	16697	-0,580174086	8,25362037	0,001865877
A_51_P172573	NM_013671	Sod2	20656	-0,580700318	10,64458333	0,010416948
A_66_P120605	NM_001146085	Fbxo34	78938	-0,580982512	8,420037037	0,009315135
A_55_P2136902	NM_001085500	Cisd3	217149	-0,582262321	11,02477778	0,00267833
A_55_P2152607	NM_172306	Cyp4a12b	13118	-0,582459459	8,019407407	0,000824411
A_55_P2208579	NM_194268	Onecut2	225631	-0,582617647	10,66141667	0,024120728
A_51_P237106	NM_001011804	Olfr1211	258025	-0,58282194	8,44062963	0,008346316
A_55_P2109032	NM_146840	Olfr545	258837	-0,582964229	8,66162037	0,015539325
A_55_P2083401	NM_001110239	Acp1	11431	-0,583976948	9,17125	0,035220781
A_55_P2108983	NM_001033378	A430078G23Rik	319493	-0,585720986	15,51973148	0,01582156
A_55_P2162432	NR_027847	D630029K05Rik	103175	-0,585805246	7,86937963	0,00211168
A_66_P110088	XM_001476538	Gm3265	100041308	-0,587626391	8,807925926	0,00206063
A_55_P2025468	NM_009437	Tst	22117	-0,587846582	9,267157407	0,007744278
A_55_P2169104	NM_011858	Tenm4	23966	-0,588417329	9,275472222	0,01358816
A_51_P139678	NM_009264	Sprr1a	20753	-0,588488076	11,23662963	0,00720638
A_55_P2035102	BC083122	Odc1	18263	-0,589667727	10,6567037	5,25E-06
A_51_P375771	NM_001093750	Ptchd1	211612	-0,589903021	9,137277778	0,027136698
A_52_P663704	NM_001013384	Podnl1	244550	-0,589935612	10,70749074	0,010738532
A_55_P2083368	AK144366	Gm2044	100039095	-0,590656598	10,33655556	0,007443124
A_55_P2009116	NR_033558	F830002L21Rik	414125	-0,592926073	7,509324074	0,00546925
A_52_P334796	NM_001008499	Taar4	209513	-0,593244833	12,11237037	0,054589382
A_51_P221337	NM_145824	Ranbp10	74334	-0,594325914	10,76512963	0,034864751
A_55_P2316760	AK020472	9430063H18Rik	77359	-0,594497615	8,94012963	0,00372165
A_55_P1977071	NM_028666	Fam110a	73847	-0,594629571	11,08633333	0,007798435
A_55_P2046499	NM_134228	Vmn1r70	171262	-0,594883943	11,47262963	0,004307484
A_55_P1979126	NM_009446	Tuba3a	22144	-0,595279809	10,85024074	0,008435834
A_66_P125962	NM_010432	Hipk1	15257	-0,595469793	8,953287037	0,010880272
A_52_P87503	NM_001033981	Hbq1b	544763	-0,59627186	12,4982963	0,011939115
A_55_P1973448	NM_016875	Ybx2	53422	-0,596705087	10,37117593	0,007427134
A_55_P2279140	AK089751	F830014O18Rik	403347	-0,597169316	10,56041667	0,076435484
A_55_P2088846	NM_172799	Ttll6	237930	-0,597297297	8,147240741	0,002247979
A_51_P452153	NM_027222	Mzb1	69816	-0,598025437	8,141388889	4,39E-08
A_52_P169901	NM_001009949	Slc25a51	230125	-0,598469793	11,03223148	0,079557124

A_55_P2163138	NM_029360	Tm4sf5	75604	-0,598470588	9,331407407	3,77E-05
A_55_P1996171	NM_029508	Pcgf5	76073	-0,599185215	10,49306481	0,001480374
A_55_P2063047	XM_001472600	Gm14206	100041796	-0,599294118	8,737166667	0,003029508
A_55_P2029121	AK007848	Pex11g	69129	-0,600378378	8,45812963	0,000389255
A_55_P2176802	NM_010432	Hipk1	15257	-0,600975358	9,25525	0,01186166
A_51_P150489	NM_031378	Gsdmc	83492	-0,60118601	9,12687037	0,007346067
A_55_P2001290	NM_009454	Ube2e3	22193	-0,601482512	10,38099074	0,004678966
A_55_P1960208	NM_172955	Vcan	13003	-0,601750397	8,790777778	0,00276999
A_55_P2008599	NM_001162946	Pcx	18563	-0,601980127	7,34812037	0,00149154
A_55_P2006148	NM_001177438	Aldh3b2	621603	-0,602533386	6,943564815	0,030585965
A_55_P2049186	NM_032398	Plvap	84094	-0,603352146	7,702268519	0,000891735
A_55_P1969511	NM_029430	Ttc23l	75777	-0,603883148	7,344787037	0,013716666
A_55_P2133910	NM_029264	Ttll10	330010	-0,605363275	12,58086111	0,039931834
A_66_P101013	NM_027673	Tssk4	71099	-0,605368839	8,329092593	0,007481512
A_55_P2244355	AK010586	2410024N13Rik	70000	-0,605575517	8,234333333	0,005359248
A_51_P120066	NR_033222	9330151L19Rik	414085	-0,605766296	7,395055556	0,001028728
A_51_P261835	NM_025376	Tmem8c	66139	-0,606286963	8,723787037	0,003260668
A_55_P2025746	NM_007401	Adam5	11499	-0,607076312	8,886481481	0,005579566
A_51_P513541	NM_019437	Rfk	54391	-0,607808426	10,32150926	0,028720928
A_55_P2096630	NM_199066	Ssxb9	387131	-0,608570747	11,45022222	0,018012061
A_55_P2101508	NM_010205	Fgf8	14179	-0,609538156	9,452324074	0,006152839
A_55_P1975266	NM_027010	Crygf	12969	-0,610100159	8,363703704	0,005707766
A_55_P1972792	NM_009185	Stil	20460	-0,610946741	9,029064815	0,012698337
A_51_P508770	NM_011846	Mmp17	23948	-0,611028617	8,820685185	0,003222674
A_55_P2416097	AK038153	A130082J08Rik	320015	-0,611177266	8,782083333	0,025776204
A_55_P2031636	NM_010512	Igf1	16000	-0,611709062	8,611277778	0,001831445
A_51_P131216	NM_030180	Usp54	78787	-0,611791733	10,89507407	0,007863254
A_55_P2022812	NM_001101445	Cyp4f17	208285	-0,61218283	10,00712963	0,042565761
A_52_P484838	NM_011266	Rfxank	19727	-0,614133545	8,238351852	3,28E-07
A_55_P2159885	NM_001166584	Tead1	21676	-0,614730525	10,59047222	0,025532049
A_55_P1980671	NM_001011743	Olfr324	257892	-0,615166137	9,34087963	0,020255862
A_51_P237040	NM_008711	Nog	18121	-0,61540461	10,34215741	0,013630649
A_52_P276525	NM_175207	Ankrd9	74251	-0,615558824	9,263787037	0,002730641
A_55_P2110200	NM_080562	Ubox5	140629	-0,615644674	9,018787037	0,006954828
A_66_P132870	AK039020	Fcnaos	545410	-0,615746423	8,466305556	0,002866507
A_55_P2170514	NM_018754	Sfn	55948	-0,615817965	7,771638889	0,000100302
A_52_P318673	NM_009117	Saa1	20208	-0,616033386	9,473314815	0,001839792
A_55_P1989928	NM_173756	Lin52	217708	-0,616133545	8,948481481	0,009031642
A_51_P131653	NM_029801	Tsacc	76927	-0,616248808	10,29549074	0,004614021
A_51_P300506	NM_183405	Cox6b2	333182	-0,616286169	10,37575926	0,068875458
A_55_P2015375	NM_009446	Tuba3a	22144	-0,617053259	11,35902778	0,005133677
A_55_P2045278	NR_033535	Gm10845	100038734	-0,61736089	13,71211111	0,006971495
A_51_P145511	NM_019991	Prl2a1	56635	-0,617513514	11,72738889	0,013155525
A_51_P310949	NM_008911	Ppox	19044	-0,617606518	10,48497222	0,006882274
A_51_P289588	NM_009454	Ube2e3	22193	-0,618488871	10,78525	0,003023602
A_51_P398833	NM_153154	Tfap2d	226896	-0,618572337	7,693722222	0,001085652
A_55_P1953728	NM_016701	Nes	18008	-0,619129571	8,896046296	0,00383222
A_55_P2397854	AK019540	4921518K17Rik	78758	-0,619947536	9,541074074	0,012074532
A_55_P2149382	NM_001123367	Gm3448	100041639	-0,620122417	9,792277778	0,003146305
A_55_P1985313	XM_001475212	Gm2703	100040307	-0,62013275	9,047064815	0,008337818
A_55_P2319035	BC051535	AW011956	105332	-0,620392687	7,849092593	0,003624938

A_55_P2312783	AK050884	D030029J20Rik	100502854	-0,620550079	10,07392593	0,004307484
A_55_P2077003	NM_009973	Csn1s2b	12992	-0,620665342	10,76143519	0,031449067
A_55_P2358399	NR_033213	1700111N16Rik	74305	-0,62122337	8,967435185	0,003741298
A_55_P2038362	NM_145444	Acot5	217698	-0,622764706	7,817555556	0,050065815
A_55_P2171993	NM_028479	Mrgbp	73247	-0,622992051	7,90237037	0,000253645
A_55_P2081840	NM_001126338	Prnd	26434	-0,623015898	9,901148148	0,016278623
A_55_P2045812	NM_011014	Sigmar1	18391	-0,623189984	9,745675926	0,0609324
A_52_P654752	NM_029839	Trub1	72133	-0,623190779	10,03335185	0,003802124
A_55_P1983368	NM_001162533	Sh3d21	66938	-0,623294118	11,07772222	0,002907826
A_55_P2032458	XM_893730	Gm6934	628919	-0,624941176	8,408240741	4,42E-05
A_55_P1997534	NM_001101463	Gm4871	231885	-0,625992846	8,576990741	0,003157876
A_55_P2045437	NM_177629	Fam216b	219170	-0,626183625	7,221712963	0,000323679
A_55_P2242089	AK041796	Cog5	238123	-0,626562003	8,900953704	0,002232871
A_66_P108685	NM_009894	Cideb	12684	-0,626706677	9,230472222	0,054777381
A_55_P2036605	NM_021318	Fhl5	57756	-0,627329094	9,178222222	0,005846495
A_55_P2225470	AK039069	A230092J17Rik	320669	-0,62772973	8,288888889	0,013531294
A_51_P259879	NM_173430	Fkrp	243853	-0,627845787	9,183074074	0,023198777
A_51_P295034	NM_010915	Klk1b4	18048	-0,628177266	8,720435185	0,005014815
A_55_P1987953	NM_001085534	Gm5938	546335	-0,628537361	9,19712963	0,007875388
A_55_P2073915	BC083183	Nos1	18125	-0,62913911	10,08880556	0,008011664
A_55_P2080598	NR_033355	5430416O09Rik	71406	-0,62918601	7,785185185	0,026581572
A_55_P2001334	AK036897	Gpr31b	436440	-0,629243243	10,30185185	0,012102512
A_55_P2089840	NM_001136070	Eif2d	16865	-0,629349762	8,589425926	0,001286375
A_55_P2126950	NM_001085417	Zfp467	68910	-0,629662957	9,230740741	0,010532809
A_55_P1997554	NM_080466	Kcnn3	140493	-0,630666932	9,380435185	0,007398508
A_55_P1986630	XM_001477989	Gm3627	100042018	-0,631391892	8,70587963	0,003293776
A_55_P2216996	NM_001110497	Tmem87a	211499	-0,631438792	10,77025926	0,008847503
A_51_P312846	NM_023605	Fbxo9	71538	-0,632114467	11,6537963	0,007398305
A_55_P2153990	NM_001177439	Tldc2	383766	-0,632538951	8,863240741	0,00305434
A_52_P352735	NM_001081369	Ccdc153	270150	-0,633272655	7,11462037	0,000407581
A_55_P2082929	NM_010389	H2-Ob	15002	-0,63381558	8,198777778	0,000204983
A_55_P2168014	NM_145404	Prmt7	214572	-0,634331479	7,799953704	0,001252066
A_51_P317433	NM_008589	Mesp2	17293	-0,6346407	11,98838889	0,010872818
A_55_P1953093	NM_148917	Pabpc4	230721	-0,634708267	9,722842593	0,001785206
A_55_P2186615	NM_001110239	Acp1	11431	-0,635361685	8,759953704	0,023037136
A_55_P2044385	NM_028263	Fgfbp3	72514	-0,635647059	10,04411111	0,024758544
A_51_P168613	NM_001004363	Nuak1	77976	-0,635809221	8,853	0,004551155
A_55_P1958697	NM_028234	Rbm33	381626	-0,636260731	9,255425926	0,001448842
A_55_P2258832	AK030345	5230400M03Rik	100502767	-0,636418919	9,138935185	0,010809111
A_66_P130916	NM_010389	H2-Ob	15002	-0,636962639	7,892268519	0,014898264
A_55_P2043862	NM_019641	Stmn1	16765	-0,637209062	10,12130556	0,002494232
A_55_P2070239	NM_016703	Preb	50907	-0,63727345	10,09505556	0,001259813
A_55_P1971734	NM_016678	Reck	53614	-0,637964229	7,693935185	0,001847059
A_55_P2002376	NM_009272	Srm	20810	-0,639000795	9,760842593	0,002277301
A_55_P2039429	NM_007860	Dio1	13370	-0,640709062	9,451407407	0,005332245
A_51_P322273	NM_008975	Ptp4a3	19245	-0,640771065	12,47512963	0,021595168
A_55_P2043833	NM_001081011	Srgap2	14270	-0,641422099	11,93421296	0,005213924
A_55_P2157260	NR_033225	Gm13375	433408	-0,643792528	9,330824074	0,00245032
A_55_P1996998	NM_009089	Polr2a	20020	-0,643809221	11,69501852	0,004954804
A_55_P2009066	NM_001166029	Cfap74	544678	-0,644777424	13,2772963	0,006802355
A_55_P2183288	NM_133679	Cryzl1	66609	-0,644900636	9,688064815	0,007558561

A_55_P2158384	NM_009591	Aanat	11298	-0,645116057	10,73074074	0,003445247
A_52_P24631	NM_018745	Azin1	54375	-0,646187599	8,446888889	0,004509031
A_55_P2070054	NM_030702	Senp3	80886	-0,647537361	11,46111111	0,000625156
A_52_P522977	NM_024272	Ssbp2	66970	-0,64768283	10,56630556	0,019889339
A_51_P483473	NM_011375	St3gal5	20454	-0,648032591	12,04221296	0,034083659
A_52_P276935	NM_001037940	Dnajb6	23950	-0,648676471	10,07071296	0,003860866
A_51_P358112	NM_146094	Fads1	76267	-0,648793323	8,204574074	0,017915921
A_55_P2374197	AK035904	Spag17os	320613	-0,651792528	8,507842593	0,014452702
A_66_P135700	NM_001104531	Cyp2d11	545123	-0,653255962	11,18822222	0,003766676
A_55_P2003592	BC059910	Unkl	74154	-0,654434817	8,47262037	0,006701804
A_55_P2108389	NM_177135	D830030K20Rik	320333	-0,657051669	9,271768519	0,009474022
A_55_P1993728	NM_001161627	Tmem116	77462	-0,657097774	10,51747222	0,019603633
A_51_P122035	NM_153578	Nipa1	233280	-0,657281399	7,583611111	0,001039262
A_52_P67212	NR_015605	2900052N01Rik	73040	-0,658387122	11,39432407	0,030662673
A_51_P343323	NM_053071	Cox6c	12864	-0,658586645	12,24298148	0,000156048
A_55_P1964348	NM_023608	Gdpd2	71584	-0,659508744	13,00255556	0,025773039
A_55_P2083988	NM_008505	Lmo2	16909	-0,660031797	11,09181481	0,000685911
A_55_P2108943	NM_009835	Ccr6	12458	-0,661036566	7,923685185	8,70E-05
A_66_P104296	NM_028275	1700112E06Rik	76633	-0,661761526	7,12512963	0,000298411
A_51_P198675	NM_138951	Ttc36	192653	-0,662228935	7,120574074	0,000547558
A_51_P230507	NM_138953	Ell2	192657	-0,662513514	9,000055556	0,01030317
A_55_P2014531	NM_033041	Hes7	84653	-0,662942766	8,942055556	0,001740091
A_55_P2061796	AK007238	1700122H20Rik	73617	-0,663669316	8,783851852	0,002624825
A_55_P2036788	NR_004444	Zfhx2os	432855	-0,663949126	11,31277778	0,004802285
A_55_P2384009	C81489	C81489	97547	-0,665306836	11,09275926	0,03180474
A_55_P2105970	NM_001122668	E330014E10Rik	665943	-0,666180445	8,197101852	0,000484938
A_51_P172251	NM_025903	Ifrd2	15983	-0,666186804	9,209009259	0,01538586
A_55_P1959091	NM_031193	Ren2	19702	-0,668117647	10,32683333	0,002561984
A_55_P1957729	NM_139219	Defb9	246079	-0,668843402	8,639953704	0,001518122
A_55_P2050508	NM_011465	Spta1	20739	-0,669299682	9,695935185	0,01157741
A_55_P1953402	NM_029993	Mlana	77836	-0,670503975	10,03446296	0,00262999
A_66_P124136	AK039088	Prkx	19108	-0,671294118	11,11683333	0,00330856
A_55_P2059680	NM_001127354	Gm11938	100041412	-0,67145628	11,72972222	0,014898264
A_55_P2076303	NM_027920	March8	71779	-0,672083466	10,25852778	0,006834381
A_55_P2053309	XM_889451	Rgs21	624910	-0,672586645	7,959888889	0,001137249
A_51_P481920	NM_009828	Ccna2	12428	-0,673542925	8,680527778	0,016080351
A_55_P1960053	NM_001042489	Hvcn1	74096	-0,674647854	8,843564815	0,00023187
A_55_P1970062	NM_001033323	Igsf9b	235086	-0,675347377	9,95737963	0,014933372
A_55_P2032024	NM_001081252	Uggt2	66435	-0,675442766	9,761490741	0,005931344
A_55_P2024909	NM_001167746	Dnah17	69926	-0,675856916	11,57464815	0,0090958
A_51_P421303	NM_026769	Caly	68566	-0,676246423	9,230351852	0,001410514
A_55_P2128821	NM_019979	Selk	80795	-0,678806836	11,90200926	1,12E-06
A_51_P151835	NM_001110013	Tmtc3	237500	-0,680317965	8,399444444	0,001300749
A_55_P2013188	NM_001037940	Dnajb6	23950	-0,681724165	10,20115741	0,002241875
A_55_P2009146	NM_032544	Gtpbp3	70359	-0,682509539	10,68347222	0,048117198
A_52_P125350	NM_023210	Anp32e	66471	-0,682756757	8,415185185	0,000675735
A_55_P1957911	AK004063	Tom1	21968	-0,682922099	11,03677778	0,026341207
A_51_P128575	NM_011681	Scgb1a1	22287	-0,684848967	8,240722222	0,001472733
A_55_P2331804	AK139027	AU015791	104932	-0,685131161	7,040175926	0,00504124
A_51_P501069	NM_011465	Spta1	20739	-0,685717806	9,521509259	0,024059204
A_55_P2020080	XM_001479627	Gm4326	100043267	-0,686375199	9,509	0,001270787

A_55_P2185526	XM_001002582	1700016P03Rik	668604	-0,687259141	12,10262963	0,008101024
A_55_P2129057	XM_912516	Gm7193	636947	-0,687399046	10,56959259	0,024281086
A_52_P608132	NM_001024560	Snx32	225861	-0,688027822	9,42725	0,001592419
A_55_P2015143	NM_144881	Hhat	226861	-0,690764706	9,961962963	0,012958333
A_52_P229972	NM_009202	Slc22a1	20517	-0,691445151	9,427555556	0,001622475
A_55_P1970159	NM_013876	Rnf11	29864	-0,693023847	12,22894444	0,002346228
A_55_P2026370	NM_027946	Dcaf7	71833	-0,693249603	9,185407407	0,003538804
A_55_P2006698	NR_033241	Bmp1	12153	-0,694383148	9,483277778	0,009810966
A_55_P2166123	NM_015731	Atp9a	11981	-0,697397456	11,57788889	0,007237909
A_55_P2005248	NM_008627	Meis3	17537	-0,697608903	11,21318519	0,030999623
A_55_P1964245	NM_146959	Olfr631	258961	-0,698110493	9,582694444	0,005492418
A_55_P1987231	NM_026928	Fuom	69064	-0,701972973	10,14801852	0,006154498
A_55_P2179309	NM_001122596	A630033H20Rik	213438	-0,701997615	11,21047222	0,020956733
A_51_P155152	NM_020332	Ank	11732	-0,702066773	8,880777778	0,006823724
A_55_P1980041	NM_008468	Kpna6	16650	-0,705976948	10,99915741	0,027037886
A_55_P2091985	NM_177366	Gpr157	269604	-0,707786963	12,70374074	0,056149499
A_51_P221632	NM_026091	1700037H04Rik	67326	-0,70790938	11,83083333	0,007363429
A_51_P351860	NM_009777	C1qb	12260	-0,70904849	9,095935185	0,006883091
A_55_P2060402	NM_153419	Grwd1	101612	-0,709131955	10,58490741	0,003323375
A_55_P2058871	NM_138604	Otud5	54644	-0,710103339	11,95538889	6,75E-05
A_55_P2154536	NM_001159626	Hagh	14651	-0,711695548	12,90276852	0,003976165
A_55_P2048518	NM_009569	Zfpm1	22761	-0,715267886	9,52337963	1,07E-05
A_55_P1993473	NM_001081657	Gm5935	546282	-0,715829889	11,77125926	0,010765861
A_55_P2060330	NM_199145	3110062M04Rik	78412	-0,717470588	11,70087037	0,0064703
A_55_P2145029	NM_001033411	Gm826	329554	-0,718340223	10,22933333	0,010408285
A_55_P2145449	NM_172260	Cep68	216543	-0,718541335	7,726842593	0,000213844
A_51_P156631	NM_028419	Glrx5	73046	-0,718682035	14,18698148	0,005976878
A_55_P2067798	NM_207543	Vmn1r59	404284	-0,719608108	9,852935185	0,003206829
A_55_P1968643	NM_001166718	Vmn1r173	545934	-0,719822734	10,23193519	0,005833146
A_55_P2100973	NM_016851	Irf6	54139	-0,720701908	11,31400926	0,007578378
A_51_P417891	NM_011280	Trim10	19824	-0,727824324	10,28530556	0,050212413
A_55_P2010641	NM_172479	Slc38a5	209837	-0,728219396	11,58624074	0,000373804
A_55_P1963549	NM_001166842	Vmn1r168	100043101	-0,729899841	11,71764815	0,002655001
A_55_P1994887	NM_009569	Zfpm1	22761	-0,73036089	9,592685185	3,31E-06
A_55_P2026639	NM_001100614	Gm11564	670496	-0,730539746	7,473268519	0,002029433
A_55_P2104071	NM_001012307	Defa23	497114	-0,734337838	11,25834259	0,005193884
A_55_P1970120	NM_146168	Vopp1	232023	-0,736702703	12,89322222	0,003952076
A_55_P2020326	NM_013591	Madcam1	17123	-0,739155803	11,08812963	0,010714306
A_55_P2113439	NM_021371	Caln1	140904	-0,7398593	10,78182407	0,004541317
A_51_P294643	NM_007672	Cdr2	12585	-0,740596184	12,32940741	0,01446503
A_51_P498772	NM_144953	1700019D03Rik	67080	-0,742526232	9,658055556	0,004321648
A_66_P136102	NM_177099	Lefty2	320202	-0,742567568	9,563703704	0,002628409
A_55_P2166069	NM_027173	2310079G19Rik	69699	-0,745355326	11,43617593	0,004481419
A_51_P432851	NM_177564	Dhrs11	192970	-0,746661367	10,11122222	0,030203936
A_51_P151020	NM_009407	Tnp1	21958	-0,746802067	8,786361111	0,000881161
A_51_P455647	NM_009801	Car2	12349	-0,746819555	11,71223148	0,092425887
A_51_P433388	NM_027070	1700019A02Rik	69397	-0,747201113	10,10267593	0,016189315
A_55_P1959985	NM_020559	Alas1	11655	-0,7485469	10,45751852	0,003503703
A_51_P520857	NR_004857	Gm12060	100134990	-0,750143084	11,08877778	2,53E-07
A_55_P2094049	AK139096	Gm15800	269700	-0,750666932	9,139712963	0,000901055
A_51_P504815	NM_025288	Stfa3	20863	-0,751536566	11,63617593	0,004480311

A_55_P2396446	AI593498	Dusp8	18218	-0,75172655	9,719722222	0,083690033
A_55_P2018111	NM_001101479	Pabpc4l	241989	-0,753807631	8,801296296	0,00231264
A_55_P1972590	NM_173396	Tgif2	228839	-0,756542925	9,696657407	0,00319152
A_66_P111534	AK017368	5430431A17Rik	71368	-0,757101749	8,093333333	0,079428237
A_55_P2004208	NM_007847	Defa-rs2	13222	-0,757279809	8,673740741	0,036658587
A_51_P269084	NM_175329	Chchd10	103172	-0,7580938	11,10838889	1,06E-05
A_55_P1969152	NM_030735	Vmn1r172	81010	-0,759391097	8,636	0,001152229
A_55_P2105140	NM_001177731	Mrap2	244958	-0,76108744	8,776925926	0,00122535
A_55_P2087622	NM_010215	Il4i1	14204	-0,761750397	7,956148148	6,52E-05
A_51_P291819	NM_024210	2310033P09Rik	67862	-0,761986486	10,87939815	0,0012484
A_55_P2154982	NM_009858	Cd8b1	12526	-0,763934817	11,25159259	0,000211724
A_51_P451151	NM_026785	Ube2c	68612	-0,763943561	12,27952778	0,009242518
A_55_P2138422	NM_027802	Obox1	71468	-0,765930048	11,88516667	0,020174525
A_55_P2052719	NM_001085410	Nadk2	68646	-0,767990461	9,871166667	0,002137252
A_55_P2023562	NM_199225	Cd300c	387565	-0,770017488	9,909277778	0,000902081
A_55_P2167123	NR_033575	Cyp4b1-ps2	631037	-0,770081081	10,15885185	0,010487294
A_55_P2080756	NM_001011749	Olfr704	257902	-0,78422337	11,95475	0,015578458
A_51_P103975	NM_027430	Mpc2	70456	-0,785816375	10,42530556	0,008011337
A_55_P2091323	NM_145514	Wdr26	226757	-0,786388712	8,239972222	0,00057415
A_55_P1961127	NR_001592	H19	14955	-0,789143084	10,97205556	0,007827823
A_55_P2081405	BC066226	BC052688	432812	-0,78986089	10,43841667	1,68E-07
A_66_P111449	AK142058	1700018A04Rik	71307	-0,791578696	10,33059259	0,003097471
A_55_P2021953	NM_146600	Olfr700	258593	-0,792252782	11,71727778	0,00701332
A_52_P414420	NM_033623	Dcun1d1	114893	-0,793383148	10,12344444	0,002717515
A_55_P2014570	NM_001164581	Zfp961	234413	-0,802374404	10,79334259	0,002028946
A_51_P496432	NM_007981	Acsl1	14081	-0,803787758	11,03563889	0,006882551
A_52_P354373	AK012387	1190002F15Rik	381822	-0,80436725	9,080888889	0,035573624
A_55_P1996941	NM_026785	Ube2c	68612	-0,809759936	11,51637037	0,005212352
A_66_P115406	NM_008774	Pabpc1	18458	-0,816570747	16,0467037	1,60E-05
A_51_P147034	NM_027407	Ica1l	70375	-0,819669316	9,851462963	0,087129595
A_55_P1960566	NM_139117	Ybx3	56449	-0,823790143	14,47096296	0,000760097
A_51_P154585	NM_134223	Vmn1r195	171257	-0,828693164	12,17157407	0,002294028
A_55_P2097393	NM_026132	Txndc8	67402	-0,836240859	10,12049074	0,00380138
A_55_P2099785	NM_027604	Usp15	14479	-0,840381558	10,89788889	0,001600569
A_51_P435251	NM_146207	Cul4a	99375	-0,840935612	9,727509259	0,001613565
A_55_P2051455	NM_178882	D2hgdh	98314	-0,854957075	9,596666667	0,013186686
A_66_P122155	NM_145514	Wdr26	226757	-0,858822734	10,54619444	0,000531916
A_52_P637730	NM_145405	Ubl4	27643	-0,860864865	9,326148148	1,52E-05
A_55_P2151855	NM_001110239	Acp1	11431	-0,868923688	10,16668519	0,006165157
A_55_P2161347	NM_001033041	Acmsd	266645	-0,889591415	7,798759259	9,28E-05
A_55_P2142668	XM_001480974	Gm4653	100043795	-0,895435612	10,79716667	0,069855444
A_55_P2073709	NM_178667	Tfdp2	211586	-0,896669316	12,4357037	0,001457678
A_51_P466591	AK005436	1600012P17Rik	72025	-0,903759141	11,13728704	0,003104604
A_52_P660945	NM_019861	Ctsf	56464	-0,909717011	9,366851852	0,003435587
A_55_P2178510	NM_008561	Mc3r	17201	-0,910364865	9,892731481	0,087125515
A_51_P376347	NM_013546	Hebp1	15199	-0,928251192	10,18724074	0,005022783
A_55_P2175579	NM_146694	Olfr1466	258689	-0,930243243	11,98911111	0,066843614
A_55_P1974587	NM_177545	Vangl1	229658	-0,949236089	8,563509259	0,000482223
A_51_P205968	NM_026912	Snx15	69024	-0,972065183	10,42092593	0,005399744
A_55_P2085546	NM_021422	Dnaja4	58233	-0,988234499	11,49193519	0,000212585
A_52_P612382	NM_023117	Cdc25b	12531	-0,989111288	12,48548148	0,005253016

A_55_P1957168	NM_133777	Ube2s	77891	-1,010892687	10,78652778	4,78E-05
A_55_P2117630	NM_030093	Snrnp25	78372	-1,049064388	9,505490741	6,89E-06
A_51_P329370	NM_030093	Snrnp25	78372	-1,053204293	11,20371296	2,83E-06
A_55_P2036813	NM_030082	Hist3h2ba	78303	-1,053439587	8,491287037	3,88E-05
A_55_P2043682	BC021811	Ubac1	98766	-1,077646264	12,10008333	0,000784235
A_55_P2043684	NM_133835	Ubac1	98766	-1,086734499	12,40544444	0,000617006
A_55_P2410875	AK034163	C030037D09Rik	193280	-1,109784579	8,506268519	0,003950051
A_52_P449417	NM_177545	Vangl1	229658	-1,252853736	8,536092593	1,32E-06
