# Supplementary material for: Of mice and men: the host response to influenza virus infection
Source: Mamm Genome. 2018 Jun 15;29(7):446–70. doi: 10.1007/s00335-018-9750-y (PMC6132725; doi:10.1007/s00335-018-9750-y)
Supplement: Supplementary file 8 — Supplementary material 8 (PDF 66 KB) [file 335_2018_9750_MOESM8_ESM.pdf]

ID	GB_ACC	Gene.Symbol	ENTREZ_GENE_ID	logFC	AveExpr	adj.P.Val
202411_at	NM_005532	IFI27	3429	3,416836825	8,150112117	0,008481459
213797_at	AI337069	RSAD2	91543	3,11434895	10,24745338	0,000548902
219519_s_at	NM_023068	SIGLEC1	6614	3,101833575	7,97324445	0,001166055
216598_s_at	S69738	CCL2	6347	3,088349425	4,258964367	0,009598804
203153_at	NM_001548	IFIT1	3434	3,059099125	10,10652852	0,000511411
204439_at	NM_006820	IFI44L	10964	2,91706445	10,72298278	0,00067711
214453_s_at	NM_006417	IFI44	10561	2,6899689	9,43015445	0,000548902
205483_s_at	NM_005101	ISG15	9636	2,514738625	11,75364757	0,000548902
219863_at	NM_016323	HERC5	51191	2,395255475	10,74451798	0,000548902
214038_at	AI984980	CCL8	6355	2,370504075	5,49217275	0,028369807
204747_at	NM_001549	IFIT3	3437	2,347163375	11,59648473	0,000511411
219211_at	NM_017414	USP18	11274	2,338219175	8,376424283	0,002352192
205569_at	NM_014398	LAMP3	27074	2,31282425	8,076373733	0,002036716
200986_at	NM_000062	SERPING1	710	2,304107198	9,590013849	0,001221931
44673_at	N53555	SIGLEC1	6614	2,2913736	8,59181705	0,001166055
214059_at	BE049439	IFI44	10561	2,2304782	9,289810767	0,001218806
205660_at	NM_003733	OASL	8638	2,195440475	10,17883643	0,001591348
219895_at	NM_017938	FAM70A	55026	2,152981085	5,255091088	0,00642036
204533_at	NM_001565	CXCL10	3627	2,128379275	6,289347767	0,010413123
218400_at	NM_006187	OAS3	4940	2,089504375	10,71898337	0,001609385
219364_at	NM_024119	DHX58	79132	2,050524575	7,772215433	0,003567845
222154_s_at	AK002064	SPATS2L	26010	2,045642975	8,906082633	0,001862144
204415_at	NM_022873	IFI6	2537	2,01578665	8,911632217	0,00166876
205552_s_at	NM_002534	OAS1	4938	2,00067055	10,30232257	0,001812051
202869_at	NM_016816	OAS1	4938	1,9880394	11,0382415	0,001591348
219684_at	NM_022147	RTP4	64108	1,839691725	8,9973835	0,000548902
202086_at	NM_002462	MX1	4599	1,83571475	12,38766333	0,001225711
202145_at	NM_002346	LY6E	4061	1,833352575	11,26279775	0,002368417
209999_x_at	AB005043	SOCS1	8651	1,8231806	5,637261433	0,026411531
211267_at	U82811	HESX1	8820	1,815933475	4,252610267	0,028369807
210657_s_at	U88870	Sep 04	5414	1,8111847	6,318408467	0,007400283
215495_s_at	AL117523	SAMD4A	23034	1,79468305	5,873432567	0,013287356
217502_at	BE888744	IFIT2	3433	1,771319575	10,83291507	0,003596228
204972_at	NM_016817	OAS2	4939	1,7692661	11,27776178	0,000967121
210797_s_at	AF063612	OASL	8638	1,757560875	10,3505182	0,001899777
203595_s_at	N47725	IFIT5	24138	1,726594	9,036730267	0,000548902
212845_at	AB028976	SAMD4A	23034	1,699512025	7,597603567	0,000548902
218943_s_at	NM_014314	DDX58	23586	1,6574062	9,426587017	0,00166876
202430_s_at	NM_021105	PLSCR1	5359	1,633638675	10,32412415	0,000548902
208087_s_at	NM_030776	ZBP1	81030	1,614758	8,270658533	0,002284383
206461_x_at	NM_005951	MT1H	4496	1,595310275	9,839666883	0,008745686
210362_x_at	AF230409	PML	5371	1,58779655	6,536435883	0,009041178
218986_s_at	NM_017631	DDX60	55601	1,576774925	9,517636683	0,001042023
202672_s_at	NM_001674	ATF3	467	1,575506025	7,04936455	0,01767391
211165_x_at	D31661	EPHB2	2048	1,54628275	3,822508417	0,020432649
203596_s_at	NM_012420	IFIT5	24138	1,543839475	8,273881767	0,00406341
202270_at	NM_002053	GBP1	2633	1,52937355	11,20040657	0,001049801
212185_x_at	NM_005953	MT2A	4502	1,508759525	11,40996353	0,006906823
220492_s_at	NM_004802	OTOF	9381	1,507645325	3,536635733	0,059379919
206026_s_at	NM_007115	TNFAIP6	7130	1,507290425	8,934334233	0,006403497
202269_x_at	NM_002053	GBP1	2633	1,50159385	11,37396322	0,000548902
215617_at	AU145711	SPATS2L	26010	1,497631835	4,606671588	0,031971642
208581_x_at	NM_005952	MT1X	4501	1,49501935	10,76977802	0,006224237
206133_at	NM_017523	XAF1	54739	1,477197075	9,1116344	0,007979514
208436_s_at	NM_004030	IRF7	3665	1,472801125	11,30074707	0,001691049
219209_at	NM_022168	IFIH1	64135	1,459353525	9,4908162	0,002284383
202446_s_at	AI825926	PLSCR1	5359	1,4547915	11,900317	0,000967121
219607_s_at	NM_024021	MS4A4A	51338	1,44819915	7,2556522	0,003060038
214511_x_at	L03419	FCGR1B	2210	1,43853385	10,11071977	0,001218806
221680_s_at	AF147782	ETV7	51513	1,438176475	7,678269617	0,004558106
209035_at	M69148	MDK	4192	1,437419775	4,4655717	0,011126309
219352_at	NM_017912	HERC6	55008	1,434361075	9,478887517	0,000965387
202687_s_at	NM_003810	TNFSF10	8743	1,4295577	10,49022747	0,002297425
217933_s_at	NM_015907	LAP3	51056	1,42159525	11,78747767	0,001218806
205899_at	NM_003914	CCNA1	8900	1,41647865	4,97967405	0,006715891
211456_x_at	AF333388	MT1P2	645745	1,394409525	10,692073	0,010128636
201427_s_at	NM_005410	SEPP1	6414	1,387352477	3,178567306	0,009496312
202688_at	NM_003810	TNFSF10	8743	1,37375335	11,58080297	0,001225711
210029_at	M34455	IDO1	3620	1,355721525	7,94623065	0,013868327
209417_s_at	BC001356	IFI35	3430	1,3398	10,70526545	0,002425849
207500_at	NM_004347	CASP5	838	1,33654785	7,89156385	0,010003105
216950_s_at	X14355	FCGR1A /// FCGR1C	100132417 /// 2209	1,32765825	9,8073919	0,00161738
210001_s_at	AB005043	SOCS1	8651	1,3207541	8,327655433	0,007382103
214329_x_at	AW474434	TNFSF10	8743	1,313591475	10,15529695	0,001812051
206025_s_at	AW188198	TNFAIP6	7130	1,312282025	9,507880283	0,014443822
210770_s_at	AF004884	CACNA1A	773	1,310188625	6,381108283	0,019945613
211012_s_at	BC000080	GOLGA6L4 /// PML	161527 /// 5371	1,302972025	9,276021017	0,005125854
219691_at	NM_017654	SAMD9	54809	1,2873197	8,075451633	0,003918023
222134_at	AL050350	DDO	8528	1,27565825	4,711586183	0,007570229
202898_at	NM_014654	SDC3	9672	1,262016	4,5201907	0,050894686
203773_x_at	NM_000712	BLVRA	644	1,25555055	9,5058766	0,000548902
210348_at	AF176379	Sep 04	5414	1,255092825	4,0311013	0,063908795
206503_x_at	NM_002675	PML	5371	1,251318375	7,4615978	0,003620745
214770_at	AI299239	MSR1	4481	1,234004193	4,060943662	0,080206237
219716_at	NM_030641	APOL6	80830	1,2271798	8,565767367	0,003949509
219062_s_at	BE676543	ZCCHC2	54877	1,214266475	9,514472083	0,001609385
202087_s_at	NM_001912	CTSL1	1514	1,211008775	7,591303133	0,000548902
202357_s_at	NM_001710	CFB	629	1,210598	4,406010433	0,026753924
58916_at	AI672101	KCTD14	65987	1,204797523	4,583303698	0,079698209
203052_at	NM_000063	C2	717	1,201821225	6,46140425	0,05418992
220722_s_at	AB043997	SLC5A7	60482	1,1971287	3,70830635	0,054634334
209640_at	M79462	PML	5371	1,1908059	7,78375125	0,008901264
206715_at	NM_012252	TFEC	22797	1,190180375	6,766375433	0,032073198
213294_at	AV755522	EIF2AK2	5610	1,186925225	10,89583028	0,001265153
205875_s_at	NM_016381	TREX1	11277	1,182701575	9,892661167	0,004558106

219545_at	NM_023930	KCTD14		65987	1,177062625	3,825739767	0,081439893
206553_at	NM_002535	OAS2		4939	1,169111975	8,466390983	0,004953048
211588_s_at	AF230401	LOC652346 /// PML	5371 /// 652346		1,1661146	6,420401783	0,035172735
205241_at	NM_005138	SCO2		9997	1,165379825	10,99413638	0,001218806
211599_x_at	U19348	MET		4233	1,163155625	5,488199833	0,041293016
218543_s_at	NM_022750	PARP12		64761	1,160922775	11,20398902	0,00166876
209498_at	X16354	CEACAM1		634	1,15274815	9,662269317	0,00814378
216244_at	BE563442	IL1RN		3557	1,15175471	2,294777243	0,083363532
211889_x_at	D12502	CEACAM1		634	1,1501679	8,36609795	0,020331917
202953_at	NM_000491	C1QB		713	1,147816	6,337800217	0,050253492
211729_x_at	BC005902	BLVRA		644	1,147110175	9,792951967	0,000548902
201641_at	NM_004335	BST2		684	1,147032625	10,54139162	0,001049801
218408_at	NM_012456	TIMM10		26519	1,1251258	8,733760067	0,006117055
214926_at	AK026484	SPTAN1		6709	1,11961135	4,410422317	0,019562161
201649_at	NM_004223	UBE2L6		9246	1,118538	12,608918	0,000548902
200923_at	NM_005567	LGALS3BP		3959	1,118491211	9,447323686	0,008850064
212659_s_at	AW083357	IL1RN		3557	1,111587275	10,11195153	0,020981765
213293_s_at	AA083478	TRIM22		10346	1,1078745	12,749676	0,000548902
219874_at	NM_024628	SLC12A8		84561	1,106781775	3,955245317	0,059466598
210610_at	M69176	CEACAM1		634	1,10504085	6,9308881	0,010413123
202837_at	NM_006700	TRAFD1		10906	1,09726415	9,450469233	0,002284383
204211_x_at	NM_002759	EIF2AK2		5610	1,0957219	9,618682267	0,003315563
205819_at	NM_006770	MARCO		8685	1,0951732	7,081760767	0,043488053
210271_at	AB021742	NEUROD2		4761	1,090808875	4,511727817	0,036389595
218435_at	NM_013238	DNAJC15		29103	1,089238725	6,5268982	0,003206471
221915_s_at	AI221318	RANBP1		5902	1,084135575	4,27756675	0,016235772
218232_at	NM_015991	C1QA		712	1,0671311	6,205367433	0,080206237
211434_s_at	AF015524	CCRL2		9034	1,0529066	7,023736133	0,011336596
208181_at	NM_003543	HIST1H4H		8365	1,05217453	4,139788832	0,024574447
205837_s_at	BC005319	GYPA		2993	1,040292775	4,188879067	0,098257507
209593_s_at	AF317129	TOR1B		27348	1,03693285	10,21902782	0,006852044
205972_at	NM_006841	SLC38A3		10991	1,032985825	3,780167667	0,032828665
204685_s_at	R52647	ATP2B2		491	1,032302475	4,44430465	0,05418992
206513_at	NM_004833	AIM2		9447	1,031107925	8,542884283	0,012052057
206740_x_at	NM_003176	SYCP1		6847	1,028717475	3,8140612	0,049199065
201015_s_at	NM_021991	JUP		3728	1,025934175	7,827481977	0,040423997
205043_at	NM_000492	CFTR		1080	1,014754675	3,145647317	0,006224237
212657_s_at	AW083357	IL1RN		3557	1,00675575	12,3739745	0,00470268
211883_x_at	M76742	CEACAM1		634	1,006506575	8,980666283	0,014833716
208134_x_at	NM_031246	PSG2		5670	1,0060405	3,735338867	0,032323922
213769_at	AA971768	KSR1		8844	1,004883825	4,5659436	0,034223692
209969_s_at	BC002704	STAT1		6772	1,00483375	11,49506817	0,000548902
217518_at	BF056029	MYOF		26509	1,00149135	6,5726128	0,025341334
220146_at	NM_016562	TLR7		51284	0,995492325	8,2387016	0,004558106
204162_at	NM_006101	NDC80		10403	0,994530075	5,44087505	0,021325436
204043_at	NM_000355	TCN2		6948	0,994170275	7,885097383	0,010579198
208912_s_at	BC001362	CNP		1267	0,986270275	9,800946917	0,003181381
209369_at	M63310	ANXA3		306	0,985065475	7,880192717	0,036826869
220194_at	NM_024677	NSUN7		79730	0,9824225	3,834047483	0,031971642
214633_at	AI824954	SOX3		6658	0,981668393	2,867495685	0,082254268
204224_s_at	NM_000161	GCH1		2643	0,977649	11,295742	0,001221931
208373_s_at	NM_004154	P2RY6		5031	0,97243145	5,594868583	0,065219861
220752_at	NM_016158	LOC51145		51145	0,968999225	3,934855583	0,034223692
221766_s_at	AW246673	FAM46A		55603	0,9685991	11,38651963	0,001428731
209568_s_at	AF186779	RGL1		23179	0,96713425	7,537547017	0,006467195
217165_x_at	M10943	MT1F		4494	0,96568245	9,4034437	0,006403497
221881_s_at	AI638420	CLIC4		25932	0,96510895	5,761753417	0,025177258
214727_at	X95152	BRCA2		675	0,95972642	4,913573893	0,024948337
216243_s_at	BE563442	IL1RN		3557	0,95591685	10,6107239	0,032831098
205907_s_at	AI765819	OMD		4958	0,95479504	3,647508962	0,097852925
206576_s_at	NM_001712	CEACAM1		634	0,954099	7,8428758	0,051414534
209546_s_at	AF323540	APOL1		8542	0,94932795	8,6601782	0,012344091
202620_s_at	NM_000935	PLOD2		5352	0,94737375	3,8741972	0,046511359
AFFX-HUMISGF3A/M97935_MA_at		STAT1		6772	0,94331025	8,90835265	0,044282857
204929_s_at	NM_006634	VAMP5		10791	0,94240355	8,217907733	0,0055085
203924_at	NM_000846	GSTA1		2938	0,942402775	3,779866017	0,054109315
213361_at	AW129593	TDRD7		23424	0,9385448	9,499393783	0,008209237
212285_s_at	AI424797	AGRN		375790	0,93531605	6,919188433	0,001185893
203771_s_at	AA740186	BLVRA		644	0,929273325	7,5531436	0,007068801
205170_at	NM_005419	STAT2		6773	0,925385925	8,245662	0,006138947
214647_s_at	BG402460	HFE		3077	0,921762075	5,1778527	0,009946281
206145_at	AF178841	RHAG		6005	0,91913585	4,388055183	0,06106351
211014_s_at	AF230410	GOLGA6L4 /// PML	161527 /// 5371		0,914936925	7,8114698	0,020331917
210705_s_at	AF220028	TRIM5		85363	0,913913075	9,115873733	0,005773397
208423_s_at	NM_002445	MSR1		4481	0,912018425	4,601846333	0,035172735
200887_s_at	NM_007315	STAT1		6772	0,904022831	12,6354284	0,000168336
210219_at	U36501	SP100		6672	0,89405705	5,686976283	0,083363532
213182_x_at	R78668	CDKN1C		1028	0,8937774	8,4810835	0,068521654
215311_at	AL109696	NTRK3		4916	0,892613175	4,56427495	0,083363532
211044_at	BC006333	TRIM14		9830	0,8890799	6,710763983	0,011126309
206637_at	NM_014879	P2RY14		9934	0,883367075	8,385475833	0,032268993
1255_g_at	L36861	GUCA1A		2978	0,883209066	3,578828695	0,060657156
209589_s_at	AF025304	EPHB2		2048	0,878474625	5,91003805	0,049530656
206842_at	NM_004979	KCND1		3750	0,87396135	5,1703548	0,078359687
AFFX-HUMISGF3A/M97935_MB_at		STAT1		6772	0,873680375	10,98548888	0,003181381
212658_at	N66633	LHFPL2		10184	0,87238905	8,304122	0,01092428
207433_at	NM_000572	IL10		3586	0,87154718	3,400087098	0,085715645
204994_at	NM_002463	MX2		4600	0,8710995	12,467533	0,00814378
214933_at	AA769818	CACNA1A		773	0,87099935	7,414682933	0,016280365
211013_x_at	AF230411	PML		5371	0,870012275	7,826450983	0,099191359
213348_at	N33167	CDKN1C		1028	0,867460275	9,7848135	0,082254268
205698_s_at	U39657	MAP2K6		5608	0,86575035	8,37857855	0,01294011
220119_at	NM_022140	EPB41L4A		64097	0,864188525	4,628097533	0,056789554
208966_x_at	AF208043	IFI16		3428	0,8624525	12,80580783	0,000871292
220791_x_at	NM_014139	SCN11A		11280	0,85932394	4,334944137	0,032445973
201798_s_at	NM_013451	MYOF		26509	0,8570987	9,316012883	0,005286017

218390_s_at	NM_022063	C10orf84	63877	0,85706105	5,181519233	0,06581545
208180_s_at	NM_003543	HIST1H4H	8365	0,85649765	7,121292633	0,032522298
205509_at	NM_001871	CPB1	1360	0,854158525	3,159649467	0,096047997
207777_s_at	NM_007237	SP140	11262	0,853179225	9,58527705	0,003174331
205098_at	AI421071	CCR1	1230	0,84953775	11,849644	0,004873064
34689_at	AJ243797	TREX1	11277	0,846932075	10,88517803	0,004558106
206800_at	NM_005957	MTHFR	4524	0,841082	5,827326583	0,036995366
204249_s_at	NM_005574	LMO2	4005	0,84083415	11,03945645	0,002755809
220315_at	NM_020367	PARP11	57097	0,837593725	6,568148217	0,0055085
207018_s_at	NM_004163	RAB27B	5874	0,834351335	4,574482763	0,057292104
219014_at	NM_016619	PLAC8	51316	0,833709	12,937934	0,000511411
207949_s_at	NM_004968	ICA1	3382	0,833437	5,519383817	0,053545557
208121_s_at	NM_002848	PTPRO	5800	0,8289008	6,883470633	0,003620745
203593_at	NM_012120	CD2AP	23607	0,8253716	7,929604633	0,008745686
208189_s_at	NM_000260	MYO7A	4647	0,8243002	4,430278417	0,094239632
221865_at	BF969986	C9orf91	203197	0,821922775	8,782801167	0,002036716
205897_at	NM_004554	NFATC4	4776	0,815891725	4,377088517	0,09267196
211967_at	BG538627	TMEM123	114908	0,81553075	11,73782167	0,001218806
219356_s_at	NM_016410	CHMP5	51510	0,812204175	8,4882197	0,015854548
205249_at	NM_000399	EGR2	1959	0,811942125	5,2582327	0,038518539
209283_at	AF007162	CRYAB	1410	0,811667525	4,930002183	0,049217191
220104_at	NM_020119	ZC3HAV1	56829	0,80881745	8,849686033	0,022850086
200629_at	NM_004184	WARS	7453	0,803461659	12,04706296	0,005351688
210353_s_at	M65105	SLC6A2	6530	0,80173885	3,128206267	0,096807635
209213_at	BC002511	CBR1	873	0,8004961	8,292461317	0,008326798
209684_at	AL136924	RIN2	54453	0,797627925	9,06250035	0,04208911
203236_s_at	NM_009587	LGALS9	3965	0,795285875	10,79010008	0,013287356
202863_at	NM_003113	SP100	6672	0,79278355	10,03539937	0,001257225
65517_at	AA910946	AP1M2	10053	0,791761525	5,667595317	0,09903155
35254_at	AB007447	TRAFD1	10906	0,7915041	10,6879094	0,005315547
210247_at	AW139618	SYN2	6854	0,787580075	5,898003183	0,052379197
204745_x_at	NM_005950	MT1G	4495	0,786432125	9,499332883	0,048388958
218429_s_at	NM_018381	C19orf66	55337	0,78637135	10,68893092	0,002036716
53720_at	AI862559	C19orf66	55337	0,78504915	10,4032295	0,00212728
209906_at	U62027	C3AR1	719	0,7838761	10,25313027	0,009598804
200628_s_at	M61715	WARS	7453	0,783791014	11,28756611	0,01397946
213988_s_at	BE971383	SAT1	6303	0,7821095	11,90174683	0,005267857
209457_at	U16996	DUSP5	1847	0,781374775	8,280758717	0,009946281
209392_at	L35594	ENPP2	5168	0,780151875	6,37663225	0,030536364
205099_s_at	AI421071	CCR1	1230	0,7791125	11,03627533	0,019667564
217371_s_at	Y09908	IL15	3600	0,77492925	7,1901815	0,00378776
218085_at	NM_015961	CHMP5	51510	0,772970275	8,054077717	0,023076056
217683_at	AA115963	HBE1	3046	0,7689525	5,3853586	0,088776777
203276_at	NM_005573	LMNB1	4001	0,768182975	10,06174748	0,004740669
212807_s_at	BF447105	SORT1	6272	0,76808445	8,19654045	0,005825176
204991_s_at	NM_000268	NF2	4771	0,76547805	5,26565155	0,096638502
220160_s_at	NM_007059	KPTN	11133	0,764273725	7,479018517	0,006301643
205692_s_at	NM_001775	CD38	952	0,76389525	8,05468545	0,034223692
216881_x_at	X07882	PRB4	5545	0,762076175	4,269616233	0,031356756
205228_at	NM_002898	RBMS2	5939	0,76073955	6,0559158	0,073870097
206697_s_at	NM_005143	HP	3240	0,758146925	7,058329983	0,0105847
206332_s_at	NM_005531	IFI16	3428	0,757582	12,54981517	0,002066354
206994_at	NM_001899	CST4	1472	0,75655995	5,984295	0,094544996
219439_at	NM_020156	C1GALT1	56913	0,753706225	7,590495417	0,024515166
203258_at	NM_006442	DRAP1	10589	0,753460725	9,57839215	0,006783954
AFFX-HUMISGF3A/M97935_5_at		STAT1	6772	0,7528495	11,88743767	0,000860951
211826_s_at	L22179	AFF1	4299	0,7492093	5,881622817	0,057756142
211864_s_at	AF207990	MYOF	26509	0,748560925	8,051617717	0,041882749
218686_s_at	NM_022450	RHBDF1	64285	0,744308975	3,638672833	0,083749425
204698_at	NM_002201	ISG20	3669	0,7425045	12,0833705	0,008850064
205773_at	NM_014912	CPEB3	22849	0,741147425	5,865376283	0,028455319
209893_s_at	M58596	FUT4	2526	0,740573675	7,247931133	0,007967346
205992_s_at	NM_000585	IL15	3600	0,7400979	8,253705	0,02231803
212681_at	AI770004	EPB41L3	23136	0,739754975	8,113816383	0,025177258
221245_s_at	NM_030804	FZD5	7855	0,738886675	5,570165517	0,012573163
202779_s_at	NM_014501	UBE2S	27338	0,73703175	8,9245636	0,032583519
204326_x_at	NM_002450	MT1X	4501	0,733256275	9,829240217	0,012933485
222217_s_at	BC003654	SLC27A3	11000	0,730885325	9,987626483	0,014652044
208176_at	NM_012146	DUX1	26584	0,728379875	4,760182183	0,085715645
205612_at	NM_007351	MMRN1	22915	0,727470225	4,45499585	0,075088039
217388_s_at	D55639	KYNU	8942	0,726793675	8,961108117	0,003121726
202284_s_at	NM_000389	CDKN1A	1026	0,7243666	9,183019967	0,027412228
33304_at	U88964	ISG20	3669	0,72049275	11,8072735	0,008701694
201762_s_at	NM_002818	PSME2	5721	0,71947025	11,99975183	0,000168336
200730_s_at	BF576710	PTP4A1	7803	0,718812497	8,493090633	0,039152888
205126_at	NM_006296	VRK2	7444	0,717550075	8,261565267	0,000967121
221816_s_at	BF055474	PHF11	51131	0,7126445	11,81222933	0,001265153
219010_at	NM_018265	C1orf106	55765	0,710819425	4,857418567	0,097581658
218815_s_at	NM_018022	TMEM51	55092	0,710469125	6,560123133	0,012024077
202949_s_at	NM_001450	FHL2	2274	0,707255675	7,007595383	0,072415168
212380_at	D43949	FTSJD2	23070	0,707176425	9,6882097	0,06106351
202864_s_at	NM_003113	SP100	6672	0,7068866	10,38382338	0,003350846
204205_at	NM_021822	APOBEC3G	60489	0,706884725	10,52542908	0,004631762
215754_at	AU148040	SCARB2	950	0,702739575	8,39260615	0,013223411
219806_s_at	NM_020179	C11orf75	56935	0,7023968	10,35787723	0,002472918
202307_s_at	NM_000593	TAP1	6890	0,7012015	12,24642317	0,005333777
213716_s_at	BF939675	SECTM1	6398	0,70037425	11,66886617	0,04666697
219799_s_at	NM_005771	DHRS9	10170	0,699628775	9,714486183	0,00791681
219403_s_at	AF155510	HPSE	10855	0,69452555	9,481992883	0,034527577
204038_s_at	NM_001401	LPAR1	1902	0,693570825	6,82809105	0,009793986
205420_at	NM_000288	PEX7	5191	0,691303725	5,52792275	0,080448531
203397_s_at	BF063271	GALNT3	2591	0,689419625	8,464972483	0,033261553
210659_at	U79526	CMKLR1	1240	0,6854708	6,813602983	0,019562161
210755_at	U46010	HGF	3082	0,6851228	4,870468133	0,034223692
218559_s_at	NM_005461	MAFB	9935	0,6848115	11,415389	0,038021913
210218_s_at	U36501	SP100	6672	0,683892675	8,5149427	0,027424948

201646_at	AA885297	SCARB2		950	0,683262075	7,52359505	0,03032375
217419_x_at	AK021586	AGRN		375790	0,6812175	6,62127195	0,016674239
209703_x_at	BC004492	METTL7A		25840	0,68033235	6,7043211	0,006218044
221827_at	BE788439	RBCK1		10616	0,678474475	10,54502212	0,003502905
206710_s_at	NM_012307	EPB41L3		23136	0,6760681	8,654287767	0,04208911
211138_s_at	BC005297	KMO		8564	0,673077775	6,935478267	0,022983764
203805_s_at	NM_000135	FANCA		2175	0,6719438	8,327598883	0,05418992
201761_at	NM_006636	MTHFD2		10797	0,66975685	8,465957367	0,005125854
207158_at	NM_001644	APOBEC1		339	0,66880135	5,202156167	0,035172735
210873_x_at	U03891	APOBEC3A		200315	0,6683444	11,48060873	0,033261553
211776_s_at	BC006141	EPB41L3		23136	0,66739755	8,2768131	0,026918929
204769_s_at	NM_000544	TAP2		6891	0,664416525	9,34700075	0,059834764
218776_s_at	NM_024956	TMEM62		80021	0,660555825	7,9186405	0,011126309
216336_x_at	AL031602	LOC100505584 /// MT1E	100505584 /// 4493		0,65587745	8,975934133	0,052290298
210152_at	U82979	LILRB4		11006	0,6536651	7,847623583	0,023136051
210985_s_at	AF056322	SP100		6672	0,65330885	6,811217133	0,05922265
220252_x_at	NM_025159	CXorf21		80231	0,651136625	7,829695783	0,055629308
210889_s_at	M31933	FCGR2B		2213	0,6506182	9,447685567	0,027744429
201138_s_at	NM_003142	SSB		6741	0,650507588	8,222118469	0,004558106
210224_at	AF031469	MR1		3140	0,649861025	8,510448533	0,003625851
204342_at	NM_013386	SLC25A24		29957	0,64899025	8,619880917	0,043751627
209762_x_at	AA969194	SP110		3431	0,6473955	11,9209545	0,00166876
206247_at	NM_005931	MICB		4277	0,6443669	10,14432493	0,006569381
205306_x_at	AI074145	KMO		8564	0,643981675	6,926619317	0,008745686
217508_s_at	BE783279	C18orf25		147339	0,64384525	7,125526267	0,029560763
221050_s_at	NM_019096	GTPBP2		54676	0,64218275	9,408555433	0,011712582
208965_s_at	BG256677	IFI16		3428	0,641567325	10,94070015	0,014833716
220358_at	NM_018664	BATF3		55509	0,641050375	7,331248717	0,041149375
203127_s_at	BC005123	SPTLC2		9517	0,64003485	9,97953815	0,013525545
218589_at	NM_005767	LPAR6		10161	0,639069225	7,8855913	0,079692659
209476_at	AL080080	TMX1		81542	0,638748875	8,826016983	0,056776863
216202_s_at	U15555	SPTLC2		9517	0,63851685	9,2523378	0,048718406
204804_at	NM_003141	TRIM21		6737	0,637332225	10,83201025	0,006954861
207574_s_at	NM_015675	GADD45B		4616	0,63196935	10,4531602	0,007114078
212735_at	AI798908	KIAA0226		9711	0,6318788	9,156799233	0,007400283
208012_x_at	NM_004509	SP110		3431	0,63127775	12,04471083	0,003181381
207091_at	NM_002562	P2RX7		5027	0,631240775	7,304427533	0,002755809
202609_at	NM_004447	EPS8		2059	0,6304014	6,01821025	0,048620774
220576_at	NM_024989	PGAP1		80055	0,624451325	7,132811233	0,080206237
203148_s_at	NM_014788	TRIM14		9830	0,61871965	10,78432527	0,001899777
205513_at	NM_001062	TCN1		6947	0,6173379	8,5172013	0,008255297
203964_at	NM_004688	NMI		9111	0,6171835	11,10132217	0,006113931
200042_at	NM_014306	C22orf28		51493	0,615404138	9,531863026	0,001042023
215209_at	AU143984	SEC24D		9871	0,615291875	7,066207183	0,055364973
201538_s_at	AL048503	DUSP3		1845	0,614217475	6,349327467	0,06286533
211760_s_at	BC005974	VAMP4		8674	0,613831425	6,33196855	0,041849885
219159_s_at	AL121985	SLAMF7		57823	0,6122372	7,528520833	0,031025882
209276_s_at	AF162769	GLRX		2745	0,60946005	8,6399492	0,051668437
221345_at	NM_005306	FFAR2		2867	0,60940375	12,33059517	0,014127287
201647_s_at	NM_005506	SCARB2		950	0,608309875	6,741370967	0,047735914
204385_at	NM_003937	KYNU		8942	0,6077453	7,761786283	0,023973574
219055_at	NM_018079	SRBD1		55133	0,60659185	8,622497317	0,001591348
221641_s_at	AF241787	ACOT9		23597	0,604579	9,730738317	0,044047326
209304_x_at	AF087853	GADD45B		4616	0,6036149	9,684357683	0,018386556
213734_at	BG260658	WSB2		55884	0,6016375	7,761853467	0,024170302
210592_s_at	M55580	SAT1		6303	0,59951475	13,1675555	0,001166055
214681_at	AI830490	GK		2710	0,599011925	8,050005783	0,068521654
209761_s_at	AA969194	SP110		3431	0,5982955	12,01583867	0,004915944
204279_at	NM_002800	PSMB9		5698	0,5977895	13,07501333	0,001428731
214022_s_at	AA749101	IFITM1		8519	0,596787	14,3788125	0,000548902
219885_at	NM_018042	SLFN12		55106	0,59666535	7,154164	0,055912033
218397_at	NM_018062	FANCL		55120	0,595295025	6,74513165	0,006218044
203817_at	W93728	GUCY1B3		2983	0,595109725	7,686329117	0,016344205
212647_at	NM_006270	RRAS		6237	0,591406975	8,577722617	0,094710866
215641_at	AK000709	SEC24D		9871	0,58867975	6,351566817	0,099569761
218974_at	NM_018013	SOBP		55084	0,58498095	6,0250747	0,0697034
217119_s_at	Z79783	CXCR3		2833	-0,589671025	7,202299083	0,045976069
208694_at	U47077	PRKDC		5591	-0,59023665	9,6177742	0,003663211
215471_s_at	AJ242502	MAP7		9053	-0,591282725	6,240397767	0,079766009
207838_x_at	NM_020524	PBXIP1		57326	-0,592770425	8,721529717	0,009973124
202438_x_at	BF346014	IDS		3423	-0,5941763	7,779958067	0,047552586
215005_at	AV723666	NECAB2		54550	-0,595475875	7,572225883	0,048620774
212823_s_at	AI738980	PLEKHG3		26030	-0,602497025	9,596144517	0,058807177
213478_at	AB028949	KAZ		23254	-0,605169825	8,44043125	0,049249054
203936_s_at	NM_004994	MMP9		4318	-0,605702	11,78504317	0,053769193
207681_at	NM_001504	CXCR3		2833	-0,607053225	7,78038395	0,090424903
220888_s_at	NM_020356	CASS4		57091	-0,607422675	6,984897	0,072191906
213549_at	AI890972	PDZD8		118987	-0,6078093	7,3371368	0,016235772
206208_at	NM_000717	CA4		762	-0,6146653	8,206182333	0,038021913
201392_s_at	BG031974	IGF2R		3482	-0,617679942	10,78173134	0,006113931
211413_s_at	AF229067	PADI4		23569	-0,617963025	8,4686933	0,026873504
206608_s_at	NM_020366	RPGRIP1		57096	-0,625159475	7,285609117	0,004745329
206209_s_at	NM_000717	CA4		762	-0,629178225	8,5646736	0,025341334
220797_at	NM_024086	METT10D		79066	-0,630161575	6,228995933	0,059463884
208031_s_at	NM_000635	RFX2		5990	-0,630534575	6,712832117	0,030444744
210791_s_at	BC000277	ARHGAP32		9743	-0,632138625	5,58830715	0,092887966
201188_s_at	NM_002224	ITPR3		3710	-0,63473611	7,436480215	0,055036253
213045_at	AB011133	MAST3		23031	-0,636778175	10,72903922	0,000168336
211926_s_at	AI827941	MYH9		4627	-0,64260185	11,35835177	0,001193076
203977_at	NM_000116	TAZ		6901	-0,6453888	7,6883706	0,091089516
203887_s_at	NM_000361	THBD		7056	-0,646319275	8,972305483	0,003174331
202316_x_at	NM_006048	UBE4B		10277	-0,647441	8,144630317	0,005649429
207826_s_at	NM_002167	ID3		3399	-0,656450525	8,021092617	0,096605725
213269_at	N21541	ZNF248		57209	-0,6607674	6,94849995	0,013559261
202888_s_at	NM_001150	ANPEP		290	-0,66353615	11,52011057	0,011883091
209460_at	AF237813	ABAT		18	-0,668238725	8,874626967	0,003181381

219499_at	NM_018144	SEC61A2	55176	-0,674799875	6,503313967	0,004740669
201971_s_at	NM_001690	ATP6V1A	523	-0,689098975	9,762147683	0,006224237
219452_at	NM_022355	DPEP2	64174	-0,7027175	11,81471217	0,002755809
204560_at	NM_004117	FKBP5	2289	-0,702910075	8,259279683	0,082264209
213354_s_at	AI935343	NR2F6	2063	-0,719156395	2,360531003	0,051668437
206653_at	BF062139	POLR3G	10622	-0,720499873	4,340159352	0,096183603
201739_at	NM_005627	SGK1	6446	-0,7242823	11,11854563	0,01074069
214321_at	BF440025	NOV	4856	-0,726460025	7,629588167	0,032954807
208331_at	NM_004678	BPY2	9083	-0,742906375	2,049988583	0,073579902
221874_at	AI565067	KIAA1324	57535	-0,7443185	7,683617117	0,087583958
217184_s_at	X52213	LTK	4058	-0,7447342	6,257859833	0,078359687
201416_at	NM_003107	SOX4	6659	-0,756992552	7,257064189	0,013800421
203888_at	NM_000361	THBD	7056	-0,76114925	8,064624417	0,00161738
213433_at	AF038193	ARL3	403	-0,78523005	5,74851695	0,025177258
213121_at	W67744	SNRNP70	6625	-0,802613275	5,534776683	0,047648119
204621_s_at	AI935096	NR4A2	4929	-0,803726125	4,950287833	0,071617956
203061_s_at	AI673553	MDC1	9656	-0,840510525	6,38444135	0,092322819
219102_at	NM_020650	RCN3	57333	-0,84160725	5,73309605	0,094904652
221309_at	NM_006450	RBM17	84991	-0,844356375	3,72772365	0,081005175
209184_s_at	AF073310	IRS2	8660	-0,85342125	10,13721555	0,000312938
222177_s_at	AF244812	SCAND2	54581	-0,86005935	3,57919045	0,073822694
206114_at	NM_004438	EPHA4	2043	-0,863923975	6,312910833	0,093243225
216156_at	AK022897	RECK	8434	-0,8676061	2,679308533	0,097300005
204990_s_at	NM_000213	ITGB4	3691	-0,8722181	4,757993917	0,098257507
215443_at	BE740743	TSHR	7253	-0,887292025	2,900311333	0,058440169
203463_s_at	H05668	EPN2	22905	-0,887386775	4,515493817	0,063249363
210147_at	U47054	ART3	419	-0,88922295	3,34489895	0,097935513
210100_s_at	AF327657	ABCA2	20	-0,903035525	4,177950417	0,082838926
221557_s_at	AF288571	LEF1	51176	-0,903775475	5,857425167	0,03288812
214804_at	BF793446	CENPI	2491	-0,916576925	3,554511467	0,088656355
210344_at	AF323729	OSBPL7	114881	-0,92371815	4,35796625	0,080206237
204428_s_at	NM_000229	LCAT	3931	-0,9291102	5,32313325	0,067135265
220654_at	NM_021092	PPY2	23614	-0,947117875	2,919426933	0,0278965
202291_s_at	NM_000900	MGP	4256	-0,9566876	3,915645417	0,088656355
220179_at	NM_022357	DPEP3	64180	-0,961319275	7,277552733	0,01010358
207092_at	NM_000230	LEP	3952	-0,965774325	3,57615175	0,09030058
209185_s_at	AF073310	IRS2	8660	-0,96914825	10,99525517	0,000548902
204896_s_at	AI675173	PTGER4	5734	-0,984387525	6,1845214	0,076937656
219935_at	NM_007038	ADAMTS5	11096	-0,988239863	5,294173475	0,055034527
203641_s_at	NM_014900	COBLL1	22837	-0,997092875	5,339731167	0,050894686
215559_at	AI074459	ABCC6	368	-1,010775225	5,0917009	0,056362057
205051_s_at	NM_000222	KIT	3815	-1,013066	5,688695867	0,015084322
220057_at	NM_020411	XAGE1A /// XAGE1B /// XAGE1C /// XAGE1D /// XAGE1E	653048 /// 653067 /// 653219 /// 653220 /// 9503	-1,01741745	3,61863855	0,028447476
206336_at	NM_002993	CXCL6	6372	-1,020612725	4,389767367	0,01397946
214418_at	AI656822	CT62	196993	-1,02799684	3,202188473	0,020331917
212793_at	BF513244	DAAM2	23500	-1,033015675	5,472154733	0,034223692
220727_at	NM_021161	KCNK10	54207	-1,0375374	4,60109045	0,01208934
220655_at	NM_024873	TNIP3	79931	-1,039184125	3,828719333	0,065647815
219313_at	NM_017577	GRAMD1C	54762	-1,050284875	5,928738633	0,005825176
209866_s_at	AF307080	LPHN3	23284	-1,0690162	3,098664383	0,027814841
205675_at	AI623321	MTTP	4547	-1,073331225	2,8390806	0,014833716
207712_at	NM_001187	BAGE	574	-1,110460215	3,67547599	0,032268993
220724_at	NM_025087	CWH43	80157	-1,12058087	2,124566577	0,014443822
204138_s_at	NM_003422	MZF1	7593	-1,2222996	5,0268451	0,013868327
218839_at	NM_012258	HEY1	23462	-1,259787	4,18629205	0,009598804
215757_at	AK022387	PRKDC	5591	-1,329783525	5,0021247	0,009344168
212158_at	AL577322	SDC2	6383	-1,414220843	4,217150755	0,038190435
