# Supplementary material for: Of mice and men: the host response to influenza virus infection
Source: Mamm Genome. 2018 Jun 15;29(7):446–70. doi: 10.1007/s00335-018-9750-y (PMC6132725; doi:10.1007/s00335-018-9750-y)
Supplement: Supplementary file 9 — Supplementary material 9 (PDF 57 KB) [file 335_2018_9750_MOESM9_ESM.pdf]

ID	Entrez_Gene_ID	Accession	Symbol	logFC	AveExpr	adj.P.Val
ILMN_1693192	5266	NM_002638.2	PI3	-1,539338919	8,926154617	8,91E-25
ILMN_1749722	57674	NM_020914.3	RNF213	-1,145663857	10,33022481	4,72E-08
ILMN_3235180	100131726	NR_024479.1	LOC100131726	-1,072862023	9,602176633	2,35E-08
ILMN_1701603	249	NM_000478.3	ALPL	-1,03081331	10,09125348	2,14E-09
ILMN_1701386	55437	NM_018571.5	STRADB	-1,01702362	9,651964027	1,71E-09
ILMN_1804938	131601	NM_016372.1	GPR175	-0,956332033	9,596753181	1,73E-10
ILMN_1804350	644852	XM_934213.1	LOC644852	-0,922333873	9,450221314	6,78E-10
ILMN_3249578	100132394	XM_001713809.1	LOC100132394	-0,922035337	9,544381149	3,36E-06
ILMN_1714765	389599	XM_001131588.1	LOC389599	-0,918189966	9,482311452	6,71E-09
ILMN_1788095	84926	NM_032840.1	SPRYD3	-0,91649679	9,971597973	8,20E-10
ILMN_3285762	100131164	XM_001721919.1	LOC100131164	-0,907545786	8,912155011	1,37E-09
ILMN_1733559	100008589	NR_003287.1	LOC100008589	-0,901595238	9,282886707	2,02E-06
ILMN_1749984	440313	NM_001013704.1	LOC440313	-0,898748169	8,902163282	1,55E-10
ILMN_1786328	25853	NM_015397.1	WDR40A	-0,896097571	10,0276043	1,23E-08
ILMN_1701933	6622	NM_007308.1	SNCA	-0,893827011	10,06553902	1,53E-05
ILMN_1706434	440359	XM_496143.2	LOC440359	-0,882446962	9,856530207	5,28E-08
ILMN_3276971	642469	XR_042342.1	LOC642469	-0,879013184	9,086513777	1,25E-09
ILMN_1679919	84164	NM_032204.3	ASCC2	-0,877305108	10,00718064	4,91E-11
ILMN_1745962	25793	NM_001033024.1	FBXO7	-0,858605268	9,708500723	9,56E-11
ILMN_1743046	388007	NM_207378.2	SERPINA13	-0,856877777	8,75306834	1,42E-10
ILMN_2404049	55544	NM_183425.1	RBM38	-0,856176661	9,357765989	5,17E-14
ILMN_1814397	2038	NM_000119.1	EPB42	-0,842985983	8,484056585	1,48E-11
ILMN_2084825	3048	NM_000184.2	HBG2	-0,82896688	11,40568245	0,005692866
ILMN_1804509	56896	NM_020134.2	DPYSL5	-0,828148785	9,01643875	6,33E-10
ILMN_1796678	3047	NM_000559.2	HBG1	-0,825696345	11,34564943	0,004820987
ILMN_2090105	8407	NM_003564.1	TAGLN2	-0,823961312	10,81208512	4,84E-21
ILMN_2060115	6653	NM_003105.3	SORL1	-0,794377187	9,287899207	4,08E-18
ILMN_3224856	729660	XR_039044.1	LOC729660	-0,789481299	9,443789165	9,92E-13
ILMN_1766165	6622	NM_000345.2	SNCA	-0,785794421	9,050607941	4,21E-06
ILMN_3183620	100128326	XR_038006.1	LOC100128326	-0,779953743	9,243497633	2,94E-14
ILMN_1750181	54997	NM_017899.2	TESC	-0,765285573	9,309491197	5,24E-09
ILMN_1743911	51629	NM_016016.1	SLC25A39	-0,753236762	12,31635234	8,23E-07
ILMN_1654118	598	NM_138578.1	BCL2L1	-0,750299209	9,882478639	2,43E-08
ILMN_2348487	84658	NM_032571.2	EMR3	-0,747643887	8,487966351	5,52E-29
ILMN_2072598	401357	NM_001013685.1	LOC401357	-0,741295768	9,04767459	1,35E-13
ILMN_1772731	3029	NM_005326.4	HAGH	-0,736430304	9,419813059	4,20E-09
ILMN_1809173	729021	XR_015834.1	LOC729021	-0,718956948	8,93373909	1,21E-16
ILMN_1657932	4588	NM_005961.2	MUC6	-0,717330699	8,699537106	1,29E-10
ILMN_3183139	100130914	XR_037646.1	LOC100130914	-0,711534033	8,914254564	5,97E-14
ILMN_1815527	3045	NM_000519.3	HBD	-0,710826993	10,02850573	0,000149348
ILMN_1762725	51386	NM_016091.2	EIF3L	-0,703667455	9,803868904	5,84E-17
ILMN_1783753	51060	NM_015913.2	TXNDC12	-0,702185216	8,440216803	4,48E-20
ILMN_3287583	648390	XR_037845.1	LOC648390	-0,700850067	11,28045454	4,54E-05
ILMN_2212763	3385	NM_002162.2	ICAM3	-0,695555905	11,18592617	3,50E-20
ILMN_1680652	8991	NM_003944.2	SELENBP1	-0,689073015	8,261827729	1,21E-09
ILMN_3240740	51386	NM_016091.2	EIF3L	-0,685644233	10,83753845	2,75E-13
ILMN_1715963	25793	NM_001033024.1	FBXO7	-0,683195948	8,465074144	3,84E-12
ILMN_2094061	3613	NM_014214.1	IMPA2	-0,676563227	8,687417383	2,21E-34
ILMN_1760714	6188	NM_001005.3	RPS3	-0,675037379	11,24473634	5,57E-11
ILMN_3283155	642357	XR_018361.2	LOC642357	-0,674181514	11,06503494	3,13E-11
ILMN_1759818	6653	NM_003105.3	SORL1	-0,668826219	9,825247005	1,30E-14
ILMN_1688322	51094	NM_015999.2	ADIPOR1	-0,66614303	10,375139	1,66E-06
ILMN_1782788	8531	NM_003651.3	CSDA	-0,665180504	11,72069094	2,30E-05

ILMN_1778374	682	NM_198590.1	BSG	-0,662007823	9,29090441	5,71E-12
ILMN_3249327	100134530	XM_001714618.1	LOC100134530	-0,660063573	8,679226484	6,15E-16
ILMN_1766539	643319	XM_927980.1	LOC643319	-0,654310315	8,787525346	1,55E-24
ILMN_1691892	8407	NM_003564.1	TAGLN2	-0,64027718	8,784338803	3,36E-22
ILMN_1744795	6907	NM_005647.2	TBL1X	-0,637137315	8,640005021	9,56E-33
ILMN_1738383	1938	NM_001961.3	EEF2	-0,627131233	10,96378571	4,63E-15
ILMN_2262288	1937	NM_001404.4	EEF1G	-0,623154506	10,80299106	1,73E-10
ILMN_1658624	80700	NM_025241.1	UBXN6	-0,622893607	9,45536758	1,93E-08
ILMN_2166831	6191	NM_001007.3	RPS4X	-0,619594777	10,12357268	1,20E-08
ILMN_1668039	2995	NM_016815.2	GYPC	-0,614945904	11,36381469	2,19E-06
ILMN_1796336	55201	NM_018174.4	MAP1S	-0,60739549	8,683192814	1,42E-09
ILMN_1682332	2995	NM_016815.2	GYPC	-0,606862414	10,4776392	1,03E-07
ILMN_1750130	2935	NM_002094.2	GSPT1	-0,605765271	10,24370272	1,18E-07
ILMN_3248247	100134634	XM_001726406.1	LOC100134634	-0,60410206	8,51150891	3,72E-17
ILMN_1810577	6191	NM_001007.3	RPS4X	-0,60336637	11,04915316	2,43E-07
ILMN_3211132	645173	XR_017590.2	LOC645173	-0,602099374	10,28395354	1,79E-09
ILMN_1715969	51312	NM_016612.2	SLC25A37	-0,601423303	10,28168898	5,14E-06
ILMN_3305772	730286	XR_038094.1	LOC730286	-0,600111798	8,462750798	7,43E-15
ILMN_1799028	10098	NM_005723.2	TSPAN5	-0,597555133	8,255369489	1,20E-12
ILMN_2059535	9647	NM_014634.2	PPM1F	-0,595474001	9,411614622	5,75E-20
ILMN_1707810	6193	NM_001009.3	RPS5	-0,592463788	10,90730648	2,45E-08
ILMN_1689160	64174	NM_022355.1	DPEP2	-0,591347682	9,27697483	2,86E-18
ILMN_1708934	133	NM_001124.1	ADM	0,587028653	9,965913101	1,85E-05
ILMN_1675848	10627	NM_006471.2	MYL12A	0,591752327	11,61677809	3,44E-15
ILMN_1664010	1997	NM_172373.2	ELF1	0,601047208	9,959324138	1,35E-13
ILMN_1751851	51816	NM_177405.1	CECR1	0,607116439	10,50653759	2,98E-13
ILMN_1783621	129607	NM_207315.2	CMPK2	0,609002261	7,959816718	4,98E-11
ILMN_1791759	3627	NM_001565.2	CXCL10	0,6118184	7,916770197	0,0001304
ILMN_2109489	3002	NM_004131.3	GZMB	0,615832873	9,375992761	8,59E-08
ILMN_1727271	7453	NM_173701.1	WARS	0,61589797	9,086594133	0,000167337
ILMN_3243928	91351	NM_001012967.1	DDX60L	0,616642976	8,251679133	5,99E-09
ILMN_2412214	3965	NM_009587.2	LGALS9	0,621910699	8,881755447	1,08E-08
ILMN_2088437	1524	NM_001337.3	CX3CR1	0,626358133	10,2952558	1,60E-09
ILMN_1807277	10437	NM_006332.3	IFI30	0,627311602	9,46294275	4,08E-09
ILMN_3223126	1890	NM_001113756.1	TYMP	0,628227751	8,858155468	8,12E-10
ILMN_1748473	55303	NM_018326.2	GIMAP4	0,628641188	11,27949937	8,75E-13
ILMN_1659047	8337	NM_003516.2	HIST2H2AA3	0,62897473	9,619535979	2,59E-06
ILMN_2170814	27074	NM_014398.2	LAMP3	0,630813108	7,963187734	6,40E-05
ILMN_2248970	4939	NM_001032731.1	OAS2	0,632467041	8,002406532	3,79E-12
ILMN_2384181	10170	NM_005771.3	DHRS9	0,634693101	8,51750059	1,32E-12
ILMN_3243061	100049587	NM_001098612.1	SIGLEC14	0,641781909	9,828922261	3,75E-05
ILMN_1776777	103	NM_001111.3	ADAR	0,645368549	10,91740433	3,34E-09
ILMN_1704972	85363	NM_033034.1	TRIM5	0,648714905	8,379553505	9,68E-12
ILMN_1678766	6993	NM_006519.1	DYNLT1	0,651771923	9,093176862	2,24E-09
ILMN_1814726	950	NM_005506.2	SCARB2	0,651853589	8,714538989	1,96E-16
ILMN_1678422	79132	NM_024119.2	DHX58	0,654144602	8,036399271	2,47E-11
ILMN_2374036	1514	NM_145918.2	CTSL1	0,654563007	8,224204787	1,27E-14
ILMN_1729801	6279	NM_002964.3	S100A8	0,656844524	11,58292037	1,92E-07
ILMN_2173975	64108	NM_022147.2	RTP4	0,658258813	8,01866817	1,12E-15
ILMN_3295494	389386	XR_037483.1	LOC389386	0,658477774	8,191104622	2,26E-10
ILMN_1745471	10379	NM_006084.4	IRF9	0,661758642	10,70990922	2,28E-17
ILMN_1691436	644	NM_000712.3	BLVRA	0,662288658	8,556430452	2,13E-25
ILMN_1769734	51251	NM_001002010.1	NT5C3	0,665831295	8,543569378	2,56E-09
ILMN_1764709	9935	NM_005461.3	MAFB	0,667112213	8,678643628	1,68E-11

ILMN_2186806	3134	NM_018950.1	HLA-F	0,668314833	11,78822293	3,71E-14
ILMN_1795181	55601	NM_017631.4	DDX60	0,67643212	8,146661005	9,78E-20
ILMN_2337655	7453	NM_004184.3	WARS	0,694281854	9,765732777	6,53E-05
ILMN_1709333	4939	NM_016817.2	OAS2	0,709838875	8,079916293	1,90E-12
ILMN_3240420	11274	NM_017414.3	USP18	0,71574447	8,00081466	2,68E-08
ILMN_2262044	84875	NM_032789.1	PARP10	0,718260992	9,000556665	3,32E-10
ILMN_2326512	834	NM_033294.2	CASP1	0,727294407	9,385653218	1,07E-15
ILMN_2326509	834	NM_033294.2	CASP1	0,733275564	9,406973521	2,97E-17
ILMN_1786612	5721	NM_002818.2	PSME2	0,734012729	10,31425024	1,29E-22
ILMN_2261600	2210	NM_001017986.1	FCGR1B	0,734071574	8,628946899	1,72E-07
ILMN_1681721	8638	NM_003733.2	OASL	0,742784993	8,154252761	5,17E-09
ILMN_2112301	10589	NM_006442.2	DRAP1	0,750449358	9,624155059	9,25E-16
ILMN_1716815	634	NM_001024912.1	CEACAM1	0,754624556	8,672024665	1,53E-06
ILMN_1701455	26270	NM_018438.4	FBXO6	0,760203504	8,379147617	1,06E-13
ILMN_2053527	83666	NM_031458.1	PARP9	0,766181545	8,601166787	4,26E-14
ILMN_1740466	55603	NM_017633.2	FAM46A	0,766608583	9,364189878	2,18E-08
ILMN_1731418	3431	NM_004510.2	SP110	0,766811886	10,06425138	1,27E-12
ILMN_1730628	6036	NM_002934.2	RNASE2	0,770385749	8,911173223	5,18E-14
ILMN_1800078	4005	NM_005574.2	LMO2	0,772953926	9,326156383	2,71E-12
ILMN_1703108	9246	NM_004223.3	UBE2L6	0,773568093	9,219478096	1,07E-09
ILMN_1733998	10170	NM_005771.3	DHRS9	0,775380333	9,169491053	5,74E-13
ILMN_2415144	3431	NM_004510.2	SP110	0,778420173	9,95136566	2,08E-12
ILMN_1737308	2745	NM_002064.1	GLRX	0,79246101	9,662212394	2,15E-19
ILMN_1745242	5359	NM_021105.1	PLSCR1	0,794619514	8,330436686	3,42E-12
ILMN_1809467	10791	NM_006634.2	VAMP5	0,794697074	9,047148298	1,97E-12
ILMN_3259146	100129681	XM_001726834.1	LOC100129681	0,79578657	9,254137229	6,77E-10
ILMN_2109708	1890	NM_001953.2	ECGF1	0,800573946	11,98883271	3,28E-10
ILMN_1736863	55281	NM_018295.2	TMEM140	0,808134669	9,96257708	2,07E-07
ILMN_3219806	643384	XR_016363.2	LOC643384	0,815641022	9,924732771	1,10E-11
ILMN_3251545	51510	NM_016410.4	CHMP5	0,816179326	8,604671032	1,59E-17
ILMN_1659913	3669	NM_002201.4	ISG20	0,82552048	10,96825234	2,17E-08
ILMN_1723480	684	NM_004335.2	BST2	0,826511401	9,378761755	4,41E-11
ILMN_1687768	135112	NM_181782.2	NCOA7	0,832577228	8,559991298	3,83E-09
ILMN_1835092	NA	BQ437417		0,839588577	8,146893495	1,10E-19
ILMN_1654639	55008	NM_017912.3	HERC6	0,852297939	8,414812963	5,52E-18
ILMN_1765332	26519	NM_012456.2	TIMM10	0,855314463	8,336116011	2,58E-11
ILMN_2390162	51131	NM_001040443.1	PHF11	0,855491223	9,769174745	1,48E-20
ILMN_1753342	6303	NM_002970.1	SAT1	0,869302107	11,69810888	1,76E-11
ILMN_1658247	4938	NM_002534.2	OAS1	0,869467411	8,523022878	3,86E-08
ILMN_1683678	26010	NM_001100422.1	SPATS2L	0,890760857	8,202614282	8,34E-12
ILMN_1776723	51131	NM_001040443.1	PHF11	0,894524171	9,933285021	2,38E-20
ILMN_2352121	51251	NM_001002010.1	NT5C3	0,899564387	9,197420415	6,19E-12
ILMN_2370573	54739	NM_199139.1	XAF1	0,90302437	8,383157644	2,08E-15
ILMN_2231928	4600	NM_002463.1	MX2	0,90465081	9,908021489	2,31E-07
ILMN_1691731	54625	NM_017554.1	PARP14	0,921711861	9,155195548	9,26E-10
ILMN_1771385	115361	NM_052941.3	GBP4	0,940055286	8,954092691	7,37E-09
ILMN_1690105	6772	NM_007315.2	STAT1	0,968089198	9,394385904	2,05E-13
ILMN_1736729	4939	NM_002535.2	OAS2	0,978594746	8,316972372	2,69E-16
ILMN_1663347	9381	NM_194323.1	OTOF	0,999512569	8,232027537	2,09E-14
ILMN_1710937	3428	NM_005531.1	IFI16	1,000883082	9,93339784	2,53E-16
ILMN_1701621	9997	NM_005138.1	SCO2	1,001412807	8,97134367	1,04E-15
ILMN_1751079	6890	NM_000593.5	TAP1	1,011239689	10,38168646	8,81E-19
ILMN_2139100	51246	NM_016479.3	SHISA5	1,017932573	10,89853371	9,36E-22
ILMN_1774874	3557	NM_173843.1	IL1RN	1,02199061	9,446498191	3,04E-07

ILMN_1689734	3557	NM_173842.1	IL1RN	1,022396483	9,3019285	1,63E-07
ILMN_1787509	85441	NM_033405.2	PRIC285	1,026530956	9,377259904	1,55E-11
ILMN_1653026	51316	NM_016619.1	PLAC8	1,036311496	10,25665911	9,66E-19
ILMN_1758418	10673	NM_006573.3	TNFSF13B	1,050399657	9,998121261	6,52E-09
ILMN_1781373	64135	NM_022168.2	IFIH1	1,058109357	8,659576755	8,36E-15
ILMN_1785732	7130	NM_007115.2	TNFAIP6	1,059298488	8,948451133	9,62E-10
ILMN_1691364	6772	NM_139266.1	STAT1	1,073190839	10,96659429	1,32E-12
ILMN_1777325	6772	NM_007315.2	STAT1	1,080031274	9,916301691	2,41E-21
ILMN_1670305	710	NM_001032295.1	SERPING1	1,081687669	8,340860761	2,25E-10
ILMN_1718558	64761	NM_022750.2	PARP12	1,083208342	8,995368968	8,85E-15
ILMN_2093343	51316	NM_016619.1	PLAC8	1,098339776	10,26654449	3,07E-19
ILMN_2066858	10673	NM_006573.3	TNFSF13B	1,108297235	10,61940794	2,12E-10
ILMN_1653466	57801	NM_021170.2	HES4	1,110435168	8,625483851	2,72E-09
ILMN_2114568	115362	NM_052942.2	GBP5	1,122013216	9,401610069	2,63E-11
ILMN_1731224	83666	NM_031458.1	PARP9	1,151811949	9,323301261	9,99E-18
ILMN_1769520	9246	NM_004223.3	UBE2L6	1,162843929	11,32402261	5,84E-13
ILMN_1814305	54809	NM_017654.2	SAMD9	1,165401116	9,117334207	3,38E-19
ILMN_1690921	6773	NM_005419.2	STAT2	1,201861857	10,17799951	1,31E-13
ILMN_1745374	3430	NM_005533.2	IFI35	1,21504202	9,215612963	4,56E-13
ILMN_2349061	3665	NM_004029.2	IRF7	1,224596224	8,745083463	2,25E-15
ILMN_1724139	114908	NM_052932.2	TMEM123	1,264336188	10,49284278	1,25E-25
ILMN_1683792	51056	NM_015907.2	LAP3	1,270227138	9,25120366	6,39E-16
ILMN_1801307	8743	NM_003810.2	TNFSF10	1,271937255	9,929154005	2,21E-11
ILMN_1765994	81030	NM_030776.1	ZBP1	1,298074483	9,386652856	2,76E-14
ILMN_1801246	8519	NM_003641.3	IFITM1	1,301161577	11,58925582	1,00E-19
ILMN_1706502	5610	NM_002759.1	EIF2AK2	1,311816249	9,289789399	8,26E-19
ILMN_1799467	219285	NM_152703.2	SAMD9L	1,31569086	9,152374394	1,00E-17
ILMN_1779252	10346	NM_006074.3	TRIM22	1,350750532	9,566756931	2,22E-23
ILMN_1701114	2633	NM_002053.1	GBP1	1,367021837	9,141417527	8,94E-16
ILMN_1691156	4489	NM_005946.2	MT1A	1,588099508	9,945051064	5,11E-12
ILMN_2148785	2633	NM_002053.1	GBP1	1,652750292	9,788693202	1,04E-18
ILMN_1674811	8638	NM_198213.1	OASL	1,678871156	9,19617466	2,28E-15
ILMN_1798181	3665	NM_004029.2	IRF7	1,763426617	10,33888089	2,42E-15
ILMN_1745397	4940	NM_006187.2	OAS3	1,814881282	9,157653963	2,22E-18
ILMN_1675640	4938	NM_001032409.1	OAS1	1,841839437	9,422582851	5,04E-21
ILMN_1742618	54739	NM_199139.1	XAF1	1,855563647	9,799098112	9,47E-29
ILMN_1687384	2537	NM_022873.2	IFI6	1,920510013	11,51819674	1,62E-25
ILMN_1664543	3437	NM_001031683.1	IFIT3	1,949858918	9,340597495	2,00E-19
ILMN_2410826	4938	NM_001032409.1	OAS1	1,969922901	9,542106883	1,29E-22
ILMN_1739428	3433	NM_001547.4	IFIT2	2,063362349	11,13344291	1,30E-20
ILMN_1674063	4939	NM_016817.2	OAS2	2,082168906	10,44494827	3,88E-24
ILMN_2239754	3437	NM_001549.2	IFIT3	2,170369291	10,04801514	1,22E-22
ILMN_1729749	51191	NM_016323.2	HERC5	2,221454767	10,16081991	5,63E-19
ILMN_2347798	2537	NM_022872.2	IFI6	2,293082318	10,37338612	1,73E-22
ILMN_1701789	3437	NM_001031683.1	IFIT3	2,329062395	10,10359673	1,16E-20
ILMN_1662358	4599	NM_002462.2	MX1	2,339669439	11,59206363	9,90E-28
ILMN_1760062	10561	NM_006417.3	IFI44	2,414594587	9,613238894	2,79E-36
ILMN_2388547	94240	NM_033255.2	EPSTI1	2,470387279	10,26677337	7,79E-36
ILMN_1657871	91543	NM_080657.4	RSAD2	2,522601824	9,604186356	4,54E-22
ILMN_1695404	4061	NM_002346.1	LY6E	2,727461271	11,79369241	8,67E-52
ILMN_2054019	9636	NM_005101.1	ISG15	2,765050279	10,12319327	1,42E-22
ILMN_1707695	3434	NM_001548.3	IFIT1	2,772827094	10,41005803	2,25E-27
ILMN_1805750	10410	NM_021034.2	IFITM3	2,87202849	12,00608677	8,60E-33
ILMN_1723912	10964	NM_006820.1	IFI44L	3,148803279	10,16068476	2,86E-36

ILMN_2058782	3429	NM_005532.3	IFI27	3,974087743	10,59696181	6,38E-53
