# Supplementary material for: Of mice and men: the host response to influenza virus infection
Source: Mamm Genome. 2018 Jun 15;29(7):446–70. doi: 10.1007/s00335-018-9750-y (PMC6132725; doi:10.1007/s00335-018-9750-y)
Supplement: Supplementary file 10 — Supplementary material 10 (PDF 118 KB) [file 335_2018_9750_MOESM10_ESM.pdf]

ProbeID	EntrezGeneID	GeneSymbol	EnsemblID	logFC	AveExpr	adj.P.Val
A_24_P270460	3429	IFI27	ENST00000614648	6,257478286	12,35123875	2,65E-09
A_24_P28722	91543	RSAD2	ENST00000382040	2,944864219	10,92596736	0,000763662
A_23_P45871	10964	IFI44L	ENST00000476521	2,816445057	10,40212251	0,001685274
A_23_P23074	10561	IFI44	ENST00000485662	2,527650143	10,24651081	0,000629558
A_33_P3270451	81567	TXNDC5	ENST00000439343	2,375818842	8,833780228	0,002074862
A_23_P52266	3434	IFIT1	ENST00000371804	2,365883703	11,3138445	0,004620893
A_23_P819	9636	ISG15	ENST00000379389	2,324864708	13,47354412	0,002871155
A_33_P3257678	333932	HIST2H3A	ENST00000369158	2,310010298	8,997379827	6,42E-05
A_23_P105794	94240	EPSTI1	ENST00000476830	2,142969035	8,870419654	6,59E-05
A_23_P107421	7083	TK1	ENST00000301634	2,130194223	10,48616632	1,23E-05
A_23_P118815	332	BIRC5	ENST00000301633	2,108317304	8,251930048	3,32E-05
A_23_P118815	332	BIRC5	ENST00000301633	2,071583766	8,087570969	3,27E-05
A_23_P139786	8638	OASL	ENST00000257570	2,058592341	11,02249251	0,000424949
A_23_P58266	6286	S100P	ENST00000296370	2,048259371	12,99440848	0,012264985
A_23_P50096	7298	TYMS	ENST00000581920	2,031754153	8,388210603	7,00E-05
A_23_P118815	332	BIRC5	ENST00000301633	2,019296277	8,066779068	4,48E-05
A_24_P304071	3433	IFIT2	ENST00000611722	2,003544772	11,44364669	0,000862597
A_23_P118815	332	BIRC5	ENST00000301633	2,003358633	8,148784983	5,03E-05
A_23_P118815	332	BIRC5	ENST00000301633	2,001854214	8,108876566	5,84E-05
A_33_P3283611	3437	IFIT3	ENST00000371818	1,994893745	11,76424845	0,004196077
A_23_P165624	7130	TNFAIP6	ENST00000460812	1,990450953	9,946359949	1,43E-05
A_23_P118815	332	BIRC5	ENST00000301633	1,98886821	8,081836618	5,62E-05
A_23_P118815	332	BIRC5	ENST00000301633	1,985054415	8,082820696	5,40E-05
A_23_P118815	332	BIRC5	ENST00000301633	1,980398152	8,071630711	5,62E-05
A_23_P118815	332	BIRC5	ENST00000301633	1,972937179	8,080495482	6,89E-05
A_24_P335305	4940	OAS3	ENST00000228928	1,961296385	9,421855509	0,005263153
A_23_P118815	332	BIRC5	ENST00000301633	1,950975285	8,145700012	7,00E-05
A_23_P65757	9133	CCNB2	ENST00000621385	1,931854168	7,927817943	6,10E-05
A_23_P137366	713	C1QB	ENST00000314933	1,853796831	8,243358673	0,000889354
A_23_P17481	6614	SIGLEC1	ENST00000344754	1,850220152	7,413576244	0,002228749
A_24_P317762	4061	LY6E	ENST00000522024	1,835152794	11,35332732	0,005979722
A_23_P253791	820	CAMP	ENST00000296435	1,833334862	11,45515576	0,018895912
A_23_P206760	3240	HP	ENST00000566821	1,824794733	10,90749996	0,012453462
A_23_P370682	116071	BATF2	ENST00000527454	1,809349707	9,411509125	0,001424593
A_23_P201459	2537	IFI6	ENST00000362020	1,782125086	11,35230603	0,003796519
A_23_P151637	6036	RNASE2	ENST00000304625	1,770873188	9,950319811	0,00048109
A_33_P3401826	129607	CMPK2	ENST00000478738	1,763800263	9,5644862	0,009995386
A_23_P167168	3512	IGJ	ENST00000543780	1,753318646	9,480226541	0,005473513
A_23_P64828	4938	OAS1	ENST00000549820	1,750982514	9,32212311	0,001246782
A_33_P3319967	383	ARG1	ENST00000356962	1,731191129	8,045425973	0,005188418
A_23_P104651	113130	CDCA5	ENST00000275517	1,728025674	7,728931022	3,32E-05
A_23_P434809	6279	S100A8	ENST00000368733	1,727848741	16,36796604	7,03E-05
A_23_P84596	51237	MZB1	ENST00000503351	1,720497846	9,825160324	0,009071715
A_23_P17663	4599	MX1	ENST00000455164	1,709885441	12,78661363	0,021153415
A_23_P141173	4353	MPO	ENST00000225275	1,70810179	8,625572204	0,02572142
A_23_P117852	9768	KIAA0101	ENST00000300035	1,701791669	7,64702773	0,000667768
A_23_P167328	952	CD38	ENST00000502843	1,687999736	8,951974698	8,44E-05
A_33_P3352382	383	ARG1	ENST00000356962	1,686280427	8,045552377	0,005128552
A_23_P166848	4057	LTF	ENST00000426532	1,668983116	10,13049142	0,09046657
A_23_P75769	51338	MS4A4A	ENST00000529991	1,668799452	8,818885707	6,42E-05
A_23_P132159	11274	USP18	ENST00000215794	1,651400604	8,110604251	0,01153739
A_24_P557479	54739	XAF1	ENST00000361842	1,627473275	10,58278987	0,003507829

A_23_P139123	710	SERPING1	ENST00000531133	1,622389999	7,855447526	0,005541963
A_33_P3413989	710	SERPING1	ENST00000405496	1,604979929	7,860549858	0,00788188
A_33_P3258346	54739	XAF1	ENST00000576724	1,601044149	9,18658907	0,002503771
A_23_P117852	9768	KIAA0101	ENST00000300035	1,595327013	7,577495893	0,001002298
A_23_P74001	6283	S100A12	ENST00000368737	1,595261619	14,09042841	0,0008398
A_22_P00013137	643332	ECRP	NA	1,56953289	8,329685384	0,000380289
A_33_P3289236	3250	HPR	ENST00000356967	1,560049331	9,709529154	0,011757148
A_23_P259141	81030	ZBP1	ENST00000461547	1,559468195	10,5665657	0,000455869
A_33_P3385785	6283	S100A12	ENST00000368737	1,554763022	13,29623173	0,00134545
A_23_P117852	9768	KIAA0101	ENST00000300035	1,540566982	7,468770934	0,001104415
A_23_P259863	57126	CD177	ENST00000618265	1,533809253	7,579615712	0,039319292
A_23_P117852	9768	KIAA0101	ENST00000300035	1,528099888	7,439131341	0,001124636
A_23_P110196	51191	HERC5	ENST00000502913	1,511854256	8,914590376	0,00863065
A_33_P3386262	81620	CDT1	ENST00000301019	1,511339395	7,385830816	6,53E-05
A_23_P250385	3009	HIST1H1B	ENST00000331442	1,510991914	7,611203159	0,000195013
A_32_P108254	54757	FAM20A	ENST00000375556	1,507097764	7,364646463	0,000677632
A_23_P117852	9768	KIAA0101	ENST00000300035	1,498413546	7,516609388	0,001577108
A_24_P261929	122509	IFI27L1	ENST00000393115	1,495989069	9,988492514	1,50E-05
A_23_P117852	9768	KIAA0101	ENST00000300035	1,493415345	7,462195213	0,001428342
A_23_P117852	9768	KIAA0101	ENST00000300035	1,484794103	7,364166741	0,001252098
A_33_P3400389	26010	SPATS2L	ENST00000409718	1,483561277	8,360128106	0,008614181
A_23_P117852	9768	KIAA0101	ENST00000300035	1,475961769	7,479934776	0,001491537
A_23_P117852	9768	KIAA0101	ENST00000300035	1,473050283	7,370002718	0,001166398
A_23_P169437	3934	LCN2	ENST00000373017	1,468428705	9,360433929	0,049154766
A_23_P117852	9768	KIAA0101	ENST00000300035	1,464160186	7,420574853	0,001412029
A_23_P142750	5610	EIF2AK2	ENST00000395127	1,457863671	11,36179841	0,000612079
A_33_P3350863	56729	RETN	ENST00000221515	1,408967208	10,61041963	0,02920101
A_23_P330561	199675	MCEMP1	ENST00000333598	1,399519712	11,0859135	0,002773045
A_23_P168229	81567	TXNDC5	ENST00000439343	1,395940305	9,052581842	0,017031044
A_23_P121716	306	ANXA3	ENST00000505805	1,388820862	9,717561326	0,006759563
A_23_P104471	51207	DUSP13	ENST00000607131	1,381007194	7,386607972	9,49E-05
A_33_P3387831	79019	CENPM	ENST00000402338	1,376817471	8,91029623	0,000736154
A_33_P3379039	100423062	IGLL5	ENST00000390321	1,376385786	13,70275686	0,080710885
A_21_P0014172	8365	HIST1H4H	NA	1,369801954	8,948446571	3,32E-05
A_24_P378019	3665	IRF7	ENST00000330243	1,356991558	13,4615153	0,006987419
A_23_P204087	4939	OAS2	ENST00000620097	1,355014977	8,683874395	0,010303292
A_24_P239076	3543	IGLL1	ENST00000412472	1,347989721	12,90200688	0,073765822
A_33_P3224858	5610	EIF2AK2	ENST00000390013	1,344769845	8,654121554	0,000199224
A_33_P3226810	8743	TNFSF10	ENST00000241261	1,343679586	12,2343938	9,46E-05
A_23_P119222	56729	RETN	ENST00000221515	1,340660682	9,327104791	0,031338097
A_33_P3743432	449491	DEFA8P	ENST00000457582	1,321632646	6,907902175	0,043900885
A_33_P3352578	338339	CLEC4D	ENST00000382064	1,311448793	8,991657128	0,000135065
A_32_P44394	9447	AIM2	ENST00000612470	1,309569562	9,536905616	0,000726332
A_24_P297539	11065	UBE2C	ENST00000617055	1,297436967	7,97006866	0,000124577
A_24_P318656	3690	ITGB3	ENST00000559488	1,290553164	9,431266288	0,022576072
A_23_P155765	3148	HMGB2	ENST00000511316	1,286050211	10,13278612	0,000594114
A_23_P81859	85235	HIST1H2AH	NA	1,270351487	11,78686371	4,61E-06
A_24_P192805	440068	CARD17	ENST00000375707	1,269236755	9,454708168	7,00E-05
A_23_P166797	64108	RTP4	ENST00000259030	1,261597473	10,08739756	0,01302563
A_23_P122863	2887	GRB10	ENST00000402497	1,259184848	8,162154216	0,003592543
A_21_P0010536	2635	GBP3	ENST00000394662	1,252038816	9,295341734	0,003098066
A_23_P41114	1475	CSTA	ENST00000264474	1,248712829	9,715712756	0,001026768
A_33_P3225512	4939	OAS2	ENST00000620097	1,24723009	8,580589045	0,021150196

A_23_P132388	9997	SCO2	ENST00000252785	1,235083503	11,48522731	0,00119466
A_23_P67847	79623	GALNT14	ENST00000349752	1,233783805	8,732436337	0,010303292
A_23_P106844	4502	MT2A	ENST00000562017	1,231127624	14,6898297	0,01070432
A_33_P3402489	4940	OAS3	ENST00000228928	1,225959896	7,100083974	0,00553533
A_23_P57379	8318	CDC45	ENST00000493724	1,219971788	7,698329858	7,00E-05
A_33_P3807062	55355	HJURP	ENST00000433484	1,210420323	7,012045163	0,000674994
A_23_P309381	723790	HIST2H2AA4	ENST00000607355	1,209614769	10,27550987	0,0003415
A_33_P3413987	710	SERPING1	ENST00000278407	1,208981961	7,721301428	0,013003359
A_23_P118246	51659	GINS2	ENST00000253462	1,205495553	7,151313181	0,000502401
A_23_P102235	6637	SNRPG	ENST00000429728	1,201966973	9,243705561	0,000307274
A_23_P93258	8358	HIST1H3B	ENST00000621411	1,200410691	10,74736778	0,000609906
A_24_P217848	8330	HIST1H2AK	ENST00000618958	1,194466972	12,14477613	4,61E-06
A_23_P23048	6280	S100A9	ENST00000368738	1,194251412	15,35079489	0,000208971
A_23_P428184	3013	HIST1H2AD	ENST00000341023	1,183346263	12,80974927	0,00019119
A_23_P152002	597	BCL2A1	ENST00000267953	1,176713685	9,28622855	0,001048109
A_33_P3211666	8809	IL18R1	ENST00000334376	1,172750073	8,550331123	0,004743598
A_24_P117410	113730	KLHDC7B	ENST00000395676	1,171929024	8,758303546	0,001372954
A_33_P3298810	2865	FFAR3	ENST00000327809	1,169627029	7,511052555	0,003716161
A_33_P3326235	3042	HBM	ENST00000472539	1,169463101	15,40658868	0,00467174
A_24_P66027	9582	APOBEC3B	ENST00000335760	1,168883936	11,72249633	0,00058366
A_33_P3342628	57801	HES4	ENST00000304952	1,167563349	10,41219697	0,062396292
A_23_P118246	51659	GINS2	ENST00000253462	1,160198037	7,069972788	0,000403025
A_33_P3263666	122416	ANKRD9	ENST00000559651	1,159596873	9,721427109	0,014970461
A_23_P42353	51513	ETV7	ENST00000339796	1,159516812	8,036372957	0,036080788
A_23_P68155	64135	IFIH1	ENST00000263642	1,150819457	10,53421358	0,007760916
A_22_P00000867	375790	AGRN	ENST00000461111	1,150772273	8,215603971	0,019953418
A_23_P48056	10970	CKAP4	ENST00000378026	1,145389489	10,13637186	5,72E-06
A_23_P74609	50486	G0S2	ENST00000367029	1,140685925	7,684468447	0,000919767
A_23_P118246	51659	GINS2	ENST00000253462	1,138186223	7,117094658	0,000269187
A_22_P00017844	54877	ZCCHC2	ENST00000585873	1,134511642	9,58487229	0,003128636
A_33_P3360216	8329	HIST1H2AI	ENST00000358739	1,131458188	7,5716605	0,000475296
A_23_P30950	116369	SLC26A8	ENST00000465492	1,130738812	7,188386869	0,000395484
A_33_P3344086	8331	HIST1H2AJ	ENST00000333151	1,129338045	12,06992584	3,53E-05
A_24_P320328	10923	SUB1	ENST00000511988	1,1269065	8,708974823	0,000504918
A_23_P206724	4493	MT1E	ENST00000306061	1,125270485	10,85477875	0,002640481
A_23_P7636	9232	PTTG1	ENST00000520452	1,120725518	9,572280554	0,001147221
A_23_P64173	114769	CARD16	ENST00000525374	1,110877183	10,32417385	8,68E-05
A_21_P0010728	2210	FCGR1B	ENST00000579737	1,1101405	10,12510072	0,008315538
A_33_P3241269	1066	CES1	ENST00000421606	1,104044195	10,84496297	0,042466203
A_23_P63789	11130	ZWINT	ENST00000489649	1,101266062	7,249829674	0,001621692
A_23_P215913	1191	CLU	ENST00000405140	1,100800032	10,02794369	0,012901704
A_32_P221799	8336	HIST1H2AM	NA	1,095333182	7,975976181	0,000120197
A_23_P60248	7295	TXN	ENST00000487892	1,093390526	11,59383933	0,000276392
A_23_P88731	5888	RAD51	ENST00000525066	1,091771042	7,170254105	0,000446292
A_23_P118246	51659	GINS2	ENST00000253462	1,091734846	7,00021907	0,000491843
A_24_P222655	712	C1QA	ENST00000374642	1,090684901	7,60948986	0,010620262
A_23_P427703	4500	MT1L	ENST00000565768	1,090608638	12,04689578	0,00973636
A_23_P69109	5359	PLSCR1	ENST00000493432	1,088765992	9,519935525	0,010149114
A_23_P59045	3012	HIST1H2AE	NA	1,085045659	10,44366857	3,80E-05
A_23_P37983	4490	MT1B	ENST00000334346	1,084962497	11,88020283	0,010830022
A_23_P152782	3430	IFI35	ENST00000538473	1,084262317	10,18850484	0,009753967
A_23_P401	1063	CENPF	ENST00000366955	1,081187812	7,479956581	0,000798876
A_23_P8185	6993	DYNLT1	ENST00000367088	1,080009916	11,66467929	1,04E-05

A_23_P29124	2812	GP1BB	ENST00000366425	1,078863545	13,06887443	0,017438088
A_23_P122197	891	CCNB1	ENST00000256442	1,078757454	7,390499281	0,001894439
A_23_P118246	51659	GINS2	ENST00000253462	1,077085061	7,027371482	0,000633018
A_33_P3340847	84674	CARD6	ENST00000254691	1,074221189	8,560455382	5,72E-06
A_23_P34788	11004	KIF2C	ENST00000372217	1,072233567	7,095247583	0,000398703
A_23_P37736	608	TNFRSF17	ENST00000053243	1,069870522	7,048942522	0,016069903
A_23_P126613	89872	AQP10	ENST00000484864	1,068156578	8,427268117	0,016654503
A_23_P30069	91351	DDX60L	ENST00000511577	1,06730446	9,938664414	0,001134028
A_23_P101992	8685	MARCO	ENST00000494979	1,066942454	9,814731575	0,015753572
A_32_P9543	200315	APOBEC3A	ENST00000402255	1,064200111	11,93678667	0,007415002
A_33_P3308105	8836	GGH	ENST00000260118	1,06125829	8,033695979	0,002547034
A_33_P3258977	338339	CLEC4D	ENST00000299665	1,05925446	8,035558984	0,000380289
A_23_P23221	1647	GADD45A	ENST00000370986	1,058363362	8,806639259	0,0006601
A_32_P105549	728113	ANXA8L1	ENST00000584982	1,053177598	7,001466573	0,003386396
A_23_P423331	84628	NTNG2	ENST00000393229	1,05271695	11,27394782	0,000401142
A_23_P63390	2210	FCGR1B	ENST00000466915	1,050811036	11,95754884	0,018709838
A_23_P59877	2171	FABP5	ENST00000524732	1,048785374	7,911537662	0,000229335
A_21_P0000197	8969	HIST1H2AG	ENST00000359193	1,048498163	8,058575385	0,001916136
A_33_P3399571	8876	VNN1	ENST00000367928	1,044430852	8,974570326	0,014585531
A_24_P208567	8809	IL18R1	ENST00000409599	1,044044422	8,553390683	0,006595939
A_23_P385861	157313	CDCA2	ENST00000521098	1,039503333	6,711530035	0,000480579
A_33_P3251876	8809	IL18R1	ENST00000410040	1,036683489	7,657533857	0,004708581
A_23_P118246	51659	GINS2	ENST00000253462	1,0362109	6,964635822	0,000605418
A_23_P118246	51659	GINS2	ENST00000253462	1,036180163	6,975300646	0,000774949
A_23_P18604	51056	LAP3	ENST00000606142	1,033533639	9,875758404	0,030267072
A_24_P38081	2289	FKBP5	ENST00000357266	1,033396665	8,574015716	0,001687678
A_24_P462899	387103	CENPW	ENST00000368325	1,031731926	7,401447951	0,000354876
A_23_P210425	10398	MYL9	ENST00000346786	1,029734091	8,196491288	0,033240106
A_23_P373119	128872	HMGB3P1	ENST00000393368	1,026260719	6,90688634	0,000674994
A_22_P00010235	4490	MT1B	ENST00000334346	1,01896751	11,93278722	0,006580235
A_23_P118246	51659	GINS2	ENST00000253462	1,014049141	6,939810224	0,000594212
A_23_P118246	51659	GINS2	ENST00000253462	1,013584048	6,947883549	0,000685132
A_33_P3323847	9401	RECQL4	ENST00000617875	1,008258446	8,248677517	0,000589802
A_33_P3212679	6637	SNRPG	ENST00000429728	1,007163205	10,05604529	0,000573467
A_23_P162300	11213	IRAK3	ENST00000261233	1,003408061	8,73722605	0,000489927
A_23_P64343	26519	TIMM10	ENST00000525158	1,001876192	9,815343629	0,001650009
A_33_P3265030	2812	GP1BB	ENST00000431044	1,000759702	14,21411351	0,021712135
A_33_P3350488	51203	NUSAP1	ENST00000414849	0,999816924	7,274650889	0,000507564
A_23_P118246	51659	GINS2	ENST00000253462	0,997102525	6,889315036	0,000633623
A_23_P120435	140686	WFDC3	ENST00000467679	0,996696748	9,497437334	0,002876881
A_23_P80032	1869	E2F1	ENST00000343380	0,992388295	6,936869113	9,98E-06
A_23_P150935	10024	TROAP	ENST00000257909	0,992289398	7,588475503	9,35E-05
A_23_P71148	644	BLVRA	ENST00000265523	0,990010588	12,18231709	0,002782685
A_24_P124992	5685	PSMA4	ENST00000557929	0,988487536	9,467078448	0,000293919
A_33_P3304983	5577	PRKAR2B	ENST00000393613	0,985876632	10,64409656	0,025418413
A_23_P106042	116173	CMTM5	ENST00000553750	0,985746777	9,27687029	0,029293369
A_23_P8640	2852	GPER1	ENST00000617001	0,983134203	7,774620181	0,008634833
A_33_P3344127	8334	HIST1H2AC	ENST00000602637	0,979983142	9,261024796	3,32E-05
A_23_P50108	10403	NDC80	ENST00000261597	0,979928058	7,917834364	8,11E-05
A_23_P140146	83982	IFI27L2	ENST00000554909	0,970341359	10,59357459	1,16E-05
A_33_P3260605	8727	CTNNAL1	ENST00000374593	0,970074706	7,865160798	0,005470224
A_23_P64372	6947	TCN1	ENST00000257264	0,968012229	8,278273872	0,081193887
A_23_P74115	8438	RAD54L	ENST00000371975	0,967831808	6,74955475	0,000134749

A_23_P72737	8519	IFITM1	ENST00000408968	0,967310048	15,83541154	0,005128208
A_23_P80032	1869	E2F1	ENST00000343380	0,96721882	6,937435833	1,06E-05
A_24_P399888	79019	CENPM	ENST00000396437	0,967088797	6,698349573	0,000692526
A_23_P80032	1869	E2F1	ENST00000343380	0,965417851	6,928828748	8,83E-06
A_23_P31671	7381	UQCRB	ENST00000521948	0,964054339	9,440285845	0,016496767
A_23_P80032	1869	E2F1	ENST00000343380	0,963948368	6,931095144	1,06E-05
A_23_P327140	57674	RNF213	ENST00000560083	0,963614138	8,43507391	0,004487548
A_23_P38346	79132	DHX58	ENST00000251642	0,963526509	10,76833193	0,046973976
A_23_P25155	53831	GPR84	ENST00000551809	0,96319063	7,314420654	0,002517419
A_21_P0012079	541471	MIR4435-1HG	ENST00000409569	0,961752259	11,71806482	0,000198132
A_33_P3392077	9540	TP53I3	ENST00000238721	0,960131427	8,959956034	0,0008398
A_33_P3263170	6637	SNRPG	ENST00000429728	0,958137586	10,4532688	0,001212779
A_33_P3286278	2896	GRN	ENST00000053867	0,955618765	16,01167882	0,001146714
A_32_P54553	373856	USP41	ENST00000454608	0,955392646	6,771502533	0,015036589
A_33_P3315314	645745	MT1HL1	ENST00000464121	0,953513977	11,13450684	0,012031299
A_33_P3423941	8519	IFITM1	ENST00000408968	0,950568508	14,71027679	0,007027954
A_23_P138507	983	CDK1	ENST00000448257	0,945172744	6,63244864	0,00134685
A_33_P3413597	4709	NDUFB3	ENST00000433898	0,944717775	10,49152261	0,000209418
A_23_P10385	51514	DTL	ENST00000475419	0,944130261	6,632551678	0,000173477
A_23_P255104	10184	LHFPL2	ENST00000380345	0,939664422	8,526245883	0,000390951
A_23_P32500	23166	STAB1	ENST00000462741	0,939481855	8,508198126	0,001205332
A_23_P80032	1869	E2F1	ENST00000343380	0,93692921	6,899072771	1,68E-05
A_23_P17130	84281	C2orf88	ENST00000450357	0,936470275	8,601568704	0,047530917
A_23_P124417	699	BUB1	ENST00000302759	0,936449181	6,648077397	0,000909602
A_23_P83278	51510	CHMP5	ENST00000223500	0,936018685	9,401968351	0,000758487
A_23_P159650	1349	COX7B	ENST00000373335	0,93517371	9,373864615	0,013908102
A_23_P105012	54979	HRASLS2	ENST00000255695	0,927349558	6,860956257	0,008335414
A_23_P66241	4499	MT1M	ENST00000379818	0,92703462	7,223427083	8,49E-05
A_33_P3397599	79168	LILRA6	ENST00000430421	0,925075223	12,02746267	0,001847953
A_22_P00010386	140465	MYL6B	ENST00000548571	0,924255825	8,191373684	8,09E-05
A_23_P71727	1164	CKS2	ENST00000314355	0,92380329	8,078438708	0,001857714
A_23_P25735	5687	PSMA6	ENST00000554541	0,922582041	11,13318594	0,000729464
A_23_P358944	5371	PML	ENST00000435786	0,920165266	11,47149533	0,024902512
A_33_P3363420	257019	FRMD3	ENST00000621208	0,919003132	8,135520099	0,005078031
A_33_P3357609	81030	ZBP1	ENST00000480037	0,917095491	7,803161688	0,009962254
A_24_P48539	8778	SIGLEC5	ENST00000570106	0,917033826	9,19060454	0,002059045
A_23_P28334	8807	IL18RAP	ENST00000264260	0,915827429	11,81630088	0,020368484
A_23_P40611	6948	TCN2	ENST00000471659	0,912540958	6,970065643	0,005735204
A_23_P202978	834	CASP1	ENST00000534497	0,910761858	11,34031736	0,000472212
A_24_P382319	634	CEACAM1	ENST00000403444	0,910674477	10,26174308	0,01599546
A_33_P3362915	2993	GYPA	ENST00000513677	0,910182953	8,130839093	0,076968581
A_23_P80032	1869	E2F1	ENST00000343380	0,909827345	6,94775734	1,74E-05
A_33_P3296303	51510	CHMP5	ENST00000487080	0,907241709	8,972480854	0,000390951
A_23_P80032	1869	E2F1	ENST00000343380	0,906884036	6,847107304	8,01E-06
A_24_P82880	7171	TPM4	ENST00000586193	0,906778589	10,04701886	7,99E-06
A_23_P81212	51023	MRPS18C	ENST00000514581	0,90433338	9,411629638	0,000225066
A_23_P124190	53840	TRIM34	ENST00000491385	0,903213271	8,200674555	1,45E-05
A_23_P121253	8743	TNFSF10	ENST00000241261	0,903153646	12,29400752	0,005709227
A_23_P89509	10615	SPAG5	ENST00000580676	0,900886157	7,692683842	0,002416361
A_23_P138507	983	CDK1	ENST00000448257	0,898281193	6,578050318	0,00102887
A_32_P162187	717	C2	ENST00000299367	0,897479093	7,677231747	0,008640015
A_24_P30194	24138	IFIT5	ENST00000371795	0,896063874	9,850444801	0,028897516
A_23_P138507	983	CDK1	ENST00000448257	0,895891097	6,606963012	0,001718903

A_21_P0011496	1066	CES1	ENST00000566467	0,89225468	8,019993546	0,046256301
A_23_P427760	143689	PIWIL4	ENST00000446230	0,891889581	7,713649274	0,0026746
A_21_P0000015	83666	PARP9	NA	0,890153862	9,91664535	0,018954491
A_23_P359540	8361	HIST1H4F	ENST00000244537	0,889828599	8,215583157	8,10E-06
A_23_P138507	983	CDK1	ENST00000448257	0,889657541	6,563528034	0,001016607
A_23_P68610	22974	TPX2	ENST00000340513	0,888723099	6,790073808	0,000345812
A_23_P111041	8346	HIST1H2BI	ENST00000377733	0,88870978	10,67761233	6,19E-05
A_23_P41470	55601	DDX60	ENST00000393743	0,888543997	8,257613578	0,027849845
A_23_P10385	51514	DTL	ENST00000475419	0,887854709	6,596221468	0,000404875
A_24_P941912	151636	DTX3L	ENST00000296161	0,887335605	9,501131899	0,008364032
A_23_P252322	514	ATP5E	ENST00000395659	0,885035168	14,9692158	2,41E-05
A_23_P254852	9381	OTOF	ENST00000272371	0,884275636	11,15143817	0,011027299
A_23_P80032	1869	E2F1	ENST00000343380	0,884274482	6,898688012	2,69E-05
A_23_P62890	2633	GBP1	ENST00000370473	0,884248632	8,723410968	0,04958668
A_24_P161018	54625	PARP14	ENST00000475640	0,88336484	10,94855057	0,012407515
A_24_P302998	521	ATP5I	ENST00000515202	0,882525943	10,95753951	0,000546257
A_33_P3220663	246329	STAC3	ENST00000557176	0,882117212	9,302181343	0,000711739
A_23_P117582	122953	JDP2	ENST00000267569	0,88201781	10,91012401	0,000240236
A_23_P10385	51514	DTL	ENST00000475419	0,881420301	6,658483214	0,000674994
A_33_P3252394	10912	GADD45G	ENST00000252506	0,880620501	8,251307503	8,09E-05
A_24_P169148	3146	HMGB1	ENST00000522557	0,879677362	10,7601279	0,000298008
A_23_P121602	8819	SAP30	ENST00000296504	0,878487879	8,744195893	0,005481268
A_23_P86653	5552	SRGN	ENST00000462445	0,877293004	13,97589165	0,002036829
A_21_P0002006	104326052	NRIR	NA	0,877186296	7,164374582	0,00328737
A_23_P121602	8819	SAP30	ENST00000296504	0,874623837	8,81172919	0,006149436
A_33_P3246833	3557	IL1RN	ENST00000259206	0,87218573	13,55820628	0,021400171
A_23_P370989	4173	MCM4	ENST00000518382	0,870416151	7,096338877	0,000979782
A_33_P3374210	4288	MKI67	ENST00000617118	0,869090756	7,40363139	0,000157236
A_21_P0000671	4493	MT1E	ENST00000563395	0,86855366	9,035608605	4,06E-05
A_23_P145874	219285	SAMD9L	ENST00000411955	0,868325098	11,21876359	0,031037305
A_23_P201711	6277	S100A6	ENST00000462776	0,866860312	14,83095254	4,45E-05
A_33_P3417695	440836	ODF3B	ENST00000329363	0,863216944	10,97228078	0,002219444
A_23_P80032	1869	E2F1	ENST00000343380	0,862880028	6,920814462	5,93E-05
A_23_P117582	122953	JDP2	ENST00000267569	0,862331028	10,95052184	0,000389706
A_23_P137016	6303	SAT1	ENST00000474223	0,861727922	13,25185603	0,000631527
A_33_P3397443	9088	PKMYT1	ENST00000574680	0,86037007	9,574541676	0,001268083
A_23_P121602	8819	SAP30	ENST00000296504	0,859083781	8,814903549	0,006710852
A_23_P213718	27089	UQCRQ	ENST00000378665	0,858117546	11,86860488	0,004752607
A_23_P121602	8819	SAP30	ENST00000296504	0,856133283	8,79124743	0,007736235
A_23_P39840	10791	VAMP5	ENST00000306384	0,855722195	9,868286975	0,001895923
A_33_P3372910	23586	DDX58	NA	0,855452231	9,760557886	0,037509646
A_23_P51085	57405	SPC25	ENST00000611144	0,854824204	6,493503462	0,000690539
A_33_P3267799	11006	LILRB4	ENST00000617098	0,85435814	9,409905353	0,012040427
A_21_P0000508	100170227	SNAR-D	NA	0,853256511	8,19451501	0,053470535
A_33_P3290909	8243	SMC1A	ENST00000375340	0,851281242	9,195942931	9,06E-06
A_23_P138507	983	CDK1	ENST00000448257	0,850074042	6,547153643	0,001440755
A_23_P52017	259266	ASPM	ENST00000367408	0,849610525	6,772929569	0,005606171
A_23_P121602	8819	SAP30	ENST00000296504	0,849452159	8,733324895	0,007480662
A_23_P98382	26521	TIMM8B	ENST00000507614	0,848758057	10,37346682	0,005980818
A_24_P3783	8342	HIST1H2BM	ENST00000621112	0,848010661	10,64959098	8,33E-05
A_23_P121602	8819	SAP30	ENST00000296504	0,846932135	8,990494228	0,008387318
A_23_P208880	29128	UHRF1	ENST00000624301	0,846794964	6,767599469	0,002577057
A_23_P130194	5831	PYCR1	ENST00000619204	0,845909954	7,470786941	0,003650378

A_23_P81690	1347	COX7A2	ENST00000459637	0,844879074	12,45162328	0,000240995
A_23_P8913	760	CA2	ENST00000285379	0,84388709	9,856361095	0,026868037
A_23_P10385	51514	DTL	ENST00000475419	0,841315701	6,58557745	0,000620991
A_23_P117582	122953	JDP2	ENST00000267569	0,840686587	11,0278338	0,000380289
A_23_P145965	8460	TPST1	ENST00000304842	0,838302512	8,02112167	0,067899224
A_23_P10385	51514	DTL	ENST00000475419	0,837203082	6,541501492	0,000547453
A_33_P3402526	55454	CSGALNACT2	ENST00000538229	0,837020687	9,196745384	4,55E-05
A_23_P5392	9540	TP53I3	ENST00000238721	0,83590719	8,212946275	0,001478818
A_23_P170186	26873	OPLAH	ENST00000618853	0,835273434	8,769914397	0,004143182
A_24_P33982	284021	MILR1	ENST00000612535	0,833741933	8,549559186	0,000788082
A_33_P3269678	541471	MIR4435-1HG	ENST00000409054	0,833275667	10,25558506	8,75E-05
A_24_P274270	6772	STAT1	ENST00000452281	0,831718839	11,07695317	0,023027511
A_23_P59069	8348	HIST1H2BO	NA	0,830914324	10,91734339	4,35E-05
A_23_P122233	29093	MRPL22	ENST00000519059	0,830877036	8,59333517	0,000298008
A_23_P121602	8819	SAP30	ENST00000296504	0,830713259	8,75363692	0,00903055
A_23_P206059	9055	PRC1	ENST00000361188	0,829836774	7,573307302	0,004799689
A_23_P48669	1033	CDKN3	ENST00000555837	0,829572567	6,656275071	0,001234902
A_32_P199301	7027	TFDP1	ENST00000544902	0,829110992	8,107295384	0,004596697
A_23_P10385	51514	DTL	ENST00000475419	0,829108594	6,533004605	0,000638107
A_23_P138507	983	CDK1	ENST00000448257	0,828841756	6,490540686	0,001264872
A_23_P59547	51251	NT5C3A	ENST00000242210	0,828647717	10,77778451	0,001424593
A_23_P395374	8360	HIST1H4D	NA	0,828346883	9,65912309	0,006776524
A_32_P205553	51121	RPL26L1	ENST00000602923	0,827214127	8,669692998	0,000666963
A_23_P258493	4001	LMNB1	ENST00000460265	0,826353464	8,723405029	0,001304445
A_23_P111701	2791	GNG11	ENST00000248564	0,825467248	10,19787063	0,036400735
A_23_P80032	1869	E2F1	ENST00000343380	0,825385736	6,861591591	3,32E-05
A_23_P159833	4694	NDUFA1	ENST00000371437	0,825129263	11,37560591	0,00048109
A_23_P117582	122953	JDP2	ENST00000267569	0,824944456	11,15516881	0,000531613
A_23_P117582	122953	JDP2	ENST00000267569	0,824333112	10,93132273	0,000458415
A_33_P3221960	8807	IL18RAP	ENST00000264260	0,823650402	11,43111971	0,022655239
A_24_P941167	80830	APOL6	ENST00000409652	0,822521247	11,02646958	0,024356954
A_23_P126803	10092	ARPC5	ENST00000294742	0,822257668	14,01377454	3,53E-05
A_24_P295245	444	ASPH	ENST00000517928	0,821939661	8,574343663	0,011946181
A_23_P121602	8819	SAP30	ENST00000296504	0,821422328	8,827883506	0,011988037
A_23_P208866	9535	GMFG	ENST00000601731	0,820713344	14,74689781	8,96E-05
A_23_P10385	51514	DTL	ENST00000475419	0,820533837	6,515731587	0,000547453
A_23_P143190	4605	MYBL2	ENST00000396863	0,818517274	7,143413914	0,000398703
A_23_P85903	7100	TLR5	ENST00000366881	0,816988627	8,975363915	0,013291139
A_23_P70007	3161	HMMR	ENST00000358715	0,815181791	6,623157732	0,002090163
A_23_P10385	51514	DTL	ENST00000475419	0,814978795	6,572990252	0,000722947
A_23_P122233	29093	MRPL22	ENST00000519059	0,814937226	8,412096839	0,000144162
A_23_P154832	522	ATP5J	ENST00000400093	0,814704371	10,64881223	0,000766652
A_23_P161190	7431	VIM	ENST00000544301	0,814695838	15,17845738	9,46E-05
A_32_P35512	6741	SSB	ENST00000420252	0,813448486	9,426315699	3,32E-05
A_23_P19712	51053	GMNN	ENST00000620958	0,812903907	7,782614053	0,003868776
A_33_P3264846	219285	SAMD9L	ENST00000610760	0,812122478	9,411222059	0,028289518
A_24_P223384	8335	HIST1H2AB	ENST00000615868	0,811781027	7,60129077	0,000316681
A_23_P138507	983	CDK1	ENST00000448257	0,811753566	6,539402247	0,000758481
A_23_P58321	890	CCNA2	ENST00000618014	0,811630057	6,601773774	0,00082067
A_24_P150486	9517	SPTLC2	ENST00000216484	0,811544591	8,213250419	0,001741364
A_23_P121602	8819	SAP30	ENST00000296504	0,811501388	8,917776391	0,009391487
A_23_P111701	2791	GNG11	ENST00000248564	0,808963876	9,980200502	0,040558252
A_23_P117582	122953	JDP2	ENST00000267569	0,808406276	11,05782151	0,000896437

A_23_P56559	10170	DHRS9	ENST00000428522	0,80819634	9,970640588	0,021319304
A_33_P3271594	57159	TRIM54	ENST00000380075	0,807876868	7,688311048	0,002937838
A_24_P158946	121512	FGD4	ENST00000395740	0,806335943	9,66681668	6,85E-05
A_23_P363174	8332	HIST1H2AL	NA	0,806166808	6,773236725	0,0004699
A_24_P23245	4700	NDUFA6	ENST00000605927	0,801817092	9,911365111	0,000504365
A_23_P122233	29093	MRPL22	ENST00000519059	0,801583729	8,800911163	0,000445164
A_23_P308722	92749	DRC1	ENST00000288710	0,801517551	6,940122754	0,000704011
A_23_P111701	2791	GNG11	ENST00000248564	0,800777211	10,08084787	0,047917796
A_23_P122233	29093	MRPL22	ENST00000519059	0,800511517	8,624509858	0,000717031
A_33_P3265359	55502	HES6	ENST00000409160	0,800511208	9,125816538	0,011891248
A_33_P3244931	1622	DBI	ENST00000409094	0,799593249	10,81090311	0,002311562
A_33_P3284933	246778	IL27	ENST00000356897	0,799191943	7,689008822	0,008991569
A_23_P138507	983	CDK1	ENST00000448257	0,798348726	6,518172913	0,002143338
A_23_P111701	2791	GNG11	ENST00000248564	0,796868031	10,09060465	0,046647263
A_23_P111701	2791	GNG11	ENST00000248564	0,796742081	10,12967191	0,039604435
A_24_P172481	10346	TRIM22	ENST00000379965	0,795992953	10,75717784	0,018895912
A_23_P351275	7378	UPP1	ENST00000416681	0,795270963	9,311162396	0,000752586
A_24_P226008	11343	MGLL	ENST00000476682	0,795077328	9,76244747	0,0026746
A_23_P10385	51514	DTL	ENST00000475419	0,794355595	6,612164875	0,001008059
A_24_P29001	27258	LSM3	ENST00000306024	0,794197253	9,21091236	0,007061467
A_23_P132956	7345	UCHL1	ENST00000472501	0,79326161	6,44091581	0,022923282
A_23_P111701	2791	GNG11	ENST00000248564	0,791949606	9,986751154	0,038420032
A_23_P212475	51246	SHISA5	ENST00000417962	0,791225879	15,29920341	0,014523565
A_24_P348265	2204	FCAR	ENST00000614052	0,790698407	8,495774324	0,001243103
A_21_P0012993	4671	NAIP	ENST00000447012	0,790018106	9,676193742	0,026199946
A_33_P3400477	6491	STIL	ENST00000418131	0,789917876	7,150849851	0,000107016
A_23_P137470	57568	SIPA1L2	ENST00000308942	0,788290561	8,466572166	0,021196496
A_24_P370172	353514	LILRA5	ENST00000446712	0,787586686	9,234511145	0,004988345
A_23_P10385	51514	DTL	ENST00000475419	0,787317236	6,558790245	0,000767966
A_23_P200001	91624	NEXN	ENST00000480732	0,7871919	7,671460037	0,0071864
A_33_P3225522	4939	OAS2	ENST00000449768	0,78690863	9,488432072	0,070291748
A_33_P3229918	171558	PTCRA	ENST00000616441	0,782912399	10,55881039	0,057864871
A_33_P3393836	51251	NT5C3A	ENST00000409467	0,782072986	9,298186737	0,00195636
A_33_P3363425	257019	FRMD3	ENST00000621208	0,780679257	7,911537534	0,012097184
A_33_P3273436	130589	GALM	ENST00000272252	0,780549772	8,043888949	0,001174098
A_23_P122233	29093	MRPL22	ENST00000519059	0,780043047	8,479593301	0,000460317
A_33_P3395008	55289	ACOXL	ENST00000439055	0,778445424	6,925574281	0,000101233
A_23_P111701	2791	GNG11	ENST00000248564	0,778124377	10,08612485	0,048616098
A_33_P3398448	84875	PARP10	ENST00000526007	0,778068942	12,02193661	0,025356578
A_33_P3854217	79932	KIAA0319L	ENST00000325722	0,777960326	9,218029654	0,004043783
A_23_P254733	79682	CENPU	ENST00000502461	0,777579874	6,876931622	0,005327183
A_23_P206396	51192	CKLF	ENST00000527845	0,77454339	11,36935689	0,000814043
A_23_P167997	8339	HIST1H2BG	ENST00000541790	0,773218453	9,207230108	8,92E-05
A_23_P160025	3428	IFI16	ENST00000562225	0,772678	10,26515684	0,009093213
A_33_P3287879	8357	HIST1H3H	ENST00000369163	0,77228147	10,16559768	0,000511984
A_23_P16523	9518	GDF15	ENST00000252809	0,772124063	7,147096244	0,09700003
A_32_P25253	81689	ISCA1	ENST00000426679	0,771342152	9,314518403	0,053671447
A_23_P121602	8819	SAP30	ENST00000296504	0,77115313	8,833825084	0,015175007
A_23_P372834	358	AQP1	ENST00000311813	0,770618893	8,570907928	0,081119828
A_23_P58321	890	CCNA2	ENST00000618014	0,767651785	6,617844326	0,00094064
A_23_P145761	10124	ARL4A	ENST00000404894	0,76618592	8,141235236	0,005631934
A_24_P65373	3674	ITGA2B	ENST00000262407	0,765491922	8,310648515	0,09105853
A_23_P379614	11339	OIP5	ENST00000220514	0,764974291	6,750225955	0,007026131

A_33_P3245824	80148	PQLC1	ENST00000466449	0,764186917	11,52126113	0,012932163
A_24_P212481	79772	MCTP1	ENST00000505208	0,76287105	8,091254411	0,000557544
A_24_P235429	19	ABCA1	ENST00000374736	0,762266737	8,248423208	0,001228104
A_23_P78092	2123	EVI2A	ENST00000578021	0,76159306	10,27193685	0,019840483
A_23_P122233	29093	MRPL22	ENST00000519059	0,761414608	8,596836984	0,000909817
A_33_P3268507	634	CEACAM1	ENST00000403136	0,761268839	7,271660156	0,01008309
A_23_P14174	10673	TNFSF13B	ENST00000486502	0,75825092	11,90407519	0,035303753
A_23_P117582	122953	JDP2	ENST00000267569	0,757938891	11,01187811	0,001077456
A_23_P122233	29093	MRPL22	ENST00000519059	0,757870397	8,572365268	0,000532911
A_33_P3242863	56953	NT5M	ENST00000616989	0,75785478	10,67949165	0,035782407
A_33_P3371718	6303	SAT1	ENST00000474223	0,75766747	12,59958948	0,001092547
A_23_P82748	56943	ENY2	ENST00000517350	0,756631771	10,0445011	0,000555927
A_23_P28886	5111	PCNA	ENST00000379160	0,755922813	9,395074971	0,00190833
A_23_P111701	2791	GNG11	ENST00000248564	0,755803795	10,15113465	0,052028471
A_23_P107644	6632	SNRPD1	ENST00000300413	0,755275705	9,35383609	0,003111545
A_21_P0000507	100170217	SNAR-B2	NA	0,753949073	8,67602749	0,064997873
A_24_P141214	2040	STOM	ENST00000286713	0,753145781	10,85526259	0,038160651
A_33_P3406072	257019	FRMD3	ENST00000304195	0,753061873	6,960599167	0,000445231
A_23_P408094	4084	MXD1	ENST00000410000	0,753010421	11,97797938	0,006424251
A_23_P111054	3018	HIST1H2BB	ENST00000615966	0,752631477	9,478094065	0,000198132
A_23_P30813	8362	HIST1H4K	NA	0,751951376	9,225839487	0,000753296
A_24_P16124	340198	IFITM4P	ENST00000414714	0,751878649	15,19124479	0,029086283
A_23_P111701	2791	GNG11	ENST00000248564	0,751742646	10,0872036	0,055783019
A_23_P259506	84418	CYSTM1	ENST00000261811	0,751180528	11,61308531	0,02521049
A_23_P29005	64092	SAMSN1	ENST00000619120	0,750659044	8,822612267	0,00410102
A_24_P283288	1432	MAPK14	ENST00000474429	0,750484254	9,200410031	0,002409572
A_33_P3383029	4601	MXI1	ENST00000369612	0,75041734	11,58510464	0,080866089
A_23_P19482	23564	DDAH2	ENST00000375789	0,749750873	9,375926583	0,000398703
A_23_P67971	130589	GALM	ENST00000272252	0,749726608	8,867383578	0,003093374
A_23_P131024	27033	ZBTB32	ENST00000392197	0,748736976	7,441501845	0,011896211
A_23_P58321	890	CCNA2	ENST00000618014	0,748166209	6,515543865	0,000555927
A_23_P18579	10744	PTTG2	ENST00000504686	0,747923234	8,336105277	0,000316806
A_24_P927716	643036	SLED1	ENST00000506741	0,747182502	8,62353626	0,020213326
A_23_P117582	122953	JDP2	ENST00000267569	0,747123304	10,9843242	0,001298578
A_23_P165402	51639	SF3B6	ENST00000233468	0,745389051	9,430790727	0,001138991
A_24_P342096	100132948	FAM27C	ENST00000377542	0,742770317	7,953778435	0,002537392
A_33_P3370094	4311	MME	ENST00000360490	0,742086224	10,06878629	0,018478969
A_33_P3362641	493856	CISD2	ENST00000273986	0,741688042	8,109498844	0,031226607
A_21_P0010727	2209	FCGR1A	ENST00000489479	0,738677048	8,120293211	0,060101672
A_23_P138507	983	CDK1	ENST00000448257	0,738363669	6,509064248	0,003995721
A_23_P124164	57461	ISY1	ENST00000485703	0,738052008	8,127342802	7,21E-07
A_23_P133691	58528	RRAGD	ENST00000369415	0,736548561	7,930253232	0,000909817
A_23_P138507	983	CDK1	ENST00000448257	0,735977097	6,491319236	0,00335106
A_24_P183128	51316	PLAC8	ENST00000505406	0,735238026	12,04035582	0,033679581
A_33_P3256347	26521	TIMM8B	ENST00000541231	0,734652275	9,835089899	0,008729681
A_23_P122233	29093	MRPL22	ENST00000519059	0,734536554	8,432849885	0,000883171
A_33_P3258612	5111	PCNA	ENST00000379160	0,734481472	8,640182322	0,00248955
A_33_P3242543	4128	MAOA	ENST00000338702	0,733911403	6,675558763	0,086223798
A_23_P97064	26270	FBXO6	ENST00000449067	0,733600375	8,21679174	0,012424972
A_24_P56130	4637	MYL6	ENST00000550639	0,733521847	13,46645447	0,003415439
A_33_P3407880	118932	ANKRD22	NA	0,732085148	6,736743561	0,005219389
A_23_P6535	113730	KLHDC7B	ENST00000395676	0,731053914	8,709552804	0,017480519
A_23_P126278	1118	CHIT1	ENST00000484834	0,729660977	6,632182156	0,043989585

A_23_P115482	29089	UBE2T	ENST00000367274	0,729408037	7,379667556	0,005933718
A_23_P100127	57082	CASC5	ENST00000527044	0,729276914	6,727264409	0,001469869
A_33_P3302632	8344	HIST1H2BE	NA	0,728669291	7,249291213	0,00089436
A_23_P117582	122953	JDP2	ENST00000267569	0,728133887	11,23461334	0,001633635
A_32_P1173	115004	MB21D1	ENST00000370318	0,728016948	9,054888346	3,94E-05
A_23_P111701	2791	GNG11	ENST00000248564	0,727997401	10,22766515	0,061003559
A_33_P3315779	55008	HERC6	ENST00000273960	0,727608006	7,449355495	0,053030506
A_24_P217834	8351	HIST1H3D	ENST00000356476	0,727120655	12,16974498	0,001264209
A_23_P118061	51192	CKLF	ENST00000526149	0,726941553	11,30480017	0,001370419
A_33_P3404588	121512	FGD4	ENST00000395740	0,726246355	8,402391742	0,000516619
A_23_P145238	85236	HIST1H2BK	NA	0,725955755	11,7397874	0,003988467
A_33_P3268555	11262	SP140	ENST00000544128	0,725573429	8,386086349	0,006763573
A_23_P57588	51512	GTSE1	ENST00000491863	0,724963564	6,574167051	0,000341054
A_33_P3228612	777	CACNA1E	ENST00000367570	0,72398908	7,043526075	0,01609604
A_32_P204676	2171	FABP5	ENST00000486269	0,723716615	7,190137068	0,000997645
A_23_P381261	196883	ADCY4	ENST00000554781	0,722818427	8,716306249	0,000340883
A_23_P69908	2745	GLRX	ENST00000507412	0,722565452	11,88095155	0,001516637
A_33_P3404601	717	C2	ENST00000460487	0,722416223	6,584125616	0,013922413
A_23_P366216	8345	HIST1H2BH	NA	0,722275777	11,2707042	0,0003415
A_23_P121064	5806	PTX3	ENST00000295927	0,721118177	7,115870366	0,061929152
A_33_P3231472	27348	TOR1B	ENST00000486372	0,719176353	8,804149649	0,01453554
A_33_P3396370	84519	ACRBP	ENST00000229243	0,718865193	11,46210389	0,042500205
A_33_P3879161	118788	PIK3AP1	ENST00000371109	0,718387907	8,365179333	0,002887609
A_23_P154235	9111	NMI	ENST00000243346	0,718276185	10,27182867	0,002420242
A_33_P3310780	2017	CTTN	ENST00000301843	0,718168781	8,818581533	0,089500981
A_33_P3323939	643418	LIPN	ENST00000404459	0,717575096	8,058491705	0,002416361
A_23_P356526	85363	TRIM5	ENST00000465634	0,717528765	7,076018312	0,002199293
A_23_P353035	3490	IGFBP7	ENST00000295666	0,716016554	9,974491691	0,003121513
A_23_P54477	55505	NOP10	ENST00000328848	0,715160363	12,98375606	2,41E-05
A_33_P3374205	4288	MKI67	ENST00000617118	0,715091607	6,835921601	0,00094064
A_23_P30799	8968	HIST1H3F	ENST00000618052	0,714788055	10,76520403	0,001218243
A_23_P14734	51065	RPS27L	ENST00000482846	0,714716833	9,643117033	0,022137437
A_24_P235266	2887	GRB10	ENST00000403097	0,714486435	7,740045747	0,040548678
A_23_P87545	10410	IFITM3	ENST00000531688	0,714447071	16,30996576	0,026199946
A_23_P122233	29093	MRPL22	ENST00000519059	0,713383454	8,426342174	0,000789695
A_24_P146211	3017	HIST1H2BD	ENST00000377777	0,712423718	10,120699	0,000206684
A_33_P3213772	23380	SRGAP2	ENST00000604247	0,711891027	10,65103732	0,015009567
A_23_P154235	9111	NMI	ENST00000243346	0,711704226	10,17099994	0,002371335
A_24_P380536	8763	CD164	ENST00000506649	0,711509345	10,81143962	9,72E-05
A_23_P154235	9111	NMI	ENST00000243346	0,711014285	10,2477075	0,001847953
A_23_P46369	5872	RAB13	ENST00000484297	0,710005512	6,732737546	0,005153275
A_22_P00024462	101929693	RARA-AS1	NA	0,709178332	7,560943777	0,000487471
A_24_P673063	2171	FABP5	ENST00000437960	0,708764773	7,007467168	0,000685132
A_23_P7827	441168	FAM26F	ENST00000368605	0,708430476	9,839367621	0,089049363
A_24_P68631	317772	HIST2H2AB	ENST00000331128	0,707872989	12,75073747	0,001412029
A_23_P154235	9111	NMI	ENST00000243346	0,707637929	10,41078803	0,002794139
A_23_P207445	5608	MAP2K6	ENST00000592348	0,707588414	8,712609432	0,000269187
A_23_P200507	29097	CNIH4	ENST00000366860	0,707263727	10,3210117	0,00075452
A_23_P151150	2305	FOXM1	ENST00000359843	0,707247465	6,49986303	0,000532911
A_24_P270144	967	CD63	ENST00000551173	0,706214239	13,27995673	0,000499737
A_23_P58321	890	CCNA2	ENST00000618014	0,705115235	6,537061394	0,001249592
A_23_P122233	29093	MRPL22	ENST00000519059	0,704853262	8,39884251	0,000790579
A_33_P3395605	338773	TMEM119	ENST00000392806	0,704797376	6,652563126	0,004593684

A_23_P145777	4697	NDUFA4	ENST00000482299	0,704726324	11,54432307	0,026368115
A_32_P95729	55215	FANCI	ENST00000566615	0,704684925	7,584481643	0,000774756
A_33_P3372705	647135	SRGAP2B	ENST00000304465	0,702031183	10,32508645	0,010179035
A_24_P385611	6672	SP100	ENST00000462751	0,699772667	10,34165134	0,000573872
A_23_P85693	2634	GBP2	ENST00000463660	0,698386508	11,31355952	0,006217351
A_24_P175187	54809	SAMD9	ENST00000379958	0,698185791	7,981173102	0,003155336
A_23_P69383	83666	PARP9	ENST00000489652	0,698081844	9,430166847	0,037754786
A_23_P157795	8727	CTNNAL1	ENST00000374594	0,697551975	8,252003924	0,079375113
A_23_P121064	5806	PTX3	ENST00000295927	0,697182073	7,080477402	0,074948325
A_33_P3229032	6320	CLEC11A	ENST00000250340	0,69702027	9,237137478	0,072936334
A_24_P55148	8970	HIST1H2BJ	ENST00000339812	0,696944063	8,769982514	0,000125493
A_21_P0000511	100170221	SNAR-H	NA	0,696608402	7,723675888	0,087622173
A_33_P3389230	2833	CXCR3	ENST00000373691	0,695709639	8,338662482	0,023142116
A_24_P227091	3832	KIF11	ENST00000260731	0,695475719	6,791405034	0,006902849
A_32_P199252	3320	HSP90AA1	ENST00000530115	0,694786262	9,329264509	0,001234902
A_23_P87709	79887	PLBD1	ENST00000240617	0,694347129	13,30001417	0,019198304
A_23_P157879	2219	FCN1	ENST00000371806	0,69349357	15,70811196	0,019144647
A_33_P3404989	8357	HIST1H3H	ENST00000369163	0,693221582	7,931741183	0,007176246
A_23_P256821	1378	CR1	ENST00000400960	0,693028666	8,12964035	0,014592138
A_23_P128991	81892	SLIRP	ENST00000556956	0,692576952	9,293043818	0,002474791
A_23_P154235	9111	NMI	ENST00000243346	0,690709341	10,34098254	0,003121063
A_23_P117582	122953	JDP2	ENST00000267569	0,689172151	11,0836069	0,002929242
A_23_P214627	199	AIF1	ENST00000459762	0,688765106	13,60887512	0,003980394
A_33_P3229083	85236	HIST1H2BK	ENST00000356950	0,687437815	10,10168504	0,000475296
A_23_P338479	29126	CD274	ENST00000381577	0,686528435	7,19041321	0,034523206
A_33_P3351851	8368	HIST1H4L	ENST00000618305	0,686003789	7,030637989	0,000383538
A_21_P0006788	100505869	CCDC147-AS1	NA	0,685534446	6,930111757	0,001400177
A_24_P328504	11262	SP140	ENST00000476126	0,685471625	10,52976567	0,007950038
A_23_P112260	2790	GNG10	ENST00000374293	0,685305715	10,05694195	0,065128062
A_23_P128470	160364	CLEC12A	ENST00000396507	0,685298469	8,336756528	0,08582277
A_23_P98744	119774	OR52K2	ENST00000325719	0,685160248	8,14594449	0,014069384
A_23_P317056	4541	ND6	ENST00000361681	0,684631791	9,404578826	0,016192181
A_23_P88740	55839	CENPN	ENST00000299572	0,684510762	6,825934237	0,001260201
A_23_P94533	1514	CTSL	ENST00000375894	0,684225883	8,693333369	0,020609476
A_23_P140301	5684	PSMA3	ENST00000557087	0,683173676	9,842823067	0,000901169
A_23_P15414	8578	SCARF1	ENST00000576012	0,682598541	8,976714192	0,039832363
A_23_P208009	90701	SEC11C	ENST00000299714	0,682161813	10,27900988	0,058719599
A_23_P121064	5806	PTX3	ENST00000295927	0,680843015	7,155630389	0,080986229
A_33_P3246318	9978	RBX1	ENST00000216225	0,679411437	11,41368442	0,007164302
A_23_P149200	991	CDC20	ENST00000372462	0,678673391	7,326874833	0,002123155
A_23_P99253	8825	LIN7A	ENST00000261203	0,677858837	8,516698763	0,009332778
A_33_P3246829	3557	IL1RN	ENST00000409930	0,677116037	8,043330713	0,016222086
A_23_P206733	1066	CES1	ENST00000569260	0,676920315	8,333744687	0,011946581
A_33_P3354564	2820	GPD2	ENST00000409861	0,676899382	7,535222361	3,32E-05
A_23_P406025	158471	PRUNE2	ENST00000223609	0,675769922	6,704053819	0,005188418
A_23_P32707	9700	ESPL1	ENST00000552462	0,675027612	7,128267822	0,000307507
A_23_P70398	7422	VEGFA	ENST00000518538	0,674475947	7,38832113	0,000332113
A_33_P3313596	728642	CDK11A	ENST00000401096	0,67356106	9,095634813	5,72E-06
A_22_P00009106	100874375	FAM155A-IT1	NA	0,673063215	9,285476902	0,038299617
A_33_P3363560	55092	TMEM51	ENST00000376014	0,672486075	7,501039504	0,052619317
A_21_P0012944	11039	SMA4	NA	0,672284598	10,94265901	0,053293005
A_33_P3293768	386674	KRTAP10-6	ENST00000400368	0,671928215	7,143575925	0,099278936
A_33_P3361412	55356	SLC22A15	ENST00000369502	0,671529006	7,295970788	0,058053256

A_24_P239731	9334	B4GALT5	ENST00000371711	0,670944771	10,73411954	0,005232703
A_23_P127663	79056	PRRG4	ENST00000257836	0,670460242	7,955002811	0,004755362
A_24_P314571	147841	SPC24	ENST00000592540	0,670332342	6,592305725	0,00335106
A_23_P94230	23643	LY96	ENST00000284818	0,66924033	9,582593336	0,046493025
A_23_P58321	890	CCNA2	ENST00000618014	0,669070411	6,481566111	0,001284241
A_23_P58321	890	CCNA2	ENST00000618014	0,669028851	6,503867757	0,002285856
A_23_P250358	55008	HERC6	ENST00000273960	0,6682485	6,837752669	0,014142729
A_23_P37441	567	B2M	ENST00000557901	0,667489564	15,86293617	0,00335106
A_23_P37441	567	B2M	ENST00000557901	0,66710378	16,13322573	0,002713719
A_23_P433152	132321	C4orf33	ENST00000425929	0,666572603	8,278252624	0,000208971
A_23_P88331	9787	DLGAP5	ENST00000247191	0,666371473	6,473014412	0,004271344
A_24_P88763	84695	LOXL3	ENST00000264094	0,665905819	8,709879132	0,000846472
A_23_P154235	9111	NMI	ENST00000243346	0,665771164	10,64229927	0,002542983
A_23_P16944	6382	SDC1	ENST00000381150	0,665296293	7,619668076	0,007242042
A_33_P3307163	282996	RBM20	ENST00000369519	0,664981238	8,393011541	0,023208725
A_23_P259189	25932	CLIC4	ENST00000488683	0,66386162	7,895385008	0,001280879
A_24_P388810	6728	SRP19	ENST00000520401	0,662503498	8,475055735	3,56E-05
A_33_P3349536	1111	CHEK1	ENST00000528276	0,661945752	6,536687428	0,000902059
A_23_P58321	890	CCNA2	ENST00000618014	0,661744108	6,491563517	0,001589633
A_23_P126241	8672	EIF4G3	ENST00000602326	0,661484333	8,928526907	0,000126395
A_23_P154235	9111	NMI	ENST00000243346	0,660017454	10,26879148	0,004376569
A_33_P3317523	3925	STMN1	ENST00000374291	0,66000206	10,49926137	0,078595029
A_23_P58321	890	CCNA2	ENST00000618014	0,658738356	6,433101629	0,001509152
A_33_P3278068	1997	ELF1	ENST00000239882	0,658476537	10,78389326	9,81E-05
A_33_P3220911	684	BST2	ENST00000252593	0,657991348	12,07555836	0,049029248
A_33_P3278941	9985	REC8	ENST00000559939	0,656657011	10,65477834	0,000744195
A_23_P79094	11026	LILRA3	ENST00000612127	0,655282762	10,2275362	0,049081399
A_23_P108751	2274	FHL2	ENST00000358129	0,654026913	8,357847483	0,072089338
A_23_P143958	200916	RPL22L1	ENST00000494771	0,653810107	9,69381504	0,084928497
A_23_P93180	8347	HIST1H2BC	ENST00000396984	0,653269512	9,408213881	0,000726332
A_23_P127385	4607	MYBPC3	ENST00000256993	0,653013637	9,147944751	0,013636226
A_33_P3252196	2146	EZH2	ENST00000320356	0,652805383	7,839255648	0,002438327
A_33_P3281816	10487	CAP1	ENST00000449311	0,651401455	9,571772041	3,53E-05
A_24_P55225	221421	RSPH9	ENST00000372163	0,650491945	6,881954675	0,000531279
A_33_P3322999	414152	C10orf105	ENST00000398786	0,650458144	8,728502367	0,07270601
A_33_P3236071	51266	CLEC1B	ENST00000348658	0,650058561	7,52619897	0,031226607
A_23_P121064	5806	PTX3	ENST00000295927	0,649730436	6,962743306	0,078915791
A_32_P96719	79801	SHCBP1	ENST00000303383	0,648852582	6,363904176	0,002584977
A_24_P383523	23034	SAMD4A	ENST00000554335	0,648315005	7,629139679	0,089177163
A_33_P3236065	51266	CLEC1B	ENST00000348658	0,648274598	7,950280344	0,053030506
A_23_P39465	684	BST2	ENST00000533098	0,64790865	11,32745365	0,051853586
A_23_P94703	27348	TOR1B	ENST00000259339	0,647137046	8,410024484	0,023820806
A_33_P3224710	22797	TFEC	ENST00000393485	0,646696859	8,407964896	0,033359358
A_23_P79572	85009	MGC16025	NA	0,646506266	7,294016289	0,000398703
A_33_P3400374	85441	HELZ2	ENST00000427522	0,645799859	12,12357791	0,085531565
A_32_P210202	144455	E2F7	ENST00000416496	0,645397034	6,476000903	0,001960219
A_23_P120566	6238	RRBP1	ENST00000246043	0,643134519	8,155642336	0,000316681
A_33_P3369760	152007	GLIPR2	ENST00000377959	0,643079992	10,8098122	0,000247293
A_23_P375	55143	CDCA8	ENST00000327331	0,642678204	7,694861779	0,015663926
A_23_P213584	3101	HK3	ENST00000292432	0,642576686	9,476323056	0,019452269
A_33_P3290573	55603	FAM46A	ENST00000369754	0,642474286	8,94614138	0,013627784
A_33_P3318796	10272	FSTL3	ENST00000166139	0,641154172	7,14032497	0,043452903
A_23_P216094	444	ASPH	ENST00000541428	0,640183165	7,65672882	0,02609726

A_32_P171328	27338	UBE2S	ENST00000264552	0,63987823	9,943073109	0,0003415
A_33_P3219279	84502	JPH4	ENST00000397118	0,639808721	7,0524208	0,001993426
A_23_P37441	567	B2M	ENST00000557901	0,63973172	16,04465288	0,003024787
A_23_P371039	4923	NTSR1	ENST00000370501	0,6388653	6,66187052	0,015611554
A_33_P3235987	5303	PIN4	ENST00000451079	0,638497685	8,135537908	0,000628014
A_19_P00804072	57674	RNF213	ENST00000559070	0,6366818	9,247937092	0,073280253
A_23_P37441	567	B2M	ENST00000557901	0,63664866	15,87787881	0,003552827
A_33_P3379669	727800	RNF208	ENST00000392827	0,636484084	7,36351682	0,008017302
A_33_P3282973	79870	BAALC	ENST00000297574	0,636230916	7,794789982	0,020444215
A_23_P128084	3679	ITGA7	ENST00000452168	0,63622556	8,651267559	0,040558252
A_19_P00809119	401237	CASC15	ENST00000444265	0,635958045	8,296341656	0,073280253
A_23_P65157	10063	COX17	ENST00000468918	0,635049412	9,767629114	0,000875553
A_32_P155247	2512	FTL	ENST00000427242	0,634086103	14,46214828	0,000266616
A_32_P91250	7332	UBE2L3	ENST00000496722	0,633922333	9,718147165	1,55E-05
A_33_P3267118	8672	EIF4G3	ENST00000374933	0,633046107	7,401014882	0,000135065
A_24_P195037	148423	C1orf52	ENST00000493514	0,632947164	8,57637796	3,73E-05
A_32_P103633	4171	MCM2	ENST00000265056	0,63225191	7,952817176	0,02928164
A_23_P99138	51258	MRPL51	ENST00000229238	0,63187856	11,02403787	0,005339987
A_23_P69310	9034	CCRL2	ENST00000400882	0,631564229	7,84881418	0,001825061
A_23_P114232	10549	PRDX4	ENST00000379341	0,631022444	9,912634046	0,025801131
A_33_P3313595	984	CDK11B	ENST00000341028	0,630028824	10,82266581	0,000655591
A_24_P163113	55573	CDV3	ENST00000431519	0,629932145	11,94436444	0,000486077
A_23_P2431	719	C3AR1	ENST00000307637	0,629660068	10,44052893	0,012041014
A_23_P130089	90410	IFT20	ENST00000322326	0,629474718	7,450701033	0,000253441
A_23_P41854	84674	CARD6	ENST00000254691	0,629346839	10,30089495	0,001709439
A_23_P19691	23593	HEBP2	ENST00000607197	0,629306867	12,163931	0,00322438
A_23_P121064	5806	PTX3	ENST00000295927	0,629141145	6,96984111	0,090432283
A_33_P3409765	8972	MGAM	NA	0,628787353	7,894259641	0,075880661
A_33_P3263902	4601	MXI1	ENST00000393134	0,627319227	9,013016882	0,091211541
A_33_P3397865	7138	TNNT1	ENST00000588981	0,626560407	8,932505841	0,058064072
A_23_P256455	6119	RPA3	ENST00000483031	0,626443354	9,625647609	0,004956407
A_23_P425752	9830	TRIM14	ENST00000342043	0,625729991	7,51407065	0,001649147
A_23_P8013	8340	HIST1H2BL	ENST00000377401	0,625550296	11,55644088	0,000674994
A_33_P3340025	9837	GINS1	ENST00000262460	0,624840687	6,767021979	0,010303292
A_23_P420551	11113	CIT	ENST00000545913	0,624662556	6,747298086	0,00301548
A_33_P3212994	11130	ZWINT	ENST00000318387	0,623838584	6,538245395	0,005412648
A_21_P0000052	27185	DISC1	ENST00000468399	0,622850104	8,438651079	0,018243943
A_23_P122924	3624	INHBA	ENST00000442711	0,622716945	6,514833681	0,096588789
A_23_P122852	6604	SMARCD3	ENST00000356800	0,62236119	11,29975164	0,009027536
A_23_P306941	266747	RGL4	ENST00000615003	0,62230981	10,95947831	0,075879089
A_32_P150891	81624	DIAPH3	ENST00000400324	0,622025272	6,348236751	0,000170499
A_23_P59005	6890	TAP1	ENST00000354258	0,620797538	13,63650534	0,033672507
A_33_P3393573	5110	PCMT1	ENST00000460828	0,620552922	9,033348826	0,000345637
A_23_P153745	10437	IFI30	ENST00000593731	0,62033814	15,17821977	0,047835467
A_33_P3231297	8804	CREG1	ENST00000370509	0,620265932	11,74635381	0,015614046
A_23_P29422	2992	GYG1	ENST00000296048	0,620127774	8,80983618	0,017302079
A_32_P212058	84984	CEP19	ENST00000409690	0,619940876	8,577547493	0,008160288
A_23_P48596	6035	RNASE1	ENST00000412779	0,619863021	9,104061153	0,008916716
A_32_P347617	164668	APOBEC3H	ENST00000613677	0,619706415	7,123704188	0,001164406
A_24_P148836	200942	KLHDC8B	ENST00000459846	0,619100398	7,09993412	0,004071372
A_23_P37441	567	B2M	ENST00000557901	0,618977086	15,86373864	0,005606171
A_23_P420196	8651	SOCS1	ENST00000332029	0,618823015	8,539421424	0,073143432
A_33_P3332955	51266	CLEC1B	ENST00000348658	0,618688149	7,852771873	0,043360445

A_24_P9883	91056	AP5B1	ENST00000532090	0,61865254	8,940030859	0,011755022
A_23_P127948	133	ADM	ENST00000528655	0,617742238	11,65835045	0,09340089
A_23_P127948	133	ADM	ENST00000528655	0,617704284	11,62355848	0,085909038
A_24_P334718	353345	GPR141	ENST00000334425	0,617153203	7,083108809	0,000582995
A_23_P45917	1163	CKS1B	ENST00000477676	0,616882022	9,325256684	0,008282926
A_23_P353717	116028	RMI2	ENST00000572173	0,616831647	7,69890645	0,016358382
A_24_P343095	1719	DHFR	ENST00000513048	0,616705443	7,136431435	0,000909817
A_33_P3306048	503645	DPRXP4	NA	0,616533785	8,746806522	0,003204488
A_32_P220715	81631	MAP1LC3B	ENST00000556529	0,616302344	10,45269136	0,001355777
A_23_P58321	890	CCNA2	ENST00000618014	0,615483524	6,451706078	0,003238388
A_23_P401904	26147	PHF19	ENST00000312189	0,615396182	9,790994667	0,002486556
A_33_P3410459	950	SCARB2	ENST00000264896	0,615010132	10,18459953	0,015905026
A_33_P3329255	8509	NDST2	ENST00000398701	0,613652188	7,869726916	0,006221995
A_23_P32404	3669	ISG20	ENST00000558942	0,613471417	14,7796693	0,026625728
A_24_P304723	5479	PPIB	ENST00000558492	0,612561771	10,50253693	0,004159179
A_23_P105571	56994	CHPT1	ENST00000550385	0,61211577	8,993256199	0,073749987
A_23_P37441	567	B2M	ENST00000557901	0,612095479	15,63806653	0,005541963
A_33_P3358099	342510	CD300E	ENST00000392619	0,612025178	10,46676566	0,028759696
A_23_P127948	133	ADM	ENST00000528655	0,611784004	11,76482985	0,098347414
A_23_P58321	890	CCNA2	ENST00000618014	0,611428242	6,458792596	0,003615624
A_23_P11461	7335	UBE2V1	ENST00000490555	0,610959934	9,997240922	0,000404875
A_23_P141974	7171	TPM4	ENST00000520239	0,610789863	10,21016404	4,85E-05
A_23_P119835	58484	NLRC4	ENST00000404025	0,610669827	10,48013713	0,021736308
A_23_P121064	5806	PTX3	ENST00000295927	0,609383702	6,981560618	0,098600819
A_24_P181677	80148	PQLC1	ENST00000474967	0,608727168	8,880839871	0,031840094
A_23_P130113	433	ASGR2	ENST00000254850	0,607579215	10,10515211	0,082978011
A_24_P257108	10010	TANK	ENST00000441987	0,606908831	8,805360423	8,73E-05
A_33_P3419733	80331	DNAJC5	ENST00000360864	0,606390354	8,367578478	0,000316681
A_23_P50368	126014	OSCAR	ENST00000610577	0,60622884	11,98110272	0,001416506
A_24_P218979	83461	CDCA3	ENST00000536241	0,604827677	6,700891124	0,001424593
A_23_P24515	38	ACAT1	ENST00000533597	0,6048137	8,025528036	0,001517147
A_23_P354547	10379	IRF9	ENST00000558468	0,604271779	8,118512205	0,004069844
A_23_P93282	8356	HIST1H3J	ENST00000359303	0,603687912	11,43937462	0,000460317
A_24_P167473	10094	ARPC3	ENST00000467622	0,603258644	13,75071694	0,000562873
A_33_P3292540	1031	CDKN2C	ENST00000371761	0,603243847	7,394834338	0,001004217
A_33_P3229122	8343	HIST1H2BF	NA	0,602889761	7,554867388	0,013055028
A_22_P00009369	790952	ESRG	NA	0,602559203	6,597770481	0,089644416
A_24_P283320	5110	PCMT1	ENST00000367378	0,602307084	7,919292915	0,000243186
A_23_P147805	7378	UPP1	ENST00000457596	0,601886062	9,689305397	0,001382745
A_33_P3345816	2852	GPER1	ENST00000619052	0,601277934	6,838531001	0,01940064
A_33_P3373364	25932	CLIC4	ENST00000422221	0,601195248	7,416955412	0,005459398
A_33_P3281795	11343	MGLL	ENST00000434178	0,600881036	8,519625042	0,036524843
A_33_P3360675	5575	PRKAR1B	ENST00000430040	0,600860689	7,69032915	0,095449069
A_23_P384517	2992	GYG1	ENST00000345003	0,600125506	10,24510295	0,039901143
A_23_P304287	5701	PSMC2	ENST00000292644	0,599650357	9,491258063	0,001495374
A_23_P157875	2219	FCN1	ENST00000371806	0,599284595	14,12427266	0,029633814
A_33_P3390580	9720	CCDC144A	ENST00000436374	0,598775366	8,096754835	0,053671447
A_23_P29257	3005	H1F0	ENST00000340857	0,598628686	7,765875152	0,040558252
A_21_P0000856	100288432	IL10RB-AS1	NA	0,598529438	7,704661152	0,001790093
A_23_P162874	3320	HSP90AA1	ENST00000554401	0,598103718	11,34063787	0,007778589
A_24_P86389	8336	HIST1H2AM	ENST00000359611	0,597941486	11,66066958	0,002016893
A_23_P160934	81611	ANP32E	ENST00000616917	0,597420393	8,758304821	0,001357797
A_24_P649624	23095	KIF1B	ENST00000377093	0,597328201	7,936352094	0,00184152

A_23_P31116	55856	ACOT13	ENST00000476436	0,597307816	8,640292108	0,000433122
A_33_P3220545	340024	SLC6A19	ENST00000515652	0,596774721	6,69919452	0,073396177
A_23_P428827	55122	AKIRIN2	ENST00000257787	0,596646767	9,061113433	0,000472212
A_23_P37441	567	B2M	ENST00000557901	0,596506647	15,95947909	0,007862789
A_33_P3423365	2934	GSN	ENST00000449733	0,596142814	9,629762792	0,009059737
A_22_P00008038	3490	IGFBP7	ENST00000514062	0,5942098	9,217928771	0,00949696
A_32_P70158	11025	LILRB3	ENST00000346401	0,594156662	14,99925347	0,021999495
A_23_P49768	51264	MRPL27	ENST00000225969	0,593135665	9,233539919	0,0005029
A_23_P105571	56994	CHPT1	ENST00000550385	0,592822734	8,89479663	0,088229399
A_33_P3807268	94236	DNAJA1P5	NA	0,592750482	10,00050819	0,001609355
A_23_P345591	5683	PSMA2	ENST00000457444	0,592016274	10,26833655	0,01230303
A_24_P196117	404672	GTF2H5	ENST00000607778	0,591670727	9,018911328	0,004218142
A_24_P12435	135112	NCOA7	ENST00000368357	0,591333608	9,436955139	0,035367025
A_33_P3388466	11119	BTN3A1	ENST00000425234	0,591076768	8,568141853	0,005478395
A_32_P134290	54877	ZCCHC2	ENST00000269499	0,590590011	8,286151407	0,082997087
A_33_P3211054	127396	ZNF684	ENST00000472043	0,590472735	6,789858532	0,001983857
A_19_P00801279	4478	MSN	ENST00000429601	0,589438006	7,133449337	0,011978924
A_23_P109881	3700	ITIH4	ENST00000464000	0,58847882	9,286258901	0,001118149
A_23_P144877	475	ATOX1	ENST00000313115	0,588076465	9,950132637	7,09E-05
A_23_P156180	6583	SLC22A4	ENST00000200652	0,587981556	9,446812245	0,044637691
A_23_P37441	567	B2M	ENST00000557901	0,587878809	15,82084419	0,008149852
A_33_P3400273	6402	SELL	ENST00000236147	0,587239985	14,56883781	0,055461779
A_23_P132936	60559	SPCS3	ENST00000507678	0,58720967	10,40810959	0,006964461
A_24_P160874	1854	DUT	ENST00000617900	0,586939331	8,846341762	0,005538336
A_23_P20122	56829	ZC3HAV1	ENST00000471652	0,586926575	8,545514943	0,017169704
A_23_P151791	1241	LTB4R	ENST00000553481	0,586576281	8,911314093	0,001370419
A_24_P286114	6507	SLC1A3	ENST00000613445	0,586318095	6,47960774	0,031339181
A_33_P3242483	394	ARHGAP5	ENST00000345122	-0,585416065	6,737050538	0,000101233
A_33_P3414789	79187	FSD1	ENST00000221856	-0,585638028	7,813515925	0,071116796
A_33_P3411427	116412	ZNF837	ENST00000597582	-0,58597795	7,947439793	0,001672127
A_23_P109774	27107	ZBTB11	ENST00000312938	-0,586234023	8,624456224	0,003348099
A_32_P98683	4302	MLLT6	ENST00000618876	-0,586262203	10,41583121	0,023830229
A_24_P927189	92106	OXNAD1	ENST00000285083	-0,586292049	7,631291611	0,010545259
A_22_P00003672	56882	CDC42SE1	ENST00000491825	-0,586554539	12,66340653	0,004766663
A_23_P151565	253959	RALGAPA1	ENST00000554259	-0,586554634	8,055381943	0,001954305
A_23_P100654	57659	ZBTB4	ENST00000311403	-0,586597322	9,092608868	0,007421917
A_23_P354798	93058	COQ10A	ENST00000308197	-0,586692692	7,700244932	0,002773045
A_23_P433785	5026	P2RX5	ENST00000552456	-0,587093577	8,748287245	0,040517154
A_32_P87697	3122	HLA-DRA	ENST00000613328	-0,587343067	12,88771972	0,094734677
A_23_P212715	868	CBLB	ENST00000476370	-0,587603631	9,224472119	0,013125035
A_24_P944616	50809	HP1BP3	ENST00000375003	-0,587678186	11,14258696	0,003680179
A_32_P60223	84289	ING5	ENST00000313552	-0,587867603	7,342522088	0,00094064
A_23_P12343	2947	GSTM3	ENST00000488824	-0,588105594	7,759596997	0,09131
A_23_P38219	10594	PRPF8	ENST00000572723	-0,588329917	10,13679601	0,000790579
A_23_P100654	57659	ZBTB4	ENST00000311403	-0,590458383	9,103028876	0,01104268
A_24_P267293	23098	SARM1	ENST00000585482	-0,592384516	7,823768503	0,001391849
A_23_P94095	157567	ANKRD46	ENST00000520311	-0,592991479	7,156336073	0,001581818
A_23_P566	22887	FOXJ3	ENST00000361346	-0,593309113	9,802359078	0,001784503
A_33_P3382746	3932	LCK	ENST00000469765	-0,593323359	11,24808779	0,005932897
A_23_P500601	89122	TRIM4	ENST00000355947	-0,593865376	10,07411014	0,002932702
A_23_P20463	5885	RAD21	ENST00000297338	-0,594000533	10,7075588	0,03396901
A_23_P336198	113263	GLCCI1	ENST00000482540	-0,59403393	8,211090792	0,041360162
A_19_P00801042	7643	ZNF90	ENST00000469078	-0,594956259	11,98008654	0,010314799

A_23_P120710	7267	TTC3	ENST00000355666	-0,595186894	8,104544137	0,00053076
A_23_P32414	51765	STK26	ENST00000394334	-0,595280531	9,725514815	0,051765507
A_23_P205389	64112	MOAP1	ENST00000556883	-0,595467057	8,077141089	0,0004511
A_23_P106727	9727	RAB11FIP3	ENST00000611004	-0,595902205	8,198457672	0,000452934
A_23_P117928	22893	BAHD1	ENST00000561234	-0,596142991	9,678256355	0,001439193
A_23_P122531	50854	C6orf48	ENST00000375640	-0,59625819	12,34428277	0,013973993
A_33_P3226605	11168	PSIP1	ENST00000380733	-0,596284597	9,436686013	0,00593055
A_24_P182947	257144	GCSAM	ENST00000308910	-0,596949063	6,81909977	0,000475296
A_33_P3257861	1757	SARDH	ENST00000439388	-0,59703254	7,738834583	0,002161614
A_33_P3227944	147081	CRHR1-IT1	ENST00000586362	-0,597067245	7,161351113	0,000843416
A_24_P320254	3187	HNRNPH1	ENST00000510678	-0,597718317	11,64914742	0,018040611
A_23_P162037	406	ARNTL	ENST00000472842	-0,597964086	9,691156993	0,002336016
A_23_P363399	81539	SLC38A1	ENST00000439706	-0,598599764	10,42498207	0,018154886
A_32_P41065	23023	TMCC1	ENST00000426664	-0,599263588	8,817378709	0,007319103
A_23_P109774	27107	ZBTB11	ENST00000312938	-0,599523261	8,753561209	0,003418016
A_23_P9135	79937	CNTNAP3	ENST00000377561	-0,599734113	6,870958748	0,056046823
A_23_P120710	7267	TTC3	ENST00000355666	-0,599811396	7,998849025	0,000677632
A_24_P411186	53335	BCL11A	ENST00000335712	-0,600013358	7,502810593	0,000491843
A_33_P3685216	1	A1BG	ENST00000598345	-0,60032238	8,320137027	0,000350396
A_23_P122805	84928	TMEM209	ENST00000397622	-0,601000551	7,840312709	0,004296835
A_23_P118306	9093	DNAJA3	ENST00000612103	-0,60101513	9,201530643	0,004376569
A_24_P759674	79991	OBFC1	NA	-0,601018052	7,397450447	4,64E-05
A_33_P3234277	3113	HLA-DPA1	ENST00000463243	-0,601433964	12,55387231	0,075585417
A_23_P157283	79161	TMEM243	ENST00000481425	-0,602624579	9,227465132	0,000307507
A_23_P100654	57659	ZBTB4	ENST00000311403	-0,602744429	9,195454451	0,006818111
A_33_P3718269	406938	MIR146A	ENST00000517927	-0,603306301	6,646811968	7,00E-05
A_32_P104746	57732	ZFYVE28	ENST00000290974	-0,60400914	7,711769266	0,00491659
A_23_P122531	50854	C6orf48	ENST00000375640	-0,604071356	12,03697406	0,01022654
A_23_P215931	23484	LEPROTL1	ENST00000321250	-0,604146258	10,6271287	0,041957697
A_23_P29836	131616	TMEM42	ENST00000477126	-0,604636796	9,637847186	0,005735204
A_33_P3638471	6981	TRGV7	NA	-0,604838503	7,561982878	0,018154886
A_33_P3411397	293	SLC25A6	ENST00000381401	-0,605038959	14,13853288	0,004273233
A_23_P103442	64769	MEAF6	ENST00000487788	-0,605149454	9,7453803	0,00172596
A_23_P164773	2208	FCER2	ENST00000597934	-0,605262396	6,836423558	0,005805661
A_33_P3400324	80012	PHC3	ENST00000495893	-0,605571301	10,04037522	0,012901704
A_23_P37623	23015	GOLGA8A	ENST00000432566	-0,607871072	7,666915351	0,001739745
A_23_P106727	9727	RAB11FIP3	ENST00000611004	-0,607888813	8,100443344	0,000446292
A_23_P338952	64766	S100PBP	ENST00000373475	-0,609383617	9,172103705	0,000664768
A_23_P130836	3004	GZMM	ENST00000264553	-0,609449726	10,889861	0,01859339
A_23_P110882	23270	TSPYL4	ENST00000420283	-0,609988421	8,083866895	0,000438576
A_24_P234732	10608	MXD4	ENST00000337190	-0,610199439	8,071623095	0,000189071
A_23_P13604	5037	PEBP1	ENST00000261313	-0,610725114	11,02862865	0,039186136
A_23_P80278	9814	SFI1	ENST00000464333	-0,610731137	10,2252273	0,013880069
A_33_P3379371	6259	RYK	ENST00000473208	-0,610743659	8,630200929	0,006274819
A_23_P117928	22893	BAHD1	ENST00000561234	-0,611178182	9,658259835	0,000667768
A_23_P69877	643836	ZFP62	ENST00000512132	-0,611458963	8,728859082	0,011529424
A_23_P337726	4508	ATP6	ENST00000361899	-0,611653427	14,03312081	0,004565781
A_23_P207742	7067	THRA	ENST00000264637	-0,611656202	10,19047556	0,00123832
A_23_P131676	57007	ACKR3	ENST00000272928	-0,611806007	7,104557628	0,000891954
A_32_P37592	677769	SCARNA17	NA	-0,611820658	13,79353365	0,023629117
A_23_P212002	4820	NKTR	ENST00000617821	-0,612560305	10,12932265	0,006291545
A_23_P417148	10743	RAI1	ENST00000353383	-0,612569427	7,371572792	0,000307507
A_23_P109636	26018	LRIG1	ENST00000496559	-0,613873722	8,401012119	0,0005029

A_23_P109636	26018	LRIG1	ENST00000496559	-0,613983554	8,402757379	0,000638084
A_23_P120710	7267	TTC3	ENST00000355666	-0,614305457	8,023163922	0,000628014
A_23_P152807	55316	RSAD1	ENST00000258955	-0,614384254	9,533209458	0,001167832
A_23_P135084	6130	RPL7A	ENST00000588490	-0,614663652	15,41199818	0,016222086
A_24_P89891	7185	TRAF1	ENST00000373887	-0,614736153	8,056950818	0,004572866
A_23_P371885	51326	ARL17A	ENST00000615623	-0,615036289	8,534407793	0,017877516
A_23_P33809	55272	IMP3	ENST00000403490	-0,615541138	10,70581275	0,002486556
A_23_P156327	7045	TGFBI	ENST00000504411	-0,615904137	12,50412992	0,065589154
A_23_P109774	27107	ZBTB11	ENST00000312938	-0,615939428	8,478022727	0,001138705
A_21_P0011808	375248	ANKRD36	ENST00000295246	-0,616032393	9,550760388	0,003507094
A_23_P1594	7423	VEGFB	ENST00000426086	-0,616045461	10,76863928	0,00102887
A_23_P56328	83483	PLVAP	ENST00000252590	-0,616467037	6,709288878	0,002459429
A_33_P3341601	349136	WDR86	ENST00000621812	-0,616589571	6,923214395	0,001460831
A_23_P99771	9240	PNMA1	ENST00000316836	-0,616646511	9,439035999	0,000433122
A_33_P3294252	466	ATF1	ENST00000262053	-0,616764905	8,103580337	0,018895912
A_23_P96777	57470	LRRC47	ENST00000378251	-0,617579995	9,56543927	0,00129207
A_24_P926960	1953	MEGF6	NA	-0,617718328	7,497655829	0,000346029
A_24_P916496	5578	PRKCA	ENST00000413366	-0,618201563	8,593750394	0,003156576
A_22_P00007531	641518	LEF1-AS1	NA	-0,618421336	6,996781384	0,010807276
A_33_P3250840	123606	NIPA1	ENST00000437912	-0,618627376	7,536633172	0,000704836
A_22_P00007370	100506915	CHRM3-AS2	NA	-0,618674299	6,975263449	0,005327183
A_23_P82324	84433	CARD11	ENST00000396946	-0,618842661	8,225064407	0,008562434
A_23_P118306	9093	DNAJA3	ENST00000612103	-0,618849533	9,173144286	0,0025404
A_24_P250650	11159	RABL2A	ENST00000452831	-0,619017035	10,45833602	0,001862182
A_23_P122531	50854	C6orf48	ENST00000375640	-0,620686701	12,00561448	0,011409609
A_23_P120710	7267	TTC3	ENST00000355666	-0,62105696	8,138328244	0,000700271
A_24_P354724	117289	TAGAP	ENST00000326965	-0,621339267	10,95254023	0,004885734
A_23_P387523	9923	ZBTB40	ENST00000374651	-0,6220611	9,298351435	0,000759722
A_24_P248053	116447	TOP1MT	ENST00000517857	-0,622068301	9,38021248	0,000331248
A_23_P27381	10194	TSHZ1	ENST00000584217	-0,622425115	8,5689393	0,002488169
A_33_P3385488	6222	RPS18	ENST00000412454	-0,622431417	12,93722487	0,065380188
A_23_P128663	26278	SACS	ENST00000382292	-0,622520644	7,312470035	0,003093374
A_24_P252130	5467	PPARD	ENST00000311565	-0,622791362	9,836375725	0,001877848
A_33_P3342410	29904	EEF2K	ENST00000263026	-0,622791508	8,409835559	0,001059935
A_23_P57667	5361	PLXNA1	ENST00000393409	-0,622964839	7,910709186	0,009651812
A_23_P120710	7267	TTC3	ENST00000355666	-0,623025914	8,002249526	0,000788082
A_23_P146849	321	APBA2	ENST00000620457	-0,623054245	6,761344579	0,000555996
A_24_P419300	25845	PP7080	ENST00000454935	-0,623457065	7,378284313	0,000554994
A_23_P100654	57659	ZBTB4	ENST00000311403	-0,623570529	9,154677348	0,006648697
A_24_P363087	51149	C5orf45	ENST00000610475	-0,624282123	9,67227265	0,001473788
A_33_P3323803	9659	PDE4DIP	ENST00000369356	-0,624495231	7,119584394	0,00019119
A_23_P43369	27240	SIT1	ENST00000474403	-0,624540417	8,852236712	0,008562434
A_23_P346376	27334	P2RY10	ENST00000475374	-0,624559152	7,023687661	5,03E-05
A_33_P3254320	10045	SH2D3A	ENST00000245908	-0,624952362	9,246240233	0,008185106
A_24_P413126	56937	PMEPA1	ENST00000265626	-0,625154055	6,677139119	3,39E-05
A_23_P14165	2841	GPR18	ENST00000340807	-0,625210501	8,008343466	0,005412648
A_23_P87560	694	BTG1	ENST00000256015	-0,625314395	11,35361542	0,000562873
A_23_P118306	9093	DNAJA3	ENST00000612103	-0,625557131	9,452546053	0,005144556
A_22_P00015497	100506776	TRG-AS1	NA	-0,625600138	9,026468197	0,021882174
A_33_P3262555	399664	MEX3D	ENST00000402693	-0,626317018	11,05082655	0,000745722
A_23_P209700	10316	NMUR1	ENST00000305141	-0,626729793	8,769017414	0,065144367
A_33_P3329444	284358	MAMSTR	ENST00000594582	-0,626982874	6,772335578	0,001770683
A_32_P99100	5796	PTPRK	ENST00000619256	-0,627129429	6,702179842	9,46E-05

A_33_P3387691	256380	SCML4	ENST00000369022	-0,627749528	10,88253923	0,094787072
A_24_P67308	100129424	RPL19P12	ENST00000432746	-0,628061763	14,0507557	0,002343706
A_32_P169179	55545	MSX2P1	NA	-0,628250982	7,225232354	0,003276576
A_23_P89310	22905	EPN2	ENST00000314728	-0,628267981	8,335484847	0,001515789
A_33_P3366336	388228	SBK1	ENST00000341901	-0,628456102	9,284073391	0,063159683
A_23_P31765	5569	PKIA	ENST00000352966	-0,628790827	7,681065804	0,001502242
A_33_P3368014	84329	HVCN1	ENST00000356742	-0,629484056	9,436845737	0,000269187
A_33_P3388651	3983	ABLIM1	ENST00000277895	-0,629563318	8,466088132	0,023626047
A_33_P3358923	151888	BTLA	ENST00000334529	-0,63020862	7,29642932	0,002688785
A_32_P71113	286205	SCAI	ENST00000336505	-0,630526803	7,038773566	0,001603957
A_23_P120710	7267	TTC3	ENST00000355666	-0,630683132	7,996309082	0,000891954
A_23_P345460	25894	PLEKHG4	ENST00000379344	-0,630708804	7,306987756	0,007924466
A_24_P354715	4907	NT5E	ENST00000257770	-0,631236475	6,532940584	5,72E-06
A_23_P50775	79414	LRFN3	ENST00000246529	-0,631330736	8,481410579	0,040391529
A_23_P19517	3710	ITPR3	ENST00000605930	-0,632392474	8,120498667	0,01283471
A_19_P00322948	641638	SNHG6	ENST00000520619	-0,632981721	11,14314989	0,009836513
A_24_P294124	9792	SERTAD2	ENST00000313349	-0,633991273	8,718637191	0,000313104
A_33_P3344204	79844	ZDHHC11	ENST00000283441	-0,634468128	7,938924356	0,019758498
A_24_P943193	9581	PREPL	ENST00000409936	-0,634861761	7,643259223	0,004196077
A_23_P47517	55048	VPS37C	ENST00000301765	-0,63492417	10,54169015	0,000603178
A_24_P319647	79368	FCRL2	ENST00000469986	-0,634934111	7,574760867	0,015653704
A_33_P3246163	6125	RPL5	ENST00000425640	-0,635008809	14,08888582	0,031105163
A_33_P3221680	6234	RPS28	NA	-0,635329058	15,75924908	0,006441867
A_23_P131096	5442	POLRMT	ENST00000589961	-0,635416495	9,75042032	0,002185335
A_23_P147888	6181	RPLP2	ENST00000321153	-0,635741184	16,97067821	0,001267369
A_23_P8108	3119	HLA-DQB1	ENST00000475182	-0,636693065	12,00216898	0,043639354
A_23_P122906	26053	AUTS2	ENST00000611706	-0,636830634	8,003058222	0,04231098
A_22_P00015308	27040	LAT	ENST00000562701	-0,637380777	9,5339064	0,001288918
A_33_P3333826	92912	UBE2Q2	ENST00000426727	-0,638172842	8,903329092	0,011408252
A_24_P237389	1964	EIF1AX	ENST00000379607	-0,638358888	7,980309831	0,008315538
A_23_P100420	23174	ZCCHC14	ENST00000568020	-0,638543977	7,748272895	3,73E-05
A_23_P12896	2188	FANCF	ENST00000327470	-0,639125297	8,176422439	0,001382745
A_23_P141505	10462	CLEC10A	ENST00000571664	-0,639652194	8,291803577	0,017534698
A_32_P193288	6142	RPL18A	ENST00000456046	-0,639761426	14,71513815	0,007932581
A_33_P3372074	3993	LLGL2	ENST00000578638	-0,641615534	10,54373515	0,040624373
A_33_P3287223	1803	DPP4	ENST00000360534	-0,641972723	7,953980746	0,011648578
A_23_P169838	23353	SUN1	ENST00000413171	-0,642558547	7,99760546	0,001993211
A_33_P3368830	4063	LY9	ENST00000368039	-0,642619675	9,718646768	0,001882571
A_23_P117928	22893	BAHD1	ENST00000561234	-0,644302459	9,530335331	0,000438576
A_33_P3663705	9696	CROCC	ENST00000375541	-0,644362624	8,685222788	0,001998959
A_24_P272061	23521	RPL13A	ENST00000430133	-0,645617841	14,29759119	0,005784716
A_33_P3296308	54876	DCAF16	ENST00000382247	-0,645719721	9,347039407	0,000498401
A_23_P26905	11232	POLG2	ENST00000577506	-0,645875039	9,061163587	0,001509152
A_23_P371215	29851	ICOS	ENST00000316386	-0,646340909	8,36419545	0,026426246
A_23_P128281	3823	KLRC3	ENST00000381903	-0,647351281	7,196912896	0,020872295
A_23_P68031	6775	STAT4	ENST00000392320	-0,647901117	9,571948116	0,010929622
A_24_P7085	118432	RPL29P2	ENST00000488409	-0,64830384	11,52814623	0,00129207
A_23_P109636	26018	LRIG1	ENST00000496559	-0,649628383	8,555778989	0,000616194
A_32_P45493	10772	SRSF10	ENST00000341154	-0,649775367	9,085683103	0,013958174
A_23_P117928	22893	BAHD1	ENST00000561234	-0,650398276	9,482386232	0,000438576
A_23_P65983	92922	CCDC102A	ENST00000569068	-0,650942842	7,797173266	0,004383618
A_32_P42197	3178	HNRNPA1	ENST00000340913	-0,650964575	13,1154598	0,023210077
A_23_P109636	26018	LRIG1	ENST00000496559	-0,650979952	8,406866897	0,000228567

A_33_P3390758	3312	HSPA8	ENST00000453788	-0,651022784	11,14291401	0,007343803
A_24_P289178	404550	C16orf74	ENST00000284245	-0,652652523	7,172637327	7,84E-05
A_23_P318581	57587	CFAP97	ENST00000458385	-0,652720062	8,581229483	0,00410102
A_23_P31335	7559	ZNF12	ENST00000342651	-0,653473466	8,693317715	0,001174367
A_24_P236091	2026	ENO2	ENST00000535366	-0,65442623	7,381425107	0,000416184
A_23_P122906	26053	AUTS2	ENST00000611706	-0,654866708	7,996105421	0,03897794
A_23_P315571	23180	RFTN1	ENST00000334133	-0,655070686	10,10724489	0,005793267
A_33_P3282836	6234	RPS28	ENST00000417088	-0,655570679	16,33081094	0,014970461
A_24_P56689	7755	ZNF205	ENST00000219091	-0,655757411	10,02876484	0,031657187
A_23_P125748	84460	ZMAT1	ENST00000488347	-0,657879893	8,185483075	0,000786688
A_33_P3214625	8821	INPP4B	ENST00000262992	-0,657918968	8,275637157	0,01493655
A_23_P251881	259197	NCR3	ENST00000376073	-0,658031335	10,62370017	0,05145468
A_24_P115932	11251	PTGDR2	ENST00000332539	-0,658198065	7,370888999	0,008434994
A_23_P122906	26053	AUTS2	ENST00000611706	-0,658298798	7,978939644	0,02760154
A_33_P3340060	23521	RPL13A	ENST00000446057	-0,65837033	13,84762475	0,013635473
A_23_P149368	115350	FCRL1	ENST00000495126	-0,658473425	7,561928033	0,012932163
A_24_P122050	9491	PSMF1	ENST00000381899	-0,658888167	10,42174724	0,032761611
A_23_P252155	55342	STRBP	ENST00000407982	-0,659033165	8,757968213	0,003021366
A_23_P83028	8434	RECK	ENST00000377966	-0,659479568	7,194971683	0,001115737
A_23_P132536	22906	TRAK1	ENST00000487159	-0,660408746	8,894645181	1,55E-05
A_23_P21057	1731	Sep 01	ENST00000566517	-0,660481977	9,013652392	0,009837012
A_23_P393051	126695	KDF1	ENST00000616918	-0,660485779	6,749919241	7,84E-05
A_23_P170857	3556	IL1RAP	ENST00000447382	-0,661238751	8,479788779	0,031291909
A_24_P276576	84824	FCRLA	ENST00000367950	-0,662812526	6,812535925	0,000966025
A_23_P100654	57659	ZBTB4	ENST00000311403	-0,663679767	9,038576992	0,002960657
A_23_P78888	2091	FBL	ENST00000593503	-0,663910335	13,20878596	0,01053528
A_23_P24763	6207	RPS13	ENST00000525828	-0,664623243	15,02198517	0,002176262
A_23_P24433	8722	CTSF	ENST00000527141	-0,66556407	8,892722917	0,009899376
A_23_P433676	57711	ZNF529	ENST00000591340	-0,665950446	7,413019954	9,24E-05
A_33_P3383912	3125	HLA-DRB3	ENST00000412634	-0,666298614	12,32841852	0,051643475
A_33_P3369761	54704	PDP1	ENST00000396200	-0,66660137	8,666211659	0,015772377
A_33_P3280993	414777	HCG18	NA	-0,666984226	8,637229149	0,001238715
A_23_P109636	26018	LRIG1	ENST00000496559	-0,667628944	8,598982332	0,000439905
A_32_P208120	57118	CAMK1D	ENST00000615792	-0,667830854	10,51023408	0,001118149
A_23_P145024	154	ADRB2	ENST00000305988	-0,668016356	10,50497692	0,004598292
A_23_P122906	26053	AUTS2	ENST00000611706	-0,668839642	8,022125403	0,036587605
A_23_P122906	26053	AUTS2	ENST00000611706	-0,670139698	8,28529985	0,055461779
A_23_P206228	54832	VPS13C	ENST00000560637	-0,670358849	8,983102634	0,005434711
A_24_P763243	1915	EEF1A1	ENST00000434005	-0,671371642	15,83449231	0,045004981
A_32_P52609	23175	LPIN1	ENST00000396097	-0,67197369	9,550265532	0,005308887
A_33_P3369461	57463	AMIGO1	ENST00000369862	-0,674451976	8,107750328	0,005735409
A_24_P317835	56623	INPP5E	ENST00000371712	-0,675015075	9,027928318	0,000745722
A_23_P122906	26053	AUTS2	ENST00000611706	-0,675523344	8,147129012	0,045138833
A_21_P0000841	100505483	PRKAG2-AS1	NA	-0,676192967	7,855749811	0,000546257
A_24_P135902	6187	RPS2	ENST00000402069	-0,676826866	15,34119073	0,004329079
A_23_P142146	6141	RPL18	ENST00000549370	-0,677127453	14,69240652	0,005153275
A_32_P147622	100289410	MCF2L-AS1	NA	-0,677345523	6,954338316	9,55E-05
A_23_P362183	203286	ANKS6	ENST00000353234	-0,677684359	8,04313164	0,00467174
A_33_P3289121	84417	C2orf40	ENST00000479337	-0,677784472	6,558053831	0,000216196
A_23_P140290	6252	RTN1	ENST00000557422	-0,678080854	7,126107081	0,001633117
A_23_P121250	1974	EIF4A2	ENST00000496382	-0,67857185	11,22071765	0,008160288
A_24_P47467	79073	TMEM109	ENST00000227525	-0,679828196	10,31764541	0,00532541
A_23_P53646	4673	NAP1L1	ENST00000261182	-0,680135564	10,22314536	0,001370555

A_33_P3234317	22800	RRAS2	ENST00000537760	-0,680720293	7,928539056	0,006552258
A_33_P3366039	6159	RPL29	ENST00000451924	-0,68126042	11,08253369	0,00108091
A_33_P3287959	10156	RASA4	ENST00000262940	-0,681663717	9,369187799	0,038224098
A_23_P398294	9026	HIP1R	ENST00000253083	-0,682563357	9,750534069	0,021693057
A_33_P3221458	7754	ZNF204P	ENST00000416749	-0,683715348	6,663001536	0,000594114
A_23_P15146	9235	IL32	ENST00000529550	-0,684377443	12,06480702	0,023804059
A_23_P345799	199786	FAM129C	ENST00000332386	-0,68462271	7,156389484	0,001089158
A_33_P3323722	10123	ARL4C	ENST00000390645	-0,685167777	11,4981371	0,017449681
A_24_P97374	8320	EOMES	ENST00000449599	-0,686903907	8,54390079	0,016943226
A_23_P70670	9308	CD83	ENST00000379153	-0,687388267	8,198467718	0,00345736
A_23_P109636	26018	LRIG1	ENST00000496559	-0,687403592	8,46232823	0,000229335
A_23_P22672	79868	ALG13	ENST00000624161	-0,687412202	8,808793911	0,00546605
A_33_P3271051	9265	CYTH3	ENST00000396741	-0,688154744	8,396527824	0,000488184
A_33_P3359306	9112	MTA1	ENST00000552286	-0,688549915	9,836487354	0,000350396
A_33_P3239347	4824	NKX3-1	ENST00000380871	-0,689218506	6,88329472	0,045410806
A_23_P316460	113763	ZBED6CL	ENST00000343855	-0,689470254	8,314061378	0,000245365
A_23_P211207	104	ADARB1	ENST00000437626	-0,689964232	8,985265191	0,00337024
A_23_P109636	26018	LRIG1	ENST00000496559	-0,690943328	8,632782776	0,000472212
A_23_P93524	154075	SAMD3	ENST00000437477	-0,69258762	9,006204338	0,017083269
A_33_P3212172	79856	SNX22	ENST00000557789	-0,693043513	8,072073252	0,001002298
A_33_P3373375	975	CD81	ENST00000468153	-0,693410219	11,23182753	0,00579525
A_23_P33759	9249	DHRS3	ENST00000616661	-0,693434085	9,113376513	0,008597124
A_23_P78742	2323	FLT3LG	ENST00000593422	-0,693532297	9,088736123	0,009495455
A_23_P404091	134266	GRPEL2	ENST00000507562	-0,693622048	7,77531446	0,000595549
A_23_P112798	1397	CRIP2	ENST00000548309	-0,693725484	6,793113903	8,96E-05
A_23_P6293	53347	UBASH3A	ENST00000473381	-0,694589206	8,343261514	0,006803929
A_32_P17635	10929	SRSF8	ENST00000587424	-0,695283746	8,328898786	0,000135368
A_33_P3411388	340260	UNCX	ENST00000316333	-0,695615748	12,33531131	0,000907494
A_23_P393777	5729	PTGDR	ENST00000306051	-0,696928554	8,256463576	0,007847554
A_23_P99661	55701	ARHGEF40	ENST00000553709	-0,698078729	11,25419094	0,039541076
A_33_P3326992	9659	PDE4DIP	ENST00000369356	-0,698885729	7,099910335	0,0003415
A_33_P3334308	158586	ZXDB	ENST00000374888	-0,699676807	7,874499924	0,000531613
A_32_P210252	6146	RPL22	ENST00000234875	-0,700343755	12,17963815	0,00390955
A_23_P167599	54463	FAM134B	ENST00000306320	-0,700985912	7,894519758	0,007377175
A_24_P358164	653162	RPSAP9	ENST00000483479	-0,702595264	12,87041499	0,012320597
A_23_P218549	84658	EMR3	ENST00000253673	-0,702611174	10,19459393	0,01130668
A_33_P3424222	3119	HLA-DQB1	ENST00000484729	-0,703088053	11,37013058	0,078835923
A_22_P00005863	2081	ERN1	ENST00000433197	-0,703369926	9,914530331	0,008360856
A_23_P120710	7267	TTC3	ENST00000355666	-0,703928029	8,083882557	0,000247293
A_32_P216548	26119	LDLRAP1	ENST00000488127	-0,704010341	8,759114273	0,002047722
A_23_P50678	4145	MATK	ENST00000612526	-0,704091142	8,812674519	0,001147221
A_23_P155417	84836	ABHD14B	ENST00000361143	-0,705087249	8,393135479	6,66E-05
A_23_P342131	220002	CYB561A3	ENST00000540755	-0,705599998	9,469246701	4,21E-05
A_24_P194081	53827	FXYD5	ENST00000496493	-0,705789004	11,35808447	0,000472212
A_23_P109636	26018	LRIG1	ENST00000496559	-0,706663051	8,626943631	0,00041343
A_23_P215956	4609	MYC	ENST00000613283	-0,706777322	9,294991684	0,006141398
A_23_P91390	7056	THBD	ENST00000377103	-0,707661772	10,25830901	0,026793084
A_23_P85952	79961	DENND2D	ENST00000357640	-0,710078003	11,97563353	0,012018056
A_23_P120710	7267	TTC3	ENST00000355666	-0,710682046	8,097020455	0,000232284
A_23_P100654	57659	ZBTB4	ENST00000311403	-0,713044826	9,040032053	0,002188911
A_23_P91764	115650	TNFRSF13C	ENST00000291232	-0,713672448	7,46077375	0,00266058
A_23_P215956	4609	MYC	ENST00000613283	-0,714157765	9,303670541	0,005846826
A_32_P24581	6233	RPS27A	ENST00000443181	-0,715366825	13,17855308	0,00642976

A_23_P163567	55512	SMPD3	ENST00000219334	-0,719488804	8,707095502	0,079543899
A_23_P215956	4609	MYC	ENST00000613283	-0,720087513	9,352094135	0,005784716
A_33_P3592015	22927	HABP4	ENST00000466976	-0,721303684	8,432139512	0,00337024
A_33_P3217347	64219	PJA1	ENST00000374571	-0,722990366	8,919384576	0,003583357
A_23_P145074	10957	PNRC1	ENST00000336032	-0,723091144	12,68116061	0,000338495
A_32_P225604	6125	RPL5	ENST00000426545	-0,723361725	13,73492322	0,015595029
A_23_P35205	11123	RCAN3	ENST00000374395	-0,72385073	8,412052689	0,001770683
A_24_P153043	118433	RPL23AP7	ENST00000450734	-0,723987564	14,18798763	0,000326018
A_33_P3382560	6147	RPL23A	ENST00000479658	-0,724039658	14,39181224	0,001264872
A_23_P19510	3120	HLA-DQB2	ENST00000416131	-0,724291203	8,951384142	0,027581915
A_23_P69431	6124	RPL4	ENST00000564647	-0,724616246	13,57720537	0,002074862
A_33_P3259393	145864	HAPLN3	ENST00000558770	-0,725102102	10,78921852	0,022745084
A_33_P3589217	293	SLC25A6	ENST00000484026	-0,725803684	11,50462033	0,000403025
A_33_P3404775	8761	PABPC4	ENST00000468476	-0,726804021	10,67209396	0,000316806
A_23_P202269	288	ANK3	ENST00000280772	-0,727599156	7,358904494	0,002473105
A_33_P3387272	1915	EEF1A1	ENST00000455918	-0,727685337	12,34415024	0,009502821
A_23_P70095	972	CD74	ENST00000523813	-0,728227933	10,34191541	0,017649378
A_24_P218006	84277	DNAJC30	ENST00000395176	-0,728845696	8,353046863	4,67E-05
A_23_P166536	23774	BRD1	ENST00000457780	-0,729434053	9,28277584	0,001485321
A_21_P0000336	677833	SNORA54	NA	-0,731502651	9,856121413	0,097144982
A_23_P16722	55619	DOCK10	ENST00000409592	-0,731880028	9,296460702	0,003383311
A_23_P371787	9766	SUSD6	ENST00000342745	-0,733466371	12,05294054	0,001449575
A_33_P3306068	4610	MYCL	NA	-0,734338858	9,285896574	0,022743886
A_22_P00000985	439949	PRKCQ-AS1	ENST00000455810	-0,73558438	9,674714307	0,014542199
A_23_P100654	57659	ZBTB4	ENST00000311403	-0,736767063	9,123996766	0,002671273
A_23_P41716	10399	GNB2L1	ENST00000508963	-0,73709929	13,41893913	0,0004511
A_33_P3420204	23373	CRTC1	ENST00000338797	-0,737119661	9,608580816	0,000779643
A_23_P204929	11215	AKAP11	ENST00000025301	-0,737229671	8,646263494	0,000396564
A_33_P3269723	125150	ZSWIM7	ENST00000497434	-0,737358272	8,695280686	0,003076392
A_23_P404494	3575	IL7R	ENST00000303115	-0,737603826	13,2091226	0,069138375
A_33_P3378665	123036	TC2N	ENST00000340892	-0,738672291	7,666264415	0,001252098
A_23_P14804	10099	TSPAN3	ENST00000560715	-0,739002759	8,679080972	0,000472212
A_23_P71867	3590	IL11RA	ENST00000602473	-0,739019659	8,263968381	0,00129207
A_24_P269814	59338	PLEKHA1	ENST00000368988	-0,739152112	7,970424516	0,005782313
A_23_P215956	4609	MYC	ENST00000613283	-0,740868298	9,495657246	0,005541963
A_24_P678104	50861	STMN3	ENST00000370053	-0,74111257	8,034436193	0,000151164
A_23_P30736	3112	HLA-DOB	ENST00000426644	-0,741114637	8,834432731	0,003980394
A_33_P3379916	2744	GLS	ENST00000320717	-0,741156417	8,015473679	0,000176618
A_23_P122906	26053	AUTS2	ENST00000611706	-0,742177924	8,325368266	0,035745238
A_23_P64898	10219	KLRG1	ENST00000538029	-0,74281823	10,91843311	0,064777761
A_23_P213592	22838	RNF44	ENST00000274811	-0,74343648	10,54373662	0,000170109
A_33_P3280950	144571	A2M-AS1	NA	-0,744824417	10,55465918	0,02067863
A_32_P44316	1915	EEF1A1	ENST00000443590	-0,745075225	12,36706603	0,00562278
A_33_P3240538	51175	TUBE1	ENST00000368662	-0,748388711	7,262287875	0,000475296
A_33_P3259148	729288	ZNF286B	ENST00000421016	-0,748863302	7,548938559	0,000221766
A_23_P82929	4856	NOV	ENST00000259526	-0,748910533	8,347733816	0,074708063
A_23_P122906	26053	AUTS2	ENST00000611706	-0,749849353	8,182616056	0,027437381
A_23_P201596	271	AMPD2	ENST00000528958	-0,750171311	9,686464081	0,000172439
A_24_P288836	3116	HLA-DPB2	ENST00000461425	-0,751948706	9,953846852	0,004206908
A_23_P251593	4736	RPL10A	ENST00000472730	-0,752535919	15,26878708	0,006221995
A_32_P195401	150864	FAM117B	ENST00000392238	-0,754693507	9,24117077	0,001685274
A_23_P109636	26018	LRIG1	ENST00000496559	-0,755636295	8,696581982	0,000172214
A_32_P146659	401431	ATP6V0E2-AS1	NA	-0,756949452	7,066062968	0,000240995

A_23_P11070	84636	GPR174	ENST00000276077	-0,758448007	7,289931586	0,000160586
A_23_P122906	26053	AUTS2	ENST00000611706	-0,758673025	8,314693362	0,034824301
A_33_P3336113	220359	TIGD3	ENST00000309880	-0,760493108	8,033457592	0,00568151
A_23_P218058	8302	KLRC4	ENST00000309384	-0,761547948	8,55622751	0,02760154
A_23_P200710	5287	PIK3C2B	ENST00000424712	-0,761628158	9,084144067	0,001952984
A_21_P0000792	100506779	BZRAP1-AS1	NA	-0,762048486	9,419616053	0,000214097
A_23_P354827	162972	ZNF550	ENST00000325134	-0,762846708	7,160917002	0,00078859
A_23_P132515	54847	SIDT1	ENST00000264852	-0,76293706	8,103925538	0,000491369
A_23_P418031	126917	IFFO2	ENST00000455833	-0,763026209	9,63079064	0,003980394
A_23_P24586	84680	ACCS	ENST00000263776	-0,763117256	8,134217035	0,008243891
A_23_P109636	26018	LRIG1	ENST00000496559	-0,764305258	8,735641517	0,000229335
A_33_P3277714	60468	BACH2	NA	-0,765769531	8,37546677	0,020213326
A_23_P410613	90488	TMEM263	ENST00000280756	-0,766360661	8,182745108	0,012918195
A_23_P68942	6122	RPL3	ENST00000481985	-0,766753046	14,95103672	0,009796096
A_33_P3301620	140545	RNF32	ENST00000469382	-0,767259099	10,02278125	0,005612446
A_21_P0000222	94161	SNORD46	NA	-0,768649516	8,023220962	0,032456651
A_23_P258769	3115	HLA-DPB1	ENST00000399500	-0,769265586	13,78223587	0,091807752
A_33_P3386671	6097	RORC	ENST00000356728	-0,770208371	7,534333012	0,004546531
A_23_P153676	7089	TLE2	ENST00000443826	-0,770494546	7,384160474	0,00114606
A_23_P125519	6191	RPS4X	ENST00000486733	-0,770839716	14,77084908	0,009333282
A_23_P100730	8631	SKAP1	ENST00000579476	-0,770968289	11,30243039	0,014071709
A_23_P140427	51466	EVL	ENST00000554045	-0,771200441	12,65953157	0,01912475
A_23_P215956	4609	MYC	ENST00000613283	-0,771561448	9,516476305	0,004741475
A_23_P13425	975	CD81	ENST00000468153	-0,772844744	11,28232341	0,005539887
A_23_P250735	23492	CBX7	ENST00000216133	-0,77337281	11,72361523	0,000411372
A_33_P3226995	924	CD7	ENST00000312648	-0,773739926	9,245128397	0,005459398
A_23_P55682	65982	ZSCAN18	ENST00000596372	-0,774280872	8,617799109	0,00999243
A_33_P3297245	22800	RRAS2	NA	-0,774706779	8,017296548	0,001792109
A_23_P37375	9252	RPS6KA5	ENST00000261991	-0,776940663	10,11812502	0,000389791
A_33_P3346826	9235	IL32	ENST00000528652	-0,777508866	10,74022466	0,007442801
A_23_P126844	8718	TNFRSF25	ENST00000513135	-0,777975725	11,90711207	0,031886151
A_32_P47701	1915	EEF1A1	ENST00000488500	-0,779798645	12,04863122	0,019615847
A_24_P218970	1975	EIF4B	ENST00000262056	-0,78021903	8,057310242	2,32E-05
A_23_P104641	738	VPS51	ENST00000534591	-0,780624941	11,6212569	1,45E-05
A_24_P261734	81539	SLC38A1	ENST00000398637	-0,781135246	9,379775288	0,005466328
A_23_P121657	9957	HS3ST1	ENST00000002596	-0,785054783	6,847030549	4,45E-05
A_23_P91095	940	CD28	ENST00000374481	-0,78550453	7,709417694	0,001503076
A_23_P161076	914	CD2	ENST00000369478	-0,785945937	12,76808673	0,020849557
A_23_P142776	8665	EIF3F	ENST00000434851	-0,785964385	10,5741539	0,000192854
A_33_P3295786	348378	FAM159A	ENST00000517870	-0,786090386	9,385099911	0,000950063
A_32_P206479	128611	ZNF831	ENST00000371030	-0,787619501	8,567982751	0,004205603
A_33_P3421626	57189	KIAA1147	ENST00000536163	-0,787795784	8,446249849	0,000667768
A_23_P125078	284129	SLC26A11	ENST00000411502	-0,788866222	9,890384335	0,00207799
A_33_P3248992	36	ACADSB	ENST00000358776	-0,790881097	7,888437685	0,001600415
A_23_P351148	117157	SH2D1B	ENST00000367929	-0,792645088	8,824797518	0,050198872
A_33_P3210965	79600	TCTN1	ENST00000460357	-0,792692393	7,823136922	4,48E-05
A_23_P58132	399	RHOH	ENST00000610353	-0,793286659	10,07702219	0,002373839
A_23_P98350	330	BIRC3	ENST00000263464	-0,793656842	9,882317454	0,002150008
A_22_P00007720	3119	HLA-DQB1	ENST00000460185	-0,794453048	12,09185233	0,051371709
A_33_P3271635	3115	HLA-DPB1	ENST00000402095	-0,795292713	11,0913275	0,016077057
A_32_P353072	54664	TMEM106B	ENST00000396667	-0,795332386	7,924944243	0,002006756
A_23_P208850	6217	RPS16	ENST00000601390	-0,796660641	14,83505173	0,001718435
A_24_P298360	4054	LTBP3	ENST00000528516	-0,797495138	7,792267431	0,000350396

A_23_P324490	9710	KIAA0355	ENST00000299505	-0,798106837	8,76980943	0,000234438
A_33_P3271651	3115	HLA-DPB1	ENST00000471170	-0,798558966	12,14063536	0,014125963
A_21_P0000463	100113382	SNORD105B	NA	-0,798902594	10,29642016	0,007834267
A_24_P14731	27344	PCSK1N	ENST00000218230	-0,799917736	12,07563834	0,00467174
A_23_P203115	84866	TMEM25	ENST00000533627	-0,802516234	7,559687242	0,000753296
A_23_P51231	864	RUNX3	ENST00000308873	-0,802986668	10,86791693	0,017519316
A_22_P00012003	5335	PLCG1	ENST00000423733	-0,804167939	9,286725031	5,53E-05
A_33_P3464555	57118	CAMK1D	ENST00000619168	-0,80568734	10,72867564	0,000155335
A_24_P67699	6147	RPL23A	ENST00000446445	-0,807050706	16,03326824	0,00180687
A_33_P3248794	4665	NAB2	ENST00000342556	-0,808480077	9,000400503	0,000247189
A_33_P3239587	439921	MXRA7	ENST00000375036	-0,80903513	10,16986625	0,066947039
A_23_P310931	1269	CNR2	ENST00000374472	-0,810126521	7,696231425	7,00E-05
A_24_P299911	23178	PASK	ENST00000358649	-0,813633604	7,893977628	0,003277501
A_23_P36888	91523	PCED1B	ENST00000546455	-0,815016082	10,97512899	0,00456788
A_24_P324674	4063	LY9	ENST00000392203	-0,815253038	8,509826426	0,000247973
A_32_P232559	439949	PRKCQ-AS1	ENST00000449648	-0,815879349	10,24867214	0,009851585
A_33_P3883985	64788	LMF1	ENST00000565467	-0,815917565	8,047140765	0,01906055
A_23_P109143	5621	PRNP	ENST00000379440	-0,816531282	9,45248827	0,010762109
A_23_P215956	4609	MYC	ENST00000613283	-0,817904532	9,417572353	0,002772123
A_23_P34676	919	CD247	ENST00000483825	-0,818813267	11,26304949	0,023670827
A_33_P3341616	387751	GVINP1	ENST00000531871	-0,818816185	9,147683339	0,000140499
A_33_P3393694	29997	GLTSCR2	ENST00000598681	-0,818858882	14,1358002	0,006406744
A_23_P165061	166	AES	ENST00000592414	-0,820274176	12,44509214	0,004383618
A_23_P215956	4609	MYC	ENST00000613283	-0,822520509	9,282807253	0,003054708
A_24_P169234	7535	ZAP70	ENST00000451498	-0,822657426	9,788280207	0,0124505
A_23_P357881	926	CD8B	ENST00000416057	-0,825759811	8,064711415	0,006804764
A_33_P3417626	375704	ENHO	ENST00000399775	-0,826174114	7,196757387	7,09E-05
A_23_P29747	6147	RPL23A	ENST00000580755	-0,826212883	16,13859655	0,00162031
A_21_P0014671	6207	RPS13	ENST00000525828	-0,830966698	14,52025233	0,000470565
A_23_P215956	4609	MYC	ENST00000613283	-0,832867752	9,306580405	0,002224839
A_23_P404481	1901	S1PR1	ENST00000305352	-0,832892863	9,493411789	0,002689002
A_33_P3360341	2625	GATA3	ENST00000379328	-0,83513045	7,818067565	0,000541076
A_23_P12140	6125	RPL5	ENST00000461952	-0,83603802	10,8635949	0,00485204
A_24_P166443	3115	HLA-DPB1	ENST00000402095	-0,837986655	12,74338219	0,008237385
A_23_P39131	29997	GLTSCR2	ENST00000599582	-0,839377661	10,82991086	0,000997645
A_33_P3234546	926	CD8B	ENST00000390655	-0,839433807	10,06965098	0,03941422
A_23_P255896	129293	TRABD2A	ENST00000335459	-0,840201114	7,679434664	0,00353966
A_23_P6413	140606	SELM	ENST00000490967	-0,842006275	7,819886988	0,000401718
A_23_P89062	100132341	CLUHP3	ENST00000254109	-0,843498974	8,546726063	0,001348315
A_33_P3298990	921	CD5	ENST00000347785	-0,845480678	10,77486703	0,026728133
A_24_P849801	6146	RPL22	ENST00000234875	-0,845494883	9,119884945	0,000350396
A_23_P209055	933	CD22	ENST00000594250	-0,848138755	7,366618025	0,001259034
A_24_P208345	85414	SLC45A3	ENST00000367145	-0,848678224	7,82665438	0,011545651
A_23_P124642	10125	RASGRP1	ENST00000539159	-0,849419103	10,13010655	0,006347449
A_21_P0000252	606500	SNORD68	ENST00000487034	-0,850544877	9,33604767	0,001541841
A_24_P388528	6480	ST6GAL1	ENST00000470633	-0,850884531	11,26984846	0,003272118
A_23_P76078	51561	IL23A	ENST00000228534	-0,85789349	7,791712531	0,00455608
A_23_P30634	60468	BACH2	ENST00000257749	-0,858303965	7,320451909	0,000278746
A_19_P00812587	6125	RPL5	ENST00000406671	-0,859330756	11,93529506	0,00390955
A_23_P151166	84329	HVCN1	ENST00000356742	-0,860559298	10,22750159	1,76E-05
A_33_P3223056	81794	ADAMTS10	ENST00000270328	-0,861218921	8,110231201	0,006804764
A_23_P21485	55022	PID1	ENST00000392055	-0,862799646	7,939095362	0,001382745
A_23_P207201	974	CD79B	ENST00000349817	-0,86831125	8,701454342	0,001956278

A_33_P3285734	343413	FCRL6	ENST00000392235	-0,868432454	7,744015005	0,011421198
A_23_P406385	146330	FBXL16	ENST00000324361	-0,870017025	9,02957029	0,00897071
A_23_P311875	923	CD6	ENST00000344931	-0,871261539	11,40076433	0,015210621
A_24_P808522	6208	RPS14	ENST00000401695	-0,871972897	12,38451727	0,00133251
A_23_P215956	4609	MYC	ENST00000613283	-0,874722687	9,463625284	0,001650009
A_33_P3363933	343413	FCRL6	ENST00000321935	-0,874844625	9,567829824	0,027615969
A_23_P44155	10225	CD96	ENST00000283285	-0,875360582	9,138346118	0,001794881
A_33_P3399064	100008587	RNA5-8S5	NA	-0,876115798	14,51454384	0,00484269
A_23_P501849	6137	RPL13	ENST00000562879	-0,876288555	13,16083704	0,001146714
A_33_P3335124	6187	RPS2	ENST00000468126	-0,879429401	14,05546678	0,000158411
A_23_P416747	916	CD3E	ENST00000531913	-0,880975135	8,954782299	0,00114606
A_23_P159335	926	CD8B	ENST00000390655	-0,883565987	11,37970341	0,036202977
A_23_P156218	3003	GZMK	ENST00000231009	-0,886469216	9,775402042	0,061017544
A_33_P3250671	6932	TCF7	ENST00000522561	-0,888071219	8,259414174	0,004692523
A_23_P253200	6138	RPL15	ENST00000413699	-0,892379659	10,36066193	0,000195013
A_23_P53763	80183	KIAA0226L	ENST00000441284	-0,892865682	8,870699602	0,0006601
A_32_P182941	6188	RPS3	ENST00000407940	-0,893290309	12,38800991	0,000472212
A_33_P3411296	5813	PURA	ENST00000331327	-0,894325107	9,801670151	0,000442573
A_33_P3259135	27065	NSG1	ENST00000621129	-0,89654973	7,589756433	0,010427851
A_33_P3368334	115352	FCRL3	ENST00000480682	-0,902703169	9,204611553	0,006450051
A_24_P766208	6122	RPL3	ENST00000417615	-0,903643931	14,48492126	0,002490665
A_23_P215956	4609	MYC	ENST00000613283	-0,904906881	9,655129571	0,001039611
A_24_P203000	3560	IL2RB	ENST00000216223	-0,906884815	10,96813277	0,012041014
A_23_P120227	81606	LBH	ENST00000395323	-0,914945447	10,16083166	0,001092547
A_23_P133543	26249	KLHL3	ENST00000309755	-0,917918848	7,532647527	0,000140993
A_23_P340019	197358	NLRC3	ENST00000615877	-0,922080722	10,62503267	0,002577057
A_24_P22079	2308	FOXO1	ENST00000379561	-0,927068289	8,579804626	3,32E-05
A_23_P103361	3932	LCK	ENST00000469765	-0,927427269	11,47142497	0,002773045
A_32_P62008	4736	RPL10A	ENST00000472730	-0,930559006	14,27722343	0,000843416
A_24_P87763	1938	EEF2	ENST00000309311	-0,931981107	12,85209687	9,98E-06
A_22_P00020127	100506776	TRG-AS1	NA	-0,933962169	8,385396697	0,000803986
A_23_P3911	57125	PLXDC1	ENST00000461225	-0,936337175	7,56684572	0,00370158
A_33_P3334515	57447	NDRG2	ENST00000556147	-0,937202656	9,419245267	7,09E-05
A_33_P3251462	128653	C20orf141	ENST00000603872	-0,937740916	11,45068787	0,000680631
A_24_P56270	8445	DYRK2	ENST00000344096	-0,937872761	8,349579149	0,000307167
A_23_P250302	1232	CCR3	ENST00000357422	-0,93873964	9,082990307	0,00553533
A_24_P136182	256355	RPS2P32	ENST00000423464	-0,942198127	14,06021444	3,56E-05
A_33_P3281985	1380	CR2	ENST00000367057	-0,94305827	7,093350004	9,46E-05
A_23_P107283	3212	HOXB2	ENST00000330070	-0,943491369	8,721131677	0,00033109
A_24_P319374	10223	GPA33	ENST00000367868	-0,947475324	7,964758515	0,000774949
A_23_P202520	3983	ABLIM1	ENST00000369252	-0,950138503	11,34844302	0,004626524
A_23_P43157	4603	MYBL1	ENST00000522677	-0,954280114	8,859069104	0,009496169
A_23_P354151	3702	ITK	ENST00000519402	-0,954989031	8,856860338	0,001004217
A_23_P96590	9737	GPRASP1	ENST00000537097	-0,957512785	7,478721531	0,000285987
A_33_P3364741	9902	MRC2	ENST00000583597	-0,957800937	10,28029803	0,061003559
A_23_P201731	7188	TRAF5	ENST00000336184	-0,957844053	9,102619396	0,000891954
A_23_P17134	4118	MAL	ENST00000353004	-0,959996409	11,57317789	0,012309099
A_23_P114057	54910	SEMA4C	ENST00000467747	-0,96213616	9,599677562	0,001895923
A_23_P50137	51320	MEX3C	ENST00000616921	-0,965699072	8,490600887	0,000733031
A_32_P15320	1915	EEF1A1	ENST00000415278	-0,971163489	12,4016681	0,001993211
A_33_P3214948	9806	SPOCK2	ENST00000373109	-0,975024504	12,58031571	0,009073773
A_32_P128258	284367	SIGLEC17P	ENST00000611992	-0,977806004	8,097893674	0,0026746
A_23_P99275	3820	KLRB1	ENST00000229402	-0,978814395	9,650996965	0,006357918

A_32_P163247	925	CD8A	ENST00000409511	-0,980646522	11,18389153	0,01960273
A_23_P114057	54910	SEMA4C	ENST00000467747	-0,98239378	9,680198925	0,001087039
A_23_P114057	54910	SEMA4C	ENST00000467747	-0,98841287	9,649672209	0,001278392
A_33_P3383233	57447	NDRG2	ENST00000397847	-0,996482454	8,284813241	0,000120156
A_23_P114057	54910	SEMA4C	ENST00000467747	-0,99666387	9,81542637	0,001649147
A_32_P114284	22807	IKZF2	ENST00000457361	-0,999099045	8,153133807	0,001187413
A_23_P350551	113246	C12orf57	ENST00000229281	-0,999828031	11,05117703	0,000499523
A_23_P148047	5734	PTGER4	ENST00000302472	-1,000318997	9,68293561	0,000432028
A_23_P205567	5583	PRKCH	ENST00000556245	-1,002626974	10,55745693	0,00220475
A_23_P19673	6446	SGK1	ENST00000367857	-1,00465547	10,25055452	0,001156883
A_24_P852756	3118	HLA-DQA2	ENST00000546801	-1,006548962	9,339791576	0,018325743
A_23_P122906	26053	AUTS2	ENST00000611706	-1,008653268	8,397468662	0,042925576
A_23_P8834	2053	EPHX2	ENST00000521400	-1,011446903	8,624111144	0,001770683
A_23_P114057	54910	SEMA4C	ENST00000467747	-1,016130305	9,68656699	0,000856465
A_24_P931443	8111	GPR68	ENST00000535815	-1,017199967	9,176788654	0,000983305
A_23_P341325	140801	RPL10L	ENST00000298283	-1,021295736	13,48686737	7,00E-05
A_23_P25566	1880	GPR183	ENST00000376414	-1,023573596	9,32539477	0,000211632
A_23_P151805	10516	FBLN5	ENST00000267620	-1,025323533	7,78201354	0,000809842
A_22_P00005343	3755	KCNG1	ENST00000506387	-1,025992315	7,450838163	0,001407737
A_23_P114057	54910	SEMA4C	ENST00000467747	-1,029945425	9,946300163	0,001685274
A_23_P114057	54910	SEMA4C	ENST00000467747	-1,033366962	9,879066301	0,001000736
A_23_P114057	54910	SEMA4C	ENST00000467747	-1,039456913	9,703439004	0,00071919
A_23_P114057	54910	SEMA4C	ENST00000467747	-1,043184843	9,975809624	0,001416506
A_33_P3299254	29802	VPREB3	ENST00000248948	-1,046732438	9,728010549	0,000536937
A_23_P113572	930	CD19	ENST00000611258	-1,076996763	10,2533699	0,013150027
A_23_P103765	2205	FCER1A	ENST00000368115	-1,081595259	8,658570323	0,01065057
A_23_P10232	55024	BANK1	ENST00000322953	-1,089354028	8,256313194	0,000124568
A_23_P114057	54910	SEMA4C	ENST00000467747	-1,090058646	9,8972729	0,000663982
A_33_P3379456	26751	SH3YL1	ENST00000356150	-1,094163437	8,391563196	0,001112112
A_33_P3308332	58473	PLEKHB1	ENST00000426191	-1,098973652	9,142847412	0,001981221
A_23_P501933	59285	CACNG6	ENST00000352529	-1,103540555	7,843928644	0,007333171
A_23_P57521	51386	EIF3L	ENST00000477256	-1,110146261	10,78845145	4,61E-06
A_33_P3257714	6228	RPS23	ENST00000296674	-1,117302694	9,189540691	0,000433797
A_23_P205738	64919	BCL11B	ENST00000345514	-1,128271297	10,28901751	0,001357046
A_23_P31725	640	BLK	ENST00000526097	-1,136734145	8,967310344	0,000541359
A_33_P3406567	931	MS4A1	ENST00000389939	-1,139680266	8,068431912	0,000402089
A_23_P46039	84824	FCRLA	ENST00000236938	-1,145994475	8,321023243	0,000472212
A_23_P30900	3118	HLA-DQA2	ENST00000552745	-1,149059657	8,062641931	0,015905026
A_23_P354805	11278	KLF12	ENST00000377669	-1,152960067	8,636998718	0,000663982
A_23_P21495	8857	FCGBP	ENST00000616721	-1,155468022	7,656546883	0,000541634
A_23_P343398	1236	CCR7	ENST00000246657	-1,168046984	13,21638612	0,010677809
A_23_P250212	157285	SGK223	ENST00000615670	-1,169763531	10,24750871	0,001154636
A_23_P39067	6689	SPIB	ENST00000270632	-1,174658793	9,166262551	9,62E-05
A_33_P3250680	959	CD40LG	ENST00000370629	-1,177420557	9,829145393	0,001999416
A_24_P252945	643	CXCR5	ENST00000292174	-1,191367579	8,513234174	0,000594114
A_23_P103601	57134	MAN1C1	ENST00000263979	-1,19576748	9,72014326	0,001491149
A_23_P138125	9214	FAIM3	ENST00000463473	-1,213670845	9,405700948	0,000170109
A_23_P112531	399665	FAM102A	ENST00000300434	-1,215441319	12,06324016	0,002104441
A_23_P112531	399665	FAM102A	ENST00000300434	-1,238295865	11,8175418	0,001847763
A_23_P112531	399665	FAM102A	ENST00000300434	-1,240316899	11,81211915	0,001463552
A_23_P112531	399665	FAM102A	ENST00000300434	-1,247274001	11,86591129	0,001572693
A_23_P112531	399665	FAM102A	ENST00000300434	-1,253843474	11,79207384	0,001464166
A_33_P3282556	79652	TMEM204	ENST00000566264	-1,260435926	9,372302497	7,95E-05

A_23_P112531	399665	FAM102A	ENST00000300434	-1,263772731	11,8359578	0,001586593
A_23_P112531	399665	FAM102A	ENST00000300434	-1,273653232	11,92425588	0,001463552
A_23_P56703	129293	TRABD2A	ENST00000335459	-1,285015048	9,639381213	0,000971585
A_24_P20630	51176	LEF1	ENST00000503879	-1,288700564	11,50191655	0,002348953
A_23_P112531	399665	FAM102A	ENST00000300434	-1,301167037	11,98672275	0,001348315
A_23_P112531	399665	FAM102A	ENST00000300434	-1,316947418	11,82776765	0,001284241
A_23_P357717	8115	TCL1A	ENST00000556450	-1,333121117	11,71961081	0,00973636
A_23_P7582	6932	TCF7	ENST00000522653	-1,336371161	10,81610946	0,001382745
A_33_P3401990	29802	VPREB3	ENST00000248948	-1,345807273	9,041895173	0,000959012
A_33_P3391796	9241	NOG	ENST00000332822	-1,349874633	7,503387556	0,000424949
A_23_P112531	399665	FAM102A	ENST00000300434	-1,368408161	12,04645208	0,000774949
A_32_P356316	3111	HLA-DOA	ENST00000229829	-1,374410755	10,46745065	3,82E-05
A_23_P31376	54674	LRRN3	ENST00000308478	-1,506684256	8,334942905	0,000439324
A_23_P10025	4753	NELL2	ENST00000437801	-1,523887021	8,592980206	6,59E-05
A_33_P3367396	400823	FAM177B	ENST00000360827	-1,567812324	10,09029715	0,009852505
