# Supplementary material for: Of mice and men: the host response to influenza virus infection
Source: Mamm Genome. 2018 Jun 15;29(7):446–70. doi: 10.1007/s00335-018-9750-y (PMC6132725; doi:10.1007/s00335-018-9750-y)
Supplement: Supplementary file 11 — Supplementary material 11 (PDF 162 KB) [file 335_2018_9750_MOESM11_ESM.pdf]

PROBE	ACCNUM	SYMBOL	ENTREZID	logFC	AveExpr	adj.P.Val
A_55_P2017929	NM_013492	Clu	12759	3,25806899	13,14549462	1,08E-16
A_55_P1961499	NM_010741	Ly6c1	17067	2,588938834	9,798752688	2,63E-13
A_51_P426875	NM_024228	Gdpd3	68616	2,556396871	7,987806452	0,036455655
A_51_P237752	NM_008986	Ptrf	19285	2,261526316	10,30196774	4,40E-08
A_51_P103397	NM_011708	Vwf	22371	2,255598862	10,75293548	1,86E-09
A_51_P343517	NM_010742	Ly6d	17068	2,216409673	8,777752688	5,79E-13
A_52_P553890	NM_016780	Itgb3	16416	2,111751778	11,69263441	3,02E-10
A_51_P254471	NM_007465	Birc2	11797	2,066019203	8,200139785	1,81E-14
A_55_P2141938	NR_027875	1810058I24Rik	67705	2,005026316	7,560870968	0,000272932
A_51_P105520	NM_153057	Nomo1	211548	1,974811522	9,93688172	3,47E-08
A_55_P2008061	NM_019923	Itpr2	16439	1,912814367	9,683655914	8,47E-17
A_55_P2068812	AK203298	Pkm	18746	1,910339972	12,00363441	4,72E-13
A_55_P1963764	NM_017461	Sep 01	54204	1,837328592	11,01411828	0,088229093
A_55_P2000107	NM_009720	Atox1	11927	1,833809388	12,34510753	1,83E-10
A_55_P1983754	NM_025557	Pcp4l1	66425	1,814171408	10,59837634	0,001136868
A_51_P453736	NM_001143686	Apol11b	328563	1,791680654	10,03232258	0,003477948
A_55_P2064771	NM_010741	Ly6c1	17067	1,734475107	12,40869892	3,98E-07
A_51_P151576	NM_001001999	Gp1bb	14724	1,706090327	9,984376344	3,34E-06
A_55_P2121408	NM_009416	Tpm2	22004	1,679754623	7,804	6,13E-06
A_52_P315976	NM_009416	Tpm2	22004	1,61664367	7,593677419	2,59E-05
A_55_P2085295	NM_198411	Inf2	70435	1,603283784	9,953731183	3,29E-10
A_66_P139618	NM_001082545	Stfa2	20862	1,591291607	9,333978495	0,00301006
A_55_P2130178	NM_010233	Fn1	14268	1,585829303	8,488526882	5,07E-06
A_55_P1966838	NM_001037713	Xaf1	327959	1,558310811	7,586946237	0,011039629
A_51_P265495	NM_010738	Ly6a	110454	1,55499431	10,43309677	3,52E-06
A_52_P398925	NM_173869	Stfa2l1	268885	1,542201991	9,132602151	0,004176047
A_55_P1999102	NM_023734	Pi16	74116	1,541047653	9,039473118	0,00522067
A_55_P2031436	NM_001164036	Ly6e	17069	1,538949502	11,78717204	5,94E-10
A_55_P2001920	NM_010227	Flna	192176	1,528492888	12,19695699	6,39E-09
A_52_P562676	NM_013873	Sult4a1	29859	1,502236131	8,355064516	0,000338826
A_52_P459564	NM_010575	Itga2b	16399	1,496818634	10,17980645	8,26E-05
A_51_P428372	NM_023785	Ppbp	57349	1,47183357	13,10597849	3,24E-07
A_51_P279062	NM_027763	Treml1	71326	1,45528165	12,3243871	3,67E-05
A_51_P153995	NM_018762	Gp9	54368	1,448406117	12,70202151	7,31E-06
A_55_P1955308	NM_001002898	Sirpb1a	320832	1,444322902	10,17219355	4,98E-06
A_55_P1986296	NM_178598	Tagln2	21346	1,442017781	13,72156989	6,98E-08
A_51_P108226	NM_183249	Wfdc21	66107	1,439885491	7,763978495	0,000683692
A_55_P2027278	NM_144560	Gas2l1	78926	1,432650782	11,75053763	1,53E-05
A_55_P2019577	NR_027818	1500011B03Rik	66236	1,431461593	7,991591398	1,64E-09
A_55_P1953459	NM_001171147	Yap1	22601	1,430955903	7,958978495	3,29E-10
A_51_P359636	NM_011150	Lgals3bp	19039	1,427689189	11,08201075	1,12E-05
A_51_P174961	NM_007972	F10	14058	1,426506401	8,668806452	7,10E-06
A_55_P2211164	AK017236	5330406M23Rik	76671	1,424570413	8,073634409	5,86E-09
A_55_P2016852	NM_011777	Zyx	22793	1,407481508	13,11324731	6,77E-06
A_55_P2110758	NM_177083	B430306N03Rik	320148	1,389206259	9,145021505	4,64E-07
A_55_P2078735	NR_015519	AI662270	100043636	1,377008535	6,926	0,000469241
A_55_P2076545	NM_007976	F5	14067	1,374726885	9,015763441	4,12E-07
A_52_P661	NM_175750	Plxna4	243743	1,358972262	7,940806452	9,48E-06
A_55_P2060107	NM_011099	Pkm	18746	1,347330725	10,01480645	0,000681054

A_55_P2112737	NM_007393	Actb	11461	1,333655761	16,12345161	6,53E-07
A_55_P1986531	NM_001191012	G6b	106722	1,331857752	8,982494624	0,000176281
A_55_P2099650	NM_053195	Slc24a3	94249	1,316958748	9,323376344	4,07E-07
A_55_P2083121	NM_001164567	Vill	22351	1,313324324	7,407010753	1,05E-05
A_55_P2028600	NM_022410	Myh9	17886	1,310463016	11,99136559	4,35E-09
A_55_P1991770	NM_019417	Pdlim4	30794	1,307874822	8,329043011	4,05E-11
A_51_P507801	NM_028784	F13a1	74145	1,307526316	10,24087097	6,45E-07
A_51_P286814	NM_011424	Ncor2	20602	1,306094595	8,863258065	9,59E-12
A_55_P1956973	NM_011602	Tln1	21894	1,302980797	11,63656989	6,44E-06
A_55_P2048607	NM_017370	Hp	15439	1,2776266	12,33929032	0,000309217
A_52_P207335	NM_025944	Tmem246	67063	1,275022048	8,197849462	0,00021333
A_51_P401907	NM_001082547	Gm5483	433016	1,272687055	8,288096774	0,01533845
A_51_P407323	NM_007976	F5	14067	1,272445946	7,869483871	3,33E-06
A_51_P133684	NM_013808	Csrp3	13009	1,265014225	6,806096774	0,047582519
A_55_P2141395	NM_023463	Ly6g6c	68468	1,260059744	10,16783871	2,81E-06
A_55_P2016049	NM_001163014	Gp6	243816	1,258966572	9,423021505	1,70E-06
A_52_P472324	NM_011414	Slpi	20568	1,256113798	11,84569892	4,90E-05
A_52_P487686	NM_001082546	BC100530	100034684	1,253741821	8,938548387	0,015778679
A_55_P2007196	NM_001177307	Aldoa	11674	1,233351351	12,76237634	1,51E-12
A_51_P359570	NM_010501	Ifit3	15959	1,232918919	9,949032258	0,006818352
A_51_P364485	NM_009396	Tnfaip2	21928	1,22966074	8,789387097	9,40E-08
A_52_P37894	NM_009945	Cox7a2	12866	1,222075391	10,48419355	1,46E-07
A_55_P1998943	NM_145211	Oas1a	246730	1,222067568	10,91839785	0,002977832
A_51_P427663	NM_007725	Cnn2	12798	1,21474111	10,86098925	8,71E-07
A_55_P2212161	AK009622	2310034O05Rik	69571	1,210560455	7,937978495	0,009195645
A_55_P1963017	NM_001082543	Stfa1	20861	1,208726174	8,119150538	0,012728892
A_55_P2171116	NM_001145953	Lgals3	16854	1,207271693	11,07767742	8,72E-05
A_55_P2037697	NM_001076679	Gm9733	751864	1,203219772	7,840032258	1,51E-08
A_55_P2004137	NM_008138	Gnai2	14678	1,201182077	12,31351613	3,98E-07
A_51_P222657	NM_009775	Tspo	12257	1,196406117	10,67650538	3,05E-11
A_51_P365019	NM_010295	Gclc	14629	1,195578947	7,225741935	2,04E-18
A_51_P405476	NM_010185	Fcer1g	14127	1,192815789	12,74780645	3,41E-09
A_52_P38639	NM_153795	Fermt3	108101	1,188711238	9,036172043	5,21E-06
A_55_P2046145	AK137448	Gm10693	675749	1,180105263	9,859903226	5,20E-06
A_55_P2152009	NM_021344	Tesc	57816	1,173654339	9,953086022	0,000285977
A_51_P231320	NM_008611	Mmp8	17394	1,170416074	8,326193548	0,000246141
A_51_P212754	NM_009369	Tgfbi	21810	1,164721195	10,61845161	0,000224136
A_55_P2052769	NM_001042743	Mast2	17776	1,162691323	9,061731183	1,43E-05
A_52_P467449	NM_007440	Alox12	11684	1,160876956	11,03665591	0,000183884
A_55_P2066230	NM_001172117	Hck	15162	1,157972973	10,30739785	7,68E-07
A_55_P2180869	NM_029865	Ocel1	77090	1,15205761	8,847322581	1,40E-06
A_55_P1953301	NM_146126	Sord	20322	1,146901138	8,130430108	0,000443929
A_55_P2008907	NM_009609	Actg1	11465	1,145926743	13,44660215	1,04E-06
A_51_P225592	NM_001001491	Tpm4	326618	1,145105263	11,62805376	8,35E-06
A_55_P2041075	NM_001081636	Ccnd3	12445	1,143669275	11,78927957	1,88E-06
A_51_P176156	NM_001173459	LOC100038947	100038947	1,141766003	8,715344086	3,31E-06
A_51_P341746	NM_010823	Mpl	17480	1,140880512	9,437376344	4,93E-05
A_55_P2060630	NM_027886	Stk11ip	71728	1,140346373	7,412688172	0,023932291
A_51_P357573	NM_145575	Cald1	109624	1,140004979	7,265784946	1,08E-06
A_55_P2026233	NM_023738	Uba7	74153	1,131990754	11,48988172	4,69E-05

A_51_P173622	NM_011202	Ptpn11	19247	1,125578947	10,45004301	8,17E-06
A_55_P2151685	NM_011088	Pira11	18724	1,122941679	9,527365591	1,27E-05
A_55_P2000548	NM_007687	Cfl1	12631	1,119705548	12,50105376	1,27E-07
A_66_P120380	NM_177820	Apol10b	328561	1,111499289	8,180892473	0,024713502
A_55_P2139181	NM_145575	Cald1	109624	1,102348506	7,099032258	2,45E-06
A_55_P2058757	NM_007598	Cap1	12331	1,09493101	10,5481828	4,27E-07
A_55_P2071858	NM_008598	Mgmt	17314	1,094122333	7,55011828	3,06E-06
A_51_P217336	NM_029153	Scamp1	107767	1,09162091	7,934075269	7,64E-06
A_55_P2088385	NM_011618	Tnnt1	21955	1,08820697	8,266043011	1,57E-05
A_55_P2015670	NM_008397	Itga6	16403	1,08497155	12,40425806	0,001331077
A_55_P2021266	NM_152803	Hpse	15442	1,084862731	9,626645161	6,07E-06
A_52_P633410	NM_020590	Gabarapl1	57436	1,081071124	11,61583871	6,37E-05
A_52_P151853	NM_001025262	Tpd52	21985	1,080401849	8,241043011	3,80E-08
A_55_P1990919	NM_013759	Msrb1	27361	1,070554054	13,61383871	0,000130497
A_55_P2176492	NM_023764	Tollip	54473	1,06702276	7,823870968	0,039909903
A_55_P2144526	NM_001080381	Fam65b	193385	1,066623755	9,402290323	1,08E-05
A_66_P132493	NM_031181	Siglece	83382	1,058993599	12,06790323	0,028116916
A_51_P482711	NM_053272	Dhcr24	74754	1,056836415	10,35587097	0,000149051
A_52_P409769	NM_194464	Mrvi1	17540	1,053560455	8,898053763	0,000807548
A_52_P425839	NM_181596	Retnlg	245195	1,051901138	12,01383871	0,005937941
A_51_P198645	NM_133167	Parvb	170736	1,051433144	7,885150538	5,79E-05
A_51_P294555	NM_001033632	Ifitm6	213002	1,051197724	9,718752688	9,45E-05
A_55_P2007470	NM_008808	Pdgfa	18590	1,047019203	9,263322581	0,000197001
A_51_P192800	NM_008175	Grn	14824	1,045738265	8,388408602	3,32E-06
A_55_P1956457	NM_029998	6030458C11Rik	77877	1,039187055	7,287301075	0,000594345
A_55_P1962010	NM_019985	Clec1b	56760	1,030564011	10,9573871	0,000166842
A_55_P2000127	NM_009365	Tgfb1i1	21804	1,025220484	7,922924731	7,10E-06
A_55_P2034928	NM_001037925	BC147527	625360	1,018542674	8,459032258	1,23E-05
A_51_P279100	NM_008969	Ptgs1	19224	1,015215505	8,94927957	0,000510812
A_55_P1962305	NM_139198	Plac8	231507	1,014470128	12,11310753	0,003376527
A_55_P2056973	NM_001033405	Treml2	328833	1,013015647	9,005634409	1,27E-05
A_55_P2017636	NM_011580	Thbs1	21825	1,010467994	9,744344086	0,004038794
A_52_P239086	NM_177744	Apol10a	245282	1,006642959	7,871774194	0,010530057
A_51_P441426	NM_019932	Pf4	56744	1,003448791	15,27054839	0,001164738
A_55_P2086433	NM_145209	Oasl1	231655	0,99905761	9,998580645	0,009457159
A_55_P1974577	NM_028728	Nfam1	74039	0,997490043	9,793333333	3,44E-05
A_55_P2139385	NR_002890	Gm12070	654472	0,993852063	11,87833333	2,00E-07
A_51_P346938	NM_029796	Lrg1	76905	0,991990043	10,27395699	0,030429804
A_55_P2139473	NM_001161724	Ilk	16202	0,990842817	10,63609677	0,00022795
A_55_P2012201	NM_007471	App	11820	0,987305121	7,858387097	3,47E-07
A_55_P2125588	NM_008808	Pdgfa	18590	0,986184211	8,848021505	0,000240389
A_55_P2030506	NM_018739	Rp9	55934	0,981226174	7,792129032	2,85E-09
A_51_P240019	NM_013755	Gyg	27357	0,981133001	9,203107527	3,12E-06
A_55_P2081488	NM_009402	Pglyrp1	21946	0,979995733	9,422623656	0,000229449
A_55_P1955305	NM_001002898	Sirpb1a	320832	0,976093883	9,699150538	6,56E-05
A_55_P1992910	NM_013755	Gyg	27357	0,974375533	9,170204301	6,40E-06
A_55_P2079579	NM_011094	Pira7	18730	0,966284495	9,961397849	0,000225802
A_51_P439996	NM_008148	Gp5	14729	0,964179943	8,752354839	0,001219212
A_55_P2013948	NM_146120	Gsn	227753	0,963679943	10,81495699	0,000183354
A_55_P2038007	NM_007791	Csrp1	13007	0,962647226	10,82692473	0,000345679

A_51_P343252	NM_009842	Cd151	12476	0,961568279	10,23164516	0,000247234
A_51_P308298	NM_172118	Myl9	98932	0,960263869	14,40034409	0,012568406
A_55_P2033120	NM_029688	Srxn1	76650	0,958042674	10,20589247	0,000773949
A_52_P370203	NM_021878	Jarid2	16468	0,953935277	8,268150538	8,60E-06
A_66_P133328	NM_001025261	Tpd52	21985	0,953880512	9,492580645	3,15E-07
A_51_P238448	NM_007632	Ccnd3	12445	0,953062589	9,919666667	1,10E-05
A_51_P440047	NM_173752	Lgalsl	216551	0,951325036	8,491494624	0,003283242
A_55_P2106956	NR_004447	Gm5523	433273	0,950639403	12,81258065	5,46E-06
A_55_P1970597	NM_009609	Actg1	11465	0,949303698	14,74409677	0,000676365
A_55_P2090209	NM_022314	Tpm3	59069	0,945953058	12,51595699	2,88E-05
A_51_P154842	NM_145153	Oas1f	243262	0,945495733	10,01017204	0,008740493
A_55_P1962084	NM_013549	Hist2h2aa1	15267	0,944366999	12,68001075	2,74E-05
A_51_P423290	NM_027613	Mmrn1	70945	0,944331437	9,281666667	0,000250927
A_51_P181517	NM_144559	Fcgr4	246256	0,944032717	8,880376344	0,000132084
A_55_P2093994	NM_030700	Maged2	80884	0,94333357	9,097451613	0,002032335
A_55_P2019113	NM_001024848	Apol7b	278679	0,941627312	7,342462366	6,56E-06
A_51_P187082	NM_008062	G6pdx	14381	0,940219772	9,393763441	1,03E-06
A_55_P1980831	NM_029711	Arpc2	76709	0,938767425	12,94587097	5,96E-05
A_55_P2033041	NM_001173460	Sirpb1b	668101	0,937463016	10,08956989	0,000444355
A_55_P2005853	NM_001037098	Nacc2	67991	0,935608108	7,764462366	2,38E-06
A_52_P478256	NM_178614	Samm50	68653	0,933696302	7,821204301	0,014496713
A_51_P358722	NM_173414	Lancl3	236285	0,931261024	7,03316129	0,000691472
A_66_P113059	NM_010578	Itgb1	16412	0,931134424	8,437376344	6,80E-06
A_55_P1985984	NM_009609	Actg1	11465	0,929603129	11,045	5,86E-05
A_55_P1983523	NM_145437	Cd300ld	217305	0,929549075	8,778956989	0,010244186
A_51_P235687	NM_009663	Alox5ap	11690	0,927453058	12,02923656	0,000239719
A_51_P512992	NM_178676	Entpd3	215446	0,927400427	6,949247312	0,001691984
A_55_P2064351	NM_011703	Vipr1	22354	0,925103841	7,863946237	6,07E-05
A_55_P2035038	XM_001473590	Gm2437	100039816	0,921768137	10,76387097	0,001657933
A_55_P2002846	NM_001081365	Cystm1	66060	0,917073969	7,374451613	0,049168823
A_55_P2170225	NM_172507	Sh3bgrl2	212531	0,916835704	7,542946237	7,77E-08
A_55_P2186027	NM_008084	Gapdh	14433	0,916306543	12,96194624	1,33E-05
A_55_P2142830	NM_013610	Ninj1	18081	0,913916785	11,75462366	8,51E-05
A_55_P2114993	NM_009609	Actg1	11465	0,913130156	14,47189247	0,000344283
A_55_P1966843	NR_002890	Gm12070	654472	0,907832148	12,84315054	2,17E-05
A_55_P1992617	NM_019985	Clec1b	56760	0,907573257	12,28836559	0,00074983
A_55_P1962011	NM_019985	Clec1b	56760	0,905459459	12,57847312	0,000985409
A_55_P2079560	NM_011090	Lilra6	18726	0,899981508	9,947430108	0,00034787
A_52_P317393	NM_018882	Gpr56	14766	0,899016358	8,485451613	0,001007324
A_55_P2116650	NR_002860	A130040M12Rik	319269	0,898933855	10,48433333	0,00093836
A_51_P413866	NM_008198	Cfb	14962	0,898559744	7,870193548	0,006426624
A_66_P125862	NR_015487	A930005H10Rik	68161	0,898117354	8,337473118	6,54E-06
A_52_P559975	NM_009909	Cxcr2	12765	0,897326458	9,757580645	0,005337225
A_55_P2051334	NR_004446	H2-K2	630499	0,8965633	12,84823656	0,006584566
A_52_P126266	NM_182997	Prkab2	108097	0,896251067	7,951935484	7,68E-05
A_52_P52618	NM_007780	Csf2rb	12983	0,89306899	9,284666667	0,000140712
A_55_P2063251	NM_028207	Dusp3	72349	0,889395448	7,571322581	0,000295087
A_55_P1970700	NM_011029	Rpsa	16785	0,887785917	14,23398925	0,008723999
A_51_P144868	NM_029934	Mboat7	77582	0,885966572	8,735225806	6,08E-07
A_51_P109449	NM_008519	Ltb4r1	16995	0,885724751	8,385086022	4,74E-06

A_55_P2037425	NM_008084	Gapdh	14433	0,885464438	11,70724731	1,65E-05
A_55_P1970105	NM_013589	Ltbp2	16997	0,883767425	7,300107527	0,000129115
A_55_P2176729	NM_007453	Prdx6	11758	0,883259602	10,91191398	4,41E-05
A_55_P2098697	NM_009396	Tnfaip2	21928	0,882985775	7,799064516	8,13E-06
A_55_P1960238	NM_172659	Slc2a6	227659	0,882726174	9,298752688	0,001175823
A_55_P1999561	NM_001002842	Pram1	378460	0,882608108	8,242290323	0,000962983
A_51_P162437	NM_001081412	Bcr	110279	0,879077525	8,690849462	0,000405127
A_55_P2081530	NM_080559	Sh3bgrl3	73723	0,879056899	13,83156989	5,06E-05
A_51_P474459	NM_007707	Socs3	12702	0,87783357	9,726333333	0,000386038
A_55_P1957954	NM_009105	Rsu1	20163	0,876940967	9,144096774	0,000295087
A_51_P448664	NM_011539	Tbxas1	21391	0,874707681	8,562688172	0,000121297
A_55_P2092831	XM_001472097	Gm2008	100039018	0,872834282	10,76488172	0,001430571
A_51_P262721	AK005967	0610009L18Rik	66838	0,872638691	7,511989247	5,81E-10
A_51_P381260	NM_008761	Fxyd5	18301	0,872241821	11,68298925	0,000253155
A_55_P2081133	NM_008084	Gapdh	14433	0,871560455	11,45723656	1,65E-05
A_55_P1979341	NM_007806	Cyba	13057	0,871222617	11,82596774	7,71E-05
A_55_P2064996	NM_009798	Capzb	12345	0,870886913	9,38027957	9,61E-05
A_52_P59681	NM_008287	Hrsp12	15473	0,868976529	8,344075269	0,001384228
A_55_P2064507	NM_026322	Msra	110265	0,868430299	7,620430108	1,41E-09
A_55_P2070766	XM_001476070	Gm3241	100041267	0,867811522	10,81704301	0,004362423
A_52_P90363	NM_029803	Ifi27l2a	76933	0,867261735	13,84776344	0,022606236
A_55_P1973347	NR_002860	A130040M12Rik	319269	0,866740398	9,67255914	0,0010116
A_55_P2044653	NM_009999	Cyp2b10	13088	0,866355619	7,808516129	0,007588616
A_55_P2146254	NM_001112715	Ifitm1	68713	0,862216927	10,7465914	0,020170077
A_55_P2077055	NM_018746	Itih4	16427	0,860515647	6,595290323	0,001619808
A_55_P2039038	NM_001081750	Zfp664	269704	0,859838549	8,292698925	0,002650295
A_55_P2074206	NM_146128	Dlgap4	228836	0,857226885	9,113989247	2,70E-07
A_51_P452779	NM_133198	Pygl	110095	0,856921764	10,38095699	0,001693522
A_55_P1981494	AY989854	H2-Bl	14963	0,856892603	8,887354839	0,001956787
A_51_P389988	NM_016917	Slc40a1	53945	0,856600996	7,947333333	0,011009117
A_51_P193794	NM_008512	Lrp1	16971	0,855987909	7,33216129	6,03E-05
A_55_P1992099	NM_008982	Ptprj	19271	0,8559367	8,750225806	0,000131362
A_55_P2029574	NM_008898	Por	18984	0,855821479	8,798870968	3,16E-06
A_55_P2021398	XM_001473399	Gm2393	100039733	0,855498578	10,90866667	0,001990026
A_55_P2179413	NM_011150	Lgals3bp	19039	0,854616643	10,27933333	0,000385086
A_55_P2045136	AK153212	I830127L07Rik	546643	0,853322902	6,905935484	0,000882068
A_52_P84096	NM_001081274	Pgd	110208	0,851806543	9,194623656	6,65E-05
A_51_P272106	NM_007705	Cirbp	12696	0,850837838	8,104010753	0,001402959
A_55_P1998578	NM_030694	Ifitm2	80876	0,85037909	9,701172043	7,75E-05
A_55_P2086954	XM_001479991	Gm4372	100043336	0,849611664	10,87366667	0,001924294
A_66_P136801	NR_002864	Peg13	353342	0,849600996	7,816763441	1,46E-07
A_52_P354682	NM_029001	Elovl7	74559	0,849450213	6,784064516	7,88E-06
A_51_P390715	NM_011577	Tgfb1	21803	0,849328592	7,820967742	1,13E-05
A_55_P2095345	NM_001177302	Rara	19401	0,849305121	10,45258065	0,00036961
A_51_P405397	NM_007899	Ecm1	13601	0,84805761	11,68043011	0,004458763
A_55_P1957413	NM_019391	Lsp1	16985	0,846922475	13,20252688	0,000839415
A_55_P2176731	NM_007453	Prdx6	11758	0,845165718	10,34212903	6,94E-05
A_55_P2023076	NM_023142	Arpc1b	11867	0,844041963	11,03565591	2,45E-06
A_55_P2059342	NM_010684	Lamp1	16783	0,842654339	11,87770968	8,58E-07
A_51_P312121	NM_011723	Xdh	22436	0,842088905	7,42283871	1,12E-05

A_52_P13815	NM_010686	Laptm5	16792	0,834766003	8,477064516	0,000575613
A_51_P146149	NM_008437	Napsa	16541	0,831389758	8,676956989	4,69E-05
A_51_P478895	NM_172894	Ppp6r1	243819	0,830451636	7,437	1,79E-07
A_55_P2041584	XM_001003844	F420015M19Rik	619329	0,829290185	8,739602151	3,51E-05
A_52_P196105	NM_175274	Ttyh3	78339	0,829066856	9,08916129	0,000215874
A_52_P456640	NM_010208	Fgr	14191	0,82586202	8,545032258	0,000157349
A_55_P2074636	NM_172723	Adap1	231821	0,824610953	9,885247312	1,48E-05
A_55_P1993940	NM_080559	Sh3bgrl3	73723	0,824405405	11,85797849	7,51E-05
A_52_P476754	NM_176860	Ubash3b	72828	0,823825747	9,350569892	0,000842538
A_51_P494675	NM_028071	Cotl1	72042	0,823749644	12,03619355	0,000225802
A_55_P2172852	NM_025760	Ptplad2	66775	0,823549075	8,718612903	0,000350985
A_52_P515247	NM_007486	Arhgdib	11857	0,823428165	13,02104301	0,000138102
A_55_P1977653	NM_001081390	Palld	72333	0,821783073	6,750365591	0,000421236
A_55_P1965772	NM_001163337	Atp2a3	53313	0,819154339	9,957010753	0,000174692
A_55_P1965655	NM_026849	Mtmr14	97287	0,818191323	6,612612903	2,17E-05
A_55_P2063505	XM_001479508	Gm4235	100043109	0,818157183	10,29732258	0,000882336
A_55_P1956160	NM_001081032	Gm8909	667977	0,8156899	7,269569892	0,021080845
A_52_P365011	NM_010194	Fes	14159	0,81247155	8,657430108	0,000108588
A_55_P2020371	XM_975056	Gm11543	665174	0,810564011	6,812333333	0,05985704
A_55_P1960479	XM_001475709	Gm3114	100041051	0,810370555	10,33202151	0,001075404
A_55_P1991783	XM_001477578	Gm7149	635092	0,810058321	10,75172043	0,002710164
A_55_P1972872	NM_001005858	I830012O16Rik	667370	0,807228307	8,637258065	0,045216382
A_55_P2462377	NM_008740	Nsf	18195	0,806339972	7,738182796	1,65E-06
A_51_P455807	NM_133838	Ehd4	98878	0,80587909	10,58676344	1,95E-05
A_55_P2183208	NM_001045532	Prl2c1	666317	0,804463727	6,823268817	0,000618899
A_51_P341465	NM_009970	Csf2ra	12982	0,804238976	9,626774194	0,001092063
A_55_P2055638	AK168707	Gsn	227753	0,803918208	10,09515054	0,001018524
A_55_P2054409	NM_011089	Pira2	18725	0,803263158	8,266892473	0,000409749
A_55_P1996826	NM_026818	Cilp2	68709	0,801522048	6,630086022	0,080795672
A_55_P2106690	NM_011527	Tal1	21349	0,801254623	8,818978495	0,000474739
A_51_P111962	NM_001141922	Bean1	65115	0,800476529	7,061569892	0,000818354
A_55_P2019833	XM_001477941	Gm3790	100042323	0,799564011	10,66158065	0,002201141
A_51_P329928	NM_013750	Phlda3	27280	0,79792532	8,414645161	0,001945276
A_55_P2175880	NM_019946	Mgst1	56615	0,797788051	8,247956989	0,001429829
A_55_P2144110	NM_001045964	Mink1	50932	0,796048364	8,643204301	8,17E-05
A_55_P2076580	NM_130864	Acaa1a	113868	0,796027738	9,742924731	1,35E-05
A_55_P2172470	NM_022029	Nrgn	64011	0,796007112	11,47164516	0,002652856
A_52_P563617	NM_133772	Ssbp4	76900	0,79565505	8,69083871	2,77E-07
A_51_P288138	NM_008039	Fpr2	14289	0,793541252	8,834946237	0,011523902
A_55_P1955747	NR_002890	Gm12070	654472	0,790371266	11,57163441	1,13E-05
A_55_P2065991	NM_016740	S100a11	20195	0,789793741	9,895806452	0,003130199
A_55_P2007768	NR_033633	Gm8709	667572	0,789067568	11,25686022	7,05E-05
A_51_P140942	NM_018739	Rp9	55934	0,788075391	8,660860215	6,61E-05
A_52_P606774	NM_021420	Stk4	58231	0,78790256	9,394827957	9,77E-05
A_51_P326191	NM_009251	Serpina3g	20715	0,786521337	8,600623656	0,020360886
A_51_P281089	NM_011313	S100a6	20200	0,786317212	12,2034086	0,004137145
A_51_P308590	NM_134084	Ppif	105675	0,783972262	7,15127957	5,10E-05
A_51_P321150	NM_017372	Lyz2	17105	0,783461593	9,698978495	0,00613565
A_55_P1994042	NM_001139519	Zbp1	58203	0,78234495	9,856774194	0,003078741
A_55_P1952882	AF233647	Cyp4f18	72054	0,781921764	11,02427957	0,000924496

A_55_P2004016	NM_030209	Crispld2	78892	0,780225462	7,958666667	0,034698761
A_55_P2022585	NM_011093	Pira6	18729	0,779806543	9,822333333	0,000123143
A_55_P2109857	NM_009061	Rgs2	19735	0,778459459	10,95741935	0,001581864
A_52_P12806	NR_003623	Gm5069	277333	0,777433144	10,88412903	2,23E-06
A_55_P2068248	XM_001473755	Gm2488	100039907	0,777237553	9,962236559	0,00128669
A_55_P2030638	NM_026725	Dusp23	68440	0,777004267	7,138419355	0,00086552
A_55_P2124097	NR_023357	Gm6981	629557	0,770519915	10,65383871	4,49E-05
A_51_P173100	NM_145925	Pttg1ip	108705	0,76956899	11,20139785	0,00701005
A_55_P2075731	XM_001475177	Gm2943	100040748	0,769330725	10,48289247	0,008514124
A_52_P363216	NM_133219	Gcnt2	14538	0,767438834	8,051387097	0,000750241
A_55_P2055819	NM_001037722	Adam15	11490	0,766817212	8,825892473	0,000295239
A_55_P2472435	NM_018734	Gbp3	55932	0,765931721	8,076924731	0,003787498
A_55_P2079561	NM_011090	Lilra6	18726	0,765577525	10,56353763	0,003115063
A_51_P202633	NM_015766	Ebi3	50498	0,765014225	8,089247312	1,68E-05
A_55_P2092826	NM_010730	Anxa1	16952	0,763763158	10,76946237	0,013177706
A_52_P190973	NM_009502	Vcl	22330	0,762882646	7,456182796	2,95E-05
A_55_P2106666	NM_009624	Adcy9	11515	0,761007112	7,196795699	0,000352607
A_55_P2141884	NM_178598	Tagln2	21346	0,759503556	9,970967742	0,000198339
A_55_P1961760	XM_001476058	Gm3237	100041259	0,759480085	10,52258065	0,006638879
A_51_P401668	NM_010686	Laptm5	16792	0,759418208	9,377741935	0,002280064
A_55_P2124528	NM_009795	Capns1	12336	0,759290896	10,55343011	4,86E-06
A_52_P72237	NM_009609	Actg1	11465	0,758003556	10,44377419	0,000964586
A_55_P1959425	NM_030696	Slc16a3	80879	0,757928876	9,019451613	0,001898622
A_52_P483336	NM_007641	Ms4a1	12482	0,757860597	9,257870968	0,001965389
A_51_P513254	NM_009007	Rac1	19353	0,757219061	10,44262366	0,000222323
A_55_P2171463	NM_009145	Nptn	20320	0,75550569	11,61836559	0,003871915
A_55_P2066613	NM_008879	Lcp1	18826	0,754844239	9,043365591	0,001103145
A_55_P2000853	NM_175184	Mvb12b	72543	0,754699858	8,543408602	0,001073145
A_55_P2121662	NM_011952	Mapk3	26417	0,753889047	7,739763441	3,03E-07
A_55_P2022251	NM_009878	Cdkn2d	12581	0,753541963	10,10264516	1,45E-05
A_51_P463120	NM_025760	Ptplad2	66775	0,753516358	9,729677419	0,001593993
A_55_P1998471	NM_009114	S100a9	20202	0,753411807	15,39022581	0,057309385
A_51_P502456	NR_004446	H2-K2	630499	0,752894026	11,99265591	0,008991302
A_51_P183812	NM_011410	Slfn4	20558	0,751613798	8,542268817	0,061671055
A_55_P2115257	NM_001037997	Fert2	14158	0,751520626	6,913247312	0,033712947
A_55_P2067505	NM_030696	Slc16a3	80879	0,751435989	8,942548387	0,001515996
A_52_P282762	NM_010851	Myd88	17874	0,750991465	8,209247312	1,97E-05
A_55_P2002963	NM_009898	Coro1a	12721	0,750221195	12,84963441	0,000996135
A_52_P635338	NM_010194	Fes	14159	0,750123755	9,534451613	0,000789401
A_55_P2029902	NM_181584	Gab3	210710	0,749216216	9,717150538	0,002082588
A_55_P1967330	NR_003623	Gm5069	277333	0,747854196	9,934172043	4,62E-05
A_55_P2068892	NM_010559	Il6ra	16194	0,747337127	10,32612903	0,000877019
A_51_P406796	NM_013873	Sult4a1	29859	0,745229018	6,394451613	6,14E-05
A_52_P527800	NM_145158	Emilin2	246707	0,74335633	10,16417204	0,004947969
A_55_P2082841	XM_001474255	Gm2658	100040199	0,743285917	10,17101075	0,00264143
A_55_P2052834	NM_010734	Lst1	16988	0,742192745	11,50219355	0,000890141
A_66_P112305	NM_053214	Myo1f	17916	0,741521337	9,160817204	0,003253001
A_51_P327295	NM_021473	Akr1a1	58810	0,740761024	9,558580645	2,17E-05
A_55_P2039439	NM_031868	Ppp1ca	19045	0,74028165	11,75446237	4,24E-05
A_55_P1961761	NM_007831	Dcc	13176	0,73978734	10,44027957	0,008457011

A_55_P1965150	NM_021549	Pnkp	59047	0,739318634	9,791064516	0,000421692
A_52_P132165	NM_053262	Hsd17b11	114664	0,738192745	8,056010753	9,94E-08
A_51_P279163	NM_172285	Plcg2	234779	0,736510669	10,00193548	4,66E-05
A_55_P2146185	NM_011302	Rs1	20147	0,734694168	10,89227957	0,021684838
A_55_P2142873	NM_001045964	Mink1	50932	0,732377667	8,522225806	0,000247234
A_55_P1977583	NM_013469	Anxa11	11744	0,7323734	8,907064516	0,000427086
A_55_P2037584	NM_133977	Trf	22041	0,732310811	7,974698925	0,009823789
A_66_P119034	NM_013737	Pla2g7	27226	0,731698435	10,00905376	0,004577914
A_55_P2034300	NM_001168256	Tmem40	94346	0,730658606	12,24375269	0,019293775
A_51_P256202	NM_022325	Ctsz	64138	0,730541963	10,54234409	0,000469576
A_51_P384629	NM_009983	Ctsd	13033	0,729682788	11,50787097	0,000658545
A_55_P2134148	NM_029354	Mzt2	72083	0,729421764	7,002505376	0,002614674
A_55_P2144850	NM_011186	Psmb5	19173	0,72921266	11,49960215	5,07E-06
A_66_P123055	NR_033535	Gm10845	100038734	0,728858464	9,354215054	0,014617728
A_55_P1984284	NM_008084	Gapdh	14433	0,728300142	11,2006129	0,000150235
A_55_P1966194	NM_019549	Plek	56193	0,728273115	11,6005914	0,002513806
A_51_P493558	NM_145417	Rnpep	215615	0,727534139	8,20416129	2,14E-05
A_55_P2118037	NM_007547	Sirpa	19261	0,726820768	8,207698925	0,000186459
A_51_P462448	NM_001037741	Gpx4	625249	0,726397582	12,3612043	0,000239719
A_55_P2017944	NM_026695	Etfb	110826	0,726177809	9,479397849	3,66E-05
A_51_P234864	NM_178639	Sfxn5	94282	0,726071835	7,414892473	0,00042455
A_51_P403334	NM_024192	Cuedc2	67116	0,725487909	9,058322581	6,10E-06
A_52_P199614	NM_001146022	Wdfy4	545030	0,725052632	9,340870968	0,000581919
A_52_P613498	NM_026127	4833420G17Rik	67392	0,725021337	8,128688172	0,000843654
A_55_P2134790	NM_001024945	Qsox1	104009	0,724911095	9,529021505	0,000157681
A_51_P450100	NM_026124	1110008F13Rik	67388	0,722449502	9,695483871	4,55E-06
A_55_P2174743	NM_018747	Akap7	432442	0,722163585	8,499677419	0,002754781
A_55_P2041070	NM_001083927	Tle3	21887	0,721933144	9,074967742	6,68E-05
A_51_P254656	NM_008230	Hdc	15186	0,721598862	9,954333333	0,024561999
A_55_P2136832	NR_023357	Gm6981	629557	0,720733997	11,08764516	3,44E-05
A_55_P1998416	NM_008330	Ifi47	15953	0,719778805	11,34104301	0,00038835
A_55_P2110713	NM_007585	Anxa2	12306	0,719435989	11,85008602	0,003905475
A_55_P1998942	NM_145211	Oas1a	246730	0,719027027	8,830129032	0,027074918
A_51_P109171	NM_177614	Os9	216440	0,718903272	8,625451613	4,02E-07
A_51_P237688	NM_133697	Smim14	68552	0,715726885	9,060397849	0,005254073
A_52_P400999	NM_020260	Arhgap31	12549	0,715251778	6,57044086	0,000218426
A_55_P2177539	NM_011434	Sod1	20655	0,714256046	11,33780645	4,45E-08
A_55_P1962747	NM_207105	H2-Ab1	14961	0,714093172	11,86515054	0,01731246
A_51_P396854	NM_019502	Timm10b	14356	0,713672831	9,547763441	9,09E-05
A_51_P433824	NM_009415	Tpi1	21991	0,713630156	9,881150538	0,000116019
A_55_P2035613	NM_027154	Tmbim1	69660	0,713421764	7,684774194	0,014049849
A_51_P225793	NM_175181	Prr5l	72446	0,712371266	9,644569892	0,00181838
A_55_P1992455	NM_011696	Vdac3	22335	0,711537696	9,900064516	0,000562572
A_55_P2105271	NM_001114088	Pdlim7	67399	0,711320057	9,74383871	0,005495828
A_51_P470079	NM_010555	Il1r2	16178	0,711105263	8,07572043	0,04137318
A_51_P295215	NM_172943	Alkbh5	268420	0,709457326	9,164935484	0,00118487
A_51_P460954	NM_009139	Ccl6	20305	0,709323613	11,19388172	0,006713351
A_51_P492346	NM_026685	Tmem174	68344	0,708921764	7,149193548	2,32E-05
A_51_P270733	NM_009303	Syngr1	20972	0,708428876	8,142064516	0,014128549
A_51_P238786	NM_008562	Mcl1	17210	0,707929587	11,46219355	0,000229594

A_55_P2064043	NM_009851	Cd44	12505	0,707463727	8,362086022	1,57E-05
A_51_P374869	NM_013850	Abca7	27403	0,706982219	10,2913871	4,22E-05
A_55_P2041457	XM_001475948	Gm3195	100041198	0,706736131	9,901086022	0,004144236
A_51_P469008	NM_010119	Ehd1	13660	0,706387624	10,92691398	0,007846472
A_55_P1961188	NR_023357	Gm6981	629557	0,705187055	9,830537634	0,000152478
A_66_P139546	NM_008344	Igfbp6	16012	0,704337838	7,71744086	0,005841505
A_55_P2120141	XM_001480410	Gm4522	100043565	0,703478663	10,45496774	0,015480078
A_55_P2238406	NR_028126	6330407A03Rik	70720	0,702760313	7,384817204	0,001067807
A_52_P592909	NM_026384	Dgat2	67800	0,70059744	7,859569892	0,006110113
A_55_P2181738	NM_013590	Lyz1	17110	0,700576102	12,21576344	0,010643953
A_52_P351816	NM_010578	Itgb1	16412	0,700381223	6,980397849	4,21E-05
A_51_P481238	NM_027293	Dopey2	70028	0,700182077	8,395870968	0,000215142
A_55_P2158701	NM_175654	Hist1h4d	319156	0,699145092	9,533677419	0,003378118
A_55_P2184364	NM_020579	B4galt3	57370	0,698076102	7,798774194	0,003055208
A_55_P1979457	NM_016764	Prdx4	53381	0,697911095	7,272537634	2,70E-06
A_52_P135873	XM_001475453	Gm2744	100040381	0,697780228	9,232591398	0,024564527
A_52_P187132	NM_011732	Ybx1	22608	0,697397582	9,875129032	0,004001058
A_52_P318438	NM_008667	Nab1	17936	0,696807255	7,266817204	8,20E-10
A_55_P1981829	NM_001004193	Rhox8	434768	0,696542674	10,1928172	0,021329793
A_55_P2146560	NM_207105	H2-Ab1	14961	0,696448791	11,91726882	0,019030942
A_51_P505823	NM_028013	Endod1	71946	0,696375533	8,6	0,007680669
A_55_P2103561	NM_007453	Prdx6	11758	0,696006401	11,84454839	0,001615123
A_55_P2007022	NM_011925	Cd97	26364	0,69516643	12,16630108	0,005935623
A_51_P228768	NM_011409	Slfn3	20557	0,693315078	6,928827957	0,02322085
A_52_P59579	NM_026669	Tmbim6	110213	0,693182077	9,531301075	4,55E-05
A_55_P2054743	NM_172507	Sh3bgrl2	212531	0,692231152	7,470860215	0,000516704
A_55_P2013113	NM_007523	Bak1	12018	0,691972262	8,624075269	8,41E-05
A_55_P1987770	NM_025498	Psenen	66340	0,690275249	11,92493548	0,00075651
A_51_P328652	NM_133348	Acot7	70025	0,689943101	9,12488172	3,44E-05
A_55_P1957038	NM_181796	Gstp2	14869	0,689674964	11,38224731	4,74E-06
A_51_P323620	NM_144543	Thyn1	77862	0,689362731	7,777946237	1,56E-07
A_55_P1960999	NM_011082	Pigr	18703	0,689321479	10,8783871	0,005312585
A_51_P324572	NM_009818	Ctnna1	12385	0,688861309	8,992494624	0,000749617
A_55_P1952950	NM_001110132	Cic	71722	0,688820768	10,23646237	0,001617132
A_52_P604629	NM_153287	Csrnp1	215418	0,68840825	8,077870968	0,000133549
A_55_P2066116	NM_033601	Bcl3	12051	0,687795875	9,005225806	0,000312267
A_55_P1987264	NM_010312	Gnb2	14693	0,686686344	11,14429032	0,001293573
A_55_P2020378	NM_133838	Ehd4	98878	0,686378378	7,455150538	6,56E-06
A_55_P2002103	NM_001142701	Hmha1	70719	0,686342817	12,24780645	0,002181944
A_55_P1990785	NM_027678	Zranb3	226409	0,685843528	7,651935484	0,015094105
A_66_P134453	NM_001033780	I830077J02Rik	433638	0,68347155	7,766784946	0,001001339
A_55_P1959818	NM_001168256	Tmem40	94346	0,682110242	12,01733333	0,02377068
A_55_P1956130	BC025170	LOC68395	68395	0,681012091	11,99882796	0,005734005
A_51_P156857	NM_134133	Smim3	106878	0,680327169	10,70144086	0,030054443
A_55_P1967504	NM_145215	Abhd11	68758	0,680290185	7,739043011	3,28E-05
A_55_P2068247	XM_001473755	Gm2488	100039907	0,679066856	9,666806452	0,004538583
A_52_P576442	NM_146057	Dap	223453	0,678645092	11,25136559	0,004789733
A_51_P291460	NR_003630	Gm6498	624367	0,678532717	10,77611828	7,92E-05
A_55_P1994258	NM_008084	Gapdh	14433	0,678219772	8,423290323	3,02E-05
A_52_P116134	NM_009656	Aldh2	11669	0,677265292	10,27721505	0,050300821

A_55_P1954921	NM_013908	Fbxw5	30839	0,67721835	8,57244086	9,28E-06
A_52_P466090	NM_018851	Samhd1	56045	0,676214083	8,931483871	0,001680891
A_51_P272876	NM_001160378	Fam46a	212943	0,676048364	8,15372043	0,00025976
A_52_P578790	NM_013739	Dok3	27261	0,6749367	8,521204301	8,48E-05
A_51_P517870	NM_198429	Nfatc1	18018	0,67473542	8,073096774	0,000933913
A_55_P2052016	NM_030209	Crispld2	78892	0,673617354	7,70783871	0,05283575
A_52_P195018	NM_139206	Arap3	106952	0,673507112	8,500172043	0,000219336
A_52_P253004	NM_022327	Ralb	64143	0,673426031	8,478107527	2,15E-05
A_55_P2027213	NM_001163615	Krtap20-2	622935	0,673123755	7,050182796	3,96E-05
A_55_P1959953	NM_183162	Helz2	229003	0,673029872	9,383634409	0,002669683
A_55_P2083411	NM_024444	Cyp4f18	72054	0,671365576	10,76773118	0,007595059
A_55_P2045741	NM_011815	Fyb	23880	0,670537696	7,72044086	0,003254336
A_51_P241943	NM_001081168	Sap30l	50724	0,670311522	10,00089247	0,000127737
A_55_P2003996	NM_008207	H2-T24	15042	0,669774538	8,493096774	0,000315199
A_55_P2114697	NM_008103	Gcm1	14531	0,668967283	9,874086022	0,011419947
A_55_P2022211	NM_026162	Plxdc2	67448	0,668837838	6,934193548	0,027550832
A_55_P2028847	XM_001476302	Gm9468	669759	0,668523471	9,951311828	0,016135096
A_55_P1965725	NM_008084	Gapdh	14433	0,668210526	8,522483871	1,54E-05
A_55_P2251181	AK013795	2900076A13Rik	73002	0,667545519	7,667849462	0,000285977
A_51_P297679	NM_008225	Hcls1	15163	0,667142248	11,28210753	0,000738018
A_55_P1987201	NM_009794	Capn2	12334	0,66677027	9,174548387	0,000309671
A_55_P1952166	NM_007880	Arid3a	13496	0,666550498	8,298741935	1,01E-05
A_51_P260548	NM_009149	Glg1	20340	0,666445235	8,697763441	0,000180328
A_52_P84901	NM_133891	Slc44a1	100434	0,666421764	8,560376344	0,02712252
A_55_P1957459	NM_013532	Lilrb4	14728	0,665544808	10,00173118	0,028596675
A_51_P338600	NM_025387	Tmem14c	66154	0,665489331	7,436634409	0,02355622
A_55_P1959973	NM_175539	Dcaf12l2	245403	0,664453058	8,273967742	0,027209136
A_51_P278034	NM_027356	Ufsp1	70240	0,664447368	7,188752688	0,001066413
A_52_P56751	NM_008879	Lcp1	18826	0,66435633	10,07631183	0,004133295
A_52_P508089	NM_011179	Psap	19156	0,664301565	10,07674194	0,003185551
A_52_P516021	NM_011201	Ptpn1	19246	0,662780228	8,429741935	8,07E-05
A_55_P2096127	XM_001473875	Gm2527	100039971	0,661383357	9,496946237	0,00352992
A_51_P474169	NM_144883	Proser2	227545	0,661098151	8,498666667	0,018776549
A_52_P322141	NM_001081291	Ccdc88b	78317	0,660458748	10,38	0,00128643
A_55_P2135200	NM_181542	Slfn10-ps	237887	0,659090327	6,96527957	7,68E-06
A_55_P2072985	NM_001076554	Sptan1	20740	0,658889047	8,986172043	0,003433785
A_51_P490305	NM_023065	Ifi30	65972	0,65875889	8,932752688	0,000238945
A_52_P514061	NM_011061	Padi4	18602	0,657669275	6,776569892	0,000622618
A_52_P353417	NM_010299	Gm2a	14667	0,656736842	7,420827957	7,39E-06
A_55_P2125208	NM_001172205	Arid5a	214855	0,655975818	10,13906452	0,00252535
A_55_P2115136	NM_028732	4632428N05Rik	74048	0,655685633	8,793096774	0,000222919
A_55_P2119463	NM_010281	Ggh	14590	0,655541963	8,101666667	0,001307157
A_55_P1957378	NM_011676	Unc119	22248	0,654915363	9,360483871	3,16E-05
A_55_P2070913	NM_025974	Rpl14	67115	0,654671408	13,26176344	0,006632143
A_55_P2171788	XM_001474084	Gm2598	100040097	0,654403983	9,943655914	0,027609859
A_51_P440399	NM_012060	Bcap31	27061	0,653917496	9,469268817	0,00052292
A_55_P2062936	NM_013855	Abca3	27410	0,653513514	8,241	1,84E-06
A_55_P2091691	NM_019837	Nudt3	56409	0,652644381	11,14315054	0,017896925
A_51_P382849	NM_010330	Emb	13723	0,651901138	8,53255914	0,002895203
A_51_P222475	NM_053252	Ehbp1l1	114601	0,651894737	8,391817204	6,44E-08

A_55_P2099560	NM_031159	Apobec1	11810	0,651534851	8,995215054	0,001422832
A_51_P507942	NM_029097	Atp13a2	74772	0,651456615	9,457258065	0,000701552
A_51_P384148	NM_013649	Ryk	20187	0,650620199	6,359645161	0,003170152
A_66_P115061	NM_025695	Smc6	67241	0,650334282	7,22083871	0,000716997
A_55_P2004562	NM_001170851	Klra2	16633	0,649206259	6,684204301	0,025345741
A_51_P227718	NM_145149	Rasgrp4	233046	0,649153627	6,565215054	9,39E-08
A_52_P70854	NM_020507	Tob2	57259	0,649038407	7,672860215	4,18E-07
A_55_P2033445	NM_011610	Tnfrsf1b	21938	0,648513514	6,901021505	2,81E-06
A_55_P2151986	NM_001038664	Gngt2	14710	0,648479374	9,542731183	0,002285717
A_51_P143805	NM_025339	Tmem42	66079	0,646388336	7,784698925	0,00011145
A_51_P247249	NM_009662	Alox5	11689	0,646043385	7,89316129	0,001595499
A_51_P451346	NM_011803	Klf6	23849	0,645870555	10,30712903	0,001193027
A_55_P2122334	NM_008688	Nfic	18029	0,64519559	7,37611828	0,000556341
A_52_P549827	NM_019946	Mgst1	56615	0,644582504	7,818946237	0,003369405
A_55_P1995175	AK010293	Sult4a1	29859	0,643755334	6,366182796	8,99E-05
A_51_P252677	NM_026738	1110007C09Rik	68480	0,643631579	10,60950538	0,000686692
A_66_P116252	NM_130447	Dusp16	70686	0,643541963	7,62111828	0,00128669
A_55_P1976928	NM_001009573	Unc13d	70450	0,643347084	8,727752688	2,70E-07
A_51_P392687	NM_011701	Vim	22352	0,643229018	10,70829032	0,011904608
A_66_P102733	NM_025455	Ccdc28b	66264	0,642793741	7,78083871	5,79E-09
A_51_P268343	NM_025857	Aagab	66939	0,641949502	8,198430108	1,66E-07
A_55_P2052425	NM_145482	Setd4	224440	0,641852774	7,502774194	2,45E-05
A_55_P1957353	XM_907302	Slfn13l	631406	0,641100284	8,955333333	0,00932952
A_55_P2074647	NM_144521	Snap47	67826	0,641042674	6,541655914	9,13E-07
A_51_P163953	NM_008741	Nsg2	18197	0,641034851	8,987860215	0,017186143
A_52_P517896	NM_028636	Man2c1	73744	0,640945946	8,439	5,97E-05
A_52_P590396	NM_019972	Sort1	20661	0,64047724	6,784784946	5,99E-06
A_55_P1971840	NM_010739	Muc13	17063	0,640265292	7,195774194	0,003050576
A_55_P2143219	NM_011242	Rasgrp2	19395	0,639594595	10,63692473	0,00632775
A_51_P418526	NM_027324	Sfxn1	14057	0,639148649	8,186569892	0,00010951
A_55_P2093874	NM_146131	Pbxip1	229534	0,638529872	10,6961828	0,000683651
A_66_P129048	NM_001190445	2610002J02Rik	67513	0,63842532	9,379731183	8,62E-05
A_51_P172853	NM_009841	Cd14	12475	0,637615932	7,488139785	0,027703833
A_55_P2019719	NM_145227	Oas2	246728	0,636404694	9,584860215	0,054901853
A_51_P293982	NM_153119	Plekho2	102595	0,635864154	7,888430108	0,001438497
A_55_P2106250	NM_025960	Trappc6a	67091	0,635745377	9,35144086	6,80E-06
A_52_P303176	NM_024461	1810037I17Rik	67704	0,63564367	6,655096774	0,025198261
A_55_P2151116	NM_144822	Micu1	216001	0,634940256	10,32601075	4,68E-05
A_55_P2071329	NM_019566	Rhog	56212	0,634551209	11,77180645	3,06E-06
A_52_P316933	NM_172507	Sh3bgrl2	212531	0,634152205	9,765225806	0,01042668
A_55_P2097279	NM_011095	Pirb	18733	0,632558321	10,67548387	0,032785486
A_51_P193146	NM_028595	Ms4a6c	73656	0,632392603	8,30672043	0,001410465
A_55_P2037585	NM_133977	Trf	22041	0,632307966	7,090021505	0,005910198
A_66_P114784	NM_013737	Pla2g7	27226	0,632034139	7,546172043	0,000689841
A_55_P2075140	NM_022433	Sirt3	64384	0,631648649	8,497602151	0,000464772
A_55_P2130970	NM_001163576	Parp10	671535	0,631179943	10,5983871	0,007740974
A_55_P1955227	NM_010261	Rabac1	14470	0,629533428	11,20965591	0,000102943
A_51_P330428	NM_007918	Eif4ebp1	13685	0,628771693	10,12036559	0,000664897
A_55_P2027999	NM_001146100	Hk1	15275	0,628554765	10,06466667	0,001544956
A_52_P288873	NM_029362	Chmp4b	75608	0,627536984	9,404698925	3,03E-06

A_55_P2072373	NM_010833	Msn	17698	0,626267425	11,30021505	0,000487404
A_55_P2024888	NM_021281	Ctss	13040	0,626157895	11,07407527	0,011292646
A_55_P2067362	AK171155	Dpep2	319446	0,625078236	6,639268817	0,000282916
A_55_P1955841	NM_008906	Ctsa	19025	0,624507112	10,8131828	0,005685984
A_51_P510849	NM_030018	Tmem50b	77975	0,623134424	8,625301075	7,10E-06
A_55_P1958246	NM_011552	Tcof1	21453	0,623128023	8,05655914	0,006890095
A_55_P2044045	NM_011732	Ybx1	22608	0,623103129	10,03211828	6,60E-05
A_51_P312437	NM_025522	Dhrs7	66375	0,622302276	9,381849462	0,007854051
A_55_P2046398	NM_144958	Eif4a1	13681	0,621213371	8,525193548	6,49E-05
A_55_P2386256	AK051660	D130062J10Rik	319386	0,620756046	7,778408602	0,000761792
A_51_P308681	NM_001081059	Mcur1	76137	0,619323613	7,404268817	7,18E-06
A_55_P1992959	NM_173019	Pfkfb4	270198	0,61844808	9,265677419	0,020234965
A_55_P2052166	NM_029332	Akap13	75547	0,61720128	7,851053763	0,001990026
A_51_P201480	NM_213659	Stat3	20848	0,616842105	9,574978495	0,004144236
A_51_P267239	NM_019980	Litaf	56722	0,616790896	11,50204301	0,007882429
A_55_P2007001	NM_023465	Ctnnbip1	67087	0,61678165	6,760193548	0,002233924
A_52_P507214	NM_013599	Mmp9	17395	0,616587482	7,017354839	0,005560645
A_51_P175771	NM_022009	Flii	14248	0,615766714	8,791333333	4,55E-06
A_55_P2020361	NM_197945	Lzts3	241638	0,615709104	9,861655914	0,049610392
A_52_P161495	NM_009744	Bcl6	12053	0,615424609	8,897903226	0,000722173
A_51_P261517	NM_011662	Tyrobp	22177	0,615084637	11,93805376	0,021972147
A_55_P2077866	NM_010918	Nktr	18087	0,614248933	8,077967742	4,22E-05
A_51_P365409	NM_028040	Rpusd4	71989	0,613819346	6,430731183	2,58E-15
A_55_P1963868	NM_153119	Plekho2	102595	0,613241821	11,41390323	0,000356925
A_55_P1981804	NM_010326	Gp1ba	14723	0,613086771	8,977043011	0,056982713
A_55_P2059606	NM_019963	Stat2	20847	0,611532006	8,50127957	0,007550013
A_55_P1964664	NM_139149	Fus	233908	0,610147937	8,755967742	0,012741645
A_51_P165330	NM_025796	Mrpl33	66845	0,610056899	10,72652688	0,002304948
A_55_P2136752	NM_029972	Ermn	77767	0,609917496	8,694258065	0,009189887
A_66_P124969	AK020834	A930009A15Rik	77798	0,609423186	6,403075269	0,001691984
A_51_P332201	NM_009976	Cst3	13010	0,608646515	12,06472043	0,004255398
A_55_P2222870	NM_139300	Mylk	107589	0,608295875	6,778967742	0,000231969
A_55_P2169445	NM_133829	Mfsd6	98682	0,60799431	8,375569892	1,22E-06
A_55_P2078088	NM_001164420	Pqlc1	66943	0,60735633	8,921956989	2,97E-07
A_55_P2049647	NM_001001892	H2-K1	14972	0,607078236	11,99494624	0,009716064
A_55_P2052799	NM_009806	Cask	12361	0,606997866	6,918408602	4,51E-05
A_55_P1955717	NM_008222	Hccs	15159	0,605175676	14,50253763	0,004651428
A_51_P243914	NM_018773	Skap2	54353	0,605088905	10,05125806	0,001888549
A_51_P460734	NM_011103	Prkcd	18753	0,604890469	8,390258065	9,08E-05
A_55_P2104572	NM_144905	6330416G13Rik	230279	0,604463727	7,270129032	0,000655102
A_55_P1954758	NM_001033217	Prickle1	106042	0,603928876	6,896913978	3,60E-06
A_52_P446457	NM_001163192	Ly6g6f	433099	0,603518492	6,912903226	0,004176047
A_55_P1956267	NM_018804	Syt11	229521	0,603194879	6,850172043	2,13E-06
A_52_P681310	NM_011113	Plaur	18793	0,602775249	8,468311828	0,004155249
A_55_P1980302	NM_025498	Psenen	66340	0,602747511	11,26876344	0,003630899
A_55_P2058942	NM_026316	Aldh3b1	67689	0,602290896	7,673505376	0,002124712
A_52_P38964	NM_001081962	Sap25	751865	0,601645092	8,250096774	5,89E-05
A_52_P467690	NM_175836	Sptbn1	20742	0,601279516	7,08727957	0,000600054
A_52_P542794	NM_001002004	2610507B11Rik	72503	0,60052276	9,568677419	0,000215874
A_52_P307761	NM_010311	Gnaz	14687	0,599466572	6,647258065	0,00023357

A_55_P2003221	NM_031247	Gimap3	83408	0,599455192	9,640827957	0,013116293
A_55_P2015426	NM_133728	Asnsd1	70396	0,599206259	8,120419355	2,15E-08
A_66_P134294	NM_011667	Uba1y	22202	0,599168563	7,070494624	0,088308042
A_52_P87964	NM_183423	Pla2g12a	66350	0,599098862	7,953860215	0,007499044
A_51_P258435	NM_026917	Zdhhc3	69035	0,59902845	6,772698925	0,002985829
A_55_P2088825	NM_053202	Foxp1	108655	0,598963727	9,360752688	0,011740048
A_55_P1968763	NM_199022	Shc4	271849	0,598888336	9,567903226	0,064859494
A_55_P2000062	NM_008390	Irf1	16362	0,598738976	10,31725806	0,012745106
A_55_P2016623	XM_204772	Gm5068	277089	0,598721195	8,585086022	0,011905493
A_51_P186476	NM_013612	Slc11a1	18173	0,598268137	7,934462366	0,001034577
A_55_P2035320	NM_017373	Nfil3	18030	0,597849929	7,688655914	0,001526126
A_55_P2057877	NM_001163554	Pou2f2	18987	0,597758179	7,264688172	2,14E-05
A_55_P2147101	NM_013757	Sytl4	27359	0,597633001	8,887956989	0,03473515
A_51_P194628	NM_173037	Tango6	272538	0,597251778	10,58254839	0,089635711
A_55_P2128734	NM_146131	Pbxip1	229534	0,596578947	10,56711828	0,000571387
A_51_P127915	NM_173742	Rnasek	52898	0,595705548	8,144107527	0,000637663
A_51_P479786	NM_013870	Smtn	29856	0,5954367	6,927946237	0,001229051
A_55_P1988158	NM_016903	Esd	13885	0,59514936	11,37808602	0,040444486
A_51_P131800	NM_007806	Cyba	13057	0,594654339	9,146376344	0,00037431
A_55_P2033215	NM_013877	Cabp5	29865	0,592369132	9,810505376	0,058757204
A_55_P2241299	NM_001025613	Otud7b	229603	0,591697724	7,298129032	0,003503358
A_55_P1996862	NM_011190	Psme2	19188	0,591423898	11,85627957	0,000848042
A_51_P218774	NM_026418	Rgs10	67865	0,591131579	12,28573118	0,004655977
A_51_P206346	NM_178606	Reep3	28193	0,59094808	7,789795699	2,42E-05
A_55_P2070352	NM_007782	Csf3r	12986	0,590184922	12,64345161	0,001082574
A_51_P413147	NM_008693	Klk1b3	18050	0,589777383	6,563548387	0,08909529
A_51_P440743	NM_009886	Celsr1	12614	0,589756757	6,512268817	5,86E-07
A_55_P1967533	NR_003623	Gm5069	277333	0,589512802	8,783548387	0,00021884
A_55_P2100824	XM_992161	LOC676689	676689	0,589299431	11,57848387	0,029477728
A_55_P2094901	AK046118	Phyhd1	227696	0,589153627	14,85047312	0,003638857
A_51_P350817	NM_009922	Cnn1	12797	0,588987198	6,718182796	0,026678189
A_51_P425048	NM_010392	H2-Q2	15013	0,588651494	10,67149462	0,01115472
A_55_P2045258	NM_010119	Ehd1	13660	0,588622333	8,050892473	0,007919634
A_51_P399985	NM_015742	Myo9b	17925	0,588433144	9,398741935	0,000400715
A_51_P121447	NM_054099	1110038F14Rik	117171	0,588386202	7,891967742	4,62E-05
A_55_P2154595	NM_017404	Mrpl39	27393	0,588113798	7,012172043	6,95E-08
A_55_P2144597	NM_001123370	9030025P20Rik	100041574	0,58790256	6,834688172	0,00014071
A_55_P1976224	NM_021273	Ckb	12709	0,587766003	6,682526882	0,000174386
A_51_P436727	NM_177231	Arrb1	109689	0,587256046	9,973752688	0,000703626
A_55_P2030433	NM_008155	Gpi1	14751	0,586677098	11,16687097	0,011492255
A_52_P679101	NM_011597	Tjp2	21873	0,586455903	6,987010753	0,000705307
A_52_P515036	NM_016865	Htatip2	53415	0,585741821	6,755021505	2,64E-05
A_55_P2142251	NM_054045	Hist2h3c2	97114	0,585376956	6,590731183	0,000263648
A_55_P2040539	NM_172618	Btbd9	224671	0,584751778	6,474602151	4,55E-06
A_55_P2146127	NM_029362	Chmp4b	75608	0,584039118	8,624666667	1,27E-06
A_55_P2165869	NM_009883	Cebpb	12608	0,583613087	9,776064516	0,023466055
A_55_P1994032	NM_013842	Xbp1	22433	0,582573969	9,521290323	0,00089868
A_65_P03442	NM_008828	Pgk1	18655	0,581388336	9,426978495	3,28E-05
A_55_P2065829	NM_199017	9230110C19Rik	234912	0,580600284	11,39919355	0,003433435
A_52_P683306	NM_146084	Fam13b	225358	0,580599573	7,351666667	8,63E-05

A_52_P276792	NM_053202	Foxp1	108655	0,580561166	7,926215054	0,001336057
A_52_P408757	NM_010188	Fcgr3	14131	0,580378378	8,028806452	0,000843654
A_51_P426754	NM_009673	Anxa5	11747	0,580157895	8,963473118	0,001456664
A_55_P2139027	NM_001163540	Plec	18810	0,580027027	8,952526882	0,00394846
A_55_P2039878	NM_010327	Gp1bb	14724	0,579960882	6,739526882	0,000619328
A_55_P2140212	XM_001474216	Gm2627	100040148	0,579082504	11,71482796	0,043714282
A_55_P2066778	NM_206536	AB124611	382062	0,578525605	8,13027957	0,000933913
A_52_P676956	NM_054096	Tirap	117149	0,578042674	6,727526882	1,83E-05
A_55_P2105888	NM_175935	G6pc3	68401	0,577359175	7,492193548	1,98E-05
A_55_P2143233	NM_011242	Rasgrp2	19395	0,577113798	10,24841935	0,006961555
A_51_P156274	NM_010736	Ltbr	17000	0,577071124	7,855806452	0,00068834
A_55_P1999349	NM_027722	Nudt4	71207	0,576644381	9,155677419	0,003856848
A_51_P151126	NM_013706	Cd52	23833	0,576455192	12,75952688	0,011738919
A_51_P148069	NM_001134829	Lpgat1	226856	0,576293741	6,898924731	2,84E-05
A_51_P347862	NM_134156	Actn1	109711	0,576223329	6,857763441	3,86E-05
A_51_P421664	NM_133739	Tmem123	71929	0,57598293	8,983677419	0,002510543
A_55_P1964068	NM_144793	Slc25a38	208638	0,5758101	8,434645161	2,98E-06
A_51_P115326	NM_011512	Surf4	20932	0,574277383	8,879215054	0,000295417
A_51_P457528	NM_007630	Ccnb2	12442	0,574108108	8,315817204	0,005679216
A_51_P153170	NM_029787	Cyb5r3	109754	0,574046942	7,641505376	0,000115079
A_51_P309158	NM_027840	Snx20	71607	0,574007112	10,431	0,002341089
A_55_P1978506	NM_207648	H2-Q6	110557	0,573698435	11,47333333	0,016517707
A_55_P2131379	M60419	Ybx1	22608	0,573682077	8,707215054	0,000379098
A_51_P453351	NM_172266	Lpgat1	226856	0,573677098	7,328526882	9,93E-05
A_51_P277336	NM_138741	Sdpr	20324	0,573472973	10,23631183	0,085625863
A_51_P369628	NM_133949	Ptov1	84113	0,573288051	6,483322581	3,07E-10
A_51_P499698	NM_026414	Asprv1	67855	0,573217639	8,51316129	0,058676714
A_55_P2151273	NM_001039176	Elovl1	54325	0,57320128	9,509043011	0,000808877
A_55_P2036280	NM_001128605	Psen2	19165	0,572349218	8,00783871	0,001471261
A_52_P358349	NM_026002	Mtdh	67154	0,57209175	8,840107527	0,000731572
A_55_P2129291	NM_146114	Dclre1c	227525	0,571458037	6,998655914	0,000214992
A_52_P148514	NM_152803	Hpse	15442	0,571428876	6,782053763	9,04E-05
A_51_P336161	NM_010764	Man2b1	17159	0,571204836	9,717086022	0,000594345
A_55_P1997275	NM_133216	Xpnpep1	170750	0,570501422	9,323	0,016227307
A_55_P2124273	NM_053214	Myo1f	17916	0,570404694	8,852344086	0,010486795
A_55_P2125049	AB359227	Gm11127	100529082	0,570229018	12,1182043	0,015363595
A_55_P1992208	NM_008155	Gpi1	14751	0,570031294	11,02373118	0,010437864
A_55_P2001233	NM_001162938	Pydc3	100033459	0,569762447	8,769569892	0,056362505
A_55_P2083997	NM_001081750	Zfp664	269704	0,569330725	8,486752688	0,013594135
A_55_P2007500	NM_001013749	Tmem151b	210573	0,568934566	6,59127957	0,000220487
A_55_P2024431	NM_008064	Gaa	14387	0,56886771	7,423752688	0,000444355
A_55_P2061737	NM_021278	Tmsb4x	19241	0,568630156	16,36377419	0,006234557
A_55_P2041121	NR_002841	Rn4.5s	19799	0,567369132	14,7926129	0,000249672
A_51_P342669	NM_023418	Pgam1	18648	0,567322902	9,204365591	0,001128078
A_55_P2117525	NM_001166410	Rbm3	19652	0,56666643	9,500053763	0,003629601
A_55_P2108690	NR_033633	Gm8709	667572	0,566482219	8,529591398	0,000329889
A_55_P2166501	NM_009851	Cd44	12505	0,566144381	8,440322581	0,002437626
A_52_P600318	NM_022813	Scamp2	24044	0,56613229	8,422365591	1,12E-05
A_51_P346668	NM_012057	Irf5	27056	0,565999289	7,62783871	1,80E-05
A_52_P426740	NM_023635	Rab27a	11891	0,565544808	6,54816129	2,15E-08

A_55_P2036567	NM_011691	Vav1	22324	0,565163585	10,41175269	0,002223083
A_55_P2019083	NM_001114334	Rps6kb1	72508	0,564058321	8,524978495	6,51E-05
A_65_P03728	NM_009177	St3gal1	20442	0,563275249	10,81588172	0,016484452
A_55_P1983448	NM_011311	S100a4	20198	0,563160028	10,52352688	0,03891614
A_52_P354744	NM_011401	Slc2a3	20527	0,563044097	10,38764516	0,036314007
A_52_P456134	NM_010046	Dgat1	13350	0,562276671	6,992139785	0,00012061
A_55_P2022434	NM_008155	Gpi1	14751	0,56190825	11,10021505	0,010093031
A_55_P2305010	AK043374	A730089K16Rik	320411	0,561872688	8,083870968	0,048222622
A_55_P2149763	NM_001025313	Tapbp	21356	0,561794452	9,659913978	0,000737984
A_52_P243249	NM_011952	Mapk3	26417	0,561519203	9,195010753	1,83E-05
A_51_P223443	NM_133655	Cd81	12520	0,561429587	10,62862366	0,034643183
A_55_P2083982	NM_013811	Dnah8	13417	0,560369844	6,588569892	2,32E-05
A_52_P350664	NM_153781	Pygb	110078	0,560327169	7,226956989	6,08E-05
A_55_P2130975	NM_010881	Ncoa1	17977	0,559389047	8,179	0,004095683
A_55_P2115068	NM_011186	Psmb5	19173	0,559332148	9,090849462	1,51E-05
A_55_P2021011	NM_030207	Sfi1	78887	0,558535562	6,634741935	6,96E-05
A_51_P393426	NM_011173	Pros1	19128	0,558440967	7,659666667	0,002854605
A_51_P495581	NM_030682	Tlr1	21897	0,557391181	7,168989247	0,000274813
A_55_P2014511	NM_007444	Amd2	100041585	0,55683926	7,711129032	0,0022442
A_51_P362054	NM_025983	Atp5e	67126	0,556483642	11,76168817	4,51E-05
A_51_P117618	NM_023154	Ethe1	66071	0,555704836	7,601752688	2,06E-06
A_65_P18433	NM_001024604	Ankrd28	105522	0,555539829	7,075516129	9,63E-05
A_55_P1991134	NM_008019	Fkbp1a	14225	0,555147937	10,1322043	0,011556651
A_55_P1990548	NM_145476	Tbc1d22a	223754	0,554889758	8,942946237	0,002476767
A_55_P2045026	NM_029657	Mgrn1	17237	0,554624467	8,806419355	0,000732854
A_52_P327664	NM_153564	Gbp5	229898	0,554463727	7,481344086	0,013052799
A_51_P121891	NM_009008	Rac2	19354	0,554320057	10,86591398	0,009578913
A_66_P124549	NM_001159551	H13	14950	0,553766714	9,667	0,003011732
A_55_P2091551	NM_146011	Arhgap9	216445	0,553176387	10,45968817	0,005985323
A_65_P19784	NM_080443	Asb7	117589	0,552475818	7,019709677	0,000961593
A_55_P1996299	NM_001039055	Pofut1	140484	0,552451636	7,928795699	2,89E-07
A_55_P2107347	NM_013835	Trove2	20822	0,552412518	7,548817204	0,001857698
A_51_P220135	NM_016791	Nfatc1	18018	0,551748222	7,089709677	1,13E-05
A_51_P503542	NM_026712	Zfp414	328801	0,551384068	7,998946237	0,000192287
A_51_P203675	NM_021407	Trem3	58218	0,550754623	7,221655914	0,000611767
A_55_P2143516	XM_001473058	Gm2291	100039527	0,550509957	7,701408602	0,007609952
A_55_P1997509	NM_021397	Zbtb32	58206	0,55028165	6,86927957	0,00090985
A_51_P437144	NM_026556	Dynll2	68097	0,550237553	7,310612903	5,07E-06
A_51_P155313	NM_010362	Gsto1	14873	0,549814367	10,16211828	0,094410379
A_55_P2143204	NM_146131	Pbxip1	229534	0,549665718	7,935	0,000164217
A_55_P1972605	NM_009631	Adora3	11542	0,548035562	6,829387097	0,004708322
A_51_P449325	NM_008206	H2-Oa	15001	0,547750356	8,605107527	0,000749504
A_51_P199608	NM_178869	Ttll1	319953	0,547571124	6,771333333	9,96E-05
A_55_P2183597	NM_198664	Tbc1d2	381605	0,547416074	6,993473118	0,003531413
A_52_P279425	NM_032465	Cd96	84544	0,546596017	7,256978495	0,000104879
A_55_P1994175	NM_027351	Ppil3	70225	0,54605192	8,045913978	0,002258409
A_55_P2016462	NM_021274	Cxcl10	15945	0,545788762	7,262967742	0,029822826
A_55_P2048912	NM_001033308	Themis2	230787	0,545628734	8,115419355	0,001687306
A_51_P344947	NM_029576	Rab1b	76308	0,545497155	9,245946237	5,12E-05
A_66_P110633	NM_001168660	Apol9b	71898	0,544085349	6,591086022	0,011699188

A_55_P1982737	NM_016775	Dnajc5	13002	0,543352063	13,41687097	0,007951453
A_55_P1954092	XM_001473985	Gm2563	100040034	0,543285206	12,41134409	0,019927869
A_55_P2114269	NM_001100116	1700047I17Rik2	100101807	0,542911095	15,5902043	0,01184041
A_51_P150722	NM_010748	Lyst	17101	0,542852063	6,580473118	1,40E-05
A_51_P258620	NM_029391	Rab4b	19342	0,542667141	7,252483871	1,67E-08
A_55_P1987914	NM_008696	Map4k4	26921	0,542567568	7,50572043	2,12E-06
A_52_P282741	NM_011520	Sdc3	20970	0,542357752	6,647290323	0,00038522
A_55_P2011678	NM_001164557	Pdzk1ip1	67182	0,542207681	10,84311828	0,026943174
A_51_P295192	NM_010907	Nfkbia	18035	0,541981508	10,47056989	0,015224308
A_51_P202050	NM_008052	Dtx1	14357	0,541535562	8,906215054	0,021634454
A_66_P113487	NM_011862	Pacsin2	23970	0,541211238	10,27895699	0,01508196
A_66_P104815	NM_007899	Ecm1	13601	0,540882646	7,921537634	0,021713043
A_55_P2070241	NM_016703	Preb	50907	0,540378378	9,095978495	0,006008627
A_55_P2005753	NM_026968	Manbal	69161	0,538731152	6,76872043	1,79E-07
A_55_P2069721	NM_031165	Hspa8	15481	0,53801138	11,35551613	0,02668826
A_51_P293688	NM_026405	Rab32	67844	0,53794239	7,623408602	0,001108376
A_55_P2076489	NM_009007	Rac1	19353	0,537767425	11,2317957	0,000504461
A_51_P183995	NM_019458	Paf1	54624	0,537619488	7,979204301	8,07E-05
A_52_P609109	NM_145823	Pitpnc1	71795	0,537607397	6,757247312	3,44E-05
A_55_P2037081	NM_145078	2610305D13Rik	112422	0,537400427	6,729924731	0,016298209
A_52_P66371	NM_178420	Nlrx1	270151	0,537253201	8,134860215	0,045129523
A_66_P126138	NM_010881	Ncoa1	17977	0,536894737	7,344311828	0,000780916
A_55_P1987245	NM_011740	Ywhaz	22631	0,536832148	7,830946237	0,002781059
A_55_P2154107	NM_008103	Gcm1	14531	0,536755334	9,580043011	0,052986167
A_52_P570266	NM_013640	Psmb10	19171	0,53665505	10,54375269	0,019553942
A_55_P2018847	NM_001164735	Crlf2	57914	0,535138691	10,39544086	0,00391497
A_55_P2080603	NM_031165	Hspa8	15481	0,535102418	13,47156989	0,044896681
A_55_P1983006	NM_001077696	Hdac5	15184	0,535009957	10,49107527	0,000344283
A_51_P495242	NM_010689	Lat	16797	0,533950213	9,471021505	0,024338
A_51_P357341	NM_033444	Clic1	114584	0,533771693	11,48877419	0,001335444
A_55_P1993064	NM_033072	Mbd6	110962	0,533498578	8,062451613	3,90E-05
A_55_P2136572	NM_178626	Cdc42se2	72729	0,53348293	8,632860215	9,83E-05
A_55_P1956687	NM_001163753	Rab37	58222	0,533347795	7,867752688	4,92E-07
A_55_P1976754	NM_008907	Ppia	268373	0,532390469	13,69156989	0,001331077
A_51_P262171	NM_008326	Irgm1	15944	0,53227596	9,173741935	0,020260653
A_55_P1953489	NM_001033550	Lrrc8b	433926	0,532261735	7,123204301	0,006800532
A_55_P1965416	NM_015731	Atp9a	11981	0,532151494	7,811537634	0,046286344
A_51_P247359	NM_016933	Ptprcap	19265	0,531981508	11,0236129	0,056363845
A_55_P2076871	AK083328	Lef1	16842	0,531921764	10,72935484	0,030007898
A_55_P2136501	NM_021565	Midn	59090	0,53178734	9,013774194	0,001994909
A_51_P311904	NM_172498	Ptk2b	19229	0,53163798	8,609129032	8,09E-05
A_55_P2102322	NM_029887	Yif1b	77254	0,531418208	8,372322581	0,000592468
A_55_P2030080	NM_001017426	Kdm6b	216850	0,531390469	7,611774194	0,000676365
A_55_P1968789	NM_009025	Rasa3	19414	0,530734708	11,25098925	0,009739905
A_52_P653966	NM_019426	Atf7ip	54343	0,53062091	7,549688172	0,000272932
A_55_P2022082	NM_021366	Klf13	50794	0,530061878	8,71055914	0,005471333
A_55_P1978618	NM_013535	Grcc10	14790	0,529376245	10,25764516	0,000210563
A_52_P370311	NM_023625	Plbd2	71772	0,52929303	7,292	0,00038629
A_55_P2035877	NM_001190445	2610002J02Rik	67513	0,529084637	8,004623656	6,53E-06
A_51_P140321	NM_026779	Mocos	68591	0,528963727	7,202053763	0,000673794

A_52_P46419	NM_001038018	Grk6	26385	0,528366999	8,405	5,48E-05
A_51_P464918	NM_019453	Mefv	54483	0,528274538	8,592505376	0,022985724
A_51_P246653	NM_020008	Clec7a	56644	0,527485775	10,36019355	0,076184692
A_51_P519276	NM_025843	Ndufb7	66916	0,527458037	10,13164516	0,000114723
A_55_P1980267	NM_172257	Sidt2	214597	0,527353485	8,966193548	0,001232832
A_55_P2040838	NM_001166672	Gm14548	100038909	0,527100996	9,139150538	0,038915775
A_51_P487360	NM_016677	Hpcal1	53602	0,526288762	8,777451613	0,007854051
A_55_P2009861	XM_001472138	Gm2015	100039039	0,526165718	10,00830108	0,009006874
A_51_P203182	NM_138310	Apobr	171504	0,525849929	7,522365591	0,002592016
A_51_P220993	NM_011072	Pfn1	18643	0,525583926	9,806311828	0,00330134
A_51_P479808	NM_027418	Mapk6	50772	0,525233997	7,202086022	0,001843117
A_55_P1979463	NM_012021	Prdx5	54683	0,525133713	9,988795699	0,026293741
A_55_P2041961	NM_007553	Bmp2	12156	0,525048364	6,767569892	0,046247492
A_55_P2074776	XM_993473	Gm8894	667952	0,524285917	15,86175269	0,001125687
A_51_P303238	NM_026775	Tmed10	68581	0,523897582	8,725075269	0,000200315
A_55_P2060966	NM_001163440	Mov10	17454	0,52384495	8,648086022	0,002776116
A_55_P2037692	NM_001033403	Gm1968	328657	0,523758179	7,113806452	0,001003753
A_55_P2002517	NM_001077361	Fhl1	14199	0,523592461	7,045043011	0,001494524
A_55_P1988882	NM_001113487	Sep 09	53860	0,523111664	8,571870968	0,001713975
A_55_P2192961	NM_175648	Trim30b	244183	0,522957326	6,966537634	0,003326244
A_52_P422494	NM_145634	Cd300lf	246746	0,522738265	9,543258065	0,092178753
A_51_P111259	NM_026373	Cdk2ap2	52004	0,522607397	10,03231183	0,012849492
A_51_P212782	NM_008361	Il1b	16176	0,52254623	9,662	0,07589499
A_55_P2077618	NM_172753	Csgalnact1	234356	0,521876245	7,900677419	0,044823844
A_55_P1971074	NM_015804	Atp11a	50770	0,521585349	7,172182796	0,011335298
A_51_P189361	NM_027950	Osgin1	71839	0,521545519	7,718677419	0,000795711
A_55_P2128263	NM_001109691	Phf21a	192285	0,521417496	8,40183871	0,000166969
A_51_P419246	NR_028427	5830416P10Rik	381232	0,521257468	8,279290323	0,04357227
A_55_P1964364	NM_001012434	Kctd14	233529	0,520650782	17,07586022	0,001608986
A_51_P148355	NM_175433	Zfp710	209225	0,520030583	8,658419355	0,007755011
A_51_P416556	NM_026155	Ssr3	67437	0,520009957	8,676139785	0,000291818
A_55_P2046338	NM_001168514	Mapk14	26416	0,519926031	9,318967742	0,000239453
A_51_P146753	NM_007781	Csf2rb2	12984	0,519590327	8,55727957	0,006511755
A_55_P2391619	BG063913	AI449595	58901	0,519465149	6,490548387	0,039894512
A_55_P2035937	NM_001163663	Rab6a	19346	0,519413229	9,686086022	0,004002666
A_55_P2033510	NM_198931	Ppm1m	67905	0,518882646	8,296032258	0,000365199
A_55_P2146749	NM_026533	Rps13	68052	0,518522048	12,86290323	0,08713159
A_55_P1954986	NM_007922	Elk1	13712	0,518438122	16,84773118	0,002598426
A_55_P2095177	NM_013700	Usp5	22225	0,518130156	9,066591398	0,000457836
A_55_P2074536	NM_011151	Ppm1b	19043	0,517985775	9,096634409	0,000254486
A_51_P233603	NM_011969	Psma7	26444	0,517922475	9,701741935	0,001581864
A_52_P346458	NM_023764	Tollip	54473	0,517546942	8,390548387	0,044887345
A_66_P120567	NM_010730	Anxa1	16952	0,517461593	8,963322581	0,062285192
A_55_P1959595	NM_001161765	Fmo5	14263	0,515570413	7,336344086	0,010349632
A_55_P2133266	NM_028375	Cxx1c	72865	0,515509246	9,515032258	0,001714968
A_52_P467389	NM_023044	Slc15a3	65221	0,515460882	7,277204301	0,002767192
A_55_P1955239	NM_009096	Rps6	20104	0,51521266	11,81832258	0,003813044
A_55_P1960924	NM_053208	Egln2	112406	0,514892603	10,16222581	0,002798733
A_55_P2005570	NM_010160	Celf2	14007	0,513870555	9,473096774	0,002469656
A_52_P142208	NM_023423	Akirin1	68050	0,513364865	8,606483871	2,61E-06

A_55_P2086885	NM_001033219	Slc45a4	106068	0,513157895	6,74716129	0,000221332
A_51_P143200	NM_153523	Tcstv3	236219	0,513113087	6,452967742	0,000760516
A_51_P345663	NM_024281	Rrbp1	81910	0,512018492	7,669096774	0,000216372
A_55_P2082733	NM_007807	Cybb	13058	0,511999289	6,797870968	7,45E-05
A_55_P2042823	NM_001145859	Sh3bp2	24055	0,510982219	8,280430108	0,003640645
A_55_P2095342	NM_001177302	Rara	19401	0,510864865	8,263494624	0,004437913
A_55_P2171459	NM_009145	Nptn	20320	0,509667141	7,469225806	0,003575176
A_52_P114722	NM_001077705	Ptpn6	15170	0,509540541	9,72944086	0,00673409
A_55_P1993864	NM_008507	Sh2b3	16923	0,50951138	7,67783871	5,26E-08
A_51_P394802	NM_026640	Fam111a	107373	0,509503556	7,757043011	3,35E-05
A_55_P2151609	NM_011436	Sorl1	20660	0,509112376	9,040258065	0,040831942
A_55_P2096257	NM_010860	Myl6	17904	0,509002845	14,95011828	0,000464772
A_51_P217047	NM_025277	Gng10	14700	0,508911095	7,728150538	5,94E-05
A_55_P2069850	NM_011123	Plp1	18823	0,508352063	8,829021505	0,060957391
A_55_P1960936	XM_001473982	Gm2562	100040033	0,508349929	12,40744086	0,024338
A_55_P2158990	NM_010591	Jun	16476	0,508275249	7,41711828	0,008452902
A_52_P222725	NM_026148	Lims1	110829	0,508237553	9,409193548	0,024223257
A_55_P1999648	NM_007408	Plin2	11520	0,507820057	8,098913978	0,008470269
A_51_P144349	NM_172442	Dtx4	207521	0,50748293	6,627172043	4,19E-08
A_52_P748958	NM_007687	Cfl1	12631	0,50733926	12,21974194	3,70E-06
A_55_P2093665	XM_001478701	Gm3950	100042656	0,506302276	8,028548387	0,009810599
A_52_P179068	NM_017469	Gucy1b3	54195	0,506275249	7,742526882	0,021282591
A_52_P1197913	NM_008655	Gadd45b	17873	0,506225462	7,712645161	0,000308519
A_51_P475995	NM_178060	Thra	21833	0,506172119	7,220129032	0,000211508
A_51_P327121	NM_009515	Was	22376	0,505534851	9,354043011	0,000101299
A_51_P378298	NM_026976	Faim3	69169	0,505219772	9,612634409	0,058328868
A_51_P278550	NM_134118	Tecr	106529	0,50466074	7,981505376	6,07E-06
A_55_P2137203	NM_020579	B4galt3	57370	0,503834993	8,661795699	0,006480235
A_55_P1963491	NM_008972	Ptma	19231	0,503763158	8,936580645	0,003901726
A_52_P78023	NM_172397	Limd2	67803	0,503620199	12,58967742	0,010347513
A_55_P2007510	NM_026441	Pef1	67898	0,503529872	9,092763441	0,000393064
A_51_P486001	NM_172457	Mob3a	208228	0,503418208	8,062043011	6,34E-06
A_51_P501453	NM_022430	Ms4a8a	64381	0,503273115	6,68655914	0,001515818
A_55_P1992257	NM_007457	Ap1s1	11769	0,503266714	9,076817204	3,05E-06
A_52_P70796	NM_007551	Cxcr5	12145	0,503146515	7,935774194	0,00852497
A_66_P124551	NM_029332	Akap13	75547	0,502800142	9,141182796	0,005175143
A_52_P113700	NM_027324	Sfxn1	14057	0,502614509	8,088666667	0,00012002
A_55_P2033557	AB041803	Lincpint	232685	0,502368421	7,212365591	0,000678115
A_55_P1956063	NM_026701	Pbld1	68371	0,502292319	15,68898925	0,022909146
A_55_P2033530	NR_023357	Gm6981	629557	0,502256046	7,27211828	0,0001185
A_55_P2031668	NM_013541	Gstp1	14870	0,50222973	8,057623656	0,001222547
A_52_P479500	NM_008942	Npepps	19155	0,502024893	7,16188172	8,81E-06
A_55_P2049752	NM_173749	Pamr1	210622	0,501566145	6,665043011	0,032420423
A_66_P122086	NM_001039720	9030619P08Rik	105892	0,50155761	7,91272043	0,040590763
A_55_P2005555	NM_001110229	Celf2	14007	0,501554054	7,118086022	4,63E-05
A_52_P191975	NM_001003934	Rtn3	20168	0,501479374	7,781763441	0,004178977
A_55_P2033420	NM_023220	Sppl2a	66552	0,50133357	7,730752688	0,000299954
A_51_P401964	NM_146104	Aph1a	226548	0,501165007	7,44088172	5,05E-05
A_55_P2188464	NM_029166	Uhrf1bp1l	75089	0,501128734	7,565010753	0,000128856
A_55_P2174490	NM_007645	Cd37	12493	0,500890469	8,014505376	0,000392941

A_51_P385718	NM_026862	Cd177	68891	0,500721195	6,462107527	7,39E-06
A_52_P359965	NM_007754	Cpd	12874	0,500592461	6,581741935	0,00128643
A_55_P2062851	NM_020575	March7	57438	0,500179943	8,935150538	0,005404763
A_55_P2019699	NM_001139520	Samhd1	56045	0,500134424	10,79143011	0,015122902
A_55_P1954021	NM_016741	Scarb1	20778	0,500007112	7,22211828	0,001563481
A_55_P2166024	NM_030742	Vmn1r63	81017	-0,500125178	7,242419355	0,000621601
A_55_P1958200	NM_008771	P2rx1	18436	-0,500163585	12,00296774	0,075558805
A_55_P2149881	NM_010613	Khsrp	16549	-0,500284495	7,543397849	0,0030666
A_55_P2102621	NM_001113401	Eaf2	106389	-0,500357752	10,87712903	0,016615848
A_55_P2090441	AK138849	BC028471	414071	-0,500470839	8,408354839	0,006922502
A_51_P400040	NM_145441	Ubxn2a	217379	-0,500809388	6,68855914	1,76E-06
A_66_P137475	NM_001033155	Dnajb14	70604	-0,50105192	10,02789247	0,006670772
A_55_P1992299	AK031042	5830418K08Rik	319675	-0,501430299	8,291591398	0,028136674
A_55_P2014460	NM_007539	Bdkrb1	12061	-0,501463727	6,535827957	0,000823281
A_55_P2069969	NM_001122660	Gm10639	100042314	-0,501578236	6,214	0,004926751
A_55_P2117699	NM_172858	Pak7	241656	-0,501788051	8,888569892	0,060257515
A_52_P627269	NM_198171	Ces2b	234669	-0,501870555	6,587451613	0,000250551
A_55_P2156530	XM_001478556	Gm3941	100042638	-0,501921053	7,012043011	0,0008512
A_55_P2044710	NM_080444	Asb10	117590	-0,50208606	7,852806452	0,008847301
A_55_P2033312	NM_144556	Lgi4	243914	-0,502100996	7,048741935	3,03E-05
A_52_P670026	NM_021384	Rsad2	58185	-0,502124467	15,09535484	0,083661362
A_51_P483658	NM_018880	Trim3	55992	-0,502160028	7,700010753	0,002203285
A_55_P2007467	AB106871	Gm10244	621427	-0,50216074	7,822483871	0,018199745
A_52_P625171	NM_133723	Asph	65973	-0,502294452	6,351430108	2,25E-06
A_55_P2011151	NM_001163776	Tmprss3	140765	-0,502692034	7,840903226	0,000101107
A_55_P2017001	XM_979682	Gm8426	667024	-0,502819346	6,881591398	0,015268465
A_51_P219444	NM_013880	Plcl2	224860	-0,503104552	10,05131183	0,002225347
A_51_P397468	NM_016759	Rundc3a	51799	-0,503236842	8,624311828	0,011380551
A_55_P2353004	AK016253	Pitpnm2os1	75927	-0,503312945	8,926935484	0,016016225
A_55_P2398995	NM_027042	Lelp1	69332	-0,503535562	7,649021505	0,002373817
A_55_P2004821	NM_146936	Olfr1417	258938	-0,503761735	9,907473118	0,03952381
A_51_P360004	NM_029021	Ocstamp	74614	-0,503800853	13,14349462	0,089699665
A_55_P2037712	NM_133786	Smc4	70099	-0,503963016	8,390365591	0,000104815
A_51_P498882	NM_010001	Cyp2c37	13096	-0,504066856	7,48244086	0,021711131
A_55_P2115732	NM_153103	Kif1c	16562	-0,504697013	8,076623656	0,012639085
A_66_P121059	NR_028265	Mirg	100040724	-0,505613087	7,302	0,01746141
A_55_P2007656	NM_009964	Cryab	12955	-0,506096017	7,579247312	0,006618068
A_55_P1998892	NM_001177833	Smox	228608	-0,506564011	13,43855914	0,014461132
A_51_P457664	NM_010010	Cyp46a1	13116	-0,506985775	9,21611828	0,075057974
A_51_P143023	NM_001033172	Rab11fip2	74998	-0,507133713	6,992526882	0,000368444
A_55_P2093949	NM_146183	Zfp428	232969	-0,507610953	6,734989247	0,00716921
A_51_P160344	NM_028448	Cenpv	73139	-0,507696302	8,826763441	0,021763493
A_55_P2166543	NM_025571	Pam16	66449	-0,508401849	9,558096774	0,004344324
A_55_P2057132	NM_001077499	Scn8a	20273	-0,508470839	7,926989247	0,000260779
A_55_P2113774	NM_029887	Yif1b	77254	-0,508714794	8,457268817	0,003871326
A_55_P1965154	NM_025565	Spc25	66442	-0,508821479	7,009236559	7,06E-08
A_55_P2071716	NM_153590	Klre1	243655	-0,508951636	6,182817204	4,57E-07
A_55_P2038484	NM_175017	4933427D06Rik	232217	-0,509169275	9,737591398	0,000482465
A_55_P2035087	NM_177566	Arhgef15	442801	-0,509297297	8,111752688	5,99E-05
A_55_P2126950	NM_001085417	Zfp467	68910	-0,509381223	9,136580645	0,09245295

A_55_P2169804	NM_194059	Nanos3	244551	-0,509721195	10,71673118	0,010296778
A_51_P290904	AK040404	Raver2	242570	-0,50978734	8,858731183	0,012223592
A_55_P2002773	AK019036	1810053B23Rik	69857	-0,509820057	8,139494624	0,059431217
A_55_P2012389	NM_053197	Sfxn3	94280	-0,509825036	8,125806452	0,015299745
A_55_P2134387	NM_020289	Olfr544	257926	-0,510147226	12,34212903	0,03504785
A_55_P2102065	NM_001122660	Gm10639	100042314	-0,510445235	6,446365591	0,040738582
A_55_P2117400	AK032173	Cacng7	81904	-0,510513514	6,830784946	0,000538304
A_51_P361201	NM_013477	Atp6v0d1	11972	-0,510714794	9,221677419	0,000517937
A_55_P2048800	NM_001136055	Cd82	12521	-0,510839972	8,336946237	0,015224308
A_51_P506111	NM_019544	Msgn1	56184	-0,511152205	8,068645161	0,0375432
A_65_P07361	AK172895	Trim14	74735	-0,51129303	7,780322581	1,64E-07
A_55_P1990653	NM_001111062	Comt	12846	-0,511337838	8,262129032	9,97E-05
A_55_P2055920	AK018929	Kcnd3os	78635	-0,511372688	7,045311828	0,002572375
A_51_P131561	NM_029639	Plet1	76509	-0,51144239	7,074677419	0,007216016
A_51_P516728	NM_010404	Hap1	15114	-0,511455903	10,22266667	0,058000361
A_55_P1964363	NM_001012434	Kctd14	233529	-0,511479374	7,28127957	0,027595376
A_66_P126662	AK076313	4732414G09Rik	100043309	-0,511654339	7,562666667	0,017940012
A_55_P2020453	XM_001004753	Gm9304	668693	-0,511776671	8,830043011	0,013994458
A_55_P1960506	NM_001001493	Wdr83os	414077	-0,511954481	8,051322581	0,014782743
A_55_P1985591	NM_024170	Cxx1a	66158	-0,512219772	8,009795699	0,00343594
A_55_P2120682	NM_020585	Golga7	57437	-0,51236202	7,251419355	0,00068834
A_55_P2045765	NM_001033410	Rnf224	329360	-0,512388336	8,217924731	0,008662237
A_55_P2419711	AK016521	4932412D23Rik	75722	-0,512733997	9,434860215	0,031703233
A_55_P2004375	NM_053111	Ear6	93719	-0,512950925	7,46216129	0,002203285
A_51_P328400	NM_009939	Cops2	12848	-0,513647937	7,543817204	0,000913793
A_51_P348804	NM_019764	Amotl2	56332	-0,5136899	8,382731183	0,011774915
A_51_P151020	NM_009407	Tnp1	21958	-0,51395377	8,656258065	0,065351236
A_55_P1978136	NM_139064	Tnip2	231130	-0,513973684	8,886505376	0,000707083
A_52_P559498	NM_007713	Clk3	102414	-0,514255334	7,352860215	1,26E-10
A_55_P1978481	NM_009473	Nr1h2	22260	-0,515172119	11,50170968	0,003208959
A_55_P2053943	NM_146534	Olfr1368	258527	-0,51529872	8,044924731	0,00128643
A_55_P2314229	AK083360	C920021A13	328066	-0,515459459	8,882849462	0,023102973
A_66_P111049	NM_001101647	Prlh	623503	-0,516837838	7,259752688	0,006788226
A_51_P286321	NM_026760	2310036O22Rik	68544	-0,516899716	11,73591398	0,001176233
A_52_P180408	NM_026602	Bcas2	68183	-0,51708606	8,385870968	0,002871843
A_51_P353392	NM_019936	Cript	56724	-0,517284495	9,333870968	0,08563857
A_55_P1974542	NM_198612	Gxylt2	232313	-0,517495733	8,209387097	0,000246307
A_51_P224983	NM_012000	Cln8	26889	-0,517540541	7,485193548	0,013177706
A_51_P408946	NM_007633	Ccne1	12447	-0,518866999	7,55155914	0,000178918
A_55_P2035972	NM_021516	Mark3	17169	-0,518975107	7,301473118	0,001798669
A_51_P403273	NM_025816	Tax1bp1	52440	-0,51944808	10,50409677	0,042023115
A_52_P665386	NM_145578	Ube2m	22192	-0,519786629	9,969301075	0,016565092
A_51_P176505	NM_175109	Rps19bp1	66538	-0,520500711	8,019311828	1,54E-06
A_52_P110052	NM_010045	Ackr1	13349	-0,520502845	8,910731183	0,01698465
A_55_P2035495	NM_019778	Zbtb20	56490	-0,520907539	9,086935484	0,005754943
A_55_P2029176	AK142244	Atr	245000	-0,520923186	7,871344086	0,006721112
A_66_P126984	AK135960	Gm10523	100038505	-0,522027738	8,354096774	0,006341714
A_55_P2067463	AK007159	1700110I01Rik	73549	-0,522036984	8,511247312	0,000312382
A_55_P2042086	NM_008738	Nrtn	18188	-0,522100284	11,04935484	0,035027382
A_55_P2042526	NM_026673	Apoo	68316	-0,522353485	7,784784946	0,007100387

A_51_P239166	NM_020330	Adam21	56622	-0,522374822	6,735978495	0,000318322
A_55_P2101074	XM_001472853	Gm3848	100042441	-0,522458037	8,476333333	0,039113707
A_55_P2050453	NM_010416	Hemt1	15202	-0,522492176	7,120043011	0,010886609
A_66_P112005	NM_207568	Olfr1252	404331	-0,522492888	7,600516129	0,000113026
A_51_P450527	NM_011526	Tagln	21345	-0,522662873	11,6685914	0,062425967
A_52_P511821	NM_027057	Wdfy1	69368	-0,522716216	6,909075269	0,009875137
A_55_P1965298	NM_001099302	Gm5640	434797	-0,523066145	6,938849462	0,000346682
A_55_P2175195	NM_011505	Stxbp4	20913	-0,523110242	8,185182796	0,004701387
A_55_P2059332	NM_001039509	Pnkd	56695	-0,523202703	7,270688172	0,012352806
A_55_P2013765	NM_023130	Raly	19383	-0,523216927	9,769150538	0,002026121
A_51_P154469	NM_010761	Ccndbp1	17151	-0,52335064	14,30509677	0,041974929
A_51_P261835	NM_025376	Tmem8c	66139	-0,52402845	8,639978495	0,043182451
A_55_P1981105	NM_007454	Ap1b1	11764	-0,524325036	9,652268817	0,000344283
A_55_P2050628	NM_201640	Cyp4a31	666168	-0,524378378	7,446752688	0,0006181
A_66_P136813	NR_033803	6030408B16Rik	77717	-0,5248734	7,744043011	0,009000081
A_66_P102719	NM_025287	Spop	20747	-0,525034851	10,48144086	0,004743237
A_51_P304859	NM_022985	Zfand6	65098	-0,525497866	8,21444086	0,032478632
A_55_P1972449	NM_009164	Sh3bp1	20401	-0,525783073	8,01716129	0,003067918
A_52_P162486	NM_007542	Bgn	12111	-0,525965861	8,189752688	0,014603997
A_55_P2017373	NM_146100	Ina	226180	-0,526018492	9,255763441	0,025702283
A_51_P373890	NM_146079	Guca1b	107477	-0,526302987	9,130010753	0,003141587
A_55_P2121275	NM_001034864	Gm4907	236749	-0,526418208	7,885655914	0,001805863
A_55_P2125491	NM_011659	Tnfrsf4	22163	-0,5264367	9,072956989	0,013649141
A_55_P2064486	NM_001039047	Trim58	216781	-0,526780939	6,650946237	5,12E-05
A_55_P2020054	AK164572	2610035D17Rik	72386	-0,52686202	7,32988172	0,000786153
A_52_P533304	NM_173363	Eif5	217869	-0,527049075	12,21731183	0,01462483
A_55_P2334177	AK016962	4933428C20Rik	71250	-0,527316501	8,236204301	0,020685264
A_55_P2019009	NM_172495	Ncoa7	211329	-0,527391181	7,19588172	0,002173091
A_55_P2275249	NM_009377	Th	21823	-0,52764936	11,78862366	0,073715746
A_52_P612137	NM_009822	Runx1t1	12395	-0,528401849	6,217182796	1,58E-10
A_55_P2075941	NM_010897	Nf1	18015	-0,528503556	9,33283871	0,007422797
A_66_P125079	NM_008811	Pdha2	18598	-0,529233997	7,222731183	0,001143811
A_55_P2065754	NM_010676	Krtap19-5	16704	-0,529432432	9,102731183	0,012630722
A_55_P2144248	NM_146185	Zfp790	233056	-0,529433855	8,291677419	0,045741433
A_55_P2197847	CA492922	Lhx1os	78365	-0,529482219	9,557849462	0,021077609
A_55_P2018205	XM_001478261	Gm3855	100042461	-0,52976458	6,532677419	0,000499349
A_51_P499551	NM_133983	Cd276	102657	-0,530332859	7,222698925	0,001956787
A_51_P129546	NM_009219	Sstr4	20608	-0,530518492	7,700182796	0,003289682
A_55_P1970642	XM_001479403	Gm4164	100043007	-0,531307255	8,975870968	0,005764055
A_52_P414368	NM_178715	Tmem30b	238257	-0,53149431	8,468301075	0,037447778
A_55_P2155002	NM_001038998	Ccdc23	69216	-0,532268137	8,866053763	0,004172997
A_55_P2185990	NM_001081049	Kmt2a	214162	-0,532499289	7,566817204	0,005940875
A_51_P511482	NM_028680	Ift57	73916	-0,532728307	7,45116129	0,012202384
A_55_P2018283	NM_010439	Hmgb1	15289	-0,532751778	8,167774194	0,003069063
A_55_P2042161	NM_027105	Krtap26-1	69533	-0,532866287	7,382946237	0,001748752
A_52_P275069	NM_001177416	Gm6792	627821	-0,532955903	13,51155914	0,021674655
A_55_P2428968	NM_028838	Lrrc2	74249	-0,533864154	8,240731183	0,012105708
A_55_P2410240	AK079813	Gm20556	328576	-0,534531294	8,319516129	0,058364398
A_55_P2081405	BC066226	BC052688	432812	-0,534559033	10,29896774	0,015836192
A_52_P599624	NM_001159519	Pfas	237823	-0,535180654	8,149473118	0,012568406

A_55_P2146535	NM_009924	Cnr2	12802	-0,535253912	9,633204301	0,000140083
A_55_P1970740	NM_183086	Mrps10	64657	-0,535566145	11,42790323	0,010813802
A_55_P2376423	BU523966	Igkv4-72	385109	-0,535756046	8,785225806	0,005667468
A_51_P484842	NM_007493	Asgr2	11890	-0,535852063	7,931096774	0,005226634
A_55_P2129786	NM_001164802	Slc38a10	72055	-0,536135846	8,936870968	0,014415057
A_52_P637730	NM_145405	Ubl4	27643	-0,536233286	9,164688172	0,030668705
A_52_P392509	AK018404	Gin1	252876	-0,53630441	7,059	0,000157239
A_55_P2031157	NM_010108	Efna3	13638	-0,536682077	8,428225806	0,01942209
A_55_P2102408	XM_898851	Gm6603	625586	-0,536766714	6,866526882	0,008641141
A_52_P352735	NM_001081369	Ccdc153	270150	-0,537006401	7,024967742	0,014309543
A_55_P2422318	AK014953	4921523P09Rik	70926	-0,537214794	7,029591398	0,001650758
A_55_P2129373	NM_001013767	Capn11	268958	-0,537285206	10,44943011	0,024958228
A_66_P118989	AK087483	Gm8350	666892	-0,537293741	8,119107527	0,014224099
A_51_P105263	NM_201355	Nat14	269854	-0,537364865	6,873419355	0,000407318
A_55_P2051039	NM_001099346	Gm11937	100041488	-0,537731863	11,03054839	0,039513865
A_55_P1978424	NM_007536	Bcl2a1d	12047	-0,537761024	8,172946237	0,011325979
A_51_P311540	NM_007748	Cox6a1	12861	-0,537797297	14,41307527	0,003045568
A_66_P101132	NM_178202	Hist1h2bp	319188	-0,537842817	7,661354839	0,055527935
A_55_P2259500	AK083809	D130012P04Rik	319377	-0,537899716	8,552204301	0,016766509
A_55_P2388197	AK003103	1010001B22Rik	75403	-0,53835633	9,939946237	0,007755011
A_66_P140658	NM_001011822	Olfr787	258069	-0,538427454	12,43825806	0,073091911
A_55_P2115297	NM_009681	Ap3s1	11777	-0,538498578	9,414569892	0,00195542
A_55_P2171912	NM_016975	Gja3	14611	-0,538950925	12,90847312	0,041471401
A_55_P2023012	NM_026136	4930449I24Rik	67410	-0,539078947	7,401634409	0,00210841
A_55_P2154235	NR_024331	1700008J07Rik	629159	-0,539346373	8,510526882	0,017645076
A_55_P2010672	NM_010623	Kif17	16559	-0,539370555	9,964612903	0,025995122
A_55_P2117574	NM_001110130	Ppih	66101	-0,539889758	7,445989247	0,004172018
A_55_P1993640	NR_033141	Gm6994	629678	-0,53994808	9,595784946	0,000218426
A_55_P2041521	NM_001029987	Doxl2	243376	-0,540309388	7,502129032	0,002081944
A_55_P2017764	NM_028120	Cep89	72140	-0,540926743	7,120795699	1,36E-05
A_55_P2002122	NM_173402	Rgs12	71729	-0,542069701	7,003096774	4,56E-06
A_55_P2024145	NM_030741	Vmn1r62	81016	-0,54244239	7,161010753	0,000457164
A_51_P254299	NM_207666	Dlk2	106565	-0,542837127	8,442591398	0,007216016
A_55_P2000613	NM_178703	Slc6a1	232333	-0,543177098	8,607741935	0,02739496
A_55_P2100715	AK003929	Auh	11992	-0,543709815	9,275107527	0,075547502
A_55_P1988498	NM_026425	Naa20	67877	-0,544182077	8,249258065	2,00E-06
A_55_P2017729	XM_001478531	Gm2138	100039286	-0,544209104	8,791236559	0,002155212
A_55_P2197998	C77672	D4Ertd58e	52054	-0,544236842	12,45768817	0,047223729
A_55_P2240523	AK086741	Zmiz1os1	414118	-0,545088905	7,352483871	0,00213055
A_51_P499195	NM_024253	Nkg7	72310	-0,545386913	9,26772043	0,023586231
A_55_P2162879	NM_146384	Olfr208	258382	-0,545405405	6,202021505	9,69E-12
A_55_P2405244	AV207074	1700061N14Rik	73432	-0,545667141	7,900129032	0,028427579
A_55_P2124831	NM_001079873	Brdt	114642	-0,54584495	12,0004086	0,081006184
A_51_P379976	NM_001039515	Arl4a	11861	-0,546066856	8,839021505	0,033906927
A_55_P2099373	AK135677	Gm12633	619842	-0,546379801	7,165193548	0,000895957
A_66_P118141	NM_021309	Sh2d2a	27371	-0,546536273	9,66327957	0,007323248
A_55_P2007699	NM_028016	Nanog	71950	-0,546567568	10,41409677	0,004833017
A_51_P494992	NM_019732	Runx3	12399	-0,54685633	8,248182796	0,006106922
A_55_P2067682	NM_013795	Atp5l	27425	-0,547106686	11,14472043	0,039734206
A_55_P2083184	NR_026831	Gm10272	16697	-0,547178521	8,832505376	0,017206899

A_55_P1986341	NR_002858	Gm4956	241041	-0,547251778	6,772236559	0,002340198
A_55_P2096967	NM_008124	Gjb1	14618	-0,547369844	8,461774194	0,001322534
A_55_P2157378	NM_001045539	Xlr5a	574438	-0,547460882	7,770333333	0,015867484
A_55_P1967672	NM_207017	Tas2r109	387343	-0,547743243	8,86916129	0,025854959
A_51_P453475	NM_009201	Slc1a5	20514	-0,547984353	7,790548387	3,14E-10
A_55_P2216996	NM_001110497	Tmem87a	211499	-0,548105974	10,68345161	0,076184692
A_55_P2068731	NM_001177580	Gm13308	621580	-0,548106686	8,817924731	0,008427117
A_55_P2136154	NM_001033432	Heca	380629	-0,548931721	8,103741935	1,10E-07
A_55_P2132681	NM_019757	Fzr1	56371	-0,549939545	8,463150538	0,008483499
A_55_P2006792	NM_001025074	Ntrk2	18212	-0,550120199	14,19732258	0,027472231
A_66_P125634	NM_172864	Wdr63	242253	-0,550133001	10,36183871	0,025701927
A_52_P370392	NM_021921	Mapk8ip2	60597	-0,550302276	6,59344086	7,39E-06
A_55_P1985825	XM_001475310	Gm2980	100040810	-0,550490754	13,0978172	0,040734317
A_55_P2146891	BC051971	Ggt5	23887	-0,551503556	9,121537634	0,018329695
A_51_P514029	NM_010780	Cma1	17228	-0,55154623	6,322924731	1,11E-05
A_55_P2013576	AK016502	Agbl4	78933	-0,551740398	7,331086022	0,003146279
A_66_P117294	XM_001477027	Gm3516	100041800	-0,552025605	7,412806452	0,003113654
A_55_P2063980	NM_181549	Clec18a	353287	-0,552298009	7,895010753	0,017644736
A_66_P134181	NM_001012726	Aym1	503692	-0,552562589	8,383010753	0,015607528
A_55_P2002988	NM_001001559	Usp17ld	384701	-0,55266643	7,650086022	0,00252535
A_55_P2184567	XM_989827	Gm9677	676402	-0,552983642	8,799096774	0,016553771
A_55_P2142590	AF240782	Mark3	17169	-0,55501138	7,381956989	0,001661447
A_55_P2042713	NM_011822	Pigq	14755	-0,55541394	10,39374194	0,000855724
A_55_P1976634	NM_146312	Olfr657	258309	-0,555470128	7,40211828	0,004218852
A_51_P251205	NM_028627	Psd	73728	-0,555539118	8,612010753	0,007061788
A_51_P357744	NM_010592	Jund	16478	-0,555645092	13,20850538	0,009829758
A_52_P359819	NM_198190	Ntf5	78405	-0,555663585	9,356806452	0,036448415
A_55_P1976430	NM_207667	Fgf14	14169	-0,555684922	7,383	0,002441079
A_52_P179729	NM_025299	Txnl4a	27366	-0,555842105	7,97055914	2,28E-05
A_55_P2048388	NR_033736	E330020D12Rik	626058	-0,555958037	8,709677419	0,01979475
A_55_P2087013	NM_010432	Hipk1	15257	-0,556038407	8,112139785	0,032478632
A_55_P1959555	XM_990090	Gm8591	667357	-0,556105263	7,236612903	0,001699829
A_52_P633752	NM_007462	Apc	11789	-0,556320057	7,513494624	0,001079333
A_51_P475076	NM_011692	Vbp1	22327	-0,55635064	7,94983871	0,016464874
A_55_P2050652	NM_001136067	Ikbkg	16151	-0,556534851	6,695376344	5,62E-07
A_55_P2039699	NM_009921	Camp	12796	-0,55658037	8,279655914	0,005003434
A_55_P2096772	NM_172462	Zfp11	22648	-0,557369844	7,263817204	0,001660862
A_55_P1967133	NM_177741	Ppp1r3b	244416	-0,557809388	9,059204301	0,000814346
A_55_P2046550	NM_013906	Adamts8	30806	-0,557821479	12,49724731	0,025494039
A_55_P2154536	NM_001159626	Hagh	14651	-0,558020626	12,79272043	0,091780861
A_52_P610987	NM_022317	Slc28a3	114304	-0,558073969	8,844204301	0,028326624
A_51_P211506	NM_146071	Muc20	224116	-0,558540541	8,689569892	0,010246812
A_51_P184385	NM_023530	Pla2g12b	69836	-0,559274538	7,195827957	0,001820239
A_55_P2040367	NM_175277	Bola3	78653	-0,559696302	12,9803871	0,043871817
A_55_P2119962	NM_028637	1110034G24Rik	73747	-0,559737553	9,601827957	0,024167813
A_55_P2353462	AK162496	9530036O11Rik	654796	-0,560100284	8,22411828	0,004372641
A_51_P447258	NM_009659	Alox12b	11686	-0,560103841	7,868483871	0,007102155
A_51_P378210	NM_008190	Guca2a	14915	-0,560395448	8,004462366	0,012199158
A_55_P1991214	NM_001113474	Lair1	52855	-0,560433855	9,671010753	0,026489424
A_55_P2097340	NM_145621	Camkv	235604	-0,560556899	7,058860215	0,000601466

A_51_P418116	NM_146162	Tmem119	231633	-0,560738265	7,653505376	0,001796847
A_55_P2171578	NM_018810	Mkrn1	54484	-0,561026316	16,69011828	0,006653067
A_55_P1964615	NM_133763	Dnttip1	76233	-0,561125178	7,20188172	8,36E-06
A_51_P367423	NM_028849	Cldnd2	74276	-0,561303698	7,840634409	0,001331077
A_55_P2015253	NM_001081033	Pde11a	241489	-0,561539118	7,128236559	0,001205917
A_55_P2171773	AK076976	LOC102636217	102636217	-0,56188478	8,370172043	0,031703233
A_55_P2088113	NR_033543	Gm16287	100038595	-0,562032717	7,036580645	0,000147039
A_55_P2011061	NM_173007	Tspan12	269831	-0,562225462	6,081795699	1,83E-07
A_55_P2161278	AK220503	Spata2l	78779	-0,562295164	7,11116129	0,00024852
A_55_P2015405	NM_001114679	9930111J21Rik1	667214	-0,562332148	9,829763441	0,03476372
A_51_P156263	NM_027201	Zfp511	69752	-0,562446657	8,992935484	0,000962983
A_51_P424854	NM_013465	Ahsg	11625	-0,562539118	8,35344086	0,057309385
A_55_P2067727	NM_026280	Mxra7	67622	-0,563386202	7,256924731	0,004447506
A_55_P2053309	XM_889451	Rgs21	624910	-0,56348293	7,863268817	0,035969811
A_55_P2026669	NM_013795	Atp5l	27425	-0,563598862	12,3773871	0,03201015
A_55_P2089304	NR_024329	2900097C17Rik	347740	-0,563654339	8,059290323	0,001450544
A_51_P316173	NM_145845	Vmn1r192	252907	-0,563821479	7,415473118	0,000839535
A_51_P297069	NM_021883	Tmod1	21916	-0,563863442	7,009387097	0,008301517
A_55_P2037454	NM_023794	Etv5	104156	-0,564007824	12,62743011	0,03580237
A_55_P2008407	NM_133485	Ppp1r14c	76142	-0,564082504	11,83210753	0,055987666
A_55_P2115119	NM_027187	Rnaseh2a	69724	-0,564257468	7,739494624	2,67E-12
A_55_P2146295	NM_010725	Lmx1b	16917	-0,564394737	8,586806452	0,006950365
A_55_P2168426	NM_172293	Pced1b	239647	-0,564426743	9,354096774	2,50E-06
A_55_P2168451	AK044505	A930017K11Rik	100034748	-0,564482219	7,539	0,001750869
A_55_P2019814	NM_001163263	Rnf20	109331	-0,56464936	10,3661828	0,016855688
A_51_P108629	NM_008865	Prl3b1	18776	-0,564669986	8,133903226	0,002346897
A_52_P638283	NM_207258	Fam71f1	330277	-0,564862731	7,11311828	0,002747921
A_55_P2047626	NM_030021	Cutal	77996	-0,564963727	9,646301075	0,013905388
A_51_P153224	NM_025428	Zdhhc12	66220	-0,564970128	8,53555914	0,015455593
A_55_P2013043	NM_011454	Serpinb6b	20708	-0,56498293	7,132075269	1,06E-05
A_55_P2131238	NM_153392	Ttc39a	230603	-0,565603129	7,779634409	0,04315478
A_55_P2374197	AK035904	Spag17os	320613	-0,567088193	8,418505376	0,085375925
A_55_P1970159	NM_013876	Rnf11	29864	-0,567492888	12,12670968	0,071784802
A_55_P2248330	AK012503	2700068H02Rik	72563	-0,567572546	7,630698925	0,004144236
A_55_P2070054	NM_030702	Senp3	80886	-0,56795377	11,37329032	0,016543933
A_55_P2121142	NM_198647	Tbc1d22b	381085	-0,568465861	9,904935484	0,000943641
A_55_P1980677	NM_027037	1700007K09Rik	69318	-0,568526316	12,68415054	0,056102747
A_52_P106789	NM_026434	Rbm18	67889	-0,569435989	7,806268817	3,62E-08
A_55_P2025008	NM_001042489	Hvcn1	74096	-0,570036984	12,23305376	0,013122518
A_55_P2179805	NM_021482	Syngr4	58867	-0,570319346	7,70388172	0,000565725
A_55_P2183854	NM_001012322	Sctr	319229	-0,570524182	12,86913978	0,054095653
A_55_P2076941	NM_001081406	Lrr1	69706	-0,570611664	7,104333333	0,000169674
A_52_P438919	NM_001011814	Olfr524	258055	-0,570702703	7,817892473	0,004637349
A_55_P2076998	NM_009973	Csn1s2b	12992	-0,570707681	6,905204301	8,79E-05
A_55_P2001871	NM_009479	Uros	22276	-0,570901849	7,652365591	0,001976405
A_55_P2155620	NM_025282	Mef2c	17260	-0,570914651	9,61488172	0,049858802
A_51_P121455	NM_026542	Slc25a39	68066	-0,570930299	12,50329032	0,032612268
A_51_P377526	NM_009293	Sts	20905	-0,571507112	9,027989247	0,007755011
A_51_P144648	NM_177151	Vps13b	666173	-0,571547653	6,225956989	4,45E-08
A_55_P1996683	NM_008406	Itih1	16424	-0,571722617	7,925344086	0,012826677

A_55_P2148478	NM_029652	Klhl25	207952	-0,571852063	9,107451613	0,009828156
A_55_P2125496	NM_001129886	Gm10731	100039043	-0,571950213	11,91474194	0,036179639
A_55_P2174203	NM_144862	Lims2	225341	-0,572157183	8,540419355	0,012331924
A_55_P2215880	NM_010142	Ephb2	13844	-0,572630156	8,356043011	0,001631223
A_66_P125962	NM_010432	Hipk1	15257	-0,573175676	8,882924731	0,044789962
A_55_P1953093	NM_148917	Pabpc4	230721	-0,573499289	9,640193548	0,020884963
A_55_P2186778	NM_146804	Olfr905	258800	-0,573567568	7,081612903	0,006653067
A_55_P2173629	NM_001115154	Samd3	268288	-0,573733997	6,680322581	9,13E-07
A_55_P2029630	NM_011900	Mpdu1	24070	-0,57490256	8,772655914	0,02725816
A_55_P1960664	XM_001475067	Gm3035	100040908	-0,574906828	10,87849462	0,030522775
A_55_P2040962	NM_001033442	Gm1604b	381059	-0,575334993	10,43531183	0,00666451
A_55_P2422243	AK013766	2900072G11Rik	73005	-0,575517781	10,76390323	0,039488888
A_55_P2001159	NM_025699	Oser1	66680	-0,575539118	11,20909677	0,015235991
A_55_P2070373	NM_201374	Ccdc155	384619	-0,575642248	7,267698925	0,003705434
A_51_P114693	NM_145562	Parm1	231440	-0,575980085	7,389430108	0,000348573
A_52_P157170	NM_027258	Rnf157	217340	-0,576188478	7,549053763	0,006818352
A_55_P2039896	NM_028873	Dnajc14	74330	-0,576586771	8,944	0,048222622
A_55_P1995104	NR_024078	Btbd19	78611	-0,576699147	7,352752688	0,009263159
A_51_P450278	NM_027237	2010003K11Rik	69861	-0,576836415	7,391064516	0,005651257
A_55_P2173199	NM_001104615	Vmn2r4	637053	-0,576912518	7,419688172	0,004819705
A_55_P2037787	XM_001478340	Gm3798	100042341	-0,577387624	6,392731183	9,96E-05
A_51_P487048	NM_008955	Rhox6	19202	-0,577463016	7,04288172	0,001232912
A_52_P147803	NM_153577	Syne4	233066	-0,577484353	7,905075269	0,002836333
A_51_P295034	NM_010915	Klk1b4	18048	-0,577798009	8,64072043	0,030668705
A_52_P549954	XM_001477150	Gm3559	100041875	-0,578064011	7,690666667	0,001307157
A_55_P2150555	NM_029508	Pcgf5	76073	-0,57833357	9,470978495	0,003351006
A_55_P1971734	NM_016678	Reck	53614	-0,578484353	7,61127957	0,025480942
A_51_P446469	NM_010071	Dok2	13449	-0,57862091	9,55411828	0,005154592
A_55_P2077958	NM_008463	Klra5	16636	-0,578842817	6,324731183	7,15E-06
A_55_P1970274	NM_001033425	Zscan10	332221	-0,579744666	8,494645161	0,018812632
A_52_P416327	NM_178687	Cd226	225825	-0,580013514	9,738483871	0,01859799
A_51_P156631	NM_028419	Glrx5	73046	-0,580691323	14,07936559	0,095911574
A_55_P2117033	NM_028860	Mtmr3	74302	-0,580780939	9,763978495	0,00522067
A_55_P1996365	NM_206975	Ifna14	404549	-0,581046942	7,789924731	0,011977295
A_55_P2134938	NM_001135991	Krtap10-4	100191037	-0,581459459	8,326333333	0,004764679
A_55_P2003178	NM_172414	Zc2hc1c	72350	-0,581538407	6,517849462	1,11E-08
A_55_P2131498	AK160774	Tamm41	68971	-0,582020626	7,350623656	0,001953358
A_55_P2000909	NM_013795	Atp5l	27425	-0,582736842	12,86405376	0,024951908
A_52_P141687	NM_134438	Gpr37l1	171469	-0,582861309	8,668849462	0,010499387
A_55_P2206605	AK030886	5830444B04Rik	641454	-0,58301138	6,174677419	0,002311307
A_51_P501364	NM_019507	Tbx21	57765	-0,583038407	7,504655914	0,000201889
A_51_P127297	NM_008288	Hsd11b1	15483	-0,583133001	8,580580645	1,22E-06
A_55_P2054310	NM_010749	M6pr	17113	-0,583415363	9,049408602	0,000121061
A_51_P267278	NM_021301	Slc15a2	57738	-0,583998578	6,177365591	0,003110732
A_52_P337259	NM_013905	Heyl	56198	-0,584209104	9,960408602	0,042227661
A_55_P2127425	NM_025575	Sys1	66460	-0,584914651	10,54774194	0,016553771
A_51_P263246	NM_008748	Dusp8	18218	-0,585165007	6,34627957	1,34E-06
A_55_P1999202	NM_134239	Vmn1r217	171273	-0,585214794	9,359397849	0,018974514
A_55_P2170514	NM_018754	Sfn	55948	-0,585650782	7,697419355	0,005804158
A_55_P2071656	NM_010610	Kcnma1	16531	-0,585815789	6,943182796	0,000842538

A_55_P2018377	XM_001477144	Gm3502	100041765	-0,586174253	7,775634409	0,004377682
A_55_P2225751	AK031835	6330415G19Rik	320020	-0,586397582	8,720193548	0,008664748
A_55_P2151209	NM_130873	Krtap19-4	170654	-0,586434566	12,8277957	0,016543377
A_51_P177691	NM_008075	Gabrr1	14408	-0,586628734	7,876956989	0,00128643
A_55_P1961152	NM_011141	Pou3f1	18991	-0,586713371	9,022258065	0,009249802
A_51_P501922	NM_008881	Plxna1	18844	-0,586733997	9,988978495	0,002592016
A_55_P2099952	NM_009799	Car1	12346	-0,586823613	6,478591398	0,000315958
A_52_P129697	NM_172656	Stradb	227154	-0,58708037	8,600387097	0,007082159
A_55_P2169356	AK156879	Gm1966	434223	-0,587198435	9,294924731	0,07374495
A_55_P1963549	NM_001166842	Vmn1r168	100043101	-0,587608819	11,60791398	0,058083109
A_55_P2274592	NM_001163634	Wnt7b	22422	-0,588034139	7,41083871	0,002173091
A_55_P1961320	NM_207176	Tes	21753	-0,588277383	7,684645161	2,94E-05
A_51_P132718	AK019082	2310010J17Rik	78329	-0,588495733	10,97389247	0,015502978
A_51_P289107	NM_133849	Hrh3	99296	-0,588509246	7,48788172	0,003554641
A_51_P145511	NM_019991	Prl2a1	56635	-0,589118777	11,65334409	0,050567043
A_55_P2034027	NM_008713	Nos3	18127	-0,590152916	7,522258065	0,007525424
A_55_P2107901	NM_207666	Dlk2	106565	-0,591007824	12,42194624	0,058274691
A_51_P330144	NM_134126	Ift140	106633	-0,59116074	7,374666667	0,003975528
A_55_P1968464	NM_001081149	Kat6a	244349	-0,591258179	8,585483871	8,24E-05
A_55_P2072906	NM_001177391	Gm6788	627788	-0,591533428	9,420580645	4,74E-06
A_55_P1953236	AK158370	Epb4.2	13828	-0,591913229	6,885225806	0,007038216
A_55_P2065834	NM_144831	Dhx8	217207	-0,592337127	10,9992043	0,013911465
A_55_P1973941	NM_011404	Slc7a5	20539	-0,592633001	8,303021505	0,022414732
A_55_P2084646	NM_153409	Csrnp3	77771	-0,593713371	9,251688172	0,010497403
A_55_P2105970	NM_001122668	E330014E10Rik	665943	-0,593937411	8,10872043	0,008613576
A_55_P2024021	NM_013795	Atp5l	27425	-0,594018492	12,40465591	0,023895595
A_52_P476560	NM_145853	Tpcn1	252972	-0,594093883	8,508806452	0,005162576
A_55_P1989312	NM_001171004	Prkd3	75292	-0,594248933	7,557946237	0,014128549
A_51_P139678	NM_009264	Sprr1a	20753	-0,594319346	11,17278495	0,027754728
A_66_P120612	NR_002873	Vax2os	574519	-0,595439545	8,659946237	0,025644999
A_55_P2033500	NM_175641	Ltbp4	108075	-0,595456615	7,722301075	0,007216016
A_55_P2108389	NM_177135	D830030K20Rik	320333	-0,59571266	9,186623656	0,043034317
A_51_P430423	NM_007398	Ada	11486	-0,595822191	6,769483871	1,27E-07
A_51_P419959	NM_028932	Eaf1	74427	-0,596099573	7,157	6,34E-05
A_55_P2136902	NM_001085500	Cisd3	217149	-0,596579659	10,96335484	0,00835165
A_55_P2028949	NR_002880	Gm21944	654455	-0,596748933	6,977956989	0,000336673
A_55_P2155421	NM_001109971	Sec61g	20335	-0,596800142	16,03702151	0,030054443
A_55_P2099571	NM_028727	Nol9	74035	-0,597061166	7,997655914	0,006796192
A_55_P2055809	NM_134050	Rab15	104886	-0,597241821	8,212827957	0,005214814
A_52_P343661	NM_178036	Lcn10	332578	-0,598534139	8,219483871	0,004527144
A_66_P135700	NM_001104531	Cyp2d11	545123	-0,598906117	11,10492473	0,05133776
A_55_P1953411	AK162207	Efcab3	70894	-0,599321479	7,208225806	0,000802941
A_52_P447196	NM_053185	Col4a6	94216	-0,599381223	8,715967742	0,003477948
A_55_P2122666	NM_153169	Pnma3	245468	-0,599622333	10,54935484	0,025471492
A_55_P2089955	NM_001025086	Rhox7	547168	-0,599773115	11,45765591	0,029557496
A_51_P447976	NM_001142952	Fam46c	74645	-0,601004979	16,33609677	0,009993707
A_55_P1992889	NM_009776	Serping1	12258	-0,601802276	11,58846237	0,069260666
A_55_P2071191	NM_133351	Prss8	76560	-0,602193457	7,312677419	0,001656124
A_55_P2056774	NM_025330	Hsd17b14	66065	-0,602282361	9,361817204	0,013177706
A_55_P2077263	NM_021790	Cenpk	60411	-0,602980797	8,101419355	0,002577696

A_51_P364560	NR_033224	D330050I16Rik	414115	-0,603401849	7,308505376	0,003787498
A_51_P501248	NM_025367	Sphk1	20698	-0,603847795	7,020150538	0,006336989
A_55_P1977802	NM_176807	Folr4	64931	-0,60409744	7,955215054	0,000200315
A_51_P334942	NM_013467	Aldh1a1	11668	-0,604192745	14,4026129	0,010603963
A_51_P101006	NM_001033874	Ak8	68870	-0,604193457	8,042086022	0,007207691
A_55_P1970062	NM_001033323	Igsf9b	235086	-0,604334993	9,868236559	0,079281938
A_55_P2152387	NM_019573	Wwox	80707	-0,604535562	7,959655914	0,001066413
A_55_P2001290	NM_009454	Ube2e3	22193	-0,604891181	10,31521505	0,021850335
A_51_P394474	NM_008806	Pde6b	18587	-0,605190612	8,180397849	0,006106922
A_55_P2173947	NM_001159693	Zar1l	545824	-0,605344239	6,583591398	0,000100963
A_55_P2062444	NM_133771	Memo1	76890	-0,605391181	11,1018172	0,044292761
A_55_P2121697	AK140370	E530001K10Rik	414123	-0,6059367	8,784698925	0,01154775
A_55_P2091928	NM_009017	Raet1b	19369	-0,606544097	8,266741935	0,00275157
A_51_P194004	NM_174846	Glyctk	235582	-0,606668563	7,329537634	0,001663437
A_55_P2097478	NM_010266	Gda	14544	-0,606785917	15,02060215	0,043828976
A_51_P245631	NM_028713	Rftn2	74013	-0,607182077	8,226129032	0,004176047
A_55_P1979699	NM_025287	Spop	20747	-0,60793101	8,615322581	0,000309671
A_55_P2048656	NM_013795	Atp5l	27425	-0,607990043	12,3685914	0,015539563
A_55_P2137984	XM_979177	Gm7767	665748	-0,608409673	10,89673118	0,004701387
A_55_P2155136	NM_001008230	Rtp2	224055	-0,608709104	10,11591398	0,02085014
A_55_P2051444	NM_031190	Pgk2	18663	-0,608766714	8,084236559	0,005850502
A_55_P2167840	NM_001097977	Gm14151	433486	-0,60894239	7,478096774	0,00227057
A_55_P2062911	NM_007937	Epha5	13839	-0,609010669	9,332516129	3,28E-05
A_55_P1962209	NM_030712	Cxcr6	80901	-0,609239687	6,763860215	2,15E-05
A_55_P2020549	AK157864	Iscu	66383	-0,609832148	8,550967742	0,005520471
A_55_P2038111	NM_001033393	Tmem104	320534	-0,6098734	9,136408602	0,009286546
A_55_P2178632	NR_024509	Tmem80	71448	-0,610049075	9,260268817	0,008010254
A_51_P324303	NM_153789	Mylip	218203	-0,6101266	9,72883871	0,001450274
A_55_P2100149	NM_001038609	Mapt	17762	-0,610316501	7,77716129	0,006020519
A_55_P2053888	NM_146964	Olfr61	18362	-0,610514225	7,580688172	0,005842162
A_51_P470851	NM_146223	Cplx3	235415	-0,610525605	12,48525806	0,045394727
A_52_P558087	NM_026173	Poc5	67463	-0,611018492	8,272129032	0,000210656
A_51_P140347	NM_172830	Slc4a9	240215	-0,612719772	8,265827957	0,00128669
A_51_P226417	NM_178376	Rraga	68441	-0,613120199	7,678193548	8,17E-05
A_55_P2089677	NM_001115009	Synrg	217030	-0,613968706	10,99562366	0,005646358
A_66_P106095	AK036720	Nctc1	330677	-0,613974395	8,759408602	0,002697874
A_55_P2029417	NM_183287	2610318N02Rik	70458	-0,613990043	7,434817204	0,000311497
A_55_P2440441	NM_172598	Wdhd1	218973	-0,614048364	7,566612903	0,001657819
A_55_P2362601	AK078562	D4Ertd681e	52421	-0,614464438	9,225387097	0,01078593
A_55_P2001903	NR_024069	Smim4	66487	-0,614995733	9,278806452	3,12E-09
A_52_P276525	NM_175207	Ankrd9	74251	-0,615894737	9,195827957	0,015716611
A_55_P2043317	NM_001171739	Bag1	12017	-0,616165718	13,84208602	0,004547054
A_55_P2104662	NM_026213	Ttc33	67515	-0,616186344	9,043806452	0,022083368
A_55_P2100973	NM_016851	Irf6	54139	-0,616246799	11,21302151	0,071386266
A_66_P107671	NM_001081289	Fam217b	71532	-0,61649431	8,115666667	0,002697874
A_55_P1988678	NM_001081094	Znhit6	229937	-0,616970839	7,436655914	0,004912978
A_51_P412914	NM_010112	Efs	13644	-0,617241821	7,379913978	0,000937265
A_51_P454993	NM_178874	Tmcc2	68875	-0,617445235	8,500225806	0,004703303
A_55_P2002351	NM_153382	Lats2	50523	-0,61766074	6,685580645	3,01E-10
A_55_P1991505	NM_010255	Gamt	14431	-0,617796586	7,637946237	8,85E-05

A_66_P107703	NM_145533	Smox	228608	-0,618178521	10,98301075	0,011354066
A_52_P382876	NM_177692	Bloc1s3	232946	-0,618294452	8,658129032	0,010347513
A_51_P343323	NM_053071	Cox6c	12864	-0,618509246	12,16201075	0,013178466
A_55_P2108850	NM_145486	March2	224703	-0,618585349	12,63263441	0,012655196
A_55_P1999294	NM_028487	Gpbp1	73274	-0,618591038	10,33741935	0,00443827
A_51_P113773	NM_029420	Slx1b	75764	-0,618842817	10,56590323	0,027219173
A_51_P160413	NM_178691	Yod1	226418	-0,618885491	7,490290323	0,007245692
A_55_P1975285	NM_001025067	Lrig2	269473	-0,618992176	8,956258065	0,001669843
A_55_P2167189	NM_027600	4921504E06Rik	70909	-0,619703414	12,67437634	0,037449722
A_55_P2079516	NM_008621	Mpp1	17524	-0,619780228	8,778473118	0,049427706
A_55_P2246700	AU023048	D7Ertd595e	52137	-0,619998578	8,26472043	0,001407732
A_55_P1966528	NM_025928	Pmf1	67037	-0,620811522	10,12249462	0,002775352
A_55_P1978661	NM_153423	Wasf2	242687	-0,621417496	8,061591398	0,005734005
A_55_P2025468	NM_009437	Tst	22117	-0,621694879	9,209107527	0,025732489
A_55_P2061492	AK139523	Pnldc1	240023	-0,621756757	9,764268817	0,008569704
A_55_P2008567	NM_009096	Rps6	20104	-0,621815789	7,374537634	4,65E-06
A_55_P2176802	NM_010432	Hipk1	15257	-0,622314367	9,193193548	0,034600077
A_55_P2114427	NM_009952	Creb1	12912	-0,622325747	7,586763441	0,002473562
A_55_P2065113	NM_001177484	Gm11559	100415785	-0,622431721	10,79125806	0,012658329
A_51_P261107	NM_139144	Ogt	108155	-0,622665718	7,673913978	0,000474691
A_55_P1970067	NM_001129787	Igsf9b	235086	-0,62294239	8,955956989	0,008045551
A_55_P2108983	NM_001033378	A430078G23Rik	319493	-0,623083215	15,46263441	0,025322082
A_55_P2028651	BC120533	E030019B06Rik	212124	-0,623761024	7,910516129	0,001272708
A_55_P1974877	NM_009743	Bcl2l1	12048	-0,623861309	15,33122581	0,013219165
A_51_P205740	NM_054093	Ube3b	117146	-0,624422475	8,844516129	4,81E-06
A_55_P2157448	NM_198018	Abr	109934	-0,625465149	7,589107527	0,000345679
A_51_P133422	AK014772	Nck2	17974	-0,626330014	10,36341935	0,01387747
A_55_P2006133	NM_181321	Tmc6	217353	-0,626757468	13,58876344	0,051749547
A_55_P2114776	NM_015764	Greb1	268527	-0,62721266	8,14155914	0,002332298
A_51_P276479	NM_178098	4930486L24Rik	214639	-0,627837127	8,176956989	0,027373413
A_55_P1986703	AK006803	1700055N04Rik	73458	-0,627879801	8,520344086	0,005997001
A_51_P298486	NM_026225	Cog6	67542	-0,628159317	8,03927957	0,005948539
A_55_P2135383	NM_001177798	Taf6l	225895	-0,628407539	6,770397849	1,79E-05
A_65_P12359	NM_001033768	Pin1rt1	241593	-0,628463016	10,22030108	0,01090736
A_51_P511511	NM_054103	Stk33	117229	-0,628553343	7,342387097	0,000256684
A_55_P2173506	AK142977	Nhsl2	100042480	-0,628591038	7,329462366	0,000105895
A_55_P2019784	NM_028880	Lrrtm1	74342	-0,628630156	8,370483871	0,007854051
A_55_P2056521	NM_019647	Rpl21	19933	-0,630006401	6,520967742	4,80E-09
A_55_P2279997	AK016075	4930549C15Rik	75243	-0,630027738	10,69016129	0,031034874
A_55_P2295146	AK019394	4930445N18Rik	73985	-0,630615932	7,464430108	9,65E-05
A_55_P1994688	NM_001037740	Strip2	320609	-0,630868421	7,710387097	0,001929991
A_55_P2071952	NM_178909	Wdr92	103784	-0,630912518	10,99172043	0,001367614
A_55_P2085616	NM_021552	Nsa2	59050	-0,631162162	7,898784946	0,0014009
A_55_P1966070	NM_199063	Ssx9	382206	-0,631621622	9,418419355	0,026639558
A_55_P2114437	NM_013645	Pvalb	19293	-0,631964438	7,600462366	0,00063764
A_52_P145349	NM_172656	Stradb	227154	-0,633071124	9,380634409	0,001384662
A_55_P2024061	NM_153116	Gtpbp10	207704	-0,633182788	9,110913978	0,002106317
A_55_P2052066	XM_001473267	Gm2082	100039173	-0,633220484	7,088989247	0,000599996
A_66_P122219	NM_001039347	Kcnd3	56543	-0,633578236	9,392967742	0,024777558
A_55_P2094966	NM_178216	Hist2h3c1	15077	-0,633874822	7,291258065	2,42E-07

A_51_P489337	NM_019584	Becn1	56208	-0,634112376	9,89627957	0,001030981
A_55_P2074435	XM_001472207	Gm2046	100039100	-0,634172831	7,290494624	0,00128643
A_55_P2160726	AK147898	Tfcp2	21422	-0,634260313	7,913688172	0,003322707
A_55_P2004526	NM_010650	Klra8	16639	-0,634955903	7,106397849	0,000106777
A_55_P2135351	AF212920	Fam89b	17826	-0,635174964	10,53111828	0,002821996
A_55_P2077956	NM_001099918	Klrb1	100043861	-0,635349929	6,588505376	2,74E-09
A_52_P131062	NM_010675	Krtap8-1	16703	-0,635502134	9,087752688	0,008691577
A_55_P2184385	XM_001475079	Gm2908	100040689	-0,635576102	9,697956989	0,052600555
A_55_P2004741	NM_175471	Cyb5rl	230582	-0,635578236	7,104376344	0,000247234
A_66_P132496	AY261387	Asic3	171209	-0,635593883	8,281784946	0,018199745
A_52_P652316	NM_153058	Mapre2	212307	-0,635694879	8,451967742	0,000279282
A_55_P1973838	NM_008135	Slc6a9	14664	-0,635768137	8,555698925	0,007760174
A_51_P346884	NM_025665	Snrnp27	66618	-0,63593101	8,430043011	0,000125693
A_55_P2316760	AK020472	9430063H18Rik	77359	-0,636184922	8,882946237	0,009427878
A_66_P120558	NM_025571	Pam16	66449	-0,636497155	8,654645161	0,001331077
A_55_P1996171	NM_029508	Pcgf5	76073	-0,637462304	10,43466667	0,005395926
A_51_P428483	NM_181849	Fgb	110135	-0,637487198	7,693225806	0,003055208
A_55_P2161347	NM_001033041	Acmsd	266645	-0,63871835	7,649193548	0,035817643
A_51_P340829	NM_177604	AA986860	212439	-0,638778094	8,001935484	0,002173091
A_51_P426347	NM_009435	Tssk1	22114	-0,638959459	6,781580645	3,94E-05
A_55_P2157388	NM_177864	Skint9	329918	-0,639105974	8,665043011	0,003001033
A_52_P87793	NM_147118	Olfr635	259122	-0,639342105	8,66311828	0,015282412
A_55_P2070239	NM_016703	Preb	50907	-0,639462304	10,02507527	0,008662237
A_55_P2010788	NM_008135	Slc6a9	14664	-0,639552632	8,33916129	0,006964415
A_55_P2118441	NM_010846	Mx1	17857	-0,639822191	7,680473118	0,062043004
A_55_P2089198	NM_011880	Rgs7	24012	-0,640731152	10,57711828	0,028132189
A_52_P365768	NM_145354	Nsun2	28114	-0,641974395	7,910129032	2,20E-07
A_55_P2152547	NM_010859	Myl3	17897	-0,642260313	9,213322581	0,011319934
A_55_P2244355	AK010586	2410024N13Rik	70000	-0,642521337	8,174956989	0,019985949
A_55_P2081323	NM_019690	Gnas	14683	-0,643267425	6,890258065	9,82E-10
A_55_P1964348	NM_023608	Gdpd2	71584	-0,644225462	12,92654839	0,097903286
A_51_P401263	NM_177752	Eme1	268465	-0,644381935	8,615634409	0,017560336
A_55_P2034230	NM_009090	Polr2c	20021	-0,645010669	7,828870968	0,000703626
A_55_P2020572	NM_011192	Psme3	19192	-0,645269559	8,490086022	0,000482465
A_51_P337856	NM_027086	Ubl7	69459	-0,646353485	8,37611828	0,000421692
A_55_P2121076	NM_029402	Cul2	71745	-0,64672973	9,070397849	0,007514904
A_55_P2037235	NM_175397	Sp110	109032	-0,646878378	9,07727957	0,002192376
A_55_P2045278	NR_033535	Gm10845	100038734	-0,647295875	13,65	0,024440669
A_55_P2015143	NM_144881	Hhat	226861	-0,647763158	9,87683871	0,060711194
A_55_P2106150	NM_021790	Cenpk	60411	-0,648283073	7,811688172	0,00074983
A_51_P338878	NM_027571	P2ry12	70839	-0,648359175	9,853311828	0,01691429
A_66_P114169	NR_003372	Gm7904	666043	-0,648685633	6,888473118	0,000237147
A_51_P359516	NM_199223	Rtn4rl2	269295	-0,64874111	7,398376344	0,000144097
A_55_P1968774	NM_001162365	Ptk2b	19229	-0,648783784	9,274763441	0,00331333
A_55_P1975266	NM_027010	Crygf	12969	-0,649575391	8,304344086	0,017644736
A_55_P2090612	AK041413	Pilra	231805	-0,649847084	7,883075269	0,006134348
A_55_P2001023	NM_175305	Lrrc19	100061	-0,650244666	12,42754839	0,004729631
A_55_P2003532	NM_206935	Arl9	384185	-0,650905405	7,945075269	0,002877646
A_51_P234140	NM_183294	Cdkl1	71091	-0,651502134	9,147021505	0,007385676
A_55_P2057851	NM_146567	Olfr843	258560	-0,651940256	8,172462366	0,038832608

A_51_P239924	NM_009090	Polr2c	20021	-0,651966572	9,431967742	0,001082191
A_55_P2023057	NM_178055	Dnajb2	56812	-0,65206899	7,438096774	0,004655667
A_55_P1983959	NM_008049	Ftl2	14337	-0,653176387	15,59074194	0,00207825
A_55_P2032192	NM_001164249	Tpm1	22003	-0,65319559	10,05051613	0,011841576
A_55_P2078680	NM_001164251	Tpm1	22003	-0,654145092	8,881129032	0,012573301
A_55_P2173298	NM_177568	Plcb2	18796	-0,654196302	8,440193548	0,004458763
A_52_P52303	NM_001131020	Gfap	14580	-0,654322191	6,185935484	3,79E-06
A_55_P2203141	AK013525	2900011F02Rik	72901	-0,654369132	9,28016129	0,002469656
A_55_P1985911	NM_010919	Nkx2-2	18088	-0,654841394	8,307311828	0,003993383
A_55_P2035286	NM_010931	Uhrf1	18140	-0,655179232	7,277043011	2,23E-06
A_55_P2316783	NM_001081414	Grm5	108071	-0,655290185	8,678849462	0,004262992
A_55_P2157260	NR_033225	Gm13375	433408	-0,655588193	9,262086022	0,014478566
A_55_P2149873	NM_178684	Mapk1ip1l	218975	-0,656021337	8,390215054	0,004832101
A_55_P2129776	NM_030890	Prrt1	260297	-0,656410384	8,486010753	0,003741368
A_55_P2060343	NM_153102	Zfp352	236537	-0,656679943	10,51332258	0,012797507
A_52_P230688	NM_025620	Rep15	66532	-0,656756757	7,154419355	0,00018589
A_51_P289588	NM_009454	Ube2e3	22193	-0,656880512	10,72474194	0,012479017
A_52_P317653	NM_009799	Car1	12346	-0,656918919	7,353290323	0,000392941
A_55_P1983373	AK171276	Ampd3	11717	-0,657760313	8,325989247	0,002304948
A_51_P131653	NM_029801	Tsacc	76927	-0,657776671	10,23587097	0,01691264
A_52_P583155	NM_019661	Ykt6	56418	-0,658241821	8,176290323	0,000910043
A_55_P2149382	NM_001123367	Gm3448	100041639	-0,658314367	9,731548387	0,012486939
A_55_P2242089	AK041796	Cog5	238123	-0,65871835	8,83827957	0,007583022
A_55_P1996973	NM_029000	Gvin1	74558	-0,659443812	11,11986022	0,019585961
A_55_P2004541	NM_001110323	Klra7	16638	-0,659743243	7,247043011	0,001581864
A_55_P2051596	XM_001479558	Gm4499	100043524	-0,659974395	7,850806452	0,037298162
A_55_P2164428	NM_001033126	Cd27	21940	-0,660278805	9,164301075	0,005379642
A_55_P2152607	NM_172306	Cyp4a12b	13118	-0,660327881	7,970946237	0,001349006
A_55_P2009345	NM_172908	Ovch2	244199	-0,660997155	10,95615054	0,02173772
A_55_P2032024	NM_001081252	Uggt2	66435	-0,66120128	9,683935484	0,022904201
A_55_P1955437	NM_026066	Cmtm5	67272	-0,661569701	8,423010753	0,013188385
A_55_P2015375	NM_009446	Tuba3a	22144	-0,661703414	11,29995699	0,016024108
A_55_P2163143	NM_009870	Cdk4	12567	-0,662162873	11,3555914	0,00103393
A_55_P1989996	NM_013688	Tcte1	21645	-0,662421764	8,862806452	0,004903672
A_66_P132870	AK039020	Fcnaos	545410	-0,662540541	8,407817204	0,010583022
A_55_P2061432	NM_207262	Scgb2b2	381970	-0,662667852	8,318505376	0,004922792
A_55_P2051259	NM_001040026	Sco1	52892	-0,662875533	8,264655914	0,016645659
A_52_P481279	NM_001033460	Drc1	381738	-0,66355761	6,720376344	0,005980696
A_55_P2098428	NM_133724	Bloc1s4	117197	-0,663560455	7,292268817	9,01E-06
A_55_P2117764	NM_033509	Vangl2	93840	-0,663608108	8,394967742	0,00729665
A_55_P2152872	NM_054071	Fgfrl1	116701	-0,664036984	8,330258065	0,003359525
A_55_P1958867	NM_001112668	Gm9790	100042265	-0,664277383	8,819172043	0,000332988
A_51_P371876	NM_027230	Zmynd8	228880	-0,664536984	9,495860215	0,001224315
A_55_P2013011	XM_001477391	Gm9588	672987	-0,6645633	7,310365591	0,000474691
A_52_P663704	NM_001013384	Podnl1	244550	-0,665545519	10,65774194	0,024534413
A_55_P2083401	NM_001110239	Acp1	11431	-0,666068279	9,123483871	0,035295217
A_55_P2008599	NM_001162946	Pcx	18563	-0,667023471	7,29488172	0,001826381
A_51_P463452	NM_007981	Acsl1	14081	-0,667394026	7,823376344	0,005356493
A_66_P119735	XM_001477889	BC049265	414075	-0,667964438	8,487817204	0,006588129
A_51_P229163	NM_007762	Crhr1	12921	-0,668131579	8,904	0,008066182

A_55_P1983988	NM_145951	Enox2	209224	-0,668495021	7,487763441	0,00160626
A_55_P1960496	NM_025800	Ppp1r2	66849	-0,669897582	9,272752688	0,004176047
A_55_P1955542	NM_146952	Olfr522	258954	-0,669955192	8,995075269	0,006656195
A_55_P1955120	NM_173770	Fam69c	240479	-0,670302276	6,685956989	0,000100973
A_55_P2262593	AK142388	4930429F24Rik	74633	-0,670423898	7,447860215	0,00323989
A_55_P1982344	NM_001045544	Vmn1r204	632793	-0,671861309	7,211032258	1,01E-05
A_55_P2146297	NM_010725	Lmx1b	16917	-0,672160028	7,506741935	0,000386038
A_66_P106536	NM_027360	2010107E04Rik	70257	-0,672891892	11,83658065	0,000632177
A_51_P295315	NM_020033	Ankrd2	56642	-0,672901849	8,120204301	0,003720487
A_55_P1984118	NM_020014	Gfra4	14588	-0,674014936	13,76863441	0,019112373
A_55_P1985313	XM_001475212	Gm2703	100040307	-0,674109531	8,98955914	0,018928681
A_55_P1987231	NM_026928	Fuom	69064	-0,674236131	10,06477419	0,035561566
A_55_P2040200	NM_030695	Lrba	80877	-0,67487909	7,201473118	0,001657819
A_55_P2027323	NM_020585	Golga7	57437	-0,674937411	9,495268817	0,001527801
A_51_P346453	NM_153570	Noc4l	100608	-0,675315078	9,971494624	0,008256418
A_55_P2101508	NM_010205	Fgf8	14179	-0,675935989	9,398526882	0,012972073
A_55_P2251082	AK033279	Qk	19317	-0,676093883	8,92255914	0,008851613
A_55_P2113738	XM_001472127	Gm4102	100042906	-0,676441679	7,009913978	1,29E-05
A_55_P2105152	NM_025863	Trim59	66949	-0,676571835	7,963494624	0,02019634
A_52_P460929	NM_001001185	BC048507	408058	-0,677061166	8,785	1,36E-06
A_55_P1977330	NM_178744	Zbtb1	268564	-0,677347084	7,749193548	0,000603997
A_55_P1964613	AK168315	Dnttip1	76233	-0,677416785	9,69955914	0,000861161
A_52_P518808	BC027020	Mmd	67468	-0,677652205	8,70316129	0,032016351
A_55_P2358399	NR_033213	1700111N16Rik	74305	-0,677871977	8,910354839	0,007408691
A_52_P640194	NM_001033531	Klhl32	212390	-0,678551209	8,957182796	0,004841469
A_51_P141818	NM_029271	Mrpl32	75398	-0,678583926	8,449946237	0,002178796
A_51_P438083	NM_144512	Slc6a13	14412	-0,678993599	6,613462366	1,07E-08
A_66_P135872	AK019124	2410080I02Rik	68248	-0,679346373	8,282129032	0,001889265
A_51_P296866	NM_008629	Msi1	17690	-0,680785917	9,28372043	0,0018475
A_51_P131216	NM_030180	Usp54	78787	-0,680790185	10,84155914	0,020688042
A_55_P2002577	NM_010145	Ephx1	13849	-0,681405405	9,319806452	0,000260293
A_55_P2416097	AK038153	A130082J08Rik	320015	-0,682271693	8,729064516	0,034018143
A_51_P342716	NM_172707	Ppp1cb	19046	-0,682318634	13,95629032	0,014369866
A_55_P2032808	NM_053166	Trim7	94089	-0,682697724	7,673462366	0,000370671
A_55_P2002376	NM_009272	Srm	20810	-0,683008535	9,699215054	0,007709699
A_66_P136102	NM_177099	Lefty2	320202	-0,683646515	9,469602151	0,021854702
A_55_P2001403	NR_003960	Gm5478	432987	-0,684041252	8,436182796	0,000862455
A_55_P2225460	AK157022	AW555355	99413	-0,684083926	9,900569892	0,008140155
A_51_P164296	NM_021475	Adamdec1	58860	-0,684307255	6,965129032	0,050232535
A_55_P2159565	NM_207030	Tas2r131	387356	-0,684633713	7,854290323	0,004926751
A_51_P499061	NM_173755	Ube2o	217342	-0,685732575	14,92525806	0,004650095
A_55_P1989928	NM_173756	Lin52	217708	-0,686170697	8,894698925	0,017824915
A_55_P2186615	NM_001110239	Acp1	11431	-0,686172831	8,70011828	0,030893129
A_55_P1985623	NM_029600	Abcc3	76408	-0,687859175	6,72488172	1,92E-06
A_55_P2182700	NM_031869	Prkab1	19079	-0,687903983	9,414688172	0,002890726
A_55_P2096630	NM_199066	Ssxb9	387131	-0,689049075	11,3994086	0,027141769
A_55_P2089080	NM_024272	Ssbp2	66970	-0,68971266	8,849827957	1,72E-05
A_51_P133638	NR_030776	3110021A11Rik	67289	-0,68987909	7,904537634	0,001539647
A_55_P2006861	NM_001081302	Trio	223435	-0,690053343	9,903451613	0,011431875
A_55_P2096485	NM_025784	Bcs1l	66821	-0,691170697	9,72872043	0,000722034

A_55_P2158121	NM_025312	Sostdc1	66042	-0,691562589	8,670408602	0,033838464
A_66_P102919	XM_001474831	Gm3587	100041951	-0,691598862	8,421537634	0,001337124
A_55_P2097393	NM_026132	Txndc8	67402	-0,692243954	9,998655914	0,056457095
A_55_P1961908	NM_001099324	Gm15217	100041724	-0,693322191	9,610903226	0,01411245
A_55_P1994117	NM_177396	Ifnl3	338374	-0,693452347	10,47548387	0,01952458
A_55_P2076159	NM_207301	Wrb	71446	-0,693608108	8,444096774	0,007289379
A_55_P2083368	AK144366	Gm2044	100039095	-0,693707681	10,29233333	0,008812135
A_55_P2013559	NM_010075	Dpp6	13483	-0,694277383	8,599301075	0,003038539
A_55_P1953728	NM_016701	Nes	18008	-0,694285917	8,842978495	0,006146782
A_55_P2001334	AK036897	Gpr31b	436440	-0,69445377	10,24563441	0,024565436
A_55_P2169046	NM_146444	Olfr458	258436	-0,694654339	9,168053763	0,006653067
A_55_P2103626	NM_011989	Slc27a4	26569	-0,69605192	9,455731183	0,001249263
A_66_P118316	AK006709	1700047G07Rik	73323	-0,696065434	8,18772043	0,001244574
A_55_P2127991	NM_011888	Ccl19	24047	-0,696221906	7,103806452	7,39E-08
A_55_P2088325	NM_001113470	Ctdsp2	52468	-0,697103129	9,163	0,009260178
A_66_P133928	NM_026126	Fundc2	67391	-0,698865576	12,7813871	0,006107704
A_55_P1998811	XM_001476688	Gm3430	100041612	-0,699837838	8,528139785	0,033903907
A_55_P2396375	AK007978	1810073O08Rik	72285	-0,700183499	10,23607527	0,009365983
A_55_P2143070	NM_007494	Ass1	11898	-0,700487909	9,493569892	0,002606548
A_55_P2106844	XM_001476912	Gm7475	665070	-0,70055761	9,449935484	0,01847371
A_55_P2067707	NM_008585	Mep1a	17287	-0,700569701	7,049978495	0,000703425
A_51_P358872	NM_174853	Disc1	244667	-0,701189189	7,957064516	0,000724799
A_55_P1972663	NM_001039889	Smok3b	622474	-0,701282361	8,250043011	9,66E-05
A_55_P2046499	NM_134228	Vmn1r70	171262	-0,701490754	11,42866667	0,011479743
A_55_P2177112	NM_022999	Prrg2	65116	-0,701948791	8,008354839	8,45E-05
A_52_P42245	NM_010737	Klrb1a	17057	-0,702060455	6,887634409	0,000224084
A_55_P2044385	NM_028263	Fgfbp3	72514	-0,703157895	9,987655914	0,038521367
A_55_P2120596	NR_003364	BC018473	193217	-0,703586771	7,730946237	0,000102518
A_55_P2149615	NM_009750	Ngfrap1	12070	-0,703658606	11,53473118	0,007680669
A_55_P2312144	AK046489	B230398E01Rik	109334	-0,70479872	8,758139785	0,004236611
A_55_P1964842	NM_133752	Opa1	74143	-0,704874822	9,958763441	0,000861161
A_55_P2088690	XM_001478162	Gm3652	100042078	-0,705549787	7,918752688	0,003433785
A_55_P1991931	NM_025533	Nosip	66394	-0,70571266	7,823096774	2,16E-06
A_55_P1963031	NM_028779	Ampd2	109674	-0,706037696	9,312311828	0,002711386
A_55_P2357303	AK017255	5430401H09Rik	100504461	-0,706868421	7,698913978	0,008276428
A_51_P310949	NM_008911	Ppox	19044	-0,707985775	10,4351828	0,009701568
A_55_P2158384	NM_009591	Aanat	11298	-0,708193457	10,67233333	0,011207284
A_55_P1984770	NM_026050	Fam220a	67238	-0,708641536	15,43239785	0,014946212
A_55_P2083894	XM_001478221	Gm3697	100042151	-0,709045519	6,560129032	6,51E-05
A_51_P521052	NM_029627	Ly6k	76486	-0,70985064	8,870698925	0,002806708
A_55_P2312783	AK050884	D030029J20Rik	100502854	-0,710064011	10,02363441	0,011237726
A_51_P485240	NM_028320	Adipor1	72674	-0,710147937	13,25946237	0,002252588
A_51_P465148	NM_007798	Ctsb	13030	-0,71020128	12,38794624	0,000466113
A_52_P654752	NM_029839	Trub1	72133	-0,710311522	9,98227957	0,009816134
A_55_P1983095	NM_009616	Adam19	11492	-0,710876245	9,966935484	8,91E-06
A_55_P2024595	NM_001033436	Atxn7l1	380753	-0,710890469	9,607397849	0,000632087
A_55_P2047047	AK019009	Pla2g1b	18778	-0,711497155	9,937967742	0,008140155
A_55_P2301058	AK043315	C78653	97640	-0,711504979	8,460172043	0,002347187
A_51_P151835	NM_001110013	Tmtc3	237500	-0,712256046	8,330784946	0,009955774
A_52_P510877	NM_009743	Bcl2l1	12048	-0,713687767	9,279172043	0,009896288

A_55_P2139640	NM_015792	Fbxo18	50755	-0,71429303	7,327795699	8,65E-05
A_55_P2174977	BC045114	Cngb1	333329	-0,71494808	8,325537634	0,001331077
A_55_P2131153	NM_009211	Smarcc1	20588	-0,715553343	11,75616129	0,016148366
A_55_P1973006	NM_001171739	Bag1	12017	-0,715554765	12,48010753	0,001968394
A_55_P2048022	NR_003631	Gm6578	625347	-0,715625889	8,359419355	0,002133748
A_55_P1953402	NM_029993	Mlana	77836	-0,716253201	9,969709677	0,008140155
A_51_P414115	NM_172607	Naprt	223646	-0,716317923	7,220763441	1,80E-05
A_52_P116006	NM_010266	Gda	14544	-0,717470839	10,83668817	0,064741752
A_55_P2172822	AK008925	Mettl7a1	70152	-0,71779872	9,167795699	0,024940018
A_55_P2149511	NM_029409	Mff	75734	-0,717906117	11,23223656	0,001882927
A_55_P2166414	NM_197979	Uqcr10	66152	-0,718456615	10,89843011	0,000127247
A_55_P2115442	NM_053109	Clec2d	93694	-0,719067568	9,64783871	0,000461746
A_55_P2168014	NM_145404	Prmt7	214572	-0,719164296	7,747182796	0,002995231
A_55_P1978316	NM_001081127	Adamts14	237360	-0,719428876	7,704182796	0,00041788
A_55_P2024835	NR_033570	Gm7104	633093	-0,720415363	6,411397849	3,13E-10
A_55_P1992487	XM_888318	Gm6453	623818	-0,720475818	9,020612903	0,002155212
A_55_P1987953	NM_001085534	Gm5938	546335	-0,720532717	9,146462366	0,012028696
A_55_P2008258	NM_001040399	Larp1b	214048	-0,721177098	8,347107527	0,003974622
A_55_P2043833	NM_001081011	Srgap2	14270	-0,722137269	11,8798172	0,011342466
A_55_P2079967	NM_008580	Map3k5	26408	-0,72236202	9,604322581	0,005057535
A_66_P117204	AK041468	A630012P03Rik	100504594	-0,72297155	8,340784946	0,000565725
A_51_P501069	NM_011465	Spta1	20739	-0,723417496	9,453430108	0,053677034
A_51_P456721	NM_013478	Azgp1	12007	-0,72359175	6,923344086	2,42E-08
A_55_P1972322	NM_009770	Btg3	12228	-0,725849929	10,83849462	0,006679782
A_66_P111660	NM_013602	Mt1	17748	-0,726828592	9,280505376	0,014678369
A_55_P2131163	AK173092	Sv2c	75209	-0,727904694	7,358333333	0,000100973
A_55_P2045437	NM_177629	Fam216b	219170	-0,728103129	7,173333333	0,000814406
A_51_P150489	NM_031378	Gsdmc	83492	-0,72833926	9,086408602	0,010763783
A_52_P169901	NM_001009949	Slc25a51	230125	-0,72933357	10,99282796	0,062910245
A_55_P2030209	NM_025854	Cir1	66935	-0,729556899	8,905387097	0,008336111
A_55_P2048550	NM_001127686	Hbb-bh2	436003	-0,729790185	6,985569892	0,018199745
A_51_P291819	NM_024210	2310033P09Rik	67862	-0,730118065	10,78867742	0,017750077
A_55_P1959091	NM_031193	Ren2	19702	-0,730605263	10,26576344	0,010243162
A_52_P414420	NM_033623	Dcun1d1	114893	-0,731113798	10,02304301	0,033633045
A_55_P2063047	XM_001472600	Gm14206	100041796	-0,731394737	8,697924731	0,0015798
A_55_P1983368	NM_001162533	Sh3d21	66938	-0,731763158	11,031	0,006963432
A_55_P2005055	NM_008820	Pepd	18624	-0,732763869	8,956677419	0,000670193
A_55_P1972590	NM_173396	Tgif2	228839	-0,734145092	9,608473118	0,027012955
A_51_P517012	NM_027912	Tysnd1	71767	-0,734248222	10,23807527	0,002575432
A_55_P2114651	AK009098	Dynap	75577	-0,734352063	7,605150538	0,001597373
A_55_P2036605	NM_021318	Fhl5	57756	-0,736007112	9,131096774	0,007157459
A_51_P508770	NM_011846	Mmp17	23948	-0,73614936	8,77872043	0,002336427
A_55_P2036788	NR_004444	Zfhx2os	432855	-0,736348506	11,25419355	0,011319321
A_55_P1957729	NM_139219	Defb9	246079	-0,736555477	8,579870968	0,003513371
A_55_P2020326	NM_013591	Madcam1	17123	-0,736567568	11,00591398	0,034804423
A_52_P342159	NM_023699	Nfatc4	73181	-0,736664296	9,067258065	0,003794455
A_55_P2088846	NM_172799	Ttll6	237930	-0,737376245	8,109849462	0,00128643
A_55_P2153990	NM_001177439	Tldc2	383766	-0,737891892	8,814860215	0,004838228
A_55_P2183288	NM_133679	Cryzl1	66609	-0,738012091	9,635817204	0,016114899
A_51_P103541	NM_001081023	Cacna1s	12292	-0,738023471	8,553387097	0,002203285

A_55_P2136763	XM_886587	Gm6293	622178	-0,738497155	9,775483871	0,000305168
A_66_P121583	NM_001039244	Gm7120	633640	-0,73863798	9,10616129	0,002052628
A_55_P1997534	NM_001101463	Gm4871	231885	-0,739870555	8,531075269	0,003995783
A_51_P168613	NM_001004363	Nuak1	77976	-0,741056899	8,804236559	0,003551403
A_51_P358700	NM_146902	Olfr1221	258904	-0,741237553	6,734408602	0,000132722
A_51_P230507	NM_138953	Ell2	192657	-0,74172404	8,943021505	0,01993293
A_55_P1957850	NM_025664	Snx9	66616	-0,742056899	7,80944086	0,003743553
A_55_P2006634	NM_201519	Map4k5	399510	-0,742232575	8,368301075	0,003881926
A_55_P2079142	NM_173425	Fam124b	241128	-0,743364865	10,36650538	0,011553927
A_55_P2147736	NM_011993	Dpysl4	26757	-0,743871977	9,07983871	0,001597373
A_51_P148684	NM_175006	Pou6f2	218030	-0,744343528	9,012516129	0,008157025
A_55_P1973448	NM_016875	Ybx2	53422	-0,744359886	10,33539785	0,004726453
A_55_P2024909	NM_001167746	Dnah17	69926	-0,744700569	11,51402151	0,026411408
A_55_P2144210	NM_024225	Snx5	69178	-0,745193457	7,936892473	2,35E-07
A_55_P1986630	XM_001477989	Gm3627	100042018	-0,745681366	8,659451613	0,004151928
A_52_P450835	NM_197998	Cdpf1	72355	-0,746279516	7,399344086	1,04E-12
A_55_P2018949	NM_153195	Fbxo7	69754	-0,747296586	12,42090323	0,002886871
A_51_P124568	NM_008621	Mpp1	17524	-0,747620199	12,41017204	0,041204009
A_55_P1997554	NM_080466	Kcnn3	140493	-0,747687055	9,334645161	0,0113987
A_55_P1977071	NM_028666	Fam110a	73847	-0,748128023	11,05197849	0,006866357
A_52_P486322	NM_019757	Fzr1	56371	-0,748133001	9,729290323	0,002854328
A_52_P497424	AF004109	Aanat	11298	-0,750885491	10,41325806	0,027209136
A_55_P2177721	NM_007416	Adra1b	11548	-0,751504267	10,79187097	0,015637122
A_55_P2104071	NM_001012307	Defa23	497114	-0,751890469	11,18077419	0,018411985
A_55_P2122195	NM_001033876	Kcnk9	223604	-0,753979374	8,929322581	0,053852083
A_55_P2083180	NR_026831	Gm10272	16697	-0,754605974	8,225139785	0,001026878
A_55_P2050508	NM_011465	Spta1	20739	-0,755507112	9,639580645	0,023297068
A_55_P2001589	NM_178741	Klhl8	246293	-0,757636558	7,779096774	0,002713257
A_52_P183524	NM_023440	Tmem86b	68255	-0,75983926	6,499344086	1,56E-05
A_55_P2185526	XM_001002582	1700016P03Rik	668604	-0,760782361	12,04169892	0,022755577
A_66_P130634	NM_021288	Tyms	22171	-0,761109531	7,422333333	0,00018589
A_52_P457529	NM_177069	Fbxw21	320082	-0,761110953	11,39227957	0,007216016
A_51_P267700	NM_026860	Gkn3	68888	-0,761222617	9,17455914	0,001817248
A_55_P2061796	AK007238	1700122H20Rik	73617	-0,762344239	8,730666667	0,005997106
A_55_P2089840	NM_001136070	Eif2d	16865	-0,762965861	8,547172043	0,000680238
A_55_P2082688	NM_021515	Ak1	11636	-0,764307966	8,108946237	0,00042654
A_55_P2117681	NM_026406	Rnf115	67845	-0,764722617	8,811774194	0,000192287
A_51_P159612	NM_019487	Hebp2	56016	-0,765598862	8,499655914	0,00373827
A_51_P481768	NM_018756	Tcstv1	54382	-0,765623044	6,998741935	0,000114838
A_55_P2136765	NM_197979	Uqcr10	66152	-0,765800142	9,464548387	9,83E-05
A_55_P2199040	AK038608	LOC102635358	102635358	-0,766242532	8,288827957	0,001565396
A_55_P2025463	NM_001081315	Brpf3	268936	-0,766852063	8,463290323	0,003462536
A_55_P2096797	NM_001040696	Nlrp1b	637515	-0,768734708	8,200634409	0,00023357
A_51_P421303	NM_026769	Caly	68566	-0,768751067	9,174516129	0,001952413
A_55_P2105220	NM_153571	Hscb	100900	-0,768772404	12,28370968	0,022960184
A_55_P2096768	NM_009027	Rasgrf2	19418	-0,768837838	7,990236559	0,000293238
A_55_P2109003	AK040967	Gm4117	100042940	-0,769250356	8,102645161	0,00018098
A_55_P2170349	NM_053152	Klra22	93969	-0,7696899	8,518032258	0,008814532
A_51_P205968	NM_026912	Snx15	69024	-0,770252489	10,27226882	0,075962053
A_52_P136751	NM_007886	Dtnb	13528	-0,770612376	8,018666667	0,006342583

A_51_P263667	NM_007391	Acrv1	11451	-0,771121622	7,986419355	0,002181944
A_55_P2080756	NM_001011749	Olfr704	257902	-0,771345661	11,86545161	0,063663897
A_55_P2025746	NM_007401	Adam5	11499	-0,771687767	8,853021505	0,005935623
A_55_P2031636	NM_010512	Igf1	16000	-0,771981508	8,576419355	0,000663712
A_55_P2162432	NR_027847	D630029K05Rik	103175	-0,772093172	7,842698925	0,000637663
A_55_P1972381	NM_008127	Gjb4	14621	-0,772275249	6,571817204	0,031959653
A_55_P2129578	NM_001162924	Pkp3	56460	-0,773826458	11,60668817	0,002214985
A_55_P2097869	NM_172862	Frem2	242022	-0,774029161	9,562526882	0,004164362
A_51_P477419	NM_008688	Nfic	18029	-0,774428165	11,42894624	0,006426624
A_55_P2091323	NM_145514	Wdr26	226757	-0,7744367	8,150623656	0,004876468
A_52_P190405	NM_010509	Ifnar2	15976	-0,775912518	9,018344086	1,06E-07
A_55_P2258832	AK030345	5230400M03Rik	100502767	-0,776208393	9,09716129	0,007680669
A_51_P433584	NM_138681	Bcas3	192197	-0,777162162	9,364612903	0,002739454
A_52_P67212	NR_015605	2900052N01Rik	73040	-0,777788051	11,34595699	0,029305293
A_55_P1993728	NM_001161627	Tmem116	77462	-0,778760313	10,46970968	0,018765085
A_55_P2004208	NM_007847	Defa-rs2	13222	-0,778872688	8,594462366	0,050946174
A_55_P2014531	NM_033041	Hes7	84653	-0,778911807	8,892483871	0,004908543
A_52_P229972	NM_009202	Slc22a1	20517	-0,780174253	9,369268817	0,004472205
A_55_P2140107	NM_009995	Cyp21a1	13079	-0,780399716	8,887247312	0,00123819
A_51_P422124	NM_053090	Fam126a	84652	-0,781065434	8,311086022	2,26E-06
A_55_P2066017	XM_001478769	Gm4015	100042759	-0,782273115	6,39844086	2,64E-08
A_51_P235726	NM_023892	Icam4	78369	-0,783792319	6,668021505	5,49E-07
A_55_P2128324	NM_153489	Ubap2l	74383	-0,784403272	10,03343011	0,013911465
A_55_P2091985	NM_177366	Gpr157	269604	-0,785081081	12,64131183	0,076886704
A_51_P209319	NM_016752	Slc35b1	110172	-0,785291607	8,717354839	1,23E-06
A_51_P299062	NM_032540	Kel	23925	-0,786128023	9,784741935	0,008288309
A_51_P234544	NM_018745	Azin1	54375	-0,786842817	10,99622581	0,006850115
A_51_P177491	NM_023182	Ctrl	109660	-0,787213371	8,46872043	0,002713257
A_55_P2409306	AK035840	9030624J02Rik	71517	-0,787441679	8,738591398	0,001468406
A_55_P2032458	XM_893730	Gm6934	628919	-0,788710526	8,372634409	9,81E-05
A_55_P2145449	NM_172260	Cep68	216543	-0,788891181	7,661806452	0,000915211
A_55_P2117630	NM_030093	Snrnp25	78372	-0,789138691	9,336451613	0,004822978
A_55_P1976574	NM_016712	Tmod4	50874	-0,789458748	8,861354839	0,007183974
A_55_P1965902	NM_145501	Pi4k2a	84095	-0,789935277	9,244344086	0,002173091
A_55_P2366358	AK086457	4930444F02Rik	73968	-0,792669986	7,891483871	0,001768365
A_55_P2073915	BC083183	Nos1	18125	-0,793519915	10,05286022	0,006339946
A_51_P312846	NM_023605	Fbxo9	71538	-0,794044808	11,61702151	0,009542868
A_55_P2123331	NM_029746	Cog2	76332	-0,794376956	10,97396774	0,037569276
A_55_P2159885	NM_001166584	Tead1	21676	-0,794738265	10,55931183	0,018400553
A_52_P334796	NM_001008499	Taar4	209513	-0,795181366	12,08806452	0,029928889
A_55_P2157705	NM_197979	Uqcr10	66152	-0,795198435	10,01141935	2,54E-05
A_55_P2026340	NM_001161765	Fmo5	14263	-0,796136558	7,752462366	0,000576091
A_55_P2043862	NM_019641	Stmn1	16765	-0,796297297	10,0833871	0,000480072
A_55_P2064457	NM_001038655	Gng7	14708	-0,796784495	8,096473118	0,0016674
A_66_P110088	XM_001476538	Gm3265	100041308	-0,79780441	8,785924731	0,000655102
A_51_P122035	NM_153578	Nipa1	233280	-0,798310811	7,539784946	0,001139588
A_55_P2113439	NM_021371	Caln1	140904	-0,799041963	10,71215054	0,016865248
A_55_P2000148	NM_175138	Dnaic1	68922	-0,799884068	8,177322581	0,000442377
A_55_P2079520	NM_008621	Mpp1	17524	-0,80213229	11,70384946	0,034598586
A_55_P2066927	AK088379	Galt	14430	-0,804607397	9,141301075	0,00090985

A_55_P2132800	AK041361	Zfp367	238673	-0,805396871	9,913462366	0,002154518
A_52_P318673	NM_009117	Saa1	20208	-0,805516358	9,443946237	0,000780916
A_55_P2102155	NM_001177795	Rgs20	58175	-0,805604552	11,2796129	0,00676609
A_66_P124136	AK039088	Prkx	19108	-0,805763158	11,07011828	0,003920269
A_55_P2009066	NM_001166029	Cfap74	544678	-0,80705761	13,23919355	0,003901726
A_55_P1960208	NM_172955	Vcan	13003	-0,807293741	8,766268817	0,000621601
A_51_P368009	NM_177733	E2f2	242705	-0,808860597	13,87454839	0,001726586
A_55_P2154982	NM_009858	Cd8b1	12526	-0,8090633	11,1763871	0,002214985
A_55_P2210301	AK047689	Gm12992	545681	-0,810416074	9,284258065	0,000721792
A_55_P2094831	NM_021471	Slco1c1	58807	-0,810869132	7,249634409	0,000123773
A_55_P2004527	NM_010650	Klra8	16639	-0,811043385	7,233548387	2,21E-06
A_51_P117477	NM_011977	Slc27a1	26457	-0,812327881	10,305	0,019180584
A_55_P1960423	AK018877	Atp8b3	67331	-0,813276671	9,702139785	0,004671707
A_55_P2168185	XM_357571	Gm5299	384325	-0,81434495	15,09376344	5,49E-07
A_66_P130449	NM_207270	Ptprh	545902	-0,817214794	7,813795699	0,001331077
A_51_P348617	NM_001145552	2310045N01Rik	72368	-0,819171408	11,22434409	3,83E-05
A_52_P510202	NR_030696	9930014A18Rik	320469	-0,819857752	9,588903226	0,006155703
A_51_P273005	NM_009611	Actl7a	11470	-0,820296586	7,259817204	9,49E-05
A_55_P2049867	NM_017466	Ccrl2	54199	-0,821405405	8,438408602	0,000210563
A_55_P2094060	NM_010370	Gzma	14938	-0,823670697	11,87929032	0,005836692
A_55_P1953545	NM_010238	Brd2	14312	-0,825062589	7,847182796	0,000895957
A_66_P122155	NM_145514	Wdr26	226757	-0,82633357	10,44464516	0,004698586
A_55_P2009774	NM_009573	Zic1	22771	-0,826561878	8,307516129	5,41E-05
A_55_P1978681	NM_146010	Tspan8	216350	-0,826827881	6,743462366	1,33E-06
A_66_P132446	NM_027604	Usp15	14479	-0,828417496	7,13688172	0,000259143
A_51_P351860	NM_009777	C1qb	12260	-0,829073257	9,042096774	0,006129274
A_66_P103398	NM_001081079	Ogfrl1	70155	-0,829331437	8,16483871	0,001137428
A_55_P1955243	NM_011297	Rps24	20088	-0,830582504	8,708053763	3,54E-05
A_66_P133397	NM_145126	Chil4	104183	-0,831089616	9,483387097	0,001745185
A_55_P2023562	NM_199225	Cd300c	387565	-0,83197724	9,83683871	0,004994017
A_55_P2018111	NM_001101479	Pabpc4l	241989	-0,832118777	8,733989247	0,005713854
A_55_P2030938	NM_025863	Trim59	66949	-0,832266714	8,757397849	0,002443021
A_51_P439612	NM_020266	Dnajb2	56812	-0,834509957	10,0616129	0,005836919
A_51_P329370	NM_030093	Snrnp25	78372	-0,835009246	11,04274194	0,002762425
A_66_P111594	NM_053223	V1ra8	113850	-0,836596728	7,290741935	7,63E-05
A_55_P2022094	NM_001171053	Mta3	116871	-0,836708393	9,374021505	0,000565725
A_55_P1997997	NM_024470	Klra23	79410	-0,837096017	6,860870968	9,01E-07
A_55_P2011991	NM_022332	St7	64213	-0,837522048	9,251268817	0,002228672
A_55_P2041634	NM_001081041	Vps51	68505	-0,838029161	11,69860215	0,013945293
A_55_P2000813	NM_009939	Cops2	12848	-0,838215505	7,97355914	0,000295239
A_55_P2094626	NM_175211	Ralgps1	241308	-0,838349218	8,479505376	1,70E-06
A_55_P1956812	NM_178618	Fam83g	69640	-0,838594595	6,984731183	8,07E-06
A_55_P1963175	NM_145835	Lctl	235435	-0,839503556	7,725376344	0,000233182
A_55_P2228327	AK085171	4921529L05Rik	320178	-0,840860597	8,842086022	0,001554104
A_55_P2057031	NM_144867	Slmo1	225655	-0,842614509	9,227795699	0,000337806
A_55_P2152901	AK013627	2900040C04Rik	72893	-0,843079659	10,72821505	0,005157386
A_66_P126332	NM_001110508	Zfp703	353310	-0,843142248	12,04184946	0,0037936
A_55_P2067798	NM_207543	Vmn1r59	404284	-0,843502845	9,79872043	0,003697237
A_55_P2021953	NM_146600	Olfr700	258593	-0,843819346	11,64025806	0,018504096
A_51_P159711	NM_053070	Car7	12354	-0,844347084	11,43731183	0,001704777

A_55_P1958697	NM_028234	Rbm33	381626	-0,845562589	9,227870968	0,000637663
A_55_P2074045	XM_001478164	Gm3655	100042085	-0,84579872	9,703946237	0,001479762
A_55_P2080163	NM_025821	Carhsp1	52502	-0,846448791	15,02537634	0,000177416
A_55_P2050932	NM_133211	Tlr7	170743	-0,846449502	9,916849462	0,000405908
A_55_P2077009	NM_207223	Acap3	140500	-0,848414651	10,48107527	0,001691984
A_51_P466591	AK005436	1600012P17Rik	72025	-0,848808677	11,0261828	0,030283541
A_55_P1959748	NM_012055	Asns	27053	-0,848881223	7,98644086	0,000657
A_52_P608132	NM_001024560	Snx32	225861	-0,850509246	9,384408602	0,000937595
A_51_P134030	NM_145210	Oas1e	231699	-0,851192745	6,724075269	1,63E-08
A_51_P308549	NM_021422	Dnaja4	58233	-0,851731863	6,647698925	4,85E-10
A_55_P2063785	NM_001110783	Ank1	11733	-0,851797297	7,996225806	0,007554911
A_55_P2089362	NM_001012704	Wfdc13	408190	-0,851838549	10,21766667	0,003804355
A_55_P2136786	NM_133906	Zkscan1	74570	-0,851997155	14,44917204	0,00604429
A_55_P2070347	NM_008503	Rps2	16898	-0,852240398	11,90297849	0,000680238
A_55_P2166123	NM_015731	Atp9a	11981	-0,852662162	11,53253763	0,010129967
A_51_P480861	NM_021453	Pga5	58803	-0,853918919	8,210537634	0,00040615
A_55_P1993473	NM_001081657	Gm5935	546282	-0,855515647	11,72068817	0,012118422
A_55_P2208579	NM_194268	Onecut2	225631	-0,857473684	10,6531828	0,007734395
A_55_P1968643	NM_001166718	Vmn1r173	545934	-0,857780939	10,18056989	0,006703928
A_66_P136632	NM_019769	Chp1	56398	-0,859330725	7,636494624	0,000365569
A_55_P2063166	NM_177767	Ogfod1	270086	-0,861878378	10,8812043	1,69E-05
A_55_P2117425	NM_016908	Syt5	53420	-0,862268137	7,443473118	6,53E-06
A_52_P612382	NM_023117	Cdc25b	12531	-0,862694879	12,35034409	0,072583671
A_55_P1964245	NM_146959	Olfr631	258961	-0,86571266	9,539784946	0,004291858
A_55_P2058871	NM_138604	Otud5	54644	-0,866162162	11,9087957	0,00040845
A_55_P2073709	NM_178667	Tfdp2	211586	-0,86721266	12,3305914	0,020985785
A_55_P2014570	NM_001164581	Zfp961	234413	-0,871625178	10,7188172	0,006840656
A_51_P433388	NM_027070	1700019A02Rik	69397	-0,872396159	10,04567742	0,019936242
A_51_P418147	NM_181420	Fn3krp	238024	-0,87277027	6,529537634	0,001821257
A_51_P196972	NM_011403	Slc4a1	20533	-0,872960882	14,05133333	0,030054443
A_55_P2060330	NM_199145	3110062M04Rik	78412	-0,876526316	11,65407527	0,00547353
A_55_P1970154	NM_016698	Rnf10	50849	-0,877381935	13,65223656	0,001015209
A_55_P2076303	NM_027920	March8	71779	-0,87758037	10,22623656	0,003309797
A_55_P1987494	NM_008076	Gabrr2	14409	-0,880401849	10,59909677	0,006684705
A_55_P2023937	NM_022016	Impg1	63859	-0,880509957	10,29701075	0,003567776
A_55_P2081840	NM_001126338	Prnd	26434	-0,881460171	9,885096774	0,004148293
A_52_P638459	NM_013653	Ccl5	20304	-0,885175676	11,92266667	0,022779001
A_55_P1984640	NM_026454	Ube2f	67921	-0,887140114	9,806419355	0,003185551
A_55_P1963639	NM_026716	Sycn	68416	-0,887443101	9,241967742	0,003347882
A_51_P496432	NM_007981	Acsl1	14081	-0,887773826	10,96396774	0,015556211
A_55_P2117959	NM_007823	Cyp4b1	13120	-0,888794452	7,939892473	0,006789664
A_55_P2062538	BC100504	1700027A15Rik	69449	-0,888815078	9,154612903	0,000360089
A_52_P73126	NM_013513	Epb4.2	13828	-0,889358464	8,670075269	0,010870823
A_55_P1972192	NM_008832	Phka1	18679	-0,891426031	6,519430108	5,50E-22
A_51_P296608	NM_007836	Gadd45a	13197	-0,892889047	9,687634409	9,05E-05
A_52_P449417	NM_177545	Vangl1	229658	-0,893429587	8,324204301	0,005585275
A_55_P2077003	NM_009973	Csn1s2b	12992	-0,893485775	10,74858065	0,008154611
A_55_P1965448	XM_907154	Gm14989	632883	-0,893842817	12,27508602	0,007659679
A_55_P2022509	NM_172948	Mgat5b	268510	-0,894254623	9,747021505	0,003065423
A_52_P432685	NM_146012	Ctdsp2	52468	-0,897750356	8,225397849	0,000822334

A_55_P2185652	NM_001146311	Cln3	12752	-0,89970128	10,16716129	0,001524035
A_55_P1961127	NR_001592	H19	14955	-0,903167852	10,90813978	0,011905883
A_55_P2225470	AK039069	A230092J17Rik	320669	-0,903308677	8,275817204	0,003001033
A_52_P354373	AK012387	1190002F15Rik	381822	-0,90375889	9,012301075	0,03419085
A_51_P376347	NM_013546	Hebp1	15199	-0,907670697	10,08045161	0,029826732
A_55_P2165046	NM_145479	Klhl22	224023	-0,908757468	11,22037634	0,003292375
A_55_P1972792	NM_009185	Stil	20460	-0,908849218	9,022408602	0,00171529
A_51_P516148	NM_016984	Trpc4	22066	-0,909194879	8,548344086	0,003856848
A_55_P1969152	NM_030735	Vmn1r172	81010	-0,911330725	8,58311828	0,00099116
A_55_P2020080	XM_001479627	Gm4326	100043267	-0,91171266	9,479182796	0,000426549
A_55_P1974587	NM_177545	Vangl1	229658	-0,91285064	8,451172043	0,011266275
A_51_P241769	NM_011270	Rhd	19746	-0,913897582	9,136602151	0,019963689
A_55_P2152791	NM_001039093	Tom1l2	216810	-0,914029872	8,994602151	0,002886871
A_55_P2094686	AK078025	2700089E24Rik	381820	-0,915967283	7,842376344	3,56E-08
A_55_P2043466	NM_173363	Eif5	217869	-0,918407539	11,33764516	0,000451746
A_51_P142896	NM_007652	Cd59a	12509	-0,918963727	8,514623656	0,024356764
A_52_P467046	NM_026236	Wdr48	67561	-0,921094595	6,998086022	3,47E-08
A_55_P2144364	XM_001479624	Gm4320	100043258	-0,924311522	6,732	3,70E-07
A_51_P435251	NM_146207	Cul4a	99375	-0,925492888	9,651849462	0,007126697
A_52_P676198	NM_025912	Fam210b	67017	-0,925512802	9,370096774	0,014425248
A_52_P10732	NM_001048167	Map6	17760	-0,926603841	8,205860215	3,51E-05
A_51_P498772	NM_144953	1700019D03Rik	67080	-0,930965861	9,614494624	0,003472728
A_55_P2039429	NM_007860	Dio1	13370	-0,931139403	9,439935484	0,000975741
A_52_P658034	NM_009433	Tspyl1	22110	-0,931985775	10,61394624	4,13E-06
A_51_P345046	NM_019648	Metap2	56307	-0,933715505	9,867569892	0,002210132
A_55_P2057390	NM_028534	Smap1	98366	-0,934189189	11,27766667	0,000453042
A_55_P2138422	NM_027802	Obox1	71468	-0,934327881	11,83492473	0,020789047
A_55_P2060402	NM_153419	Grwd1	101612	-0,935600996	10,55280645	0,001965389
A_52_P483959	NM_207233	C1ql2	226359	-0,936653627	9,656021505	0,001515996
A_55_P1986213	NM_001004156	Plekhg5	269608	-0,942061878	9,500612903	0,002432284
A_55_P2069842	NM_001081232	D5Ertd579e	320661	-0,942326458	8,043612903	3,08E-09
A_55_P1975903	NM_001003961	Dnmt3b	13436	-0,942954481	9,615376344	0,000624268
A_66_P111089	NM_026454	Ube2f	67921	-0,943973684	8,496354839	0,001652103
A_55_P1971054	NM_198414	Paqr9	75552	-0,945387624	7,281913978	0,000232086
A_55_P2085716	NM_026217	Atg12	67526	-0,945679943	9,189365591	3,64E-07
A_55_P2117028	NM_028860	Mtmr3	74302	-0,946213371	10,4072043	0,00025578
A_55_P2140546	NM_001009949	Slc25a51	230125	-0,946801565	8,645473118	0,003477948
A_55_P1999108	NM_010031	Defa1	13216	-0,947170697	10,38502151	0,000744909
A_55_P2166069	NM_027173	2310079G19Rik	69699	-0,94736771	11,39507527	0,002869318
A_51_P455647	NM_009801	Car2	12349	-0,949194168	11,67104301	0,060907594
A_55_P2200628	AK014629	4733401D01Rik	70847	-0,950153627	10,61227957	0,000861161
A_55_P2042184	XM_001476516	Gm3181	100041175	-0,950299431	6,713268817	1,70E-06
A_51_P133097	NR_033452	Gm4013	100042757	-0,95127596	8,665860215	0,000172238
A_51_P418737	AK014289	Rbm33	381626	-0,952756757	7,693892473	8,71E-07
A_55_P2026014	NM_013596	Mc5r	17203	-0,954484353	8,954096774	0,003167206
A_55_P2045812	NM_011014	Sigmar1	18391	-0,956039829	9,744806452	0,024439976
A_51_P441745	AK021396	E130119H09Rik	78550	-0,959279516	9,768860215	0,009222927
A_55_P1991075	NM_029624	Lmf1	76483	-0,961992888	8,572806452	0,000200325
A_66_P111449	AK142058	1700018A04Rik	71307	-0,963871266	10,27831183	0,003247355
A_55_P2063376	NM_013458	Add2	11519	-0,964320768	8,201322581	0,000482465

A_55_P2168254	XM_001478363	Gm3880	100042516	-0,96572404	7,502784946	0,001581758
A_55_P2144781	NM_173421	Erich5	239368	-0,966686344	9,634913978	0,001110428
A_55_P2145029	NM_001033411	Gm826	329554	-0,967810811	10,20091398	0,006564942
A_55_P2109554	NM_012032	Serinc3	26943	-0,969067568	11,00091398	0,018641861
A_55_P2101910	NM_009438	Rpl13a	22121	-0,977743243	8,513010753	1,04E-09
A_52_P412452	NM_017383	Cntn6	53870	-0,982279516	11,10955914	0,002806506
A_55_P2105140	NM_001177731	Mrap2	244958	-0,984886202	8,738537634	0,000405238
A_55_P2063965	NM_008156	Gpld1	14756	-0,984999289	7,805763441	0,000369478
A_55_P2008021	NM_026838	Srpx2	68792	-0,986204125	9,982225806	0,000429691
A_55_P1980041	NM_008468	Kpna6	16650	-0,986989331	10,97854839	0,008005861
A_52_P87503	NM_001033981	Hbq1b	544763	-0,994050498	12,51366667	0,000556903
A_55_P2113498	NM_010654	Klrd1	16643	-0,995142959	7,545376344	2,70E-07
A_55_P2029121	AK007848	Pex11g	69129	-0,995825747	8,472569892	3,48E-06
A_55_P2179309	NM_001122596	A630033H20Rik	213438	-0,996474395	11,19305376	0,007993713
A_55_P1969511	NM_029430	Ttc23l	75777	-0,996955903	7,358354839	0,000253985
A_55_P2080598	NR_033355	5430416O09Rik	71406	-0,998312945	7,791064516	0,000835755
A_51_P136781	NM_018874	Pnliprp1	18946	-0,999578236	7,542741935	5,15E-07
A_51_P128575	NM_011681	Scgb1a1	22287	-0,99993101	8,229408602	0,000153225
A_51_P272817	NM_008928	Map2k3	26397	-1,005989331	9,436430108	7,52E-11
A_51_P172251	NM_025903	Ifrd2	15983	-1,007032006	9,205021505	0,00184801
A_51_P259879	NM_173430	Fkrp	243853	-1,007896871	9,191333333	0,001933868
A_55_P2083988	NM_008505	Lmo2	16909	-1,007972973	11,08995699	9,05E-05
A_52_P24631	NM_018745	Azin1	54375	-1,008487909	8,449494624	0,000385086
A_52_P77080	NM_010369	Gypa	14934	-1,0091266	9,304354839	0,008847301
A_51_P136888	NM_009029	Rb1	19645	-1,009908962	10,02278495	7,79E-05
A_55_P2052719	NM_001085410	Nadk2	68646	-1,011450213	9,836032258	0,001494135
A_51_P147034	NM_027407	Ica1l	70375	-1,011475818	9,800064516	0,095717078
A_52_P650855	NM_177390	Myo1d	338367	-1,017180654	8,752946237	0,001075404
A_51_P109050	NM_023824	Paqr4	76498	-1,017289474	6,675333333	1,41E-12
A_52_P522977	NM_024272	Ssbp2	66970	-1,019541963	10,57069892	0,002340862
A_51_P111544	NM_009273	Srp14	20813	-1,022789474	9,925956989	0,012356717
A_55_P2009146	NM_032544	Gtpbp3	70359	-1,022970839	10,67760215	0,009716064
A_51_P154585	NM_134223	Vmn1r195	171257	-1,024258179	12,11994624	0,002258409
A_52_P357133	NM_053267	Selm	114679	-1,034246088	9,281946237	0,000975503
A_51_P163444	NM_025821	Carhsp1	52502	-1,038146515	10,41952688	0,000305141
A_52_P482124	NM_026455	Fam32a	67922	-1,040001422	9,017419355	8,84E-11
A_51_P311362	AK015429	4930449E01Rik	74864	-1,040998578	8,125731183	1,27E-05
A_55_P1960566	NM_139117	Ybx3	56449	-1,042220484	14,42454839	0,001804775
A_55_P2026370	NM_027946	Dcaf7	71833	-1,046837838	9,181032258	0,000294456
A_55_P2066219	XM_001473544	Gm3455	100041653	-1,047093172	7,84188172	2,81E-06
A_55_P2167123	NR_033575	Cyp4b1-ps2	631037	-1,047844239	10,13049462	0,00835165
A_52_P237652	NR_015556	2610035D17Rik	72386	-1,051975818	7,53083871	0,000351538
A_55_P1976112	NM_178692	C130074G19Rik	226777	-1,055973684	11,09623656	5,22E-05
A_55_P2045886	NM_019635	Stk3	56274	-1,063676387	7,498107527	0,000118829
A_55_P1980883	NM_001146351	Ephb6	13848	-1,06669559	17,04249462	0,070100882
A_51_P513541	NM_019437	Rfk	54391	-1,071153627	10,349	0,00112624
A_55_P1988844	NM_016970	Klrg1	50928	-1,07470128	7,084913978	1,14E-10
A_52_P475870	NM_001164686	Tmem29	382245	-1,077901138	7,656473118	1,57E-07
A_51_P112627	NM_009180	St6galnac2	20446	-1,078645804	7,119827957	0,001799063
A_52_P91235	NM_017367	Ccni	12453	-1,082279516	11,27198925	1,17E-07

A_51_P417891	NM_011280	Trim10	19824	-1,083508535	10,27753763	0,014946212
A_55_P2087118	NM_020498	Ly6i	57248	-1,084010669	7,78955914	6,96E-06
A_55_P2005248	NM_008627	Meis3	17537	-1,08569559	11,21537634	0,005601956
A_55_P2076543	XM_001476757	Gm10144	100041121	-1,086798009	10,75070968	0,001498965
A_55_P2151855	NM_001110239	Acp1	11431	-1,088549075	10,11552688	0,004730752
A_51_P249118	NM_008830	Abcb4	18670	-1,090727596	8,231688172	1,48E-05
A_51_P342189	AK080303	A630091E08Rik	233544	-1,092405405	9,121774194	0,002026121
A_66_P106388	NM_029499	Ms4a4c	64380	-1,096945946	7,46816129	0,028250894
A_55_P2279140	AK089751	F830014O18Rik	403347	-1,098949502	10,59693548	0,004103354
A_65_P20167	NM_021395	Hyou1	12282	-1,099278805	7,504989247	4,15E-05
A_51_P192860	NM_008248	Hint1	15254	-1,104090327	11,40463441	2,72E-14
A_55_P2142668	XM_001480974	Gm4653	100043795	-1,106624467	10,74135484	0,046177871
A_55_P2397854	AK019540	4921518K17Rik	78758	-1,108252489	9,572322581	0,000170074
A_51_P237040	NM_008711	Nog	18121	-1,116370555	10,37649462	0,000121537
A_55_P2006698	NR_033241	Bmp1	12153	-1,117640114	9,493010753	0,000320106
A_51_P126198	NM_010019	Dapk2	13143	-1,118079659	8,440397849	1,03E-05
A_52_P313285	AF108397	Slc8a1	20541	-1,122278094	7,156215054	1,55E-10
A_55_P2078494	NM_001080812	Cib3	234421	-1,123605263	6,948053763	7,75E-05
A_55_P2167451	NM_033569	Cnnm2	94219	-1,126099573	7,94727957	1,38E-05
A_55_P2003592	BC059910	Unkl	74154	-1,126247511	8,496688172	7,63E-05
A_52_P309337	NM_019648	Metap2	56307	-1,128984353	7,452247312	2,38E-06
A_55_P2139814	AF199608	Fgf13	14168	-1,133674964	11,14146237	0,007680669
A_55_P1970120	NM_146168	Vopp1	232023	-1,14159744	12,89452688	0,000391711
A_51_P300506	NM_183405	Cox6b2	333182	-1,14236202	10,41512903	0,002394704
A_66_P108685	NM_009894	Cideb	12684	-1,153929587	9,268924731	0,001107091
A_55_P2090184	NM_026218	Fgfr1op2	67529	-1,154916785	10,2796129	3,03E-05
A_55_P2155876	NM_001113395	Gm16501	100042840	-1,159375533	7,603591398	1,65E-07
A_55_P2021413	NM_001146292	Celf4	108013	-1,161556188	12,32917204	0,004408491
A_51_P221337	NM_145824	Ranbp10	74334	-1,162499289	10,81552688	0,000401522
A_55_P2054913	NM_001011863	Olfr406	258181	-1,16820128	8,951516129	0,000934583
A_51_P197378	NM_026217	Atg12	67526	-1,171635846	10,8442043	4,19E-08
A_55_P2112085	NM_027906	Vwa8	219189	-1,187041963	9,987419355	0,000847259
A_55_P2168316	NM_001177438	Aldh3b2	621603	-1,191093883	6,663774194	0,000405908
A_55_P2334424	AK158188	D630024D03Rik	414116	-1,19227027	7,336311828	3,62E-05
A_52_P463346	NM_012032	Serinc3	26943	-1,195882646	9,65427957	0,002600557
A_55_P2099785	NM_027604	Usp15	14479	-1,19783357	10,87804301	0,000789401
A_52_P609200	NM_012032	Serinc3	26943	-1,218107397	12,70227957	0,012507271
A_55_P2100088	NM_001013784	E130309D14Rik	432582	-1,218246799	6,509010753	1,35E-11
A_55_P2150717	NM_010136	Eomes	13813	-1,223901849	8,240774194	2,90E-10
A_55_P2180096	NM_001178039	Gm10136	672214	-1,243444523	12,91064516	1,26E-10
A_55_P2038362	NM_145444	Acot5	217698	-1,248868421	7,876645161	0,00021884
A_55_P2329298	AK087850	Gm12758	100126229	-1,252424609	7,788236559	0,000280676
A_51_P485594	NM_025729	Tab3	66724	-1,25483926	8,151473118	1,43E-05
A_55_P2006148	NM_001177438	Aldh3b2	621603	-1,255595306	7,010397849	0,001782495
A_66_P104296	NM_028275	1700112E06Rik	76633	-1,25709744	7,173623656	9,25E-11
A_55_P2175579	NM_146694	Olfr1466	258689	-1,262164296	11,95411828	0,033276522
A_55_P2009116	NR_033558	F830002L21Rik	414125	-1,264285206	7,580956989	1,52E-06
A_55_P2043684	NM_133835	Ubac1	98766	-1,273254623	12,32345161	0,001191801
A_55_P2178510	NM_008561	Mc3r	17201	-1,292970128	9,870290323	0,028132189
A_51_P517695	NM_008530	Ly6f	17071	-1,309403272	7,727688172	6,95E-08

A_52_P493620	NM_026218	Fgfr1op2	67529	-1,321456615	9,218623656	3,29E-06
A_55_P2043682	BC021811	Ubac1	98766	-1,321596728	12,03082796	0,000871858
A_51_P260169	NM_010360	Gstm5	14866	-1,346461593	9,685043011	0,020471461
A_55_P2085546	NM_021422	Dnaja4	58233	-1,354754623	11,45760215	0,000150583
A_55_P2010292	NM_009247	Serpina1e	20704	-1,363968706	9,747849462	0,002537527
A_55_P2065567	NM_026527	Chac2	68044	-1,39454623	8,771150538	5,37E-05
A_55_P2094731	NM_011439	Sox13	20668	-1,414455903	14,94215054	0,043359544
A_55_P2036813	NM_030082	Hist3h2ba	78303	-1,434877667	8,452795699	3,90E-05
A_51_P120066	NR_033222	9330151L19Rik	414085	-1,435753912	7,497677419	1,04E-13
A_51_P501312	NM_025294	Natd1	24083	-1,440820057	7,768698925	5,07E-33
A_52_P484838	NM_011266	Rfxank	19727	-1,443197013	8,339860215	1,43E-17
A_55_P1979147	NM_001159904	Klrb1c	17059	-1,45173542	7,383645161	3,84E-08
A_55_P1959985	NM_020559	Alas1	11655	-1,457398293	10,5196129	5,59E-07
A_51_P198675	NM_138951	Ttc36	192653	-1,523484353	7,223344086	2,67E-14
A_52_P450934	NM_198414	Paqr9	75552	-1,555623755	10,5726129	0,001031129
A_55_P2156288	XM_001479076	Gm3932	100042616	-1,567237553	6,53316129	6,80E-07
A_55_P2096340	NM_029965	Rnf170	77733	-1,570621622	11,43025806	0,002029265
A_55_P2105858	NM_030693	Atf5	107503	-1,58713229	10,71622581	0,000171068
A_55_P2396446	AI593498	Dusp8	18218	-1,595353485	9,809	0,000841954
A_55_P2170509	NM_026417	Yipf4	67864	-1,671	10,1913871	0,000572445
A_52_P104824	NM_019670	Diap3	56419	-1,726324324	10,65836559	0,001125687
A_55_P1988975	BC028433	Ms4a4b	60361	-1,766899716	8,171290323	0,001112405
A_55_P1960231	NM_019913	Txn2	56551	-1,819573969	11,38143011	8,72E-07
A_55_P2026639	NM_001100614	Gm11564	670496	-1,952242532	7,642129032	2,90E-15
A_55_P2051455	NM_178882	D2hgdh	98314	-2,082539118	9,752978495	2,39E-08
A_55_P2331804	AK139027	AU015791	104932	-2,116881935	7,256967742	1,85E-16
A_55_P2410875	AK034163	C030037D09Rik	193280	-2,845318634	8,738193548	2,07E-12
