# Supplementary material for: Of mice and men: the host response to influenza virus infection
Source: Mamm Genome. 2018 Jun 15;29(7):446–70. doi: 10.1007/s00335-018-9750-y (PMC6132725; doi:10.1007/s00335-018-9750-y)
Supplement: Supplementary file 12 — Supplementary material 12 (PDF 22 KB) [file 335_2018_9750_MOESM12_ESM.pdf]

VENN gene lists resistant versus control for CC strains

```
$CC_resis_ctr
  [1] "1110038F14RIK" "1110046J04RIK" "1110059E24RIK" "1500011B03RIK" "1700037H04RIK" "1810058I24RIK" "2010002M12RIK"
  [8] "2610002J02RIK" "2700046G09RIK" "2700089E24RIK" "4833420G17RIK" "4930449E01RIK" "5033406O09RIK" "5330406M23RIK"
 [15] "6030458C11RIK" "6430548M08RIK" "9030619P08RIK" "9330151L19RIK" "A130040M12RIK" "A530040E14RIK" "A530064D06RIK"
 [22] "A930005H10RIK" "ABCB4"         "ABCC3"         "ACKR1"         "ACTB"          "ACTG1"         "ADA"
 [29] "ADAM8"         "ADAMTS14"      "ADD2"          "AGO2"          "AI449595"      "AI662270"      "AKAP7"
 [36] "ALAS1"         "ALDOA"         "ALKBH5"        "AMICA1"        "ANXA1"         "ANXA2"         "APLF"
 [43] "APOL7B"        "ARRDC3"        "ASNS"          "ASNSD1"        "ATF5"          "ATG12"         "ATXN7L1"
 [50] "AU015791"      "AW011738"      "AW112010"      "BB163080"      "BCL2A1D"       "BIRC2"         "BLNK"
 [57] "BRCC3"         "BST1"          "BTG3"          "C030037D09RIK" "C130026I21RIK" "C130074G19RIK" "C3"
 [64] "C5AR2"         "CACNA1I"       "CALD1"         "CALM3"         "CAMK4"         "CARHSP1"       "CASP4"
 [71] "CCDC82"        "CCL5"          "CCNI"          "CCR6"          "CD226"         "CD300LF"       "CD33"
 [78] "CD37"          "CD47"          "CD55"          "CD93"          "CEBPB"         "CEBPD"         "CENPK"
 [85] "CEP89"         "CHAC2"         "CHCHD10"       "CHIL1"         "CHST3"         "CHSY1"         "CIB3"
 [92] "CIR1"          "CITED4"        "CLDN13"        "CLEC4A3"       "CLEC7A"        "CLK3"          "CLN8"
 [99] "CMA1"          "CMTR1"         "CNN2"          "CNNM2"         "COMT"          "CRISPLD2"      "CSPRS"
[106] "CTDSP2"        "CXCR2"         "D130062J21RIK" "D17WSU92E"     "D2HGDH"        "D5ERTD579E"    "D630024D03RIK"
[113] "D9WSU90E"      "DAPK2"         "DAPL1"         "DFNA5"         "DIAP3"         "DIRAS2"        "DNAJA4"
[120] "DNAJC9"        "DNMT3B"        "DNTTIP1"       "DTX1"          "DUSP1"         "DUSP8"         "DYNLT1C"
[127] "DYRK3"         "E130309D14RIK" "E2F2"          "EIF5"          "ENDOD1"        "F5"            "F830002L21RIK"
[134] "FADS3"         "FAM126A"       "FAM32A"        "FAM65B"        "FAM83F"        "FASL"          "FCER2A"
[141] "FCGR1"         "FCGR4"         "FGD2"          "FGFR1OP2"      "FGL2"          "FHDC1"         "FLNA"
[148] "FOSL2"         "FOXP1"         "FPR1"          "FPR2"          "FUNDC2"        "FZR1"          "GAA"
[155] "GBP11"         "GBP7"          "GBP9"          "GCLC"          "GFI1B"         "GID4"          "GIMAP3"
[162] "GIMAP6"        "GM10136"       "GM10144"       "GM10639"       "GM10845"       "GM11564"       "GM12250"
[169] "GM14446"       "GM14548"       "GM14635"       "GM14989"       "GM1966"        "GM3181"        "GM3430"
[176] "GM3697"        "GM3798"        "GM3932"        "GM4015"        "GM4320"        "GM4499"        "GM4951"
[183] "GM5150"        "GM5299"        "GM5483"        "GM6453"        "GM6578"        "GM8884"        "GM8995"
[190] "GM9706"        "GM9790"        "GRAP"          "GSTP2"         "GVIN1"         "GZMA"          "H2-AB1"
[197] "H2-D1"         "H2-DMB1"       "H2-OA"         "H2-OB"         "H2-Q10"        "H2-Q2"         "H2-Q7"
[204] "H2-T10"        "H2-T23"        "H2-T9"         "HCAR2"         "HDC"           "HINT1"         "HIST2H3C1"
[211] "HMGA2-PS1"     "HN1"           "I830012O16RIK" "ICAM4"         "IFI202B"       "IFI204"        "IFI27L2A"
[218] "IFITM2"        "IFITM6"        "IFNAR2"        "IGH-VJ558"     "IGHV14-2"      "IGSF6"         "IGTP"
[225] "IL18BP"        "IL1B"          "IL1F9"         "IL1R2"         "IL4I1"         "INF2"          "IRG1"
[232] "IRGM1"         "IRGM2"         "ITPR2"         "JOSD2"         "JUND"          "KEL"           "KIF21B"
[239] "KLHL25"        "KLRA15"        "KLRA22"        "KLRA23"        "KLRA4"         "KLRA5"         "KLRA7"
[246] "KLRA8"         "KLRA9"         "KLRB1A"        "KLRB1C"        "KLRC1"         "KLRD1"         "LGALS1"
[253] "LIMD2"         "LOC547349"     "LRG1"          "LRP1"          "LRRC25"        "LTB"           "LY6D"
[260] "LY6F"          "LY6I"          "MAD2L1BP"      "MAN2C1"        "MAP2K3"        "MAP4K1"        "MAPRE2"
[267] "MARCKS"        "MAU2"          "MEFV"          "METAP2"        "MGST1"         "MIF4GD"        "MMD"
[274] "MMP8"          "MNDA"          "MPEG1"         "MRAS"          "MRGPRA2A"      "MRGPRA2B"      "MRGPRA5"
[281] "MRGPRA6"       "MRPL32"        "MRPL39"        "MS4A4B"        "MS4A4C"        "MS4A6D"        "MSRB1"
[288] "MTCP1"         "MZT1"          "NACC2"         "NATD1"         "NCF2"          "NCF4"          "NCOR2"
[295] "NFATC1"        "NFE2"          "NKG7"          "NLRC5"         "NOMO1"         "NOSIP"         "NUPR1"
[302] "OAS1A"         "OAS1C"         "OAS1E"         "OAS1F"         "OASL1"         "OASL2"         "OCEL1"
[309] "OGDH"          "OGFOD1"        "OGFR"          "OGFRL1"        "P2RY1"         "P2RY12"        "P2RY13"
[316] "PAQR4"         "PAQR9"         "PCP4L1"        "PDE6H"         "PDLIM4"        "PEG13"         "PFDN2"
[323] "PHF11A"        "PHF11B"        "PHF11D"        "PIGQ"          "PILRA"         "PIRB"          "PKM"
[330] "PLA2G2D"       "PLA2G7"        "PLEKHG2"       "POFUT1"        "POU2AF1"       "POU2F2"        "PPBP"
[337] "PPP1R15A"      "PPP1R3B"       "PPP1R3D"       "PRAM1"         "PRL2C1"        "PROKR1"        "PRR5L"
[344] "PSENEN"        "PTP4A3"        "PTPRCAP"       "PTRF"          "PYGL"          "RAB3IL1"       "RALGPS1"
[351] "RANGAP1"       "RFXANK"        "RGCC"          "RGS12"         "RGS18"         "RHD"           "RNASEH2C"
[358] "RNF114"        "RNF115"        "RNF149"        "RNU1B6"        "RP9"           "RPL14"         "RPS3A1"
[365] "RPS6KB1"       "RPUSD4"        "SAA3"          "SAMM50"        "SCNN1A"        "SEPW1"         "SERPINA3G"
[372] "SERPINB6A"     "SERPINB6B"     "SERPINB6C"     "SETD4"         "SFXN1"         "SH3BGRL2"      "SIGLECE"
[379] "SIRPB1B"       "SKA1"          "SKINT3"        "SLAMF1"        "SLC15A3"       "SLC16A3"       "SLC24A3"
[386] "SLC35B1"       "SLC43A1"       "SLC6A13"       "SLC7A5"        "SLC8A1"        "SLCO1C1"       "SLFN1"
[393] "SLFN4"         "SLFN8"         "SMC6"          "SNX8"          "SOCS3"         "SOD1"          "SORD"
[400] "SPATA2"        "SPHK1"         "SPPL2B"        "SSBP2"         "ST3GAL6"       "ST6GALNAC2"    "ST7"
[407] "STK11IP"       "STK3"          "SULT4A1"       "SYNGR1"        "SYTL3"         "TAB3"          "TANGO6"
[414] "TBX21"         "TG"            "TGM2"          "TGTP2"         "TLN1"          "TLR13"         "TLR2"
[421] "TLR6"          "TMCC2"         "TMEM29"        "TMEM37"        "TMEM86B"       "TNFRSF13B"     "TNFRSF1A"
```

```
[428] "TNFSF14"       "TOR1AIP2"      "TOR3A"         "TPCN1"         "TREM1"         "TRF"           "TRIB3"
[435] "TRIM12C"       "TRIM30A"       "TRIM30B"       "TRIM30D"       "TRIOBP"        "TSPAN12"       "TSPAN8"
[442] "TTC36"         "TTC39A"        "TTPAL"         "TUBA8"         "TUBB2A"        "TUBB2A-PS2"    "TUBB2B"
[449] "TUSC1"         "TXN2"          "TXNRD2"        "UBAP1"         "UBE2O"         "UGT1A6B"       "UROS"
[456] "VILL"          "VIPR1"         "VMP1"          "VWF"           "WDFY1"         "WDFY4"         "WDR48"
[463] "WFDC17"        "WHSC1"         "XKRX"          "XLR3B"         "XLR4A"         "XLR4B"         "YAP1"
[470] "YIPF4"         "ZIC1"          "ZKSCAN1"

$Tang
  [1] "A1BG"          "A2M-AS1"       "ABCA1"         "ABHD14B"       "ABLIM1"        "ACADSB"        "ACAT1"
  [8] "ACCS"          "ACKR3"         "ACOT13"        "ACOXL"         "ACRBP"         "ADAMTS10"      "ADARB1"
 [15] "ADCY4"         "ADRB2"         "AES"           "AKAP11"        "AKIRIN2"       "ALG13"         "AMIGO1"
 [22] "AMPD2"         "ANK3"          "ANKRD22"       "ANKRD36"       "ANKRD46"       "ANKRD9"        "ANKS6"
 [29] "ANXA8L1"       "AP5B1"         "APBA2"         "APOBEC3B"      "APOBEC3H"      "AQP1"          "AQP10"
 [36] "ARG1"          "ARHGAP5"       "ARHGEF40"      "ARL17A"        "ARL4A"         "ARL4C"         "ARNTL"
 [43] "ARPC3"         "ARPC5"         "ASGR2"         "ASPH"          "ASPM"          "ATF1"          "ATP5I"
 [50] "ATP5J"         "ATP6"          "ATP6V0E2-AS1"  "AUTS2"         "B4GALT5"       "BAALC"         "BACH2"
 [57] "BAHD1"         "BATF2"         "BCL11A"        "BCL11B"        "BCL2A1"        "BIRC3"         "BIRC5"
 [64] "BLK"           "BRD1"          "BTG1"          "BTN3A1"        "BUB1"          "BZRAP1-AS1"    "C10orf105"
 [71] "C12orf57"      "C16orf74"      "C1orf52"       "C20orf141"     "C2orf40"       "C2orf88"       "C4orf33"
 [78] "C5orf45"       "C6orf48"       "CA2"           "CACNA1E"       "CACNG6"        "CAMK1D"        "CAP1"
 [85] "CARD11"        "CARD16"        "CARD17"        "CARD6"         "CASC15"        "CASC5"         "CBLB"
 [92] "CBX7"          "CCDC102A"      "CCDC144A"      "CCDC147-AS1"   "CCNA2"         "CCNB1"         "CCR3"
 [99] "CD164"         "CD177"         "CD2"           "CD22"          "CD247"         "CD28"          "CD300E"
[106] "CD3E"          "CD40LG"        "CD5"           "CD6"           "CD63"          "CD7"           "CD81"
[113] "CD8A"          "CD8B"          "CDC20"         "CDC42SE1"      "CDC45"         "CDCA2"         "CDCA3"
[120] "CDCA5"         "CDK1"          "CDK11A"        "CDK11B"        "CDKN2C"        "CDKN3"         "CDT1"
[127] "CDV3"          "CENPF"         "CENPM"         "CENPN"         "CENPU"         "CENPW"         "CEP19"
[134] "CES1"          "CFAP97"        "CHEK1"         "CHIT1"         "CHPT1"         "CHRM3-AS2"     "CISD2"
[141] "CIT"           "CKAP4"         "CKLF"          "CKS1B"         "CKS2"          "CLEC10A"       "CLEC11A"
[148] "CLEC12A"       "CLEC1B"        "CLUHP3"        "CMTM5"         "CNIH4"         "CNR2"          "CNTNAP3"
[155] "COQ10A"        "COX17"         "COX7B"         "CR1"           "CREG1"         "CRHR1-IT1"     "CRIP2"
[162] "CROCC"         "CRTC1"         "CSGALNACT2"    "CSTA"          "CTNNAL1"       "CTSF"          "CTSL"
[169] "CTTN"          "CYB561A3"      "CYSTM1"        "CYTH3"         "DBI"           "DCAF16"        "DDAH2"
[176] "DEFA8P"        "DENND2D"       "DHFR"          "DIAPH3"        "DISC1"         "DLGAP5"        "DNAJA1P5"
[183] "DNAJA3"        "DNAJC30"       "DNAJC5"        "DOCK10"        "DPP4"          "DPRXP4"        "DRC1"
[190] "DTL"           "DUSP13"        "DUT"           "DYRK2"         "E2F1"          "E2F7"          "ECRP"
[197] "EEF1A1"        "EEF2K"         "EIF1AX"        "EIF3F"         "EIF4A2"        "EIF4G3"        "ENHO"
[204] "ENO2"          "ENY2"          "EPHX2"         "ERN1"          "ESPL1"         "ESRG"          "EVI2A"
[211] "EVL"           "EZH2"          "FABP5"         "FAM102A"       "FAM117B"       "FAM129C"       "FAM134B"
[218] "FAM155A-IT1"   "FAM159A"       "FAM177B"       "FAM20A"        "FAM26F"        "FAM27C"        "FANCF"
[225] "FANCI"         "FBL"           "FBLN5"         "FBXL16"        "FCAR"          "FCER1A"        "FCER2"
[232] "FCGBP"         "FCGR1A"        "FCN1"          "FCRL1"         "FCRL2"         "FCRL3"         "FCRL6"
[239] "FFAR3"         "FGD4"          "FLT3LG"        "FOXJ3"         "FOXM1"         "FRMD3"         "FSD1"
[246] "FSTL3"         "FTL"           "FXYD5"         "G0S2"          "GADD45G"       "GALM"          "GALNT14"
[253] "GATA3"         "GBP3"          "GCSAM"         "GDF15"         "GGH"           "GINS1"         "GINS2"
[260] "GLCCI1"        "GLIPR2"        "GLS"           "GLTSCR2"       "GMFG"          "GMNN"          "GNB2L1"
[267] "GNG10"         "GNG11"         "GOLGA8A"       "GP1BB"         "GPA33"         "GPD2"          "GPER1"
[274] "GPR141"        "GPR174"        "GPR18"         "GPR183"        "GPR68"         "GPR84"         "GPRASP1"
[281] "GRB10"         "GRN"           "GRPEL2"        "GSN"           "GSTM3"         "GTF2H5"        "GTSE1"
[288] "GVINP1"        "GYG1"          "GZMK"          "GZMM"          "H1F0"          "HABP4"         "HAPLN3"
[295] "HBM"           "HCG18"         "HEBP2"         "HELZ2"         "HES6"          "HIP1R"         "HIST1H1B"
[302] "HIST1H2AB"     "HIST1H2AC"     "HIST1H2AD"     "HIST1H2AE"     "HIST1H2AG"     "HIST1H2AH"     "HIST1H2AI"
[309] "HIST1H2AJ"     "HIST1H2AK"     "HIST1H2AL"     "HIST1H2AM"     "HIST1H2BB"     "HIST1H2BC"     "HIST1H2BD"
[316] "HIST1H2BE"     "HIST1H2BF"     "HIST1H2BG"     "HIST1H2BH"     "HIST1H2BI"     "HIST1H2BJ"     "HIST1H2BK"
[323] "HIST1H2BL"     "HIST1H2BM"     "HIST1H2BO"     "HIST1H3B"      "HIST1H3D"      "HIST1H3F"      "HIST1H3H"
[330] "HIST1H3J"      "HIST1H4D"      "HIST1H4F"      "HIST1H4K"      "HIST1H4L"      "HIST2H2AA4"    "HIST2H2AB"
[337] "HIST2H3A"      "HJURP"         "HK3"           "HLA-DOA"       "HLA-DOB"       "HLA-DPA1"      "HLA-DPB1"
[344] "HLA-DPB2"      "HLA-DQA2"      "HLA-DQB1"      "HLA-DQB2"      "HLA-DRA"       "HLA-DRB3"      "HMGB1"
[351] "HMGB2"         "HMGB3P1"       "HMMR"          "HNRNPA1"       "HNRNPH1"       "HOXB2"         "HP1BP3"
[358] "HPR"           "HRASLS2"       "HS3ST1"        "HSP90AA1"      "HSPA8"         "ICOS"          "IFFO2"
[365] "IFI27L1"       "IFI27L2"       "IFITM4P"       "IFT20"         "IGFBP7"        "IGJ"           "IGLL1"
[372] "IGLL5"         "IKZF2"         "IL10RB-AS1"    "IL11RA"        "IL18R1"        "IL18RAP"       "IL1RAP"
```

```
[379] "IL23A"      "IL27"       "IL2RB"      "IL32"       "IL7R"       "ING5"       "INHBA"
[386] "INPP4B"     "INPP5E"     "IRAK3"      "ISCA1"      "ISY1"       "ITGA2B"     "ITGA7"
[393] "ITIH4"      "ITK"        "JDP2"       "JPH4"       "KCNG1"      "KDF1"       "KIAA0101"
[400] "KIAA0226L"  "KIAA0319L"  "KIAA0355"   "KIAA1147"   "KIF11"      "KIF1B"      "KIF2C"
[407] "KLF12"      "KLHDC7B"    "KLHDC8B"    "KLHL3"      "KLRC3"      "KLRC4"      "KRTAP10-6"
[414] "LAT"        "LBH"        "LCK"        "LCN2"       "LDLRAP1"    "LEF1-AS1"   "LEPROTL1"
[421] "LILRA3"     "LILRA5"     "LILRA6"     "LILRB3"     "LIN7A"      "LIPN"       "LLGL2"
[428] "LMF1"       "LOXL3"      "LPIN1"      "LRFN3"      "LRIG1"      "LRRC47"     "LRRN3"
[435] "LSM3"       "LTB4R"      "LTBP3"      "LTF"        "LY9"        "LY96"       "MAL"
[442] "MAMSTR"     "MAN1C1"     "MAOA"       "MAP1LC3B"   "MAPK14"     "MATK"       "MB21D1"
[449] "MCF2L-AS1"  "MCM2"       "MCM4"       "MEAF6"      "MEGF6"      "MEX3C"      "MEX3D"
[456] "MGAM"       "MGC16025"   "MGLL"       "MILR1"      "MIR146A"    "MIR4435-1HG" "MKI67"
[463] "MLLT6"      "MME"        "MOAP1"      "MPO"        "MRC2"       "MRPL22"     "MRPL27"
[470] "MRPL51"     "MRPS18C"    "MSN"        "MSX2P1"     "MT1B"       "MT1E"       "MT1HL1"
[477] "MT1L"       "MT1M"       "MTA1"       "MXD4"       "MXI1"       "MXRA7"      "MYBL1"
[484] "MYBL2"      "MYBPC3"     "MYC"        "MYCL"       "MYL6"       "MYL6B"      "MYL9"
[491] "MZB1"       "NAB2"       "NAIP"       "NAP1L1"     "NCR3"       "ND6"        "NDRG2"
[498] "NDST2"      "NDUFA1"     "NDUFA4"     "NDUFA6"     "NDUFB3"     "NELL2"      "NEXN"
[505] "NIPA1"      "NKTR"       "NKX3-1"     "NLRC3"      "NLRC4"      "NMUR1"      "NOG"
[512] "NOP10"      "NRIR"       "NSG1"       "NT5C3A"     "NT5E"       "NT5M"       "NTNG2"
[519] "NTSR1"      "NUSAP1"     "OBFC1"      "ODF3B"      "OIP5"       "OPLAH"      "OR52K2"
[526] "OSCAR"      "OXNAD1"     "P2RX5"      "P2RY10"     "PABPC4"     "PASK"       "PCED1B"
[533] "PCMT1"      "PCNA"       "PCSK1N"     "PDE4DIP"    "PDP1"       "PEBP1"      "PHC3"
[540] "PHF19"      "PID1"       "PIK3AP1"    "PIK3C2B"    "PIN4"       "PIWIL4"     "PJA1"
[547] "PKIA"       "PKMYT1"     "PLBD1"      "PLCG1"      "PLEKHA1"    "PLEKHB1"    "PLEKHG4"
[554] "PLVAP"      "PLXDC1"     "PLXNA1"     "PMEPA1"     "PNMA1"      "PNRC1"      "POLG2"
[561] "POLRMT"     "PP7080"     "PPARD"      "PPIB"       "PQLC1"      "PRC1"       "PRDX4"
[568] "PREPL"      "PRKAG2-AS1" "PRKAR1B"    "PRKAR2B"    "PRKCA"      "PRKCH"      "PRKCQ-AS1"
[575] "PRNP"       "PRRG4"      "PRUNE2"     "PSIP1"      "PSMA2"      "PSMA3"      "PSMA4"
[582] "PSMA6"      "PSMC2"      "PSMF1"      "PTCRA"      "PTGDR"      "PTGDR2"     "PTPRK"
[589] "PTTG1"      "PTTG2"      "PTX3"       "PURA"       "PYCR1"      "RAB11FIP3"  "RAB13"
[596] "RABL2A"     "RAD21"      "RAD51"      "RAD54L"     "RAI1"       "RALGAPA1"   "RARA-AS1"
[603] "RASA4"      "RASGRP1"    "RBM20"      "RBX1"       "RCAN3"      "REC8"       "RECQL4"
[610] "RETN"       "RFTN1"      "RGL4"       "RHOH"       "RMI2"       "RNA5-8S5"   "RNASE1"
[617] "RNF208"     "RNF32"      "RNF44"      "RORC"       "RPA3"       "RPL10A"     "RPL10L"
[624] "RPL13"      "RPL15"      "RPL18"      "RPL18A"     "RPL19P12"   "RPL22"      "RPL22L1"
[631] "RPL23A"     "RPL23AP7"   "RPL26L1"    "RPL29"      "RPL29P2"    "RPL3"       "RPL4"
[638] "RPL5"       "RPL7A"      "RPLP2"      "RPS13"      "RPS14"      "RPS16"      "RPS18"
[645] "RPS2"       "RPS23"      "RPS27A"     "RPS27L"     "RPS28"      "RPS2P32"    "RPS6KA5"
[652] "RPSAP9"     "RRAGD"      "RRAS2"      "RRBP1"      "RSAD1"      "RSPH9"      "RTN1"
[659] "RUNX3"      "S100A12"    "S100P"      "S100PBP"    "SACS"       "SAMSN1"     "SAP30"
[666] "SARDH"      "SARM1"      "SBK1"       "SCAI"       "SCARF1"     "SCARNA17"   "SCML4"
[673] "SDC1"       "SEC11C"     "SELL"       "SELM"       "SEMA4C"     "Sep 01"     "SERTAD2"
[680] "SF3B6"      "SFI1"       "SGK223"     "SH2D1B"     "SH2D3A"     "SH3YL1"     "SHCBP1"
[687] "SIDT1"      "SIGLEC17P"  "SIGLEC5"    "SIPA1L2"    "SIT1"       "SKAP1"      "SLC1A3"
[694] "SLC22A15"   "SLC22A4"    "SLC25A6"    "SLC26A11"   "SLC26A8"    "SLC38A1"    "SLC45A3"
[701] "SLC6A19"    "SLED1"      "SLIRP"      "SMA4"       "SMARCD3"    "SMC1A"      "SMPD3"
[708] "SNAR-B2"    "SNAR-D"     "SNAR-H"     "SNHG6"      "SNORA54"    "SNORD105B"  "SNORD46"
[715] "SNORD68"    "SNRPD1"     "SNRPG"      "SNX22"      "SPAG5"      "SPC24"      "SPC25"
[722] "SPCS3"      "SPOCK2"     "SRGAP2"     "SRGAP2B"    "SRGN"       "SRP19"      "SRSF10"
[729] "SRSF8"      "ST6GAL1"    "STAB1"      "STAC3"      "STAT4"      "STIL"       "STK26"
[736] "STMN1"      "STMN3"      "STOM"       "STRBP"      "SUB1"       "SUN1"       "SUSD6"
[743] "TAGAP"      "TANK"       "TC2N"       "TCF7"       "TCL1A"      "TCTN1"      "TFDP1"
[750] "TIGD3"      "TIMM8B"     "TK1"        "TLE2"       "TLR5"       "TMCC1"      "TMEM106B"
[757] "TMEM109"    "TMEM119"    "TMEM204"    "TMEM209"    "TMEM243"    "TMEM25"     "TMEM263"
[764] "TNFRSF17"   "TNFRSF25"   "TOP1MT"     "TP53I3"     "TPM4"       "TPST1"      "TPX2"
[771] "TRABD2A"    "TRAF1"      "TRAF5"      "TRAK1"      "TRG-AS1"    "TRGV7"      "TRIM34"
[778] "TRIM4"      "TRIM54"     "TROAP"      "TSHZ1"      "TSPAN3"     "TSPYL4"     "TTC3"
[785] "TUBE1"      "TXN"        "TXNDC5"     "TYMS"       "UBASH3A"    "UBE2C"      "UBE2L3"
[792] "UBE2Q2"     "UBE2T"      "UBE2V1"     "UCHL1"      "UNCX"       "UPP1"       "UQCRB"
[799] "UQCRQ"      "USP41"      "VEGFA"      "VEGFB"      "VIM"        "VNN1"       "VPS13C"
[806] "VPS37C"     "VPS51"      "WDR86"      "WFDC3"      "ZAP70"      "ZBED6CL"    "ZBTB11"
[813] "ZBTB32"     "ZBTB4"      "ZBTB40"     "ZCCHC14"    "ZDHHC11"    "ZFP62"      "ZFYVE28"
```

```
[820] "ZMAT1"        "ZNF12"        "ZNF204P"      "ZNF205"       "ZNF286B"      "ZNF529"       "ZNF550"
[827] "ZNF684"       "ZNF831"       "ZNF837"       "ZNF90"        "ZSCAN18"      "ZSWIM7"       "ZWINT"
[834] "ZXDB"

$Woods
  [1] "ABAT"                                                 "ABCA2"
  [3] "ABCC6"                                                "ACOT9"
  [5] "ADAMTS5"                                              "AFF1"
  [7] "ANPEP"                                                "AP1M2"
  [9] "APOBEC1"                                              "APOBEC3G"
 [11] "APOL1"                                                "ARHGAP32"
 [13] "ARL3"                                                 "ART3"
 [15] "ATF3"                                                 "ATP2B2"
 [17] "ATP6V1A"                                              "BAGE"
 [19] "BATF3"                                                "BPY2"
 [21] "BRCA2"                                                "C10orf84"
 [23] "C11orf75"                                             "C18orf25"
 [25] "C19orf66"                                             "C1GALT1"
 [27] "C1orf106"                                             "C22orf28"
 [29] "C9orf91"                                              "CA4"
 [31] "CACNA1A"                                              "CASP5"
 [33] "CASS4"                                                "CBR1"
 [35] "CCL2"                                                 "CCL8"
 [37] "CCNA1"                                                "CCR1"
 [39] "CD2AP"                                                "CDKN1A"
 [41] "CDKN1C"                                               "CENPI"
 [43] "CFTR"                                                 "CMKLR1"
 [45] "CNP"                                                  "COBLL1"
 [47] "CPB1"                                                 "CPEB3"
 [49] "CRYAB"                                                "CST4"
 [51] "CT62"                                                 "CWH43"
 [53] "CXCL6"                                                "CXorf21"
 [55] "DAAM2"                                                "DDO"
 [57] "DNAJC15"                                              "DPEP3"
 [59] "DUSP3"                                                "DUSP5"
 [61] "DUX1"                                                 "EGR2"
 [63] "ENPP2"                                                "EPB41L3"
 [65] "EPB41L4A"                                             "EPHA4"
 [67] "EPHB2"                                                "EPS8"
 [69] "FAM70A"                                               "FANCA"
 [71] "FANCL"                                                "FCGR1A /// FCGR1C"
 [73] "FCGR2B"                                               "FFAR2"
 [75] "FTSJD2"                                               "FUT4"
 [77] "FZD5"                                                 "GADD45B"
 [79] "GALNT3"                                               "GK"
 [81] "GOLGA6L4 /// PML"                                     "GRAMD1C"
 [83] "GSTA1"                                                "GTPBP2"
 [85] "GUCA1A"                                               "GUCY1B3"
 [87] "HBE1"                                                 "HESX1"
 [89] "HEY1"                                                 "HFE"
 [91] "HGF"                                                  "HPSE"
 [93] "ICA1"                                                 "ID3"
 [95] "IDO1"                                                 "IDS"
 [97] "IGF2R"                                                "IL10"
 [99] "IL15"                                                 "IRS2"
[101] "ITGB4"                                                "JUP"
[103] "KAZ"                                                  "KCND1"
[105] "KCNK10"                                               "KCTD14"
[107] "KIAA0226"                                             "KIAA1324"
[109] "KIT"                                                  "KMO"
[111] "KPTN"                                                 "KSR1"
[113] "KYNU"                                                 "LCAT"
[115] "LEP"                                                  "LOC100505584 /// MT1E"
```

```
[117] "LOC51145"                                           "LOC652346 /// PML"
[119] "LPAR1"                                              "LPAR6"
[121] "LPHN3"                                              "LTK"
[123] "MAP7"                                               "MAST3"
[125] "MDC1"                                               "MDK"
[127] "MET"                                                "METT10D"
[129] "METTL7A"                                            "MGP"
[131] "MICB"                                               "MMP9"
[133] "MMRN1"                                              "MR1"
[135] "MSR1"                                               "MT1F"
[137] "MT1G"                                               "MT1H"
[139] "MT1P2"                                              "MT1X"
[141] "MTHFD2"                                             "MTHFR"
[143] "MTTP"                                               "MYO7A"
[145] "MYOF"                                               "MZF1"
[147] "NECAB2"                                             "NEUROD2"
[149] "NF2"                                                "NFATC4"
[151] "NR2F6"                                              "NR4A2"
[153] "NSUN7"                                              "NTRK3"
[155] "OMD"                                                "OSBPL7"
[157] "P2RX7"                                              "P2RY14"
[159] "P2RY6"                                              "PADI4"
[161] "PARP11"                                             "PBXIP1"
[163] "PDZD8"                                              "PEX7"
[165] "PGAP1"                                              "PLEKHG3"
[167] "PLOD2"                                              "POLR3G"
[169] "PPY2"                                               "PRB4"
[171] "PRKDC"                                              "PSG2"
[173] "PSMB9"                                              "PTP4A1"
[175] "PTPRO"                                              "RAB27B"
[177] "RANBP1"                                             "RBCK1"
[179] "RBM17"                                              "RBMS2"
[181] "RCN3"                                               "RFX2"
[183] "RGL1"                                               "RHAG"
[185] "RHBDF1"                                             "RIN2"
[187] "RPGRIP1"                                            "RRAS"
[189] "SCAND2"                                             "SCN11A"
[191] "SDC2"                                               "SDC3"
[193] "SEC24D"                                             "SEC61A2"
[195] "SECTM1"                                             "SEPP1"
[197] "SEPT4"                                              "SLAMF7"
[199] "SLC12A8"                                            "SLC25A24"
[201] "SLC27A3"                                            "SLC38A3"
[203] "SLC5A7"                                             "SLC6A2"
[205] "SLFN12"                                             "SNRNP70"
[207] "SOBP"                                               "SORT1"
[209] "SOX3"                                               "SOX4"
[211] "SRBD1"                                              "SYCP1"
[213] "SYN2"                                               "TAP2"
[215] "TAZ"                                                "TMEM62"
[217] "TMX1"                                               "TNIP3"
[219] "TRIM21"                                             "TSHR"
[221] "UBE4B"                                              "VAMP4"
[223] "VRK2"                                               "WSB2"
[225] "XAGE1A /// XAGE1B /// XAGE1C /// XAGE1D /// XAGE1E" "ZNF248"

$Zhai
 [1] ""             "ADAR"         "ADIPOR1"      "ALPL"         "ASCC2"        "BCL2L1"       "BSG"
 [8] "CECR1"        "CSDA"         "CX3CR1"       "DPYSL5"       "ECGF1"        "EEF1G"        "EPB42"
[15] "FBXO7"        "GBP4"         "GBP5"         "GIMAP4"       "GPR175"       "GSPT1"        "HAGH"
[22] "HBD"          "HBG1"         "HBG2"         "HIST2H2AA3"   "HLA-F"        "ICAM3"        "IMPA2"
[29] "LOC100008589" "LOC100128326" "LOC100129681" "LOC100130914" "LOC100131164" "LOC100131726" "LOC100132394"
[36] "LOC100134530" "LOC100134634" "LOC389386"    "LOC389599"    "LOC401357"    "LOC440313"    "LOC440359"
```

```
[43] "LOC642357"    "LOC642469"    "LOC643319"    "LOC643384"    "LOC644852"    "LOC645173"    "LOC648390"
[50] "LOC729021"    "LOC729660"    "LOC730286"    "MAP1S"        "MT1A"         "MUC6"         "MYL12A"
[57] "NT5C3"        "PI3"          "PPM1F"        "PRIC285"      "RBM38"        "RPS5"         "SELENBP1"
[64] "SERPINA13"    "SIGLEC14"     "SLC25A37"     "SLC25A39"     "SNCA"         "SORL1"        "SPRYD3"
[71] "STRADB"       "TBL1X"        "TMEM140"      "TSPAN5"       "TXNDC12"      "TYMP"         "UBXN6"
[78] "WDR40A"

$CC_resis_ctrTang
 [1] "AIF1"      "ANP32E"    "ATOX1"     "ATP5E"     "B2M"       "BANK1"     "BTLA"      "CAMP"      "CCNB2"
[10] "CCR7"      "CD19"      "CD274"     "CD74"      "CD79B"     "CD83"      "CD96"      "CDCA8"     "CLEC4D"
[19] "CLU"       "COX7A2"    "CR2"       "CXCR5"     "DHRS3"     "DTX3L"     "EIF4B"     "EOMES"     "FAIM3"
[28] "FCRLA"     "FOXO1"     "GADD45A"   "GBP2"      "HVCN1"     "IMP3"      "ITGB3"     "KLRB1"     "KLRG1"
[37] "MCEMP1"    "MCTP1"     "MS4A1"     "MXD1"      "PRPF8"     "RPL13A"    "RYK"       "S100A6"    "S100A9"
[46] "S1PR1"     "SAMD3"     "SPIB"      "TGFBI"     "THRA"      "TMEM42"    "TNFRSF13C" "TNNT1"     "UHRF1"
[55] "VPREB3"

$CC_resis_ctrWoods
[1] "CFB"      "GCH1"     "LGALS3BP" "MYH9"     "SPTAN1"   "TDRD7"    "TLR7"     "TRAFD1"   "TREX1"

$CC_resis_ctrZhai
[1] "GYPC"   "GZMB"   "TAGLN2" "TESC"

$TangWoods
 [1] "AIM2"     "ANXA3"    "APOBEC3A" "APOL6"    "C1QA"     "C1QB"     "C2"       "C3AR1"    "CCRL2"    "CD38"
[11] "CLIC4"    "CXCR3"    "DDX58"    "EPN2"     "ETV7"     "FHL2"     "FKBP5"    "GYPA"     "HIST1H4H" "IFIT5"
[21] "LHFPL2"   "LMNB1"    "MAP2K6"   "MARCO"    "MS4A4A"   "MT2A"     "NDC80"    "NMI"      "NOV"      "PML"
[31] "PTGER4"   "RECK"     "SAMD4A"   "SGK1"     "SIGLEC1"  "SOCS1"    "SPTLC2"   "SSB"      "TCN1"     "TCN2"
[41] "TFEC"     "THBD"     "TMEM51"   "TOR1B"    "TRIM14"   "ZC3HAV1"  "ZCCHC2"

$TangZhai
 [1] "ADM"      "CASP1"    "DDX60L"   "DYNLT1"   "EEF2"     "EIF3L"    "ELF1"     "EMR3"     "EPSTI1"   "FBXO6"
[11] "HES4"     "IFI30"    "NCOA7"    "PARP10"   "RNASE2"   "RPS3"     "RPS4X"    "SHISA5"   "TNFSF13B"

$WoodsZhai
 [1] "CTSL1"  "DPEP2"  "DRAP1"  "LAMP3"  "LMO2"   "MAFB"   "PHF11"  "PSME2"  "SP110"  "UBE2L6" "WARS"

$CC_resis_ctrTangWoods
[1] "AGRN"   "HP"     "ITPR3"  "LEF1"   "LILRB4" "SP100"  "SP140"  "UBE2S"

$CC_resis_ctrTangZhai
[1] "CMPK2"  "IFITM3" "IRF9"   "PARP14" "PARP9"  "RNF213" "S100A8" "SAMD9L"

$CC_resis_ctrWoodsZhai
[1] "CXCL10"  "LGALS9"  "MX2"     "PARP12"  "STAT2"   "TMEM123"

$TangWoodsZhai
 [1] "BLVRA"    "CEACAM1"  "CHMP5"    "DDX60"    "DHRS9"    "EIF2AK2"  "FAM46A"   "FCGR1B"   "GBP1"     "GLRX"
[11] "HERC5"    "IFI16"    "IFI44L"   "IFI6"     "IL1RN"    "ISG20"    "LAP3"     "LY6E"     "OAS1"     "OASL"
[21] "OTOF"     "PLSCR1"   "SAMD9"    "SAT1"     "SCARB2"   "SCO2"     "SERPING1" "SPATS2L"  "STAT1"    "TAP1"
[31] "TIMM10"   "TNFAIP6"  "TNFSF10"  "TRIM22"   "TRIM5"    "VAMP5"

$CC_resis_ctrTangWoodsZhai
 [1] "BST2"   "DHX58"  "HERC6"  "IFI27"  "IFI35"  "IFI44"  "IFIH1"  "IFIT1"  "IFIT2"  "IFIT3"  "IFITM1" "IRF7"   "ISG15"
[14] "MX1"    "OAS2"   "OAS3"   "PLAC8"  "RSAD2"  "RTP4"   "USP18"  "XAF1"   "ZBP1"
```
