# Supplementary material for: Of mice and men: the host response to influenza virus infection
Source: Mamm Genome. 2018 Jun 15;29(7):446–70. doi: 10.1007/s00335-018-9750-y (PMC6132725; doi:10.1007/s00335-018-9750-y)
Supplement: Supplementary file 13 — Supplementary material 13 (PDF 26 KB) [file 335_2018_9750_MOESM13_ESM.pdf]

VENN gene lists severe versus control for CC strains

```
$CC_svre_ctr
  [1] "0610040J01RIK"  "1110007C09RIK"  "1110008F13RIK"  "1190002F15RIK"  "1500011B03RIK"  "1600012P17RIK"
  [7] "1600014C10RIK"  "1700007K09RIK"  "1700016P03RIK"  "1700018A04RIK"  "1700019A02RIK"  "1700019D03RIK"
 [13] "1700037H04RIK"  "1700047G07RIK"  "1700061N14RIK"  "1700111N16RIK"  "1700112E06RIK"  "1700122H20RIK"
 [19] "1810073O08RIK"  "2010107E04RIK"  "2310033P09RIK"  "2310079G19RIK"  "2410024N13RIK"  "2610035D17RIK"
 [25] "2900040C04RIK"  "2900052N01RIK"  "2900076A13RIK"  "3110021A11RIK"  "3110062M04RIK"  "4632428N05RIK"
 [31] "4732465J04RIK"  "4733401D01RIK"  "4921518K17RIK"  "4921523P09RIK"  "4930449E01RIK"  "4930549C15RIK"
 [37] "5230400M03RIK"  "5430416O09RIK"  "5430431A17RIK"  "5830416P10RIK"  "6330416G13RIK"  "6430548M08RIK"
 [43] "9030619P08RIK"  "9330151L19RIK"  "9430063H18RIK"  "9930111J21RIK1" "A130082J08RIK"  "A230092J17RIK"
 [49] "A430078G23RIK"  "A530064D06RIK"  "A630033H20RIK"  "AANAT"          "AB124611"       "ACMSD"
 [55] "ACOT5"          "ACOT7"          "ACP1"           "ACRV1"          "ACSL1"          "ACTB"
 [61] "ACTG1"          "ACTL7A"         "ADAM15"         "ADAM5"          "ADAM8"          "ADCY9"
 [67] "ADRA1B"         "ADSSL1"         "AF251705"       "AGO2"           "AKR1A1"         "AKT1"
 [73] "ALAS1"          "ALDH2"          "ALDH3B1"        "ALDH3B2"        "ALDOA"          "ALOX12"
 [79] "ALOX5"          "ALOX5AP"        "AMICA1"         "ANK"            "ANKRD2"         "ANXA1"
 [85] "ANXA11"         "ANXA2"          "APOBR"          "APOL9B"         "APP"            "ARAP3"
 [91] "ARG2"           "ARHGAP18"       "ARHGDIB"        "ARHGEF10L"      "ARID3A"         "ARID5A"
 [97] "ARMCX6"         "ARPC1B"         "ARPC2"          "ASB7"           "ASIC3"          "ASPRV1"
[103] "ATF7"           "ATP11A"         "ATP1A3"         "ATP2A3"         "ATP5K"          "ATP8B3"
[109] "ATP9A"          "AU015791"       "AW011738"       "AW011956"       "AW555355"       "AZIN1"
[115] "B430306N03RIK"  "B4GALT3"        "BAK1"           "BC052688"       "BC094916"       "BC100530"
[121] "BC147527"       "BCL3"           "BCL6"           "BCR"            "BCS1L"          "BIN3"
[127] "BMP1"           "BRD2"           "BST1"           "C030037D09RIK"  "C130093G08RIK"  "C1GALT1C1"
[133] "C1QL2"          "C3"             "C5AR2"          "C81489"         "CALD1"          "CALN1"
[139] "CALY"           "CAMKV"          "CAPN2"          "CAPZB"          "CAR2"           "CAR7"
[145] "CARHSP1"        "CASK"           "CASP4"          "CBR3"           "CCDC153"        "CCDC167"
[151] "CCDC82"         "CCDC92"         "CCL4"           "CCL6"           "CCL9"           "CCND3"
[157] "CCR2"           "CCR6"           "CD14"           "CD151"          "CD27"           "CD300C"
[163] "CD300LD"        "CD300LF"        "CD302"          "CD33"           "CD44"           "CD55"
[169] "CD80"           "CD8B1"          "CD93"           "CD97"           "CDC25B"         "CDK2AP2"
[175] "CDKN2D"         "CDR2"           "CDS2"           "CEBPB"          "CEBPD"          "CEP68"
[181] "CFAP74"         "CFL1"           "CFP"            "CHCHD10"        "CHIL1"          "CHST12"
[187] "CHST3"          "CHSY1"          "CIDEB"          "CISD3"          "CLEC4A1"        "CLEC4A3"
[193] "CLEC4E"         "CLEC7A"         "CLIC1"          "CMAS"           "CMTM6"          "CMTR1"
[199] "CNN1"           "CNN2"           "CNST"           "CNTN6"          "COG5"           "COTL1"
[205] "COX6B2"         "COX6C"          "CPD"            "CRISPLD2"       "CRLF2"          "CRYGF"
[211] "CRYZL1"         "CSF2RA"         "CSF2RB"         "CSF2RB2"        "CSN1S2B"        "CSPRS"
[217] "CSRNP1"         "CSRP1"          "CST3"           "CTLA2A"         "CTLA2B"         "CTNNBIP1"
[223] "CTRL"           "CTSA"           "CTSC"           "CTSD"           "CTSS"           "CTSZ"
[229] "CUEDC2"         "CUL4A"          "CXCR2"          "CYBA"           "CYP21A1"        "CYP2B10"
[235] "CYP2D11"        "CYP4A12B"       "CYP4B1"         "CYP4B1-PS2"     "CYP4F17"        "CYP4F18"
[241] "D030029J20RIK"  "D130012P04RIK"  "D2HGDH"         "D430041D05RIK"  "D4ERTD681E"     "D630029K05RIK"
[247] "D830030K20RIK"  "DAPP1"          "DAXX"           "DCAF7"          "DCLRE1C"        "DCUN1D1"
[253] "DEDD2"          "DEFA-RS2"       "DEFA1"          "DEFA23"         "DEFB9"          "DENND5A"
[259] "DFNA5"          "DGAT1"          "DGAT2"          "DHCR24"         "DHRS11"         "DHRS7"
[265] "DIO1"           "DLGAP4"         "DNAH17"         "DNAJA4"         "DNAJB2"         "DNAJB6"
[271] "DNAJC14"        "DOC2A"          "DOK3"           "DPP6"           "DPYSL4"         "DSTN"
[277] "DTNB"           "DUSP1"          "DUSP16"         "DUSP23"         "DUSP8"          "E130119H09RIK"
[283] "E330014E10RIK"  "EBI3"           "ECM1"           "EFHD2"          "EFS"            "EHBP1L1"
[289] "EHD4"           "EIF2D"          "EIF4EBP1"       "ELL2"           "ELOVL1"         "EMB"
[295] "EME1"           "EMILIN2"        "ENTPD3"         "EPHX1"          "EPOR"           "ERGIC3"
[301] "ERICH5"         "ESD"            "ETFB"           "F10"            "F11R"           "F13A1"
[307] "F420015M19RIK"  "F5"             "F830002L21RIK"  "F830014O18RIK"  "FADS1"          "FAM110A"
[313] "FAM216B"        "FAM71F1"        "FBXL5"          "FBXO31"         "FBXO34"         "FBXO9"
[319] "FBXW21"         "FCER1G"         "FCGR1"          "FCGR3"          "FCGR4"          "FCNAOS"
[325] "FERMT3"         "FES"            "FGF8"           "FGFBP3"         "FGL2"           "FGR"
[331] "FHL5"           "FKRP"           "FLNA"           "FLOT1"          "FN1"            "FOSL2"
[337] "FPR1"           "FPR2"           "FUOM"           "FURIN"          "FYB"            "G6B"
[343] "G6PDX"          "GABARAPL1"      "GALT"           "GAPDH"          "GAS2L1"         "GATA1"
[349] "GATA2"          "GBP6"           "GBP7"           "GBP9"           "GCNT2"          "GDAP10"
[355] "GDPD2"          "GFI1B"          "GFRA4"          "GKN3"           "GLIPR1"         "GLRX5"
[361] "GM10272"        "GM10693"        "GM10845"        "GM11127"        "GM11559"        "GM11564"
```

```
[367] "GM11937"       "GM11938"       "GM12060"       "GM12070"       "GM12250"       "GM12271"
[373] "GM13375"       "GM14206"       "GM14446"       "GM14548"       "GM15217"       "GM15645"
[379] "GM15800"       "GM1604B"       "GM16501"       "GM16510"       "GM1966"        "GM2044"
[385] "GM2437"        "GM2694"        "GM2703"        "GM2744"        "GM3035"        "GM3051"
[391] "GM3181"        "GM3224"        "GM3265"        "GM3448"        "GM3455"        "GM3627"
[397] "GM3652"        "GM3806"        "GM3848"        "GM4013"        "GM4326"        "GM4653"
[403] "GM4871"        "GM4951"        "GM4955"        "GM5068"        "GM5069"        "GM5150"
[409] "GM5483"        "GM5523"        "GM5595"        "GM5935"        "GM5938"        "GM6132"
[415] "GM6293"        "GM6498"        "GM6934"        "GM6981"        "GM7193"        "GM7475"
[421] "GM813"         "GM826"         "GM8709"        "GM8884"        "GM8909"        "GM8995"
[427] "GM9706"        "GM9733"        "GNAI2"         "GNB2"          "GNGT2"         "GP1BA"
[433] "GP49A"         "GP5"           "GP6"           "GP9"           "GPR137B-PS"    "GPR157"
[439] "GPR31B"        "GPR56"         "GRWD1"         "GSDMC"         "GSDMD"         "GSR"
[445] "GSTT1"         "GTPBP3"        "GVIN1"         "GYG"           "H13"           "H19"
[451] "H2-BL"         "H2-D1"         "H2-K1"         "H2-K2"         "H2-M2"         "H2-OB"
[457] "H2-Q10"        "H2-Q2"         "H2-Q6"         "H2-Q7"         "H2-Q8"         "H2-T10"
[463] "H2-T23"        "H2-T24"        "H2-T9"         "HBB-BH2"       "HBQ1B"         "HCAR2"
[469] "HCK"           "HCLS1"         "HDAC4"         "HDC"           "HEBP1"         "HES7"
[475] "HEXIM2"        "HEYL"          "HGSNAT"        "HHAT"          "HIPK1"         "HIST1H3C"
[481] "HIST2H2AA1"    "HIST3H2BA"     "HMGCL"         "HMGN2"         "HMOX1"         "HPCAL1"
[487] "HSCB"          "HSD17B11"      "HSH2D"         "I830012O16RIK" "I830077J02RIK" "I830127L07RIK"
[493] "ICA1L"         "IFI202B"       "IFI203"        "IFI204"        "IFI27L2A"      "IFI47"
[499] "IFITM2"        "IFITM6"        "IFNA14"        "IFRD2"         "IGF1"          "IGFBP6"
[505] "IGHV14-2"      "IGSF6"         "IGSF9B"        "IGTP"          "IL18BP"        "IL1B"
[511] "IL1F9"         "IL1R2"         "IL4I1"         "IL6RA"         "ILK"           "IMPG1"
[517] "INF2"          "IRF1"          "IRF6"          "IRG1"          "IRGM1"         "IRGM2"
[523] "ITGA6"         "ITGAL"         "ITGB1"         "ITGB5"         "ITM2B"         "JARID2"
[529] "JUN"           "KCND3"         "KCNN3"         "KLHL25"        "KLHL32"        "KLK1B4"
[535] "KLRA2"         "KPNA6"         "KRT6B"         "KRT73"         "LAMP1"         "LAMP2"
[541] "LANCL3"        "LARP1B"        "LCP1"          "LEFTY2"        "LGALS1"        "LGALS3"
[547] "LGALSL"        "LHX9"          "LIN52"         "LITAF"         "LOC100038947"  "LOC102636217"
[553] "LOC547349"     "LOC676689"     "LOC68395"      "LRG1"          "LRRC25"        "LRRTM1"
[559] "LSP1"          "LST1"          "LTB4R1"        "LTBP1"         "LTBR"          "LY6A"
[565] "LY6C1"         "LY6G6C"        "LY6K"          "LYZ1"          "LYZ2"          "MADCAM1"
[571] "MAGED2"        "MARCH7"        "MARCH8"        "MARCKS"        "MARCKSL1"      "MAST2"
[577] "MBD3"          "MBNL2"         "MBOAT7"        "MC3R"          "MCL1"          "MCUR1"
[583] "MEF2C"         "MEFV"          "MEIS3"         "MEMO1"         "MESP2"         "METAP2"
[589] "METRNL"        "METTL7A1"      "MFSD6"         "MGAT5B"        "MGMT"          "MGST1"
[595] "MIDN"          "MLANA"         "MMD"           "MMP17"         "MMP8"          "MNDA"
[601] "MOCOS"         "MOV10"         "MPC2"          "MPEG1"         "MPL"           "MRAP2"
[607] "MRAS"          "MRGBP"         "MRGPRA2A"      "MRGPRA2B"      "MRGPRA6"       "MRPL33"
[613] "MRVI1"         "MS4A4C"        "MS4A6C"        "MS4A6D"        "MS4A8A"        "MSGN1"
[619] "MSRA"          "MSRB1"         "MTMR14"        "MTMR3"         "MTUS1"         "MVB12A"
[625] "MYD88"         "MYL3"          "MYO1D"         "MYO1F"         "NAAA"          "NADK"
[631] "NADK2"         "NAIP2"         "NAMPT"         "NCF2"          "NCF4"          "NCK2"
[637] "NCOA1"         "NDEL1"         "NES"           "NFAM1"         "NFE2"          "NFIL3"
[643] "NFKBIA"        "NGFRAP1"       "NINJ1"         "NKIRAS2"       "NKX2-2"        "NLRC5"
[649] "NLRX1"         "NME1"          "NOC4L"         "NOD1"          "NOD2"          "NOMO1"
[655] "NOS1"          "NPRL3"         "NPTN"          "NRGN"          "NRON"          "NRROS"
[661] "NRTN"          "NUAK1"         "NUDT3"         "NUDT4"         "NUPR1"         "OAS1A"
[667] "OAS1C"         "OAS1F"         "OASL1"         "OASL2"         "OBOX1"         "ODC1"
[673] "OGDH"          "OGFR"          "OGT"           "OLFR1211"      "OLFR1466"      "OLFR209"
[679] "OLFR324"       "OLFR394"       "OLFR406"       "OLFR458"       "OLFR522"       "OLFR545"
[685] "OLFR631"       "OLFR635"       "OLFR700"       "OLFR704"       "ONECUT2"       "OSER1"
[691] "OSGIN1"        "OSTF1"         "OTOS"          "OTUD5"         "OTUD7B"        "OVCH2"
[697] "P2RY13"        "PABPC1"        "PABPC4L"       "PACSIN2"       "PAQR6"         "PARVB"
[703] "PCGF5"         "PCX"           "PDGFA"         "PDLIM7"        "PEX11G"        "PF4"
[709] "PFKFB4"        "PGA5"          "PGAM1"         "PGD"           "PGLYRP1"       "PGM5"
[715] "PHF11A"        "PHF11B"        "PHF11D"        "PHLDA3"        "PI16"          "PIK3R6"
[721] "PILRA"         "PILRB2"        "PIN1RT1"       "PIRA11"        "PIRA2"         "PIRA6"
[727] "PIRA7"         "PIRB"          "PKM"           "PLA2G7"        "PLAUR"         "PLD4"
[733] "PLEK"          "PLEKHG5"       "PLEKHO2"       "PLIN2"         "PLP1"          "PLXNA4"
[739] "PLXNB2"        "PNKP"          "PNLDC1"        "PNMA3"         "PNP"           "PODNL1"
```

```
[745] "POLR2A"      "POLR3H"      "POR"         "POU3F1"      "POU6F2"      "PPBP"
[751] "PPDPF"       "PPIF"        "PPOX"        "PPP1R14C"    "PPP1R15A"    "PPP1R2"
[757] "PPP1R3D"     "PRAM1"       "PRDX2"       "PRDX5"       "PRDX6"       "PREB"
[763] "PRKAB1"      "PRKAB2"      "PRKX"        "PRL2A1"      "PRMT7"       "PRND"
[769] "PROSER2"     "PRR5L"       "PRRG2"       "PRRT1"       "PSAP"        "PSMB10"
[775] "PSMB5"       "PSMB8"       "PTCHD1"      "PTGS1"       "PTK2B"       "PTP4A3"
[781] "PTPLAD2"     "PTPN1"       "PTPN11"      "PTPN18"      "PTPRD"       "PTPRH"
[787] "PTPRJ"       "PTRF"        "PTTG1IP"     "PYDC3"       "PYGL"        "PYHIN1"
[793] "QK"          "QSOX1"       "RAB31"       "RAB32"       "RAB3IL1"     "RAB44"
[799] "RALB"        "RAN"         "RANBP10"     "RARA"        "RASGRF2"     "RASGRP2"
[805] "RASGRP4"     "RB1"         "RBBP4"       "RBM19"       "RBM3"        "RBM33"
[811] "RBM47"       "RBPMS2"      "REN2"        "REP15"       "RETNLG"      "RFK"
[817] "RFXANK"      "RGS2"        "RGS20"       "RGS21"       "RGS3"        "RGS7"
[823] "RHOG"        "RHOU"        "RHOX7"       "RND1"        "RNF11"       "RNF114"
[829] "RNF149"      "RNF19B"      "RNF31"       "RNPEP"       "RSU1"        "RTN3"
[835] "S100A11"     "S100A4"      "SAA1"        "SAA3"        "SAMHD1"      "SBNO2"
[841] "SCAMP1"      "SCARB1"      "SCGB1A1"     "SCNN1A"      "SCRG1"       "SEC61B"
[847] "SELK"        "SELPLG"      "SEMA4A"      "SEMA6B"      "SENP3"       "SERPINA3G"
[853] "SERPINB6A"   "SERPINB6C"   "SFN"         "SFXN5"       "SH3BGRL3"    "SH3BP2"
[859] "SH3D21"      "SHISA9"      "SIGMAR1"     "SIRPA"       "SIRPB1A"     "SIRPB1B"
[865] "SKAP2"       "SLA"         "SLAMF1"      "SLC10A1"     "SLC11A1"     "SLC15A3"
[871] "SLC16A3"     "SLC22A1"     "SLC24A3"     "SLC25A51"    "SLC27A4"     "SLC28A2"
[877] "SLC2A3"      "SLC2A6"      "SLC38A5"     "SLC39A1"     "SLC40A1"     "SLC44A1"
[883] "SLC4A2"      "SLC6A1"      "SLC6A4"      "SLC7A7"      "SLFN1"       "SLFN10-PS"
[889] "SLFN13L"     "SLFN2"       "SLFN3"       "SLFN4"       "SLPI"        "SMC4"
[895] "SMIM3"       "SMPDL3A"     "SMTN"        "SNAP23"      "SNRNP25"     "SNX15"
[901] "SNX20"       "SNX32"       "SNX9"        "SOCS3"       "SOD2"        "SORD"
[907] "SOWAHA"      "SPAG17OS"    "SPEER1-PS1"  "SPEF1"       "SPI1"        "SPOP"
[913] "SPPL2A"      "SPRN"        "SPRR1A"      "SPTA1"       "SQRDL"       "SRM"
[919] "SRPX2"       "SRXN1"       "SSBP2"       "SSBP4"       "SSX9"        "SSXB9"
[925] "ST3GAL5"     "STARD3"      "STAT3"       "STFA1"       "STFA2"       "STFA2L1"
[931] "STFA3"       "STK40"       "SVIL"        "SYCN"        "TAAR4"       "TAB3"
[937] "TAGLN"       "TAL1"        "TAPBP"       "TAS2R131"    "TBC1D2"      "TBC1D22A"
[943] "TBKBP1"      "TBXAS1"      "TCSTV1"      "TCTE1"       "TCTEX1D2"    "TDRP"
[949] "TEAD1"       "TENM4"       "TFAP2D"      "TFDP2"       "TFE3"        "TGFB1"
[955] "TGFB1I1"     "TGIF2"       "TGM2"        "TGTP2"       "THBS1"       "THEMIS2"
[961] "TIMM10B"     "TIMM17B"     "TIMP2"       "TIRAP"       "TLDC2"       "TLN1"
[967] "TLR13"       "TLR2"        "TLR6"        "TM4SF5"      "TMA7"        "TMBIM1"
[973] "TMBIM6"      "TMEM116"     "TMEM178"     "TMEM219"     "TMEM246"     "TMEM256"
[979] "TMEM29"      "TMEM40"      "TMEM50B"     "TMEM87A"     "TMEM8C"      "TMOD4"
[985] "TMSB4X"      "TMTC3"       "TNFAIP2"     "TNFRSF1A"    "TNFRSF1B"    "TNFSF14"
[991] "TNP1"        "TOM1"        "TOR1AIP1"    "TOR1AIP2"    "TOR3A"       "TPD52"
[997] "TPI1"        "TPM1"        "TPM2"        "TPM3"
[ reached getOption("max.print") -- omitted 100 entries ]

$Tang
  [1] "A1BG"         "A2M-AS1"      "ABCA1"        "ABHD14B"      "ABLIM1"       "ACADSB"       "ACAT1"
  [8] "ACCS"         "ACKR3"        "ACOT13"       "ACOXL"        "ACRBP"        "ADAMTS10"     "ADARB1"
 [15] "ADCY4"        "ADRB2"        "AES"          "AKAP11"       "AKIRIN2"      "ALG13"        "AMIGO1"
 [22] "AMPD2"        "ANK3"         "ANKRD22"      "ANKRD36"      "ANKRD46"      "ANKS6"        "ANXA8L1"
 [29] "AP5B1"        "APBA2"        "APOBEC3B"     "APOBEC3H"     "AQP1"         "AQP10"        "ARG1"
 [36] "ARHGAP5"      "ARHGEF40"     "ARL17A"       "ARL4A"        "ARL4C"        "ARNTL"        "ARPC3"
 [43] "ARPC5"        "ASGR2"        "ASPH"         "ASPM"         "ATF1"         "ATP5E"        "ATP5I"
 [50] "ATP5J"        "ATP6"         "ATP6V0E2-AS1" "AUTS2"        "B4GALT5"      "BAALC"        "BACH2"
 [57] "BAHD1"        "BANK1"        "BATF2"        "BCL11A"       "BCL11B"       "BCL2A1"       "BIRC3"
 [64] "BIRC5"        "BLK"          "BRD1"         "BTG1"         "BTLA"         "BTN3A1"       "BUB1"
 [71] "BZRAP1-AS1"   "C10orf105"    "C12orf57"     "C16orf74"     "C1orf52"      "C20orf141"    "C2orf40"
 [78] "C2orf88"      "C4orf33"      "C5orf45"      "C6orf48"      "CA2"          "CACNA1E"      "CACNG6"
 [85] "CAMK1D"       "CAMP"         "CARD11"       "CARD16"       "CARD17"       "CARD6"        "CASC15"
 [92] "CASC5"        "CBLB"         "CBX7"         "CCDC102A"     "CCDC144A"     "CCDC147-AS1"  "CCNB1"
 [99] "CCNB2"        "CCR3"         "CCR7"         "CD164"        "CD177"        "CD19"         "CD2"
[106] "CD22"         "CD247"        "CD28"         "CD300E"       "CD3E"         "CD40LG"       "CD5"
[113] "CD6"          "CD7"          "CD74"         "CD79B"        "CD81"         "CD83"         "CD8A"
```

```
[120] "CD8B"        "CD96"        "CDC20"       "CDC42SE1"    "CDC45"       "CDCA2"       "CDCA3"
[127] "CDCA5"       "CDK1"        "CDK11A"      "CDK11B"      "CDKN2C"      "CDKN3"       "CDT1"
[134] "CDV3"        "CENPF"       "CENPM"       "CENPN"       "CENPU"       "CENPW"       "CEP19"
[141] "CES1"        "CFAP97"      "CHEK1"       "CHIT1"       "CHPT1"       "CHRM3-AS2"   "CISD2"
[148] "CIT"         "CKAP4"       "CKLF"        "CKS1B"       "CKS2"        "CLEC10A"     "CLEC11A"
[155] "CLEC12A"     "CLUHP3"      "CMTM5"       "CNIH4"       "CNR2"        "CNTNAP3"     "COQ10A"
[162] "COX17"       "COX7A2"      "COX7B"       "CR1"         "CR2"         "CREG1"       "CRHR1-IT1"
[169] "CRIP2"       "CROCC"       "CRTC1"       "CSGALNACT2"  "CSTA"        "CTNNAL1"     "CTSL"
[176] "CTTN"        "CXCR5"       "CYB561A3"    "CYSTM1"      "CYTH3"       "DCAF16"      "DDAH2"
[183] "DEFA8P"      "DENND2D"     "DHFR"        "DIAPH3"      "DISC1"       "DLGAP5"      "DNAJA1P5"
[190] "DNAJA3"      "DNAJC30"     "DNAJC5"      "DOCK10"      "DPP4"        "DPRXP4"      "DRC1"
[197] "DTL"         "DUSP13"      "DUT"         "DYRK2"       "E2F1"        "E2F7"        "ECRP"
[204] "EEF1A1"      "EEF2K"       "EIF1AX"      "EIF3F"       "EIF4A2"      "EIF4B"       "EIF4G3"
[211] "ENHO"        "ENO2"        "ENY2"        "EOMES"       "EPHX2"       "ERN1"        "ESPL1"
[218] "ESRG"        "EVL"         "EZH2"        "FABP5"       "FAIM3"       "FAM102A"     "FAM117B"
[225] "FAM129C"     "FAM134B"     "FAM155A-IT1" "FAM159A"     "FAM177B"     "FAM20A"      "FAM26F"
[232] "FAM27C"      "FANCF"       "FANCI"       "FBL"         "FBLN5"       "FBXL16"      "FCAR"
[239] "FCER1A"      "FCER2"       "FCGBP"       "FCGR1A"      "FCN1"        "FCRL1"       "FCRL2"
[246] "FCRL3"       "FCRL6"       "FCRLA"       "FFAR3"       "FGD4"        "FLT3LG"      "FOXJ3"
[253] "FOXM1"       "FOXO1"       "FRMD3"       "FSD1"        "FSTL3"       "FTL"         "G0S2"
[260] "GADD45A"     "GADD45G"     "GALM"        "GALNT14"     "GATA3"       "GCSAM"       "GDF15"
[267] "GINS1"       "GINS2"       "GLCCI1"      "GLIPR2"      "GLS"         "GLTSCR2"     "GMFG"
[274] "GMNN"        "GNB2L1"      "GNG10"       "GOLGA8A"     "GPA33"       "GPD2"        "GPER1"
[281] "GPR141"      "GPR174"      "GPR18"       "GPR183"      "GPR68"       "GPR84"       "GPRASP1"
[288] "GRB10"       "GRPEL2"      "GSTM3"       "GTF2H5"      "GTSE1"       "GVINP1"      "GYG1"
[295] "GZMK"        "GZMM"        "H1F0"        "HABP4"       "HAPLN3"      "HBM"         "HCG18"
[302] "HELZ2"       "HES6"        "HIP1R"       "HIST1H1B"    "HIST1H2AC"   "HIST1H2AD"   "HIST1H2AE"
[309] "HIST1H2AH"   "HIST1H2AI"   "HIST1H2AJ"   "HIST1H2AL"   "HIST1H2AM"   "HIST1H2BB"   "HIST1H2BC"
[316] "HIST1H2BD"   "HIST1H2BE"   "HIST1H2BF"   "HIST1H2BG"   "HIST1H2BH"   "HIST1H2BI"   "HIST1H2BJ"
[323] "HIST1H2BK"   "HIST1H2BL"   "HIST1H2BM"   "HIST1H2BO"   "HIST1H3B"    "HIST1H3D"    "HIST1H3F"
[330] "HIST1H3H"    "HIST1H3J"    "HIST1H4D"    "HIST1H4F"    "HIST1H4K"    "HIST1H4L"    "HIST2H2AA4"
[337] "HIST2H2AB"   "HIST2H3A"    "HJURP"       "HLA-DOA"     "HLA-DOB"     "HLA-DPA1"    "HLA-DPB1"
[344] "HLA-DPB2"    "HLA-DQA2"    "HLA-DQB1"    "HLA-DQB2"    "HLA-DRA"     "HLA-DRB3"    "HMGB1"
[351] "HMGB3P1"     "HMMR"        "HNRNPA1"     "HNRNPH1"     "HOXB2"       "HP1BP3"      "HPR"
[358] "HRASLS2"     "HS3ST1"      "HSP90AA1"    "ICOS"        "IFFO2"       "IFI27L1"     "IFI27L2"
[365] "IFITM4P"     "IFT20"       "IGFBP7"      "IGJ"         "IGLL1"       "IGLL5"       "IKZF2"
[372] "IL10RB-AS1"  "IL11RA"      "IL18R1"      "IL18RAP"     "IL1RAP"      "IL23A"       "IL27"
[379] "IL2RB"       "IL32"        "IL7R"        "IMP3"        "ING5"        "INHBA"       "INPP4B"
[386] "INPP5E"      "IRAK3"       "ISCA1"       "ISY1"        "ITGA7"       "ITIH4"       "ITK"
[393] "JDP2"        "JPH4"        "KCNG1"       "KDF1"        "KIAA0101"    "KIAA0226L"   "KIAA0319L"
[400] "KIAA0355"    "KIAA1147"    "KIF11"       "KIF1B"       "KIF2C"       "KLF12"       "KLHDC7B"
[407] "KLHDC8B"     "KLHL3"       "KLRB1"       "KLRC3"       "KLRC4"       "KLRG1"       "KRTAP10-6"
[414] "LAT"         "LBH"         "LCK"         "LDLRAP1"     "LEF1-AS1"    "LEPROTL1"    "LILRA3"
[421] "LILRA5"      "LILRB3"      "LIN7A"       "LIPN"        "LLGL2"       "LMF1"        "LOXL3"
[428] "LPIN1"       "LRFN3"       "LRIG1"       "LRRC47"      "LRRN3"       "LSM3"        "LTB4R"
[435] "LTBP3"       "LTF"         "LY9"         "LY96"        "MAL"         "MAMSTR"      "MAN1C1"
[442] "MAOA"        "MAP1LC3B"    "MAPK14"      "MATK"        "MB21D1"      "MCF2L-AS1"   "MCM2"
[449] "MCM4"        "MCTP1"       "MEAF6"       "MEGF6"       "MEX3C"       "MEX3D"       "MGAM"
[456] "MGC16025"    "MGLL"        "MILR1"       "MIR146A"     "MIR4435-1HG" "MKI67"       "MLLT6"
[463] "MME"         "MOAP1"       "MPO"         "MRC2"        "MRPL22"      "MRPL27"      "MRPL51"
[470] "MRPS18C"     "MS4A1"       "MSN"         "MSX2P1"      "MT1B"        "MT1E"        "MT1HL1"
[477] "MT1L"        "MT1M"        "MTA1"        "MXD4"        "MXI1"        "MXRA7"       "MYBL1"
[484] "MYBL2"       "MYBPC3"      "MYC"         "MYCL"        "MYL6"        "MYL6B"       "NAB2"
[491] "NAIP"        "NAP1L1"      "NCR3"        "ND6"         "NDST2"       "NDUFA1"      "NDUFA4"
[498] "NDUFA6"      "NDUFB3"      "NELL2"       "NEXN"        "NKTR"        "NKX3-1"      "NLRC3"
[505] "NLRC4"       "NMUR1"       "NOP10"       "NRIR"        "NSG1"        "NT5C3A"      "NT5E"
[512] "NT5M"        "NTNG2"       "NTSR1"       "NUSAP1"      "OBFC1"       "ODF3B"       "OIP5"
[519] "OPLAH"       "OR52K2"      "OSCAR"       "OXNAD1"      "P2RX5"       "P2RY10"      "PASK"
[526] "PCED1B"      "PCMT1"       "PCNA"        "PCSK1N"      "PDE4DIP"     "PDP1"        "PEBP1"
[533] "PHC3"        "PHF19"       "PID1"        "PIK3AP1"     "PIK3C2B"     "PIN4"        "PIWIL4"
[540] "PJA1"        "PKIA"        "PKMYT1"      "PLBD1"       "PLCG1"       "PLEKHA1"     "PLEKHB1"
[547] "PLEKHG4"     "PLXDC1"      "PLXNA1"      "PMEPA1"      "PNMA1"       "PNRC1"       "POLG2"
[554] "POLRMT"      "PP7080"      "PPARD"       "PPIB"        "PQLC1"       "PRC1"        "PRDX4"
```

```
[561] "PREPL"      "PRKAG2-AS1" "PRKAR1B"    "PRKAR2B"    "PRKCA"      "PRKCH"      "PRKCQ-AS1"
[568] "PRNP"       "PRPF8"      "PRRG4"      "PRUNE2"     "PSIP1"      "PSMA2"      "PSMA3"
[575] "PSMA4"      "PSMA6"      "PSMC2"      "PSMF1"      "PTCRA"      "PTGDR"      "PTGDR2"
[582] "PTPRK"      "PTTG1"      "PTTG2"      "PTX3"       "PURA"       "PYCR1"      "RAB11FIP3"
[589] "RAB13"      "RABL2A"     "RAD21"      "RAD51"      "RAD54L"     "RAI1"       "RALGAPA1"
[596] "RARA-AS1"   "RASGRP1"    "RBM20"      "RBX1"       "RCAN3"      "REC8"       "RECQL4"
[603] "RETN"       "RFTN1"      "RGL4"       "RHOH"       "RMI2"       "RNA5-8S5"   "RNASE1"
[610] "RNF208"     "RNF32"      "RNF44"      "RORC"       "RPA3"       "RPL10A"     "RPL10L"
[617] "RPL13"      "RPL13A"     "RPL15"      "RPL18"      "RPL18A"     "RPL19P12"   "RPL22"
[624] "RPL22L1"    "RPL23A"     "RPL23AP7"   "RPL26L1"    "RPL29"      "RPL29P2"    "RPL3"
[631] "RPL4"       "RPL5"       "RPL7A"      "RPLP2"      "RPS13"      "RPS14"      "RPS16"
[638] "RPS18"      "RPS2"       "RPS23"      "RPS27A"     "RPS27L"     "RPS28"      "RPS2P32"
[645] "RPS6KA5"    "RPSAP9"     "RRAGD"      "RRAS2"      "RRBP1"      "RSAD1"      "RSPH9"
[652] "RTN1"       "RUNX3"      "RYK"        "S100A12"    "S100P"      "S100PBP"    "S1PR1"
[659] "SACS"       "SAMD3"      "SAMSN1"     "SAP30"      "SARDH"      "SARM1"      "SBK1"
[666] "SCAI"       "SCARF1"     "SCARNA17"   "SCML4"      "SDC1"       "SEC11C"     "SELM"
[673] "SEMA4C"     "Sep 01"     "SERTAD2"    "SF3B6"      "SFI1"       "SGK223"     "SH2D1B"
[680] "SH2D3A"     "SH3YL1"     "SHCBP1"     "SIDT1"      "SIGLEC17P"  "SIGLEC5"    "SIPA1L2"
[687] "SIT1"       "SKAP1"      "SLC1A3"     "SLC22A15"   "SLC22A4"    "SLC25A6"    "SLC26A11"
[694] "SLC26A8"    "SLC38A1"    "SLC45A3"    "SLC6A19"    "SLED1"      "SLIRP"      "SMA4"
[701] "SMARCD3"    "SMC1A"      "SMPD3"      "SNAR-B2"    "SNAR-D"     "SNAR-H"     "SNHG6"
[708] "SNORA54"    "SNORD105B"  "SNORD46"    "SNORD68"    "SNRPD1"     "SNRPG"      "SNX22"
[715] "SPAG5"      "SPC24"      "SPC25"      "SPCS3"      "SPIB"       "SPOCK2"     "SRGAP2B"
[722] "SRP19"      "SRSF10"     "SRSF8"      "ST6GAL1"    "STAB1"      "STAC3"      "STAT4"
[729] "STK26"      "STMN3"      "STOM"       "STRBP"      "SUB1"       "SUN1"       "SUSD6"
[736] "TAGAP"      "TANK"       "TC2N"       "TCF7"       "TCL1A"      "TCTN1"      "TFDP1"
[743] "THRA"       "TIGD3"      "TIMM8B"     "TK1"        "TLE2"       "TLR5"       "TMCC1"
[750] "TMEM106B"   "TMEM109"    "TMEM119"    "TMEM204"    "TMEM209"    "TMEM243"    "TMEM25"
[757] "TMEM263"    "TMEM42"     "TNFRSF13C"  "TNFRSF17"   "TNFRSF25"   "TOP1MT"     "TP53I3"
[764] "TPST1"      "TPX2"       "TRABD2A"    "TRAF1"      "TRAF5"      "TRAK1"      "TRG-AS1"
[771] "TRGV7"      "TRIM34"     "TRIM4"      "TRIM54"     "TROAP"      "TSHZ1"      "TSPAN3"
[778] "TSPYL4"     "TTC3"       "TUBE1"      "TXN"        "TXNDC5"     "TYMS"       "UBASH3A"
[785] "UBE2L3"     "UBE2Q2"     "UBE2T"      "UBE2V1"     "UCHL1"      "UHRF1"      "UNCX"
[792] "UPP1"       "UQCRB"      "UQCRQ"      "USP41"      "VEGFA"      "VEGFB"      "VNN1"
[799] "VPS13C"     "VPS37C"     "WDR86"      "WFDC3"      "ZAP70"      "ZBED6CL"    "ZBTB11"
[806] "ZBTB32"     "ZBTB4"      "ZBTB40"     "ZCCHC14"    "ZDHHC11"    "ZFP62"      "ZFYVE28"
[813] "ZMAT1"      "ZNF12"      "ZNF204P"    "ZNF205"     "ZNF286B"    "ZNF529"     "ZNF550"
[820] "ZNF684"     "ZNF831"     "ZNF837"     "ZNF90"      "ZSCAN18"    "ZSWIM7"     "ZWINT"
[827] "ZXDB"

$Woods
  [1] "ABAT"                                           "ABCA2"
  [3] "ABCC6"                                          "ACOT9"
  [5] "ADAMTS5"                                        "AFF1"
  [7] "ANPEP"                                          "AP1M2"
  [9] "APOBEC3G"                                       "APOL1"
 [11] "ARHGAP32"                                       "ARL3"
 [13] "ART3"                                           "ATF3"
 [15] "ATP2B2"                                         "ATP6V1A"
 [17] "BAGE"                                           "BATF3"
 [19] "BPY2"                                           "BRCA2"
 [21] "C10orf84"                                       "C11orf75"
 [23] "C18orf25"                                       "C19orf66"
 [25] "C1GALT1"                                        "C1orf106"
 [27] "C22orf28"                                       "C9orf91"
 [29] "CA4"                                            "CACNA1A"
 [31] "CASP5"                                          "CASS4"
 [33] "CCL2"                                           "CCL8"
 [35] "CCNA1"                                          "CCR1"
 [37] "CD2AP"                                          "CDKN1A"
 [39] "CDKN1C"                                         "CENPI"
 [41] "CFTR"                                           "CMKLR1"
 [43] "CNP"                                            "COBLL1"
```

```
 [45] "CPB1"                                                    "CPEB3"
 [47] "CRYAB"                                                   "CST4"
 [49] "CT62"                                                    "CWH43"
 [51] "CXCL6"                                                   "CXorf21"
 [53] "DAAM2"                                                   "DDO"
 [55] "DNAJC15"                                                 "DPEP3"
 [57] "DUSP5"                                                   "DUX1"
 [59] "EGR2"                                                    "ENPP2"
 [61] "EPB41L3"                                                 "EPB41L4A"
 [63] "EPHA4"                                                   "EPHB2"
 [65] "EPS8"                                                    "FAM70A"
 [67] "FANCA"                                                   "FANCL"
 [69] "FCGR1A /// FCGR1C"                                       "FCGR2B"
 [71] "FFAR2"                                                   "FTSJD2"
 [73] "FUT4"                                                    "FZD5"
 [75] "GALNT3"                                                  "GCH1"
 [77] "GK"                                                      "GOLGA6L4 /// PML"
 [79] "GRAMD1C"                                                 "GSTA1"
 [81] "GTPBP2"                                                  "GUCA1A"
 [83] "HBE1"                                                    "HESX1"
 [85] "HEY1"                                                    "HFE"
 [87] "HGF"                                                     "ICA1"
 [89] "ID3"                                                     "IDO1"
 [91] "IDS"                                                     "IGF2R"
 [93] "IL10"                                                    "IL15"
 [95] "IRS2"                                                    "ITGB4"
 [97] "JUP"                                                     "KAZ"
 [99] "KCND1"                                                   "KCNK10"
[101] "KCTD14"                                                  "KIAA0226"
[103] "KIAA1324"                                                "KIT"
[105] "KMO"                                                     "KPTN"
[107] "KSR1"                                                    "KYNU"
[109] "LCAT"                                                    "LEP"
[111] "LOC100505584 /// MT1E"                                   "LOC51145"
[113] "LOC652346 /// PML"                                       "LPAR1"
[115] "LPAR6"                                                   "LPHN3"
[117] "LTK"                                                     "MAP7"
[119] "MAST3"                                                   "MDC1"
[121] "MDK"                                                     "MET"
[123] "METT10D"                                                 "METTL7A"
[125] "MGP"                                                     "MICB"
[127] "MR1"                                                     "MSR1"
[129] "MT1F"                                                    "MT1G"
[131] "MT1H"                                                    "MT1P2"
[133] "MT1X"                                                    "MTHFD2"
[135] "MTHFR"                                                   "MTTP"
[137] "MYO7A"                                                   "MYOF"
[139] "MZF1"                                                    "NECAB2"
[141] "NEUROD2"                                                 "NF2"
[143] "NFATC4"                                                  "NR2F6"
[145] "NR4A2"                                                   "NSUN7"
[147] "NTRK3"                                                   "OMD"
[149] "OSBPL7"                                                  "P2RX7"
[151] "P2RY14"                                                  "P2RY6"
[153] "PADI4"                                                   "PARP11"
[155] "PBXIP1"                                                  "PDZD8"
[157] "PEX7"                                                    "PGAP1"
[159] "PLEKHG3"                                                 "PLOD2"
[161] "POLR3G"                                                  "PPY2"
[163] "PRB4"                                                    "PRKDC"
[165] "PSG2"                                                    "PTP4A1"
[167] "PTPRO"                                                   "RAB27B"
[169] "RANBP1"                                                  "RBCK1"
```

```
[171] "RBM17"                                         "RBMS2"
[173] "RCN3"                                          "RFX2"
[175] "RGL1"                                          "RHAG"
[177] "RHBDF1"                                        "RIN2"
[179] "RPGRIP1"                                       "RRAS"
[181] "SCAND2"                                        "SCN11A"
[183] "SDC2"                                          "SEC24D"
[185] "SEC61A2"                                       "SECTM1"
[187] "SEPP1"                                         "SEPT4"
[189] "SLAMF7"                                        "SLC12A8"
[191] "SLC25A24"                                      "SLC27A3"
[193] "SLC38A3"                                       "SLC5A7"
[195] "SLC6A2"                                        "SLFN12"
[197] "SNRNP70"                                       "SOBP"
[199] "SOX3"                                          "SOX4"
[201] "SPTAN1"                                        "SRBD1"
[203] "SYCP1"                                         "SYN2"
[205] "TAP2"                                          "TAZ"
[207] "TLR7"                                          "TMEM62"
[209] "TMX1"                                          "TNIP3"
[211] "TREX1"                                         "TRIM21"
[213] "TSHR"                                          "UBE4B"
[215] "VAMP4"                                         "VRK2"
[217] "WSB2"                                          "XAGE1A /// XAGE1B /// XAGE1C /// XAGE1D /// XAGE1E"
[219] "ZNF248"

$Zhai
 [1] ""             "ADIPOR1"      "ALPL"         "ASCC2"        "BSG"          "CECR1"        "CSDA"
 [8] "CX3CR1"       "DPYSL5"       "ECGF1"        "EEF1G"        "EPB42"        "FBXO7"        "GIMAP4"
[15] "GPR175"       "GSPT1"        "GYPC"         "HBD"          "HBG1"         "HBG2"         "HIST2H2AA3"
[22] "HLA-F"        "ICAM3"        "IMPA2"        "LOC100008589" "LOC100128326" "LOC100129681" "LOC100130914"
[29] "LOC100131164" "LOC100131726" "LOC100132394" "LOC100134530" "LOC100134634" "LOC389386"    "LOC389599"
[36] "LOC401357"    "LOC440313"    "LOC440359"    "LOC642357"    "LOC642469"    "LOC643319"    "LOC643384"
[43] "LOC644852"    "LOC645173"    "LOC648390"    "LOC729021"    "LOC729660"    "LOC730286"    "MAP1S"
[50] "MT1A"         "MUC6"         "MYL12A"       "NT5C3"        "PI3"          "PPM1F"        "PRIC285"
[57] "RBM38"        "RPS5"         "SELENBP1"     "SERPINA13"    "SIGLEC14"     "SLC25A37"     "SLC25A39"
[64] "SNCA"         "SORL1"        "SPRYD3"       "STRADB"       "TBL1X"        "TSPAN5"       "TXNDC12"
[71] "TYMP"         "UBXN6"        "WDR40A"

$CC_svre_ctrTang
 [1] "AIF1"       "ANKRD9"     "ANP32E"     "ATOX1"      "B2M"        "CAP1"       "CCNA2"      "CD274"      "CD63"
[10] "CDCA8"      "CLEC1B"     "CLEC4D"     "CLU"        "CTSF"       "DBI"        "DHRS3"      "DTX3L"      "EVI2A"
[19] "FXYD5"      "GBP2"       "GBP3"       "GGH"        "GNG11"      "GP1BB"      "GRN"        "GSN"        "HEBP2"
[28] "HIST1H2AB"  "HIST1H2AG"  "HIST1H2AK"  "HK3"        "HMGB2"      "HSPA8"      "HVCN1"      "ITGA2B"     "ITGB3"
[37] "LCN2"       "LILRA6"     "MCEMP1"     "MXD1"       "MYL9"       "MZB1"       "NDRG2"      "NIPA1"      "NOG"
[46] "PABPC4"     "PLVAP"      "RASA4"      "S100A6"     "S100A9"     "SELL"       "SRGAP2"     "SRGN"       "STIL"
[55] "STMN1"      "TGFBI"      "TNNT1"      "TPM4"       "UBE2C"      "VIM"        "VPREB3"     "VPS51"

$CC_svre_ctrWoods
 [1] "APOBEC1"  "CBR1"     "CFB"      "DUSP3"    "GADD45B"  "GUCY1B3"  "HPSE"     "LGALS3BP" "MMP9"     "MMRN1"
[11] "MYH9"     "PSMB9"    "SDC3"     "SORT1"    "TDRD7"    "TRAFD1"

$CC_svre_ctrZhai
[1] "ADAR"    "BCL2L1"  "GBP4"    "GBP5"    "GZMB"    "HAGH"    "TAGLN2"  "TESC"    "TMEM140"

$TangWoods
 [1] "AIM2"     "ANXA3"    "APOBEC3A" "APOL6"    "C1QA"     "C2"       "C3AR1"    "CCRL2"    "CXCR3"    "EPN2"
[11] "ETV7"     "FHL2"     "FKBP5"    "GYPA"     "HIST1H4H" "IFIT5"    "ITPR3"    "LEF1"     "LHFPL2"   "MAP2K6"
[21] "MARCO"    "MS4A4A"   "MT2A"     "NDC80"    "NMI"      "NOV"      "PML"      "PTGER4"   "SAMD4A"   "SGK1"
[31] "SIGLEC1"  "SP100"    "SPTLC2"   "SSB"      "TCN1"     "TFEC"     "THBD"     "TMEM51"   "TOR1B"    "TRIM14"
[41] "ZC3HAV1"  "ZCCHC2"

$TangZhai
```

```
 [1] "ADM"      "DDX60L"   "DYNLT1"   "EEF2"     "EIF3L"    "ELF1"     "EMR3"     "EPSTI1"   "HES4"     "IFI30"
[11] "NCOA7"    "RNASE2"   "RPS3"     "RPS4X"    "SHISA5"   "TNFSF13B"

$WoodsZhai
[1] "CTSL1"   "DRAP1"   "LAMP3"   "MAFB"    "PHF11"   "TMEM123" "UBE2L6"  "WARS"

$CC_svre_ctrTangWoods
 [1] "AGRN"   "C1QB"   "CD38"   "CLIC4"  "DDX58"  "HP"     "LILRB4" "LMNB1"  "RECK"   "SOCS1"  "SP140"  "TCN2"   "UBE2S"

$CC_svre_ctrTangZhai
 [1] "CASP1"  "CMPK2"  "FBXO6"  "IFITM3" "IRF9"   "PARP10" "PARP14" "PARP9"  "RNF213" "S100A8" "SAMD9L"

$CC_svre_ctrWoodsZhai
[1] "CXCL10" "DPEP2"  "LGALS9" "LMO2"   "MX2"    "PARP12" "PSME2"  "SP110"  "STAT2"

$TangWoodsZhai
 [1] "BLVRA"   "CEACAM1" "CHMP5"   "DDX60"   "DHRS9"   "FAM46A"  "FCGR1B"  "GBP1"    "GLRX"    "HERC5"   "HERC6"
[12] "IFI16"   "IFI44L"  "IFI6"    "IL1RN"   "ISG20"   "LAP3"    "OAS1"    "OASL"    "OTOF"    "RSAD2"   "SAMD9"
[23] "SCARB2"  "SCO2"    "SPATS2L" "STAT1"   "TIMM10"  "TNFAIP6" "TNFSF10" "TRIM22"  "TRIM5"   "VAMP5"

$CC_svre_ctrTangWoodsZhai
 [1] "BST2"     "DHX58"    "EIF2AK2"  "IFI27"    "IFI35"    "IFI44"    "IFIH1"    "IFIT1"    "IFIT2"    "IFIT3"
[11] "IFITM1"   "IRF7"     "ISG15"    "LY6E"     "MX1"      "OAS2"     "OAS3"     "PLAC8"    "PLSCR1"   "RTP4"
[21] "SAT1"     "SERPING1" "TAP1"     "USP18"    "XAF1"     "ZBP1"
```
